# Program and Abstracts from the Canadian Digestive Diseases Week™ 2016

**DOI:** 10.1155/2016/4792898

**Published:** 2016-02-23

**Authors:** Canadian Association of Gastroenterology, Canadian Association for the Study of the Liver

**Affiliations:** ^1^Canadian Association of Gastroenterology (CAG), Canada; ^2^Canadian Association for the Study of the Liver, Canada

*Background*. Increased intestinal permeability (IP) has been observed in a number of autoimmune diseases. Our recent study has demonstrated that the host genetic and intestinal microbial composition has a limited influence on IP while smoking status and age as two important factors contributing to IP.

*Aims*. To investigate if demographic factors, environmental factors or bacterial functions are associated with intestinal permeability.

*Methods*. IP was measured with high-pressure liquid chromatography by timed urine collection after ingestion of an oral load of two saccharide probes, lactulose and mannitol. For each subject, the lactulose-mannitol ratio (LacMan ratio) was calculated as the fractional excretion of lactulose divided by that of mannitol. Bacterial DNA extracted from the stool of 1098 healthy subject was sequenced for the V4 hypervariable regions of the 16S rRNA using the Illumina MiSeq platform. The function of the fecal microbial communities was then imputed using PICRUSt V0.1 after a rarefaction step to 30,000 sequences per sample. The PICRUSt pre-calculated table of gene counts based on OTUs was used to identify the gene counts in the organisms present in the stool samples. The Kyoto Encyclopedia of Genes and Genomes (KEGG) and clusters of orthologous groups (COG) databases were used to identify gene families. Association was performed using a linear regression controlling for age, gender and smoking status. Bacterial functions with a mean count <10 were excluded.

*Results*. A total of 65 demographic and environmental factors were analyzed. We found that individuals currently living with a dog had higher IP (*p* = 9.6 × 10^−4^). However this association was temporary as dog exposure within younger age classes but not currently exposed shows no evidence of association. Living with other types of animals aside from dogs did not show an association with IP. Among 3,773 KEGG and 3,618 COG functions, we found several nominal associations with IP, the most significant being involved in tyrosine metabolism and degradation of aromatic compounds (K01826), possibly involved in tellurite resistance (COG3615), and DNA uptake process and recombination (COG4469) (*p* < 7.78 × 10^−4^).

*Conclusions*. Multivariate analysis controlling for major contributing factors to IP allowed us to identify that individuals currently living with a dog had increased IP. In addition, while the specific microbial taxa do not appear to be associated with IP, microbial community functions are likely contributing to IP in healthy humans. These results indicate the importance of environmental influences on IP.

Submitted on behalf of GEM Project research team.

*Funding Agencies: *CAG, CIHR

## Figures and Tables

**Figure 1:**
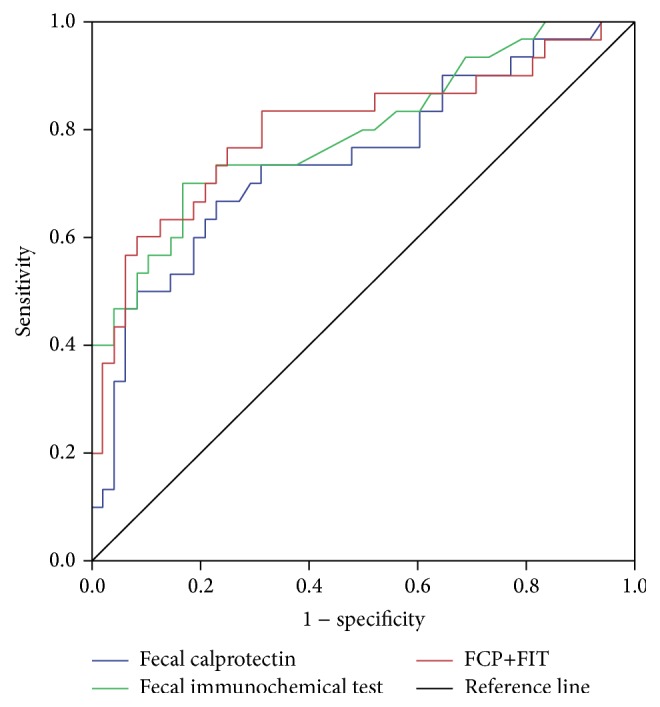
Receiver operator curves for fecal calprotectin (FCP), fecal immunochemical test (FIT), and combined FCP + FIT in the prediction of mucosal healing in 80 IBD patients presenting for colonoscopy.

**Figure 2:**
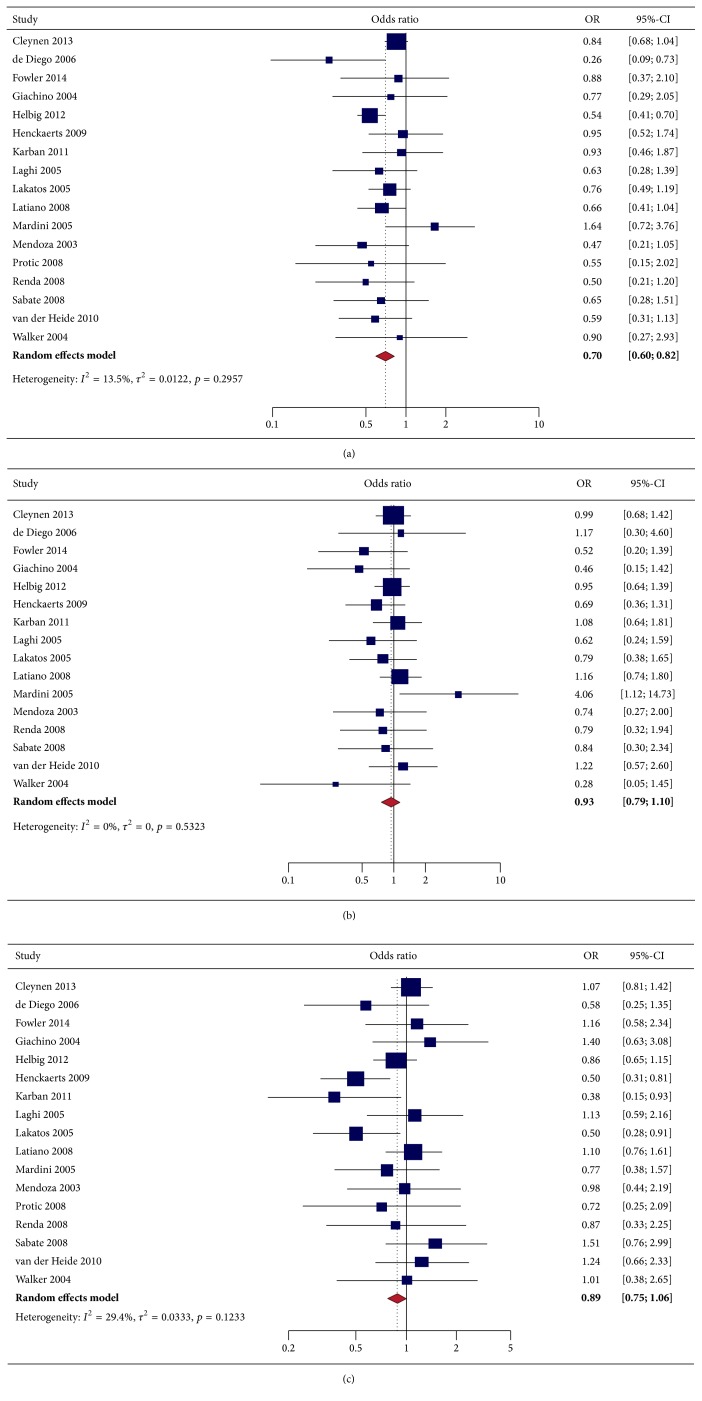
Forest plot depicting NOD2-smoking interaction among patients with Crohn's disease for the following SNPs: (a) 1007fs; (b) G908R; and (c) R702W.

**Figure 3:**
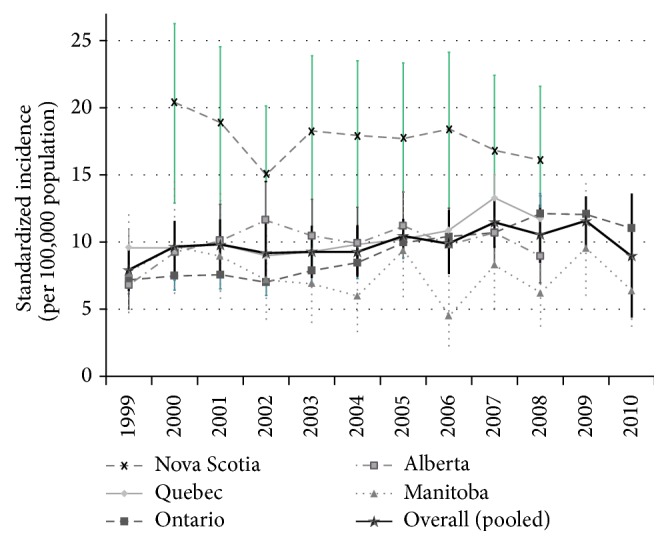
Incidence of PIBD over time in Canada.

**Figure 4 fig4:**
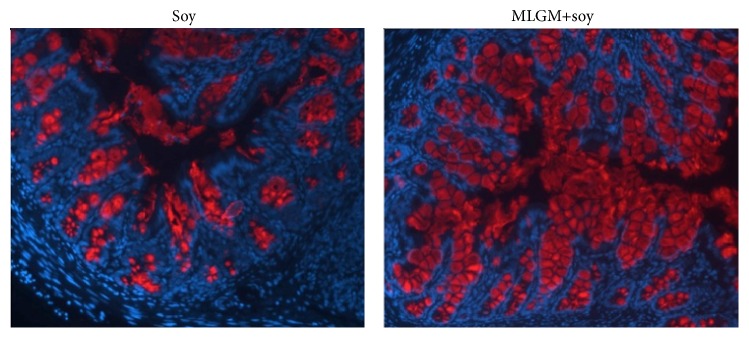
Representative immunohistochemical staining of the mucin (Muc2) in the distal colon of soy or MLGM + soy formula supplemented rat pups at post-natal day 15. (Magnification: 100x, DAPI-blue, Muc2 (appear as circular granules within crypts and in lumen)-red).

**Figure 5:**
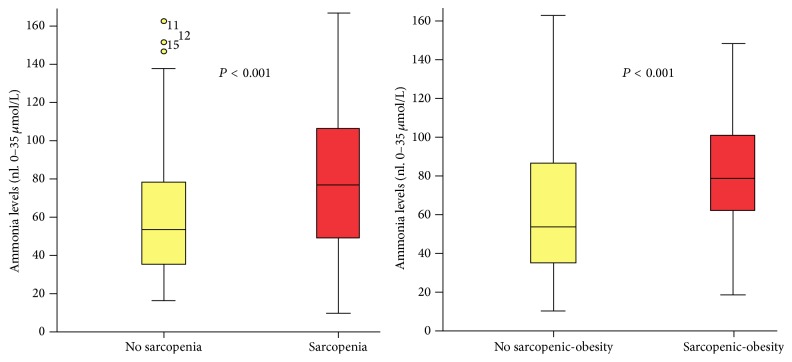


**Figure 6:**
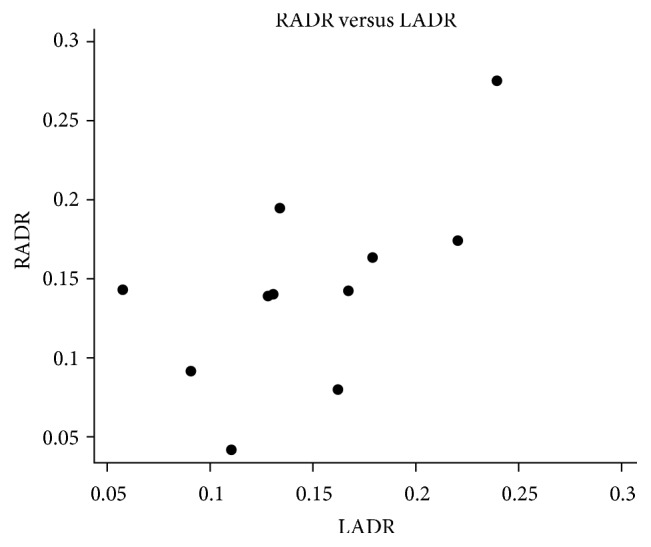


**Figure 7:**
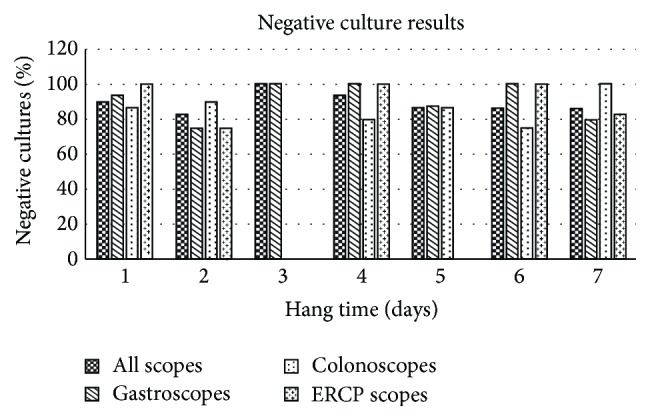
Percentage of negative cultures obtained for all endoscopes throughout the test period. The large percentage of negative cultures is fairly consistent from 1 to 7 days of hang time and between the different types of scopes.

**Figure 8:**
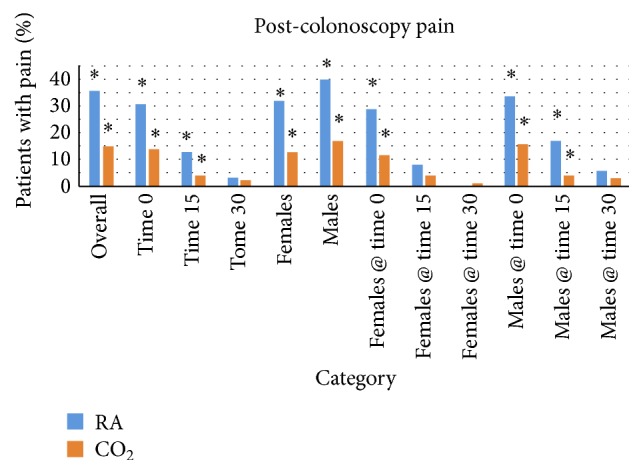
Post-colonoscopy pain experienced by patients at various time points of recovery, where ⁎ indicates statistical significance at 95% confidence interval.

**Figure 9 fig9:**
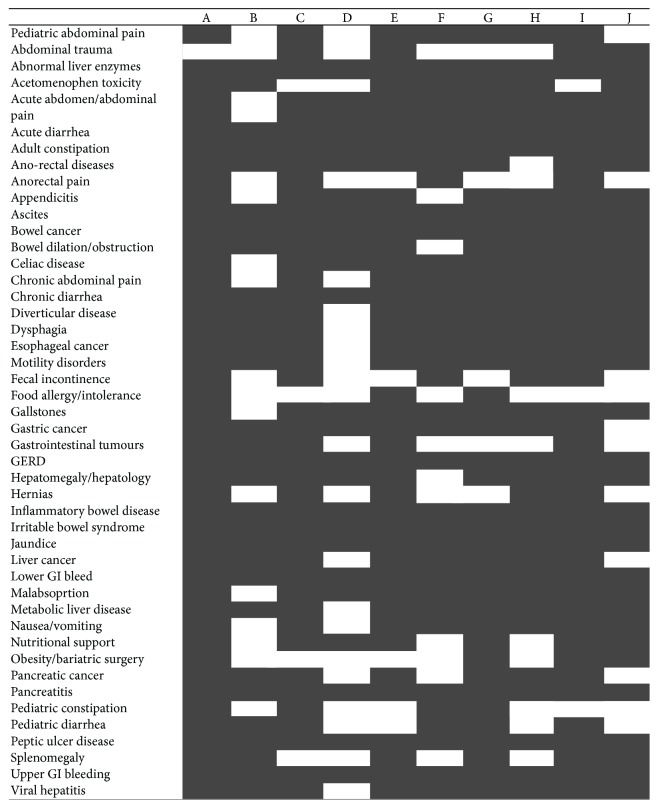
Gastroenterology curriculum map at candian medical school.

**Figure 10 fig10:**
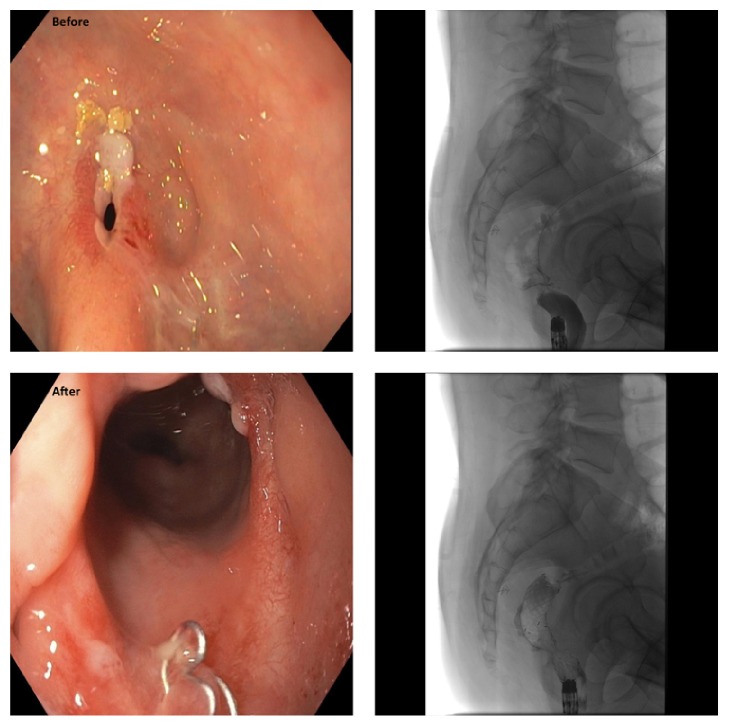
Endoscopic & fluroscopic pictures of the anastomotic stricture before & after stent insertion.

**Figure 11 fig11:**
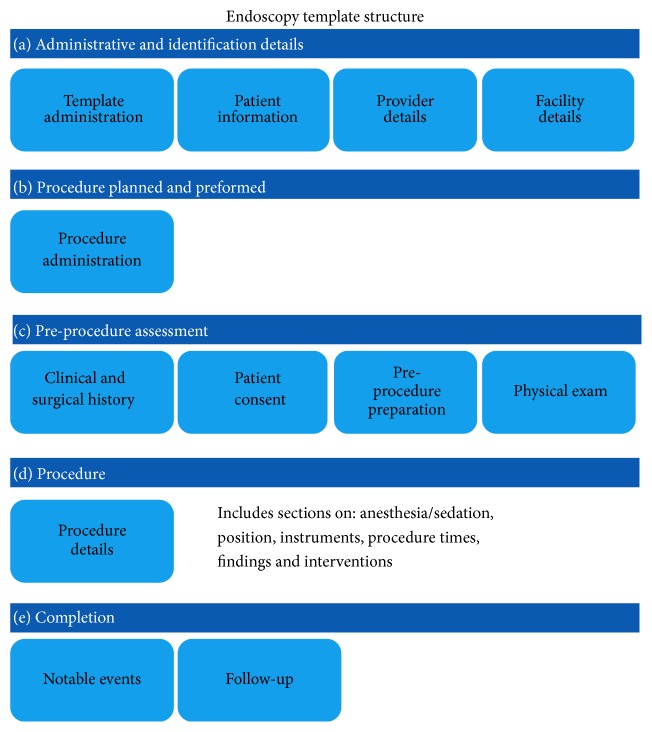


**Figure 12:**
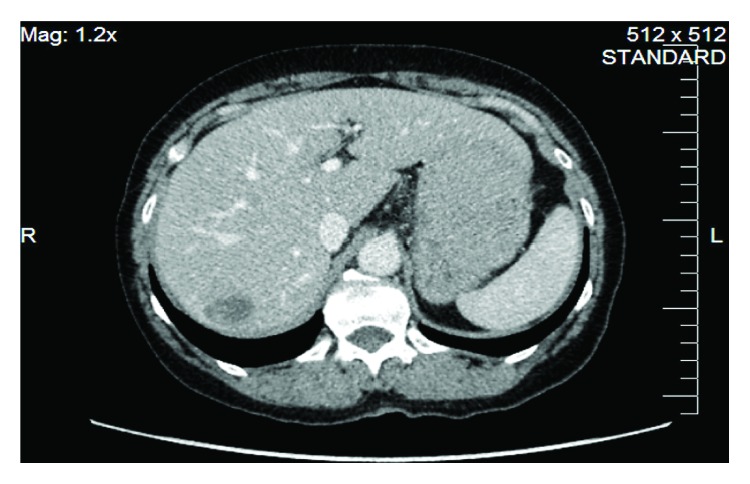
Abdominal CT showing 2.9 cm mass in liver segment 6/7.

**Figure 13 fig13:**
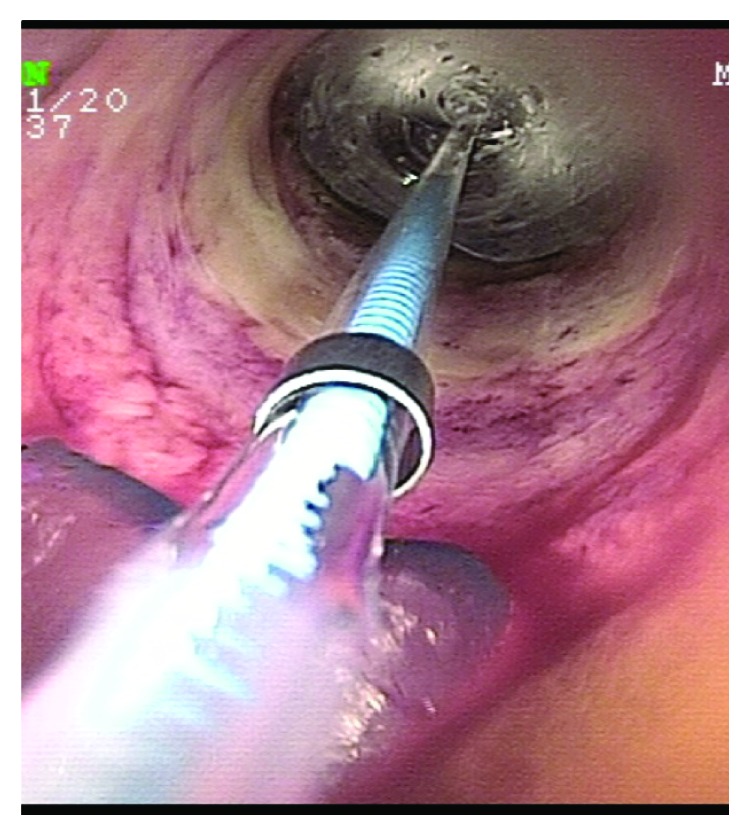
Endoscopic balloon dilation of a strictured small bowel in a patient with small bowel Crohn's disease.

**Figure 14:**
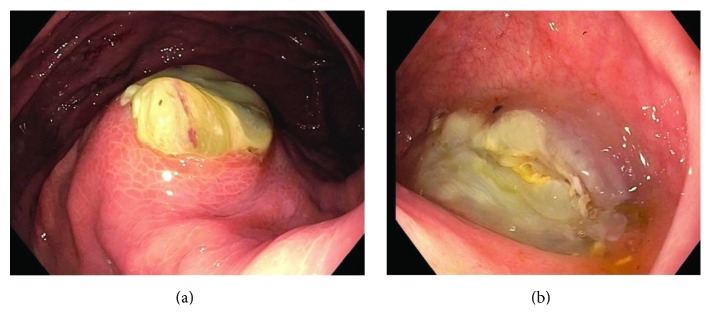
(a) Endoscopic findings of a large lesion with central ulceration along the greater curvature of the proximal body; with mucinous extrusion. (b) Large amounts of mucinous material extruding from the dudodenal wall.

**Figure 15:**
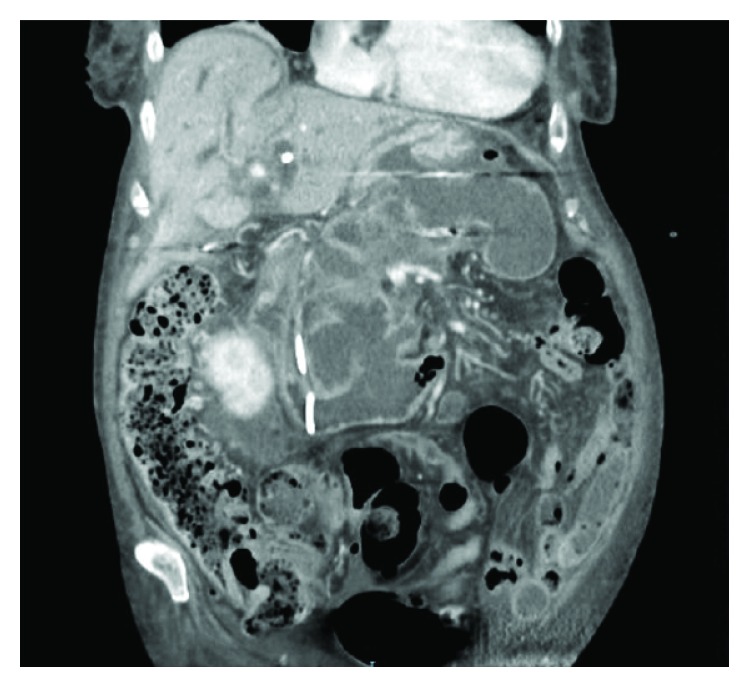
Coronal image of a contrast-enhanced abdominal CT in venous phase showing the rim-enhancing fluid density in the pancreatic bed compressing the stomach against the inferior surface of the liver; and communicating to the stomach via a 1.5 cm defect in the greater curvature.

**Figure 16:**
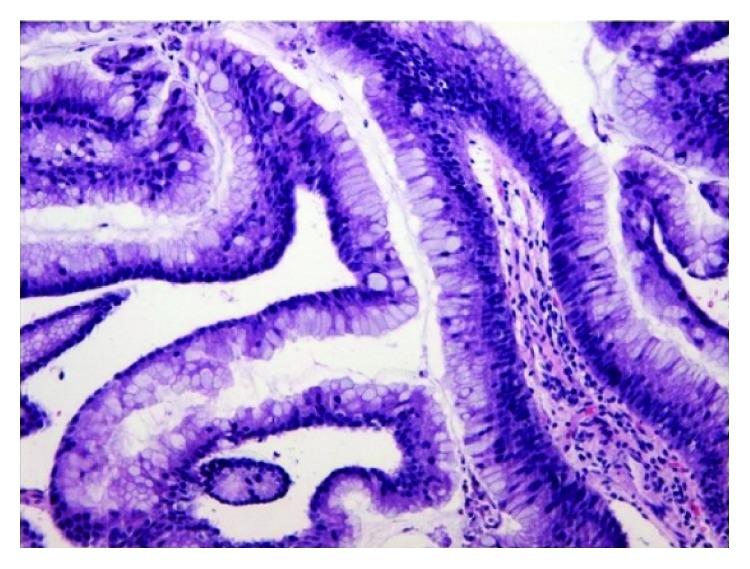
Microphotograph displaying superficial strips of gastric foveolar mucosa with intestinal metaplasia and low-grade dysplasia, surrounded by inflammatory cells (H&E staining ×100).

**Figure 17:**
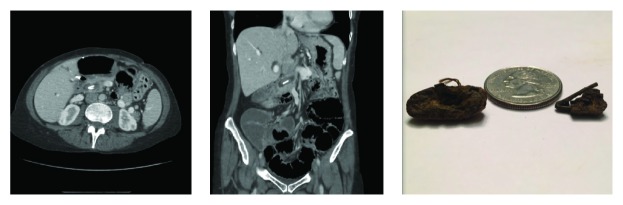


**Figure 18 fig18:**
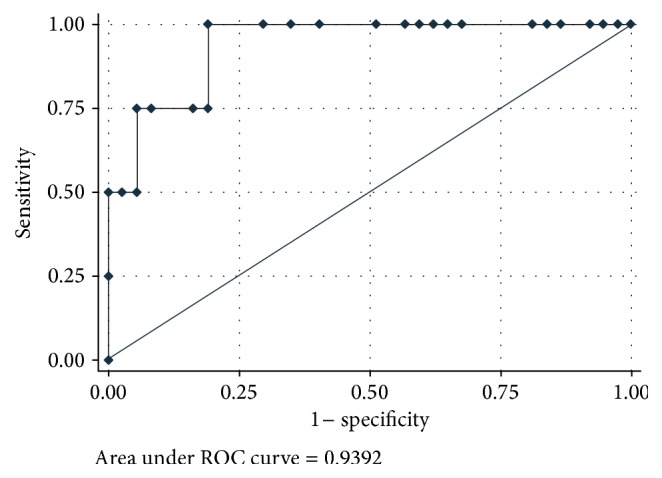
Area under the ROC of liver stiffness measurement using a cutoff value of 5.3 kPa.

**Figure 19:**
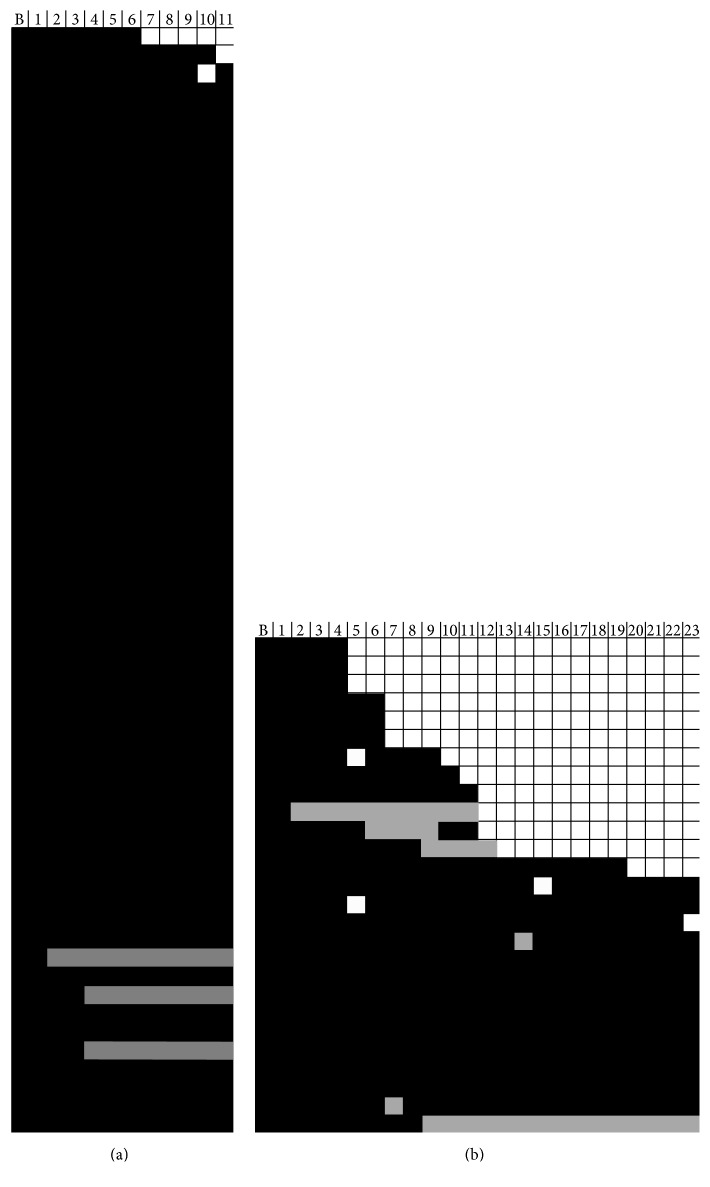
Adherence to PEG-IFN therapy among people who inject drugs in the ACTIVATE study. (a) and (b) represent the shortened arm and standard arm respectively where each row represents a patient. A black box represents a full dose taken, a grey box represents an adjusted dose taken and a white box represents a missed dose at the time point in the column header.

**Figure 20 fig20:**
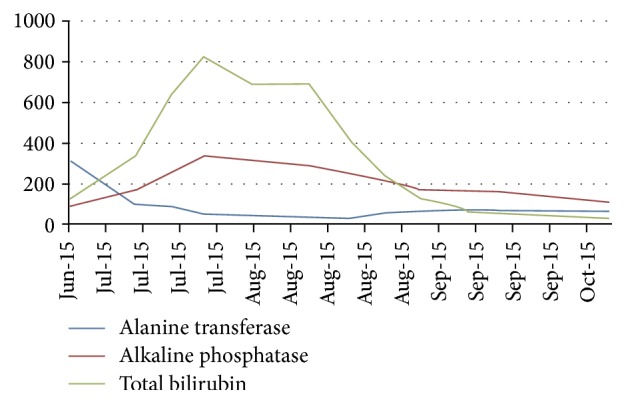
The figure Shows the trend of the liver function test from durig follow up.

**Figure 21 fig21:**
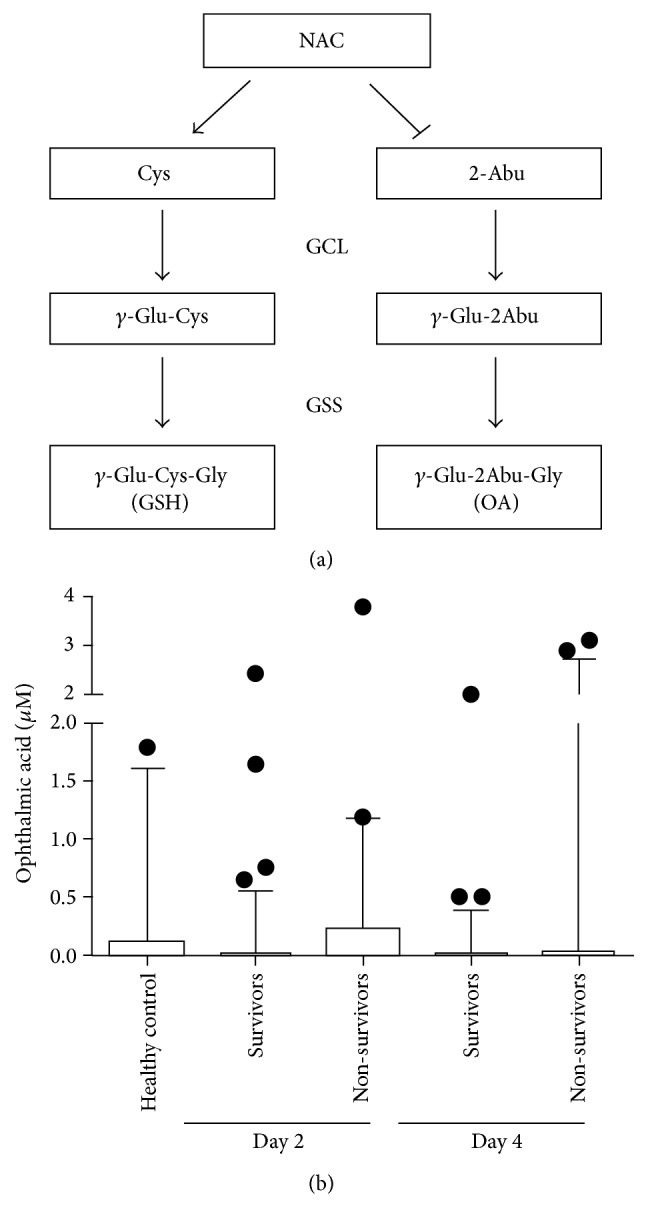
Ophthalmic acid levels in healthy controls compared with surviving and non-surviving APAP-induced ALF patients at Day 2 (early) and Day 4 (late). Blood samples were collected for 130 patients with APAP-induced ALF on Day 2 and Day 4 after admission into hospital; at Day 21, there were 82 survivors and 48 non-survivors. Serum OA levels were quantified using UPLC-MS-MS. The data are shown as a Box and Whiskers plot with boxes representing the interquartile range, lines representing the entire range, and data points representing the outliers.

**Figure 22:**
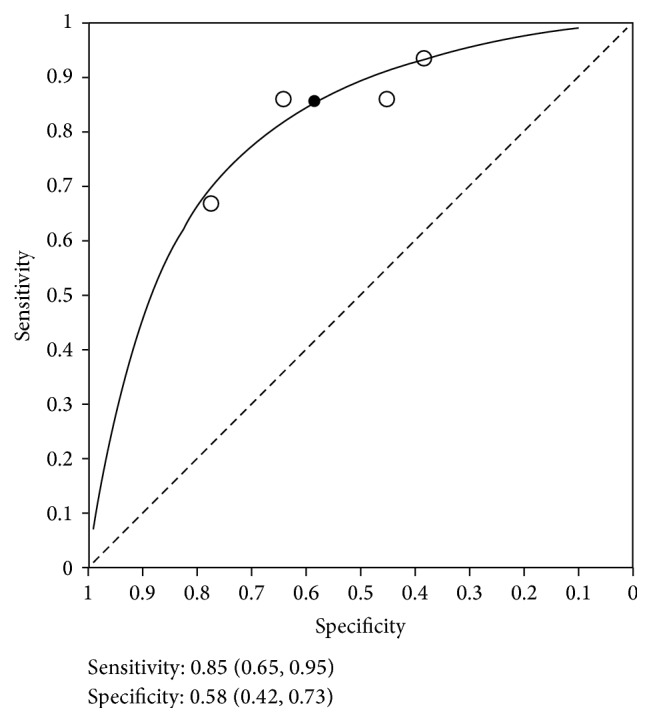
ROC for peak VO_2_ as a predictor of post-liver transplant mortality, revealing high sensitivity and low specificity.

**Figure 23:**
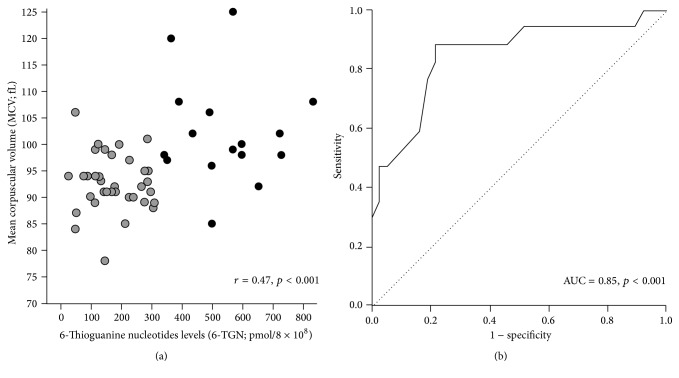


**Figure 24 fig24:**
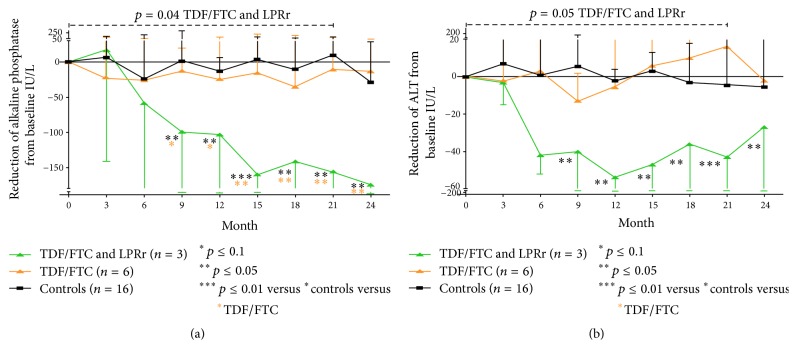


**Figure 25:**
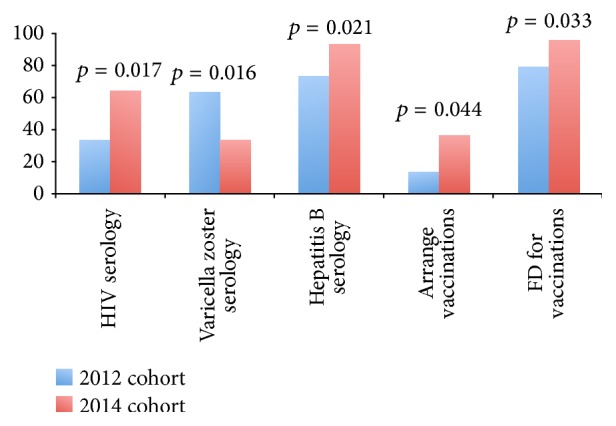
Change in screeing practices for biologic between 2012 and 2014.

**Figure 26 fig26:**
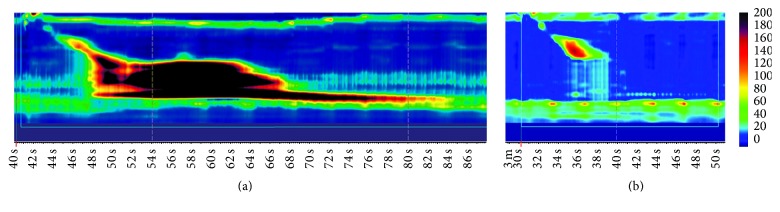
Patient #3 HRM pre and post-POEM (a) hypercontractile contractions with a mean DCI of 46700 mmHg·cm·s and median IRP 33.8 mmHg (b) Post-POEM showing no abnormal contractions and a normal contraction vigor with mean DCI of 2019.6 mmHg·cm·s and median IRP 16.2 mmHg.

**Figure 27:**
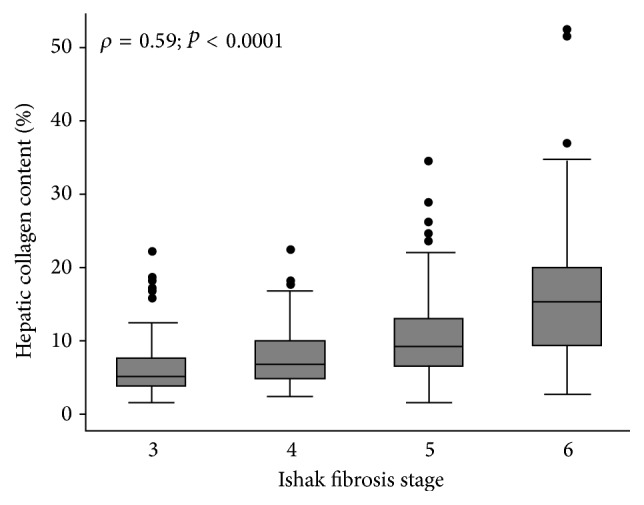


**Figure 28 fig28:**
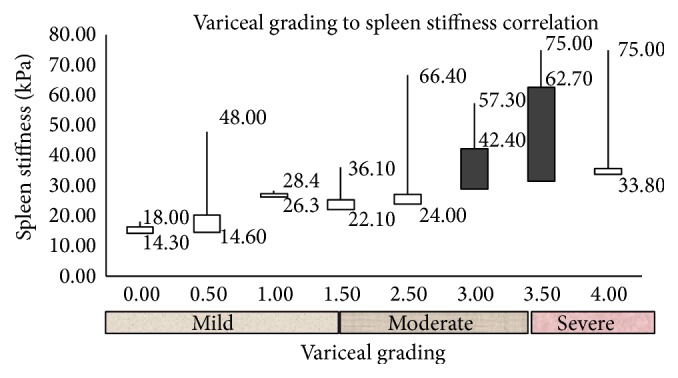


**Figure 29 fig29:**
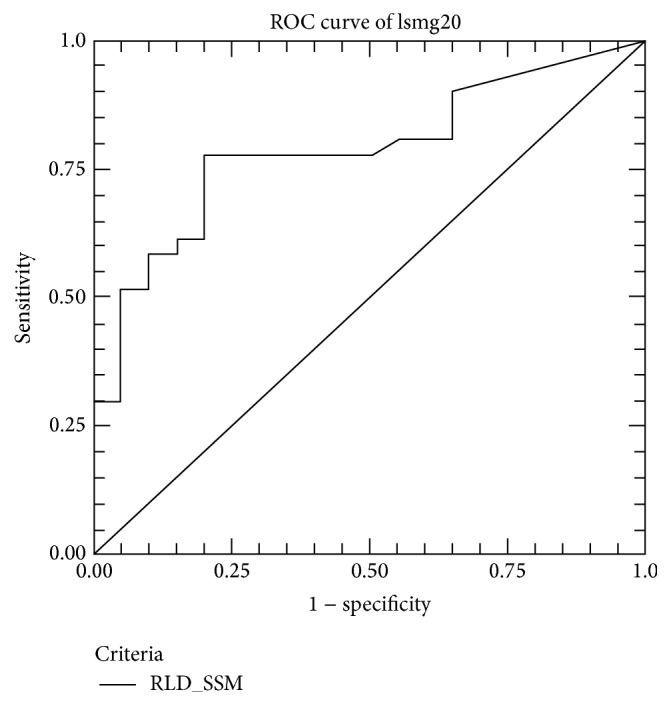


**Figure 30:**
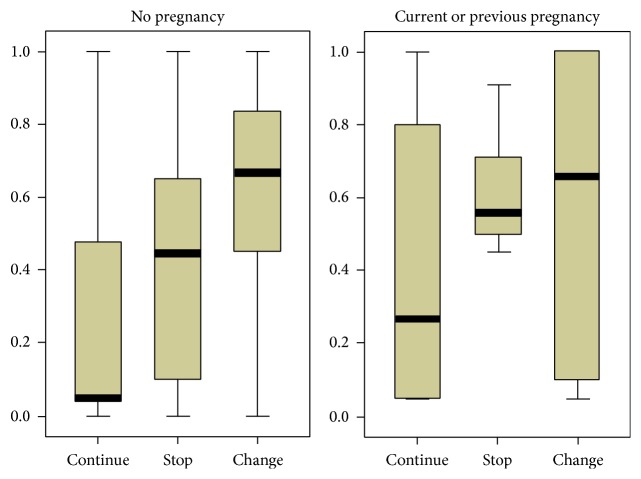
Box-and-whisker plot of TTO model for the two groups. The boxes indicate the 25th and 75th percentiles and the middle line indicating the median value. The whiskers indicate the range of health utilities reported for each hypothetical state.

**Figure 31:**
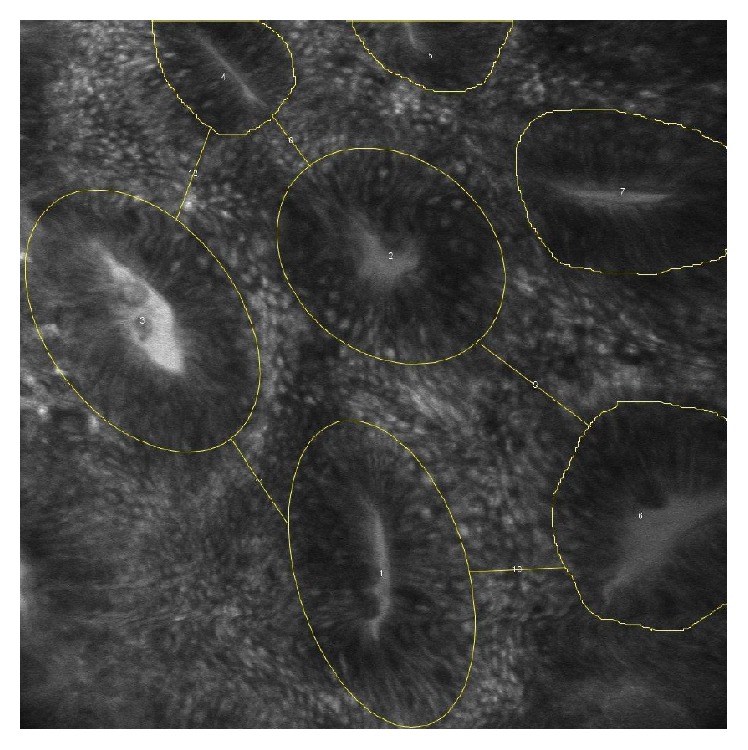


**Figure 32 fig32:**
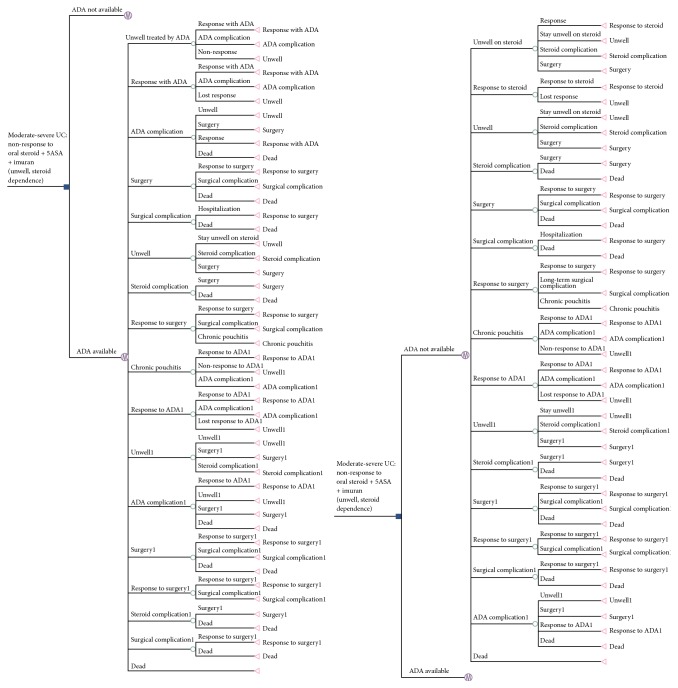
Markov model simulating the progression of a cohort of patients with moderate to severe ulcerative colitis, who are corticosteroid-dependent or refractory to thiopurines, in situations where adalimumab is readily available compared to situations when it is unavailable.

**Figure 33 fig33:**
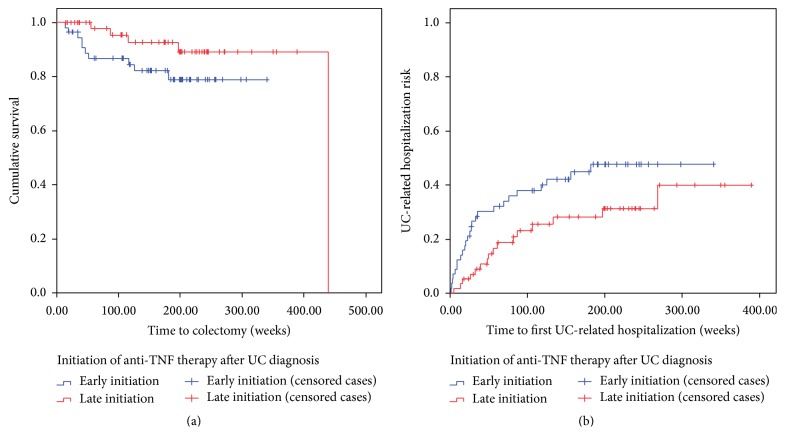
Kaplan-Meier survival curves demonstrating trend towards increased colectomy ((a), *p* = 0.06) and UC-related hospitalization ((b), *p* = 0.02) rate during maintenance infliximab or adalimumab for 57 ulcerative colitis patients starting anti-TNF therapy within three years of diagnosis (blue) compared to 58 patients starting anti-TNF therapy more than three years after diagnosis (red). Hashed lines indicate censored cases (did not meet primary outcome to last follow-up).

**Figure 34:**
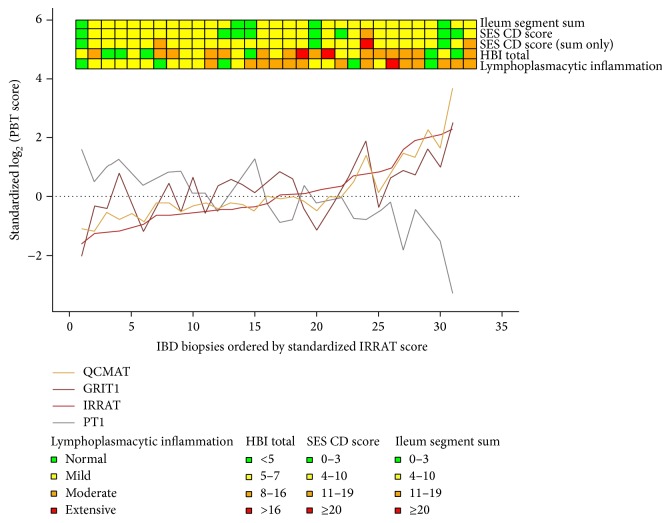


**Figure 35:**
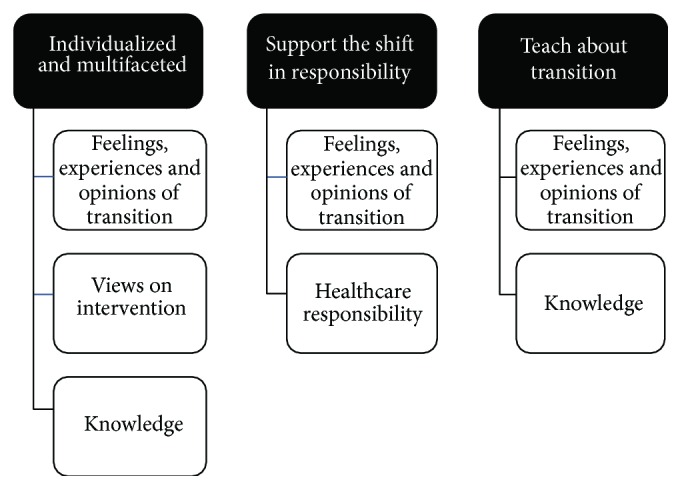
Connections between themes (top) and categories.

**Figure 36:**
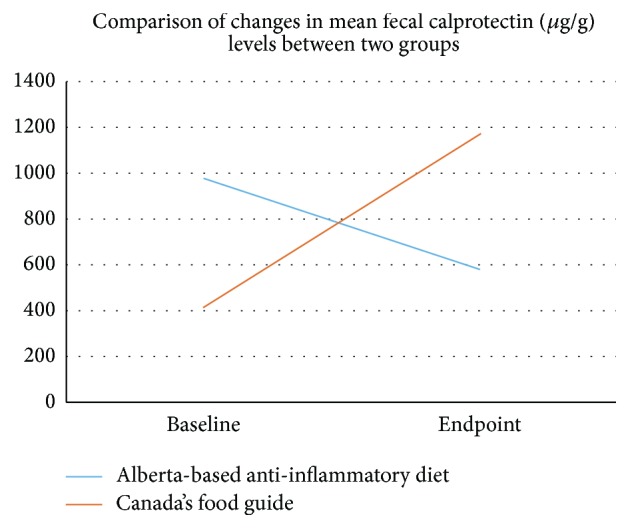


**Figure 37 fig37:**
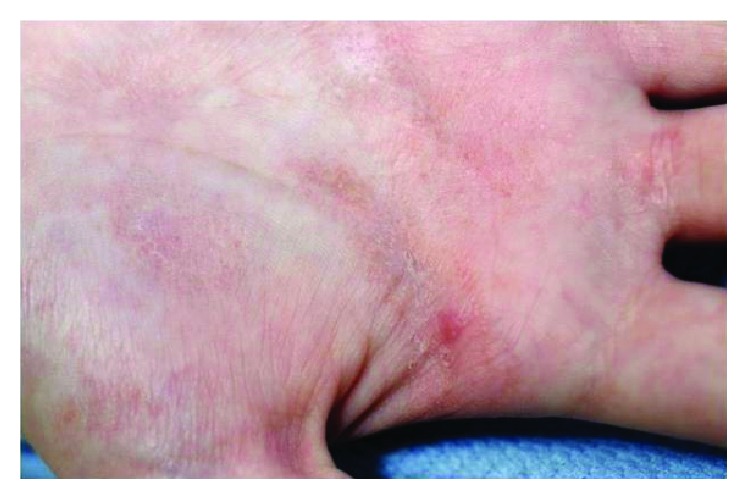
Micro-papules of both palms a presentation.

**Figure 38 fig38:**
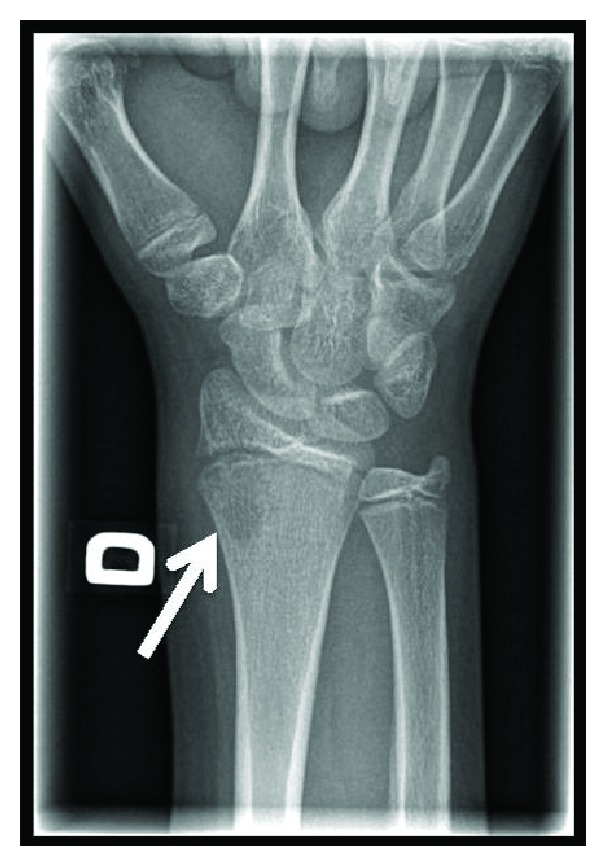
Lytic bony lesion of the distal metaphysis of the right radius compatible with an osteomyelitis.

**Figure 39 fig39:**
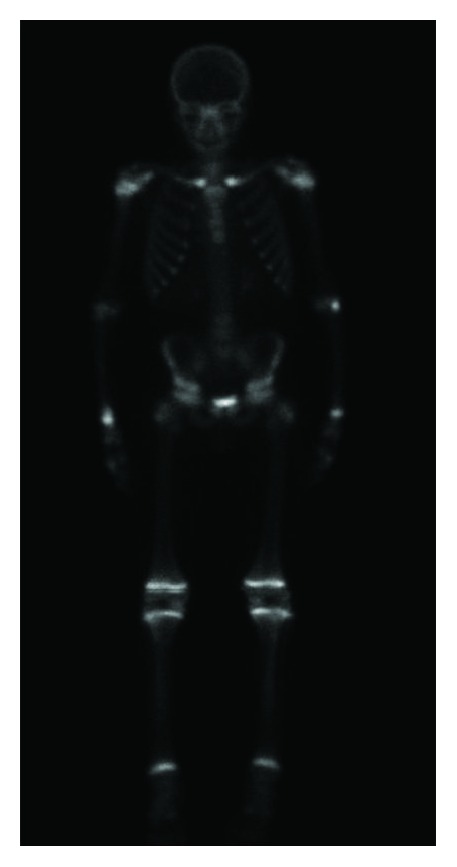
Bone gallium/scintigraphy demonstrated hypercapation at the clavicles bilaterally as well as hypercaptation at the lateral side of the right trochanter.

**Table 1:** Clinical, Endoscopic, and Laboratory Features of 80 IBD Patients Undergoing Colonoscopy at the University of Alberta Inflammatory Bowel Disease Clinic.

	Crohn's Disease	Ulcerative Colitis
*n* (%)	40 (50.0)	40 (50.0)
Male (%)	17 (42.5)	15 (37.5)
Disease Extent (%)	—	—
Ileal	20 (50.0)	—
Ileocolonic	13 (32.5)	—
Colonic	7 (17.5)	—
Proctitis	—	3 (7.5)
Left-sided	—	13 (32.5)
Pancolitis	—	24 (60.0)
Active IBD Treatment (%)	—	—
Steroids	1 (2.5)	1 (2.5)
Biologics	15 (37.5)	8 (20.0)
Immunomodulators	15 (37.5)	14 (35.0)
Endoscopic MH	26 (65.0)	24 (60.0)
Biomarkers (median, IQR)	—	—
FCP (*μ*g/g)	330 (215–599)	322 (199–607)
FIT (ng/mL)	81 (15–1000)	421 (30–1000)
Sensitivity for MH	—	—
FCP < 150 *μ*g/g	1.00	0.94
FIT < 50 *μ*g/mL	0.57	0.81

**Table 2:** 

	Current steroid use	Steroid in prior 6 months	Maintenance Therapy				
			Anti-TNF based therapy^*∗*^	Immunomodulator Monotherapy (AZA or MTX)	5-ASA/ sulfasalazine^*∗*^	Other	None^*∗*^
CD	3%	8%	61%	27%	6%	1%	5%
UC	10%	21%	31%	21%	36%	1%	11%

^*∗*^indicates CD versus UC, *p* < 0.05.

**Table 3 tab3:** High-quality data are needed to validate these conclusions.

Strategy for all patients	Cost	Incremental Cost	Effectiveness	Incremental Effectiveness	Incremental Cost-Effectiveness Ratio	Cost-Effectiveness Ratio	Cost-effectiveness characterization
T + H	9,150	0	0.9705	0	0	9,429	Reference strategy
T	9,296	145	0.8137	−0.1568	−927	11,424	dominated
H + T	9,786	635	0.9148	−0.0557	−11,413	10,697	dominated
H	11,123	1,973	0.5453	−0.4252	−4,640	20,399	dominated

**Table 4:** Multivariate logistic regression analysis of variables associated with NAFLD and significant liver fibrosis by Fibroscan/CAP in 310 HIV mono-infected patients.

Variable	Significant NAFLD (CAP > 260 dB/m)	
	Adjusted Odds Ratio (95% CI)	*p*
Overweight (BMI > 25 kg/m^2^)	4.44 (2.26–8.72)	<0.001
ALT > ULN	2.35 (1.14–4.84)	0.02
Exposure to protease inhibitors antiretrovirals	2.43 (1.19–5.00)	0.02

Variable	Significant liver fibrosis (Liver stiffness > 8 kPa)	
	Adjusted Odds Ratio (95% CI)	*p*

Age	1.11 (1.04–1.18)	0.002
Overweight (BMI > 25 kg/m^2^)	2.91 (1.02–10.29)	0.04
ALT > ULN	8.30 (2.45–28.06)	0.001
Significant NAFLD (CAP > 260 dB/m)	5.82 (1.68–20.11)	0.005

**Table 5 tab5:** 

Risk factors	Hazard Ratio (95% CI)	*P*
Demographics		
Age at LT (per yr)	1.5 (0.8–2.8)	0.27
Sex (M : F)	1.4 (1.1–1.9)	0.02
Smoking at time of LT (Yes : No)	1.9 (0.5–5.0)	0.23

Transplant-related variables		
Allograft failure (Y : N)	1.1 (0.8–1.4)	0.72
Recurrence of PSC (Y : N)	0.8 (0.6–1.1)	0.12
Steroid-resistant rejection	1.3 (0.7–2.2)	0.22
Chronic rejection	1.1 (0.8–1.9)	0.77

Immunosuppression		
Tacrolimus-based immunosuppression (v. Cyclosporine-based)	2.1 (1.5–3.1)	<0.01
Mycophenolate mofetil use after LT (Y : N)	2.4 (1.6–3.5)	<0.01
Azathioprine use after LT (Y : N)	1.1 (0.7–1.6)	0.53
Prolonged prednisone (>6 months) (Y : N)	0.3 (0.1–1.5)	0.13

IBD-related variables		
Pre-LT IBD	0.9 (0.6–1.3)	0.62

**Table 6 tab6:** 

	PBO (*n* = 209)	UST 130 mg (*n* = 209)	UST ~6 mg/kg^c^ (*n* = 209)
Clinical Response^a^			
Wk 3	45 (21.5)	68 (32.5) *p* = 0.010	81 (38.8) *p* < 0.001
^*∗*^Wk 6	60 (28.7)	108 (51.7) Delta = 23% *p* < 0.001	116 (55.5) Delta = 26.8% *p* < 0.001
Wk 8	67 (32.1)	99 (47.4) *p* < 0.001	121 (57.9) *p* < 0.001
Clinical Remission^b^			
Wk 3	24 (11.5)	33 (15.8) *p* = 0.199	48 (23.0) *p* = 0.002
Wk 6	37 (17.7)	60 (28.7) *p* = 0.007	73 (34.9) *p* < 0.001
Wk 8	41 (19.6)	64 (30.6) Delta = 11.0% *p* = 0.009	84 (40.2) Delta = 20.6% *p* < 0.001

*N* (%); ^a^≥ reduction in CDAI; ^b^CDAI < 150; ^c^weight-range based UST doses ~6 mg/kg: 260 mg (weight ≤ 55 kg), 390 mg (weight > 55 kg and ≤ 85 kg), 520 mg (weight > 85 kg); ^*∗*^primary endpoint.

**Table 7:** Neoplasia and CSPYs by FIT cut-off.

	FIT ≥ 50 (*N*/%)	FIT > 75 (*N*/%)	FIT > 100 (*N*/%)	FIT > 200 (*N*/%)
CRC	294 (2.2)	265 (3.0)	250 (3.6)	224 (5.4)
Missed CRC	—	29 (9.9)	44 (15.0)	70 (23.8)
HRP	2731 (20.2)	2159 (24.5)	1838 (26.7)	1305 (31.7)
Missed HRP	—	572 (20.9)	893 (32.7)	1426 (52.2)
Multiple LRP	768 (5.7)	496 (5.6)	386 (5.6)	209 (5.1)
LRP	3270 (24.2)	2028 (23.0)	1551 (22.5)	825 (20.0)
HRF	3793 (28.1)	2920 (33.1)	2474 (35.9)	1738 (42.2)
All Neoplasia	7063 (52.3)	4948 (56.1)	4025 (58.4)	2563 (62.2)
Saved CSPY	—	4684 (34.7)	6605 (48.9)	9375 (69.5)
Total CSPY	13,497	8813	6892	4122

CRC, colorectal cancer; CSPY, colonoscopy; HRF, high-risk finding; HRP, high-risk polyp; LRP, low-risk polyp.

**Table 8 tab8:** Detection Rates Across Time.

	Baseline (*n* = 1,133)	Year 1 (*n* = 1,169)	Year 2 (*n* = 813)	*p* Value			
				Baseline versus Year 1	Baseline versus Year 2	Year 1 versus Year 2	Overall
Adenoma Detection Rate	391 (34.5%)	460 (39.4%)	339 (41.7%)	0.016	0.001	0.295	0.003
Polyp Detection Rate	510 (45.0%)	571 (48.9%)	421 (51.8%)	0.066	0.003	0.198	0.011
High Risk ADR	132 (11.7%)	174 (14.9%)	140 (17.2%)	0.022	<0.001	0.161	0.002

**Table 9 tab9:** Colonoscopy Dictation Report Elements.

	2008 (*n* = 750)	2014 (*n* = 750)	*p*
Age range of charts	19–90	19–90	
Mean patient age	56	58	
Gender composition, %F	53.4	54.6	

Presence of dictation item, %			
Age	61.5	73.2	<0.001
Gender	97.3	98.1	0.298
Preoperative diagnosis	98.7	99.9	0.006
Post-operative diagnosis	99.1	100.0	0.015
Procedure performed	99.7	99.9	1.000
Clinical preamble/indications(s) for procedure	99.5	90.4	<0.001
Consent	40.5	99.1	<0.001
Comorbidities^*∗*^	14.8	32.4	<0.001
Endoscope used	22.5	98.4	<0.001
Quality of bowel preparation	58.8	94.1	<0.001
Sedation (type and dose)	77.6	93.9	<0.001
Medications patient is currently taking^*∗*^	19.2	50.7	<0.001
Digital rectal examination	66.1	86.1	<0.001
Extent of examination	99.6	99.9	0.624
Complications	10.8	88.0	<0.001
Patient comfort^*∗*^	48.9	21.7	<0.001
Withdrawal time	0.8	91.3	<0.001
Rectal retroflexion	34.4	91.3	<0.001
Findings	99.7	100.0	0.500
Pathology specimens taken	90.4	88.1	0.156
Location of sample	87.4	83.9	0.084
Recommendations for subsequent care	92.1	96.3	<0.001

Overall completeness of report, %			
Mean (SD)	64.27 (7.97)	85.32 (7.60)	<0.001
Median (IQR)	63.64 (59.09, 71.43)	86.36 (81.82, 90.91)	—
Range	(38.10, 86.36)	(50.00, 100.00)	—

Report length, pages			
Mean (SD)	1.02 (0.11)	1.18 (0.25)	<0.001
Median (IQR)	1.00 (1.00, 1.00)	1.00 (1.00, 1.25)	—
Range	(0.50, 2.00)	(0.50, 2.00)	—

Correlation: % completeness versus report length	0.19	0.31	

Cecal visualization rate, *n* (%)	716/734 (97.5)	708/727 (97.4)	0.845
Polyp detection rate, *n* (%)	296/750 (39.5)	366/750 (48.8)	<0.001

^*∗*^Items recommended by the CAG/ASGE guidelines that are not included in the SPH template.

**Table 10 tab10:** Ottawa Bowel Preparation Scale (OBPS).

	PEG Traditional	PEG Split-dose	P/MC Traditional	P/MC Split-dose
RCT	5.7 ± 3.4	4.2 ± 3.2	5.1 ± 2.6	4.2 ± 2.5
Real World	6.3 ± 3.3	4.8 ± 3.0	6.0 ± 2.7	5.8 ± 2.9

**Table 11 tab11:** Scale correlation with patient self-reported pain.

	Kappa^*∗*^ (95% CI)	
	VAS	Pain Intensity
SPECS	0.10 (0.03, 0.17)	0.16 (0.09, 0.23)
GS	0.07 (0.01, 0.15)	0.18 (0.12, 0.25)
NAPCOMS	0.12 (0.04, 0.21)	0.13 (0.05, 0.21)
NPAT	0.06 (−0.01, 0.13)	0.06 (0.00, 0.13)

^*∗*^Kappa measures the agreement between two categorical items.

**Table 12 tab12:** Presence of EGD procedure report variables.

	2008 (*n* = 685)	2014 (*n* = 677)
Presence of dictation item, %		
Age	65.5	63.7
Gender	98.4	98.1
Preoperative diagnosis	99.6	98.2
Post-operative diagnosis	99.3	96.2
Procedure performed	99.7	99.6
Clinical preamble/indications(s) for procedure	87.0	83.3
Consent	45.7	80.5
Comorbidities	16.9	25.8
Endoscope used	32.8	40.0
Sedation (type and dosage)	74.9	92.8
Medications	34.2	40.9
Complications (if any)	10.9	40.0
Extent of examination	99.9	99.4
Patient comfort	43.5	32.9
Findings	100.0	100.0
Pathology specimens taken	96.9	100.0
Location of sample	98.5	99.8
Recommendations for subsequent care	87.7	95.3
Overall completeness of report, %		
Mean (SD)	71.53 (10.88)	76.82 (9.11)
Median (IQR)	72.22 (61.11, 77.78)	77.78 (72.22, 83.33)
Range	(44.44, 100.00)	(47.06, 100.00)

**Table 13 tab13:** Inter-observer Reliability: GS and SPECS.

		Kappa (95% Cl)

GS	Total	Fair 0.35 (0.28, 0.41)

SPECS^*∗*^	Total	Moderate 0.43 (0.36, 0.50)
	Vocalization	Moderate 0.41 (0.34, 0.47)
	Positioning/Body Language	Fair 0.34 (0.28, 0.41)
	Patient Anxiety/Emotion	Moderate 0.42 (0.35, 0.49)

^*∗*^SPECS was categorized into 4 levels: 0, 1–3, 4–6 and 7–9.

**Table 14 tab14:** 

Mean Age	Gender M/F	Fe Infusion %	Oral Fe %	Capsule Endoscopy % done	Blood Transfusion % Received	PPI Treatment %	Surgical Repair %
67	7/14	33	43	95	33	90	33

**Table 15 tab15:** 

Gender (%)	Male 38 (84%) Female 7 (16%)

Cause of Liver Disease	
HBV	18
HCV	19
ETOH	5
Hemochrmatosis	1

Age at Diagnosis	62.8 years

Average Survival after HCC Diagnosis	28 months

Average Survival after PVT Diagnosis	15 months

Total Patient 45 Anticoagulation No Yes Initial PVT Involvement Right PVT 18 (40%) Left PVT 13 (28%) Main PVT 6 (13%) Multi Involvement 8 (17%)	PVT Progression 23 19 (49%) 4 (67%) 12 (67%) 7 (54%) 4 (67%)

HCC type	
Single Lesion	30 (67%)
Multifocal	15 (33%)

HCC Treatment Modality	
TACE	19 (42%)
RFA	3 (7%)
TACE + RFA	8 (18%)
Systemic Treatment	35 (78%)

MELD Score (average)	8.25

Child	A (71%), B (29%)

**Table 16:** 

	Value	Normal range
ALT (IU/L)	56	5–40
AST (IU/L)	71	15–55
Ferritin (ug/L)	605	15–300
Iron saturation	0.36	0.15–0.5
Alpha fetoprotein (ug/L)	6.6	0–6
6-MMP (pmol/8 × 10^8^ RBCs)	19431	<5700

**Table 17 tab17:** 

	Control (*N* = 18)	Bacon (*N* = 28)	*p*-value
Age (mean ± SD)	57.6 ± 15.7	60.4 ± 16.2	0.56
Female (%)	11 (61.1%)	17 (60.7%)	0.98
GTT (min)			
Mean ± SD	21.9 ± 16.4	69.2 ± 172.9	0.13
Median (IQR)	16.1 (11.7–23.1)	13.9 (9.2–58.8)	
SBTT (min)			
Mean ± SD	210.5 ± 104.3	233 ± 183.6	0.32
Median (IQR)	187.4 (153.1–269.1)	181.4 (120.6–279.4)	
CER (%)	18 (100%)	24 (85.7%)	0.09

**Table 18 tab18:** Procedural characteristics for patients with small bowel Crohn's disease undergoing balloon-assisted enteroscopy.

Characteristic	Total (*n* = 68)

Route	
Anal	85.3% (58)
Oral	14.7% (10)
Procedures Involving Dilations	42.6% (29)
Number of Dilations	66
Mean Dilation Diameter (mm)	16.4
Failed or Non-Traversable Dilations	20.5% (14)
Other Intervention (clip or polyp removal)	5.9% (4)
Physician Recommendation	
Surgery	16.2% (11)
Follow-Up BAE	36.8% (25)
Other Imaging (CTE, VCE, MRE)	20.6% (14)
Medication Escalation	35.3% (24)
Medication Maintenance	64.7% (44)
Complications	2.9% (2)

**Table 19:** Patient baseline characteristics.

	Colonoscopy	Capsule
	(*N* = 21)	(*N* = 22)
Age	67.8	66.3
Gender (M/F)	8 : 13	5 : 17
Concurrent IBD	3/21	4/22
Use of a biologic	2/21	3/22
Chronic PPI use	7/21	4/22
Antibiotic use prior to first CDI	15/21	21/22
#CDI episodes (median)	4.0	3.5
Charlson comorbidity index (median)	3.0	4.0
Hb	136	136

**Table 20:** Patient Characteristics and Clinical Data.

Characteristic	Success *n* = 106	Failed *n* = 30	*p*-value

Age	67.0 (17.4–97.7)^1^	74.3 (23.1–88.4)	0.354
Women	64 (60.4%)	14 (46.7%)	0.180
Diabetes	18 (17%)	8 (26.7%)	0.234
Previous MI	24 (22.6%)	7 (23.3%)	0.936
Recurrent UTI	26 (24.5%)	7 (23.3%)	0.893
Immunosuppressed	21 (19.8%)	11 (36.7%)	0.055
Chronic PPI^1^	50 (47.2%)	19 (63.3%)	0.118
Post-FMT Abx^3^	16 (15.1%)	7 (23.3%)	0.288
Chronic Statin^2^	35 (33%)	14 (46.7%)	0.169
IBD	13 (12.3%)	2 (6.7%)	0.388
Charlson Index	4 (0–11)^1^	5 (0–11)	0.013
COPD	13 (12.3%)	10 (33.3%)	0.007
Maximum WBC^4^	13.4 (4.7–45.4)^1^	21.1 (6.7–58.3)	<0.001
Hospital-acquired	36 (34%)	21 (70%)	<0.001
Inpatient Status	19 (17.9%)	21 (70%)	<0.001
Refractory to Abx^5^	0	9 (30%)	<0.001
Severe/complicated	6 (5.7%)	11 (36.7%)	<0.001

^1^Median (range); ^2^Use for >12 weeks before FMT; ^3^For reasons other than CDI; ^4^Maximum count during any recurrence; ^5^Complete non-responsiveness to antibiotics.

**Table 21:** 

	UC	PC	FC	TOTAL
Group A	59	7	27	93
Group B	8	1	12	21
Group C	1	0	20	21
Group D	0	0	15	15
TOTAL	68	8	74	150

**Table 22:** 

22 G needle	Total cytology score	Cytology score per specimen	No of core specimens achieved	Total core score	Core score per specimen
Boston	92	2.3	37.5%	24	1.6
Olympus	88	2.2	47.5%	28	1.47

**Table 23:** Branched Chain Amino Acids Prior to and During Admission.

	2 Months Prior to Admission	Day of Admission	Second Day of Admission
Valine (100–310 *μ*M/L)	504	1430	831
Isoleucine (20–140 *μ*M/L)	244	835	442
Leucine (50–180 *μ*M/L)	255	1930	1070
Allo-isoleucine (*μ*M/L)^†^		161	169

^†^Most recent level 8 *μ*M/L taken 8 months prior to admission.

**Table 24:** 

Response %	TDF	FTC/TDF
(*n*/*N*)	(*N* = 141)	(*N* = 139)
HBV DNA <69 IU/mL	83 (117/141)	83 (115/139)
HBV DNA <29 IU/mL	82 (115/141)	82 (114/139)
Normalized ALT^a^	65 (51/79)	71 (59/83)
HBeAg loss^b^	25 (16/65)	19 (13/68)
HBeAg seroconversion^b^	12 (8/65)	10 (7/68)
HBsAg loss	1.4 (2/141)	3.6 (5/139)
HBsAb seroconversion	0	1.4 (2/139)

^a^Included only patients with ALT > ULN at BL; ^b^HBeAg + patients.

**Table 25:** Demographic characteristic of eligible CCC participants based on DAAs initiation.

	DAA initiators	DAA Initiators (by clinical trials)	Did not receive DAAs
	(*n* = 43)	(*n* = 23)	(*n* = 706)
Women *n* (%)	7 (16)	4 (17)	212 (30)
Aboriginal *n* (%)	2 (5)	4 (17)	174 (25)
Income <$1500/month *n* (%)	30 (70)	12 (52)	537 (76)
Injection drug use (last 6 months) *n* (%)	2 (5)	5 (22)	249 (35)
Alcohol Use *n* (%)	3 (8)	1 (4)	132 (22)
CD4 T Count (cells/mL) median, IQR	460 (290, 754)	460 (360, 620)	470 (290, 670)
APRI >1.5 *n* (%)	12 (28)	2 (10)	139 (20)
HIV Combined Antiretroviral therapy	40 (93)	21 (91)	594 (84)

**Table 26:** Meta-analysis of Liver Stiffness Progression Rates (LSPR) in Chronic HCV infection.

	*N*	Fixed Effect		Random Effects		*I* ^2^
		Meta-Analysis		Meta-Analysis		
		LSPR (kPa/yr)	95% CI	LSPR (kPa/yr)	95% CI	
All studies						
Indirect	31	0.091	0.085–0.098	0.141	0.115–0.166	92%
Direct	8	0.099	0.067–0.131	0.137	0.069–0.205	47%
HCV monoinfected						
Indirect	13	0.082	0.072–0.092	0.100	0.073–0.128	83%
Direct	3	0.083	0.049–0.116	0.085	0.040–0.129	27%
HIV/HCV coinfected						
Indirect	16	0.097	0.088–0.105	0.167	0.127–0.208	94%
Direct	5	0.261	0.155–0.367	0.261	0.155–0.367	0%

**Table 27 tab27:** 

Area of expertise	

Medical oncology	11 (15.9%)
Hematology	55 (79.7%)
Other	3 (4.4%)

Province	

BC	13 (18.8%)
AB	6 (8.7%)
MB	1 (1.5%)
ON	36 (52.2%)
QC	6 (8.7%)
NB	4 (5.8%)
PE	1 (1.5%)
NL	2 (2.9%)

**Table 28:** 

	Group 1 (Minor Injury)	Group 2 (Moderate injury)	Group 3 (Severe injury)
Patient, *n* (%)	50 (40.7%)	55 (44.7%)	18 (14.6%)
Mean Peak AST	573	1,860	6,917
Deaths, *n* (%)	7 (14%)	13 (23.6%)	5 (27.8%)
retransplant, *n* (%)	3 (6.0%)	3 (5.5%)	2 (11.1%)
Death and retransplant	10 (20.0%)	16 (29.1%)	7 (38.9%)
Ratio M/F	1.33	2.67	1.25
Status 1, *n* (%)	28 (56.0%)	31 (56.4%)	9 (50.0%)
Status 1T, *n* (%)	13 (26.0%)	14 (25.5%)	5 (27.8%)
Status 2, *n* (%)	7 (14.0%)	3 (5.5%)	1 (5.6%)
Status 3, *n* (%)	1 (2.0%)	4 (7.3%)	2 (11.1%)
Status 4, *n* (%)	1 (2.0%)	3 (5.5%)	1 (5.6%)

**Table 29 tab29:** Patient Knowledge and Comfort with GP Management of NAFLD Before and After Education Workshop.

	Score (out of 5)	Pre-Workshop (*n*)	Post-Workshop (*n*)
Extent of Knowledge	1	39	0
	2	25	0
	3	35	5
	4	39	48
	5	4	77

Patient Confidence in Referring Physician's Ability to Manage Disease	1	1	1
	2	12	3
	3	24	5
	4	46	43
	5	34	73

**Table 30:** 

	PEAK (%)	TTP (sec)	RBV (cm^3^)	RBF (cm^3^/sec)	Stiffness (kPa)
LP Controls	56.6 ± 6.1	37.7 ± 10.8	7213.6 ± 2667.8	75.6 ± 10.2	4.1 ± 0.9
LP CHC	48.3 ± 10^*∗*^	43 ± 12.6	5679 ± 1795^*∗*^	64 ± 14.2^*∗*^	8.4 ± 2.9^*∗*^
LP steatosis grade 1	48.5 ± 8.9^*∗*^	39.2 ± 9.7	6446.9 ± 2671	64.4 ± 13.5^*∗*^	5.5 ± 2.1^*∗*,*∗∗*^
LP steatosis grade 2	40.8 ± 5.2^*∗*,*∗∗*,°^	48 ± 14.8^*∗*,°^	4680 ± 1861.7^*∗*,°^	52.4 ± 7.4^*∗*,*∗∗*,°^	6.5 ± 2.1^*∗*,*∗∗*^
LP steatosis grade 3	36.3 ± 4.8^*∗*,*∗∗*,°^	43.5 ± 15.1	3268.1 ± 582.9^*∗*,*∗∗*,°^	46.5 ± 9.8^*∗*,*∗∗*,°^	9 ± 7.9^*∗*^

**Table 31:** Characteristics of PSC Patients According to Baseline sIgG4 Concentration.

	Normal sIgG4	Elevated sIgG4	*p*-value
	(≤140 mg/dL)	(>140 mg/dL)	
	(*n* = 200)	(*n* = 34)	
Age, years	44 (37–51)	49 (38–59)	**0.046**
Male	63% (126)	68% (23)	0.70
White race	84% (168)	97% (33)	0.058
Ulcerative colitis	46% (91)	56% (19)	0.27
UDCA use	60% (119)	59% (20)	>0.99
ALT, U/L	64 (36–112)	58 (35–123)	0.60
Alkaline phosphatase, U/L	260 (127–399)	262 (158–430)	0.31
GGT, U/L	236 (93–495)	252 (114–662)	0.50
Bilirubin, *μ*mol/L	0.7 (0.5–1.1)	0.7 (0.5–1.0)	0.92
INR	1.0 (0.9–1.0)	1.0 (0.9–1.0)	0.88
Albumin, g/dL	4.1 (3.8–4.3)	3.8 (3.5–4.2)	**0.010**
Platelets, ×10^3^/*μ*L	248 (196–306)	282 (237–347)	**0.030**
FibroTest	0.41 (0.23–0.58)	0.48 (0.24–0.64)	0.20
ELF	9.46 (8.54–10.33)	9.46 (8.84–10.84)	0.25
Serum LOXL2, pg/mL	100 (70–146)	113 (88–155)	0.18
Ishak 3–6 fibrosis	50% (100)	56% (19)	0.58
MELD	7 (6–8)	6.5 (6–8)	0.81
Mayo risk score	–0.135 (–0.615, 0.365)	0.015 (–0.47, 1.11)	0.15

All data are median (IQR) or % (*n*).

**Table 32 tab32:** Metavir Score by Liver Condition.

Metavir Score	Total	Liver Condition					
		HBV	HCV	Fatty Liver	Alcohol	Autoimmune	Other Liver Condition
Sample Size	5437	3399	865	836	107	100	172

F0							
Count	1224	920	106	146	11	13	35
Column%	22.5%	27.1%	12.3%	17.5%	10.3%	13.0%	20.3%

F0-F1							
Count	1643	1222	166	174	20	28	42
Column%	30.2%	36.0%	19.2%	20.8%	18.7%	28.0%	24.4%

F1							
Count	821	505	128	153	10	14	17
Column%	15.1%	14.9%	14.8%	18.3%	9.3%	14.0%	9.9%

F1-F2							
Count	297	155	50	66	10	9	10
Column%	5.5%	4.6%	5.8%	7.9%	9.3%	9.0%	5.8%

F2							
Count	541	262	120	115	12	14	21
Column%	10.0%	7.7%	13.9%	13.8%	11.2%	14.0%	12.2%

F2-F3							
Count	171	72	41	39	4	6	10
Column%	3.1%	2.1%	4.7%	4.7%	3.7%	6.0%	5.8%

F3							
Count	178	76	52	40	3	5	5
Column%	3.3%	2.2%	6.0%	4.8%	2.8%	5.0%	2.9%

F3-F4							
Count	142	73	31	25	5	2	9
Column%	2.6%	2.1%	3.6%	3.0%	4.7%	2.0%	5.2%

F4							
Count	61	24	27	10	2	0	1
Column%	1.1%	0.7%	3.1%	1.2%	1.9%	0.0%	0.6%

Established cirrhosis							
Count	359	90	144	68	30	9	22
Column%	6.6%	2.6%	16.6%	8.1%	28.0%	9.0%	12.8%

**Table 33 tab33:** 

Metavir score	HBV
Sample size	3399

F0	
Count	920
Column%	27.10%
F0-F1	
Count	1222
Column%	36.00%

F1	
Count	505
Column%	14.90%

F1-F2	
Count	155
Column%	4.60%

F2	
Count	262
Column%	7.70%

F2-F3	
Count	72
Column%	2.10%

F3	
Count	76
Column%	2.20%

F3-F4	
Count	73
Column%	2.10%

F4	
Count	24
Column%	0.70%

Established cirrhosis	
Count	90
Column%	2.60%

**Table 34:** Mean Individual Health Care Costs in the Last Year of Life^*∗*^.

Sector	All persons *N* = 264,754	Non-IBD *N* = 262,540	IBD *N* = 2,214	CD *N* = 975	UC *N* = 1,134
Hospitalization	23,010	22,928	32,629	35,983	30,471
Emergency Department	1,273	1,270	1,623	1,812	1,452
Long-term Care	8,322	8,344	5,720	4,381	6,480
Complex Continuing Care	3,439	3,436	3,702	3,939	3,727
Home Care	4,430	4,421	5,356	5,413	5,348
Rehabilitation	890	884	1,579	1,643	1,617
Outpatient clinics	3,479	3,477	3,813	4,334	3,517
Physician Billings	5,340	5,326	7,037	7,594	6,678
Non-physician Billings	322	323	251	202	281
Laboratory	215	214	266	270	259
Drugs/Devices	2,943	2,931	4,287	4,839	3,825
Total	53,661	53,555	66,263	70,408	63,655

^*∗*^2013 Canadian dollars.

**Table 35 tab35:** 

	Liver Disease No Cirrhosis	Liver Disease With Cirrhosis	Average Risk
Number of Patients	32	20	28
Mean Age	59	63	65
FIT Positive	4	12	1
FIT Negative	28	8	27
% FIT Positive	12.5%	60.0%	3.7%
ADR	3/4 = 75%	10/12 = 83.3%	1/1 = 100%

FIT Positive Mean INR	1.01	1.18	0.98
FIT Negative Mean INR	1.00	1.05	1.04
FIT Positive Abnormal INR	0	0	0
FIT Negative Abnormal INR	0	0	0

FIT Positive Mean platelets	200	127	172
FIT Negative Mean platelets	218	144	245
FIT Positive Abnormal platelets	1/4	10/12	0
FIT Negative Abnormal platelets	2/28	5/8	0

**Table 36:** Change in prevalence of IBD between 2015 and 2025.

Analysis	Annual Percentage Change (95% CI)	2015			2025		
		Prevalence per 100,000	# of people with IBD	Total cost	Prevalence per 100,000	# of people with IBD	Total cost
Canada							
CD	3.75^*∗*^ (2.57, 4.93)	400	143,645	$761,387,855	578	227,795	$1,494,819,111
UC	1.74 (−0.02, 3.54)	260	93,163	$493,809,045	309	121,566	$797,730,230
IBD	2.79^*∗*^ (1.68, 3.91)	660	236,807	$1,255,196,900	887	349,361	$2,292,549,341

USA							
CD	3.27^*∗*^ (0.17, 6.53)	259	831,994	$8,772,936,478	357	1,240,590	$17,053,678,379
UC	1.66 (−1.12, 4.51)	275	883,669	$5,711,328,062	324	1,125,494	$9,483,218,424
IBD	2.25^*∗*^ (0.07, 4.48)	534	1,715,663	$14,484,264,540	681	2,366,083	$26,536,896,803

North America							
CD	2.67^*∗*^ (1.33, 4.03)	273	975,639	$9,534,324,334	380	1,468,384	$18,548,497,490
UC	1.86^*∗*^ (0.40, 3.34)	273	976,832	$6,205,137,106	322	1,247,059	$10,280,948,654
IBD	2.39^*∗*^ (1.39, 3.41)	547	1,952,470	$15,739,461,440	702	2,715,444	$28,829,446,144

^*∗*^Denotes significant increase.

**Table 37:** Treatment success rates of different regimens.

Treatment	1st line	2nd line	3rd line	4th line	Total
Sequential	66/80 (82.5%)	2/6 (33.3%)	2/2 (100%)	No data	70/88 (79.5%)
PPI-CA	83/207 (40.1%)	8/33 (24.2%)	1/4 (25.0%)	0/1 (0.0%)	92/245 (37.7%)
PPI-CM	5/25 (25.0%)	2/17 (11.8%)	0/3 (0.0%)	No data	7/45 (15.6%)
Bismuth Quadruple	8/13 (61.5%)	36/67 (53.7%)	20/31 (64.5%)	3/6 (50.0%)	67/117 (57.3%)
PPI-AL	1/2 (50.0%)	2/12 (16.7%)	10/23 (43.5%)	4/5 (80.0%)	17/42 (40.5%)
Miscellaneous	5/9 (55.6%)	6/16 (37.5%)	6/11 (54.5%)	2/2 (100%)	19/38 (50.0%)
Total	168/336 (50.0%)	56/151 (37.9%)	39/74 (52.7%)	9/14 (64.3%)	

**Table 38:** Examples of selected scenarios presented in our survey.

*Scenario 1* (Healthy, stable). ∖“A 50-year-old healthy woman presents with MELENA and is hemodynamically STABLE (BP 120/80, HR 65). There is NO evidence of a volume deficit on clinical exam. BELOW what hemoglobin level (in g/L) would you transfuse red blood cells in this patient?∖”	*Scenario 2* (Cardiac disease, stable). ∖“A 50-year-old man with triple-vessel coronary artery disease presents with MELENA and is hemodynamically STABLE (BP 120/80, HR 65). There is no evidence of a volume deficit on clinical exam. The patient denies having any chest pain or dyspnea, and his ECG and troponin are unremarkable. BELOW what hemoglobin level would you transfuse red blood cells in this patient?∖”

*Scenario 3* (Cirrhosis, stable). ∖“A 65-year-old patient with decompensated cirrhosis presents with HEMATEMESIS and is hemodynamically STABLE (BP 100/60, HR 85). There is no evidence of a volume deficit on clinical exam. BELOW what hemoglobin level would you transfuse red blood cells in this patient?∖”	*Scenario 4* (Warfarin therapy, unstable). ∖“A 65-year-old woman with hypertension and atrial fibrillation who is taking Warfarin (INR 2.5) presents with MELENA, and is hemodynamically UNSTABLE (BP 90/60, HR 115) There is evidence of a volume deficit on clinical exam and the patient is being resuscitated with intravenous crystalloid. BELOW what hemoglobin level would you transfuse red blood cells in this patient?∖”

**Table 39:** Patients with Jackhammer esophagus treated with POEM.

Patient	Myotomy (cm)	LES included	Median IRP (mmHg)		Mean DCI (mmHg·cm·s)		Eckardt score		IEM^*∗*^
			Before	After	Before	After	Before	After	
#1	20	−	19.5	23.5	12516.5	84.2	2	6	+
#2	21	+	16.4	10.5	18332.4	137.7	5	0	+
#3	12	+	33.8	16.2	46700	2019.6	5	0	−
#4	23	+	7.3	12.4	15388.7	234	11	2	+

^*∗*^IEM-ineffective esophageal motility after POEM = ≥50% ineffective swallows (failed or weak contraction vigor (DCI < 450 mmHg·cm·s)).

**Table 40 tab40:** 

Indication	*n*	Albumin dose (gm)	Duration (d)	Desired outcome
LVP	50	55.0 ± 13.4	1	46/50 (92%)
SBP	6	162.5 ± 89.1	2.8 ± 1.5	6/6 (100%)
HRS	9	436.1 ± 310.5	8.9 ± 6.0	3/9 (33%)
Non-HRS AKI	15	215.0 ± 184.6	3.9 ± 2.1	11/15 (73%)
Ascites mobilization	5	205.0 ± 144.0	4.0 ± 3.0	0/5 (0%)
Hyponatremia	5	205.0 ± 118.0	4.4 ± 2.3	4/5 (80%)
Hypotension	2	100, 25	2, 1	0/2 (0%)
Hypoalbuminemia	3	50, 150, 75	1, 3, 1	1/3 (33%)
Edema	2	50, 50	2, 1	1/2 (50%)
Hypovolemia	3	25, 50, 200	1, 1, 4	1/3 (33%)

**Table 41 tab41:** Characteristics of patients with hyponatremia.

	mild	Moderate	Severe
# of episodes	71	101	20
Serum [Na]	133 ± 1^*∗*^	128 ± 3	119 ± 1
Serum creatinine	105 ± 75	116 ± 67	145 ± 79^#^
C-P score	9.4 ± 1.8	10.0 ± 1.7	10.0 ± 1.9
MELD score	17 ± 7^*∗*^	20 ± 8	21 ± 11
Episodes on diuretics	52/71	75/101	14/20
Furosemide dose	39 ± 23 mg	51 ± 30 mg	55 + 23 mg
Spironolactone dose	89 ± 54 mg	112 ± 70 mg	110 ± 38 mg
Episodes on beta-blockers	34/71	30/101	1/20^#^
Survival at 1 year	58%	60%	49%

^*∗*^ *p* < 0.05 compared to moderate/severe groups, ^#^ *p* < 0.05 compared to mild/moderate groups.

**Table 42 tab42:** Patient break down based on disease etiology.

Disease	Number of patients in group
Chronic Hepatitis B (B)	12
Chronic Hepatitis C (C)	15
Non-Alcoholic Fatty Liver disease (F)	14
Autoimmune Hepatitis	11

**Table 43 tab43:** 

“Ideal 10-year survivor of pediatric LT”		
(1) No Retransplantation	(7) No PTLD	(11) No ongoing use of prednisone
(2) No Chronic Rejection	(8) No renal dysfunction	(12) No antihypertensive agent
(3) Normal ALT	(9) Linear growth ≥ −2SD	(13) No antiseizure medication
(4) Normal Total Bilirubin	(10) No diabetes	
(5) Normal Albumin		
(6) Normal GGT		

**Table 44 tab44:** Baseline demographic and clinical characteristics.

Characteristic	*n* (%), unless otherwise specified
Age, mean (SD) years	
At study inclusion	42.5 (16.5)
At IBD diagnosis	25.7 (12.3)
At time of first VTE event	37.3 (16.5)
Male gender	31 (53.4)
Crohn's Disease	26 (44.8)
Active disease	6 (23.0)
Disease Location	
Ileal	1 (3.8)
Colonic	9 (34.6)
Ileo-colonic	12 (46.1)
Unknown	4 (15.4)
Perianal Disease	9 (34.6)
Disease Behavior	
Inflammatory	12 (46.1)
Stricturing	7 (26.9)
Penetrating	2 (7.7)
Unknown	5 (23.1)
Ulcerative Colitis	32 (53.4)
Active Disease	13 (40.6) (*p* < 0.01)
Disease Extent	
Proctitis	2 (6.3)
Left-sided	10 (31.2)
Extensive	16 (50.0)
Unknown	3 (9.4)
History of thrombosis	18 (31.0)
Provoked thrombotic event	10 (17.2)
Arterial thrombus	3 (5.2)
Known Thrombophilia	5 (8.6)

**Table 45 tab45:** Index thrombotic event and anticoagulation therapy regimen and laboratory parameters at time of anticoagulation initiation.

Characteristic	*n* (%), unless otherwise specified
Index thrombotic event	
Pulmonary embolus	24 (41.4)
Isolated DVT	11 (22.9)
DVT with pulmonary embolus	10 (17.2)
Portal or mesenteric thrombus	3 (5.2)
Intracranial thrombus	3 (5.2)
DVT with portal or mesenteric thrombus	5 (8.6)
DVT with PE and portal or mesenteric thrombus	1 (1.7)
DVT with PE and intracranial thrombus	1 (1.7)
Anticoagulation Regimen	
LMWH	5 (8.6)
LMWH, bridged to warfarin	10 (17.2)
LMWH, bridged to warfarin, then NOAC	6 (10.3)
LMWH, bridged to NOAC	9 (15.5)
Warfarin	11 (19.0)
Warfarin, switched to NOAC	5 (8.6)
NOAC	12 (20.7)
Laboratory parameters at time of thrombosis, mean (SD)	
Hemoglobin	120.8 (23.1)
Platelet count	280.5 (119.2)
INR	2.7 (11.3)
aPTT	30.6 (11.6)
ESR^*∗*^	30.8 (27.8)
CRP^*∗*^	31.8 (40.8)

**Table 46:** Patients response to infliximab induction.

	Clinical Response, *n* (%)	Clinical Remission, *n* (%)	Primary non-response, *n* (%)
All (*n* = 125)	90 (72)	72 (58)	35 (28)

SR			
Intensified (*n* = 38)	34 (90)^*∗*^	27 (71)^#^	4 (10)
Standard (*n* = 36)	23 (64)	18 (50)	13 (36)

SD			
Intensified (*n* = 14)	9 (64)	7 (50)	5 (36)
Standard (*n* = 37)	24 (65)	20 (54)	13 (35)

^*∗*^ *p* < 0.05 versus standard.

^#^ *p* = 0.06 versus standard.

**Table 47:** Validation of surgical procedure codes.

Procedure	Total Codes (*n*, %)	Resections (*n*, %)	PPV (95% CI)
Surgical Approach	—	—	—
Open	74 (67.3)	79 (70.5)	0.93 (0.84–0.97)
Laparoscopic	36 (32.7)	35 (31.3)	0.88 (0.72–0.96)
Surgical Urgency	—	—	—
Elective Surgery	63 (57.2)	73 (65.1)	0.90 (0.79–0.96)
Surgical Excision	—	—	—
Partial excision small intestine	55 (50.0)	57 (50.9)	0.87 (0.75–0.94)
Partial excision large intestine	37 (33.6)	38 (33.9)	0.81 (0.64–0.91)
Partial excision rectum	7 (6.4)	5 (4.5)	0.57 (0.20–0.88)
Total excision large intestine	4 (3.6)	5 (4.5)	1.00 (0.40–1.00)
Total excision rectum	7 (6.4)	7 (6.3)	0.86 (0.42–0.99)
Post Surgical Anatomy	—	—	—
Simple Excision	16 (15.1)	10 (9.0)	0.50 (0.26–0.74)
Enteroenterostomy	6 (5.7)	11 (9.9)	0.50 (0.14–0.86)
Enterocolostomy	51 (48.1)	59 (53.2)	0.88 (0.75–0.95)
Colocolostomy	5 (4.7)	7 (6.3)	0.80 (0.30–0.99)
Colo/ileorectal anastomosis	4 (3.8)	3 (2.7)	0.50 (0.09–0.91)
Stoma or pouch	24 (22.6)	21 (18.9)	0.79 (0.57–0.92)

**Table 48:** CLE image analyses.

		Active CD—Target	Active CD—Matched	Inactive CD—Target	Inactive CD—Matched	Control—Target	Control—Matched
Crypt area	Mean (SD)	8686 (4604)	7256 (3978)	9049 (2388)	8948 (2881)	8127 (2417)	7960 (1838)
	*p*	0.051		0.8618		0.8333	

Crypt grey scale density	Mean (SD)	590 (250)	395 (176)	615 (348)	500 (182)	492 (145)	470 (308)
	*p*	0.0206		0.1280		0.8960	

Crypt diameter	Mean (SD)	95 (32)	87 (28)	108 (14)	107 (18)	103 (16)	102 (10)
	*p*	0.0559		0.8608		0.9428	

Intercryptal distance	Mean (SD)	102 (41)	98 (30)	77 (17)	83 (25)	78 (20)	82 (10)
	*p*	0.8112		0.4349		0.2769	

**Table 49 tab49:** Incremental cost-effectiveness ratios for situations where adalimumab is readily available compared to when it is unavailable.

Time Horizon	Utility score of response to adalimumab measured by time-trade-off (*u* = 0.79)	Utility score of response to adalimumab measured by visual rating scale (*u* = 0.82)
5-year	$45,000 ($25,000–$65,000)	$40,000 ($22,000–$58,000)
10-year	$59,000 ($37,000–$81,000)	$53,000 ($33,000–$72,000)
15-year	$68,000 ($45,000–$91,000)	$60,000 ($40,000–$81,000)

**Table 50 tab50:** Patient demographics and clinical outcomes.

	Early Anti-TNF	Late Anti-TNF	*p*
*n* (%)	57 (49.6)	58 (50.4)	
Infliximab (%)	40 (70.2)	38 (65.5)	0.59
Adalimumab (%)	17 (29.8)	20 (34.5)	
Median time to anti-TNF (weeks, IQR)	38.1 (23.3–91.0)	414.0 (254.0–561.3)	<0.001
Active or former smoker (%)	11 (19.3)	15 (25.9)	0.76
Pancolitis (%)	43 (75.4)	40 (69.0)	0.44
Endoscopic mayo score at anti-TNF (mean, ±SD)	2.46 (±0.66)	1.86 (±0.67)	<0.001
Colectomy (%)	10 (17.5)	5 (8.6)	0.16
UC-related hospitalization	25 (43.9)	16 (27.6)	0.07
Clinical loss of response	28 (49.1)	34 (58.6)	0.31

**Table 51:** Participant assessment scores compared to published estimates.

Measure	Participant value	Published estimate	*p* value
IBD-KID (Knowledge)	15.5 (5)^*∗*^	11.3 ± 0.37^†^	0.01^‡^

MMAS-8 (Adherence)	35% scored <6 (low adherence)	52% scored <6 (low adherence)	0.23^*¥*^

TRAQ (self-advocacy/self-management)	5.3% scored ≥18/20 (≥90% mastery of skills)	5.6% scored ≥18/20 (≥90% mastery of skills)	1.0^*¥*^

^*∗*^Median (IQR), ^†^Mean ± SD, ^‡^Sign test, ^*¥*^Fisher's exact test.

**Table 52:** Contingency table for initial clinical decision made (without level knowledge), 6 month clinical outcomes (post-levels), and ITLs and FCPLs with hypothetical decision prompts^%^.

ITL	FCP	Initial clinical decision			6 month clinical outcome					
		No action	Action – investigation^*∗*^	Action – dose De-escalation^*∗∗*^	No change (stable IFX and steroid free)	Dose escalation IFX^*∗∗∗*^ (no steroids)	Dose De-escalation^*∗∗*^	Investigation^*∗*^	Hospitalization or surgery	Dose escalation^*∗∗∗*^, steroids, and hospitalization or surgery
<3.0 *μ*g/mL (Action – Dose Escalation)	<250 *μ*g/g (No Action Required)	**1/31 (3.2%)**	0	0	0	0	0	0	0	*1/31 (3.2%)*
	≥250 *μ*g/g (Action Required)	**1/31 (3.2%)**	0	0	*1/31 (3.2%)*	0	0	0	0	0

3.0–7.0 *μ*g/mL (No Action)	<250 *μ*g/g (No Action Required)	4/31 (12.9%)	0	0	4/31 (12.9%)	0	0	0	0	0
	≥250 *μ*g/g (Action Required)	**5/31 (16.1%)**	1/31 (3.2%)	0	*5/31 (16.1%)*	0	0	1/31 (3.2%)	0	0

>7.0 *μ*g/mL (Action – Dose De-escalation)	<250 *μ*g/g (No Action Required)	12/31 (38.7%)	**3/31 (9.7%)**	0	*10/31 (32.3%)*	*3/31 (9.7%)*	1/31 (3.2%)	0	*1/31 (3.2%)*	0
	≥250 *μ*g/g (Action Required)	**3/31 (9.7%)**	0	**1/31 (3.2%)**	*4/31 (12.9%)*	0	0	0	0	0

^%^A FCPL of <250 *μ*g/g should prompt “no action" while a FCPL of ≥250 *μ*g/g should prompt “action" (e.g., investigation, dose escalation etc.); ITLs <3.0 *μ*g/mL should prompt “action - dose escalation”, ITLs 3.0–7.0 *μ*g/mL should prompt “no action”, and ITLs >7.0 *μ*g/mL should prompt “action – dose de-escalation.”

^*∗*^Investigation refers to endoscopy.

^*∗∗*^Dose de-escalation refers to the dose of IFX being decreased and/or decreasing the frequency of IFX dosing.

^*∗∗∗*^Dose escalation refers to the dose of IFX being increased and/or increasing the frequency of IFX dosing.

**Table 53:** Coefficients^a^.

Variable	Unstandardized coefficients		Standardized coefficients	*t*	Sig.
	*B*	Standard error	Beta		
(Constant)	31.696	2.427		13.059	0.000
REG_Narc	16.784	4.760	0.236	3.526	0.001
PPI	−6.796	2.344	−0.194	−2.899	0.004
Female	4.844	2.321	0.140	2.087	0.038

^a^Dependent variable: LES_Basal_Mean_Pressure.

**Table 54:** 

	Control	Trypsin	Trypsin + Dyngo4a	Trypsin + Pitstop2	Elastase	Elastase + Dyngo4a	Cathepsin S	Cathepsin S + Dyngo4a
Rheobase (pA) *T* = 0	78	56^**∗∗**^	57^**∗**^	62	42^**∗∗∗**^	36^**∗∗∗**^	52^**∗∗**^	52^**∗**^

Rheobase (pA) *T* = 30	78	58^**∗**^	80^↑^	90^↑^	45^**∗∗∗**^	24^**∗∗∗**^	33^**∗∗∗**^	51^**∗**^

^↑^denotes that trypsin time 30 is blocked by inhibitors; ^*∗*,*∗∗*,*∗∗∗*^denotes rheobase decreased *p* < 0.05, *p* < 0.001, *p* < 0.0001 respectively compared to control.

**Table 55 tab55:** Results.

Pt No	MRI findings	Mn level 2009	Mn level 2015	Date of Mn Removal	Mn %Δ	ALT 2009	ALT 2015	ALP 2009	ALP 2015
1	n/a	n/a	537	January 2013	n/a	248	24	164	102
2	n/a	286	n/a	January 2013	n/a	29	35	169	109
3	Resolved	353	213	July 2009	−39.7	34	24	245	174
4	Resolved	320	187	January 2010	−41.6	33	18	86	223
5	Resolved	435	218	January 2010	−49.9	14	17	170	87
6	Abnormal	386	233	January 2010	−39.6	64	35	113	69
7	Resolved	251	116	March 2012	−53.8	13	19	203	110
8	Resolved	109	98	October 2013	−10.1	118	36	263	131
9	n/a	326	197	March 2012	−39.6	43	13	263	166
10	Resolved	1365	644	November 2009	−52.8	17	23	253	334
11	Abnormal	222	187	January 2013	−15.8	24	25	80	106

