# Supplementary material for: Program and Abstracts from the Canadian Digestive Diseases Week™ 2016
Source: Can J Gastroenterol Hepatol. 2016 Feb 23;2016:4792898. doi: 10.1155/2016/4792898 (PMC4980872; doi:10.1155/2016/4792898)
Supplement: Related texts

# Sub article: A2: Integrin **α**1**β**1 Is Controlled by the MYC Oncogenic Factor and Confers Pro-Proliferative and Pro-Migratory Advantage to Colorectal Cancer Cells

**Authors:** S. Boudjadi, G. Bernatchez, B. Senicourt, M. Beauséjour, P. Vachon, J. Carrier, J. Beaulieu

**Affiliations:** ^2.1^Université de SherbrookeSherbrookeQCCanada

*Background*. Colorectal cancer (CRC) is a multi-step process that involves successive mutation, epigenetic alteration and gene dysregulation. Integrins are a family of heterodimeric glycoproteins involved in bidirectional cell signaling and participate in the regulation of cell shape, adhesion, migration, differentiation, gene transcription, survival and proliferation. The integrin *α*1 subunit is known to be involved in RAS/ERK proliferative pathway activation and plays an important role in mammary carcinoma cell proliferation and migration. In the small intestine, *α*1 is present in the crypt proliferative compartment and absent in the villus. In mouse models, the *α*1*β*1 integrin supports breast cancer cell motility and, together with the Kras oncogenic factor, potentiates tumor growth. Very little is known about *α*1*β*1 function in CRC.

*Aims*. As we have recently shown that *α*1 is present in 65% of CRC (Boudjadi et al., 2013) and that its expression is controlled by the MYC oncogenic factor and that they correlate in 72.3% of colon adenocarcinomas (Boudjadi et al., Oncogene 2015) we postulated that integrin *α*1*β*1 has a pro-tumoral contribution in CRC related to *α*1 function.

*Methods*. *α*1*β*1 function was studied in HT29, T84 and SW480 CRC cell lines using shRNA silencing targeting *α*1 (sh*α*1) compared to an shRNA control (shCtrl). Cell proliferation was assessed by cell count and BrdU incorporation. Migration was tested by the scratch test assay. For the survival test, cells were kept in suspension without serum for 24 hours on poly-2-hydroxyethyl methacrylate (polyHEMA)-coated dishes and were then lysed and subjected to caspase3 activity measurement and cleaved PARP expression. To test tumorigenic capacity, sh*α*1 and shCtrl HT29 cells were injected into the dorsal subcutaneous tissue of female CD1 nu/nu mice. The tumor volume was assessed by external measurement. After resection, *α*1 knockdown was confirmed at the mRNA and protein levels.

*Results*. In HT29, T84 and SW480 cells, *α*1 mRNA silencing resulted in reduced cell growth and proliferation compared to the control. Caspase3 activity measurement and PARP cleaved expression in HT29 and T84 cells showed that resistance to anoikis was altered in sh*α*1 cells compared to shCtrl. Wound healing was delayed in sh-*α*1 HT29 and T84 cells compared to shCtrl. Moreover, tumor development in xenografts was reduced in HT29 sh*α*1 cells.

*Conclusions*. Our results show that *α*1*β*1 is involved in tumor cell proliferation, survival and migration. This finding suggests that *α*1*β*1 is involved in colorectal cancer progression. (Supported by the CIHR).

*Funding Agencies: *CIHR

# Sub article: A3: Role of the Phosphatase DUSP6 in the Control of Intestinal Tumorigenesis and Inflammation

**Authors:** K. Beaudry, A. Montagne, M. Langlois, S. Cagnol, N. Rivard

**Affiliations:** ^3.1^Université de SherbrookeSherbrookeQCCanada

*Aims*. The RAS/Mitogen-activated protein kinase pathway (MAPK) is an evolutionarily conserved kinase module that links extracellular signals to the machinery that controls fundamental cellular processes such as growth, proliferation, differentiation, migration and apoptosis. The phosphatase DUSP6 controls this pathway in the cytoplasm by dephosphorylating and inactivating ERK1/2 MAP kinases. To determine the role of this phosphatase in the maintenance of intestinal homeostasis, we characterized the intestinal epithelial phenotype of* Dusp6* knock-out (KO) mice under normal, oncogenic and pro-inflammatory conditions.

*Methods*. Control (*Dusp6* ^*+/+*^),* Dusp6* ^+/−^ and* Dusp6* ^−/−^ mice were sacrificed for histology, immunofluorescence [ML1] [KB2], immunohistochemistry, Western blot, and quantitative polymerase chain reaction analysis.

*Results*. Our results show that loss of DUSP6 does not alter intestinal architecture nor crypt cell proliferation (Ki67 staining). Additionally, no significant difference was observed in the number of Goblet cells (Alcian blue coloration), Paneth cells (lysozyme immunofluorescence), enteroendocrine cells (Chromogranin A staining) and enterocytes (sucrase-isomaltase expression). We tested the progression of inflammation in* Dusp6* KO mice in the acute DSS-colitis model. Our data demonstrate that* Dusp6* KO mice are protected from colitis, compared to wild-type mice, as determined by measurement of weight loss and histologic scoring. To analyze the potential involvement of DUSP6 in intestinal tumorigenesis, we crossed* Dusp6* mutant mice with *Apc* ^Min/+^ mice. Notably, a major effect on intestinal tumor initiation is observed in *Apc* ^Min/+^;* Dusp6* ^−*/*−^ mice compared to *Apc* ^Min/+^ mice. We finally knocked out DUSP6 in colorectal cancer (CRC) cells (HT29) using Crispr-Cas9 technology. The deficiency in DUSP6 in CRC cells enhanced ERK1/2 activation levels and promoted anchorage-independent growth in soft agar.

*Conclusions*. These results demonstrate that the phosphatase DUSP6, by controlling ERK1/2 activation, regulates colonic inflammatory response and protects the intestinal epithelium against oncogenic stress.

*Funding Agencies: *CIHR

# Sub article: A4: Fecal Immunochemical Testing and Fecal Calprotectin Predict Mucosal Healing in Inflammatory Bowel Disease: A Prospective Study

**Authors:** C. Ma, R. Lumb, R. Foshaug, T. Dang, S. Verma, V. Huang, K. Kroeker, K. Wong, L. Dieleman, B. Halloran, R. Fedorak

**Affiliations:** ^4.1^University of AlbertaEdmontonABCanada

*Background*. Achieving mucosal healing (MH) in patients with Crohn's disease (CD) and ulcerative colitis (UC) is associated with improved long-term outcomes but direct endoscopic assessment for MH is costly and invasive. Non-invasive biomarkers such as fecal calprotectin (FCP) and fecal immunochemical test (FIT) are potential alternatives for assessing disease activity.

*Aims*. To evaluate the accuracy of FCP and quantitative FIT for predicting endoscopic MH.

*Methods*. A prospective cross-sectional cohort study was performed in adult (≥18 years) IBD outpatients presenting for routine colonoscopy. Patients provided a first morning stool sample for FCP and FIT within 48 hours of colonoscopy. Patients on anticoagulation were excluded. MH was defined by (a) Simple Endoscopic Score for CD (SES-CD) of 0 or 1; (b) Rutgeerts score of i0 or i1; or (c) UC Endoscopic Index of Severity (UCEIS) score of 0 or 1. Receiver operating characteristic (ROC) curves were plotted for FCP, FIT, and additive combination FCP + FIT for MH.

*Results*. Eighty patients (40 CD, 40 UC) were enrolled. Patient characteristics are summarized in Table 1. Disease extent was predominantly ileal in CD (50%) and pancolonic in UC (60%). 23 patients (29%) were on biologic therapy and 50 patients (63%) had endoscopic MH. FCP < 150 *μ*g/g had a sensitivity of 0.97 for detecting MH and an area under the curve (AUC) of 0.75 (95% CI: 0.63–0.86). In comparison, FIT < 50 ng/mL was less sensitive (0.70) but more specific for MH (AUC 0.79 (95% CI: 0.69–0.90)). When used in additive combination (FCP + FIT), performance characteristics were only modestly improved (Figure 1). Combined FCP + FIT score <375 had a sensitivity of 0.80 for MH. FIT was more sensitive for predicting MH in UC compared to CD.

*Conclusions*. FCP and FIT are sensitive non-invasive methods for predicting MH in IBD patients. They may be used to rule out active disease as an alternative to endoscopic evaluation, especially in UC.

*Funding Agencies: *The Centre of Excellence for Gastrointestinal Inflammation and Immunity Research

# Sub article: A5: The Interaction between NOD2 and Smoking Is Specific to the 1007fs SNP of the NOD2 Gene in Crohn's Disease: A Systematic Review and Meta-Analysis

**Authors:** E. Kuenzig, B. Eksteen, H. Barkema, C. Seow, C. Barnabe, M. Silverberg, P. Lakatos, R. Panaccione, S. Ghosh, G. Kaplan

**Affiliations:** ^5.1^University of CalgaryCalgaryABCanada; ^5.2^Mount Sinai HospitalTorontoONCanada; ^5.3^Semmelweis UniversityBudapestHungary

*Background*. NOD2 variants and cigarette smoking are both commonly implicated risk factors for Crohn's disease (CD). The three most commonly studied single nucleotide polymorphisms (SNP) of the NOD2 gene are 1007fs, G908R, and R072W. However, only the 1007fs SNP has been confirmed as the susceptibility NOD2 gene for Crohn's disease in a genome-wide meta-analysis. Prior studies examining the interaction between NOD2 variants and cigarette smoking have reported heterogeneous findings. Because many of these studies were underpowered, the 1007fs, G908R, and R072W SNPs were often pooled together rather than evaluated individually.

*Aims*. We will examine if some of the heterogeneity observed between studies is explained by SNP-specific NOD2-smoking interactions.

*Methods*. We searched MEDLINE and EMBASE for studies that provided data on both NOD2 and cigarette smoking among patients with CD. Authors were contacted if the interaction was not reported or when the 1007fs, G908R, and R072W variants were combined. Pooled odds ratios (OR) and 95% confidence intervals (CIs) were calculated using random effects models to estimate the NOD2-smoking interaction. Smoking status was defined as ever or never. We compared the odds of ever smoking among carriers of a NOD2 mutation to those without a NOD2 mutation. All analyses were* a priori* conducted separately for the 1007fs, G908R, and R702W variants. Heterogeneity was assessed using the *I* ^2^ and Cochran *Q* statistic. Publication bias was assessed using the Begg and Mazumdar adjusted rank correlation test.

*Results*. Eighteen studies provided SNP-specific NOD2-smoking interaction data. A significant interaction between the 1007fs SNP and smoking (OR 0.70, 95% CI 0.60 to 0.82) was observed (Figure 2). Neither the G908R variant (OR 0.93, 95% CI 0.79 to 1.10) nor the R072W variant (OR 0.89, 95% CI 0.75 to 1.06) were found to have a significant interaction with smoking. Statistically significant heterogeneity and publication bias were not observed for the pooled analyses of 1007fs, G908R, or R702W.

*Conclusions*. Only the 1007fs NOD2 variant interacts with cigarette smoking in CD. Individuals with CD who have a 1007fs NOD2 mutation are less likely to smoke prior to their diagnosis. Future gene-environment studies in CD should be designed and powered to evaluate SNP-specific mutations.

*Funding Agencies: *CIHR, Alberta Innovates-Health Solutions

# Sub article: A6: Intravenous Immunglobulin-Induced Regulatory Macrophages Produce IL-10 and May Be Useful to Treat Inflammatory Bowel Disease

**Authors:** L. Kozicky, S. Menzies, L. Sly

**Affiliations:** ^6.1^UBCVancouverBCCanada

*Background*. Macrophages are key mediators of inflammation, initiating and perpetuating the innate immune response. However, macrophages can be skewed to a regulatory phenotype (Mregs), which plays an equally important role in turning off the inflammatory response. Intravenous Immunoglobulin (IVIG) is a blood product composed of pooled polyclonal immunoglobulins from more than 1000 donors. Our laboratory has reported that IVIG can skew macrophages to Mregs, which produce high amounts of the anti-inflammatory cytokine, IL-10, in response to inflammatory stimuli, like lipopolysaccharide (LPS). High dose IVIG is used to treat some autoimmune and inflammatory diseases. It may work, in part, by skewing macrophages to a regulatory phenotype and may be useful to treat intestinal inflammation, like that, which characterizes IBD.

*Aims*. My* hypothesis* is that Mregs can reduce intestinal inflammation, by producing IL-10 in response to pro-inflammatory stimuli. To address this hypothesis, I propose three specific aims:

*Aim 1*. To determine whether IVIG-induced Mregs can block innate immune-driven inflammation* in vitro* by producing IL-10.

*Aim 2*. To determine whether adoptive transfer of IL-10-producing Mregs can reduce intestinal inflammation* in vivo *in a mouse model.

*Aim 3*. To determine whether IVIG can reduce intestinal inflammation by skewing macrophages to an Mreg phenotype* in vivo *in a mouse model.

*Methods*. Macrophages were derived from mouse bone marrow aspirates and primed with IVIG to skew them to an Mreg phenotype. The ability of Mregs to reduce innate immune-mediated inflammation and its dependence on IL-10 was assessed* in vitro* in co-culture experiments. The ability of IL-10-producing Mregs to reduce intestinal inflammation* in vivo* was assessed during dextran sodium sulfate (DSS)-induced colitis. The ability of IVIG to reduce intestinal inflammation* in vivo* by skewing macrophages to an Mreg phenotype was assessed during DSS-induced colitis.

*Results*. Mregs suppressed pro-inflammatory cytokine production from LPS-stimulated macrophages in an IL-10-dependent manner. Adoptive transfer of IL-10-producing Mregs and IVIG treatment reduced clinical disease activity and histopathological features of intestinal inflammation in mice during DSS-induced colitis.

*Conclusions*. Mregs have potent anti-inflammatory activity that can be used to reduce intestinal inflammation* in vivo*. Adoptive transfer of* in vitro*-derived Mregs or skewing macrophages to an Mreg phenotype with IVIG* in situ *may provide novel immunotherapeutic strategies to treat intestinal inflammation in people with IBD. Future studies include assessing whether IVIG skews macrophages to an Mreg phenotype in patients receiving IVIG to treat autoimmune disease.

*Funding Agencies: *CCC

# Sub article: A7: Trends in Incidence of Pediatric Inflammatory Bowel Disease in Canada: Population-Based Estimates from the Canadian Gastro-Intestinal Epidemiology Consortium (CANGIEC)

**Authors:** E. Benchimol, C. Bernstein, A. Bitton, M. Carroll, W. El-Matary, A. Otley, H. Singh, G. Nguyen, A. Griffiths, D. Mack, N. Mojaverian, M. Vutcovici, Y. Cui, Z. Nugent, D. Tanyingoh, G. Kaplan

**Affiliations:** ^7.1^University of OttawaOttawaONCanada; ^7.2^University of ManitobaWinnipegMBCanada; ^7.3^McGill UniversityMontrealQCCanada; ^7.4^University of AlbertaEdmontonABCanada; ^7.5^Dalhousie UniversityHalifaxNSCanada; ^7.6^University of TorontoTorontoONCanada; ^7.7^University of CalgaryCalgaryABCanada

*Background*. The incidence of pediatric inflammatory bowel disease (PIBD) is increasing worldwide, and Canada has amongst the highest rates. Provincial population-based health administrative data can be used to determine national Canadian disease rates and compare regional trends in epidemiology.

*Aims*. To determine the incidence of PIBD in Canada, and assess trends over time.

*Methods*. We used validated algorithms to identify children <16 years diagnosed with IBD from administrative data in 5 provinces: Alberta (AB) 1999–2008, Manitoba (MB) 1999–2010, Nova Scotia (NS) 2000–2008, Ontario (ON) 1999–2010, Quebec (QC) 1999–2008. Age- and sex-adjusted incidence was calculated with 95% confidence intervals (CI) by gamma distribution. Statistical trends over time were determined using Poisson regression analyses and reported as annual percentage change. Incidence and annual percentage change were pooled and meta-analayzed across provinces using random-effects models.

*Results*. A total of 5204 cases of PIBD were newly diagnosed (3456 CD, 1438 UC). The pooled incidence of PIBD in Canada was 9.8 (95% CI 9.2–10.4) per 100,000 children. Incidence was similar amongst provinces, but higher in NS (Figure 3 [Fig fig4]). Meta-analysis of time trends revealed a non-significant rise in incidence for IBD (+2.0%/y, 95% CI −0.7 to +4.7%), CD (+1.6%/y, 95% CI −1.1 to +4.4%), and UC (+1.6%/y, 95% CI −3.7 to +7.0%). The only age subgroup with a significant increased incidence was children 0–5 y (+7.2%/y, 95% CI +2.8–11.5%). Incidence in all children increased significantly in Ontario (IBD: +5.8%/y, 95% CI +4.7–6.9%; CD: +4.8%/y, 95% CI +3.3–6.2%; UC: +6.2%/y, 95% CI +4.3–8.2%) and Quebec (CD only: +4.3%/y, 95% CI +2.3–6.2%). Incidence increased in Ontario for children in all age groups. In addition, CD increased for adolescents aged 14–15.9 y in QC (+5.7%/y, 95% CI +2.5–8.9%) but decreased in NS (−9.8%/y, 95% CI −18.6 to −0.02%).

*Conclusions*. Canada has amongst the highest incidence of PIBD in the world. Incidence was similar amongst the provinces studied, but highest in Nova Scotia. While meta-analysis demonstrated a non-significant increased incidence overall, the rate rose rapidly and significantly in the youngest children (aged 0–5 y).

*Funding Agencies: *CCC, Ontario Early Researcher Award, CIHR/CHILD Foundation Canadian Children IBD Network

# Sub article: A8: Steroid-Free Remission among Canadian Pediatric Inflammatory Bowel Disease Patients

**Authors:** P. Church, T. Walters, E. Benchimol, K. Jacobson, W. El-Matary, C. Deslandres, H. Huynh, M. Carroll, E. Wine, D. Mack, J. Van Limbergen, A. Otley, A. Griffiths

**Affiliations:** ^8.1^The Hospital for Sick ChildrenTorontoONCanada; ^8.2^University of Ottawa/CHEOOttawaONCanada; ^8.3^BC Children's HospitalVancouverBCCanada; ^8.4^University of ManitobaWinnipegMBCanada; ^8.5^Hôpital Sainte*-*JustineMontréalQCCanada; ^8.6^Stollery Children's HospitalEdmontonABCanada; ^8.7^IWK Health CentreHalifaxNSCanada

*Background*. Achieving durable remission without ongoing corticosteroid use is a measure of quality IBD care.

*Aims*. To ascertain rates of corticosteroid free clinical remission (SFR) and normal linear growth among children with established ulcerative colitis (UC) and Crohn's disease (CD) at C.H.I.L.D. Foundation/CIHR Canadian Children IBD Network sites.

*Methods*. Over 6 months, prospective data were collected on consecutive clinic patients (<18 yrs) with diagnosed IBD ≥12 months. Physicians recorded demographics; type of IBD; date of diagnosis; medications; PCDAI/PUCAI; Physician Global Assessment (PGA) of disease activity and clinical symptom pattern; appraisal of linear growth in prior 12 months. Chi-square and Kruskal-Wallis tests were used as appropriate.

*Results*. 713 patients (CD: 62%; UC: 31%; IBD-U: 7%) were reviewed at 8 sites (6 provinces). Median disease duration was 39 months (IQR 23–62). Median ages were 15.1 and 14.1 years for CD and UC respectively (*p* = 0.01). Based on PGA, 72% of CD and 78% of UC patients had inactive disease. PCDAI and PUCAI scores were <10 in 80% of CD and 84% of UC patients. 8% of CD versus 4% of UC patients had height velocity <−2SD for age. Assessment of disease activity over the preceding 6 months (continuously quiescent or minimally active in 84%) were similar for CD and UC. In the preceding 6 months, 61% of CD and 65% of UC patients were in SFR (defined as continuous absence of symptoms, PCDAI/PUCAI < 10, normal growth, with no systemic steroid use). CD and UC patients required different therapies to achieve similar rates of SFR (Table 2 [Table tab3]).

*Conclusions*. Canadian pediatric gastroenterologists minimize steroid use after the first year of diagnosis with significant use of anti-TNF therapy and immunomodulators. 5-ASA/sulfasalazine use in CD and UC appears to follow evidence-based guidelines. Rates of SFR are similar for CD and UC.

*Funding Agencies: *CIHR, C.H.I.L.D. Foundation

# Sub article: A9: A Randomized Comparison of High Definition Colonoscopy Alone with High Definition Dye Spraying Chromoendoscopy and Electronic Virtual Chromoendoscopy Using Iscan for Detection of Colonic Dysplastic Lesions during IBD Surveillance

**Authors:** M. Iacucci, M. Fort Gasia, R. Panaccione, A. Oluseyi, S. Urbanski, M. Parham, S. Ghosh

**Affiliations:** ^9.1^Gastroenterology* -*University of CalgaryCalgaryABCanada; ^9.2^Department of pathology*-*University of CalgaryCalgaryABCanada

*Background*. Dye chromoendoscopy (DCE) is currently considered the preferred endoscopic technique for IBD surveillance colonoscopy. However, the resolution of high definition (HD) and virtual chromoendoscopy (VCE) colonoscopy has increased considerably and therefore further studies are needed to determine the optimal endoscopic technique for detection of dysplastic lesions (DL).

*Aims*. Randomized trial to compare three different techniques for surveillance colonoscopy to detect colonic DL in IBD patients: (HD), (DCE) and (VCE) using iSCAN.

*Methods*. A randomized study (NCT02098798) was conducted to determine the detection rates of DL with HD alone, DCE or EVC in patients with long standing colitis (8 years from diagnosis, including both UC and CD). Consecutive patients with inactive disease were enrolled in 1 : 1 : 1 ratio into three arms of the study. Colonoscopy was performed using a Pentax EPKi processor and HD video colonoscope (EC-3490Fi; Pentax Tokyo). Endoscopic colonic lesions were classified by the Paris classification as polypoid/non-polypoid and Kudo pit pattern. The lesions were histologically categorized by the modified Vienna classification as dysplasia (ALM and DALM), sessile serrated adenomas (SSAs), adenoma-like polyps (ALP) and hyperplastic polyps (HP). Chi square test was calculated for comparison between the three arms. Sensitivity, Specificity, PPV and NPV were calculated for each arm of the study.

*Results*. 200 patients (108 male, median age 48 years, range 20–77 years) were assessed by HD (*n* = 70, 35%), VCE (*n* = 65, 32.5%) or DCE (*n* = 65, 32.5%). Twenty-eight SSAs were found in sixteen patients (20.9%); forty-three ALPs were found in thirty-eight patients (32%); six dysplastic lesions were found in five patients (4.5%). Detection rates for ALPs favored the HD group (*p* = 0.04) and when comparing between dysplastic and non-dysplastic lesions, the detection rate favored the HD group (*p* = 0.03). The three techniques had similar sensitivity and specificity in detecting DL. HD had a sensitivity of 92.5%, specificity of 78.6%, PPV 92.5% and NPV 78.6%. DCE had a sensitivity of 90.9%, specificity of 84.5%, PPV 90.9%, NPV 89.5% and VCE had a sensitivity of 91.7%, specificity of 73.3%, PPV 84.6% and NPV 84.6%.

*Conclusions*. Our results indicate that DCE, does not yield higher detection rates for colonic DL than either HD or VCE. In fact, the majority of DL were detected in the HD group.

*Funding Agencies: *None

# Sub article: A10: A Virtual Reality Curriculum in Non-Technical Skills Improves Performance in Colonoscopy: A Randomized Trial

**Authors:** S. Grover, M. Scaffidi, B. Chana, K. Gupta, M. Zasowski, O. Zarghom, C. Dargavel, T. Alomani, A. Kamani, S. Sharma, C. Walsh

**Affiliations:** ^10.1^University of TorontoTorontoONCanada; ^10.2^Hospital for Sick Children and The Wilson CentreTorontoONCanada

*Background*. Non-technical skills (NTS) are cognitive, social and personal resource skills that complement technical skills and contribute to safe and efficient task performance. Six core NTS are relevant to endoscopy: teamwork, communication, situational awareness, decision making, leadership and professionalism. The need for NTS competence is acknowledged by gastroenterology organizations such as CAG and ASGE but there is minimal evidence supporting the effectiveness of curricular NTS training.

*Aims*. To assess the effectiveness of a simulation-based curriculum in NTS on novice endoscopists' performance of simulated colonoscopy.

*Methods*. 20 novice endoscopists were randomized to 2 groups. The* conventional training group* received 6 hours of interactive small-group didactic sessions on colonoscopy theory and 6 hours of simulation-based training (SBT) that started on bench-top simulators (low fidelity) and progressed to virtual reality (VR) simulators (high fidelity). Hours 5 and 6 of SBT were integrated scenarios wherein participants interacted with a standardized patient and nurse while performing a VR simulated colonoscopy. The* NTS group* also received the same didactic sessions with hour 6 focusing on NTS, and 6 hours of SBT that progressed from low- to high-fidelity simulators, including integrated scenarios. Prior to each integrated scenario, participants reviewed a checklist of relevant core NTS concepts. Participants were assessed at baseline, immediately after training, and 4–6 weeks post-training. The primary outcome was NTS performance during an integrated scenario test, measured by OSANTS, an assessment tool for NTS in surgery modified for endoscopy. Secondary outcomes were attitudes towards NTS measured by TEAMSTEPPS, a validated questionnaire of NTS perception; and global performance and communication during integrated scenarios respectively assessed using ISGRF and ISCRF, two previously validated rating scales.

*Results*. The NTS group outperformed the conventional training group on the integrated scenarios immediately after training and 4–6 weeks after training, in terms of NTS-specific performance (*p* < 0.003), global performance (*p* < 0.04) and communication (*p* < 0.01). The NTS group regarded NTS more positively as compared to the conventional training group (*p* < 0.003).

*Conclusions*. A colonoscopy simulation-based curriculum focused on NTS improved NTS performance, communication and global performance during simulated colonoscopy encounters, and attitudes regarding NTS. Further research should evaluate the impact of a NTS curriculum on clinical colonoscopy performance.

*Funding Agencies: *None

# Sub article: A11: Cost-Effectiveness of Hemospray™ in Patients With Non Variceal upper Gastrointestinal Bleeding

**Authors:** A. Barkun, V. Adam, Y. Chen, Y. Lu, M. Martel

**Affiliations:** ^11.1^McGill UniversityMontrealQCCanada

*Background*. Hemospray (TC-325) is an endoscopic hemostatic powder that achieves hemostasis through adherence to actively bleeding biological surfaces.

*Aims*. Compare the cost-effectiveness of traditional endoscopic hemostatic therapies (except epinephine injection alone) and Hemospray in different combinations.

*Methods*. A decision tree of patients with active Non Variceal Upper Gastrointestinal Bleeding (NVUGIB) assessed four possible treatment strategies: traditional therapy alone (T), Hemospray alone (H), traditional therapy completed by Hemospray if needed (T + H), or Hemospray completed by traditional therapy if needed (H + T). Using published probabilities, effectiveness was the likelihood of avoiding rebleeding over 30-day. Costs in 2014US$ were based on the US National Inpatient Sample. Physician and procedure fees were obtained from the American Medical Association and recent publications. A third-party payer perspective was adopted. Sensitivity and subgroup analyses were performed.

*Results*. For all patients, T + H is more efficacious and less expensive than all other approaches, with 97% of patients eventually avoiding rebleeding at an average cost per patient of US$9,150. The second most cost-effective approach is H + T, 5.57% less effective and costing on average US$635 more per patient. Sensitivity analyses show that T + H followed by a strategy of H + T remain more cost-effective than H or T alone when varying all probability assumptions across plausible, a priori determined ranges. Variations in physician, procedural fees and in the price of Hemospray do not change the final selection of preferred strategy. Varying four assumptions of disease-specific lengths of stay make T less costly than T + H (T + H still more effective). Subgroup analyses showed that patients with non-ulcer lesions at low risk of delayed rebleeding, the Hemospray first approach (H + T) was most effective at low incremental cost ($341 per patient) compared to T + H; the T + H strategy was most effective with varying costs differences relative to the different strategies for all other subgroups. It is more cost-effective to deliver T + H or H + T at the same endoscopy session than at a second-look endoscopy. A strategy of H alone appears ineffective in peptic ulcer bleeding.

*Conclusions*. Hemospray improves the effectiveness of traditional hemostasis, while being less costly in most patient populations presenting with NVUGIB; a Hemospray first approach may be the most cost-effective for non-ulcer bleeding lesions at low risk of delayed hemorrhage.

*Funding Agencies: *None

# Sub article: A12: New Oral Anticoagulants and Gastrointestinal Hemorrhage: A Systematic Review and Meta-Analysis

**Authors:** A. Dorreen, C. Miller, M. Martel, A. Barkun

**Affiliations:** ^12.1^Dalhousie UniversityHalifaxNSCanada; ^12.2^McGill UniversityThe Montreal General HospitalGI DivisionMontrealQCCanada; ^12.3^McGill University Health CenterMontrealQCCanada

*Background*. Several new oral anticoagulants (NOACs) have been approved for clinical use or are in advanced-phase clinical trials, yet evidence regarding associated risk of gastrointestinal hemorrhage (GIB) is limited.

*Aims*. To determine the risk of GIB associated with NOACs as compared to conventional anticoagulation therapy.

*Methods*. An initial search for randomized controlled trials comparing NOACs to conventional anticoagulation therapy was performed using the EMBASE, Medline, Cochrane and ISI Web of knowledge databases from inception through March 2015. NOACs already approved or in active development were included. Trials assessing NOACs for the treatment of acute coronary syndrome and other unapproved indications were excluded. Two independent reviewers analyzed abstracts and reviewed manuscript content. Data from relevant papers, including baseline characteristics, indication for and duration of NOAC and number, severity and location of GIB events were compiled. A meta-analysis was conducted with results reported as odds ratios (OR) with 95% confidence intervals (CI). The primary outcome was major GIB. Secondary outcomes included clinically-relevant non-major (CRNM), upper and lower GIB. A subgroup analysis of individual NOACs was performed. Heterogeneity and publication bias were assessed.

*Results*. An initial search yielded 1654 papers, following review 36 trials were included that assessed dabigatran, rivaroxaban, apixaban, edoxaban and betrixaban. A total of 145,639 patients were randomized. There was no difference in major GIB between NOACs and conventional anticoagulation (OR 0.98, 95% CI: 0.80–1.22). No difference was observed for CRNM GIB (OR 0.92, 95% CI: 0.63–1.34), upper GIB (OR 0.76, 95% CI: 0.37–1.56) or lower GIB (OR 0.86, 95% CI: 0.66–1.13). Subgroup analysis revealed an increased odds of major GIB with dabigatran (OR 1.27, 95% CI: 1.04–1.55) and rivaroxaban (OR 1.40, 95% CI: 1.15–1.70) when compared to conventional anticoagulation.

*Conclusions*. No difference was found between NOACs and conventional anticoagulation regarding odds of major GIB. Subgroup analysis, however, indicates that dabigatran and rivaroxaban are significantly associated with a 27% and 40% relative increase in odds of major GIB, respectively.

*Authors' Contribution*. C. Miller & A. Dorreen are co-first authors.

*Funding Agencies: *None

# Sub article: A13: The Gut Microbiota-Dependent Metabolite, TMAO, Protects against Colitis: A Role for the Inhibtion of Apoptosis?

**Authors:** A. Al Rajabi, A. Wang, D. McKay

**Affiliations:** ^13.1^University of CalgaryCalgaryABCanada

*Background*. Some patients with IBD exhibit signs of endoplasmic reticulum (ER; i.e., unfolded protein response) stress in their intestinal gut epithelium. Also, mutations in multiple genes that encode proteins associated with the ER stress response have been identified as risk factors for IBD development. Unresolved ER stress can lead to apoptosis, which in turn may cause a transient increase in gut permeability, and loss of barrier function is often a hallmark of IBD. Trimethylamine-*N*-oxide (TMAO), a gut microbiota-dependent metabolite of dietary choline, is a low molecular weight chaperone that can facilitate proper protein folding, thereby, attenuating ER stress.

*Aims*. To test if TMAO can ameliorate colitis development by means of preventing ER stress and ER stress-induced apoptosis.

*Methods*.* In vivo*- CD1 male mice (8–10 weeks old) were given free access to 3% (w/v) dextran sodium sulfate (DSS) in drinking water for 5 days followed by 3 days of normal drinking water ± TMAO (days 0–7; 35 mg/day; oral gavage) and necropsy performed on day 8. Controls received normal drinking water only and both negative and positive (i.e., DSS) controls received water via gavage. Daily body weight was recorded. Colitis was assessed by disease activity score (DAS), colon length and MPO activity.* In vitro*- Caco-2 (human colon-derived epithelial) cells were treated with tunicamycin (10 *μ*g/mL), an ER stressor, ± TMAO and apoptosis was assessed by immunoblotting for cleaved caspase-3 and cleaved PARP.

*Results*.* In vivo*- In 2 separate experiments (*n* = 6-7 mice/group), TMAO-treated mice displayed significant protection against DSS-induced colitis as gauged by colon length, tissue MPO levels and the cumulative DAS.* In vitro*- as expected tunicamycin induced apoptosis in Caco-2 cells at 20–48 h post-treatment incubation and levels of cleaved caspase-3 and PARP were significantly reduced in the TMAO (50 mM) co-treated epithelia.

*Conclusions*. The gut microbiota can be both cause and “cure” of intestinal inflammation. Our data indicate that the microbial metabolite, TMAO, inhibits DSS-induced colitis/disease in mice (possibly via inhibition of ER stress-induced apoptosis). Thus, TMAO could be a novel therapeutic in IBD and may be of particular value in individuals whose disease is characterized by ER stress.

*Funding Agencies: *CIHR, Eyes High Postdoctoral Fellowship

# Sub article: A14: Effects of Milk Lipid Globule Membrane on Post-Natal Intestinal Development

**Authors:** G. Bhinder, R. Dyer, S. Innis, B. Vallance

**Affiliations:** ^14.1^University of British ColumbiaVancouverBCCanada

*Background*. At birth, there is a drastic change in nutrient acquisition in a switch from placental nutrition transfer to the introduction of food. Although breastmilk is the ideal nutrient source during the first 6 months after birth it is often not available in great enough quantity, if at all, to the developing infant, resulting in a need for formula feeding. As achieving appropriate growth during this early time of development is essential, it is of utmost importance that the composition of formula reflects that of breastmilk as closely as possible. Interestingly, the lipid fraction of breastmilk, composed of a triglyceride core surrounded by a unique triple membrane structure-the Milk Lipid Globule Membrane (MLGM), represents the main source of energy for the newborn. Surprisingly, most available formulas do not contain this MLGM component, but rather derive their lipids from vegetable sources. To date, the ability of MLGM to effect development, specifically at the intestinal surface, has not been extensively explored.

*Aims*. To examine the effects of Milk Lipid Globule Membrane supplementation in formula on post-natal intestinal development.

*Methods*. The rat pup-in-a-cup model was utilized to examine the effects of MLGM (1.2 or 6 mg/mL) supplementation on early intestinal development. In brief, rat pups underwent gastronomy at postnatal day (PD) 5 and were supplemented with formula containing soy (control) or MLGM + soy until PD15, at which point they were euthanized. Ileal and distal colonic samples were collected for histological assessment and immunohistochemistry (IHC), while stool samples were collected for assessment of commensal microbes.

*Results*. MLGM supplementation in soy formula resulted in a dose dependent increase in colonic crypt depth at PD15, with the 6 mg/mL MLGM + soy group displaying similar crypt depth to mother-reared pups. IHC analysis of colonic intestinal mucus (Muc2+), epithelial cell proliferation (Ki-67+), and enterocyte numbers (CA-1+) revealed similar positive staining at PD15 between mother-reared and 6 mg/mL MLGM + soy supplemented pups compared to soy formula alone, while enteroendocrine (5-HT+) numbers were similar between all three groups. No overt differences in commensal microbes were found between MLGM + soy and soy formula alone supplemented rat pups.

*Conclusions*. Milk Lipid Globule Membrane supplementation in formula accelerates early intestinal development, resulting in similar colonic proliferation and mucus production to that observed in mother-reared pups. This accelerated development may be protective against early enteric infections, suggesting that MLGM supplementation may be beneficial in neonates who do not have access to breastmilk, particularly pre-term infants who are especially vulnerable to infections.

*Funding Agencies: *CIHR

# Sub article: A15: Mesotrypsin Evokes PAR2 Dependent Excitabilty of Nociceptive Dorsal Root Ganglia (DRG) Neurons

**Authors:** C. Lopez Lopez, J. Jaramillo Polanco, C. Rolland-Fourcade, N. Vergnolle, S. Vanner

**Affiliations:** ^15.1^Queen's UniversityKingstonONCanada; ^15.2^INSERM UMR-1043ToulouseFrance

*Background*. Mesotrypsin protein and mRNA levels in colonic epithelium are increased in Irritable bowel syndrome (IBS) patients. Our previous studies have shown that proteases in supernatants from IBS-D patients elicited a marked increase in nociceptive dorsal root ganglia neuronal excitability by activating PAR2 but it is unknown if mesotrypsin could play a role in this action.

*Aims*. This study examined whether mesotrypsin increased the excitability of nociceptive DRG neurons and whether PAR2 signaling was involved.

*Methods*. Nociceptive mouse DRG neurons (capacitance < 30 pF) were incubated with mesotrypsin (10 nM, 20 min) or trypsin (10 nM, 10 min), to provide a comparator to the known actions of other proteases. In order to evaluate if the protease effect in cell excitability was mediated by PAR2, neurons were incubated with the PAR2 specific antagonist GB83 (10 *μ*M, 30 min) prior to the incubation with mesotrypsin or trypsin. The excitability of neurons was measured by perforated patch clamp recordings by recording changes in the rheobase and action potential discharge at twice rheobase.

*Results*. Mesotrypsin evoked increased excitability (rheobase decreased 29%, *p* < 0.01, and the action potential number at twice rheobase increased 29%, *p* < 0.05) compared to controls. The effect of trypsin on neuronal excitability was similar to that observed with mesotrypsin (rheobase decreased by 26%, *p* < 0.01, and the action potential number at twice rheobase increased by 41%, *p* < 0.01) compared to control, as previously reported (Valdez Morales et al., 2013). In a separate series of experiments, the effects of mesotrypsin and trypsin were blocked by the PAR2 antagonist GB83. Mesotrypsin or trypsin plus GB83 treated cells showed no difference versus control cells, whereas both protease alone caused increased excitability (mesotrypsin rheobase 29% lower than mesotrypsin plus GB83, *p* < 0.01, trypsin rheobase 24% lower than trypsin plus GB83, *p* < 0.01).

*Conclusions*. These data suggest that epithelial derived mesotrypsin induces hyperexcitability of mouse DRG neurons in a PAR2 dependent fashion. Taken together with our previous studies demonstrating a PAR2 dependent role for proteases in IBS pain signaling, this data suggests that mesotrypsin could be one of the important mediators.

*Funding Agencies: *CIHR

# Sub article: A16: VacA-Disrupted Autophagy Promotes Accumulation of* Helicobacter pylori* Cytotoxin Associated Gene A during Chronic Infection

**Authors:** M. Abdullah, L. Greenfield, M. Capurro, C. O'Brien, N. Jones

**Affiliations:** ^16.1^Cell Biology ProgramResearch InstituteThe Hospital for Sick ChildrenTorontoONCanada; ^16.2^Princess Margaret Cancer CentreUniversity Health NetworkTorontoONCanada

*Background*. Infection with* Helicobacter pylori* is the most important risk factor for the development of gastric cancer. The cytotoxin associated gene A (CagA) and vacuolating cytotoxin (VacA) are major virulence determinants of* H*.* pylori*. The mechanisms regulating these virulence factors in host cells are important for understanding pathogenesis. We have shown that prolonged VacA exposure disrupts the autophagy pathway. Recent studies suggest that CagA, which is considered an oncoprotein, can be degraded by autophagy. Furthermore, alterations in autophagy can impact the ubiquitin-proteasome system (UPS), another mechanism responsible for degrading cellular proteins. We hypothesized that during chronic* H*.* pylori *infection, VacA-disrupted autophagy promotes CagA accumulation leading to enhanced downstream oncogenic CagA signaling.

*Aims*. Here we investigated the mechanisms by which autophagy modulates CagA in host cells. The objectives were to (1) determine if VacA-disrupted autophagy increases intracellular CagA levels, and (2) determine if disrupted autophagy impacts proteasomal degradation of CagA.

*Methods*. Human gastric epithelial (AGS) cells and human gastric organoids were infected with the* vacA *isogenic mutant* H*.* pylori* and co-cultured with or without VacA+ or VacA− cultured conditioned media supernatant (CCMS) for up to 48 hours using a gentamycin assay. Wild-type (WT) and autophagy deficient (*atg5* ^−*/*−^) mouse embryonic fibroblasts (MEF) were infected with the* vacA* isogenic mutant* H*.* pylori *to determine the effects of autophagy on intracellular CagA in the absence of VacA. Western blotting was performed to assess CagA levels. Bacterial viability assays were performed to control for differences in survival over time and normalize CagA levels.

*Results*. When* vacA*−* H*.* pylori *infected AGS cells were co-cultured with VacA+ CCMS, there was a significant increase in normalized CagA levels. These findings were recapitulated in human gastric organoids.* Atg5* ^−*/*−^ MEFs infected with* vacA*−* H*.* pylori* showed increased CagA levels compared to WT MEFs. Of note, CagA levels decreased over time even in the* atg5* ^−*/*−^ MEFs. AGS cells infected with* vacA*−* H*.* pylori* had a 4-fold increase in CagA levels when treated with the proteasome inhibitor, MG132, compared to vehicle control. However, proteasome inhibition of VacA+ CCMS treated cells did not increase the levels of CagA.

*Conclusions*. Taken together, our findings reveal that VacA− disrupted autophagy may modulate the UPS, which both lead to an accumulation of CagA.

*Funding Agencies: *CIHR

# Sub article: A17: The Role of SHP-1 as a Tumor Suppressor Gene in Intestinal Epithelium

**Authors:** C. Leblanc, G. Coulombe, N. Rivard

**Affiliations:** ^17.1^Université de SherbrookeSherbrookeQCCanada

*Background*. SHP-1, a src homology 2 (SH2) domain containing protein tyrosine phosphatase, functions as a negative regulator of signaling downstream of cytokine receptors, receptor tyrosine kinases and receptor complexes of the immune system. Additionally, SHP-1 has been proposed to be a tumor suppressor gene for several cancers. Of note, we demonstrated that this phosphatase negatively regulates the nuclear transcriptional function of *β*-catenin in intestinal epithelial cells in culture (Simoneau, Cell Signalling 2011).

*Aims*. These studies suggest that SHP-1 might exert a tumor suppressive action in the intestinal epithelium.

*Methods*. Colorectal cancer (CRC) samples paired with their margins were used to analyse relative expression of PTPN6. CRC cell lines were infected with lentivirus coding for a shRNA against SHP-1. Mice with a specific deletion of SHP-1 in intestinal epithelial cells (IECs) were generated using the cre-loxP system. These mice were then breeded with *APC* ^Min/+^ mice.

*Results*. SHP-1 gene expression was investigated by quantitative analysis in paired samples of CRC (resection margins and primary tumors). Importantly, relative amounts of SHP-1 transcripts are effectively found to be significantly reduced in these colorectal tumors compared to corresponding normal specimens. SHP-1 silencing in human CRC cells (HCT116, HT29) enhances BrdU incorporation in comparison to control cells, suggesting an increased growth rate for these cells. Additionally, SHP-1-depleted CRC cells form significantly more colonies in soft agar. Conversely, ectopic expression of wild-type SHP-1 inhibits the capacity of CRC cells to grow under anchorage-independent conditions. In mice, conditional deletion of SHP-1 in intestinal epithelium leads to an intestinalomegaly associated with an increase in crypt depth and cell proliferation. A marked increased expression of *β*-catenin protein and Akt phosphorylation is also observed in IECs from mutant mice. While loss of epithelial SHP-1 is not sufficient by itself to initiate tumorigenesis in mice, it severely enhances intestinal tumor load in *Apc* ^Min/+^ mice.

*Conclusions*. These results reveal an anti-proliferative function for SHP-1 in IECs and suggest that this phosphatase functions as modifier gene in colorectal neoplasia.

*Funding Agencies: *NSERC

# Sub article: A18: Prevalence and Factors Associated with Nonalcoholic Fatty Liver Disease as Diagnosed by Transient Elastography with Controlled Attenuation Parameter in HIV Mono-Infected Patients

**Authors:** E. Vuille-Lessard, L. Lennox, C. Pexos, B. Lebouche, M. Klein, G. Sebastiani

**Affiliations:** ^18.1^McGill University Health CenterMontrealQCCanada

*Aims*. Nonalcoholic fatty liver disease (NAFLD) is the most frequent liver disease in Canada and may significantly contribute to mortality among HIV-infected persons. Due to the invasiveness of liver biopsy, data on NAFLD in HIV mono-infected patients are scarce. We investigated prevalence and predictors of NAFLD and liver fibrosis in a large cohort of HIV mono-infected patients, without coinfection with hepatitis B or C, by transient elastography (TE)/controlled attenuation parameter (CAP).

*Methods*. This was a prospective cohort study at McGill University Health Centre, which included 310 consecutive HIV mono-infected persons (mean age 49.9 years, 77% men). Patients with significant alcohol intake or coinfection with hepatitis B or C were excluded. Any grade NAFLD (>10% of hepatocytes), significant NAFLD (>30%) and severe NAFLD (>60%) were defined as CAP > 232, CAP > 260 and CAP > 292 dB/m, respectively. Significant liver fibrosis and cirrhosis were defined as TE measurement >8 kPa and >13 kPa, respectively. Predictors of NAFLD and liver fibrosis were determined by multivariate logistic regression models.

*Results*. CAP identified any grade NAFLD, significant NAFLD and severe NAFLD in 55.3%, 33.7% and 16.3% of cases, respectively. Significant liver fibrosis and cirrhosis were found in 11% and 2.3% of cases, respectively. Multivariate analysis results are reported in Table 4 [Table tab5] [Table tab6]. A model combining the identified predictors for significant NAFLD (overweight, protease inhibitors and elevated ALT) showed that presence of at least two predictors had 100% sensitivity to rule in significant NAFLD.

*Conclusions*. NAFLD diagnosed by TE with CAP is frequent in HIV mono-infected persons, particularly in those with overweight, elevated ALT and exposed to protease inhibitors as antiretrovirals. Of note, significant NAFLD is a predictor of significant liver fibrosis. Longitudinal studies are needed to evaluate the impact of interventions, such as weight loss, aimed at reducing morbidity and mortality due to liver disease in this population.

*Funding Agencies: *CIHR Canadian HIV Trials Network; unrestricted research funds from ViiV; unrestricted research funds from Merck, study number IIS#51841

# Sub article: A19: Minimal Hepatic Encephalopathy Renders the Brain Susceptible to Hypotension-Induced Neuronal Cell Loss In BDL Rats

**Authors:** M. Clément, C. Bosoi, M. Tremblay, C. Bemeur, C. Rose

**Affiliations:** ^19.1^CRCHUMMontréalQCCanada; ^19.2^Université de MontréalMontrealQCCanada

*Background*. Hepatic encephalopathy (HE) is a major neuropsychiatric complication caused by liver disease characterized by cognitive and motor dysfunction. Historically, HE has always been considered to be a reversible metabolic disorder and has therefore been expected to completely resolve following liver transplantation (LT). However, persisting neurological complications remain a common problem affecting as many as 47% of LT recipients. LT is a major surgical procedure accompanied by intraoperative stress, including blood loss and hypotension.

*Aims*. We hypothesize, in the setting of minimal HE (MHE), the compromised brain becomes susceptible to hypotensive insults, resulting in cell injury and death.

*Methods*. Six-week bile-duct ligated (BDL) rats with MHE and respective controls (SHAM) were used. Blood is withdrawn from the femoral artery (inducing hypovolemia) until a mean arterial pressure of 30, 60 and 90 mmHg (hypotension) and maintained for 120 minutes. Cerebral blood flow (BCF) was assessed by injecting fluorescent microspheres through the brachial artery. Upon sacrifice, brains were extracted for apoptotic analysis (western blot) and neuronal cell count (immunohistochemistry). In a separate group, BDL rats were treated for MHE with ornithine phenylacetate (OP; OCR-002), administered orally (1 g/kg) for 3 weeks.

*Results*. Both BDL rats and SHAM-operated controls without hypotension did not display any cell injury or neuronal loss. However, BDL rats following hypotension (30 and 60 mmHg) demonstrated a significant decrease in neuronal cell count in the frontal cortex (using NeuN + DAPI and Cresyl Violet) compared to hypotensive SHAM-operated controls. In addition, neuronal loss was associated with an increased in cleaved caspase-3, suggesting apoptotic cell death. CBF decreased in BDL rats compared to SHAM and correlated with degree of hypotension insult. BDL rats treated with OP resulted in a decrease in blood ammonia and improvement in behaviour and did not lead to neuronal cell death following hypotension.

*Conclusions*. These findings strongly suggest that cirrhotic patients with MHE are more susceptible to hypotension-induced neuronal cell loss. Moreover, these results suggest a patient with HE (even MHE), with a “frail brain”, will fare worse during liver transplantation and consequently result in poor neurological outcome. Combination of MHE and hypotension may account for the persisting neurological complications observed in a number of cirrhotic patients following LT. Therefore, MHE, should not to be ignored and merits to be treated in order to reduce the risk of neurological complications occurring post-LT.

*Funding Agencies: *CIHR

# Sub article: A20: Risk Factors and Otcomes of Non-Skin Cancers after Liver Transplantation for Primary Sclerosing Cholangitis

**Authors:** M. Mouchli, S. Singh, J. Talwalkar, E. Loftus, C. Rosen, J. Heimbach, R. Wiesner, K. Watt, J. Poterucha

**Affiliations:** ^20.1^Mayo ClinicRochesterMNUSA

*Background*. PSC is associated with significantly increased risk of cancer (Ca) and related mortality. It is unclear how liver transplantation (LT) for PSC modifies this risk.

*Aims*. To determine the cumulative incidence of and risk factors for Ca and long-term cancer-related mortality in patients (pts) with PSC undergoing LT.

*Methods*. We identified all pts who underwent LT for advanced stage PSC for non-cholangiocarcinoma indications at Mayo Clinic between 1984–2012 with follow-up through 2/2015. Information on Ca incidence and Ca-related mortality in pts with PSC were extracted. Non-melanoma skin Ca were not included in the analysis. The 1-, 5-, 10- and 20-yr cumulative risks of Ca were estimated using Kaplan-Meier curves. Risk factors were assessed using multivariate Cox proportional hazard analysis.

*Results*. Two hundred ninety-three pts (mean age, 47 ± 12 yrs; 63.3% males, 2.4% smoking at time of LT). Over a median follow-up of 11.5 yrs (6.4–18.6), 60 pts (20.5%) developed 70 non-skin Ca (8 pts developed 2 Ca and 1 pt developed 3 Ca). The most commonly observed Ca were: 48 solid-organ Ca (11 renal, 11 colorectal, 7 prostate, 6 pancreatic, 5 breast, 3 ovarian/endometrial/vulvar, and 1 hepatocellular carcinoma), and 4 patients with recurrent PSC developed* de novo* cholangiocarcinoma. 22 hematological malignancies occurred (18 PTLD, 2 Hodgkin's disease, and 2 myelodysplastic syndrome). The 1-, 5-, 10- and 20-yr cumulative incidences of Ca were 1.7%, 6.8%, 14.0% and 17.7%, respectively. Median survival of pts who developed Ca was reduced compared to PSC pts without Ca (9.6 versus 22.1 years, *p* < 0.01). On multivariate Cox proportional hazard analysis, mycophenolate mofetil use, tacrolimus-based immunosuppression, and male sex were associated with increased risk of non-skin Ca.

*Conclusions*. The 10-year cumulative risk of Ca after LT for advanced stage PSC was 14.0% with decrease in overall survival. Mycophenolate mofetil use, tacrolimus-based immunosuppression, and male sex were associated with increased non-skin Ca risk.

*Funding Agencies: *None

# Sub article: A21: Cirrhotic Patients with Sarcopenia and Sarcopenic-Obesity Have an Increased Risk of Hyperammonemia and Hepatic Encephalopathy

**Authors:** A. Montano-Loza, A. Duarte-Rojo, R. Bhanji, C. Rose

**Affiliations:** ^21.1^University of AlbertaEdmontonABCanada; ^21.2^University of Arkansas for Medical SciencesLittle RockARUSA; ^21.3^Hôpital St*-*LucCRCHUMUniversité de MontréalMontrealQCCanada

*Background*. Muscle mass functions as an alternative site of ammonia detoxification in patients with cirrhosis.

*Aims*. In this study we aimed to investigate if sarcopenia, myosteatosis, and obesity are associated with hyperammonemia and hepatic encephalopathy (HE) in patients with cirrhosis.

*Methods*. A total of 204 cirrhotic patients were studied. Muscularity assessment was analyzed using CT scans at the level of the 3rd lumbar vertebral body. Sarcopenia was defined using the skeletal muscle index and myosteatosis according to the muscle attenuation index. Overweight-obesity was defined as a body mass index ≥25 kg/m^2^. Sarcopenic-obesity was defined as concomitant sarcopenia and overweight-obesity. HE was evaluated clinically (West-Heaven criteria) and defined as absent in patients not using specific treatment (i.e., lactulose, rifaximin) and with no prior episodes of HE in the preceding year. Ammonia blood levels were also performed (nl. 0–35 *μ*mol/L) at the time of the muscularity assessment.

*Results*. Mean age was 56 ± 1 years and 141 were males (69%). Sarcopenia was noted in 96 patients (47%), 137 had myosteatosis (67%), 136 were overweight-obese (67%), and 53 (28%) had sarcopenic-obesity. Patients with sarcopenia (87 ± 6 versus 61 ± 4 *μ*mol/L, *P* < 0.001), and sarcopenic-obesity (95 ± 9 versus 40 ± 3 *μ*mol/L, *P* < 0.001) had higher levels of ammonia (Figure 5). Levels of ammonia were not different among patients with myosteatosis (77 ± 5 versus 67 ± 5 *μ*mol/L, *P* = 0.2), and overweight-obesity (76 ± 5 versus 67 ± 5 *μ*mol/L, *P* = 0.2). Patients with sarcopenia (84 versus 63%, *P* = 0.001) and sarcopenic-obesity (93 versus 65%, *P* < 0.001) had higher frequency of hyperammonemia. Patients with myosteatosis had a trend (*P* = 0.06), and overweight-obesity was not associated with hyperammonemia (*P* = 0.1). Lastly, patients with sarcopenia (42 versus 26%, *P* < 0.001), and sarcopenic obesity (47 versus 30%, *P* < 0.001) had higher frequency of HE. Sarcopenia and sarcopenic-obesity increased the risk of hyperammonemia (OR 3.2, *P* = 0.001, and OR 7.0, *P* < 0.001). Also, sarcopenia increased the risk of HE (OR 2.0, *P* < 0.001, and OR 2.1, *P* < 0.001).

*Conclusions*. Cirrhotic patients with sarcopenia and sarcopenic-obesity have higher ammonia levels and risk for HE. Muscle mass plays a protective role for hyperammonemia and therapeutic strategies to avoid muscle depletion might decrease the risk of HE in cirrhosis.

*Funding Agencies: *This study has been funded with a Clinical Research Award from the American College of Gastroenterology Institute 2011.

# Sub article: A22: Reduced Hepatic PGC-1**α** Leads to Oxidative Stress and Worsened Nafld Progression

**Authors:** A. Besse-Patin, J. Estall

**Affiliations:** ^22.1^Institut de Recherches Cliniques de MontréalMontrealQCCanada

*Background*. Non-alcoholic fatty liver disease (NAFLD) is a better predictor of type 2 diabetes than anthropometric parameters, yet diagnosis is difficult and mechanisms underlying this condition are not fully understood. Inefficient mitochondrial fatty acid oxidation and increased reactive oxygen species (ROS) production link mitochondrial health to hepatic insulin resistance and NAFLD. PGC-1*α* is a transcriptional co-activator shown to regulate mitochondrial function and inflammation. In patients with NAFLD, hepatic PGC-1*α* expression is decreased, correlating with increased liver fat levels and insulin resistance.

*Aims*. A causative role or mechanistic link between reduced PGC-1*α*, hepatic insulin resistance and NAFLD progression has not been investigated. We hypothesized that low hepatic PGC-1*α* potentiates NAFLD* in vivo *by increasing ROS to cause insulin resistance.

*Methods*. Wild Type (WT), Liver Heterozygote (LH), and liver-specific knock out (LKO) mice (males and females) were fed either a chow diet or high fat diet supplemented with fructose (30%) for 25 weeks.

*Results*. We show that reduced hepatic PGC-1*α* did not alter hepatic inflammatory pathways or lipid content in chow-fed mice. However, when combined with a western diet, a 50% reduction of hepatic PGC-1*α* (LH) increased hepatic lipids, liver inflammation and oxidative damage. Interestingly, effects were sex-dependent (pathology was more pronounced in female mice) and unexpectedly, mice with a complete knock-out of PGC-1*α* in liver were similar to wild-type. To understand the mechanistic link between low PGC-1*α* and liver pathology, we analyzed ROS production (ROS-sensitive CM-H2DCFDA) and ROS-induced oxidative damage* in vitro* and found it significantly increased in LH hepatocytes, whereas there were no differences in LKO hepatocytes, agreeing with* in vivo* data. However, oxidative damage was restored when the related PGC-1b was also deleted in LKO hepatocytes, suggesting compensation by other family members in a total KO. Consistent with a causative link between increased ROS and insulin sensitivity, we show in primary hepatocytes that reduction of insulin-induced Akt activation following PGC-1*α* depletion was not dose-dependent. LH primary hepatocytes exhibited decreased phospho-Akt in response to insulin, while complete loss of PGC-1*α* lead to significantly increased phosphorylation of Akt compared to WT controls.

*Conclusions*. This work demonstrates that reduced liver PGC-1*α* can worsen fatty liver disease progression towards steatohepatitis when exacerbated by environmental factors such as diet. These data also suggests that PGC-1-dependent ROS production may be a significant contributing factor to hepatic insulin resistance in NAFLD.

*Funding Agencies: *CIHR

# Sub article: A23: Post-Transplant Cholestasis within 1-Year Predicts PSC Recurrence

**Authors:** S. Wasilenko, E. Lytvyak, A. Montano-Loza, A. Mason

**Affiliations:** ^23.1^University of AlbertaEdmontonABCanada

*Background*. Primary sclerosing cholangitis (PSC) is a chronic cholestatic liver disease affecting both the intrahepatic and extrahepatic biliary tree of which liver transplant is the only effective cure. PSC recurrence (rPSC) after liver transplant significantly affects long-term graft survival and occurs in 6–59% of transplanted patients. Numerous risk factors for recurrence have been proposed however findings are not reproducible by independent groups. We addressed the hypothesis that rPSC has similar dynamic changes in LFTs within the first year following liver transplant, as seen in patients with viral hepatitis, and that LFT changes may identify patients more likely to develop disease recurrence.

*Aims*. To determine if the development of cholestasis in the first 12 months after transplant subsequently predicts remote rPSC.

*Methods*. PSC patients who underwent liver transplant at the University of Alberta Hospital from 1991 to 2012 were included. All data was obtained from electronic medical records. Diagnosis of recurrence was defined on the basis of cholangiography and/or histological findings consistent with rPSC. Cholestasis was evaluated at 3, 6, 9, and 12 months after liver transplant. Severe cholestasis was defined as bilirubin ≥ 100 *μ*mol/L and/or alkaline phosphatase (ALP) ≥ 3XULN. Mild cholestasis was defined as those without severe cholestasis and (i) ALP ≥ 2XULN or (ii) abnormal ALP ≥ 1-2XULN and a bilirubin value from 20 to 100 *μ*mol/L. Recurrence free survival was compared between patients diagnosed with rPSC and those without rPSC.

*Results*. Seventy two patients were included. Fifty-eight (81%) were male. Mean age at transplant was 42 years (8 to 66 years). rPSC occurred in 18/71 (25%) patients. Mean time to recurrence was 77 months (9 to 172 months). rPSC rates were 9% and 28% at 5 and 10 years respectively. rPSC developed significantly earlier in patients with severe cholestasis at 3 months compared to all other patients without cholestasis (mean 81 ± 27 versus 183 ± 11 months Log Rank *P* = 0.008). Development of mild cholestasis was associated with earlier rPSC than those without cholestasis at 9 months (mean 63 ± 14 versus 179 ± 12 Log Rank *P* = 0.027) and at 12 months (mean 102 ± 16 versus 194 ± 12 Log Rank *P* = 0.001). Overall, the hazard ratio for rPSC was 4.8 (95% CI 1.3–17.0, *P* = 0.02) in patients with severe cholestasis at 3 months. Hazard ratios for mild cholestasis at 9 and 12 months was 4.9 (95% CI 1.0–22.9, *P* = 0.05) and 4.8 (95% CI 1.8–12.8, *P* = 0.002) respectively.

*Conclusions*. Our preliminary results indicate post-transplant cholestasis within the first 12 months following liver transplant is associated with rPSC. Our results mimic observations of other infectious disease recurrence following liver transplantation.

*Funding Agencies: *None

# Sub article: A24: A Multicenter, Double-Blind, Placebo-Controlled Ph3 Study of Ustekinumab, a Human Monoclonal Antibody to IL-12/23P40, In Patients with Moderately-Severely Active Crohn's Disease Who Are Naïve or Not Refractory to Anti-TNF**Α**: Uniti-2

**Authors:** B. Feagan, C. Gasink, Y. Lang, J. Friedman, J. Johanns, L. Gao, B. Sands, S. Hanauer, P. Rutgeerts, S. Targan, S. Ghosh, W. de Villiers, J. Colombel, Z. Tulassay, U. Seidler, W. Sandborn

**Affiliations:** ^24.1^Robarts Clinical Trials IncLondonONCanada; ^24.2^Janssen R & DLLCSpring HousePAUSA; ^24.3^Mt Sinai Med CtrNew YorkNYUSA; ^24.4^Northwestern UChicagoILUSA; ^24.5^U Hosp GasthuisbergLeuvenBelgium; ^24.6^Cedars*-*Sinai Med CtrLACAUSA; ^24.7^U of CalgaryCalgaryABCanada; ^24.8^U of Cape TownCape TownSouth Africa; ^24.9^Semmelweis UBudapestHungary; ^24.10^Hannover Med SchoolHannoverGermany; ^24.11^UCSDLa JollaCAUSA

*Background*. In the Ph 2b CERTIFI study, a single IV UST induction dose was effective & safe in CD pts previously failing anti-TNFs (Sandborn W. J. et al., N Engl J Med 2012; 367:1519–1528), but efficacy in pts only failing conventional therapy is unknown.

*Aims*. We evaluated 2 IV UST induction dose-regimens in a CD population not refractory to anti-TNFs.

*Methods*. Pts with moderate-severely active CD (CDAI220–450) who failed conventional therapy but were not refractory to anti-TNFs were randomized to a single dose of IV PBO, UST 130 mg, or weight-based tiered UST dosing ~6 mg/kg. Primary endpoint was clinical response at Wk 6 (reduction in CDAI score of >100 pts). At Wk 8, pts transitioned to IM-UNITI maintenance study or had safety follow-up through Wk 20.

*Results*. Of 628 pts randomized, median disease duration was 6.4 yrs; baseline (BL) mean CDAI was 303; 39%  & 35% were receiving steroids & immunomodulators, respectively at BL; 69% were naïve to anti-TNFs. At Wk 6, 55.5% &  51.7% in ~6 mg/kg & 130 mg UST grps were in clinical response versus 28.7% PBO (*p* ≤ 0.001). At Wk8, 40.2%  & 30.6% of pts in ~6 mg/kg & 130 mg UST grps were in clinical remission versus 19.6% PBO (*p* ≤ 0.009). Both UST doses showed significant improvements versus PBO in CDAI, IBDQ, CRP, & fLac & fCal. Proportions of AEs, SAEs, & infections (incl serious infections) were similar in UST & PBO grps. No malignancies, deaths, opportunistic infections or TB occurred in UST-treated pts.

*Conclusions*. IV UST induced clinical response & remission in pts with moderate-severe CD not previously failing anti-TNFs & was well-tolerated through induction.

*Funding Agencies: *Janssen Research and Development, LLC

# Sub article: A25: Small Intestinal Bacteria Determine Gluten Metabolism And Immunogenicity

**Authors:** A. Caminero Fernandez, H . Galipeau, J. McCarville, C. Johnston, S. Bernier, A. Russell, J. Jury, J. Casqueiro Blanco, J. Tye-Din, M. Surette, N. Magarvey, E. Verdu

**Affiliations:** ^25.1^McMaster UniversityHamiltonONCanada; ^25.2^The Walter and Eliza Hall Institute of Medical ResearchPARKVILLEVICAustralia; ^25.3^Universidad de LeonLEONSpain

*Background*. About 30% of the population is genetically susceptible to develop celiac disease (CD), but only 4-5% of these will develop intestinal atrophy upon ingestion of gluten. Additional environmental factors, related to alterations in gut microbiota, have been suggested to modulate CD risk. The underlying mechanisms are unknown.

*Aims*. Our aim was to investigate whether human small intestinal bacteria participate in gluten metabolism and CD pathogenesis using gnotobiotic mouse models.

*Methods*. Germ free C57BL/6 mice (*n* = 13/group) were di-colonized with* Lactobacillus rhamnosus* and* L*.* fermentum (Lactobacillus *spp) from duodenal aspirates of non-celiac subjects and mono-colonized with* Pseudomonas aeruginosa X46*.*1 (Psa)*, or di-colonized with* Staphylococcus warneri X18*.*3* and* S*.* epidermidis X18*.*1 (Staphylococcus *spp) isolated all of them from duodenal aspirates of celiac subjects. Mice were also di-colonized with* Lactobacillus spp* and* Psa*. Germ-free and altered Schaedler flora (ASF)-colonized mice were used as controls. One week after colonization, 8 out 13 mice received a gliadin gavage (7 mg/mouse). Small intestinal content was collected after 2 h to measure gluten amount by ELISA kit G12 and hydrolytic activities by incubation of gliadin with intestinal washes. Gluten peptide (33-mer) hydrolysis by bacteria was analyzed after incubations using a LC/MS/MS* in vitro*. After sequencing of peptides produced, immunogenicity was tested using peripheral blood mononuclear cells (PBMCs) induced* in vivo* in CD patients after oral gluten challenge.

*Results*. ASF colonization decreased gluten content (<2,500 ng/mL) in the small intestine compared to germ-free mice, that exhibited a range of values reaching a maximum of 12,000 ng/mL. Mono-colonization with* Psa* also decreased gluten content. Notably,* Psa* hydrolysis of 33-mer gluten peptide led to the generation of multiple peptides with retained immunogenicity, while* Lactobacillus* spp hydrolyzed* Psa*-modified gluten peptides, reducing their immunogenicity. An elastase-like protease from* Psa *was identified as responsible for the production of immunogenic peptides.

*Conclusions*. Gluten hydrolysis in the SI results from a combination of digestive and bacterial protease activity. Pathobionts and commensals determine end products of gluten hydrolysis and their antigenicity.

*Funding Agencies: *CAG, CIHR

# Sub article: A26: Characterization of Hepatitis B Virus (HBV) Lymphotropism and Immune Status in Chronic Hepatitis B (CHB) Pregnant Carriers

**Authors:** D. Wong, S. Gao, S. Joshi, T. Matwiy, G. Samadi Kochaksaraei, G. Bindra, G. van Marle, S. Martin, E. Castillo, C. Coffin

**Affiliations:** ^26.1^University of CalgaryCalgaryABCanada

*Background*. Our previous studies have shown that mild CHB disease flares are common in pregnancy in association with diverse viral quasispecies in plasma, including minor variants at positions associated with immune escape. HBV can infect lymphoid cells (i.e., peripheral blood mononuclear cells, PBMC), and the risk of HBV vertical transmission in untreated highly viremic mothers despite immunoprophylaxis has been linked to the transplacental passage of HBV-infected PBMC.

*Aims*. In pregnant CHB carriers, to characterize HBV genome and replicative intermediates in PBMC, along with assessment of cytokine/chemokine changes during pregnancy.

*Methods*. In 32 CHB pregnant patients analyzed to date (median age 30.5 y, 55% Asian [16/29], 34% African [10/29], 11% Caucasian [3/29], 3 unspecified; 10% genotype A [2/21], 43% B [9/21], 19% C [4/21], 14% D [3/21], 14% E [3/21], 12 unspecified; 22% HBeAg pos [7/32]), median HBV DNA at baseline versus post-partum in HBeAg pos (including 8/32 who received NA treatment) = 1.7log⁡(8) versus 1.6log⁡(3) IU/mL, and in HBeAg neg = 4.7log⁡(2) versus 6.2log⁡(2) IU/mL, median qHBsAg concentration in pregnant versus postpartum HBeAg pos = 4.9log⁡(5) versus 1.2log⁡(5) IU/mL and HBeAg neg = 1.7log⁡(3) versus 3.5log⁡(3) IU/mL, median ALT pregnant versus post-partum = 19 versus 25.5 U/L (range = 6–111 U/L). Total DNA or RNA was isolated from DNase/Trypsin treated PBMC from 15/32, and HBV DNA and HBV messenger (m)RNA was detected using HBV core gene specific primers by direct/nested PCR and/or reverse transcriptase (RT)-PCR. 32 patients (including 5 matched pregnant/post-partum) were tested for serum levels of cytokines/chemokines (i.e., CXCL-10, IL-12, MIP-1*β*, IFN *γ*, & CCL2 pg/mL) in parallel with healthy pregnant controls by ELISA (R&D Systems).

*Results*. Compared to healthy controls (our data and published literature), all CHB cases tested in pregnancy showed increase in 4/5 serum cytokines measured except in CXCL10. A significant difference in mean IL-12 levels in pregnancy versus post-partum (46.82 ± 9.86 versus 105.4 ± 18.37 pg/mL, *P* < 0.05) was noted along with 2-fold increase in ALT from baseline. Additionally, HBV genomes, including mRNA was detectable in 74% (14/19) of the tested PBMC samples.

*Conclusions*. In CHB pregnant carriers, HBV genomes including replicative indicative mRNA can be detected in circulating immune cells (i.e., PBMC). Compared to healthy pregnant controls, 4/5 cytokines tested were elevated and IL-12 levels significantly increased post-partum in association with ALT flares. Further analysis of maternal TH1/TH2 cytokine profile and assessment of HBV variants in PBMC, along with infant follow-up is warranted.

*Funding Agencies: *CIHR

# Sub article: A27: End-of-Life Healthcare Costs and Utilization among Patients with End-Stage-Liver-Disease in Ontario: A Population-Based Study

**Authors:** E. Kelly, S. Murthy, F. Wong, T. Shaw-Stiffel, L. Scully, M. Chalifoux, P. Tanuseputro, P. James

**Affiliations:** ^27.1^Department of MedicineThe Ottawa HospitalOttawaONCanada; ^27.2^9N/983 Toronto General HospitalTorontoONCanada; ^27.3^OHRIOttawaONCanada

*Background*. Healthcare for patients with end stage liver disease (ESLD) is often initiated in response to acute deterioration from disease progression. Despite guarded prognoses, many ESLD patients continue to receive expensive therapeutic interventions at the end-of-life (EOL).

*Aims*. The aims of this study were to evaluate EOL costs and utilization in patients with ESLD as compared to non-ESLD patients within the province of Ontario.

*Methods*. Using the Ontario Health Administrative Database, we performed population-based retrospective cohort study was conducted of all decedents within the province of Ontario, between April 1, 2010 and March 31, 2013. Patients with ESLD were defined using international classification of disease codes (ICD-9) for cirrhosis and decompensation (variceal bleeding, encephalopathy, ascites, hepatorenal syndrome and peritonitis). Patients with ESLD were compared to non-ESLD patients on direct health care costs in the last year of life, including hospitalizations, outpatient services and long-term care. Multivariate modelling was performed to compare the total costs in the final 90 days of life, adjusting for individual demographic (age and sex), co-morbidity burden.

*Results*. The study cohort consisted of 264,754 decedents, of which ESLD patients comprised 2.1% (*n* = 5,575). Direct health care expenditure for patients with ESLD increased more in the last 90 days of life compared to non-ESLD patients. The mean cost in the last 90 days of life was $35,008 for patients with ESLD and $21401 for patients without ESLD (*p* < 0.01), and this was predominantly related to increased acute care utilization (ESLD = $30,667 (88% of total costs) versus non-ESLD = $15,162 (71%)). ESLD patients had higher rates of acute care utilization in the last and 90 days of life (19 days versus 6 days, *p* < 0.01), longer hospitalization stays (additional 4.5 days (95% CI 4.2–4.9 days (*p* < 0.001)), and increased odds of dying in an institutional (OR 1.9 (95% CI 1.8–2.0; *p* < 0.001)). ESLD patients with peritonitis or hepatorenal syndrome incurred the highest mean total costs in the last 90 days of life ($43,196 and $38,584, resp.).

*Conclusions*. EOL care in ESLD patients is associated with substantially higher costs than in the general population, predominantly from increased acute care utilization including hospitalization. Whether optimizing outpatient services for patients and/or offering timely palliative services for patients with ESLD can lead to more cost-effective EOL care warrants further evaluation.

*Funding Agencies: *None

# Sub article: A28: Protective Immunity upon HCV Reinfection Is Associated with Selection of Memory CD8 T Cell Clonotypes with the Highest Functional Avidity

**Authors:** M. Boisvert, M. Abdel-Hakeem, J. Bruneau, H. Soudeyns, N. Shoukry

**Affiliations:** ^28.1^Centre de recherche du CHUMMontrealQCCanada; ^28.2^Centre hospitalier de l'Université de MontréalMontrealQCCanada; ^28.3^Département de microbiologieinfectiologie et immunologieUniversité de MontréalMontréalQCCanada.MontrealQCCanada

*Background*. While 25% of individuals with acute HCV infection can clear the infection spontaneously, long-term protection is elusive and reinfections remain common among people who inject drugs (PWID). Importantly, some individuals fail to clear subsequent infections despite a pre-existing HCV-specific memory immune response. We have previously shown that protection from viral persistence upon HCV reinfection correlates with expansion of HCV specific T cells with increased breadth. Viral persistence was associated with limited expansion of virus specific T cells.

*Aims*. We hypothesized that protective immune memory response is associated with selection of CD8 T cell clonotypes with the highest functional avidity and a polyfunctional profile.

*Methods*. We FACS sorted HCV-specific CD8 T cells to perform longitudinal T cell receptor (TCR) repertoire analysis as well as to generate CD8 T cell clones from patients that spontaneously resolved (SR) both infections (SR/SR) or that became chronically infected (CI) during reinfection (SR/CI).

*Results*. Our results showed that, upon reinfection, HCV specific CD8 T cells are recruited from the memory pool. The T cell repertoire was narrower (fewer number of dominant clonotypes) prior to reinfection in SR/SR group, compared to SR/CI group and became even more focused upon reinfection in SR/SR group. Individual HCV-1073 specific CD8 T cell clones generated from one SR/SR and one SR/CI individual exhibited comparable TCR avidity irrespective of the infection outcome. Clones established from SR/SR patient had a higher functional avidity and polyfunctionality index than clones established from SR/CI patient.

*Conclusions*. In conclusion, our results suggest that protective immune response upon HCV reinfection was associated with focusing of the HCV-specific CD8 T cell repertoire recruited from the memory pool whereby clonotypes with the highest functional avidity and a polyfunctional profile were selected.

*Funding Agencies: *CIHR, FRQS, ALF

# Sub article: A29: Developing a Prognostic Model for Significant Liver Fibrosis in Hiv-Hepatitis C (HCV) Co-Infected Individuals from the Canadian Co-Infection Cohort Study

**Authors:** N. Moqueet, C. Kanagaratham, D. Radzioch, S. Saeed, R. Platt, M. Klein

**Affiliations:** ^29.1^EpidemiologyBiostatisticsand Occupational HealthMcGill UniversityMontrealQCCanada; ^29.2^Department of Medicine and Department of Human GeneticsMcGill UniversityMontrealQCCanada; ^29.3^EpidemiologyBiostatisticsand Occupational HealthMcGill UniversityMONTREALQCCanada; ^29.4^Division of Infectious Diseases and Chronic Viral Illness ServiceMcGill University Health CentreMontrealQCCanada

*Background*. Liver fibrosis, which can lead to fatal liver failure, advances faster in HIV-Hepatitis C (HCV) co-infection due to higher inflammation. Immune and genetic markers could provide a non-invasive prognostic tool to target HCV therapy to those most at risk.

*Aims*. Does measuring pro-fibrogenic markers allow better prediction of significant liver fibrosis than clinical risk factors alone?

*Methods*. A prospective case-cohort study was nested in the Canadian Co-infection Cohort (*n* = 1119). From the eligible population (*n* = 679), a random subcohort (*n* = 171) and all cases (AST-to-platelet ratio index (APRI) ≥ 1.5) were drawn. Pro-fibrotic markers (IL8, MIP1*α*  &  *β*, MCP1, TNF*α*, RANTES, sICAM1, sVCAM1, CXCL9, CXCL11, TGF*β*1, hsCRP, sCD14) were measured from first available visit in the subcohort and cases. Genetic marker at Interferon Lambda (IFNL) rs8099917 was available for majority of the individuals in the study sample. We used Cox proportional hazards with Barlow weights for analysis. Discrimination and calibration were compared between Model 1 (clinical factors only) and Model 2 (Model 1 plus selected markers) for predicting 3-year risk of liver fibrosis. Discrimination was estimated with weighted Harrell's C index; calibration with the Hosmer-Lemeshow statistic and Gronnesby-Borgan test. Models were internally validated with bootstrapping.

*Results*. 113 individuals developed significant liver fibrosis over 1300 years of risk for an event rate of 8.63 per 100 person-years (95% CI: 7.08, 10.60 per 100 py). Model 1 included gender, current alcohol use, HIV viral load, baseline APRI, HCV genotype, and age as a restricted cubic spline with 3 knots. Model 1 was nested in Model 2, which also included IFNL rs8099917 genotype and 5 immune markers: IL-8, sICAM-1, RANTES, hsCRP, and sCD14. The C indexes for model 1 versus model 2 were 0.720 (95% CI 0.649, 0.791) and 0.756 (95% CI 0.688, 0.825) respectively. Both models were well-calibrated.

*Conclusions*. Including markers at IFNL rs8099917, IL-8, sICAM-1, RANTES, hs-CRP, and sCD14 enabled better prediction of the 3-year risk of significant liver fibrosis over clinical risk factors alone. While the improvement in discrimination was small, the model with the immune markers fit better. To assess whether this improvement justifies the additional cost of measuring these markers in the face of highly expensive HCV treatment requires further cost-benefit analyses.

*Funding Agencies: *CIHR, Canadian Network on Hepatitis C (CanHepC, formerly NCRTP-Hep C)

# Sub article: A30: Ornithine Phenylacetate Attenuates Loss of Muscle Mass and Improves Hepatic Encephalopathy in Bile-Duct Ligated Rats

**Authors:** C. Bosoi, M. Oliveira, M. Clément, M. Tremblay, G. Ten Have, N. Deutz, C. Rose

**Affiliations:** ^30.1^IRCMMontréalQCCanada; ^30.2^CRCHUMMontréalQCCanada; ^30.3^Texas A&M UniversityCollege StationTXUSA

*Background*. Chronic liver disease (CLD) induces numerous complications including muscle mass loss and hepatic encephalopathy (HE) which negatively impact the clinical outcome. Furthermore, muscle mass wasting and HE have been shown to lead to poor prognosis following liver transplantation. Hyperammonemia is considered the central component in the pathogenesis of HE, however recent studies have suggested ammonia to be toxic to other organs besides the brain, such as the muscle.

*Aims*. The aim of this study was to investigate the effect of ammonia on muscle mass in rats treated with an oral formulation of ornithine phenylacetate (OP; OCR-002).

*Methods*. Bile-duct ligated (BDL) rats were divided into 4 experimental groups; (1) Sham; (2) BDL; (3) Sham + OP; (4) BDL + OP. OP was administered orally by gavage (1 g/kg) daily for 5 weeks starting 1 week after surgery. Two days before sacrifice, locomotor activity (day/night) was assessed in infrared beam cages for 24 h. The day of the sacrifice, body weight, fat and lean mass (EchoMRI) were measured, followed by i.p. injection of a stable isotopes tracers cocktail (Phe/Gly) in order to asses fractional synthesis of protein (FSR). At sacrifice, samples were collected to measure blood ammonia (commercial kit), cerebral edema (specific gravity method) and muscle FSR.

*Results*. At 6-weeks, BDL rats demonstrated a 4-fold increase in blood ammonia versus Sham-operated controls. This increase was reduced by 40% in OP-treated BDL rats. Body weight decreased in BDL rats compared to sham-operated controls (360.2 g ± 13.6 versus 476.8 g ± 10.4; *p* < 0.001) and significantly increased following OP-treatment (429.6 g ± 117.9; *p* < 0.001 versus BDL). This was due to a higher gain of lean mass in OP-treated BDL rats compared to BDL rats (303.1 g ± 10.7 in BDL + OP versus 264.4 g ± 10.5 in BDL, *p* < 0.01). This was accompanied by increased muscle FSR in OP-treated BDL rats. Fat mass remained unchanged between treated and untreated BDL groups. OP treatment also normalized brain water content in BDL rats. Locomotor activity in BDL rats was reduced compared with sham-operated controls but no significant change was found between BDL + OP and SHAM + OP.

*Conclusions*. This is the first study demonstrating the efficient ammonia-lowering effect of an oral formulation of OP. Moreover, OP long-term treatment is a safe, non-antibiotic alternative with protective effects on the development of cirrhosis complications such as HE and muscle mass loss in rats with CLD. Whether the effect of OP on muscle mass loss attenuation is a result of lowering blood ammonia or directly improves muscle metabolism remains to be established.

*Funding Agencies: *CIHR

# Sub article: A31: Protein-Calorie Malnutrition Is Prevalent amoung Cirrhotic Patients Awaiting Liver Transplant as Measured by Direct Estimates of Protein and Calorie Intake as Well as Both Subjective and Objective Tools

**Authors:** K. Marr, A. Shaheen, L. Lam, M. Stapleton, K. Burak, M. Raman

**Affiliations:** ^31.1^University of CalgaryCalgaryABCanada; ^31.2^Alberta Health ServicesCalgaryABCanada; ^31.3^Univ CalgaryCalgaryABCanada

*Background*. Malnutrition is an important predictor of morbidity and mortality among cirrhotic patients.

*Aims*. Our objectives were to assess protein-calorie malnutrition (PCM) in cirrhotic pre-liver transplant patients and to study the correlation between subjective global assessment (SGA) and other objective measures of malnutrition.

*Methods*. We recruited pre-liver transplant adult patients at our center between October 2012 and September 2015. Nutrition status was assessed via the SGA. PCM was assessed by comparing recommended to actual protein and calorie intake. SGA was correlated with body mass index (BMI), dry BMI, handgrip strength (HGS) by calibrated dynometer, and mid-arm circumference (MAC). We used non parametric statistical Methods in our analysis.

*Results*. Seventy patients were included in this study. The majority were males (*n* = 46, 66%) with a median age of 58 years (IQR: 50–61). Moderate to severe malnutrition was prevalent in our cohort (SGA-A: *n* = 15 (21.4%), SGA-B: *n* = 30 (42.9%) and SGA-C: *n* = 25 (35.7%). There was a significant difference in the recommended calories consumed between SGA groups (A 99% versus C 72%, *P* < 0.001). A similar trend was observed for the recommended protein consumed (A 85%, C 62%; *P* = 0.08). SGA correlated with BMI (*A* = 26.4, *C* = 22.4; *P* = 0.002), Dry BMI (*A* = 25.9, *C* = 20.4; *P* < 0.001), and MAC (*A* = 29.5 cm, *C* = 22.0 cm; *P* < 0.001). HGS was significant according to gender. There was a significant difference in male HGS between SGA (*A* = 81 versus *C* = 51 PSI, *P* < 0.001), while in females the HGS trended towards a difference (*A* = 36 versus *C* = 29 PSI, *P* = 0.07). HGS and MAC were strongly correlated (Spearman correlation 0.49, *P* < 0.001).

*Conclusions*. Cirrhotic patients have significant protein-calorie malnutrition. Multiple malnutrition tools including dry BMI, HGS and MAC were precisely able to assess malnutrition.

*Funding Agencies: *Abbott and Baxter

# Sub article: A32: MIR-142-3P and Vitamin D-Mediated Regulation of Autophagy: Linking Environmental Gene Interactions in IBD

**Authors:** F. Dang, A. Hsieh, I. Verpalen, R. Murchie, L. Greenfield, M. Abdullah, D. Philpott, N. Jones

**Affiliations:** ^32.1^The Hospital for Sick ChildrenTorontoONCanada; ^32.2^University of TorontoTorontoONCanada

Not published at author's request.

*Funding Agencies: *CAG, CCC, CIHR, NASPGHAN

# Sub article: A33: Sitagliptin for the Treatment of Non-Alcoholic Steatohepatitis in Patients with Type 2 Diabetes

**Authors:** N. Malhotra, T. Joy, C. McKenzie, M. Beaton

**Affiliations:** ^33.1^Western UniversityLondonONCanada; ^33.2^London Health Sciences CentreLondonONCanada

*Background*. The prevalence of non-alcoholic fatty liver disease (NAFLD) is increasing worldwide. This is likely due to the rising numbers of those with impaired insulin sensitivity, dyslipidemia and obesity. NAFLD is best characterized based on histologic changes with non-alcoholic steatohepatitis (NASH) showing the presence of hepatocyte damage, inflammation and possible fibrosis. Pharmacotherapy has been a growing area of interest to treat NAFLD, specifically through modifying underlying risk factors. In patients with type two diabetes mellitus (DM2), oral hypoglycemic agents such as sitagliptin have proven to be effective. As a highly selective dipeptidyl peptidase-4 (DPP-4) inhibitor, it has proven to decrease HbA1C levels while being weight neutral.

*Aims*. To determine improvement in liver disease with sitagliptin therapy among patients with DM2 and NASH.

*Methods*. A randomized double-blinded, placebo-controlled pilot study of sitagliptin therapy (100 mg/day) in patients with biopsy proven non-alcoholic fatty liver disease and type two diabetes mellitus. After baseline evaluation, repeat liver biopsy, anthropometric and biochemical measurements were performed 6 months following treatment. Primary outcome was improvement in liver histology, assessed using the non-alcoholic fatty liver disease activity score (NAS) and change in hepatic steatosis measurement using MRI Iterative Decomposition of water and fat with Echo Asymmetry and Least-squares estimation (IDEAL). Secondary outcomes included improvement in the individual components of the NAS and liver fibrosis.

*Results*. Twelve patients completed follow up. There was no significant reductionin NAS (0.20, *P* > 0.999) 95% CI (−1.62, 2.02) or MRI IDEAL (2.0, *P* = 0.639) 95% CI (−7.3, 11.2) in those treated with sitagliptin compared to placebo. There was a non-significant improvement in hepatocyte ballooning, but no improvement in lobular inflammation (0.60, *P* = 0.156) 95% CI (−0.13, 1.33), steatosis (0.00, *P* = 0.908) 95% CI (−1.08, 1.08) or fibrosis (0.40, *P* = 0.233) 95% CI (−0.98, 1.78).

*Conclusions*. Use of sitagliptin therapy in non-alcoholic fatty liver disease patients with DM2 did not lead to a significant improvement in liver histology or hepatic fat measurement on MRI. The small number of patients as well as the relatively short follow up duration of study may have an effect on potential clinical significance.

*Funding Agencies: *PSI—Physicians Services Inc. Foundation

# Sub article: A34: A Quality Assurance Audit of the Atlantic Liver Transplant Program

**Authors:** A. Dorreen, S. Gruchy, M. Laryea, C. Guimont, M. MacNeil, K. Peltekian

**Affiliations:** ^34.1^Dalhousie UniversityMontrealQCCanada; ^34.2^Queen Elizabeth II Health Sciences CentreHalifaxNSCanada

*Background*. Variable outcomes for liver transplantation have been reported since its introduction as a therapeutic option for patients with end-stage liver disease. In Atlantic Canada, the Multi-Organ Transplant Program in Halifax, Nova Scotia serves as the primary referral center for liver transplantation. Regular evaluation of our program remains a main process by which quality improvement is made. Here we present a 10-year audit of the Atlantic Liver Transplant Program (ALTP).

*Aims*. We conducted a quality assurance audit of the ALTP in order to characterize patients accessing transplant services and their outcomes post transplant. The information will be used to promote development and provide feedback to all health care professionals involved in our transplant program.

*Methods*. The ALTP database was accessed to identify all patients that were referred for transplantation between 2005 and 2015. Basic demographic information was extracted, including information on the number of patients referred and transplanted, gender, age, Model for End-Stage Liver Disease (MELD), and reason for transplantation. Basic descriptive statistics and qualitative analyses were performed. Approval from the research ethics board was sought.

*Results*. Since 2005, a total of 264 transplants were performed on 248 patients, of which 37.1% were female (*n* = 98) and 62.8% male (*n* = 166). During this time period, 42 patients who were on transplant waitlist were withdrawn, 26 of which were removed for disease stability (61.9%). The mean age at transplant was 52.2 ± 10.8 for females and 54.6 ± 9.8 for males. The mean MELD was 19.9 ± 6.8 and 19.7 ± 7.7 for males and females respectively. The main indication for transplant in women was primary biliary cirrhosis (PBC) (23.5%, *n* = 23) and HCV in men (20.0%, *n* = 33). 16 patients underwent repeat transplantation for varying indications, representing 6.1% of all transplants. The mean time spent on the waitlist was 225.3 ± 313.2 days. The overall survival rate post transplant was 71.4% (*n* = 177); 71 patients died post transplant with a mean survival of 537.1 ± 832.8 days.

*Conclusions*. These data highlight that the patient population being referred to the ATLP is variable. Primary indication for transplantation varies according to gender, with PBC and HCV related cirrhosis being the main indication in females and males respectively. Survival post transplant was 71.4% and mean wait list time was 225.3 ± 313.2 days. Further development is needed to identify factors that predict poor outcomes post transplant. The data presented here will be used for program development and education.

*Funding Agencies: *None

# Sub article: A35: Correlating Fit Results with Neoplastic Findings in the BC Colon Screening Program

**Authors:** N. Shahidi, L. Gentile, G. Lovedeep, J. Hamm, M. Colleen, R. Enns, J. Telford

**Affiliations:** ^35.1^St. Paul's HospitalDivision of GastroenterologyDepartment of MedicineUniversity of British ColumbiaVancouverBCCanada; ^35.2^British Columbia Cancer AgencyVancouverBCCanada

*Background*. Fecal immunochemical test (FIT) performance depends on the test cut-off chosen with the Canadian Partnership Against Cancer (CPAC) recommending FIT positive predictive value (PPV) for neoplasia be ≥50%. Currently, there is no data assessing FIT performance at different cut-offs in a large average risk Canadian population.

*Aims*. Evaluate FIT performance for detecting neoplasia.

*Methods*. Data was obtained from a prospectively collected central database maintained at the British Columbia Cancer Agency (BCCA) (Vancouver, Canada) with consecutive participants of the BC Colon Screening Program (CSP) included. A single quantitative FIT with a cut-off of ≥50 ng hemoglobin/mL buffer solution was used. Participants with a positive FIT were referred for colonoscopy (CSPY) and were classified by the highest risk pathology. High-risk polyps (HRPs) were defined as adenomas or sessile serrated adenomas/polyps (SSA/Ps) ≥10 mm, adenomas ≥20% villous, adenomas with high-grade dysplasia, SSA/Ps with dysplasia, and traditional serrated adenomas. Cancer, HRPs and multiple (≥3) polyps were considered high-risk while 1-2 tubular adenomas or SSA/Ps <10 mm were considered low risk. This study was approved by the BCCA Research Ethics Board.

*Results*. From Nov-2013 to Dec-2014 32,129 participants had a positive FIT (positivity rate: 13.6%). Of those 20,000 (62.2%) underwent CSPY within the CSP, with 1679 (5.2%) pending CSPY at time of analysis. Pathology results were available for 13,497 (67.5%). The PPV of CRC and HRPs increased with increasing FIT cut-off alongside an increase in CSPYs saved; however, this also resulted in an increase in significant lesions missed (Table 7 [Table tab8] [Table tab9] [Table tab10] [Table tab11] [Table tab12] [Table tab13] [Table tab14] [Table tab15] ).

*Conclusions*. This is the first Canadian study evaluating the PPV of different FIT cut-offs in a large screening population. As the FIT cut-off rises PPV for CRC and HRPs increases alongside CSPYs saved but at the cost of missed lesions. The current cut-off of 50 ng/mL meets CPAC recommendations.

*Funding Agencies: *None

# Sub article: A36: Endoscopists Based Performance Report Cards Improve Adenoma Detection Rates in Screening Colonoscopies in High Risk Patients

**Authors:** A. Liu, M. Sey, S. Asfaha, C. Vinden, L. Stitt, B. Yan

**Affiliations:** ^36.1^University of Western OntarioLondonONCanada; ^36.2^Columbia UniversityNew YorkNYUSA; ^36.3^London Health Sciences CentreLondonONCanada

*Aims*. High quality colonoscopy is a necessary component of an effective colon cancer screening program. The adenoma detection rate (ADR) is considered one of the most important determinants of quality. The aim of this study is to determine the impact of endoscopists based performance report cards on ADR in patients undergoing colonoscopy for positive fecal occult blood tests or family history of colon cancer.

*Methods*. As a quality improvement initiative starting in 2012, annual report cards were issued to each endoscopist comparing their performance to the overall group mean. The histology of all polyps retrieved was manually confirmed from pathology reports. High risk adenomas were defined as those >1 cm, villous component, or serrated adenoma. The ADR at baseline, one year (Year 1), and two years (Year 2) after report card implementation were compared. Secondary outcomes include polyp detection rate (PDR) and high risk ADR. Comparison between the three years was performed with a chi-squared test followed by pairwise comparisons using a Bonferroni correction. Effect modification by endoscopist specialty and baseline ADR were assessed using interaction terms. All endoscopists included fulfilled Cancer Care Ontario colonoscopist standards and performed >200 colonoscopies yearly.

*Results*. A total of 3,115 screening colonoscopies performed by 17 endoscopists at a single center were included. The overall ADR, PDR, and high risk ADR was 38.2% (95% CI, 36.5–39.9%), 48.2% (95% CI, 46.5–50.0%), and 14.3% (95% CI, 13.1–15.6%), respectively. The overall ADR for gastroenterologists was 43.0%, general surgeons 31.8%, and hepatologists 24.0% (*p* < 0.001). The ADR increased from 34.5% at baseline to 39.4% at Year 1 (*p* = 0.016), which was sustained in Year 2 with an ADR of 41.7% (*p* = 0.001). From baseline to Year 2, the PDR improved from 45.0% to 51.8% (*p* = 0.003) and high risk ADR from 11.7% to 17.2% (*p* < 0.001). There was no evidence of effect modification by specialty or baseline ADR although there was a trend towards greater benefit among those with low and mid-ranged baseline ADR.

*Conclusions*. Significant sustained improvements were seen in the ADR in high risk patients undergoing screening colonoscopy following the introduction of performance report cards. Physician performance reporting should be included in colonoscopy quality assurance and improvement initiatives.

*Funding Agencies: *None

# Sub article: A37: The Impact of Warmed Carbon Dioxide Insufflation during Colonoscopy on Polyp Detection: A Randomized Double-Blind Controlled Trial

**Authors:** J. Green, A. Patel, L. Hookey

**Affiliations:** ^37.1^Queen's UniversityKingstonONCanada; ^37.2^Queen's UniversityMississaugaONCanada

*Background*. Colonoscopy is used for detection of neoplastic polyps, but significant miss rates are reported. Methods to reduce spasm of the colon have been investigated to increase adenoma detection rates by allowing better inspection of colonic folds. Room temperature carbon dioxide (CO_2_) insufflation has been demonstrated to be as efficacious as water immersion for both decreasing patient discomfort and achieving similar adenoma detection rates. These studies, however, utilized un-warmed CO_2_, which can produce spasms when released from high-pressure storage tanks. Warmed water instillation has been shown to reduce colon spasm; therefore, administration of warmed CO_2_ during colonoscopy may improve polyp detection.

*Aims*. To determine whether colonoscopy using warmed CO_2_ insufflation achieves greater detection of polyps per patient compared to room air insufflation.

*Methods*. This was a prospective, single centre, double-blinded, randomized control trial using warm CO_2_ versus room air insufflation. Patients undergoing colonoscopy for screening and surveillance indications were included and randomized to receive either room temperature room air or warmed CO_2_ (37 degrees Celsius). The primary outcome was polyp detection rate. A pre-specified power calculation determined that 444 enrolled patients would allow for detection of 50% increase in polyp detection rate, with alpha 5% and beta 20%. Secondary outcomes included adenoma detection rates and advanced lesion detection rates.

*Results*. The study was stopped after 222 patients had been recruited, as an interim analysis determined that continuation would be futile. Data was available for 202 participants. The room air and warmed CO_2_ groups consisted of 106 and 96 participants, respectively. The groups were similar in age (*p* = 0.809), gender (*p* = 0.778), indication for examination (*p* = 0.164), and bowel preparation score (*p* = 0.404). Sixty-five percent of participants in the room air group had polyps (*n* = 69), compared with 59% of participants in the warmed CO_2_ group (*n* = 57) (*p* = 0.402). Adenomas were detected in 51 and 44 participants in the room air and warmed CO_2_ groups, respectively (*p* = 0.746). There was no difference between groups in number of adenomas detected (*p* = 0.224).

*Conclusions*. Warmed carbon dioxide insufflation did not improve polyp or adenoma detection rates when compared with room air insufflation. One potential reason is that CO_2_ does not exert a significant effect on colonic motility. Alternatively, there may have been a loss of temperature of the CO_2_ as it travelled from the insufflator to the tip of the endoscope, thereby reducing its potential effect. At this time, warmed CO_2_ cannot be recommended as a method for increasing polyp or adenoma detection rates.

*Funding Agencies: *None

# Sub article: A38: Psychosocial Factors as Predictors of Sexual Functioning in Inflammatory Bowel Disease

**Authors:** D. Tripp, H. Yurgan, M. Ropeleski, C. Nickel, A. Muere, S. Vanner, M. Beyak

**Affiliations:** ^38.1^Queen's UniversityKingtonONCanada

*Background*. Inflammatory Bowel Disease (IBD), consisting of Crohn's Disease (CD) and Ulcerative Colitis (UC), is a painful chronic gastrointestinal disease characterized by inflammation. The impact of IBD on sexual function is poorly understood. More specifically, differences in sexual function between CD and UC have not been examined.

*Aims*. The initial objective was to examine differences in sexual function between CD and UC. The second objective was to examine predictors of sexual dysfunction.

*Methods*. 302 patients participated with 94 excluded due to incomplete measures of sexual function. Patients, both male (*n* = 88) and female (*n* = 120), were recruited from tertiary care clinics at Hotel Dieu Hospital in Kingston, Ontario, and completed a questionnaire including demographic, pain catastrophizing, social support, disability, depressive symptoms, and sexual function measures. Sexual function was assessed with the Golombok-Rust Inventory of Sexual Satisfaction (GRISS). The GRISS has subscales of impotence and premature ejaculation in males, anorgasmia and vaginismus in females, nonsensuality, lack of communication, avoidance and dissatisfaction in both males and females. Higher scores indicate more sexual dysfunction. Differences in GRISS subscales between CD (*n* = 128) and UC (*n* = 80) were examined using *t*-tests with Bonferroni corrections. A hierarchical regression with a biospsychosocial framework was used to examine predictors of total sexual functioning. Biological and demographic (age, gender, disability) variables were included in the first step, followed by psychological variables (pain catastrophizing, depressive symptoms) in step 2 and social variables (social support) in step 3.

*Results*. The overall sample had low to moderate total sexual dysfunction (M = 4.87, SD = 2.59). CD patients (M = 3.94, SD = 2.42) scored significantly higher on avoidance than UC patients (M = 3.01, SD = 2.17), *t*(217) = −2.86, *p* < .01. CD and UC did not significantly differ on any other subscale of the GRISS. The regression was significant, *F*(6,95) = 7.97, *p* < .01. In step 2 of the regression, depressive symptoms (*β* = .34, *p* = .01), age (*β* = .29, *p* < .01) and gender (*β* = −.21, *p* = .03) were significant predictors of total sexual function. However, when perceived social support (*β* = −.407, *p* < .01) was added in step 3, depressive symptoms were no longer a predictor of sexual function. Therefore, older age, being female, and less social support predict greater sexual dysfunction in this IBD sample.

*Conclusions*. CD and UC did not differ in overall sexual function. However, CD patients may avoid sexual activity more than UC patients. Furthermore, a greater perception of social support may allow for better sexual functioning.

*Funding Agencies: *CCC

# Sub article: A39: International Multicentre Study Comparing Risk Scoring Systems for Patients Presenting with upper Gastrointestinal Bleeding: Findings of the Canadian Cohort

**Authors:** A. AlNasser, H. Gregor, M. Chu, B. Yan, J. Gregor, A. Rahman, M. Sey

**Affiliations:** ^39.1^London Health Sciences CentreLondonONCanada

*Background*. Upper gastrointestinal bleeding (UGIB) is common and causes significant morbidity and mortality. Epidemiological data regarding UGIB in Canada is scarce and the last cohort study examining this was conducted nearly 15 years ago.

*Aims*. The objective of this study is to provide an update on the epidemiology of UGIB in Canada based on the Canadian Cohort of an international multicentre study.

*Methods*. A prospective open cohort study was conducted at two tertiary care hospitals in London, Ontario over 12 months.

Patients admitted to hospital with a primary diagnosis of UGIB were invited to participate. Baseline demographics, presenting symptoms, comorbidities, medication usage, vital signs, and Clinical Rockall Score (CRS) were recorded at the time of admission. Patient outcomes were evaluated during their hospitalization, including blood transfusion requirement, rebleeding, length of stay, esophagogastroduodenoscopy (EGD) finding and therapy, need for embolization or surgery, and 30-day mortality.

*Results*. Ninety-nine patients were recruited during the study (mean (SD) age was 65.6 (17), 39% females). The most common presentation was melena (73%). Twenty-two percent had established coronary heart disease and 59% were on an antiplatelet agent or anti-coagulation. Hypotension was the presenting symptom in 8% of patients and 12% had an initial hemoglobin ≤60 g/L. The mean (SD) CRS was 2.9 (1.7) and 35% has a low risk score <3. EGD was performed within 24 hours in 67% of patients and the most common finding was esophagitis/gastritis/duodenitis (23%), normal (22%), peptic ulcer disease (20%), and varices (16%). Endoscopic therapy was performed in 26% of cases and the most common modality used was combination injection and cautery/hemostatic clip (7%) or esophageal variceal banding (11%). Compared to patients with CRS ≥3, patients with low risk CRS <3 had a trend toward fewer blood transfusions (mean of 1.9 versus 1.5 units), less rebleeding (10% versus 6%), fewer embolization or surgery (2% versus 1%), and a shorter length of stay (9.8 versus 6.8 days), although none reached statistical significance. A CRS ≥ 3 was strongly associated with 30 day mortality (6% versus 0%, *p* < 0.001).

*Conclusions*. This is an update on the epidemiology of UGIB in Canada. A CRS ≥ 3 is strongly associated with 30-day mortality.

*Funding Agencies: *None

# Sub article: A40: Colonoscopy Report Completeness Improves at St. Paul's Hospital following the Implementation of a Dictation Template

**Authors:** N. Harris, J. Yonge, C. Galorport, M. Suzuki, B. Bressler, E. Lam, J. Telford, R. Enns

**Affiliations:** ^40.1^St. Paul's HospitalVancouverBCCanada

*Background*. The completeness of a colonoscopy dictation report is a quality indicator for endoscopic practice. Several guidelines outlining the key elements of a colonoscopy report were used to develop a dictation template at St. Paul's Hospital in 2013.

*Aims*. To assess if colonoscopy report completeness at St. Paul's Hospital has improved with the implementation of a dictation template.

*Methods*. Literature review findings regarding current dictation guidelines, such as those released by the CAG and ASGE, were compared to dictation template recommendations in use at St. Paul's Hospital. The colonoscopy reports of five physicians were reviewed at two time points, before (2008) and after (2014) the introduction of the dictation template. 150 charts were reviewed per doctor for each time period. The presence of variables will be assessed and percent completion of reports in 2008 and 2014 were compared. Cecal visualization rate, polyp detection rate and Spearman correlation between the percent completion of reports and report length were calculated. The study was approved by the UBC Ethics Board.

*Results*. The overall completeness of reports increased from 64% to 85% with the implementation of the dictation report template. Most item completion rates remained stable or increased; however, inclusion of a clinical preamble/indication(s) for procedure and reporting of patient comfort both decreased. Reporting of patient comfort, co-morbid conditions and current medications remained low at both time points.

*Conclusions*. Dictation report completeness increased from 2008 to 2014 following the implementation of a colonoscopy dictation template. Most items not included in the St. Paul's Hospital dictation template were consistently under-reported and modifications will be made to further improve report completeness.

*Funding Agencies: *None

# Sub article: A41: Impact of Types of Questions Asked on Gastroenterology Econsultation Outcomes

**Authors:** S. Canning, N. Saloojee, A. Afkham, C. Liddy, E. Keely

**Affiliations:** ^41.1^University Of OttawaOttawaONCanada

*Background*. Wait times in Canada to see a gastroenterologist continue to exceed the recommended targets of 2 weeks to 2 months for most indications. eConsult services facilitate primary care providers (PCPs) ability to communicate directly with specialists for advice. It can also reduce the need for patients to wait for face-to-face consultations with specialists. Since 2010, the Champlain BASE (Building Access to Specialist Advise) eConsult service has permitted PCPs to submit patient specific clinical questions to specialists via a secure web service.

*Aims*. To describe the types of Gastroenterology questions asked through a unique eConsult service, and assess the impact on referrals for face-to-face consultations.

*Methods*. Gastroenterology cases submitted to the Champlain BASE eConsult service between April 2014 and January 2015 were categorized for Gastroenterology-content using a modification of the International Classification for Primary Care (ICPC-2) taxonomy. The type of question (e.g., diagnosis or management) was classified using a validated taxonomy. Other data included the time for specialist to complete the eConsult, the perceived value of the eConsult by the PCP and the need for a face-to-face referral following the eConsult.

*Results*. Of the 121 Gastroenterology eConsults, 33% were liver related, 23% were GI symptom related (abdominal pain, gastroesophageal reflux disease, diarrhea, and constipation), and 13% were related to specific luminal diseases (irritable bowel syndrome, coeliac disease and inflammatory bowel disease). Of the 40 eConsults related to hepatology, 47% were questions regarding abnormal liver function testing. This was also the most common area of questioning overall (16%). Overall 51% of eConsults were related to diagnosis, 30% to management, 9% to drug treatments and 7% to procedures. It took the specialist <15 minutes to complete the eConsult in 67% of cases. The service was perceived as highly beneficial to providers and patients in 97% of cases. In 47% of submitted cases, a traditional referral was originally contemplated by the PCP but was now avoided and 1% resulted in a new referral that was not originally contemplated by the PCP. In the 24% in whom a referral was still needed, the PCP indicated that a more effective face-to-face consultation would occur.

*Conclusions*. The eConsult service provided timely, highly regarded advice from gastroenterologists directly to PCPs and often eliminated the need for a face-to-face consultation. With limited resources and access to gastroenterologists across Canada, eConsults provide a means to assist PCPs. Unnecessary referrals are avoided, thus reducing wait times for more urgent referrals. We plan to use the types of questions asked to inform planning of future CPD events for PCPs.

*Funding Agencies: *CIHR, Ministry of Health and Long-term Care, The Ottawa Hospital Academic Medical Organization Innovation Fund, eHealth Ontario, The Ottawa Hospital Department of Medicine and Bruyere Research Institute

# Sub article: A42: Is the Canadian Version of the Global Rating Scale a Valid and Reliable Tool for Measuring Quality Process in Endoscopy Units?

**Authors:** S. Carpentier, N. Sharara, M. Sewitch, S. El Ouali, M. Martel, A. Barkun

**Affiliations:** ^42.1^McGill UniversityMontrealQCCanada

*Background*. Quality in endoscopy is critical to ensure improved patient outcomes in colorectal cancer screening. The Global Rating Scale (GRS) was developed in the UK to provide metrics for quality in endoscopy and, although never formally validated, has been associated with improved patient outcomes nationally.

*Aims*. To psychometrically test the adapted Canadian version of the Global Rating scale (GRS-C), in view of its deployment by the Canadian Association of Gastroenterology throughout the country to participating endoscopy units.

*Methods*. The GRS-C was assessed at 3 institutions by endoscopy unit physicians, endoscopy nurses, and administrative personnel. The psychometric properties evaluated included validity, reliability, and responsiveness to change. For face validity, a group comprising staff not familiar with the GRS was assembled. Each of the groups responded to the questions of the GRS-C that span 12 items. Content validity was assessed by comparing GRS-C questions to national quality indicators in endoscopy, while construct validity was determined by ssociating questions of the GRS-C with those of its original UK GRS counterpart. The GRS-C was completed at the 3 sites both at time 0 and 2 weeks later, with no intervening change to processes, as well as 6 months later after practice changes had been implemented as a result of patient satisfaction questionnaire responses, to respectively assess test-retest reliability and responsiveness to change. Descriptive and inferential data analyses were completed, including kappa values for agreement and paired assessments when comparing question responses.

*Results*. Face validity was demonstrated as the majority of participants were able to accurately identify the overarching theme each item was intended to measure. For content validity, 18 of 23 key quality indicators (78%, 95% CI: 56–93%) determined by an expert consensus group were addressed in the GRS-C. Statements not included related to educational programs and monitoring of competency. When comparing GRS-C and GRS-UK ratings for all 3 sites, concordance ranged from 75–100% across all three sites, while Kappa agreement levels on test-retest reliability ranged from 0.65 to 0.83. Following a series of process change initiatives, responsiveness to change in 6-month post-implementation scores were statistically higher (*P* < .001) in two endoscopy units.

*Conclusions*. The GRS-C appears to exhibit satisfactory psychometric properties that can be used as part of a national quality initiative aimed at improving processes in endoscopy units. Linking GRS scores to actual patient outcomes is required to ensure that GRS-C implementation helps to achieve improved patient care.

*Funding Agencies: *None

# Sub article: A43: Gastroenterologists Concerns and Expectations towards Personalised Medicine

**Authors:** S. Veilleux, M. Villeneuve, N. Lachapelle, R. Kohen, L. Vachon, B. White Guay, J. Rioux, A. Bitton

**Affiliations:** ^43.1^Université LavalQuebecQCCanada; ^43.2^McGill University & Montreal General HospitalMontrealQCCanada; ^43.3^iGenoMed ConsortiumMontrealQCCanada; ^43.4^Université de MontréalMontrealQCCanada; ^43.5^McGill UniversityMontrealQCCanada

*Background*. Canadian physicians generally recognise the benefits of personalised medicine (PM) and using tools that predict disease course or response of treatment to inform therapeutic decisions. The implementation of PM is inconsistent across specialities and across Canada. This irregularity results in a decrease in treatment efficiency, an increase in healthcare systems costs and a diminished return on investment for firms involved in the development of predictive tests.

*Aims*. The aim of this study is to identify factors that affect the decision to use a predictive test that would assist gastroenterologists in their clinical decision-making for the personalised treatment of Inflammatory Bowel Disease (IBD).

*Methods*. Data was collected through six focus groups across Canada. Meetings were held in Vancouver, Saskatoon, Toronto (2), Quebec and Hamilton. A total of 28 gastroenterologists contributed to the study. Participants were asked about their current tools for treatment decision-making, their perception of present predictive tests and their expectations towards characteristics and implementation conditions of a new predictive test concerning IBD. Meetings were transcribed and encoded using QDA Miner software: 61 codes were initially produced and regrouped into 8 categories and 21 concepts were analysed.

*Results*. Four major concerns were raised by physicians about predictive testing are issues of accessibility (availability and cost), delays in turnaround time for the reception of results, doubts on the reliability of the test, and lack of proper training in the field of pharmacogenomics. Accordingly, physicians state three expectations: upcoming predictive tests have to show clear reliability qualities, significant benefits for the patient, and being accessible in a timely manner. Finally, the fundamental condition to the implementation of such a test refers to gastroenterologists active involvement in both the clinical trials leading to the test' endorsement by health agencies and training conferences.

*Conclusions*. Personalised medicine is spreading rapidly and new tools are being developed continuously to get the best out of the available treatments for patients and other biologics to come. Therefore, physicians are opened to predictive testing to help them in their decision-making process. However, they want to be involved in the development, regulatory approbation as well as the diffusion of such technology.

*Funding Agencies: *CCC, CIHR, Genome Quebec, Genome Canada

# Sub article: A44: Developing an Assessment Tool for Non-Technical Skills in Endoscopy: A Qualitative Study

**Authors:** M. Scaffidi, B. Chana, F. Kassam, U. Farrukh, S. Brar, W. Lee, S. Scaffidi, A. Rasheed, G. Hui, C. Walsh, S. Grover

**Affiliations:** ^44.1^University of TorontoTorontoONCanada; ^44.2^Hospital for Sick Children and The Wilson CentreTorontoONCanada

*Background*. Non-technical skills (NTS) in endoscopy are regarded by gastroenterology-focused organizations such as CAG and ASGE as core competencies. In other procedural specialities, such as surgery and anaesthesiology, instruments have been developed to assess NTS. To date, no assessment tool for NTS in endoscopy has been developed.

*Aims*. To determine the necessary NTS relevant to endoscopy and develop an assessment tool for these skills.

*Methods*. To identify existing assessment tools of NTS in other disciplines, a comprehensive literature search was conducted. Six tools were identified as relevant to NTS assessment in endoscopy. Gastroenterologists and experienced endoscopic registered nurses (RNs) from an endoscopic unit at an academic hospital were invited to participate in 3 two-hour focus groups to discuss the role of NTS in endoscopy and to identify areas of assessment of NTS. The first focus group was with gastroenterologists, and the second was with RNs. The final focus group combined participants from the previous sessions to reach concordance on the salient themes and identify exemplars of behaviours in each theme. Each group was led by a single moderator with prior experience in conducting focus groups. All focus groups were recorded and transcribed. Transcripts from the first two sessions were analyzed via thematic network analysis using NVIVO software. These findings were presented during the final focus group.

*Results*. 40 staff (18 physicians; 22 RNs) participated in the focus groups. After initial coding of the transcripts, there were 60 and 45 individual themes derived from the gastroenterologist and RN focus groups, respectively; 29 themes overlapped between the two groups. With refinement of these themes, six dimensions of NTS were found to encapsulate those relevant to endoscopy: teamwork, communication, situational awareness, decision making, leadership and professionalism. Behaviours commensurate with these dimensions identified from the focus groups were adopted into a scoring tool that ranked performance of each domain on a Likert-type scale of 1 to 5, representing a performance of poor to excellent, respectively.

*Conclusions*. We developed an instrument for the assessment of NTS in endoscopy on the basis of themes and behaviours raised from three focus groups, and arranged in a framework similar to NTS assessment tools in other procedural disciplines. Further research is required to test the feasibility, validity and reliability of the tool in the endoscopic setting.

*Funding Agencies: *None

# Sub article: A45: The Role of Colonoscopy within 24 Hours of Presentation for Acute Lower Gastrointestinal Bleeding (ALGIB)—A Systematic Review

**Authors:** I. Roshan-Afshar, M. Seyed Sadr, L. Strate, M. Martel, A. Barkun

**Affiliations:** ^45.1^McGill UniversityMontrealQCCanada; ^45.2^University of WashingtonSeattleWAUSA

*Background*. ALGIB is a common and potentially lethal disorder that often requires hospitalization. The role and benefits of EARLY colonoscopy in the management of ALGIB remain controversial.

*Aims*. The present study is a meta-analysis of published studies assessing the role of early colonoscopy (within 24 hours of presentation) in the management of ALGIB.

*Methods*. Systematic searches were completed querying MEDLINE, EMBASE, CENTRAL and ISI Web of knowledge until March 2015 using the terms related to gastrointestinal hemorrhage and early (emergency, early) colonoscopy. We included randomized controlled trials (RCTs) and observational studies assessing the role of early colonoscopy in patients with ALGIB. The primary outcome was 30 day-rebleeding. Descriptive statistics were generated as were risk ratios and weighted mean differences to characterize the utility of early colonoscopy.

*Results*. Amongst 824 citations, 10 observational studies (1 abstracts) and 2 RCTs included an early colonoscopy population (total *n* = 9,807 (including *n* = 86 in the 2 RCT intervention early colonoscopy arms), mean age range 52–78 yrs, 48.8% females, 71.1% hemodynamic instability, 1.5 ± 0.3 units of PRBC in first 24 hrs). Of the two RCT control groups (colonoscopy after 24 hours, *n* = 86; mean age 61.5 yrs, 37.2% females, 68.0% hemodynamic instability, mean 1.5 ± 0.2 units of PRBC in first 24 hrs), one included randomization to angioembolization. Endoscopic findings classified as probable or definitive cause of bleeding amongst all early colonoscopies (excluding two studies that solely assessed diverticular bleeding) were diverticula (41.5%), ulcers (7.7%), angiodysplasia (5.1%), colitis (4.1%), cancer (2.1%), and other findings (24.1%). No cause was found in 15.4% of patients. 34.7% of patients had endoscopic therapy when data were available (injection 15.3%, ligation 12.0%, thermal 4.0% or combination therapy 3.3%); other approaches included surgery (10.6%) or radiological therapy (15.5%). The rebleeding rate after 24 hours was (28/171) 16.4%, and the overall rebleeding rate (54/285) 18.9%, while all cause of mortality was (5/256) 2.0%; one study reported a mean ICU stay of 1.8 days (50 patients). Amongst the 2 RCTs there were no significant attributable differences after 24-hour rebleeding rates compared to controls RR = 0.99 (0.55; 1.65) or mortality RR = 0.50 (0.09; 2.66).

*Conclusions*. Early colonoscopy with appropriate endoscopic hemostasis can be carried out in patients with ALGIB within the first 24 hours of presentation, with diverticula being the most common etiology. Observational data are disparate, but based on RCT evidence, early colonoscopy identifies more patients with a definitive cause of bleeding but does not result in decreased rebleeding or mortality.

*Funding Agencies: *None

# Sub article: A46: Wait Time for Colonoscopy Is Reduced by Email Communication

**Authors:** A. Liao, T. Jeyalingam, F. Saibil

**Affiliations:** ^46.1^University of TorontoTorontoONCanada; ^46.2^Sunnybrook Health Sciences CentreTorontoONCanada

*Background*. Timely access to specialist care is a challenge in Canada. The CAG recommends colonoscopy be completed within 2 months for patients with a positive FOBT, but only 55% of such patients receive colonoscopy within 2 months. Delays in time-sensitive diagnosis can negatively impact outcome. Traditionally, patients see their endoscopist prior to colonoscopy (*Visit 1*) for a medical assessment and explanation of the procedure. While some endoscopists forgo this visit and first meet patients immediately prior to the procedure, this practice may negatively impact outcomes by increasing the risk of medical errors and not allowing for full informed consent. We suggest that, for some patients requiring colonoscopy,* Visit 1* can be replaced by email communication to obtain a medical history and explain the procedure and prep.

*Aims*. The goals of this study are to evaluate: (1) the impact of email communication on wait-time intervals from referral to colonoscopy; and (2) patient satisfaction with this method, compared to a standard office visit prior to colonoscopy.

*Methods*. We retrospectively reviewed 109 new patients referred for colonoscopy to a single gastroenterology practice between January 2013 and June 2015. 13 patients for whom the wait time from referral to colonoscopy was greater than 180 days were excluded, as this was beyond the maximum offered wait time for this particular practice and was often related to patient-initiated delay. The remaining 96 patients were divided into two cohorts based on whether or not email communication was established prior to colonoscopy. For patients in the email cohort (*n* = 43), a standardized email history had been obtained, and both prep instructions and procedure risks were emailed. Age, gender, reason for referral (symptomatic/FOBT+, positive family history, or routine screening), number of patients in which* Visit 1 *was eliminated, time from initial referral to colonoscopy, and patient satisfaction (determined by a standardized Likert scale questionnaire) were calculated and compared between cohorts.

*Results*. The average age and proportion of females were 44.6 years and 69.8% in the email cohort, and 56.5 years and 71.7% in controls.* Visit 1* was eliminated in 67.4% of patients in the email cohort. Mean wait time from referral to colonoscopy was 26 days shorter in the email cohort as compared to controls (72.5 days versus 98.5 days, *p* = 0.004). This effect was most pronounced in symptomatic patients; wait time was 35 days shorter in email patients within this subgroup (60.3 days versus 94.8 days, *p* = 0.003). No significant difference was observed in patient satisfaction scores.

*Conclusions*. Use of email in colonoscopy scheduling decreases wait time by roughly one month without compromising patient satisfaction. Larger, prospective/randomized studies are required to confirm these findings.

*Funding Agencies: *None

# Sub article: A47: Are We Choosing Wisely? An Analysis of Institutional Adherence to Colonoscopy Surveillance Schedules

**Authors:** M. Sheridan, A. Dorreen, S. Gruchy, J. Jones, S. Williams

**Affiliations:** ^47.1^Dalhousie UniversityBedfordNSCanada; ^47.2^QEII Health Sciences CentreHalifaxNSCanada; ^47.3^Queen Elizabeth II Health Sciences CentreHalifaxNSCanada

*Background*. Colorectal Cancer (CRC) is the second most common malignancy in males and third most common in females. Colonoscopy remains the gold standard for screening, but is a limited resource, with local wait time in excess of 2 years. Shortening the interval between screening colonoscopies exposes patients to unnecessary testing and risk, and also further limits access to colonoscopy. The factors used to determine the screening interval include the patient's family and personal history of polyps, number, size and location of polyps, their complete removal, quality of bowel preparation, and pathology of polyps. All these factors except pathology findings are available at the time of colonoscopy. It is our hypothesis that the information needed to make a recommendation is available at the time of colonoscopy for the majority of patients. If this is proven correct it would provide a strong argument to make this a standard component of each colonoscopy report.

*Aims*. The aim is to determine whether local colonoscopists are adhering to Canadian colon cancer screening guidelines when making screening interval recommendations.

*Methods*. Using Clinical Outcomes Reporting Initiative software, all colonoscopies completed from January to December 2014 at the QEII Health Sciences Centre for CRC Screening for patients with average risk, family history and previous polyp/cancer were analyzed. Data on the findings and details of the procedure were recorded (number and size of polyps, location of polyps, completeness of polyp removal, quality of bowel preparation, cecal intubation rate, operator characteristics, and recommended screening interval).

*Results*. 175 colonoscopy reports were analyzed. A screening interval recommendation was made at the time of colonoscopy in 150 cases (86%). Of the remaining 25 cases in which a recommendation was not made, sufficient information was available at time of colonoscopy in 44% (*n* = 11). If no recommendation was made at time of colonoscopy, 28% (*n* = 7) made a follow-up recommendation within 6 months. The correct screening interval was recommended in 83% (*n* = 125) of cases, which varied between gastroenterologists (92%, *n* = 78) and surgeons (80%, *n* = 55).

*Conclusions*. Further education is required to ensure all colonoscopists are aware of and comfortable following the current screening guidelines. Making a follow up recommendation at the time of colonoscopy is important to ensure appropriate follow up. Including this as a required field in colonoscopy reporting may help ensure that appropriate and timely recommendations are being made.

*Funding Agencies: *None

# Sub article: A48: Does It Work in the Real World? Effectiveness Study of Bowel Preparation for Colonoscopy—A Comparison of the Real World and Several Randomized Controlled Trials

**Authors:** C. Wang, R. Yang, S. Vanner, L. Hookey

**Affiliations:** ^48.1^Queen's UniversityKingstonONCanada

*Background*. The importance of bowel preparation quality is well recognized for high quality colonoscopies. There have been many different bowel preparations protocols tested over the years in randomized controlled trials (RCT's). However, RCT's are completed in an ideal setting. In reality, factors such as compliance, education, and patient motivation can significantly affect the effectiveness of the interventions. There have been no effectiveness studies for bowel preparations which measure the real world impact of interventions.

*Aims*. In this study, we compared the quality of bowel preparations of real-world patients undergoing colonoscopies to those enrolled in several RCT's.

*Methods*. The type of bowel preparation, age, sex, average number of bowel movements per day, comorbidities, indication for colonoscopy, the Ottawa Bowel Preparation Scale (OBPS), and colonoscopy completion rate were collected from five prospective randomized controlled trials (RCT) aimed at assessing colon cleansing using various preparation regimens, and compared with data from two real world diary studies. A total of 1372 patients who underwent colonoscopy from these 7 studies were analysed. The different bowel preparation types included a polyethylene glycol preparation (PEG) or sodium picosulfate plus magnesium citrate preparation (P/MC) taken in a traditional or split-dose regimen.

*Results*. There were no significant differences in sex and average number of bowel movements per day between the RCT and real world patients. However, RCT patients tended to be younger than real world patients (*p* = 0.002), this is seen when a prospective study assessing specifically patients over 70 was excluded. 6.0% of RCT patients undergoing colonoscopy were diagnosed diabetes mellitus (DM) and 39.8% of real world patients were diagnosed with DM, COPD, and/or kidney disease. PEG traditional and split-dose preparations do not differ significantly in OBPS between RCT and real world patients (*p* = 0.6, 0.2 resp.). However, both the traditional and split-dose Pico-Salix preparations are associated with significantly better OBPS in RCT patients when compared to real world patients (*p* < 0.0001 for both). Pooled RCT results showed that split-dose P/MC regimen produced better preparation than traditional dose P/MC (*p* < 0.001); this was not seen in the real world population (*p* = 0.45).

*Conclusions*. We have shown that real world patients have higher OBPS than RCT patients. This suggests that the motivation and education around these regimens may play a very important role in their success.

*Funding Agencies: *None

# Sub article: A49: A Proposal for Optimizing Patient Selection for Urgent Open Access upper Endoscopy in a Large Central Access Model of Acute Gastroenterology Care

**Authors:** K. Rioux, M. Swain, P. Belletrutti, K. Novak, B. Kathol, G. Heather, R. Kabani

**Affiliations:** ^49.1^Division of GastroenterologyUniversity of CalgaryCalgaryABCanada; ^49.2^Alberta Health ServicesCalgaryABCanada

*Background*. Many centres in Canada have adopted a central access model and open access endoscopy to improve consistency and efficiency in providing appropriate and timely GI services. Despite guidelines for the appropriate use of upper endoscopy, it remains difficult to determine from referring history which patients require the most urgent attention for common indications such as dysphagia.

*Aims*. Our aim was to perform a quality assurance audit of the Urgent+ Endoscopy Pathway at GI Central Access and Triage, Foothills Medical Centre, Calgary.

*Methods*. All patients triaged to urgent consultation and same-day upper endoscopy from January 1 to July 31, 2015 were identified. Anonymized patient records were obtained to determine referral indication, patient characteristics, and endoscopic findings.

*Results*. 202 patients were seen during the seven-month study period. 112 (55%) patients were referred for dysphagia with severe or progressive symptoms, 42 (21%) for suspected upper GI bleeding, and 39 (19%) for abnormal imaging. The median wait time from date of referral to date of consultation and endoscopy was 4.4 weeks. Abnormalities were identified in 79 (39%) patients, including cancer in 10 (5%) patients; 61% of patients had completely normal upper endoscopies. Of 112 patients with dysphagia, 41 (37%) had abnormal findings, the most common of which was Schatzki ring in 15 patients (13%); 4 patients (3.6%) had gastro-esophageal malignancy. Of 42 patients with suspected upper GI bleeding, 6 (14%) had gastro-duodenal erosions, and 6 (14%) had esophagitis; only one patient had a high-risk peptic ulcer requiring endoscopic therapy. Of 39 patients referred for abnormal imaging, 6 patients (15%) had malignancy.

*Conclusions*. Despite triage criteria and GI physician review of these referrals, a significant percentage of patients triaged to our Urgent+ Endoscopy Pathway have normal endoscopies or findings irrelevant to the referring indication. In patients with dysphagia in particular, lack of clinical detail or inaccuracies in referring history make it difficult to discern which patients are most likely to have significant pathology. At time of direct access endoscopy booking, we propose that a nurse-led, check-list driven, patient-reported confirmation of onset, timeline, progression, and impact of dysphagia will allow better prediction of important endoscopic findings, including malignancy, to best utilize our most urgent portal of access to outpatient upper endoscopy.

*Funding Agencies: *Division of Gastroenterology, Department of Medicine, University of Calgary

# Sub article: A50: Weak Correlation between Left and Right Adenoma Detection: A Single Regional Endoscopic Colorectal Cancer Screening Centre Experience

**Authors:** P. James, M. Hejaji, M. Alhegagi, A. Rostom, C. Dube, S. Murthy, A. Chatterjee

**Affiliations:** ^50.1^The University of OttawaOttawaONCanada; ^50.2^University of OttawaOttawaONCanada; ^50.3^the ottawa hospitalOttawaONCanada; ^50.4^The Ottawa HospitalOttawaONCanada

*Background*. Although overall and right adenoma detection (RAD) has been widely emphasized in recent studies, left-sided adenoma detection (LAD) may also be an important tool for measuring colorectal cancer (CRC) screening quality.

*Aims*. To determine if LAD is is associated with RAD and evaluate factors that may be associated with LAD and RAD.

*Methods*. This is a retrospective cohort study of patients who underwent a screening colonoscopy between May 2009 and December 2011 with a noted family history of CRC or positive fecal occult blood test (FOBT). Data regarding patient demographics (age and sex), procedure details (indication, number and location of polyps identified, bowel preparation and completeness) and polyp histology were captured. The main outcomes examined indclude adenoma detection rate (ADR), LAD rate (LADR), RAD rate (RADR), mean number of adenomas detected per positive colonoscopy (MADPC), left-sided MADPC (LMADPC) and right-sided MADPC (RMADPC).

*Results*. 2,178 patients and 14 endoscopists were included in the analysis. The median patient age was 59 years and 42% were male. 35% of procedures were performed for family history of CRC and 65% were performed for abnormal FOBT. ADR was 24%, RADR 12%, LADR 14%, MADPC 1.75, RMADPC 0.81 and LMADPC 0.83. Endoscopist RADR was found to be moderately associated with LADR (Spearman rank correlation [S] = 0.60, *p* = 0.05; see Figure 6). Two endoscopists had RADRs above the median and LADRs below the median. One endoscopist had RADR below the median and LADR above the median. Three endoscopists had both RADRs and LADRs above the median and their ADRs were greater than 25% (ADR range 27 to 43%). LMADPC was poorly associated with RMADPC (S = −0.37, *p* = 0.26). In multivariate analyses, factors associated with RAD and LAD include patient age, patient sex, procedure indication and endoscopist experience. RAD, but not LAD, was associated with endoscopist colonoscopy volume.

*Conclusions*. LAD is poorly associated with RAD. LAD may offer another opportunity to improve quality by providing detailed feedback for endoscopists involved in colon cancer screening.

*Funding Agencies: *The University of Ottawa Department of Medicine

# Sub article: A51: Do Adjuvants Add to the Efficacy of Polyethylene Glycol-Based Bowel Preparations? A Meta-Analysis of Randomized Controlled Trials

**Authors:** S. Restellini, O. Kherad, M. Martel, C. Ménard, A. Barkun

**Affiliations:** ^51.1^University of GenevaGenevaSwitzerland; ^51.2^McGill UniversityMontrealQCCanada; ^51.3^Université de SherbrookeSherbrookeQCCanada

*Background*. Polyethylene glycol (PEG) bowel preparations are safe and effective but require the consumption of large volumes of fluid, with relative low associated adherence. Alternatively, some data suggest adjuvants may enhance bowel cleansing quality and patient acceptance.

*Aims*. We performed a meta-analysis to determine the efficacy, willingness-to-repeat, and procedural outcomes of adding any type of adjuvant to a PEG bowel preparation, given as split-dose and non-split, in high (>3 L) or low-volume (<2 L) regimens.

*Methods*. We performed systematic searches of MEDLINE, EMBASE, Scopus, CENTRAL and ISI Web of knowledge until March 2015 for published randomized trials that assessed any regimen of PEG with adjuvant versus PEG without adjuvant. We excluded studies that included pediatric, hospitalized, or IBD patients, or trials in which the control group also received an adjuvant. Adjuvants were categorised as osmotic laxatives, irritant laxatives, antifoam products or other (prokinetics). The primary outcome was efficacy of bowel cleansing. Secondary outcomes included patients' willingness to repeat the procedure, polyp and adenoma detection rates.

*Results*. Of 2813 citations, 31 trials (*n* = 3920) fulfilled the inclusion criteria. PEG low-dose preparations with an adjuvant were not inferior to PEG high-dose OR = 1.03; (0.79–1.34); 20 studies. PEG high-dose preparations plus an adjuvant resulted in a significantly greater proportion of patients with adequate preparations (OR = 1.96 (1.32–2.94), 9 studies). Adjuvant combined to PEG low-dose did not enhance bowel cleansing compared to PEG low-dose alone (OR = 0.68 (0.43–1.09), 2 trials) Results were similar when analyses were restricted to split-dose comparisons. To our knowledge, no study assessed split PEG low-dose versus split PEG low-dose with adjuvants. Willingness-to-repeat was significantly greater with the use of PEG low-dose with adjuvants compared to PEG high-dose preparations (OR = 3.70 (2.0–6.67); 12 studies), but was lower for PEG high-dose with adjuvants versus PEG high-dose (OR = 0.63 (0.42–0.94)). Results were similar for split-dose comparisons. No differences were noted in polyp or adenoma detection rates.

*Conclusions*. Efficacy of bowel cleansing for PEG low-dose with the addition of an adjuvant was not inferior to PEG high-dose, and yielded a higher proportion of patient willingness to repeat the preparation. PEG high-dose was more efficient with an adjuvant compared to same dosage without adjuvant but was less tolerated. No differences were noted in polyp or adenoma detection rate. Additional research is required to further characterize the impact of adjuvants in PEG low-volume, especially with split-dosing regimens.

*Funding Agencies: *None

# Sub article: A52: Split-Dose versus Same-Day Bowel Preparations for Colonoscopy: A Meta-Analysis

**Authors:** C. Ménard, A. Barkun, M. Martel, O. Kherad, S. Restellini

**Affiliations:** ^52.1^Université de SherbrookeSherbrookeQCCanada; ^52.2^McGill UniversityMontrealQCCanada; ^52.3^University of GenevaGenevaSwitzerland

*Background*. A variety of bowel preparation types and administration schedules are available. Contemporary regimens include polyethylene glycol (PEG), sodium phosphate (NaP), picosulfate (PICO) and oral sulfate solution (OSS).

*Aims*. To compare efficacy, willingness to repeat the preparation, polyps and adenoma detection rates and side effects of split-dose versus same-day preparations amongst all contemporary regimens and subgroups comparing PEG high-dose (≥3 L) and PEG low-dose (<3 L).

*Methods*. Systematic searches were completed querying MEDLINE, EMBASE, Scopus, CENTRAL and ISI Web of knowledge from January 1980 to September 2015. All fully published randomized controlled trials with colon preparation for colonoscopy were included. Populations including pediatric, sole inpatients or sole IBD patients were excluded. The primary outcome measure was the efficacy of colon cleansing (excellent or good). Secondary outcomes included willingness to repeat, polyp and adenoma detection rates and side effects. A meta-analysis was conducted with results reported as odd-ratios (OR) with 95% confidence intervals. Heterogeneity and publication bias were assessed and quantified

*Results*. From an initial 2580 citations, 11 trials fulfilled the inclusion criteria (*n* = 1820 ITT). Same-day administration does not provide a significant benefit in bowel cleansing efficacy in comparison to split-dose regimens, regardless of preparation type, volume or use of adjuvants (OR = 1.19 (0.81; 1.75)). When performing sensitivity analysis, the exclusion of Cesaro et al. revealed a significant benefit in the efficacy of split-dose administration (OR = 1.47 (1.13; 1.91)). Willingness to repeat does not differ between the 2 groups (RC = 0.87 (0.38; 2.01)). Split-dose administration causes significantly more cramping, abdominal pain or bloating (OR = 1.50 (1.10; 2.05)). Polyp or adenoma detection rates are not different between the 2 administration regimens. 5 studies were included (*n* = 982 ITT) in subgroup analysis of PEG split high-dose versus PEG same-day low-dose. There was no significant difference between the 2 regimens (OR = 1.07 (0.64; 1.78)). Only one study (125 patients) permitted the comparison of PEG split low-dose versus PEG same-day low-dose and showed no difference in efficacy (OR = 0.91 (0.34; 2.47)).

*Conclusions*. There is no significant difference in efficacy between same-day and split-dose administration of bowel preparations. However, there is great variability in type and administration schedule of adjuvants in available same-day preparations arms, which may have an effect on the generalizability of the present Results. Further studies should focus on more rigorous comparisons of the different administration schedules.

*Funding Agencies: *None

# Sub article: A53: Randomized Prospective Study: Impact of the Patient Education Website on the Quality of Outpatient Bowel Preparation for Colonoscopy: Intrim Data

**Authors:** M. Suzuki, M. Bardi, O. Takach, C. Galorport, J. Yonge, N. Harris, E. Lam, J. Telford, G. Rosenfeld, H. Ko, R. Enns

**Affiliations:** ^53.1^St Paul's HospitalVancouverBCCanada; ^53.2^UBCVancouverBCCanada

*Background*. Low level bowel cleanliness that occurs in 25% of patients, hinders polyp detection rates and limits colonoscopy effectiveness. Those with inadequate preparation have incomplete examinations, fewer polyps detected, more repeat colonoscopies and higher resource utilization. Aside from pharmacological and timing of purgative factors to optimize bowel preparation, non-pharmacological factors that influence patient compliance (i.e., patient education) in the preparation phase can significantly improve bowel preparation quality.

*Aims*. To assess if interactive, individualized web based instruction lead to improved colonoscopy preparation through enhanced patient compliance, satisfactin and tolerability of preparation.

*Methods*. A randomized, prospective, single blinded trial initiated at St. Paul's hospital in Vancouver, B.C. Inclusion criteria: age > 19, planned outpatient colonoscopy, and willingness/ability to participate by reading the online English material supplied by sending the subject a specific domain (which contains the educational platform of information for their colonoscopy). Exclusion criteria: None. Consecutive patients enrolled into the study (target of 450 participants). Data Collected: demographics, cancellations, bowel preparation cleanliness scores as per Boston bowel preparation quality (BBPS) and Ottawa bowel preparation score. Primary end points: percentage of patients that achieve an excellent BBPS following web-based instructions versus paper instructions. Assessment of patient satisfaction, preparation tolerability and patient activation score through post colonoscopy follow-up surveys.

*Results*. As of October 2015, 285 subjects have been recruited. 127 are male; mean age 57 years (range 20–81). 142 were assigned to Group A (paper based) and 143 to Group B (web based). A Fisher's exact test showed a significant difference in the proportion of subjects achieving an excellent BBPS score ≥8 (Group A = 57% (81/142), Group B = 71% (102/143) *p* = 0.0136). There was no significant difference in patient reported satisfaction (*p* = 0.1438), helpfulness (*p* = 0.1426) or clarity of instructions (*p* = 0.1183).

*Conclusions*. Interim analysis detected a significant difference in patients achieving excellent bowel preparation scores between interactive individualized web based instructions versus written instructions. This study will continue to recruit until a target sample size of 450 to determine if our other primary end points will achieve significance. We plan to complete recruitment by Dec 2015. If preliminary data is supported by final results; the use of this platform will be encouraged to maximize ideal patient preparations for colonoscopy.

*Funding Agencies: *None

# Sub article: A54: Colonscopy Quality Assurance and Maintanance of Cometency among Pediatric Gastroenterology Ftass Members—A Pilot Project

**Authors:** C. Barker, M. Alaifan

**Affiliations:** ^54.1^University of British ColubmiaVancouverBCCanada

*Background*. Colonoscopy quality indicators in addition to maintenance of competency skills are relatively well established in the adult literature, however it is much less so in pediatric gastroenterology. One of the suggested quality assurance measures which is relevant for both adult and pediatric patients would be cecal intubation rate, which it has been suggested should be ≥90% as per ASGE guidelines.

*Aims*. The purpose of this study was to evaluate the cecal and terminal ileal (TI) intubation rates at our tertiary care pediatric centre. The aim is evulate the centre quialty of colonoscopies compared to the adult standards.

*Methods*. A retrospective chart review study was performed on all pediatric patients (age 16 months–18 year old) who underwent colonoscopies at our single centre performed between January 2013 to July 2014 (18 months period). Patients scheduled for sigmoidoscopy were excluded. The endoscopy reports were reviewed to ascertain whether the cecum and TI were reached as well as quality of bowel prep and any other stated reasons for reasons of failure. Clinical charts were reviewed to obtain indication for colonoscopy.

*Results*. A total of 288 colonoscopies were performed by 5 gastroenterologists during the 18 month period. The number of colonoscopies per staff ranged from 36–70 procedures. The numbers of year in practice ranged from (3–25 years). The overall cecal intubation rate was 98.3% (range 97.1%–100%). TI intubation rate was lower at 84.4% (range 66.7%–90%). The main stated reason for inability to enter cecum/TI was technical difficulty and poor bowel prep. No complications were encountered in those procedures.

*Conclusions*. Despite relatively low volumes, cecal intubation rates are very good exceeding some suggested standards. TI intubation rates were lower and it was noted there was a higher degree of variability. Multi centre evaluation over a longer time period and collaboration should take place to establish relevant parameters for quality assurance in pediatric endoscopy.

*Funding Agencies: *None

# Sub article: A55: The Canadian Pediatric Survey of Access to Gastroenterology (PSAGE) 2015—Preliminary Report

**Authors:** J. Yap, P. Sathya, A. Noble, M. Boland, H. Brill, W. El-Matary

**Affiliations:** ^55.1^University of AlbertaEdmontonABCanada; ^55.2^Memorial UniversitySt JohnNFCanada; ^55.3^Dalhousie UniversityHalifaxNSCanada; ^55.4^University of OttawaOttawaONCanada; ^55.5^McMaster UniversityHamiltonONCanada; ^55.6^University of ManitobaWinnipegMBCanada

*Background*. The Pediatric Survey of Access to GastroEnterology (PSAGE) program was designed to provide cross-sectional data of current wait times related to non-urgent indications for digestive health care in children. Twenty-one non-urgent pediatric-specific gastrointestinal indications were developed through consensus by the PSAGE steering committee including proposed benchmark wait-times.

*Aims*. To described the physican demographics, practices and current wait times for Canadian children accessing non-urgent digestive health care.

*Methods*. Canadian pediatric and adult gastroenterologists known to care for children were approached to complete an online or hard copy survey between the 13th to the 24th of April 2015. The survey consisted of a one-time physician demographics questionnaire. Participating physicians were requested to record seven data points per patient for 5 consecutive new patient consults and 5 consecutive new endoscopic procedures. These included the number of days off school and the second character of the patient's postal code.

*Results*. Thirty one percent (*n* = 30) of physicians approached participated in the survey, entering data for 241 patients. Ninety six percent of surveyed physicians practiced within a teaching hospital; 83.3% were in full-time practice. Surprisingly, 20% of physicians were limiting new patient referrals. The majority of physicians (86.6%) were “not at all” to “somewhat satisfied” with current wait times. Adolescence (46.1%) was the most common patient age group, followed by the 6–10 year olds (25.7%). The patient referrals from family doctors were broadly distributed between provinces (25−72.7%) with the majority of referrals from urban centres. The most common indications for referral were celiac disease confirmation (43%), chronic abdominal pain (36%) and sub-acute rectal bleeding (21%). Less than 10% of patients had indicated greater than 5 days of missed school. All physicians indicated the presence of an anaesthetist during endoscopic procedures.

*Conclusions*. The new PSAGE survey has provided useful insight into the current demographics, practices and perceived wait times concern of pediatric gastroenterologists looking after children with gastrointestinal problems. More detailed analysis of wait times relative to the proposed benchmarks is required.

*Funding Agencies: *CAG

# Sub article: A56: The 5-Ht_**4**_ Receptor Agonist Prucalopride Induced a Variety of Human Colonic Pressure Waves Assessed by High Resolution Manometry

**Authors:** W. Chen, J. Chen, J. Huizinga

**Affiliations:** ^56.1^McMaster UniversityHamiltonONCanada

*Background*. The study of human colon motility is a challenge since the colon may be inactive for long periods of time, however, to understand motor dysfunction in chronic constipation, manometry is essential. It is therefore important to evaluate the use of various stimuli for assessment of colon motor function.

*Aims*. The aim of this study was to investigate the effect of oral prucalopride on human colon intraluminal pressure activity, within the constraints of a 6 hour colon function test.

*Methods*. We examined the colonic intraluminal pressure patterns in 23 subjects using high resolution colonic manometry (HRCM) with 36 solid-state sensors, 1 cm apart. 13 of the subjects had chronic constipation, 10 were either healthy or had non-constipation IBS. Two mg of prucalopride, a 5-HT_4_ agonist, was given orally to all subjects.

*Results*. Oral prucalopride is rapidly absorbed and maximum bioavailability is reached 1–3 hours after administration (Winter et al. JPGN 57(2013)197). Prucalopride elicited an excitatory effect in 18/23 subjects, 11/13 of the constipated patients and 7/10 of the non-constipated. The induced activities were simultaneous pressure waves, propagating pressure waves including High Amplitude Propagating Pressure Waves (HAPWs, also called HAPCs) and isolated pressure transients. An increase in occurrence was seen in 3/23 subjects for HAPWs, 13/23 subjects for isolated pressure transients and simultaneous pressure waves, and 2/23 for propagating pressure waves. An increase in amplitude was seen in 9/23 subjects for simultaneous pressure waves and 3/23 subjects for propagating pressure waves. A biphasic effect was observed where prucalopride elicited an initial excitation within 5 minutes of administration and/or a second phase of excitation after 10–60 minutes of administration. We propose that the first phase is elicited by a gastrocolonic reflex involving enterochromaffin cells in the stomach and vagal colonic excitation. We propose that the second phase is elicited by the active drug in systemic circulation acting on the enteric nervous system.

*Conclusions*. In conclusion, prucalopride has a prokinetic acute effect, likely through a 5-HT_4_ transduction-pathway. The colon function test shows the sensitivity of the patient to the activation of the 5-HT_4_ pathway to elicit simultaneous pressure waves and isolated pressure transients. To evaluate a potential defect or absence of the 5HT_4_ pathway, higher doses of prucalopride may have to be given.

*Funding Agencies: *National Natural Science Foundation of China

# Sub article: A57: Bacterial Presence on Flexible Endoscopes versus Time Since Disinfection

**Authors:** K. Mallette, P. Pieroni, D. Thomson, L. Struthers, S. Dhalla

**Affiliations:** ^57.1^University of ManitobaWinnipegMBCanada; ^57.2^Brandon Regional Health CentreBrandonMBCanada

*Background*. Flexible endoscopes are extremely valuable in the diagnosis and management of gastrointestinal disease. Recently, there have been documented cases of transmission of antibiotic resistant microbes in the United States via endoscopy. Previous guidelines suggested that endoscopes be reprocessed prior to use; however, a study conducted at our institution demonstrated that endoscopes could be stored up to 7 days prior to use when maintained in a ventilated, dust free cabinet.

*Aims*. The objective of this study was to validate the prior study conducted at our institution, correlating the length of time that an endoscope was hung in a cabinet and how much bacteria was cultured prior to use.

*Methods*. Prospectively, we cultured specimens from 19 gastroscopes, 24 colonoscopes and 5 side viewing duodenoscopes during the period of 2011 to 2015. Two scopes were evaluated weekly on a rotational basis, with a total of 327 evaluations. However, only 164 results had complete data denoting date of cleansing, number of days stored and culture Results. Endoscope hang time was calculated by counting the number of days between disinfection and microbiological evaluation.

*Results*. All positive culture results were within the acceptable range for potable water (less than 200 cfu/mL). The highest count was 80 cfu/mL, which was cultured from colonoscope CHD4 after a hang time of 1 day. The highest count for ERCP scopes was 10 cfu at both 2 and 7 days, and the highest count for gastroscopes was 50 cfu/mL after 1 day. The majority of cultures, irrespective of hang time, were negative for bacterial growth (Figure 7). There was no significant difference in the number of bacteria cultured after 1 day compared to 7 days when all scopes were combined. There was no statistical difference observed in bacterial cultures after 1 day compared to subsequent days for gastroscopes, colonoscopes, or ERCP scopes.

*Conclusions*. There does not appear to be a correlation between the length of hang time and the results of the bacterial culture. However, in this study, the sample sizes were small and thus a difference may not have been detected. This data further supports the previous study conducted at our hospital. Endoscopes do not need to be reprocessed prior to use if they are decontaminated according to the manufacturer's instructions after use, hung in a ventilated, dust-free cabinet prior to use, and reused within a period of 7 days.

*Funding Agencies: *None

# Sub article: A58: Carbon Dioxide versus Room Air Insufflation in Colonoscopy: A Retrospective Study

**Authors:** K. Mallette, D. Thomson, S. Dhalla

**Affiliations:** ^58.1^University of ManitobaWinnipegMBCanada; ^58.2^Brandon Regional Health CentreBrandonMBCanada

*Background*. An increased awareness for colon cancer screening has resulted in an increased demand for colonoscopy in North America. Carbon dioxide (CO_2_) or room air (RA) can be utilized for insufflation, however, RA is the default method employed by every manufacturer of endoscopes. The use of CO_2_ as a method of insufflation is being increasingly employed due to its ability for rapid absorption and respiratory expiration; which leads to less abdominal distention and hopefully less discomfort and pain.

*Aims*. The purpose of this study was to compare CO_2_ to RA insufflation during colonoscopy to assess for differences in sedation utilized, oxygen required, endoscopy time, recovery time and post-procedure pain.

*Methods*. A total of 445 consecutive patients who underwent only colonoscopy between November 28, 2014 and May 29, 2015 utilizing CO_2_ or RA at our regional referral site were selected. Patients were excluded due to previous large bowel resection, scheduled endoscopic mucosal resection, and incomplete colonoscopy. Patient demographic data included age, gender and BMI. Intra-procedure measurements tabulated included amount of sedation, O_2_ requirement and endoscopy time. Post-procedure, recovery time (defined as time from admission to the post-procedure area to the time of discharge) and presence of pain, were noted. Two sample unpaired *t*-tests were utilized to analyze the aforementioned data and pain was further evaluated for differences between genders, and at three 15 minute time intervals.

*Results*. Patient demographics, O_2_ requirement and endoscopy time were not statistically different at the 95% confidence level. The amount of midazolam utilized was significantly higher in the CO_2_ group (3.17 mg versus 3.67 mg, *p* < 0.001), while amount of fentanyl was significantly higher in the RA group (80.2 mcg versus 64.5 mcg, *p* < 0.001); largely due to physician preference. Patient recovery time was not different between the two groups (59 mins (RA) versus 61 mins (CO_2_), *p*-value 0.225) and was not statistically longer for more invasive procedures compared to biopsy (RA: *p*-value 0.571) (CO_2_: *p*-value 0.138). Patients in the CO_2_ group experienced less pain overall (15% versus 36%), at admission to recovery (14% versus 31%), and 15 minutes after admission to recovery (4% versus 13%). Both males and females saw decreased levels of pain overall (males: 17% versus 40%) (females: 13% versus 32%) and at admission to recovery (males: 16% versus 34%) (females: 12% versus 29%). Males also experienced less pain at 15 minutes of recovery (4% versus 17%) (Figure 8 [Fig fig9] [Fig fig10] [Fig fig11]).

*Conclusions*. Insufflation utilizing CO_2_ did not significantly alter O_2_ requirement, endoscopy time or recovery time. However, CO_2_ significantly reduced the percentage of patients experiencing post-colonoscopy pain and decreased the frequency of pain in both male and female patients.

*Funding Agencies: *None

# Sub article: A59: Endoscopy Utilization and Outcome for the GI Nurse Navigator Pathway: A Quality Improvement Project for Chronic Dyspepsia, Heartburn & Irritable Bowel Syndrome

**Authors:** K. Milne, B. Kathol, M. Swain, C. Johnstone, J. Kwan, W. Schoombee, C. Andrews, K. Novak

**Affiliations:** ^59.1^University of CalgaryCalgaryABCanada; ^59.2^Alberta Health ServicesCalgary ZoneCalgaryABCanada; ^59.3^Univ CalgaryCalgaryABCanada; ^59.4^Calgary Foothills Primary Care NetworkCalgaryABCanada

*Background*. The Gastrointestinal Nurse Navigator (NN) pathway is a collaborative strategy developed by the Division of Gastroenterology (GI) and the Calgary Foothills Primary Care Network (PCN), aimed to provide comprehensive care to patients through nurse-lead medical education as well as nutrition and behaviour health support for patients with non-urgent GI concerns. Since 2012, referrals for dyspepsia, gastroesophageal reflux disease (GERD) and irritable bowel syndrome (IBS) were selected, with nurse-lead telephone assessment, direct referral to endoscopy for red flags, and group multidisciplinary medical education session with GI consultation.

*Aims*. To evaluate endoscopy usage and diagnostic outcome in the NN pathway.

*Methods*. This is an ethics approved, single center, prospective observational study, including 443 patients from July 2012 to December 2014. Demographics, endoscopic indication and diagnostic outcome were evaluated.

*Results*. Of the 443 patients, 198 had dyspepsia, 211 GERD, and 34 had IBS. 251 (56%) Underwent endoscopy, with 7 patients (1.6%) having simultaneous referrals to other gastroenterologists and endoscopy performed privately outside of the pathway. Gastroscopy was the most commonly performed procedure (193/251, 77%), followed by colonoscopy (48/251, 19%) the remainder were sigmoidoscopy (10/251 4%). More females than males (48% versus 45%) underwent endoscopy, and the average age of patients who underwent endoscopy was higher at 48 versus 46 yrs (*p* > 0.05). Of those patients who underwent endoscopy, 15 studies (5.6%) revealed diagnoses changing medical management (*H. Pylori*, adenomas, inflammatory bowel disease (IBD) and Barrett's esophagus). Those most likely to have these diagnoses had an average age of 52. There were no cancers diagnosed and IBD was mild.

*Conclusions*. The NN pathway is safe, with low morbidity given minimal significant pathology identified with no malignancies. The identification of patients for entry into this pathway is appropriate and furthermore, many may not have required endoscopy at all. Future strategies should aim at conservative therapy, focused on lifestyle and medical management within primary care.

*Funding Agencies: *None

# Sub article: A60: Effect of Feedback of Conoloscopy Patient Comfort Scores on Endoscopist Behaviour

**Authors:** B. Chan, A. Hussey, N. Rubinger, L. Hookey

**Affiliations:** ^60.1^Queen's UniversityKingstonONCanada; ^60.2^Hotel Dieu HospitalKingstonONCanada

*Background*. There have been many studies evaluating the type, amount, and risks of sedation during colonoscopy, as well as patient comfort during the procedure. For many experts, the optimal colonoscopy is a complete exam with the least amount of sedation while maintaining patient comfort.

*Aims*. Using a Canadian, validated patient comfort scoring system, this study aimed to determine whether the introduction of a patient comfort score would affect the amount of sedation used by endoscopists.

*Methods*. The* Nurse Assisted Patient Comfort Score *(NAPCOMS) was used to assess patient comfort during colonoscopy and was added as routine procedural documentation. The study was conducted over two phases; this report focuses on Phase Two. Phase One consisted of endoscopist blinded and endoscopist aware NAPCOMS collection. In Phase Two, data was collected over a five month period and scores fed back to individual endoscopists on a monthly basis.

*Results*. We previously presented Phase One data, which showed no significant differences in sedative use or NAPCOMS.

In Phase Two, we documented 13 endoscopists and 932 cases over the course of five months. There were nine gastroenterologists (773 cases) and four general surgeons (159 cases). Analysis of group data between individual months showed significant differences in midazolam (*p* < 0.001) and fentanyl use (*p* = 0.035). The amount of fentanyl declined, while there was no trend in midazolam use. Total NAPCOMS did not show any significant differences, but subgroup analysis showed a decline in pain score (*p* = 0.037). When month one was compared to all other months in the study, there were no significant differences.

Data compared between gastroenterologists (GI) and general surgeons (GS) showed no significant differences in NAPCOMS. Gastroenterologists used significantly less midazolam (*p* = 0.001) but more fentanyl (*p* = 0.037). In addition, GI utilized more position changes (*p* = 0.002) but there was no difference in procedure duration or use of abdominal pressure.

*Conclusions*. Phase One showed no significant differences in the primary endpoint. One possibility was that monitoring was unobtrusive and not recognized by the endoscopist. Phase Two was designed to test this theory and showed a significant increase in fentanyl use, with a decline in pain score but not total NAPCOMS. The correlation of increased fentanyl and decreased pain score shows that a quality control measure can affect physician activity.

Additionally, our data showed a difference between GI and GS, with GI using significantly more position changes. While this did not change NAPCOMS scores, there were differences in the amount of sedation used. These changes do not allow us to draw inferences between the two specialties but provide interesting insight into the preferences of endoscopists at this centre.

*Funding Agencies: *None

# Sub article: A61: Correlation of the St. Paul's Endoscopy Comfort Scale with Post-Procedure Pain Recollection following upper Endoscopy

**Authors:** J. Yonge, N. Harris, C. Galorport, O. Takach, I. Tavakoli, M. Suzuki, J. Amar, S. Whittaker, H. Ko, G. Rosenfeld, A. Ramji, B. Bressler, E. Lam, J. Telford, R. Enns

**Affiliations:** ^61.1^St. Paul's HospitalVancouverBCCanada; ^61.2^University of British ColumbiaVancouverBCCanada

*Background*. Patient comfort during endoscopy is an important measure of endoscopic quality and is associated with improved patient satisfaction and compliance with future procedures. We modified the original St. Paul's Endoscopy Comfort Score (SPECS) for colonoscopy to validate it for use in outpatients undergoing upper endoscopy.

*Aims*. To determine whether there is any correlation between SPECS for upper endoscopy and self-reported patient comfort and satisfaction.

*Methods*. 300 outpatients undergoing upper endoscopy at St. Paul's Hospital were prospectively enrolled between May and August 2015. Inclusion criteria: Age ≥ 19 years and outpatient upper endoscopy. Exclusion criteria: unable to speak and understand English, undergoing both colonoscopy and upper endoscopy at the same time, and unwilling or unable to complete the questionnaire. The SPECS and Gloucester Score (GS) were completed independently by the physician, nurse, and research assistant (RA). The RA also completed the Non-Verbal Pain Assessment Tool (NPAT) and Nurse Assessed Patient Comfort Score (NAPCOMS). Patient demographics were collected. Patients completed a patient satisfaction questionnaire and Visual Analogue Scale (VAS) that assessed the patient's pain and satisfaction. Cohen's kappa coefficient and Fleiss' kappa was used to assess the inter-rater reliability and agreement. This study was approved by the IRB.

*Results*. Mean age was 56 (range 19 to 88) and 160 subjects were male. VAS was used as the gold standard and compared to the other scales. VAS has slight agreement to all scales (no scale was significantly better when compared to VAS). SPECS and NAPCOMS had the highest *κ* scores. Patients were asked to rate their pain intensity (none, mild, moderate, and severe) during procedure. All scales showed slight agreement. SPECS and GS had the highest *κ* scores.

*Conclusions*. There was no significant difference in the correlation between any of the scales and the patients self-reported pain as a linear scale (VAS) or when organized categorically. SPECS was previously used as a tool to measure patient comfort in colonoscopy and could use further adjustment to be meaningfully applied to upper endoscopy.

*Funding Agencies: *None

# Sub article: A62: Adequacy of Documentation of Follow-Up Plans for Patients Undergoing Inpatient Colonoscopy

**Authors:** C. Parker, M. Brahmania, M. Kowgier, S. Sharma, T. Alomani, G. Malhi, A. Gulamhusein, N. Bollegala, M. Cino, A. Weizman, M. Bernstein, E. Irvine

**Affiliations:** ^62.1^University of TorontoTorontoONCanada; ^62.2^Toronto Western HospitalTorontoONCanada; ^62.3^Mount Sinai HospitalTorontoONCanada; ^62.4^Sunnybrook Health Sciences CentreTorontoONCanada; ^62.5^St. Michael's HospitalTorontoONCanada

*Background*. The transition of care from the inpatient to outpatient setting can be fragmented and may contribute to poor patient outcomes. Lack of appropriate follow-up for patients undergoing inpatient colonoscopy who are found to have colonic polyps may put the patient at risk for developing interval colon cancer. This may be related to inadequate documentation upon hospital discharge.

*Aims*. To assess the adequacy of documentation for appropriate follow-up among those with colonic polyps found during inpatient colonoscopy.

*Methods*. A retrospective chart review was performed on patients who had colonic polyps found during inpatient colonoscopy during a one year period at St. Michael's Hospital, Toronto, Canada. Discharge summaries were reviewed for adequate documentation of follow-up plans including the need for follow-up, time interval for follow-up, if required, and the contact information of the follow-up provider. Descriptive statistics were used to calculate the proportion of patients who had adequate documentation of follow-up plans upon discharge.

*Results*. 45 patients were included in the final analysis. All patients had a completed discharge summary. The need for follow-up was found in 46.7%, and the interval for follow-up in 24.4% of the discharge summaries. Contact information for the follow-up consultant was present in 17.8% summaries. 31 patients had one or more tubular adenoma (with or without high grade dysplasia) or tubulovillous adenoma. Of these 31 patients, 48.4% had the need for follow-up in their discharge summary, 22.6% had the interval of follow-up and 38.7% had the contact information of the follow-up provider. 27% patients had polyps that were not removed or retrieved at colonoscopy. Of these 12 patients, 50% had the need for follow-up in their discharge summary, 25% had the interval of follow-up recommended and 25% had the name of the consultant they were to follow-up with.

*Conclusions*. Adequate documentation of the need for follow-up was lacking in most discharge summaries of inpatients found to have colonic polyps during colonoscopy. The problem was magnified further in patients with adenomas or with polyps that were either not removed or not retrieved. This report highlights the importance of developing new initiatives to improve communication among healthcare providers at the time of discharge to ensure appropriate follow-up after inpatient colonoscopy.

*Funding Agencies: *None

# Sub article: A63: Endoscopic Evaluation of Graft-versus-Host Disease: Retrospective Review from a Tertiary Centre

**Authors:** S. Ip, V. Marquez, D. Schaeffer, F. Donnellan

**Affiliations:** ^63.1^University of British ColumbiaVancouverBCCanada

*Background*. Graft-versus-host disease (GVHD) is a complication of hematopoietic stem cell transplantation (HSCT) that frequently affects the gastrointestinal (GI) tract. The diagnosis requires pathologic confirmation from endoscopic biopsies; however, the ideal location of these biopsies has not been clearly established.

*Aims*. To determine the best sites for obtaining biopsies in evaluating GI GVHD.

*Methods*. All cases of biopsy-proven GI GVHD (GVHD+) were obtained from a pathology database over a two-year period at a tertiary centre (*n* = 46). Demographic, clinical, and endoscopic data were extracted. For comparison, a randomized sample of GVHD negative cases (GVHD−) was obtained (*n* = 50). Sensitivities for the diagnosis of GVHD at different sites of both the upper GI tract and colon were determined.

*Results*. Diarrhea was the most common symptom in both the GVHD+ and GVHD− groups. In the GVHD− group, they were commonly investigated with an esophagastrodudenoscopy (EGD) (60% versus 22% in the GVHD+ group, *p* < 0.01) while a colonoscopy (CLN) was commonly performed in the GVHD+ group (33% versus 12%, *p* = 0.02). Non-specific erythema was more often found in the GVHD+ group (*p* = 0.05). Among the GVHD+ patients, for EGDs, the sensitivity was highest for duodenal biopsies at 89%. There was only one case in which GVHD was not detected by duodenal biopsy but found on a gastric biopsy. For FS and CLN, the sensitivities among all sites were similar (85% agreement, kappa 0.58, *p* = 0.01). There were no cases in which GVHD was diagnosed in the right-side of the colon without a positive biopsy in the left-side of the colon. The grade of GVHD appeared to have no effect on sensitivities.

*Conclusions*. In this cohort of GI GVHD patients, duodenum biopsies seem to produce the highest yield for diagnosing GVHD with a sensitivity of 89% when compared to other sites of the upper GI tract. Sensitivities were similar among all sites on lower endoscopies, suggesting that a FS is sufficient for diagnosing GVHD in suspected patients with diarrhea. As shown in this cohort, CLNs may be overly utilized and unnecessary in the investigation for GVHD.

*Funding Agencies: *None

# Sub article: A64: Single Center Experience in the Use of Device Assisted Enteroscopy: A Retrospective Study

**Authors:** A. Benmassaoud, M. Sasson, C. Soulellis, T. Bessissow

**Affiliations:** ^64.1^McGill University Health CenterMontrealQCCanada

*Background*. Over the last 15 years, the endoscopic evaluation of the small bowel has gone through a major revolution with the development of device-assisted enteroscopy (DAE), including single and double balloon enteroscopy. Since then, it has been used for diagnostic and therapeutic purposes in various clinical situations such as obscure gastrointestinal bleeding (OGIB), Crohn's disease (CD) and small bowel tumors.

*Aims*. The main objective of this study was to evaluate the diagnostic and therapeutic yield of DAE in the evaluation and treatment of small bowel diseases using our database.

*Methods*. This was a single center retrospective cohort study from the McGill University Health Center. Adult patients who had a DAE between January 2010 and July 2015 were included. Patients were identified using a prospectively maintained database. Patients were excluded if data related to the enteroscopy was missing. Electronic and paper medical records were extensively reviewed. Demographic and clinical data was collected. A descriptive analysis of the recorded data was performed.

*Results*. 246 device-assisted enteroscopies were available for analysis. In our cohort, patients' median age was 64 years old (IQR 47–75), and were inpatients in 9% of cases. The three most common causes of referral were OGIB in 65%, CD in 9% and gastrointestinal malignancy or polyp in 8% of cases. DAE was anterograde in 92% and retrograde in 8% of cases. 58% of patients had a previous gastroscopy or colonoscopy, 17% had prior video capsule evaluation, and 17% had prior DAE. About 49% of patients had a CT scan before DAE and 40% had no previous imaging done. Sedation consisted mainly of a combination of Midazolam and Fentanyl in 96% of cases with average doses of 3.3 mg ± 1.6 mg and 93.2 mcg ± 39.1 mcg respectively. General anesthesia was required in 6 cases. Approximately 54% of entroscopies had positive findings. Amongst them, the three most common findings were an arteriovenous malformations, an ulcer or erosion and the presence of polyps or stricture in 43%, 26%, and 9% of cases respectively. A therapeutic intervention was deemed necessary in 34% of all cases, or in 62% of cases with a positive finding.

When compared to all comers, patients with a pre-endoscopic diagnosis of OGIB trended towards being more likely to have a positive finding (65% versus 54%, OR = 1.55, *p* = 0.0581) and were more likely to have treatment applied (52% versus 34%, OR = 2.13, *p* = 0.001).

*Conclusions*. Our study showed that the most common indication for the use of DAE was OGIB. Patients with a pre-endoscopic diagnosis of OGIB trended towards being more likely to have a positive finding and have treatment applied. Further studies are underway to validate these findings.

*Funding Agencies: *None

# Sub article: A65: Quality Improvement of Endoscopic Procedure Dictation Reports at St. Paul's Hospital: A Comparison of Transcriptions From 2008 and 2014

**Authors:** J. Yonge, N. Harris, C. Galorport, M. Suzuki, E. Nap-Hill, J. Amar, B. Bressler, E. Lam, J. Telford, R. Enns

**Affiliations:** ^65.1^St. Paul's HospitalVancouverBCCanada

*Background*. Following an esophagogastroduodenoscopy (EGD), the physician dictates their findings which are later transcribed and kept in patient's medical records. A dictation template for upper endoscopy was first introduced to St. Paul's Hospital approximately 3 years ago. The template was created based on the recommendations made by the ASGE and was revised by the endoscopists at St. Paul's. To evaluate the completeness of dictation reports, key points have been outlined based on the available literature and the current EGD reporting guidelines available at St. Paul's.

*Aims*. The purpose of this study is to assess and compare the quality and completeness of EGD procedure reports from 2008 and 2014 for physicians currently working at St. Paul's Hospital to determine if key quality elements of documentation were more consistently included following institution of a dictation template.

*Methods*. This was a retrospective chart review of dictation reports completed by gastroenterologists at St. Paul's Hospital from 2008 and 2014. 150 charts were reviewed for each doctor in each year. Data was collected from a comprehensive EMR system that included demographics, patient history, procedure report details (appropriate quality indicators as outlined by ASGE), and length of procedure. This study was approved by the IRB.

*Results*. The overall completeness for all gastroenterologists improved from 71.53% in 2008 to 76.82% in 2014 (*p* < 0.001). Most variables remained consistent or increased; however, reporting of comorbidities, medications, complications, and patient comfort remained low at both time periods.

*Conclusions*. The use of the dictation template has improved documentation of quality parameters from 2008 to 2014. The variables that are frequently missed from dictation reports have been identified and educational maneuvers as well as other adjustments to include these variables in future procedure reports can be targeted at these items.

*Funding Agencies: *None

# Sub article: A66: Inter-Observer Reliability of the St. Paul's Endoscopy Comfort Scale (SPECS) for upper Endoscopy

**Authors:** N. Harris, J. Yonge, C. Galorport, I. Tavakoli, O. Takach, M. Suzuki, J. Amar, S. Whittaker, H. Ko, G. Rosenfeld, A. Ramji, B. Bressler, E. Lam, J. Telford, R. Enns

**Affiliations:** ^66.1^St. Paul's HospitalVancouverBCCanada; ^66.2^University of British ColumbiaVancouverBCCanada

*Background*. Patient comfort is a key quality indicator for gastroscopy and is associated with enhanced patient satisfaction and improved compliance with future procedures. The St. Paul's Endoscopy Comfort Scale (SPECS) has been previously validated to evaluate patient comfort during colonoscopy; however, it has not yet been assessed for use during upper endoscopy. This scale has three categories namely Vocalization, Positioning/Body Language and Patient Anxiety/Emotion. Each category is scored from 0–3 based on the frequency and severity of specific indicators, yeilding a total score ranging from 0–9. Another tool used to assess patient comfort during endoscopy is the Gloucester scale (GS), which is a five point global rating system.

*Aims*. To modify SPECS for Colonoscopy for use during upper endoscopy, to compare the inter-observer reliability of SPECS for Upper Endoscopy to the GS, and to determine if the use of sedation during upper endoscopy affects the SPECS score.

*Methods*. 300 outpatients undergoing upper endoscopy at St. Paul's Hospital were enrolled (May 2015–August 2015). Inclusion criteria: outpatients ≥19 years scheduled for upper endoscopy. Exclusion criteria: individuals undergoing additional procedures during the same visit. SPECS and GS were completed independently by the physician, nurse and research assistant. To avoid behavioral bias, patients were debriefed of study aims post-procedure. Kappa statistical claculations were performed and patient demographics and sedation were documented. The study was approved by the UBC Ethics Board.

*Results*. Mean age of participants was 56.7 years and 46.7% were female. Sedation was used for 89.0% of cases; the mean Midazolam dose was 3.3 mg (SD 1.6) and the mean Fentanyl dose was 51.4 mcg (SD 29.7). The mean SPECS score, calculated using the average of the three observers, was similar for non-sedated and for sedated patients: 1.3 (SD 1.3) and 1.5 (SD 1.6), respectively.

*Conclusions*. SPECS for Upper Endoscopy demonstrated slightly higher inter-observer reliability than the GS, but the results were not statistically significant. Although not statistically significant, the category demonstrating the weakest inter-rater reliability was Positioning/Body Language and this may be due to the subjectivity in assessing the gag reflex. Sedation had no notable effect on the SPECS score.

*Funding Agencies: *None

# Sub article: A67: Incidence of Venous Thromboembolism in Gastrointestinal Bleeding

**Authors:** C. Sheasgreen, M. Almakadi, G. Leontiadis

**Affiliations:** ^67.1^McMaster UniversityHamiltonONCanada

*Background*. Venous thromboembolism (VTE) is a common complication of hospital admission. For patients admitted with gastrointestinal bleeding (GIB), confusion can arise as to whether it is in the patient's best interest to use pharmacological prophylaxis against VTE.

*Aims*. This was a pilot study to assess the use of VTE prophylaxis and the incidence of VTE in patients admitted to hospital with GIB.

*Methods*. Hospital charts of adult patients admitted for GIB from 2009–2011 at one centre in Ontario were reviewed. Charts were pulled in aliquots of 50 sequentially admitted patients. Those with previously diagnosed VTE, risk factors for VTE (malignancy, active inflammatory bowel disease, hypercoaguable state, thrombophilia, or myeloproliferative disorder), or hospital stay less than 24 hours were excluded. Patients were classified as having “confirmed" GI bleeding or “probable" GI bleeding based on reported history and physical exam. Criteria for being classified as “confirmed" included having hematochezia, melena, hematemesis, or coffee ground emesis observed by a physician or documented GIB on endoscopy at time of admission. Hospital records were reviewed for the presence of mechanical foci for thrombus formation (e.g., central venous catheters or inferior vena cava filters), smoking and alcohol use, admission to hospital within the previous 6 months, use of pharmacological prophylaxis for VTE while in hospital, death, and incidence of VTE within 6 months from index admission.

*Results*. 250 patient charts were reviewed. After exclusions, 125 patients were included in the analysis. 69 patients were “confirmed" GIB and 56 were “probable." 7 (10.1%) of the confirmed cases were given VTE prophylaxis whereas 11 (19.6%) of the probable cases received the same. There were 2 VTE events; a pulmonary embolism in “Patient A" and a right internal jugular vein thrombus in “Patient B," both of whom were confirmed GIB patients. Patient A had a history of cigarette and alcohol use and was not given pharmacological VTE prophylaxis. Patient B had a right central venous catheter and was given pharmacological VTE prophylaxis. 4 patients died, 2 of whom had been given VTE prophylaxis. Niether of the 2 patients with VTE died.

*Conclusions*. These data suggest that patients in whom the diagnosis of GIB is clinically obvious are less likely to receive pharmacological VTE prophylaxis and that this may translate into an increased risk for VTE events. VTE does not appear to increase the occurrence of death in GIB. A larger review encompassing more events will help deliniate these relationships further.

*Funding Agencies: *None

# Sub article: A68: Endoscopic Ultrasound in Nova Scotia, a Quality Assurance Study

**Authors:** A. Alghamdi

**Affiliations:** ^68.1^Dalhousie universityHalifaxNSCanada

*Background*. Endoscopic ultrasound (EUS) is technique that utilizes endoscopic technology with an ultrasound transducer at the tip to allow visualization of submucosal lesions, and structures surrounding the gastrointestinal tract. Newer technology has allowed real-time fine needle aspiration (FNA) to be performed under EUS guidance. It has proven to be a highly sensitive tool for diagnosing lesions in and adjacent to the gastrointestinal tract.

*Aims*. Since the single most important function of EUS is in its ability to obtain tissue via FNA, our primary outcome measure will be yield of FNA for the various indications. Secondary outcome measures will include the referral base, indications, waiting time and complications of EUS in Nova Scotia. This quality assurance study will help in improving the EUS program in our province.

*Methods*. It is an observational, retrospective cohort study of all the men and women who had undergone EUS in Nova Scotia, in the CDHA, throughout the calendar year of 2013. Subjects of this research consist of 114 patients. Patient files will be analyzed to determine the reason for referral to EUS, the complications if any, and the waiting time for an EUS appointment in the out patients sittings. Results of EUS with or without FNA will be charted as well as the diagnosis obtained via cytological analysis.

*Results*. The most common reasons for referral to EUS were for evaluation of pancreatic mass/cyst (44 patients, 39%), and assessment of sub-mucosal lesions (26 patients, 22.8%). Other indications were lymph node FNA (mostly mediastinal), Dilated CBD, pancreatic cancer screening, chronic unexplained pancreatitis. Rectal EUS were performed in 4 patients; in which 3 of them referred for fecal incontinence and 1 had para-anal mass for FNA.

A total of 49 FNA's were performed by EUS for different indications; most of them were from a pancreatic mass/cyst, Lymph node and submucosal lesions; 69, 10 and 8 percent respectively. 82% of total FNAs results were conclusive, either positive or negative; among the FNA obtained from a pancreatic mass 85% were conclusive, while FNAs from Lymph node and submucosal lesions were conclusive in 60 and 50 percent respectively. The most common abnormal FNA results from the pancreas were pancreatic adenocarcinoma (46%) and mucinous neoplasia (30.7%). Other results included pancreatic lymphoma, metastatic malignancy from lymph node FNA, lung cancer and anal cancer.

4 patients developed complications post EUS, 2 (1.7%) had pancreatitis and 2 (1.7%) had mild bleeding.

*Conclusions*. EUS can be used for variety of indications, most commonly to further characterize a pancreatic lesion, with the ability of obtaining a tissue diagnosis through FNA with good diagnostic yield that guided patients management. It is a minimally invasive procedure with low complication rate.

*Funding Agencies: *None

# Sub article: A69: A Retrospective Analysis of the Long-Term Outcomes of Patients with Hiatus Hernias, Cameron Erosions, and Iron Deficiency Anemia

**Authors:** E. Nap-Hill, C. Galorport, R. Enns

**Affiliations:** ^69.1^St. Paul's HospitalVancouverBCCanada

*Background*. Mechanical trauma of hiatus hernias (HH) can cause linear gastric erosions on the crest of the mucosal folds near the diaphragm called Cameron Erosions (CE). CE are an uncommon source of upper GI bleeding, and are usually identified by an upper endoscopy. The erosions bleed intermittently, become less prominent, then reappear, at times making the diagnosis difficult. There is no standard treatment regimen to remedy these lesions however most are managed with acid suppression. Long-term follow-up is uncommon in the literature.

*Aims*. This study aims to assess the long-term outcomes of patients with CE in regards to therapy required: oral/parental Fe, acid suppression, blood transfusion and/or surgery, and subsequently to determine risk factors for failure of conservative therapy.

*Methods*. A retrospective chart review of patients with HH and CE treated for iron deficiency anemia between January 2005 and June 2015 was performed. Data collected includes demographics, endoscopy dates, indication, findings, history of blood and iron transfusions, co-morbidities, history of GI surgery, medications, blood work, surgery, and hospitalizations. All patients had undergone upper and lower endoscopic examinations. Additionally, all patients referred for surgery underwent a capsule endoscopy to exclude small bowel etiology of bleeding.

*Results*. Preliminary data on 21 of 60 patients with CE is presented. 21 patients were identified with both CE, and iron deficiency anemia; followed for an average of 27 months. 67% of patients had their anemia successfully treated, while 33% had persisting anemia after five years. 43% of patients were referred with known CE as the likely cause of their anemia, while the other 57% were referred for obscure or occult bleeding, and later found to have CE. 34% of patients who were successfully treated, were treated conservatively with proton pump inhibitors (PPI), iron supplements, and occasional blood transfusions (33% required a mean of 2.3 units of blood over the course of their follow up). 33% did not respond to conservative therapy, and were successfully treated with laparoscopic repair of their HH.

*Conclusions*. Conservative treatment of PPIs, Fe supplements, and occasional blood transfusions has shown to be effective in treating most patients, however, many require ongoing monitoring and correction of anemia. In those that did not respond to conservative treatment, surgical correction of HH resolved anemia in all patients who underwent repair. Further study will determine risk factors for requiring more aggressive (i.e., surgical) therapy.

*Funding Agencies: *None

# Sub article: A70: Tidying up a Waitlist: A Quality Assurance Project

**Authors:** M. Sheridan, A. Dorreen, S. Williams

**Affiliations:** ^70.1^Dalhousie UniversityBedfordNSCanada; ^70.2^Dalhousie UniversityHalifaxNSCanada

*Background*. Access to Gastroenterology services is limited in Atlantic Canada, resulting in long waitlists for outpatient consultation. At our institution, the current wait time for non-urgent referrals exceeds three years. Periodic review of the referrals on such a long wait list is important to assess whether these patients still require consultation and to determine if their triage category is still appropriate. This report provides an update on a three-year waitlist validation pilot project carried out in our division.

*Aims*. The aim of this project is to evaluate non-urgent referrals from the 2013 calendar year to identify those referrals that no longer need to be seen or can be reassigned to a direct-to-endoscopy waitlist.

*Methods*. All non-urgent outpatient referrals with a date of referral in 2013 were identified. A letter was sent to the referring physician of each patient in the fall of 2014 asking if the referral was still necessary and if there had been a change in the patient's condition. If no response was provided, this letter was sent a second time. All responses were evaluated by a physician on the triage committee. Referrals that were deemed no longer necessary were removed from the waitlist. Referrals that were still deemed to be necessary were then re-triaged and, if appropriate, redirected to a direct-to-endoscopy waitlist.

*Results*. 404 referrals were evaluated, 66% (*n* = 270) of patients were women and 34% (*n* = 134) were men. The majority of referrals were sent by family physicians (91%, *n* = 366). The most common indications for referral were: reflux/dyspepsia 36% (*n* = 147), abdominal pain 17% (*n* = 68), colon cancer screening 14% (*n* = 57), diarrhea 12% (*n* = 49), celiac disease 4% (*n* = 15), stable IBD 3% (*n* = 14) and constipation 3% (*n* = 14). The response rate was 89% (*n* = 358). 65% (*n* = 262) of patients were deemed by the referring physician to still need to be seen. Of these, 29% (*n* = 76) were felt to be appropriate for a direct to procedure referral and only 2% (*n* = 8) were re-triaged to a semi-urgent waitlist.

*Conclusions*. A significant number of non-urgent referrals no longer require consultation after one year on a waitlist. Of those that do, very few need to be upgraded to a more urgent triage criteria. A significant proportion of these patients are appropriate for direct-to-procedure consultations. Periodic review of referrals with prolonged wait times can result in significant shortening of waitlists.

*Funding Agencies: *None

# Sub article: A71: The Use of High Volume Simethicone to Improve Visualization Quality during Small Bowel Video Capsule Endoscopy: A Pilot Study

**Authors:** D. Segal, B. Yan, N. Chande, T. Ponich, J. Gregor, M. Sey

**Affiliations:** ^71.1^Western universityLondonONCanada; ^71.2^London Health Sciences CentreLondonONCanada; ^71.3^The University of Western OntarioLondonONCanada; ^71.4^Los Alamos National LaboratoryLondonONCanada

*Background*. Poor bowel preparation affects up to one third of capsule endoscopy studies. Simethicone has been studied although its benefit has been inconsistent, possibly due to an inadequate volume being used.

*Aims*. The goal of this study is to compare standard volume with high volume simethicone for small bowel preparation during capsule endoscopy.

*Methods*. A double blind randomized clinical trial was conducted among outpatients undergoing capsule endoscopy. Patients were randomized to either 200 mL (standard volume) or 750 mL (high volume) of simethicone (1.5 mg/mL) 30 minutes prior to capsule ingestion. All patients received 2 L of PegLyte the night before the procedure and started fasting at midnight. Visualization quality (0–3) was assessed by a previously validated scale composed of the mean of the visualized mucosa (0–3) and degree of obstruction (0–3) scores.

*Results*. At the time of interim analysis, 20 patients had been randomized (10 standard volume and 10 high volume). The mean (SD) age was 64.1 (17.7) and 60% were females. The most common indication was obscure occult GI bleeding (50%). Compared to standard volume, the high volume group had higher visualization quality score (2.32 versus 2.45), visualized mucosa score (2.59 versus 2.67), and degree of obstruction score (2.18 versus 2.22) although this did not reach statistical significance given the interim analysis. This trend was seen in the proximal half, distal half, and when the entire small intestine was compared. There were no adverse events in either group.

*Conclusions*. In this interim analysis, a strong and consistent trend was seen in favour of high volume simethicone over standard volume simethicone for improved visualization quality during capsule endoscopy.

*Funding Agencies: *None

# Sub article: A72: Gastroenterology Curriculum in Canadian Medical Schools

**Authors:** T. Dang, C. Wong, L. Bistritz

**Affiliations:** ^72.1^University of AlbertaEdmontonABCanada; ^72.2^10240 Kingsway Ave.EdmontonABCanada

*Background*. Gastroenterology is a diverse subspecialty that covers a wide array of topics ranging from functional abdominal pain to gastrointestinal (GI) malignancies. The pre-clinical GI curriculum is often the only formal training that medical students receive prior to becoming residents. Recently, Canadian medical schools have shifted from didactic approaches to teaching to more interactive methods. Despite this change in teaching methodology and the diversity of topics in GI, there is no national consensus or awareness on content, learning objectives, or instructional methods for the GI curriculum at other Canadian institutions; this lack of consensus and awareness results in variable background knowledge for new residents and lack of guidance for curriculum development.

*Aims*. (1) Elucidate gastroenterology topics taught at the pre-clinical level.

(2) Determine instructional and assessment methods used at Canadian medical schools.

*Methods*. A survey of GI teaching topics, teaching methods, and assessment tools was developed by two educational content experts involved in organizing the GI curriculum at the University of Alberta. This survey was piloted internally and externally to gastroenterologists involved in organizing the GI curriculum at other institutions. The final questionnaire was sent to all the GI pre-clinical curriculum coordinators at all 17 Canadian medical schools in the October 2014. After receiving the responses from the different schools, a curriculum map of the GI topics from the different institutions was constructed and remaining results regarding curriculum content, teaching methods, and assessment tools were compiled.

*Results*. A curriculum map of GI topics was constructed from the responses gathered from 10 of the 17 Canadian medical schools and showed a heterogenous curriculum across the country. Topics often not covered included pediatric GI diseases, surgery/trauma, food allergies/intolerances, nutritional support and obesity. The curriculum was taught primarily by gastroenterologists and surgeons. Didactic teaching and small group teaching were the most common teaching methods employed. When broken down by topics, Liver Diseases used the most diverse teaching methods including small groups, online modules, and self-directed learning and the least amount of didactic teaching. Final summative exams and interim quizzes were the primary assessment tools used for evaluation.

*Conclusions*. This is the first study examining the GI curriculum at a pre-clinical level. Certain topics, such pediatric GI diseases, surgery/trauma and nutrition were not as well represented in responding programs. The data from this study can be used to reform existing curriculum or as a guide in future curriculum design.

*Funding Agencies: *None

# Sub article: A73: Initial Experience with Small Histological Cores Obtained via a New Eus-Guided Fine Needle Biopsy System

**Authors:** T. Ishikawa, R. Mohamed, P. Kumar, P. Belletrutti

**Affiliations:** ^73.1^University of CalgaryCalgaryABCanada

*Aims*. As EUS-guided tissue acquisition techniques evolve, there is increasing interest in obtaining histological samples to improve diagnostic accuracy. Several new “core" FNA needles are in development and one such needle system was tested in our tertiary care center.

*Methods*. A retrospective review of consecutive patients undergoing EUS-guided tissue sampling of solid lesions using the Beacon Sharkcore^©^ fine needle system. Three experienced endosonographers (>250 EUS cases per year) performed the procedures. Selection of needle gauge and number of passes were left to the discretion of the endoscopist but at least one pass was to be submitted in formalin for histological processing.

*Results*. Twenty-seven patients underwent 30 EUS-guided fine needle biopsy (FNB) procedures from June-Sept 2015. The specific lesions targeted were pancreas masses (16), lymph nodes (6), submucosal masses (7) and pancreas parenchyma (1). A 25 g needle was used in the majority of cases (66.7%). In 22 cases, both cytology and histology specimens were sent from the same tissue target. Of the 30 FNB specimens, 28 (93.3%) were adequate for histological examination and 25 (89.2%) of those were diagnostic specimens (includes 3 atypical/suspicious for adenocarcinoma). Two of the non-diagnostic FNB samples were proven to be adenocarcinoma after surgical resection. Of the 22 FNA cytology specimens, 20 (90.9%) were diagnostic (including 6 atypical/suspicious for adenocarcinoma). From our own historical data, based on 267 solid lesions in 244 patients (Jan 2013–May 2015), our FNA cytology diagnostic yield is 91.3%. Of the 22 cases with both cytology and histology sent, the majority yielded the same diagnosis (77.3%), but in 4 cases (18.2%) the FNB was diagnostic whereas the FNA was not; in 1 case (4.5%) only the FNA was diagnostic (FNB specimen was insufficient for analysis). There were no complications reported after FNB.

*Conclusions*. In this initial experience with a new EUS-guided FNB system we found that, even with a 25 g needle, obtaining small cores to submit for histological analysis is safe, technically feasible and usually provides an evaluable sample. In some cases only the FNB specimen yielded a diagnosis.

*Funding Agencies: *None

# Sub article: A74: Warm Carbon-Dioxide Insufflators Fail to Deliver Target Temperatures during Colonoscopies—An* Ex*-*Vivo* Study

**Authors:** F. Jowhari, K. Robertson, L. Hookey

**Affiliations:** ^74.1^Queen's UniversityKingstonONCanada; ^74.2^Hotel Dieu HospitalKingstonONCanada

*Background*. With the recent shift from air to carbon dioxide (CO_2_) for insufflation during adult colonoscopies, one manufacturer is now marketing a warm CO_2_ insufflator as a potential means of reducing pain & increasing tolerability during colonoscopies. While previous studies have shown some benefit with using warm water irrigation during colonoscopies, no studies exist assessing outcomes with warm CO_2_ insufflation. For this to even have potential for similar effects, the warm CO_2_ insufflator would first need to deliver the desired temperature of gas to the distal end of the colonoscope.

*Aims*. To assess whether warm CO_2_ insufflators deliver target temperatures to the distal end of the colonoscope, in a simulated environment replicating close to core body temperatures.

*Methods*. Three CO_2_ insufflators manufactured by Olympus® (Olympus UCR), Medivators® stratus™ (EGA-501, with the heating option) & Bracco (EZEM-CO_2_ effecient®) were chosen for this study. Using two adult colonoscopes (Olympus® (CF-H180DL) & Pentax (EC-3890Li)) with their lights on, the air button was constantly depressed & temperatures were recorded at each insufflator end & distal colonoscope end for 10 min in increments of 1 min (assuming an average cecal intubation time of ~10 min). Experiments were performed both at room temperature, and with the scope immersed in a warm water bath maintained at 34°C, as well with heat on & off for Medivators stratus. Mean temperatures were then compared at 0, 5 & 10 minutes using a one-way ANOVA, with the level of significance established at *P* < 0.05.

*Results*. The insufflator end temperatures between the heater on & off groups were similar at time 0 min (*P* = 0.474); but a difference was detected at 5 min (*P* < 0.001) & 10 min (*P* < 0.001). In spite of this, no difference was seen in the scope tip temperatures between the heater on & off groups at 0 min (*P* = 0.812), 5 min (*P* = 0.723) or 10 min (*P* = 0.621). With the heater on, temperatures at the scope tip & the insufflator end were similar at 0 min (*P* = 0.714), but did show statistically significant difference at 5 min (*P* = 0.001) & 10 min (*P* < 0.001). The addition of a warm water bath maintained at 34°C made no difference to scope tip temperatures at 0 min (*P* = 0.178), 5 min (*P* = 0.148) & 10 min (*P* = 0.159).

*Conclusions*. Our data suggests that although they warm the gas at the insufflator end, a new model of heated CO_2_ insufflators make no difference to delivered temperatures at the distal colonoscope tip. For reasons unclear, they fail to deliver target temperatures to the distal colonoscope end both at room temperature & in a heated body simulating a real colonoscopy. One possibility is the dissipation of heat as heated CO_2_ passes through the length of the colonoscope umbilicus; however, further studies are needed to demonstrate this conclusively.

*Funding Agencies: *None

# Sub article: A75: A Phase 1b/2a Randomized Open-Label Study Measuring Chyme Concentrations of Intravenously Administered Ceftriaxone in the Presence of the Oral Beta-Lactamase SYN-004

**Authors:** M. Rufiange, S. Boily, E. Sicard, M. Lefebvre, T. Roberts, H. Whalen, O. Coughlin, J. Kokai-Kun, J. Sliman

**Affiliations:** ^75.1^Algorithme PharmaLavalQCCanada; ^75.2^Synthetic Biologics Inc.RockvilleMDUSA

*Background*. SYN-004 is an oral recombinant *β*-lactamase developed by Synthetic Biologics, USA, and intended to degrade*β*-*lactam *antibiotics excreted into the gut, thereby mitigating their compromising effects on the gut microbiome, and preventing opportunistic hospital-acquired, bacterial infections such as* C*.* difficile*.

*Aims*. The purpose of this study was to evaluate the ability of SYN-004 to degrade ceftriaxone secreted into the small intestine after IV administration without affecting antibiotic pharmacokinetics (PK) in the bloodstream.

*Methods*. Nine otherwise healthy subjects with functioning ileostomies, aged 18–80 years, were enrolled at Algorithme Pharma Inc., a Phase I Clinical Research Organization based in Montreal, Canada. In the 1st treatment period, all subjects received an infusion of ceftriaxone and, in the 2nd period, 2 single oral doses of SYN-004 (75 or 150 mg) twice in 1 day (morning and early afternoon), with a single IV dose of 1 g ceftriaxone 30 min following the first dose of SYN-004. Subjects were confined in the clinical unit for 24 hrs, and medical monitoring lasted 1 week after the end of the second period. PK sampling was up to 8.5 hrs post-dose, both in plasma and chyme.

*Results*. The uniqueness of this study design resides in the special population that are patients with ileostomies. The presence of the ileostomy permits access to the chyme, the semifluid mass expelled in the ostomy bag. This constitutes a direct measurement of the degradation of ceftriaxone in the small intestine. Preliminary results show that the 75 and 150 mg single doses of SYN-004 as well as the single dose of ceftriaxone were very well tolerated. AEs were mostly mild to moderate in intensity, and all patients had recovered from their AEs at the end of their study participation. The most frequently observed AE was headache.

*Conclusions*. The clinical portion of the unique Phase 1b/2a study was successfully completed. The methodology used in this study of sequential sampling and analyzing the chyme should prove to be a powerful approach to allow the direct measurement of PK/PD for oral medications whose primary mechanism of action occurs in the gut. Chyme concentration analyses for ceftriaxone and the plasma PK analyses for systemic ceftriaxone are ongoing at the time of writing this abstract. SYN-004 is currently being investigated in a multicenter, placebo-controlled Phase 2b study in hospitalized patients being treated with IV ceftriaxone for lower respiratory infections. The primary outcome of the study will be prevention of* C*.* difficile* infection, with a secondary outcome of prevention of antibiotic-associated diarrhea.

*Funding Agencies: *None

# Sub article: A76: Results of the 2014 Equity and Gender Survey of the Canadian Association of Gastroenterology

**Authors:** S. Perera, L. Bistritz, M. Beaton

**Affiliations:** ^76.1^London Health Sciences CentreWestern UniversityLondonONCanada; ^76.2^Royal Alexandra HospitalUniversity of AlbertaEdmontonABCanada

*Background*. Achieving equity remains an ongoing challenge within the field of gastroenterology.

*Aims*. The Equity & Gender Committee of the Canadian Association of Gastroenterology (CAG) developed a survey to identify issues pertaining to equity and gender faced by its membership, as well as to determine potential areas of action that would be of most benefit.

*Methods*. In 2014 a survey was emailed out to all members of the CAG.

*Results*. A total 111 members (52% female and 48% male) responded to the study, which was a response rate of about 10%. The majority (75%) of respondents were between 26–45 years of age, and 55% were in their first 10 years of practice. Field of specialization varied with 51% in clinical adult or pediatric gastroenterology practices, 20% were basic scientists and 20% were residents or fellows. Sixty five percent worked in academic settings, while 13% worked in the community. Commitments outside of the workplace included a spouse or partner for 81% of respondents, with 52% having children under 18 years of age. Furthermore, 45% of members surveyed stated that they cared for an aging relative or had another significant area of non-work related responsibility. 70% of the respondents surveyed stated that they were either satisfied or very satisfied with their career path. The majority (77%) did not feel that age, gender, ethnicity or marital status had affected the advancement of their careers. However, 58% felt that gender and equity challenges exist within gastroenterology. To ascertain where CAG might assist in addressing the issue of equity, members were also asked to use a five-point Likert scale to rate the importance of several areas of interest. Of those surveyed, 87% ranked work life balance as important or very important, while 70% felt physician wellbeing and leadership skills were important or very important. Other areas that were highlighted were negotiation skills and academic promotion. Of potential areas for CAG involvement, mentoring and networking were ranked as important or very important by 55% and 56% respectively of the members surveyed.

*Conclusions*. This survey highlights that gender and equity challenges continue to exist within gastroenterology. Furthermore, this study revealed that work life balance, physician wellbeing and negotiation skills are areas of importance to many CAG members. The results of this survey also underscored that creating mentoring and networking opportunities were two potential areas that would be of benefit to the CAG membership.

*Funding Agencies: *CAG

# Sub article: A77: Treatmen of a Refractory Anastomotic Stricture Post-Low Anterior Resection Using a Fully Covered Enteral Stent: Case Report

**Authors:** S. Alshankiti, R. Sultanian, H. Wang

**Affiliations:** ^77.1^University of AlbertaEdmontonABCanada

*Background*. The complication of anastomotic stricturing post-low anterior resection (LAR) has been reported to occur in 5.8% to 20% of cases. Through-the-scope and over-the-wire dilation techniques are both effective and safe for treatment of benign colorectal anastomotic strictures, but often these strictures are refractory to conventional dilation and require surgical revision. Endoscopic stenting of LAR strictures is challenging due to the close proximity to the anal canal.

*Aims*. We report a case of an anastomotic stricture 4 cm proximal to the anal verge that occurred post-LAR for rectal cancer that was managed successfully by inserting a fully covered colonic stent.

*Methods*. Case Presentation: A 57 years old gentleman underwent a screening colonoscopy after having a positive fecal immunochemical test (FIT) in January 2014. He was found to have a rectal adenocarcinoma and was referred for surgical resection. He underwent low anterior resection with primary anastomosis and temporary diverting loop ileostomy. His post-operative period was complicated by an anastamotic leak that was managed in a conservative fashion with antibiotic treatment and a drain.

A flexible sigmoidoscopy exam 4 months after his surgery demonstrated an extremely tight stricture 4 cm proximal to the anal verge at the site of the rectal anastomosis. Over a six month period, the patient underwent a total of 9 endoscopic dilations via CRE balloon without sustained effect achieved. In addition to the balloon dilations, the stricture proved to be refractory to Triamcinolone injection and needle-knife stricture incision. The patient then underwent successful placement of Hanaro fully-covered 6 cm stent across the stricture. No immediate or delayed complications occurred.

*Results*. Three months later, the stent was endoscopically removed without difficulty and the anastomosis was widely patent with no significant stricture remaining. After an additional 3 months, a repeat flexible sigmoidoscopy again showed a fully patent anastomosis. Soon after this, the patient went on to have an uneventful reversal of his loop ileostomy and has been asymptomatic since.

*Conclusions*. This is the first published case report about using fully covered stent to successfully manage a refractory anastomotic stricture after LAR treatment of a rectal cancer. Further studies are needed to determine optimal strategies to treat distal rectal strictures with endoscopic stenting.

*Funding Agencies: *None

# Sub article: A78: Academic Outputs and Utility of Grit Course Abstract Presentations: The UBC Experience

**Authors:** F. AlShatti, E. Yoshida, V. Marquez

**Affiliations:** ^78.1^UBCVancouverBCCanada; ^78.2^University of BCVancouverBCCanada; ^78.3^University of British ColumbiaVancouverBCCanada

*Background*. The Gastroenterology Residents-in-Training (GRIT) Course is held in conjunction with the annual Canadian Digestive Disease Week. Its predecessor was the Post Graduate Course in Gastroenterology. The format of the GRIT Course, and its predecessor, requires Gastroenterology trainees to submit an abstract, and if accepted, they are then allowed to attend the meeting. At UBC, it is strongly recommended that trainees submit to the meeting. The academic utility of the experience to the trainee and the outcome of the submitted abstracts, however, remains unknown.

*Aims*. To assess the utility of the GRIT course from a UBC academic perspective by reviewing the outcomes (including publication and presentation at international meetings) of the projects submitted and to determine the value of the process to the trainees.

*Methods*. A list of former Gastroenterology trainees was obtained from the UBC database. A questionnaire composed of 11 multiple choice questions was sent to all former and current trainees.

*Results*. 88.8% of fellows responded (32 of 36). 43.75% are currently working in Academic Centers, 37.5% are in the Community, and 18.75% are still in training (that may be extra to core GI training). The abstract was a case report (33.3%), a clinical research (61.9%), or a basic science project (4.8%). 43.75% were presented at international meetings. 68.75% were published (only one was a non-peer review paper). The reasons for not publishing were: “Too busy and not enough time given during my training" (22.2%), “the abstract was appropriate for the GRIT/CDDW meeting, I did not feel that it was strong enough to be published in a journal" (44.5%) “the abstract reported work that was part of a greater research project and I was not significantly involved in the overall project" (33.3%). 21.8% received awards for their projects in GRIT either at the GRIT or at UBC trainee research days. 68.3% thought the GRIT experience was worthwhile, although one responder thought it was irrelevant.

*Conclusions*. We can conclude that more the two third of the projects submitted to GRIT were published, although less than half were presented internationally. The main reason for not publishing was that the abstract was not felt strong enough to be published. Most responders thought that the GRIT experience was worthwhile.

*Funding Agencies: *None

# Sub article: A79: An Atypical Intra-Abdominal Mass in a 28 Year Old Crohns Patient on Longterm Azatioprine and Infliximab

**Authors:** G. Eustace, J. Marshall

**Affiliations:** ^79.1^McMaster UniversityOakvilleONCanada; ^79.2^McMaster University Medical CentreHamiltonONCanada

*Aims*. This report presents the case of a young man with longstanding Crohn's disease, presenting to the hospital with a new atypical intra-abdominal mass of unknown etiology. With his azathioprine use in mind, lymphoma or other malignancy was considered along side an inflammatory mass related to his poorly controlled IBD. The atypical features of his mass and the diagnostic work up, as well as a framework for investigating similar clinical problems in the future will be discussed.

*Methods*. The patient was diagnosed with terminal ileal Crohns disease in 2011 and managed on azathioprine monotherapy. Infliximab was added in early 2015 after worsening symptoms and evidence of penetrating disease on an MR enterography. He then presented to the Juravinski Hospital, a large tertiary care center in Hamilton, ON on August 12th 2015 with concerns of multiple intra-abdominal abscesses visualized on an outpatient ultrasound. CRP was grossly elevated at 197 mg/L but bowel symptoms were unremarkable. The patient also complained of ongoing lower back pain.

*Results*. Intravenous antibiotics were initiated. A CT scan reported an infiltrative soft tissue mass, extending off of the small bowel into the mesenteric leaves and encasing the SMA, transverse duodenum, and pancreatic head. Associated necrotic adenopathy yielded differential diagnoses of malignancy, sclerosing retractile mesenteritis and IBD-associated fibrosis. After discussions with interventional radiology, percutaneous biopsy was deemed not to be possible. An endoscopic ultrasound guided biopsy was performed, and FNA identifiefd only benign glandular cells with evidence of chronic inflammation. Serial monitoring of the patient's mass is ongoing.

*Conclusions*. This case illustrates an atypical mass in a young man around which there was some diagnostic uncertainty. Although only 36 cases of thiopurine-associated hepatosplenic T cell lymphoma have been described in IBD patients ^1^, our patient's young age and gender raised this concern. More commonly, treatment of IBD with azathioprine carries a four-fold increase risk of lymphoma based on a 2005 review by Kandiel et al. ^2^Finally, the diagnosis of sclerosing mesenteritis was raised, a condition that may affect up to 0.6% of the population based on a recent review ^3^. The key in this case was communication with our radiologists along with quick access to EUS guided FNA. While our patient's mass was thankfully benign, his case can provide a framework for workup of similar patients in the future.

*Funding Agencies: *None

# Sub article: A80: The Snare Project: Closing the Loop on Synoptic Endoscopic Reporting and Skills Assessment

**Authors:** P. Rossos, T. Xenodemetropoulos, K. Lane, D. Armstrong

**Affiliations:** ^80.1^University of TorontoTorontoONCanada; ^80.2^McMaster UniversityHamiltonONCanada; ^80.3^Techna InstituteTorontoONCanada

*Background*. Structured reporting improves the completeness and timeliness of procedure reports to ensure effective communication and data capture. Adoption barriers to endoscopy EMRs include costs, workflow, lack of optimized content and inability to incorporate clinical best practice. Traditional mechanisms of endoscopic skill assessment are inherently biased and do not support objective comparative analysis. Peer-comparator practice audits have demonstrated a basis for evaluating variation while providing opportunities to improve clinical practice.

*Aims*. The Structured Notes Auditing and Reporting in Endoscopy Project combines synoptic point of care clinical reporting through a recently developed pan-Canadian data model with the Practice Audit in Gastroenterology (PAGE) program. The combined data model will be supported through CAG to serve as the first national initiative to combine standardized reporting, quality indicators and endoscopist practice and performance feedback.

*Methods*. Endoscopists were engaged nationally to achieve consensus on clinical content, terminology, performance, data quality indicators and patient outcomes to develop a Pan-Canadian data model. A National Endoscopist Working Group representing academic, community, adult and pediatric practice reviewed and incorporated elements and indicators from CAG Consensus Guidelines, Clinical Outcomes Research Initiative (CORI), UK Global Rating Scale (GRS), Colonoscopy Reporting and Data System (CO-RADS) and Minimal Standard Terminology (MST). Data elements were defined as either essential or optional. Participants were advised to consider use generically across technology platforms.

PAGE is a mobile IT based evaluation instrument developed for the CAG Quality Program in Endoscopy. It is a well-accepted, simple mechanism of administering peer-comparator practice audit to Gastroenterologists in independent practice. An initial SNARE pilot will combine the national trainee RPAGE program with the pan-Canadian data model at the University Health Network in Toronto to evaluate the feasibility of a scalable approach towards a nationally administered program based on CAG and industry partnerships.

*Results*. For presentation and discussion:

1. Pan-Canadian data models for colonoscopy and upper GI endoscopy; lessons learned for establishing other clinical procedures.
2. RPAGE instruments for comprehensive professionalism and performance evaluation with anonymized, peer-comparator functions.
3. SNARE pilot Results.

*Conclusions*. The integration of synoptic reporting and practice audit represents a unique Canadian opportunity to support clinical and industry collaboration to achieve both endoscopic data capture and objective performance feedback.

*Funding Agencies: *CAG, Canada Health Infoway

# Sub article: A81: Upper Gastrointestinal Bleeding due to Gastric Stromal Tumor-One of the Forgotten Differentials

**Authors:** S. Bharadwaj, M. Alzahrani, R. Alkhiari, R. Al-Dabbagh, T. Gohel, R. Spaziani

**Affiliations:** ^81.1^McMaster UniveristyHamiltonONCanada; ^81.2^McMaster UniversityStoney CreekONCanada

*Background*. Gastro-intestinal stromal tumours are the most common mesenchymal tumours of the gastro-intestinal tract. This case report highlights the importance of GIST in patients with no known risk factors for gastrointestinal bleeding.

*Aims*. This case report highlights the importance of GIST in patients with no known risk factors for gastrointestinal bleeding.

*Methods*. Case report and literature review.

*Results*. 54 year old female with past medical history of iron deficiency anemia and menorrhagia for which she underwent dilatation and curettage came with chief complaint of melena for 2 days. No known risk factors of gastrointestinal bleeding was elicited in history except for 1 dose of oral naproxen given prior to the procedure. Subsequently, also had a syncopal episode. On physical examination, was orthostatic and hypotensive. Rectal examination was evident for melena. Laboratory investigations showed a drop in hemoglobin from baseline of 114 to 83 g/L and also elevated BUN. After initial resuscitation with IV fluids and pantoprazole drip, EGD done showed an ulcerated sessile polyp about 5 cm in diameter at the gastric body. The suspicion of GIST tumor was confirmed by a CAT scan of the abdomen. A biopsy was not obtained due to friable nature of the polyp.

*Conclusions*. Gastro-intestinal stromal tumours (GIST) are the most common mesenchymal tumours of the gastro-intestinal tract (GI). They account for approximately 0.1 to 3% of all GI neoplasms. In patients with no known risk factors for gastrointestinal bleeding, GIST should be suspected as one of the etiologies.

*Funding Agencies: *None

# Sub article: A82: PAR2 Activation Inhibits Epithelial Wound Healing by Affecting E-Cadherin Expression and Lamellipodia Formation

**Authors:** E. Trusevych, P. Beck, W. MacNaughton

**Affiliations:** ^82.1^University of CalgaryCalgaryABCanada; ^82.2^Univ CalgaryCalgaryABCanada

*Background*. Protease-activated receptors (PARs) and their activating enzymes have been postulated to play a role in IBD pathogenesis. While previous studies have shown that PAR2 is highly expressed on intestinal epithelial cells and its activating enzymes are increased in IBD, the specific roles of PAR2 in disease initiation and progression remain unclear. However, PAR2 activation has both pro-proliferative and pro-migratory effects, and could be involved with restoration of the epithelial barrier following injury.

*Aims*. We tested the hypothesis that activation of PAR2 could increase the rate of epithelial wound healing.

*Methods*. Using colonic epithelial Caco2 cells, PAR2 was activated with the selective activating peptide 2f-LIGRLO (2fLI 0.5 *μ*M–10 *μ*M). For wound healing experiments, circular wounds were made in cell monolayers with a pipette tip and monitored with live-cell imaging. Proliferation was measured using an EdU assay. For immunofluorescence, wide-field images of E-cadherin and F-actin were taken at 20x and stitched together to capture the entire wound border and surrounding cells. To visualize actin dynamics, cells were transfected with a LifeAct plasmid to GFP-tag actin. Live cell videos were captured on a spinning disk confocal over 24 hr.

*Results*. Contrary to our hypothesis, PAR2 activation with 2fLI significantly inhibited the rate of wound closure over 48 hr (79.3 ± 2.5% wound closure) compared to control (94.3 ± 0.5%). In confluent monolayers, 2fLI was able to significantly increase proliferation of Caco2 cells (32.6 ± 3.6% EdU+ cells) compared to control (25.2 ± 6.5). However, 2fLI did not affect proliferation in wound-edge cells. Since both adherens junction and actin dynamics can affect epithelial migration, we next imaged the entire border of a wound stained for E-cadherin and F-actin. In control cells, there was a distinct loss of E-cadherin in cells surrounding the wound edge that was not seen in PAR2-activated cells. 2fLI treatment also resulted in a prominent actin cable surrounding the wound and prevented leader cell formation. Using LifeAct to visualize actin dynamics, 2fLI-treated cells could form filipodia projections but lacked lamellipodia. The actin cable appeared to prevent cells from migrating to close the wound.

*Conclusions*. We uncovered a novel effect of PAR2 activation, where 2fLI was able to inhibit wound closure in Caco2 cells. Although PAR2 activation had no effect on proliferation in wound-edge cells, it significantly slowed the rate of wound healing by inhibiting cell migration. PAR2 activation may be preventing the internalization of E-cadherin, which could prevent leader cell formation and sheet migration, in addition to inhibiting lamellipodia formation. Future directions include determining the role of RhoGTPases following PAR2 activation.

*Funding Agencies: *CCC, Alberta IBD Consortium, Alberta Cancer Foundation

# Sub article: A83: ATP-Induced Inflammasome Activation Increases Bacterial Clearance through ROS Production

**Authors:** M. Bording-Jorgensen, M. Alipour, G. Danesh, E. Wine

**Affiliations:** ^83.1^University of AlbertaEdmontonABCanada

*Background*. The proinflammatory cytokine interleukin (IL)-1*β* is released from macrophages and monocytes through a class of protein complexes called inflammasomes. Nod-like receptor protein-3 (NLRP3) inflammasomes have been linked to various inflammatory conditions such as inflammatory bowel diseases (IBD). Conditions associated with inflammasomes are typically characterized by an overabundance of IL-1*β* with the exception of IBD, where its dysregulation leads to an IL-1*β* reduction. ATP has been shown to be protective against* Escherichia coli *and* Staphylococcus aureus* infections. The mouse pathogen* Citrobacter rodentium*, a common mouse model pathogen for enteropathogenic* E*.* coli*, is used to understand the dynamic relationship between pathogens, the inflammasome, and the epithelial barrier. We have previously shown that* NLRP3* ^−/−^ mice given exogenous IL-1*β* had improved ability to clear* C*.* rodentium* infections. Our hypothesis was that ATP-induced inflammasome activation increases macrophages ability to eliminate* C*.* rodentium*, possibly through ROS activation.

*Aims*. To determine whether ATP-induced inflammasome activation decreases intracellular bacterial survival and define mechanisms involved.

*Methods*. Gentamicin protection assay with J774A.1 cell line macrophages was used to determine the rate of phagocytosis and bacterial killing. ATP (2.5 mM; NLRP3 activator) was utilized to stimulate endogenous IL-1*β* production; Apocynin, Diphenyleniodonium (DPI), and N-acetyl cysteine were used as ROS inhibitors. IL-1*β* was measured using an ELISA on supernatants and cell lysates. ROS production was measured using DCFDA.

*Results*. Activation of the inflammasome, using extracellular ATP, significantly increased the ability of J774A.1 macrophages to kill* C*.* rodentium* and this was associated with an increase in ROS production. ROS inhibition, using NAC and Apocynin, resulted in a reduction of microbial death and ROS production while DPI did not. Cytokine analysis showed that the secretion of IL-1*β* was not different between treatments.

*Conclusions*. Inflammasome activation appears to play a critical role in the clearance of pathogens, mediated by ROS activation, whether through direct pathogen elimination or localizing the immune response. In relation to IBD, this dysregulation of the inflammasome may contribute to an increase in host susceptibility to pathogens. Studying the role of IL-1*β* on macrophage activity during inflammation will lead to a better understanding of inflammatory diseases.

*Funding Agencies: *None

# Sub article: A84: Investigating the Underlying Mechanisms of Aquaporin 3 Involvement in Intestinal Epithelial Cell Proliferation

**Authors:** E. Morgan, M. Peplowski, W. MacNaughton

**Affiliations:** ^84.1^University of CalgaryCalgaryABCanada; ^84.2^Univ CalgaryCalgaryABCanada

*Background*. Aquaporin 3 (AQP3) is an aquaglyceroporin that is permeable to water and small solutes, such as glycerol and urea, and is known to be associated with cell proliferation, cell death and migration. In inflammatory bowel disease (IBD), water balance is disturbed resulting in impaired absorption and secretion, as well as barrier dysfunction. While aquaporins are expressed throughout the gastrointestinal tract, little is known about the physiological regulation of AQPs in intestinal epithelial cells.

*Aims*. We aim to better understand the functional importance of AQP3 in intestinal epithelial cells. We hypothesize that reduced AQP3 expression will result in reduced proliferative capacity, greater susceptibility to induced cellular stress, and decreased cell survival.

*Methods*. A stable AQP3 knockdown in HT29 human adenocarcinoma cells was developed using short hairpin RNA (shRNA). Cell proliferation was assessed in AQP3 knockdown cells, non-targetting scrambled shRNA control cells as well as untransfected HT29 cells over 72 hours under 10% FBS conditions. Fluorescence microscopy with EdU staining was also used to confirm active cell proliferation. The level of apoptosis was determined by detection of cleaved caspase-3 and cleaved poly (ADP-ribose) polymerase (PARP) using western blot. Cells were treated with 40 ng/mL IFN*γ* and 10 ng/mL TNF*α* and cleaved caspase-3 and cleaved PARP products were assessed over 24 hours. Necrosis was assessed by lactate dehydrogenase (LDH) assay over 72 hours. Flow cytometry was used to assess cell cycle progression using propidium iodide nucleid acid binding dye, which was quantified using FlowJo software.

*Results*. At 72 hours, AQP3 knockdown cell clones exhibit reduced proliferation by 47–61% under 10% FBS conditions. AQP3 knockdown cells also exhibit significantly reduced EdU incorporation, confirming decreased active DNA synthesis. Western blot did not show differences in apoptosis markers at baseline levels or following cytokine-induced apoptosis, nor were there evelated levels of LDH indicating that AQP3 knockdown cells are not undergoing significantly increased levels of cell death. However, AQP3 knockdown cells display significant differences in progression through the cell cycle with increased accumulation of cells in the G2 phase.

*Conclusions*. We have shown that AQP3 promotes proliferation in intestinal epithelial cells which appears to be due to altered progression through the cell cycle, and not due to increased rates of cell death. Our data improve the understanding of the functional role and importance of AQP3 in intestinal epithelial cell homeostasis and could lead to improvements in managing water balance, as well as improved treatments for IBD.

*Funding Agencies: *None

# Sub article: A85: CD4+ T Cell Derived TNF**α** Is Elevated in Patients with Diarrhea-Predominant but Not Constipation-Predominant Irritable Bowel Syndrome

**Authors:** Y. Nasser, C. Petes, C. Simmers, K. Gee, S. Vanner

**Affiliations:** ^85.1^Queen's UniversityKingstonONCanada

*Background*. Systemic immune activation with sustained, low-grade inflammation is thought to underlie the pathogenesis of irritable bowel syndrome (IBS), but these findings have been inconsistent. We hypothesized that this inconsistency is due to the heterogeneity of IBS and the variability of immune expression measured in serum.

*Aims*. Our primary aim was to assess whether CD4+ T-cell derived pro-inflammatory cytokines were elevated and if this was confined to subsets of IBS patients. To gain insights into potential mechanisms, our secondary aim was to assess whether this immune activation correlated with the severity of psychological scores.

*Methods*. IBS patients (*n* = 28) or healthy volunteers (*n* = 29) were recruited from the outpatient gastroenterology clinic at the Hotel Dieu Hospital. CD4+ T-cells were isolated from blood and incubated with media or phytohaemagglutinin (PHA) at 5 *μ*g/mL for 24 hrs. Supernatants were analyzed for the production of TNF*α*, IL-6 and IL-10 by ELISA. Subjects also completed validated psychological, symptom severity (IBS-SSS) and quality of life (QOL) questionnaires.

*Results*. PHA-stimulated cytokines were unchanged in IBS patients when compared to controls. When patients were analyzed by IBS subgroup, a significant increase in PHA-stimulated TNF*α* was seen in IBS-D but not IBS-C patients when compared to controls (201.3 ± 46.9 pg/mL, *n* = 29 Controls; 639.5 ± 225.9 pg/mL, *n* = 11 IBS-D; 117.3 ± 32.7 pg/mL, *n* = 10 IBS-C. *p* = 0.0159, Kruskal-Wallis Test; Control versus IBS-D and IBS-D versus IBS-C). IBS-SSS were in the moderate severity range (279.1 ± 14.93 IBS; 17.7 ± 3.7 Controls, *p* < 0.0001), yet these patients still exhibited significantly worse QOL (105.7 ± 7.4 IBS; 2.1 ± 0.6 Controls, *p* < 0.0001), increased anxiety (10.1 ± 0.9 IBS; 4.6 ± 0.6 Controls, *p* < 0.0001), depression (5.8 ± 0.7 IBS; 1.6 ± 0.3 Controls, *p* < 0.0001), and somatization (14.3 ± 0.8 IBS; 4.1 ± 1.2 Controls, *p* < 0.0001) scores when compared to controls. However, no differences in symptom severity or psychological scores were noted between IBS-C and IBS-D patients. CD4+ derived TNF*α* was not correlated with psychological or symptom severity scores.

*Conclusions*. IBS-D but not IBS-C patients have increased CD4+ T-cell derived TNF*α* when compared to controls, suggesting there is immune activation in IBS-D patients. This may suggest different underlying mechanisms in IBS-D compared to IBS-C. The cytokine elevation was not correlated with psychological scores however, suggesting that these parameters may be not mechanistically linked.

*Funding Agencies: *CAG, CCC, CIHR, YN is the recipient of a SEAMO (South Eastern Ontario Academic Medical Association) clinical fellowship. CP is supported by a Dr. Robert John Wilson Graduate Fellowship.

# Sub article: A86: The Pekin Duck Programmed Death Ligand-2: CDNA Cloning, Genomic Structure, Molecular Characterization and MRNA Expression Analysis

**Authors:** Q. Yao, K. Fischer, L. Tyrrell, K. Gutfreund

**Affiliations:** ^86.1^University of AlbertaEdmontonABCanada; ^86.2^Li Ka Shing Institute of VirologyEdmontonABCanada; ^86.3^Department of MedicineEdmontonABCanada

*Background*. Programmed death ligand 2 (PD-L2) plays an important role in the attenuation of adaptive immune responses in higher vertebrates.

*Aims*. Here we describe the identification of the Pekin duck PD-L2 orthologue (duPD-L2) and its gene structure.

*Methods*. The duPD-L2 cDNA was obtained by RT-PCR on RNA from splenocytes and primers based on duck genomic sequences. Nucleotide sequences of the 5′ and 3′ ends of the duPD-L2 ORF were determined by RACE. A homology model of duPD-L2 was obtained using the structure of mouse PD-L2 as a template. An eukaryotic expression vector was generated for expression of a C-terminally-His-tagged PD-L2 protein and culture supernatants of transfected 293T cells were assessed by immunoblot using an anti-his antibody.

*Results*. The duPD-L2 cDNA encodes a 321 amino acid protein that has an amino acid identity of 76% and 35% with chicken and human PD-L2, respectively. Mapping of the duPD-L2 cDNA with duck genomic sequences revealed an exonic structure of its coding sequence similar to those of other vertebrates. Homology modeling of the duPD-L2 extracellular domain was compatible with the extracellular domain structure of mouse PD-L2. Residues known to be important for receptor binding of mouse PD-L2 were mostly conserved in duPD-L2. DuPD-L2 mRNA was constitutively expressed in most tissues examined with highest expression levels in lung, spleen, bursa, cloaca, cecal tonsil and very low levels of expression in muscle, kidney and brain.

*Conclusions*. Our observations demonstrate evolutionary conservation of the exonic structure of its coding sequence, the extracellular domain structure and residues implicated in receptor binding but the role of the longer cytoplasmic tail in avian PD-L2 proteins remain to be determined.

*Funding Agencies: *CIHR

# Sub article: A87: Functional Impact of Colorectal Cancer-Associated Mutations in the Tyrosine Phosphatase SHP-2

**Authors:** J. Gagné Sansfaçon, M. Langlois, C. Qu, N. Rivard

**Affiliations:** ^87.1^Université de SherbrookeSherbrookeQCCanada; ^87.2^Aflac Cancer and Blood Disorders CenterAtlantaGAUSA; ^87.3^University of SherbrookeSherbrookeQCCanada

*Background*. Gain-of-function mutations of* PTPN11* gene (E76K, E76G, D61Y) were associated with pediatric leukemias (>30% of juvenile myelomonocytic leukemias) and certain solid carcinomas including colorectal cancer (CRC) (Bentires-Alj, Cancer Res 2004).* In vitro,* these specific mutations activate SHP-2 phosphatase activity and enhance its binding to signalling partners resulting in sustained activation of downstream effectors especially the RAS/MAPK pathway (Matozaki, Cancer Sci 2009). Dysregulation of this pathway is a common event in CRC. Indeed, activating mutations in* KRAS or BRAF* genes are found in up to 60% of colorectal tumors and are acquired at the very early premalignant stage.

*Aims*. The aim of this study was to investigate the functional impact of CRC-associated mutations in the tyrosine phosphatase Shp-2.

*Methods*. To determine the pathogenic effect of somatic mutation on E76 residue in intestinal epithelial cells (IECs)* in vivo*, we generated* Ptpn11* ^*E76Kneo/*+^/*Villin*-*Cre* ^+^ mice (*Shp*-2^*IEC*-*E*76*K*^ mice) by crossing* Ptpn11* ^*E76Kneo/*+^ mice with* Villin*-*Cre* mice.

We also crossed our model with Apc^*Min/+*^ mice, heterozygous for* Apc* truncation mutation frequently found in human sporadic CRC, and which spontaneously develop adenomas in the intestine (*Apc* ^Min/+^; *Shp*-2^*IEC*-*E*76*K*^).

*Results*. Our results show that mutant *Shp*-2^*IEC*-*E*76*K*^ mice exhibited similar body weight to control mice. However, colon and small intestine length and weight were significantly increased one month after birth and thereafter. Furthermore, Ki67 immunostaining revealed that there was a significant increase in the number of proliferating cells in mutant mice in comparison to control mice. Surprisingly, decreased number of Paneth cells was observed in *Shp*-2^*IEC*-*E*76*K*^ mice while Goblet cells were expanded. Additionally, reduced expression of total and active *β*-catenin protein was found in *Shp*-2^*IEC*-*E*76*K*^ IECs while MERK/ERK signalling was markedly activated. We then analyze the potential involvement of the *Shp*-2^*IEC*-*E*76*K*^ mutant in intestinal tumorigenesis. Notably, a major effect on intestinal tumor initiation was observed in *Apc* ^Min/+^; *Shp*-2^*IEC*-*E*76*K*^ mice compared to control littermate. The multiplicity of polyps with *Shp*-2^E76K^ expression was indeed increased in the small and large intestine. Increased ERK and NFkB activities were observed in polyp extracts from *Apc* ^Min/+^; *Shp*-2^*IEC*-*E*76*K*^ mice.

*Conclusions*. Therefore, these results demonstrate that CRC-associated SHP-2 mutations promote IEC proliferation and tumorigenesis probably through activation of RAS/MAPK signalling pathway.

*Funding Agencies: *Cancer Research Society; FRQS

# Sub article: A88: Exploring the Nature of Common Biological Roles between NCOR1 and Its Newly Identified Protein Interactor CHD8 in Colorectal Cancer Cells

**Authors:** A. Loiselle, S. St-Jean, F. Boisvert, F. Boudreau

**Affiliations:** ^88.1^Université de SherbrookeSherbrookeQCCanada

*Background*. NCOR1 (nuclear receptor corepressor 1) is a transcriptional corepressor that interacts with nuclear receptors. Using a quantitative protocol of SILAC (Stable Isotope Labeling In Cell Culture) immunoprecipitations combined with mass spectrometry, we previously identified CHD8 (Chromodomain helicase DNA binding 8) protein as a new interaction partner of NCOR1. However, the nature of CHD8 biological function in colorectal cancer (CRC) cell lines is currently unclearNCOR1 (nuclear receptor corepressor 1) is a transcriptional corepressor that interacts with nuclear receptors. Using a quantitative protocol of SILAC (Stable Isotope Labeling In Cell Culture) immunoprecipitations combined with mass spectrometry, we previously identified CHD8 (Chromodomain helicase DNA binding 8) protein as a new interaction partner of NCOR1. However, the nature of CHD8 biological function in colorectal cancer (CRC) cell lines is currently unclear.

*Aims*. To investigate the biological roles for CHD8 in CRC cells and to monitor biological complementary role(s) when compared to NCOR1 functions.

*Methods*. Caco-2/15 and HT-29 cells lines were depleted in NCOR1 or CHD8 by RNAi. Cell proliferation was measured by cell counting and senescence detected by measuring senescence associated secretory phenotype, SABeta-galactosidase assays and DNA damage. Tumour growth properties were assessed by xenografts in immunodeficient mice.

*Results*. CHD8 physical interaction with NCOR1 was validated by co-immunoprecipitations in various CRC cell lines. NCOR1 depletion in both Caco-2/15 and HT-29 led to a strong reduction in cell proliferation and induction of a senescence phenotype. In parallel, CHD8 depletion in these cells led to a partial reduction in cell proliferation without leading to the senescence phenotype. A similar observation was made in the context of xenografts obtained in immunodeficient mice with an intermediate decrease of tumour growth for HT-29 CHD8 depleted cells when compared to NCOR1 depleted cells. Finally, a closer analysis of the CHD8 protein expression profile among different cell lines identified a shorter CHD8 isoform in normal and CRC cells,(CHD8_S_) and the classical longer form predominantly expressed in CRC cells (CHD8_L_). Depletion of both short and long CHD8 forms in Caco-2/15 and HT-29 cells led to drastic growth arrest as observed for NCOR1 depletion assays.

*Conclusions*. CHD8 and NCOR1 are crucial regulators of cell proliferation in CRC cells. Functional characterization of the biological relevance of both CHD8 isoforms in comparison to NCOR1 is currently ongoing to better understand the functional roles of these interactions.

*Funding Agencies: *CIHR, NSERC

# Sub article: A89: HNF4A Plays Role in DNA Repair in Colorectal Cancer

**Authors:** S. Wilson, J. Babeu, F. Boudreau, F. Boisvert

**Affiliations:** ^89.1^Université de SherbrookeSherbrookeQCCanada

Not published at author's request.

*Funding Agencies: *CAG, CIHR

# Sub article: A90: Regulation of P2Y6 Receptor Expression by P53

**Authors:** C. Molle, M. Placet, G. Arguin, F. Gendron

**Affiliations:** ^90.1^Université de SherbrookeSherbrookeQCCanada

*Background*. Extracellular UDP selectively activates the G protein-coupled P2Y_6_ receptor (P2Y_6_R). In fact, extracellular nucleotides, such as UDP, are found in high concentration in the vicinity of colorectal tumours. However, the role of these nucleotides and associated receptor system is not fully understood. Previous results showed that* P2RY6* gene and protein expression is upregulated in cancerous intestinal epithelial cells (cIECs) harbouring P53 mutations. The P2Y_6_R isoform 1 is coded by different RNA variants that share an overlapping promoter region. More recently, we identified a new promoter region for the* P2RY6* gene that appears to code for P2Y_6_R isoform 2 for which the function is still unknown. In this context, the hypothesis is that* P2RY6* gene expression is regulated by epigenetic modifications caused by P53 mutations in cIECs, which could lead to the differential regulation of P2Y_6_R isoforms expression.

*Aims*. The aims are to: (1) determine if P53 or its R273H mutant isoform (P53^R273H^), a common mutant found in colorectal cancer, could regulate* P2RY6* gene expression and (2) characterize the epigenetic marks associated with* P2RY6* expression in cIECs.

*Methods*. We determined P2Y_6_R expression levels by qPCR in cIECs and receptor activity using intracellular calcium mobilization assays. We targeted two promoter regions on the 11q13.4-13.5 locus (NC_000011.10: 73,264,496,…, 73,298,625) that are coding for isoform 1 (R1 and R4: 73,262,844,…, 73,264,778) and isoform 2 (R4: 73,270,148,…, 73,272,365). We cloned these regions in the pGL4.10 luciferase-expressing vector and cotransfected these constructions with recombinant wild-type P53 (P53^wt^) or P53^R273H^ in HCT 116 cells prior to luciferase assays as previously described. The epigenetic modifications affecting* P2RY6* expression were determined by ChIP assays to measure the level of trimethylated lysine27 of histone H3 (H3K27me3) associated with the proximal promoter region.

*Results*. P2Y_6_R expression is upregulated in IECs harbouring a mutated* TP53* gene as compared to those having the wild-type isoform. Luciferase assays showed that P53 activates both R1 and R4 promoter regions, whereas the p53^R273H^ mutant could only induce the transcription of the R4 region. Hence, by increasing the quantity of p53^R273H^, we reduce the capacity of P53^wt^ to induced R1 transcription, which is dominant promoter region coding for P2Y_6_R isoform 1. This result suggests a form of competition between P53 and P53^R273H^. Finally, qPCR analyses confirmed that P53^wt and R273H^ expression increased the transcription of* P2RY6* gene.

*Conclusions*. The P53^R273H^ mutant seems associated to increase expression of P2Y_6_R isoform 2 for which the function is not yet identified but that are most likely linked to stimulation of cell proliferation and resistance to apoptosis.

*Funding Agencies: *CIHR

# Sub article: A91: Hedgehog Pathway and the Primary Cilium in Colorectal Cancer Cells

**Authors:** B. Senicourt, S. Boudjadi, N. Basora, J. Carrier, J. Beaulieu

**Affiliations:** ^91.1^Université de SherbrookeSherbrookeQCCanada

*Background*. The Hedgehog pathway is involved in the maintenance of numerous cell types both during development and in the adult. Often deregulated in cancers, its involvement in colorectal cancer has come into view during the last few years, although its role remains poorly defined. In most tissues, the Hedgehog pathway is highly connected to the primary cilium, an organelle not expressed in the normal colonic epithelium which recruits the functional components and regulates the pathway.

*Aims*. Since the intestinal epithelium is known to be a non-ciliated tissue, the HH pathway as related to the PC has not been explored. We investigated the presence of PC in colon cancer tumors and cell lines. We used cellular models to look at HH activation, focusing on the final effector Gli1. The link between PC and the HH pathway was shown by the recruitment of the Smo receptor in the PC.

*Methods*. We looked for PC in a subset of 63 tumors from a Tissue Micro Array and in 4 colorectal cell lines by immunofluorescence using two well-known markers, acetylated *α*-tubulin and polyglutamylated tubulin. 3D deconvoluted pictures were obtained to characterize the shape and size of PC. Using cellular models we investigated HH pathway expression by qPCR. We assessed the functional link between HH pathway and PC through localization of the Smo receptor in the PC using immunofluorescence.

*Results*. We observed the presence of the primary cilium in the epithelium of primary colorectal tumors at all stages but not in their normal counterparts. Using human colorectal cancer cell lines we found a clear correlation between the presence of the primary cilium and the expression of the final Hedgehog effector, GLI1, and provide evidence of a functional link between the two by demonstrating the recruitment of the SMO receptor to the primary cilium membrane.

*Conclusions*. We conclude that the primary cilium directly participates in the Hedgehog pathway in colorectal cancer cells.

*Funding Agencies: *CAG

# Sub article: A92: Prevalence of Colorectal Polyps in Liver Transplant Patients

**Authors:** A. Ma, A. Therrien, M. Bouin

**Affiliations:** ^92.1^Centre Hospitalier de l'Université de MontréalMontréalQCCanada

*Background*. Liver transplantation (LT) is a risk factor for the development of neoplasm, the third cause of mortality at one year following LT. Colorectal cancer (CRC) is particularly lethal in post transplant patients, with a survival of 30% at five years (compared to 63% in the general population). However, the risk of CRC and the risk of developing colorectal polyps post LT have not been prospectively studied.

*Aims*. The main objective of our study was to determine the prevalence of colorectal polyps post LT.

*Methods*. We undertook a prospective study of all LT recipients between January 1st, 2007 and December 31st, 2009 at our tertiary center. Were included patients who underwent a screening colonoscopy within 10 years before and 5 ± 2 years after LT. The demographic, medical and endoscopic data were extracted from charts. Patient subgroups were formed and compared based on the presence or not of polyps.

*Results*. Of 98 liver transplant recipients with a pre-LT colonoscopy, 50 patients underwent post-LT colonoscopy screening and were included (mean age 53.8 ± 7.6 years; 40% female). Twenty-four of the excluded patients had died before their follow-up colonoscopy.

In the pre-LT setting, colorectal polyps were found in 40% of patients. The most common histological findings were hyperplastic polyps (40%) and adenomas (25%). The histology results were unavailable in the rest of cases.

In the post-LT setting, the colonoscopy was performed on average at 4.8 ± 1.6 years and polyps were found in 28% of patients. The histological findings were adenomas in 42.8% and hyperplasia in 28.6% of cases (not significantly different from the pre-LT findings). There was one case of neuroendocrine tumor but none of CRC. The histology results were unavailable in the rest of cases.

Furthermore, the presence of pre-LT polyps was not predictive of developing post-LT polyps (25% of patients with pre-LT polyps versus 30% without developed post-LT polyps; *p* = 0.7).

Patients with post-LT adenomas were not significantly older than those without (56.3 years versus 53.4%; *p* = 0.38). There was a trend towards greater risk of developing post-LT polyps in men (36.7% versus 15% in women; *p* = 0.09).

*Conclusions*. In this prospective study, the prevalence of colorectal polyps at 4.8 ± 1.6 years post-LT is high (28%). Polyps were adenomatous in almost half of the cases. A trend exists between the male gender and the risk of developing polyps. This study suggests that post-LT colonoscopy surveillance may be of benefit for CRC prevention.

*Funding Agencies: *None

# Sub article: A93: Improved Sample Quality Obtained by EUS-Guided Sink Compared to FNA for Foregut Subepithelial Lesions

**Authors:** M. Boulos, D. Wang, P. James, T. Moyana, A. Chatterjee

**Affiliations:** ^93.1^University of OttawaOttawaONCanada

*Background*. Gastric subepithelial lesions (SELs) can be divided into three major categories, namely smooth muscle tumors (leiomyomas and leiomyosarcomas), neurogenic tumors (schwannomas and neurofibromas) and gastrointestinal stromal tumors (GIST). GIST are the most common type of foregut SEL and carry an important malignant potential. Small SELs (<2 cm) have been notoriously difficult to sample endoscopically. Endoscopic ultrasound (EUS)-guided single incision needle knife (SINK) biopsy has become increasingly used for deep tissue sampling of foregut SELs, however there exists limited evidence to suggest that this results in superior specimen acquisition.

*Aims*. We sought to review our experience regarding the difference in sample quality of SELs obtained by EUS-guided SINK compared to EUS-guided fine needle aspiration (FNA).

*Methods*. We performed a retrospective chart review of EUS-guided SINK cases performed at The Ottawa Hospital for the evaluation of foregut SELs. These samples were compared to consecutive EUS-guided FNA samples obtained over a similar time period. Two pathologists reviewed the specimens blindly and independently. The quality of each sample was determined based on a 5-point scale, where poor = 1, adequate = 2, good = 3, very good = 4 and excellent = 5.

*Results*. 13 patients with foregut SELs were sampled by SINK and these were compared to 26 consecutive EUS-guided FNA samples. 12 out of the 13 (92%) SINK cases were reported to be of excellent quality (5/5) whereas one case was of adequate quality (2/5). The median FNA quality score was 3 with an interquartile range of 2–5, which was found to be significantly inferior to SINK (*p* < 0.01). 8 SINK cases (62%) were reported to have a cellularity of ≥5 000. Only 4 EUS-guided FNA specimens (15%) were reported to have a cellularity of ≥5 000.

*Conclusions*. The sample quality of subepithelial lesions obtained by EUS-guided SINK may be superior to EUS-guided FNA.

*Funding Agencies: *None

# Sub article: A94: Topical Hemostatic Spray for the Management of Malignancy-Related Gastrointestinal Bleeding: A Systematic Review and Meta-Analysis

**Authors:** M. Sandhu, P. James, S. Piscopo

**Affiliations:** ^94.1^The University of OttawaOttawaONCanada

*Background*. Hemostatic powder spray agents (HPSAs) have been shown to be effective for gastrointestinal haemorrhage (GH), however their role as first-line agents is limited. Conventional endoscopic methods often fail to achieve hemostasis in cases of malignancy-related GH due to lesion location, lesion distribution and altered tissue responses secondary to the malignant process, anticoagulation and/or chemoradiation treatment. The ability of HPSAs to treat large surface areas without touching tissue render them ideal for the management of malignancy-related GH, however their role in this setting remains unclear.

*Aims*. To review the literature on the efficacy of HPSAs in malignancy-related GH.

*Methods*. We performed a systematic search of EMBASE and MEDLINE through June 2015 for studies reporting the use of HPSAs for malignancy-related GH. Duplicate articles and case reports were excluded. The primary outcome was hemostasis at 72 hours post-treatment. A pooled estimate was calculated using random effects models. The methodological quality of the included studies was assessed using the Newcastle-Ottawa scale.

*Results*. Of the 1,704 citations identified, a full-text review was performed on 89 and 8 were included in the meta-analysis (44 patients). Four different HPSAs were identified: Hemospray®, cyanoacrylate spray, Costasis®, and Endoclot®. The most commonly used spray in these patients was Hemospray (5 studies). Five studies included less than 5 patients. Nine studies scored 7 out of 9 and one study scored 6 out of 9 by using the Newcastle-Ottawa Quality Assessment Scale. Immediate hemostasis was achieved in all cases. Meta-analysis showed that treatment with HPSAs resulted in hemostasis for up to 72 hours in 90% of cases (95% confidence interval 0.67–0.99).

*Conclusions*. The limited evidence to date suggests that topical hemostatic sprays are effective in the setting of malignancy-related GH. Larger prospective studies are required.

*Funding Agencies: *None

# Sub article: A95: A Comparison of Fecal Immunochemical Testing to Guaiac-Based Fecal Occult Blood Testing for the Detection of Colorectal Adenomas and Cancer

**Authors:** L. Du, C. Teshima, B. Moysey, A. Morse, R. Sultanian

**Affiliations:** ^95.1^University of AlbertaEdmontonABCanada; ^95.2^University of TorontoTorontoONCanada

*Background*. Programmatic colorectal cancer (CRC) screening was initiated in Edmonton in 2011 as the SCOPE program, offering colonoscopy to patients with above average CRC risk, including positive guaiac-based fecal occult blood tests (gFOBT), as well as a personal or family history of CRC. The newly developed fecal immunochemical test (FIT) has demonstrated improved sensitivity without the loss of specificity for detection of advanced adenomas and CRC. In November 2013, FIT replaced gFOBT as the first-line CRC screening test in Alberta. The goal of our study was to assess the impact of the transition from gFOBT to FIT on a screening colonoscopy program.

*Aims*. To compare the detection rates of polyps, adenomas, advanced adenomas, and CRC of gFOBT to those of FIT in patients selected for colonoscopy through a region-wide CRC screening program.

*Methods*. A retrospective cohort analysis was performed using a prospectively maintained database of all patients, aged 50–74, who underwent colonoscopy in the SCOPE program between January 1, 2013 and December 31, 2014 as a result of a positive gFOBT or a positive FIT. Patients with morbid obesity, significant co-morbidities, or overt gastrointestinal symptoms were excluded. Colonoscopy was offered to patients with at least one out of three samples positive using the guaiac-based Hemoccult II® SENSA in 2013 or with a positive FIT, defined as ≥75 micrograms/gram of stool, using the Polymedco OC FIT-CHEK® in 2014. All procedures were performed during dedicated SCOPE endoscopy time and by endoscopists certified by the program.

*Results*. 633 patients underwent colonoscopy due to a positive gFOBT in 2013, and 2137 patients underwent colonoscopy due to a positive FIT in 2014, values that represent a substantial increase in the number of patients referred for colonoscopy due to a positive screening test between the two years. Patients who were FIT-positive had significantly higher polyp detection rates (PDR) (71% versus 63%; *p* < 0.001) and adenoma detection rates (ADR) (62% versus 48%; *p* < 0.001) in comparison to gFOBT-positive patients. CRC detection was significantly higher in gFOBT-positive patients in comparison to patients who were FIT-positive (5.6% versus 3.1%; *p* < 0.001).

*Conclusions*. The conversion of the programmatic CRC screening in Edmonton from gFOBT to FIT-based selection of patients resulted in significantly increased CRC screening rates, increased referral numbers, as well as higher PDR and ADR. FIT resulted in a lower CRC detection rate percentage although the total number of cases detected was higher than the FOBT-positive group due to increased FIT utilization.

*Funding Agencies: *None

# Sub article: A96: A Diagnostic Dilemma: A Case of Cholestatic Jaundice due to Al-Amyloidosis

**Authors:** R. Al-Dabbagh, S. Bharadwaj, S. Patterson, M. Puglia

**Affiliations:** ^96.1^McMaster UniversityHamiltonONCanada

*Background*. Amyloidosis is a rare, infiltrative condition associated with extracellular deposition of fibrils that can lead to end organ dysfunction. Most often, patients with primary amyloidosis present with cardiac or renal involvement. If the liver is involved, it usually as asymptomatic hepatomegaly. Furthermore, serious liver dysfunction, with initial presentation of cholestatic jaundice is very rare, accounting for less than 5% of amyloidosis.

*Aims*. We present a case of cholestatic jaundice due to amyloidosis with unclear concurrent multiple myeloma.

*Methods*. A full chart review of the case was undertaken, including assessment of radiographic, biochemical and biopsy Results. A subsequent literature review of the topic was also conducted.

*Results*. A 69 year old male initially presented with a 3-4 month history of right upper quadrant abdominal pain. He also reported reduced oral intake and an associated weight loss of 25 pounds. However, he denied fevers, night sweats, rashes, and review of systems was otherwise unremarkable. Physical examination was prominent for scleral icterus, right upper quadrant tenderness, nonpulsatile hepatomegaly, and peripheral edema. Laboratory investigations revealed hemoglobin of 121 g/L (MCV 96.0 fL), creatinine of 103 *μ*mol/L, total bilirubin of 82 *μ*mol/L (conjugated 59.6 *μ*mol/L), albumin of 21 g/L, gamma-glutamyl transpeptidase of 1773 U/L, alkaline phosphatase of 692 U/L, alanine transaminase 45 U/L, aspartate transaminase of 97 U/L, and INR of 1.1. Additionally, abdominal ultrasonography revealed a liver span of 20 cm, with diffuse fatty infiltration, spleen of 12 cm in size, and normal caliber and patency of the portal vein and common bile duct. A subsequent CAT scan of the chest, abdomen and pelvis, and MRCP were also unremarkable. His hospital course was complicated by worsening laboratory abnormalities, including worsening hyperbilirubinemia (conjugated 247 *μ*mol/L), INR (1.8), acute kidney injury (creatinine 314 *μ*mol/L), and nephrotic range proteinuria. Due to suspicion of amyloidosis in the setting of multi-organ failure, serum electrophoresis was done which revealed free kappa of 645.46 mg/L and free lambda of 38.21 mg/L. Finally, liver biopsy was performed, showing severe amyloidosis occupying the sinusoids, spaces of Disse, portal connective tissue and walls of vessels, with compression of hepatocytes. Congo red staining showed green birefringence. He was started on dexamethasone, but further chemotherapy had been withheld until further characterization can be made of possible concurrent multiple myeloma.

*Conclusions*. Cholestatic jaundice is common, but is rarely the initial presentation of amyloidosis. If initial investigations rule out any obvious etiology, suspicion for infiltrative diseases, such as amyloidosis, should be raised.

*Funding Agencies: *None

# Sub article: A97: Should Anticoagulation Be Offered in Patients with PVT in the Setting of HCC?

**Authors:** T. Mahmoudi, A. Kayal, R. Carvalho, A. Weiss

**Affiliations:** ^97.1^UBCVancouverBCCanada

*Background*. Portal vein thrombosis (PVT) is a seen in about 20–44% of patients with hepatocellular carcinoma (HCC). To our knowledge, no other study has looked at the need for anticoagulation in patients with HCC and PVT.

*Aims*. The aim of this study is to investigate the natural history and progression of portal vein thrombosis in patients with hepatocellular carcinoma with or without anticoagulation therapy.

*Methods*. Using the British Columbia Cancer Agency database, a cohort of 54 patients who were diagnosed with both conditions were evaluated retrospectively. Nine patients were excluded secondary to lack of follow up. HCC and PVT diagnosis and follow up was made with contrast enhanced CT or MRI. Most patients received a single or a combination of the following treatments: transarterial chemoembolization, radiofrequency ablation or surgical resection. Thirty five (78%) patients received systemic therapy with Sorafenib.

*Results*. Thirty eight patients were males and mean age was 62.8. Liver disease etiology was HCV in 19 (42%), HBV in 18 (40%), ETOH in 5 (11%) and hemochromatosis in 1 (2%). Results: Average survival after HCC diagnosis was 28 months and 15 months after PVT diagnosis. Among the 45 patients evaluated, 8 patients received anticoagulation while 39 did not. PVT progression occurred in 19 (49%) of the non anticoagulated group, and 4 (67%) of the anticoagulated group. Right portal vein involvement was seen in 18 (40%) patients with progression in 67% of the time, Left PVT in 13 (28%) with a progression in 7(54%), and main PVT 6 (13%) with a progression in (67%). In 1 case, PVT progressed from the main PVT to Superior mesenteric vein (SMV) and from the LPV to SMV in 2 other cases. No symptoms directly related to PVT development were reported.

*Conclusions*. The possible anticoagulation related complications need to be considered before attempting therapy in patients with HCC and PVT. Despite the small number of patients included in this study, this review shows that PVT progression in patients with HCC and the absence of clinical complications is similar in both anticoagulated and non anticoagulated groups. Thus, the usefulness of anticoagulation in this patient population needs to be further studied.

*Funding Agencies: *None

# Sub article: A98: Litterature Review of the Economic Impact of Treatments for Inflammatory Bowel Diseases (The Igenomed Consortium)

**Authors:** J. Lachaine, A. Bitton, A. Miron, D. Nait Ladjemil

**Affiliations:** ^98.1^University of MontrealMontrealQCCanada; ^98.2^McGill UniversityMontrealQCCanada

*Background*. The last decade witnessed great advances in the treatment of inflammatory bowel diseases (IBD) with the introduction of biologic therapies. Several economic evaluations have been run to evaluate these treatments.

*Aims*. The goal of this study was to analyse the existing evidences and key parameters included in IBD cost-effectiveness studies.

*Methods*. A systematic literature review was conducted to identify economic evaluations of IBD therapy. Electronic databases (Embase and Medline) were used to identify full economic evaluations published from 2004 to 2015. Cross-references of selected articles and gray literature search were also performed to find additional publications. The health outcomes, costs, incremental cost-effectiveness (ICERs) and cost-utility ratios (ICURs) were analysed.

*Results*. The literature review allowed identifying 3,631 potentially relevant studies. Titles and abstracts screening allowed the selection of 53 articles. After assessment of those articles, 36 were found relevant for the review. Four other studies were added from gray literature. Different treatments were evaluated including biologics (53%), mesalamine (28%), biologics and immunosuppressants combination (5%) and immunosuppressants alone (3%). Infliximab was the most common biologic treatment evaluated (65% of all biologics). In the cost-utility analyses (CUA) (88%), 35% had utility scores derived from IBD severity scores. The remaining studies used direct and indirect utility measurement methods, including EQ-5D (43%), standard gamble (33%), time trade off (25%) and visual analog scale (8%). Markov modeling, decision tree or a combination of both were used in 38%, 38% and 5% of the studies respectively. All studies included drug acquisition costs, 50% included treatment administration costs, 65% included hospitalization costs and 45% included surgical costs. In CUA, the main outcome measures were ICURs ranging from dominant (less costly and more effective) to USD2,757,857/QALY (CAD2,955,814/QALY). More specifically, treatment under investigation was dominant in 34% of the analyses. ICURs were bellow CAD50,000/QALY in 57% of cases and bellow a threshold of CAD100,000/QALY in 71% of cases.

*Conclusions*. Several economic evaluations especially involving biologics were conducted in the past decade. This study showed that there is some homogeneity in the selection of key parameters, such as the use of Markov modeling and decision tree in model development, use of EQ-5D utility measurements and costs included.

*Funding Agencies: *Genome Canada

# Sub article: A99: Hepatocellular Carcinoma in a Patient with Crohn's Disease on Azathioprine

**Authors:** V. Heron, K. Fortinsky, G. Spiegle, N. Hilzenrat, A. Szilagyi

**Affiliations:** ^99.1^McGill UniversityMontréalQCCanada; ^99.2^University of TorontoTorontoONCanada; ^99.3^Jewish General HospitalMontrealQCCanada

*Background*. Hepatocellular carcinoma (HCC) rarely occurs in patients without underlying liver disease. While Crohn's disease (CD) has been linked to certain forms of liver disease, HCC in these patients is rare.

*Aims*. We report the 12th case of HCC in a non-cirrhotic patient with CD and discuss the possible role of azathioprine.

*Methods*. Case report.

*Results*. A 61-year-old woman with CD was found to have elevated liver enzymes on routine blood work. Past medical history includes type 2 diabetes, dyslipidemia, and a family history of hemochromatosis in her father. She has a remote smoking history and no alcohol or drug use. Her CD was controlled with azathioprine for the past 8 years. She had previously been treated with 5-ASA, infliximab and multiple bowel resections.

Blood work revealed a mild asymptomatic transaminitis with normal liver function (lab results in Table 16 [Table tab17] [Table tab18]). A liver mass was identified on both abdominal ultrasound and CT scan (Figure 12 [Fig fig13]). Abdominal MRI confirmed a 3 cm lesion in segment 7. Liver biopsy showed well-differentiated HCC. Biopsies from non-neoplastic liver showed 30% macrovesicular steatosis without steatohepatitis, and minimal iron overload (grade 0-1/4).

The patient underwent tumor resection. Surgical pathology revealed no malignant cells despite initial biopsy showing HCC. The pathologist believes the tumor may have infarcted post biopsy. Azathioprine was stopped prior to surgery. There is no evidence of recurrence on imaging at 5-month follow-up.

This is the 12th reported case of HCC in a patient with CD in the absence of cirrhosis. Azathioprine was reported in 9 of the previous cases and has been proposed as a potential trigger given its known association with malignancies and its hepatotoxicity, especially in the setting of toxic levels of 6-MMP. Azathioprine may impair the immune system's ability to correct dysplasia caused by inflammation related to CD.

This is the 3rd reported case of biopsy proven HCC with complete histologic spontaneous regression. This may be explained by the withdrawal of azathioprine or by the liver biopsy as operative trauma, including biopsy, has been linked to spontaneous regression.

*Conclusions*. Though further studies are needed, the relationship between azathioprine and HCC remains concerning. Imaging may be considered in CD patients with abnormal liver enzymes, especially those on azathioprine with elevated drug levels.

*Funding Agencies: *None

# Sub article: A100: *Giardia Dduodenalis*-Induced Goblet Cell Mucin Depletion Is Cysteine Protease-Dependent

**Authors:** C. Amat, J. Motta, K. Chadee, A. Buret

**Affiliations:** ^100.1^University of CalgaryCalgaryABCanada

*Background*.* Giardia duodenalis *(syn.* G*.* lamblia*,* G*.* intestinalis*) is a cosmopolitan diarrheagenic parasite of the small intestine. Giardiasis leads to post-infectious irritable bowel syndrome (PI-IBS) and extra-intestinal complications via mechanisms that remain unclear. The mucus layers of the small and large intestines are protective against enteric infections, but we have hypothesized that* Giardia* may disrupt this barrier by two mechanisms: (i) by direct degradation of mucin proteins and (ii) by causing mucin depletion via hypersecretion in goblet cells. These effects could lead to both acute and chronic disease.

*Aims*. The aims of this study are to characterize how the enteropathogen* Giardia duodenalis* interacts with host mucus and assess its effects on the primary constituent of the mucus layer, mucin-2 (MUC2).

*Methods*. C57BL/6 wild-type (WT) mice and Muc2^−/−^ (KO) mice were gavaged with* Giardia* trophozoites (Assemblage B, strain GS/M). Mice were weighed daily and samples collected on day 7, at the peak of infection. Trophozoites were counted in the small intestine, and the small intestine and colon were processed for histological staining. The liver and spleen were collected aseptically and incubated on Columbia blood agar (aerobically and anaerobically) to assess bacterial translocation.* In vitro*, secreted products obtained from* Giardia* trophozoites (Assemblage A, strain NF) were co-incubated with purified human mucin. Co-incubation products were evaluated for MUC2 protein concentration by western blotting.* Giardia* trophozoites were co-incubated with human colonic goblet cells LS174T in the presence and absence of E-64, a broad spectrum irreversible cysteine protease inhibitor. Staining intensity of MUC2 was normalized to cell count and quantified by immunofluorescence compared to control.

*Results*. Infected KO mice had higher parasitic loads and failed to gain weight compared to WT mice. Goblet cell mucin was depleted in the small intestine and colon of infected WT mice. Infected mice showed increased bacterial translocation of aerobic and anaerobic species into the liver and spleen. Staining intensity of MUC2 was reduced inside the human goblet cells* in vitro* after exposure to* Giardia* trophozoites. These effects were inhibited by a cysteine protease inhibitor. In addition,* Giardia*'s secreted products degraded human purified MUC2* in vitro*.

*Conclusions*. Mucus protects the host against parasite accumulation and* Giardia*-induced weight loss.* Giardia* disrupts the mucus barrier by degrading MUC2 and causing hypersecretion leading to mucin depletion in the small intestine and colon. These effects are associated with increased bacterial translocation and may contribute to both acute and chronic disease. Mucin depletion is inhibited by a cysteine protease inhibitor, indicating a potential mechanism for hypersecretion.

*Funding Agencies: *NSERC CREATE Host-Parasite Interactions

# Sub article: A101: Temporal Deletion of IEC-Specific* Hdac1* and* Hdac2* Modifies Gut Homeostasis

**Authors:** A. Gonneaud, N. Turgeon, C. Jones, F. Boudreau, C. Asselin

**Affiliations:** ^101.1^Université de SherbrookeSherbrookeQCCanada

*Background*. Hdac1 and Hdac2 deacetylase activities regulate gene expression by controlling the acetylation levels of epigenetic histone marks and of many regulatory proteins. We have observed that* villin*Cre-mediated intestinal epithelial cell (IEC)-specific* Hdac1* and* Hdac2 *deletion from day E15 alters cell differentiation and proliferation, resulting in chronic inflammation. In addition, different levels of* Hdac1* and* Hdac2* expression lead to incremental modifications in IEC determination and intestinal homeostasis. These effects could be in part due to compensatory mechanisms established during murine development after day E15.

*Aims*. Considering the fact that the intestinal environment is crucial to maintain gut homeostasis, we hypothesized that IEC-specific inducible deletion of* Hdac1* and* Hdac2* in adult mice could result in different alterations on intestinal homeostasis. We also speculated that Hdac1 and Hdac2 would alter intrinsic IEC physiology in enteroid cultures.

*Methods*. To determine the effect of IEC-specific* Hdac1* and* Hdac2* deletion in adult mice, floxed* Hdac1* and* Hdac2* mice were crossed with* villin*Cre^ER^ mice to obtain* Hdac1* ^−/−^/*Hdac2* ^−/−^ mice after Tamoxifen injection of two-month-old mice. Small intestinal sections were stained with hematoxylin and eosin, and with Periodic Acid Schiff or lysozyme antibodies to detect respectively goblet and Paneth cells. PCNA antibodies were used to assess cell proliferation. IEC-intrinsic effect of Hdac1 and Hdac2 was determined in enteroid cultures. Jejunal* villin*Cre^ER^ *Hdac1* and* Hdac2* crypt enteroid cultures were treated with hydroxytamoxifen to induce gene deletion. Enteroid structure was observed by microscopy.

*Results*. Loss of* Hdac1* and* Hdac2* in the adult intestinal epithelium led to (1) jejunal tissue architecture defects and (2) goblet cell number decreases. In contrast to dual* villin*-Cre* Hdac1* and* Hdac2* deleted mice,* villin*Cre^ER^ mice did not display significant differences in proliferation as well as in Paneth cell numbers. Inducible deletion of both* Hdac1* and* Hdac2* resulted in the formation of enteroids with spheroid structures, suggesting alterations in cell growth and/or determination.

*Conclusions*. IEC-specific Hdac1 and Hdac2 regulate differently IEC and may alter IEC responses to the mucosal environment, leading to disturbances of intestinal homeostasis. HDAC inhibitors, which reduce intestinal inflammation severity and symptoms in mice, may well target IEC in addition to immune cells, thus controlling inflammatory responses.

*Funding Agencies: *CCC, CIHR

# Sub article: A102: Anti-Tissue Transglutaminase Normalization Post Diagnosis in Children with Celiac Disease

**Authors:** D. Migliarese Isaac, S. Rajani, J. Turner

**Affiliations:** ^102.1^Department of PediatricsUniversity of AlbertaEdmontonABCanada; ^102.2^Department of Pediatric Gastroenterology & NutritionUniversity of AlbertaEdmontonABCanada

*Background*. Celiac disease (CD) is a common autoimmune enteropathy to gluten, leading to intestinal inflammation, villous atrophy, and malabsorption. Screening for CD screening involves anti-tissue transglutaminase (atTG) IgA levels, followed by intestinal biopsy for confirmation. A gluten-free diet (GFD) is required to alleviate symptoms, normalize atTG, and heal the intestinal mucosa in CD patients. Monitoring in CD includes following atTG titers post diagnosis. Variability exists in the literature regarding time to atTG normalization, with no studies examining the trend and predictors of atTG over time in the pediatric CD population.

*Aims*. We aimed to evaluate time to normalization of atTG in children post CD diagnosis, and examine factors impacting this time.

*Methods*. A retrospective chart review was completed to evaluate the rate of atTG normalization in pediatric CD patients attending the Stollery Pediatric Celiac Clinic from 2007 to 2014. Clinical predictors that may impact time to resolution were examined, including: initial atTG, GFD compliance (GFDC), age at diagnosis, gender, ethnicity, presenting symptoms, Marsh score at diagnosis, medical comorbidities, and family history of CD. Multivariate binary logistic regression was utilized to determine independent predictors of atTG normalization. Cox hazard regression analysis was then undertaken to determine predictors of time to normalization.

*Results*. 339 patients met the inclusion criteria. Mean age was 9 years at diagnosis (range 1–17 years), with 64% females. Patients were followed for 6 months to 6 years. 83% of patients normalized atTG levels within the study time period. Mean time to normalization was 484 days for all patients (range 30 to 2084 days), and 460 days for GFD compliant patients (range 81 to 1809 days). Type 1 diabetes melllitus (T1DM) and South Asian ethnicity were independent predictors of failure to normalize atTG (OR = 0.23, *p* = 0.002; OR = 0.41, *p* = 0.031 resp.), with T1DM patients being 4 times less likely to normalize. Conversely GFDC was a positive predictor of atTG normalization (OR = 7.0, *p* < 0.001). Cox hazard regression demonstrated T1DM (HR = 0.5, *p* = 0.006) and South Asian ethnicity (HR = 0.67, *p* = 0.034) were predictors of longer time to atTG normalization. Patients with T1DM normalized atTG levels on average 240 days longer than those without.

*Conclusions*. There is a wide variation of rate and time to atTG normalization in children with CD. GFDC is the strongest predictor of early normalization. Patients with T1DM and South Asians are less likely to normalize atTG levels, and have longer time to normalization. This highlights the need for closer attention and education for these high-risk populations.

*Funding Agencies: *Women and Children's Health Research Institute Resident Trainee Research Grant

# Sub article: A103: The Effect of Sham Feeding on Small Bowel Transit Time in Patients Undergoing Capsule Endoscopy: An Interim Analysis

**Authors:** G. Ou, D. Prichard, C. Galorport, R. Enns

**Affiliations:** ^103.1^University of BCVancouverBCCanada; ^103.2^Mayo ClinicRochesterMNUSA

*Background*. Capsule endoscopy (CE) does not completely visualize the small bowel approximately 16.5% of the time due to limited battery life. We hypothesize that bacon-chewing is a potent stimulus of cephalic response, which may reduce capsule transit times and improve complete examination rate (CER).

*Aims*. To determine if chewing bacon improves CE transit times and CER.

*Methods*. A prospective, single-blinded, randomized controlled trial is in process at St. Paul's Hospital in Vancouver, BC. Inclusion: outpatient CE, age ≥ 19. Exclusion criteria: inpatient, use of motility-enhancing or slowing drugs, active bowel obstruction, bowel resection, dysphagia, diabetes with end-organ damage, untreated hypo-/hyperthyroidism, and endoscopic placement of capsule. Given Imaging PillCam SB3™ (Yoqneam, Israel) were used.

Randomization took place via concealed allocation per randomization sequence generated prior to study initiation. All subjects underwent bowel preparation (2 L) the day before, and fasted ≥2 h prior to the procedure. Clear fluids and normal diet were allowed 2 h and 4 h post-capsule ingestion, respectively. Immediately after swallowing the capsule, subjects in the bacon group were asked to chew 10 pieces of bacon, each over 30 seconds at one-minute intervals. This process was repeated an hour after ingesting the capsule. Gastric transit time (GTT), small bowel transit time (SBTT), and CER were compared between the groups.

This study was approved by institutional research ethics board and registered on clinicaltrials.gov (NCT02353208).

*Results*. From 01/2015 to 08/2015, 46 CE's were included in the study, 42 of which were for GI bleeding, 2 for Crohn's disease, 1 for abnormal CT finding, and 1 for recurrent intussusception. Female comprised 61% (*n* = 28) of the cohort. Mean age was 59.3 ± 15.9 years. CE did not pass the pylorus in 2 patients (bacon group) during the recording, but did subsequently pass through the bowel; these were excluded from further analysis. Otherwise, there were no significant differences in GTT, SBTT, and CER.

*Conclusions*. The interim analysis does not demonstrate any change in capsule transit/completion with chewing bacon. The study will continue recruitment until the goal sample size of 122.

*Funding Agencies: *None

# Sub article: A104: Mycophenolate Mofetil-Induced Immunosuppression Is Associated with a Wasting Phenotype, Altered Expression of Colonic Inflammatory Mediators and Reductioned Intestinal Microbial Diversity—Dissecting the Mechanisms of Gastrointestinal Dysfunction Triggered by Anti-Rejection Drugs

**Authors:** S. Pereira, A. Moffat, L. Alston, K. Rioux, P. Beck, M. Workentine, S. Greenway, S. Hirota

**Affiliations:** ^104.1^University of CalgaryCalgaryABCanada

*Background*. Mycophenolate mofetil (MMF) is used post-transplant to prevent allograft rejection. In some cases, MMF is associated with GI complications and a wasting phenotype that necessitates treatment cessation.

*Aims*. To explore the mechanisms responsible for these issues, we developed and characterized a mouse model of MMF-induced immunosuppression.

*Methods*. Medicated feed (0.563% MMF) was administered to C57/Bl6 mice* ad libitum* for 9 days. In some experiments, mice were then removed from MMF treatment, returned to control chow and then sacrificed 7 days later. Intestinal tissues were removed for histological assessment, TUNEL staining (to assess apoptosis) and inflammatory mediator expression (via Luminex). Fecal pellets were collected throughout the course of the experiment and microbial composition assessed.

*Results*. Within 9 days of MMF treatment, we observed significant reductions in body weight, colon length, cecum weight, spleen weight and hematocrit. Upon cessation of MMF treatment, these parameters reversed to normal but splenomegaly developed. No differences in TUNEL staining were observed. Colonic tissue isolated at day 9 of treatment exhibited increased expression of inflammatory mediators including G-CSF, IFN*γ*, IL-6, IL-7, IL-10, IL-13, KC, LIF, MCP-1, MIP-1B, MIP-2, TNF*α* and VEGF. In contrast, IL-4 and IL-5 were significantly reduced. Characterization of the fecal microbiome revealed progressive alterations of bacterial populations correlating with duration of drug exposure with significant changes in composition accompanied by loss of diversity. Furthermore, phyla analysis revealed an expansion of Proteobacteria and Firmicutes, and a contraction of Bacteroidetes, in MMF-treated mice.

*Conclusions*. MMF treatment recapitulates human clinical symptoms in the mouse. The observed symptoms are associated with alterations in the intestinal immune status and reductions in intestinal microbial diversity. We will continue to characterize this model to better understand the mechanisms responsible for MMF-related complications. Furthermore, we intend to assess microbe-targeting interventions in future studies.

*Funding Agencies: *Canadian Foundation for Innovation; The Dr. Lloyd Sutherland Investigatorship in IBD/GI Research

# Sub article: A105: Prediction of Esophageal and Gastric Histology by Macroscopic Diagnosis during Upper Endoscopy in Pediatric Celiac Disease

**Authors:** E. Boschee, J. Yap, J. Turner

**Affiliations:** ^105.1^University of AlbertaEdmontonABCanada; ^105.2^Stollery Children's HospitalEdmontonABCanada

*Background*. Celiac disease (CD) is the most common autoimmune enteropathy in children. Recent guidelines support diagnosis without biopsy in select pediatric patients, yet concerns exist over the potential for missing alternate tissue diagnoses. The frequency of endoscopic and histological abnormalities in intestinal sites other than duodenum in pediatric CD has yet to be studied.

*Aims*. The aim of the study was to determine the sensitivity of macroscopic appearance for predicting histology diagnosis at sites other than the duodenum. It was hypothesized that normal esophageal and gastric histology can be predicted by normal endoscopic appearance, obviating the need for routine biopsy from these sites.

*Methods*. Endoscopic and biopsy findings in patients diagnosed with CD at Stollery Children's Hospital from 2010–2012 were retrospectively reviewed. The primary outcome was the diagnostic performance of endoscopic findings in predicting normal esophageal and gastric histology. A secondary outcome was the prevalence of other esophageal and gastric diagnoses.

*Results*. A total of 140 patients were included (61.4% female). The mean age at biopsy was 9.1 years, and the mean aTTG was 393.9. Endoscopic appearance was reported as normal in the esophagus and stomach in 84.8% and 87.7% of patients, respectively. Abnormal endoscopic esophageal diagnoses included eosinophilic esophagitis (EE) (5.8%), esophagitis (5.1%), glycogenic acanthosis (1.4%) and non-specific abnormalities (2.9%). Endoscopic gastric abnormalities were gastritis (6.5%), pancreatic rest (0.7%) and non-specific abnormalities (5.1%). Esophageal and gastric biopsies were taken in 54.3% and 77.9% of patients, respectively. Histology was normal in 77.6% of esophageal and 87.2% of stomach specimens. Abnormal esophageal histology included EE (10.5%), esophagitis (9.2%), glycogenic acanthosis (1.3%) and non-specific abnormalities (1.3%). Gastritis was reported in 12.8% of gastric specimens.

The sensitivity and specificity of normal macroscopic diagnosis for predicting normal esophageal histology was 86.2% and 61.1%, and for normal gastric histology was 88.3% and 26.7%. The positive predictive value of endoscopic diagnosis for predicting normal histology was 87.7% in the esophagus and 88.3% in the stomach.

*Conclusions*. This study suggests that, in the absence of macroscopic abnormalities, routine esophageal and gastric biopsy during endoscopy for pediatric CD is not needed. Endoscopic diagnosis is sufficiently sensitive to predict normal histology. These findings have cost and time saving applications for current clinical practice.

*Funding Agencies: *Women and Children's Health Research Institute (WCHRI)

# Sub article: A106: Inpatient Capsule Endoscopy Guides the Need for Device Assisted Enteroscopy in Patients with Suspected Small Bowel Bleeding

**Authors:** M. Alsahafi, P. Cramer, N. Chatur, F. Donnellan

**Affiliations:** ^106.1^Division of GastroenterologyVancouver General HospitalUniversity of British ColumbiaVancouverBCCanada

*Background*. The availability of capsule endoscopy (CE) and device assisted enteroscopy has remarkably changed the management of patients with suspected small bowel bleeding. Device assisted enteroscopy is invasive, time consuming and associated with high health care cost. CE has several advantages including the non-invasive nature and the high diagnostic yield and as such it could be utilized to guide further intervention.

*Aims*. To assess the utility of inpatient CE to guide management and selection for device assisted enteroscopy in patients with suspected small bowel bleeding.

*Methods*. We retrospectively reviewed the hospital database for all patients requiring an inpatient CE between November 2011 and September 2015. Only Patients with suspected small bowel bleeding were included for analysis. Patients' characteristics, decision and outcome after CE were analyzed including the diagnostic and therapeutic yield of device assisted enteroscopy.

*Results*. A total of 44 patients were included in the analysis. The mean age was 62. 72% of patients were male. The indication was overt bleeding in 41 and occult bleeding in 3 patients. The small bowel completion rate for CE was 75% (*n* = 33) with retention rate of 2.2% (*n* = 1). Diagnostic yield was 63% (*n* = 28). Among patients who had small bowel pathology (*n* = 27), vascular lesions were the most common 59.3% (*n* = 16), followed by ulcer/erosion 22.2% (*n* = 6), mass lesion 3.7% (*n* = 1) and blood of unknown origin 14.81% (*n* = 4). Of all patients with no identified small bowel pathology on CE, no patient required device assisted enteroscopy. Of all patients with positive small bowel pathology on CE (*n* = 27), 12 patients underwent device assisted enteroscopy: 11 single balloon enteroscopy and 1 retrograde enteroscopy with through the scope balloon catheter system (NaviAid™, SMART Medical Systems). Among patients who underwent device assisted enteroscopy, the diagnostic yield was 66% (*n* = 8), all of which received therapeutic intervention: 7 had thermal coagulation and 1 had polypectomy.

*Conclusions*. Inpatient CE is a useful tool to guide the need for device assisted enteroscopy with a small bowel completion rate comparable to outpatient CE.

*Funding Agencies: *None

# Sub article: A107: Constipation as the Primary Presentation of Celiac Disease

**Authors:** A. Sarkhy, Z. Zaidi, A. Saeed, A. Assiri

**Affiliations:** ^107.1^Department of PediatricsKing Khalid University HospitalKing Saud UniversityRiyadhSaudi Arabia

*Background*. Celiac disease (CD) is a multifactorial, autoimmune disorder that occurs in genetically susceptible individuals. While typical CD presents with chronic diarrhea, steatorrhea, abdominal distention and failure to thrive, atypical CD may has subtle presentation and can be easily missed or overlooked. Constipation is one of the atypical presentations of CD and thus selected patients may need celiac screening for early diagnosis of the disease.

*Aims*. The aim of this study is to describe the frequency and clinical characteristics of children with CD patients who presented primarily with constipation.

*Methods*. This is a retrospective study conducted between the periods of January 2013 to June 2014, at King Khalid University Hospital, Riyadh, Saudi Arabia. We included children less than 18 years of age with confirmed diagnosis of CD based on serology and small bowel biopsies. We collected data about the clinical characteristics of the patients, details of the bowel movements, anthropometric measurements, treatment and the outcomes.

*Results*. 100 cases of confirmed CD were found during the study period with a mean age of 7 years (range; 9 months to 18 years). There were 68 males (68%). Fifty four percent presented with classical symptom, while 46% presented with atypical presentations. Constipation was found in 15 patients (15%). The mean duration of the constipation was 7 months (±3 months SD) before the diagnosis. Severe constipation (bowel opening once a week) was observed in 8 (53.3%) and rest had mild to moderate constipation. Mild to moderate abdominal pain and distension were observed in the severe constipated children. None of the children had a satisfactory response to laxatives therapy prior to the diagnosis of CD; however, all of them responded very well when they follow the strict GFD with total resolution of the symptoms.

*Conclusions*. CD should be kept in mind during the work up for patient with intractable constipation. Early diagnosis and early introduction of GFD improves the bowel habits among those patients.

*Funding Agencies: *None

# Sub article: A108: Balloon-Assisted Enteroscopy Is an Emerging Tool in the Management and Maintenance of Crohn's Disease

**Authors:** M. Reeson, S. Zepeda-Gomez, R. Fedorak, K. Kroeker, B. Halloran

**Affiliations:** ^108.1^University of AlbertaEdmontonABCanada; ^108.2^University of AlbertaEdmontonABCanada

*Background*. Seventy percent of CD patients suffer from small bowel involvement. Balloon-Assisted Enteroscopy (BAE) allows for improved visualization and for therapeutic intervention.

*Aims*. This study aims to assess the safety and efficacy of balloon-assisted enteroscopy in the management and maintenance of small bowel Crohn's disease.

*Methods*. A retrospective chart review was undertaken to examine all patients with small bowel CD who underwent BAE over the last four years. The data collection included patient demographics, disease characteristics, surgical history, procedure characteristics, and stricture dilation data were all recorded.

*Results*. 68 BAEs were performed on 42 patients (23 male). The mean age at BAE was 41.3 ± 15.7 yrs. 58 (85.3%) of procedures were on patients with known Crohn's disease. 35 (83.3%) of patients had ileal CD, 4 (9.5%) had jejunal. 26 (61.9%) of patients had at least one previous bowel resection. Stricturing was the most common complication with 28 (66.7%) showing stenosis. 17 patients (12 male) underwent serial (>1) BAE procedures. 21 patients underwent 29 operations involving balloon-assisted stricture dilation. 66 strictures were dilated in total. Of all BAE procedures, 2 (2.9%) were met with complications; 1 patient developing pancreatitis, the other with minor bleeding. There were no perforations or deaths.

*Conclusions*. Balloon assisted enteroscopy and balloon-assisted stricture dilation are safe and effective procedures for visualization and treatment of small bowel Crohn's disease and are likely under-utilized as an endoscopic tools. The combination of therapeutic intervention with direct and extensive mucosal visualization makes BAE the optimal modality for diagnosis and treatment of small bowel CD.

*Funding Agencies: *Self Funded (CEGIIR)

# Sub article: A109: Magnetic Resonance Enterography in the Assessment of Post-Operative Crohn's Disease

**Authors:** M. Scaffidi, K. Lim, P. Vlachou, G. Mnatzakanian, O. Zarghom, E. Irvine, E. Colak, S. Grover

**Affiliations:** ^109.1^University of TorontoTorontoONCanada

*Background*. In Crohn's disease (CD) post-ileocolic resection, the ileal component of the Rutgeerts score at colonoscopy prognosticates recurrent disease at the anastomosis, but is limited by potential inability to cannulate the neoterminal ileum due to stricturing, which occurs in >30% of cases. Magnetic resonance enterography (MRE) is a preferred imaging modality for small bowel CD, but its operating characteristics for predicting recurrent ileal disease post-ileocolic resection – for which post-surgical and inflammatory findings may be conflated – have not been clearly identified.

*Aims*. To determine the association of MRE findings of neoterminal ileal CD activity with Rutgeerts score at ileocolonoscopy.

*Methods*. This retrospective study enrolled patients with prior ileocolic resection for CD with MRE and colonoscopy performed within 90 days at a single academic centre. Exclusion criteria were poor bowel preparation or inability to reach the ileocolic anastomosis at the time of colonoscopy, or change in therapy between MRE and colonoscopy. Clinical information was collected to calculate Crohn's disease activity index (CDAI). MRE studies were interpreted by two experienced abdominal radiologists blinded to clinical and endoscopic information. The primary outcome was association between Rutgeerts score and the following MRE findings: bowel wall thickness, mural edema, enhancement pattern, ulceration, stenosis, prestenotic dilatation, mesenteric hyperemia and mesenteric lymphadenopathy. A secondary outcome was association between MRE findings and CDAI score. The association between Rutgeerts and CDAI scores to MRE findings were determined using the exact Wilcoxon rank-sum test and Spearman correlation for categorical and continuous predictors, respectively.

*Results*. 25 patients who met the inclusion criteria were included in the analysis. MRE findings that had a significant positive correlation (*ρ* > 0) with Rutgeerts score are: mural thickness (*p* = 0.02); mural edema (*p* = 0.04); ulceration (*p* = 0.02); mesenteric edema (*p* < 0.01); and mesenteric hyperemia (*p* = 0.01). We found no statistically significant association between mesenteric lymphadenopathy, luminal narrowing or fistulizing disease with the Rutgeerts score. There was a significant, positive correlation between mural thickness (*p* = 0.03), mesenteric edema (*p* = 0.02) and reduced peristalsis (*p* = 0.02) with CDAI.

*Conclusions*. In patients with ileocolic resection for CD, radiological characteristics from MRE correlate with known endoscopic-prognostic and disease activity scoring systems. Further research should codify these findings into an MRE rating tool to prognosticate recurrent CD post-ileocolic resection.

*Funding Agencies: *CAG

# Sub article: A110: Successful Eradication of Recurrent Clostridium Difficile Infection (rCDI) of Small Bowel with Frozen Encapsulated Fecal Microbiota Transplantation (FMT) in a Patient with Crohn's Disease and Ileostomy

**Authors:** J. Zhu, B. Roach, D. Kao

**Affiliations:** ^110.1^University of AlbertaEdmontonABCanada

*Aims*. We report a case of ileal-pouch Crohn's disease with rCDI of small bowel who failed vancomycin treatment, and is successfully treated with frozen FMT from upper GI tract without any adverse events.

*Methods*. Case report.

*Results*. A 31 year-old male underwent subtotal colectomy and ileostomy in Sept 2013 for ulcerative colitis as he did not respond to infliximab. His post operative course was complicated by high grade small bowel obstruction, requiring multiple hospital admissions, and subsequently found to have Crohn's involving neoterminal ileum. His maintenance therapy consisted of infliximab at 10 mg/kg q 4 weeks and methotrexate 25 mg SQ weekly. At baseline, he empties his ileostomy bag 4-5 times per day, each time about 250 cc of mushy stools. He developed his first episode of CDE in Jan 2014, during one of these post operative admissions. His stool C. difficile toxin was positive with no other enteric pathogens or alternative diagnosis identified. Ileoscopy revealed only mild patchy mucosal inflammation. He was treated with oral metronidazole 1 g daily for 10 days with symptom resolution. Unfortunately, his symptoms recurred within 2 weeks of discontinuing metronidazole. A repeat C diff toxin was again positive, and he responded well to a course of metronidazole. His symptoms recurred again within 2 weeks of discontinuing metronidazole, associated with positive C diff toxin again. He was then treated with a long tapered course of vancomycin, again with symptom resolution. Unfortunately, his diarrhea recurred shortly after discontinuing vancomycin. In total, he had 6 episodes of recurrent CDE between Jan 2014 and March 2015. He was referred to the Edmonton FMT Program for consideration of FMT in May 2015. He received encapsulated FMT, consisted of 30 capsules daily for 3 days, from one of the universal stool donors registered with the program. The patient reported having more formed stools in his ileostomy within the first week post FMT, and by week 3 his bowel habit had returned to baseline. He had no adverse events from FMT or rCDI during the follow-up period from May to Aug 2015.

There are few literatures on successful treatment of small bowel rCDI using frozen encapsulated FMT. Not only do IBD patients have an increased risk of developing CDI, but they can also develop CDI in the small bowel and ileal pouch-anal anastomosis (IPAA) following colectomy. Post operative mechanical complications, male gender and serum immunoglobulin G1 deficiency have been identified as risk factors for recurrent pouch CDI.

*Conclusions*. Frozen encapsulated FMT appeared to be a safe and effective therapeutic alternative for patients with small bowel rCDI, and warrants further investigation.

*Funding Agencies: *None

# Sub article: A111: Efficacy and Safety of Over-the-Scope Clip (OTSC) in the Endoscopic Closure of Fistula and Perforation in the Gastrointstinal Tract: A Case Series

**Authors:** A. Yau, E. Lam, R. Enns, F. Donnellan

**Affiliations:** ^111.1^University of British ColumbiaVancouverBCCanada

*Background*. Over-the-scope clip (OTSC) (Ovesco Endoscopy GmbH, Tübingen, Germany) is a novel device utilized in the management of fistula, perforation, dehiscence, and bleeding in the gastrointestinal tract via tissue approximation and compression.

*Aims*. To determine the efficacy and safety of OTSC in the endoscopic closure of fistula and perforation in the gastrointestinal tract.

*Methods*. A retrospective chart review was performed.

*Results*. Seven patients (mean age 62.9 years; 3 women (42.9%)) were treated with OTSC from 10/13 to 03/15 in an outpatient (42.9%) or inpatient (57.1%) setting and on an elective (14.3%), semi-elective (42.9%), or urgent (42.9%) basis.

The gastrointestinal diagnosis and treatment were nausea/vomiting with fistulizing percutaneous endoscopic gastrostomy tube (*n* = 1), duodenal ulcer perforation with failed Graham omental patch (*n* = 1), gastric cancer with total gastrectomy and leaking esophagojejunal anastomosis (*n* = 1), transverse colon cancer with left hemicolectomy and fistulizing primary anastomosis (*n* = 1), and rectosigmoid cancer with low anterior resection and leaking primary anastomosis (*n* = 3).

The OTSC was utilized in the endoscopic closure of gastrocutaneous fistula (*n* = 1), duodenal ulcer perforation (*n* = 1), jejunocutaneous fistula (*n* = 1), colocutaneous fistula (*n* = 1), and rectocutaneous fistula (*n* = 3). The defect size ranged from 2 to 10 mm. Technical success with defect closure was achieved completely in 62.5% (5/8 clips) and partially in 25.0% (2/8 clips). There were no complications related to OTSC application.

Additional interventions were hemoclips (*n* = 2), argon plasma coagulation (*n* = 1), sclerotherapy with histoacryl and lipiodol (*n* = 2), and hyperbaric oxygen (*n* = 1). Clinical success was achieved in 71.4% (*n* = 5). One patient required surgical resection of fistula for definitive management. Another patient died of persistent bleeding from anastomotic site.

*Conclusions*. The endoscopic application of OTSC appeared to be safe. The rates of technical success and long-term clinical success were satisfactory. Future prospective studies should compare the relative efficacy of OTSC to other endoscopic modalities in an effort to determine the most optimal indications and to maximize clinical outcomes.

*Funding Agencies: *None

# Sub article: A112: Q Fever in a Patient with Crohn's Disease on Adalimumab and Methotrexate

**Authors:** M. Alkhattabi, S. Hosseini-Moghaddam, A. AlNasser, M. Beaton

**Affiliations:** ^112.1^Western UniversityLondonONCanada; ^112.2^London Health Sciences CentreLondonONCanada

*Background*. Q fever has been rarely reported in patients with inflammatory bowel disease (IBD) on immunosuppressive therapy.

*Aims*. To present a confirmed case of Q fever in a gentleman with Crohn's disease (CD) and review the literature. The patient presented with fever of unknown origin who despite a lack of direct contact with zoonotic vectors, after an extensive evaluation he was eventually diagnosed and treated successfully for Q fever.

*Methods*. Case report and literature review.

*Results*. A 53-year-old automotive mechanic with a 30 year history of CD in remission with combination Adalimumab and Methotrexate since 2006. He was well until 2 weeks prior to his presentation when he developed a persistent fever and drenching night sweats. Over this period, he experienced a 5 lb weight loss but denied any symptoms suggestive of a flare of his underlying CD. His systemic review and physical examination were otherwise unremarkable. Initial investigations demonstrated a normal white blood cell count but significantly elevated CRP (121 mg/L). He was admitted to hospital and following acquisition of blood, stool and urine cultures, started on broad spectrum antibiotics. All cultures were negative and further evaluation demonstrated positive antinuclear antibody and rheumatoid factor, but negative viral, histoplasmosis and blastomycosis serologies. Imaging studies were unremarkable. WBC scan were negative. Gastroscopy and colonoscopy were normal. The infectious disease service was involved and requested Q fever serology which confirmed recent infection. He was started on a 10 day course of oral Doxycycline (200 mg every 24 hours) with resolution of his fever by day 3. Ongoing follow up with ID as an outpatient was arranged with serial monitoring of Q fever. Without discontinuation of treatment for CD, he continued treatment for Q fever. The process of improvement was not complicated by any significant event.

After obtaining further history, the patient was likely exposed through servicing vehicles used to transport sheep's. Only one previous case of acute hepatitis due to Q fever in an IBD patient on chronic treatment with steroids has been reported.

*Conclusions*. To the best of our knowledge, this is the first reported case of acute Q fever in a known case of CD receiving Adalimumab and metothrexate. In spite of simultaneous immunosuppressive therapy, the patient did not develop any organ involvement which was reported in previous case report. This case report shows management of acute Q fever is successful despite continuing immunosuppression with biologic therapy.

*Funding Agencies: *None

# Sub article: A113: The Effect of Longer Battery Life on Small Bowel Capsule Endoscopy: A Single Centre Experience

**Authors:** G. Ou, C. Galorport, R. Enns

**Affiliations:** ^113.1^University of BCVancouverBCCanada

*Background*. Small bowel capsule endoscopy (CE) does not completely visualize the small bowel approximately 16.5% of the time due to limited battery life. Patients with incomplete and negative CE may require additional investigation to clear the rest of the small bowel. Previous study suggests that complete examination rate (CER) may be improved by simply extending the battery life and recording time.

*Aims*. To determine the CER and diagnostic yield of CE with longer battery life.

*Methods*. A prospective observational study is currently recruiting at St. Paul's Hospital (Vancouver, BC). Inclusion criteria: age ≥ 19. Exclusion criteria: dysphagia, endoscopic placement of CE, active bowel obstruction, and concomitant enrollment in another study receiving motility-altering treatment.

All subjects underwent bowel preparation with 2L PEG3350 with electrolytes the day before, and fasted ≥ 2 h prior to the procedure. They were allowed to drink clear fluids and resume normal diet 2 h and 4 h post-capsule ingestion, respectively. Given Imaging PillCam SB3™ (Yoqneam, Israel) were used.

Gastric transit time is defined as time of first duodenal image minus time of first gastric image. Small bowel transit time is defined as time of first cecal image minus first duodenal image; or time of last image recorded minus first duodenal image if CE did not reach cecum. CE is considered complete if colonic mucosa is demonstrated on recording.

This study was approved by institutional research ethics board and registered on clinicaltrials.gov (NCT02382705).

*Results*. From 02/2015 to 09/2015, 57 subjects, consisting of 29 (50.9%) male, were enrolled in the study. Mean age was 53.6 ± 15.7 years. The most frequent indication was GI bleeding (*n* = 41, 71.9%), followed by IBD (*n* = 8, 14.0%), polyposis syndrome/malignancy (*n* = 5, 8.8%), and history of obstruction (*n* = 3, 5.2%). Seven (12.3%) CE's were incomplete, 5 of which were due to retention in the stomach for the duration of the recording. Twenty-four (42.1%) subjects had prior history of bowel resection proximal to the ileocecal valve. All 5 patients with gastric retention had previous bowel surgeries. Among the remaining 33 subjects, mean GTT was 37.6 ± 92.1 min and SBTT was 232.1 ± 98.3 min. Overall mean recording time was 785.8 ± 149.1 min Endoscopic findings were identified in 20 (35.1%) CE's.

*Conclusions*. CE with extended battery life appears to be a simple way to ensure high CER.

*Funding Agencies: *None

# Sub article: A114: Control of Gut Homeostasis by Membrane Trafficking Regulators

**Authors:** S. Jean

**Affiliations:** ^114.1^Université de SherbrookeSherbrookeQCCanada

*Background*. Approximately 233 000 Canadians suffer from Crohn's disease or ulcerative colitis, which are collectively named inflammatory bowel diseases (IBD). It is now well established that IBD are multifactorial diseases occurring in individuals with specific genetic predispositions. The impact of the host genome on IBD susceptibility and progression or the host interaction with the gut microbiota is not well defined. Genome wide association studies (GWAS) have identified multiple genes linked to IBD, illuminating some of the genetic aspect of IBD. However, the effects of these variants and how they predispose individuals to IBD are for most of them unknown. The lack of a direct an easily amenable* in vivo* system to study gene functions in the context of intestinal cell biology, and intestinal interaction with microbes has precluded large scale studies of GWAS identified variants. Drosophila melanogaster represents an ideal system in which to perform such studies since its intestinal biology is generally conserved with humans, that multiple intestinal human pathogens can cause related pathologies in flies, that a large array of tools are available for loss and gain of function studies and that it represents an economical research model. In predisposed individuals, IBD development is triggered by poorly understood environmental and microbial factors. The combination of these factors eventually leads to chronic immune response in the gut, resulting in inflammation and high stress responses in intestinal cells. The membrane-mediated process of autophagy defined as the lysosomal degradation and recycling of cytoplasmic components, is an important stress response mechanism. Importantly, multiple genes regulating specific aspects of autophagy and membrane trafficking were identified as IBD risk variants in GWAS studies. Importantly, membrane and autophagy regulators were identified in Drosophila as important regulators of gut stem cell renewal.

*Aims*. To characterize the role of membrane trafficking regulators in autophagy and to understand their importance on gut homeostasis.

*Methods*. Here, I will describe the establishment of new tools to probe and understand the role of membrane regulators in IBD.

*Results*. I will show how Drosophila can be used as an accelerated screening tool for investigating the biological relevance of membrane trafficking IBD-related risk variants.

*Conclusions*. Drosophila represents a great and affordable model organism to identify and describe genes involved in gut homeostasis. Results obtains in fly will thus dictate our rationale in developing translational strategies in the context of conditional knockout mouse models as well as human patients.

*Funding Agencies: *CIHR, Startup funds, Université de Sherbrooke and Centre de Recherche du CHUS

# Sub article: A115: A TH17-Neutrophil Axis Involved in the Containment of Commensal Microbiota

**Authors:** K. Flannigan, V. Ngo, D. Geem, A. Harusato, S. Hirota, T. Denning

**Affiliations:** ^115.1^Georgia State UniversityAtlantaGAUSA; ^115.2^University of CalgaryCalgaryABCanada

*Background*. At mucosal surfaces certain bacteria, including segmented filamentous bacteria (SFB) in mice, elicit the differentiation of T helper 17 (Th17) cells. While Th17 cells can potentiate IBD, they play an important role in barrier function by containing the microbiota. How Th17 cells achieve this is incompletely understood but involves, in part, the cytokine IL-22 produced in response to IL-23.

*Aims*. Recent evidence suggests neutrophils can also produce IL-22 to promote intestinal barrier protection, therefore we hypothesized that neutrophils may be involved in an axis with Th17 cells that can contain mucosal bacteria, specifically SFB, to protect mucosal surfaces.

*Methods*. SFB-free (SFB−) mice were obtained from Jackson and SFB+ mice were obtained from Taconic. Feces from SFB+ mice were mixed into drinking water and given to mice for 7 days. Small intestinal sections were digested in collagenase to isolate lamina propria cells for flow cytomertry. Monocloncal antibodies were used to block IL-17A or deplete CD4+ T cells and neutrophils (*α*Ly6G). SFB loads were detected via qPCR performed on fecal DNA. Ileal explants were taken using biopsy needles and stimulated with IL-23.

*Results*. After colonizing SFB− mice with SFB-containing microbiota, we observed an influx of neutrophils into the ileum correlating with the load of SFB over the course of 7 days. Neutrophil numbers also correlated with IL-17A and IL-22 expression. Based on studies suggesting a role for IL-17A in neutrophil recruitment we injected SFB- mice with *α*IL-17A antibody. 7 days post-SFB colonization *α*IL-17A-injected mice had lower levels of neutrophils in the ileum compared to isotype treated mice. This same effect was observed after *α*CD4 injection in mice with SFB-containing microbiota, suggesting CD4+ T cells as a source of IL-17A. We next utilized *α*Ly6G to deplete neutrophils and found that after 7 days of colonization there was a significant expansion in SFB loads compared to isotype treated mice. The expansion of SFB was also seen after *α*IL-17A treatment or CD4 depletion. Both *α*Ly6G and *α*IL-17A treated mice also had higher levels of Th17 cells in the ileum. Finally, incubation of ileal explants with IL-23 promoted robust IL-22 production along with an increase in the expression of the antimicrobial peptides SA100A8, SA100A9, and RegIII*α*; all effects that were lost after neutrophil depletion.

*Conclusions*. After introduction of SFB, intestinal CD4+ T cells produce IL-17A to recruit neutrophils. Upon entering the intestine neutrophils are exposed to IL-23 prompting IL-22 production, which induces expression of antimicrobial peptides to help control SFB. Without proper neutrophil responses, SFB levels expand, increasing Th17 cell number in the intestine that may potentiate disease.

*Funding Agencies: *NIH, Snyder Institute for Chronic Diseases, Cumming School of Medicine, None

# Sub article: A116: Adult Nod2^−/−^ Mice Show an Altered Microbial Resilience and an Increased Fecal IGA Response following Antibiotic Perturbation of the Microbiota

**Authors:** A. Goethel, W. Turpin, S. Rouquier, G. Zanello, D. Philpott, K. Croitoru

**Affiliations:** ^116.1^University of TorontoTorontoONCanada; ^116.2^Mount Sinai HospitalTorontoONCanada

*Background*. Inflammatory bowel diseases (IBD) are multifactorial diseases, involving genetic mutations, environmental triggers and alterations of the gut microbiota. The strongest genetic association is with nucleotide-binding oligomerization domain-containing protein 2 (NOD2), a pattern recognition receptor that recognizes a component of the bacterial cell wall. During adulthood, environmental perturbations, such as antibiotics, induce transient shifts in the microbiota composition.

*Aims*. We sought to determine whether Nod2 played a role in antibiotic-induced dysbiosis and/or resilience of the microbiota. Moreover, we investigated whether the post-antibiotic microbiota in a Nod2-deficient mouse could lead to an altered immune response.

*Methods*. Adult WT and* Nod2* ^−*/*−^ mice received control water or amoxicillin (200 mg/L)* ad libitum* in drinking water for 7 days, followed by control water for 4 weeks. Fecal samples were collected weekly to monitor changes in the microbial community structure using 16S sequencing. Fecal IgA was used to assess the mucosal immune response to the microbiota over time. On day 35, small intestinal mucosal damage was induced by acute polyclonal T cell activation following i.p. anti-CD3 injection.

*Results*. Bacterial load, measured using targeted qPCR for the beta-subunit of bacterial RNA polymerase (rpoB), was significantly reduced at day 7 in antibiotic-treated mice; coinciding with a significant shift the in the microbial community structure of both WT and* Nod2* ^−*/*−^ mice. Antibiotic treatment resulted in a significant reduction of Firmicutes and a bloom of Proteobacteria at day 7. By day 21 (two weeks after removal of antibiotics), WT mice had returned to their pre-treatment community structure, however* Nod2* ^−*/*−^ mice retained a significantly different microbiota from pre-treatment, highlighted by a significant reduction in *α*-diversity. Fecal IgA was significantly enhanced at day 7 in antibiotic-treated* Nod2* ^−*/*−^ mice; returned to normal at day 21. Water-treated* Nod2* ^−*/*−^ mice had a significantly enhanced IL-17 response following i.p. anti-CD3 injection, which was reduced by antibiotic treatment.

*Conclusions*. Our data suggests that Nod2 plays a significant role in resilience of the gut microbial community structure following perturbation. Moreover, antibiotic alteration of murine adult microbiota in* Nod2* ^−*/*−^ mice leads to an altered immune response to the commensal gut microbiota.

*Funding Agencies: *CAG, CIHR, OGS and Mount Sinai Hospital

# Sub article: A117: A Prospective, Dual Center, Randomized Trial Comparing Colonoscopy versus Capsule Delivered Fecal Microbiota Transplantation (FMT) in the Management of Recurrent Clostridium Difficile Infection (RCDI)

**Authors:** D. Kao, B. Roach, N. Hotte, M. Silva, K. Madsen, P. Beck, T. Louie

**Affiliations:** ^117.1^University of AlbertaEdmontonABCanada; ^117.2^University of CalgaryCalgaryABCanada

*Background*. FMT is highly effective in the treatment of RCDI. Various delivery mechanisms have described, resulting in success rate of 80–90%. However, the ideal route of delivering FMT has not yet been determined.

*Aims*. To compare the efficacy, safety, patient preference, and cost between colonoscopy versus encapsuled FMT.

*Methods*. This prospective, dual center trial randomized patients with RCDI in Edmonton and Calgary, Alberta, to FMT by oral capsule or colonoscopy at 1 : 1 ratio. Inclusion criteria were: (1) age > 18 and (2) at least 3 episodes of CDI. Exclusion criteria included: (1) complicated CDI; (2) chronic diarrhea; (3) chemotherapy/radiation; (4) dysphagia; (5) ileus or small bowel obstruction; (6) colostomy or ileostomy. Seven universal stool donors registered with our FMT programs provided stool materials. The primary objective was to compare the cure rate of RCDI by FMT delivered by capsules versus colonoscopy. The secondary objectives included: (1) safety of FMT by each delivery modality; (2) patient preference and satisfaction; (3) cost difference. There was a significant increase in quality of life after FMT. The cost of capsule was significantly lower than colonoscopy FMT.

*Results*. A total of 43 patients had been randomized to date, with 22 in capsule group and 21 in colonoscopy group. Patient baseline characteristics were shown in Table 19. The cure rate was 100% in colonoscopy and 92% in capsule group following one treatment. There were no fevers, infections attributable to FMT, or colonic perforation. One patient had mild nausea and vomiting from sedation following colonoscopy. One patient had nausea and vomiting following capsule ingestion. Only 7% of patients found the idea of FMT by capsules unpleasant, compared to 24% by colonoscopy.

*Conclusions*. FMT by either colonoscpy or capsules appeared to be similar in efficacy. Significant improvement in quality of life was seen in both groups of patients. No significant adverse events had occurred in any patients in either group. More patients preferred FMT by capsules to colonoscopy. Capsule administered FMT was less expensive and required less resources compared to colonsocopy.

*Funding Agencies: *CAG, Alberta Health Services

# Sub article: A118: Independent Predictors of Failure of Fecal Microbiota Transplantation (FMT) for Recurrent or Refractory* Clostridium Difficile* Infection

**Authors:** J. Dimitry, A. Keshteli, D. Kao

**Affiliations:** ^118.1^Department of MedicineUniversity of AlbertaEdmontonABCanada

*Background*. Fecal microbiota transplantation (FMT) is a safe and effective alternative therapy for treatment of refractory or recurrent* C. diff *infection (RCDI), with an overall success rate of 80–90% with one treatment. However, it is unknown which patients will require more than one FMT or who will not respond to FMT.

*Aims*. Our goal was to identify clinical predictors of treatment failure following a single FMT in recurrent or refractory disease.

*Methods*. This retrospective study included 136 patients who received FMT for refractory or recurrent CDI at the University of Alberta hospital. Patient baseline characteristics and clinical datasets were abstracted from in-patient charts and electronic medical records.

*Results*. Of the 136 patients, 106 (77.9%) were cured following one FMT. Univariate analysis identified 7 factors, shown in Table 20, associated with failure of 1 FMT. Multivariate analysis revealed that inpatient status (OR 7.4; 95% CI 2.2–24.6, *p* = 0.001), immunosuppression (OR 4.5; 95% CI 1.3–16.2, *p* = 0.020), and severe or complicated CDI (OR 5.2; 95% CI 1.2–21.6, *p* = 0.025) were factors most independently associated with failure following 1 FMT. All 9 refractory patients failed the 1st FMT. Of the 30 patients (22.1%) who failed the first FMT, 25 received a second and 16 were cured.

*Conclusions*. Inpatient status, immunosuppression, and severe or complicated disease are independent predictors of treatment failure following 1 FMT in RCDI patients. Clinicians can predict patients most at risk of treatment failure and adjust treatment and discharge planning accordingly.

*Funding Agencies: *CAG

# Sub article: A119: *Giardia muris* Protects against* Citrobacter rodentium *Infection: Effects on the Inflammosome and Gut Antimicrobial Peptides

**Authors:** A. Manko, J. Motta, J. Cotton, A. Oyeyemi, B. Vallance, J. Wallace, A. Buret

**Affiliations:** ^119.1^University of CalgaryCalgaryABCanada; ^119.2^BC Children's HospitalVancouverBCCanada; ^119.3^Antibe Therapeutics Inc.TorontoONCanada

*Background*. Our knowledge of polymicrobial gastrointestinal infections effects on host innate immune responses remains incomplete. The diarrheagenic protozoan parasite* Giardia* sp. alters gut microbiota and dampens host inflammatory responses. The production of intestinal epithelial anti-microbial peptides (AMPs) and activation of canonical and non-canonical inflammasomes play essential roles in host defense against various enteropathogens.* Citrobacter rodentium *infection in rodents is a model of Enteropathogenic* Escherichia coli* (EPEC) and Enterohemorrhagic* E*.* coli* (EHEC) in humans. In a study perfomed in Tanzania, Giardia-infected children seemed to be protected from diarrhea via unknown mechanisms.

*Aims*. The aim of this study was to determine if* Giardia muris* infection protects against* C. rodentium* colitis by enhancing AMPs production and/or activation of the inflammasome, and its key components caspase-1 and caspase-11.

*Methods*. Male C57BL/6 mice were co-infected with green fluorescent protein (GFP)-labeled* Citrobacter rodentium,* and* Giardia muris *trophozoites. Weight gain, fecal blood, histopathology, and colonic* C*.* rodentium* colonization were recorded. GFP-*Citrobacter rodentium, β*-Defensin-2 and TFF-3 AMPs were visualized by immunofluorescence. Colonic caspase-1 and -11, and TFF3 and *β*-Defensin-2 protein levels were assessed by Western blot.

*Results*. At 14 days post-infection, mice infected with* C*.* rodentium* had an inflamed colon, lost weight and passed fecal blood. Co-infection with* G*.* muris* significantly inhibited these effects. In* Giardia* co-infected animals, mucosal adhesion and translocation of* C*.* rodentium* were reduced. Caspase-1 and -11 protein levels were increased in the co-infected group compared to animals given* C*.* rodentium* alone, suggesting* Giardia *activates canonical and noncanonical inflammasome pathways. In the colon of mice co-infected with* Giardia*, concentration and expression of *β*-Defensin-2 and TFF3 proteins were increased.

*Conclusions*. The findings indicate that* Giardia* upregulates AMP production and inflammasome pathways, and protects against* C*.* rodentium* colitis. The findings shed new light on how microbial-microbial interactions may protect children against bacterial enteritis in zones endemic for giardiasis.

*Funding Agencies: *CIHR

# Sub article: A120: Afuabc Is a Phosphorylated Carbohydrate Transporter That Promotes Intestinal Colonization and Transmission of* Citrobacter rodentium*

**Authors:** S. Crowley, B. Sit, K. Bhullar, C. Lai, C. Tang, Y. Hooda, C. Calmettes, H. Khambati, C. Ma, J. Brumell, A. Schryvers, T. Moraes, B. Vallance

**Affiliations:** ^120.1^University of British ColumbiaVancouverBCCanada; ^120.2^University of TorontoTorontoONCanada; ^120.3^University of CalgaryCalgaryABCanada; ^120.4^Hospital for Sick ChildrenTorontoONCanada; ^120.5^BC Children's HospitalVancouverBCCanada

*Background*. Attaching and effacing (A/E) pathogens cause severe diarrheal disease in their respective hosts. In order to colonize the mucosal surfaces of intestinal epithelial cells, these bacterial pathogens, such as enterohemorrhagic* Escherichia coli* (EHEC) and its murine-specific relative* Citrobacter rodentium*, must scavenge extracellular nutrients required for virulence from the metabolically competitive environment of the intestinal lumen.

*Aims*. Here, we investigated the function of the bacterial AfuABC transport system and its role in* C*.* rodentium* pathogenesis.

*Methods*. This study employed a variety of biochemical and microbiological techniques as well as a three animal infection models to elucidate the role of AfuABC.

*Results*. By crystallographic and biochemical approaches, we demonstrate that AfuABC is a cyclic hexose/heptose-phosphate transporter with high specificity and selectivity for glucose-6-phosphate, fructose-6-phosphate and sedoheptulose-7-phosphate. AfuABC is present in both EHEC and* C*.* rodentium*, and located adjacent to the putative pathogenicity island OI-20 which regulates carbohydrate detection and acquisition. A competitive index assay, simultaneous infection of* C*.* rodentium* wildtype and Δ*afuA* to measure comparative fitness, revealed Δ*afuA* was significantly impaired in both shed stool (6 days post infection, d.p.i.) and colonic tissues (10 d.p.i.). Interestingly,* C*.* rodentium* Δ*afuA* did not display any significant difference from wildtype in either* in vitro *and* in vivo* single infections, type three effector secretion, bacterial tissue localization or colonic pathology. However in a transmission model, measuring* C*.* rodentium *transmission to naïve mice via shed stool, the ability of* ΔafuA *to transmit and establish colonic disease* was *significantly decreased compared to wildtype. Sugar-phosphates were detected by targeted tandem liquid chromatography/mass spectrometry in both normal and infected intestinal mucus and stool samples, indicating these nutrients are available for enteric bacteria to metabolize during infection.

*Conclusions*. These results indicate that AfuABC-dependent uptake of sugar-phosphates impacts the ability of* C*.* rodentium *to transmit to new hosts and uncovers a previously unrecognized role for these metabolites as important contributors to successful pathogenesis.

*Funding Agencies: *CAG, CIHR

# Sub article: A121: Human Microbiota Modulates Intraepithelial Lymphocyte Numbers and Phenotype in the Mouse Small Intestine: Implications for Chronic Intestinal Inflammation

**Authors:** J. McCarville, M. Meisel, H. Galipeau, Y. Sanz, B. Jabri, E. Verdu

**Affiliations:** ^121.1^McMaster UniversityHamiltonONCanada; ^121.2^University of ChicagoChicagoILUSA; ^121.3^Microbial EcologyNutrition & Health Research GroupInstitute of Agrochemistry and Food TechnologyNational Research Council (IATA*-*CSIC)ValenciaSpain

*Background*. Intraepithelial lymphocytes (IELs), *αβ*TCR and *γδ*TCR, play a critical role in mucosal barrier maintenance and as demonstrated by some gnotobiotic studies, their populations are modulated by commensal colonization. Previous research has demonstrated that IELs have increased cytolytic activity in Crohn's patients, are responsible for infection-associated damage in the small intestine, and are critical for the development of villous atrophy in celiac disease (CeD). The factors that lead to increased numbers or cytotoxic transformation of IELs described in chronic intestinal inflammatory disorders such as Crohn's and CeD are unclear. Interestingly both disorders are associated with alterations in the gut microbiota and with presence of pathobionts in the intestine.

*Aims*. To investigate whether the microbiota from CeD patients, influence IEL numbers and phenotype in the small intestine of gnotobiotic mice.

*Methods*. We transferred human fecal bacteria from patients with active CeD (collected anaerobically at diagnosis) or healthy individuals into germ-free (GF) C57BL/6 mice.

*Results*. Colonization with microbiota from a HLA-DQ8 heterozygous donor (CeD2), markedly increased IEL numbers within small intestine villi tips compared to colonization with microbiota from the other donors. The phenotype of IELs and inflammatory status of the small intestine was also affected by CeD2 microbiota, with increased proportions of CD3^+^CD8^+^ *αβ*TCR^+^IELs, and increased small intestinal IFN-*γ* expression. Sequencing of small intestinal contents in CeD2 recipient mice revealed a higher composition of Proteobacteria, specifically* Parasutterella*. Further investigation by colonization of MyD88^−/−^; Ticam1^−/−^ mice demonstrated that IEL responses to CeD2 colonization were dependent on these signaling adaptors.

*Conclusions*. These results suggest that the composition of the gut microbiota can alter IEL numbers and phenotype in the small intestine through a MyD88/TICAM1 pathway, leading to IFN-*γ* induction. In a subgroup of patients with genetic predisposition, presence of pathobionts may promote cytotoxic transformation of IELs and contribute to disease pathogenesis. This mechanism has important implications for CeD, and IBD.

*Funding Agencies: *CIHR, Canadian Celiac Association (CCA)

# Sub article: A122: Antibiotics Administration Alters the Fecal Microrna Signature of Healthy Subjects

**Authors:** A. Taibi, S. Girard, T. Tompkins, E. Comelli

**Affiliations:** ^122.1^University of TorontoTorontoONCanada; ^122.2^Lallemand Health SolutionsMontréalQCCanada

*Background*. Fecal microRNA (miRNA), likely originating from intestinal cells, are an emerging biomarker of gut health. In humans, selected fecal miRNA were found to be associated with different stages of colorectal cancer. In animals, we and others found that the presence of the gut microbiota is associated with a distinctive intestinal miRNA signature. Moreover, the gut microbiota also modifies the intestinal miRNA response to pathogenic infection. It is likely that alteration of the gut microbiota composition, such as via antibiotics, will elicit a miRNA response in the host intestine which can be monitored in the feces.

*Aims*. To investigate the effect of antibiotic administration on the fecal miRNA signature.

*Methods*. Eleven healthy subjects received 875 mg of amoxicillin and 125 mg of clavulanic acid twice a day for 7 days. Total RNA was extracted from their feces, collected before and after antibiotic treatment, and used to profile the expression of 829 miRNA with the nCounter human version 3 miRNA expression assay (NanoString Technologies). Data were analyzed with nSolver*™* 2.5; statistics and hierarchical clustering were performed using R. Data were validated by quantitative PCR.

*Results*. 700 miRNA were detected in the feces at baseline. Of these, 48 were differentially expressed in response to antibiotics (*p* < 0.05), 7 being under-expressed and 41 being over-expressed (0.2 to 4.3 fold change). Samples obtained before and after antibiotic treatment clustered separately based on the expression profile of these 48 miRNA. MiR-378b, whose expression was decreased by antibiotics, is a member of the miR-378 family previously found to depend on the gut microbiota.

*Conclusions*. This study shows that antibiotic treatment, at a dosage previously shown to change the composition of the microbiota, alters the fecal miRNA signature. It is likely that this results from microbial dysbiosis. This study may help understand the role of the microbiota in diseases that are accompanied by altered miRNA expression and microbiota composition, including inflammatory bowel disease and colorectal cancer.

*Funding Agencies: *NSERC, Lallemand Health Solutions

# Sub article: A123: The Serotonergic Endocrine- Gut Microbiota Axis in Experimental Colitis

**Authors:** E. Denou, J. Ghia, H. Wang, J. Kim, M. Shajib, M. Shah, M. Surette, S. Collins, W. Khan

**Affiliations:** ^123.1^Farncombe Family Digestive Health Research InstituteMcMaster UniversityHamiltonONCanada; ^123.2^University of ManitobaWinnipegMBCanada

*Background*. Serotonin, also known as 5-hydroxytryptamine (5-HT), is predominantly secreted in the gut by enterochromaffin (EC) cells. Tryptophan hydroxylase 1 (Tph1) catalyzes the synthesis of 5-HT in EC cells, and we have recently shown that in* Tph1* ^−/−^ mice which have significantly reduced 5-HT in the gut exhibit reduced severity of colitis. Conversely, replenishing gut 5-HT increased the severity of colitis. Due to strategic location of EC cells in gut mucosa, we hypothesize that 5-HT contributes to gut homeostasis by influencing its microbial composition.

*Aims*. To elucidate the role of 5-HT in regulation of gut microbiota in the context of experimental colitis.

*Methods*. We used PCR amplification of the v3 region of the 16S rDNA gene sequences in combination with deep Illumina sequencing to assess the composition of fecal microbiota in* Tph1* ^−/−^ and heterozygous* Tph1* ^+/−^ mice housed in the same cage.* In vitro *growth curves of 13 isolated commensals in presence of various concentrations of 5-HT was measured by optical density at 650 nm. Adoptive transfer was done using cecal microbiota from* Tph1* ^+/−^ mice gavaged to* Tph1* ^−/−^ mice and vice-versa beginning 2 days before colitis induced by 5% DSS. The extent of DSS-colitis was assessed by investigating macroscopically and histological damage scores, myeloperoxidase (MPO) activity, C-reactive protein (CRP) and cytokines levels.

*Results*. We observed the following differences in microbial composition between* Tph1* ^−/−^ and* Tph1* ^+/−^ mice. Microbiota of* Tph1* ^−/−^ mice is characterized by prevalence of* Desulfovibrio* and lower relative abundance of* Parabacteroides*,* Erysipelotrichaceae *and* Turicibacter* compared to* Tph1* ^+/−^ microbiota. In a species-dependant manner, 5-HT at low concentration (below 0.01 mg/L) stimulates in vitro bacterial growth but carries out a bacteriostatic effect at higher concentration (from 1 mg/L). Transfer of microbiota from* Tph1* ^+/−^ to* Tph1* ^−/−^ mice prior to DSS colitis significantly increased the histological score, the levels of CRP, MPO, IL-1*β*, IL-6, and IL-17. In contrast* Tph1* ^+/−^ mice that received microbiota from* Tph1* ^−/−^ mice showed a significant decrease in these inflammatory markers.

*Conclusions*. These results demonstrate that 5-HT influences the microbial composition of the gut and alters susceptibility to DSS-colitis. These data generate novel information on the interaction between 5-HT and microbiota in relation to pathogenesis in colitis and may lead to new therapeutic strategies for gut inflammatory disorders.

*Funding Agencies: *CCC, CIHR

# Sub article: A124: MUC2 Mucin and Microbiota Play Distinct Roles in Conferring Innate Host Defense and Susceptibility to Colonic Injury

**Authors:** A. Leon-Coria, K. Chadee, F. Moreau, M. Surette

**Affiliations:** ^124.1^University of CalgaryCalgaryABCanada; ^124.2^McMaster UniversityHamiltonONCanada

*Background*. The human colon has approximately 10^14^ microorganisms that form the microbiota which plays a key role in digestive health and in the development of a balanced immune system. The intestinal epithelium is covered with bilayer MUC2 mucin that acts as a protective barrier and is constantly exposed to commensals and pathogenic microorganisms. Both dysbiosis (alteration in microbiota composition) and disruption of the mucus layers are associated with gastrointestinal pathological conditions such as IBS, IBD and colorectal cancer. However, the distinct contribution of the microbiota and the mucus barrier in the pathogenesis of colitis is not well understood.

*Aims*. (1) To determine whether shifts in microbiota can alter the susceptibility, onset, progression or recovery of colitis in* Muc2* ^+*/*+^ and* Muc2* ^−*/*−^ mice. (2) To quantify the distinct roles of an intact Muc2 barrier and the microbiota in susceptibility to colonic injury.

*Methods*. To minimize variations in microbiota only* Muc2* ^+*/*+^ and* Muc2* ^−*/*−^ littermates were used. To quantify the role of the microbiota in disease pathogenesis, animals were treated with a cocktail of antibiotic (Ab) to eliminate indigenous bacteria as revealed by llumina sequencing DNA analysis, and then fecal transplanted with littermate stool and susceptibility to dextran sulphate sodium (DSS) quantified. To determine early innate responses, untreated and Ab-treated* Muc2* ^+*/*+^ and* Muc2* ^−*/*−^ animals were infected with the colonic parasite* Entamoeba histolytica* (*Eh*) in closed colonic loops. Chemokines, cytokines and other markers were measured by qPCR and luminex. Intestinal permeability was assessed using FITC-dextran.

*Results*. DNA analysis showed that* Muc2* ^+*/*+^ and* Muc2* ^−*/*−^ mice shared similar phyla distribution regardless of their distinct phenotype. Surprisingly,* Muc2* ^+*/*+^ mice that received* Muc2* ^−*/*−^ microbiota were highly susceptible to DSS-induced colitis with increased colonic permeability, ulcerated lesions and loss of crypts architecture, increased inflammatory cellular infiltrate and mortality as compared to* Muc2* ^+*/*+^ receiving their own microbiota.* Muc2* ^−*/*−^ mice showed no increase/decrease in disease susceptibility receiving their own or* Muc2* ^+*/*+^ microbiota. In colonic loops,* Eh* in Ab-treated mice showed significant increase in watery secretions, luminal pro-inflammatory cytokines (IFN-*γ* and TNF-*α*) and chemokines (MCP-1, MIG, MIP-1a, MIP-1b and MIP-2) as compared to untreated controls.

*Conclusions*.* Muc2* ^−*/*−^ microbiota alone could confer increased susceptibility to DSS-induced colitis in* Muc* ^+*/*+^ mice with an intact Muc2 layer. Indigenous microbiota in* Muc2* ^−*/*−^ animals play an important role in mediating host protection against* Eh*-induced pro-inflammatory responses in the absence of an intact Muc2 layer.

*Funding Agencies: *CCC

# Sub article: A125: Human Microbiota Induces Barrier Homeostasis in Germ-Free Mice

**Authors:** C. Hayes, J. Jury, J. Dong, A. Naidoo, J. Libertucci, D. Bowdish, E. Verdu

**Affiliations:** ^125.1^McMaster UniversityHamiltonONCanada; ^125.2^McMaster UniversityBurlingtonONCanada

*Background*. The intestinal barrier involves various immune and permeability functions that are regulated throughout life by cytokines, diet, drugs, pathogens and their toxins. Commensal colonization induces antimicrobial peptide and IgA secretion in the gut; however, the kinetics for induction of functional aspects of the barrier that are key to maintaining intestinal homeostasis remain unclear.

*Aims*. Our aim was to investigate the sequence of immune and functional barrier changes induced by colonization of germ-free mice with human bacteria.

*Methods*. Adult male and female germ-free (GF) C57BL/6 mice were colonized by oral gavage with fecal slurry derived from a healthy human. At days 1, 7 and 21 following colonization, translocation of live bacteria to the spleen was assessed by culture, and NF*κ*B-SEAP reporter assays were performed to evaluate the presence and immunostimulatory capacity of serum lipopolysaccharide (LPS) and muramyl dipeptide (MDP). At the same time points, paracellular and transcellular permeability of the proximal colon were evaluated by Ussing chambers with ^51^Cr-EDTA and HRP probes, respectively, and RNA expression of intestinal barrier genes were quantified using a custom designed NanoString Elements panel. GF and conventionally raised specific pathogen free (SPF) mice were used as controls.

*Results*. At day 1 post-colonization, <10^4^ cfu/mL were detected in the spleen, and NF*κ*B activation by serum LPS and MDP was higher following colonization compared to GF mice. Paracellular permeability was increased to a level comparable to SPF mice by day 7 of colonization and sustained by day 21. No significant changes in transcellular permeability were observed post-colonization. RNA sequencing identified a number of expression changes of barrier functional, innate immune and neuronal signaling proteins; most notably,* Pparg *and* Gpbar1 *were decreased only at day 1 post-colonization, while* Nod2 *was increased at day 7 post-colonization compared to GF mice.

*Conclusions*. Upon colonization, a rapid increase in systemic exposure to bacteria and their products occurs, which may be attributable to diminished expression of* Nod2* and barrier fortifying proteins* Pparg *and* Gpbar1 *at day 1 post-colonization. Induction of paracellular permeability, comparable to that seen in conventional SPF mice, is observed by day 7 post-colonization, once initial systemic translocation is controlled. These findings suggest immune detection precedes functional barrier changes characteristic of a colonized status.

*Funding Agencies: *CCC

# Sub article: A126: IL-22 Inhibits IL-10 And IL-25 Production and Attenuates Helminth-Inducedsuppression of Colitis in Mice

**Authors:** J. Reyes, F. Lopes, M. Fernando, G. Leung, C. Matisz, A. Wang, D. McKay

**Affiliations:** ^126.1^University of CalgaryCalgaryABCanada; ^126.2^Uni. CalgaryCalgaryABCanada

*Background*. Awareness of the precise immunological basis of host-parasite interactions has the potential to reveal new approaches to cure and/or treat inflammatory disease. We have shown that mice infected with the tapeworm* Hymenolepis diminuta* (HD) developed significantly less severe dinitrobenzene sulphonic acid (DNBS)-induced colitis, that was accompanied by a TH2 response, presumably driven by IL-25, and was mediated, in part by IL-10.

*Aims*. In ongoing analyses of this helminth-mouse model system we assessed the effect of IL-22, a cytokine associated with anti-microbial responses in the gut, in HD-induced protection of DNBS-induced colitis.

*Methods*. Colitis was induced in wild-type (WT) and IL-22 knock-out (KO) deficient mice by DNBS (5 mg, ir) +/− HD infection (5 cysticercoids), and disease assessed 72 h later by colon length, weight loss, a cumulative disease activity score and histopathology. Cytokinelevels were assessed by q-PCR and ELISA. HD were cultured onthe small intestine murine epithelial cell line, IEC-4, and IL-25 was measured.

*Results*. IL-22KO HD-infected mice displayed a slightly delayed, but enhanced TH2 response as gauged by MLN and gut cytokines, and increased IL-10 and IL-25 production. In vitro studies showed that HD directly evoked IL-25 from gut epithelia and this was reduced by addition of recombinant IL-22. The IL-22 KO mice had less DNBS-induced colitis compared to WT mice, and when infected with HD and challenged with DNBS, IL-22 KO mice had negligible disease. Finally, anti-IL-25 antibody treatment of IL-22 KO (+/− HD) resulted in more severe DNBS-induced colitis.

*Conclusions*. IL-22 is identified as an endogenous inhibitor of helminth-elicited TH2 immunity in the gut (i.e. reciprocal IL-22-IL-25 signalling), which reduces the effectiveness of HD-induced protection against colitis. We speculate that helminth therapy for inflammatory disease, under specific conditions, could be enhanced by inhibition of IL-22.

*Funding Agencies: *CIHR, NSERC

# Sub article: A127: Culturing Techniques for Primary Intestinal Epithelial Cell Monolayers and Their Use in the Study of Enteric Infection and Immunity

**Authors:** M. Stahl, B. Vallance

**Affiliations:** ^127.1^University of British ColumbiaVancouverBCCanada; ^127.2^BC Children's HospitalVancouverBCCanada

*Background*. The tool of choice for studying the cellular mechanisms of intestinal epithelial cells* in vitro* has always been immortalized cell lines. These cells grow quickly and reliably, but can be problematic as they may not accurately represent the intestinal epithelium, and they cannot represent the genetic and phenotypic diversity found amongst human patients or animal models. More recently, the cultivation of intestinal organoids (enteroids) has become a popular tool for cultivating primary intestinal epithelial cells from either mouse or human origin. This technique centers on the ability to replicate the stem cell niche and induce intestinal stem cells to proliferate and differentiate* in vitro*. The resulting enteroids can be passaged almost indefinitely, and develop into a structure similar to the intestinal epithelium. Unfortunately, their structure and growth requirements make them complicated to use effectively for certain applications.

*Aims*. We aim to employ a novel method for cultivating primary intestinal epithelial cells, derived from enteroid cultures, as a means of studying host-pathogen interations and innate immune responses of the intestinal epithelium.

*Methods*. By dissociating the cells of an enteroid culture and plating them onto a matrigel-coated culture well, coverslip or hanging well insert, we can induce the cells to adhere and proliferate to form a confluent monolayer, similar to conventional cell cultures. We have begun using these primary colonic epithelial cell monolayers as a novel model for studying the interactions between enteric pathogens and intestinal epithelial cells. We infected cells with the common intestinal pathogen* Campylobacter jejuni* and have been comparing its interactions with primary mouse colonic epithelial cell monolayers against its interactions with conventional intestinal cell lines.

*Results*. There are both similarities and differences in patterns of cell adhesion and invasion between primary cells and conventional cell cultures, as well as the cytokine responses from the cells. Importantly, we have found that* C*.* jejuni* does not attack an epithelial monolayer evenly, but rather invades in large numbers through any breach in the cell juntions, while leaving an intact monolayer largely unscathed. Furthermore, when comparing monolayers derived from wild-type or mice deficient in Single Ig IL-1-related receptor (*Sigirr* ^−*/*−^), we found a substantial decrease in* C*.* jejuni* cell invasion, suggesting a role for Toll-like receptors in cell resistance to* C*.* jejuni* invasion.

*Conclusions*. We can successfully induce primary intestinal epithelial cells to grow into a confluent monolayer, and our current work with* C*.* jejuni* suggeats that they may be a useful tool in identifying novel pathogen-epithelial cell interactions.

*Funding Agencies: *CIHR, MSFHR

# Sub article: A128: Development of a Caenorhabditis Elegans Model to Study Mechanisms of Microbiota Mediated Protection against* Clostridium difficile*

**Authors:** T. Gerbaba, A. Moeez, I. Chin-Sang, E. Petrof

**Affiliations:** ^128.1^Gastrointestinal Diseases Research UnitDepartment of MedicineQueen's UniversityKingstonONCanada; ^128.2^Department of Biological SciencesQueen's UniversityKingstonONCanada

*Background*.* Clostridium difficile*, a toxin producing bacterium, is the leading cause of antibiotic-associated diarrhea and pseudomembranous colitis. By destroying the diversity of the microbiota, antibiotic treatment creates an environment conducive for* C*.* difficile* proliferation and toxin production, rendering the host susceptible to* C*.* difficile* infection. Although the importance of a healthy microbiota for defense against* C*.* difficile* infection is well established, the keystone protective species (and mechanisms of protection) remain poorly understood.

*Aims*. To develop a simple and high-throughput applicable model to study mechanisms of microbiota-mediated protection and to discover novel microbial therapeutics against* C*.* difficile *toxins.

*Methods*. Age synchronized L1 stage worms were grown to L4 stage by feeding* E*.* coli* OP50 in the presence or absence of antibiotics (Carbenicilin). L4 stage worms were treated with* C*.* difficile* toxin (TcdB) in a 96 well plate and worm survival was monitored. The effect of toxin treatment on the cytoskeletal structure of the intestine was determined by FITC-phalloidin staining. Toxin effects on gut barrier/integrity was determined by FITC-dextran and propidium iodide (PI) assays.

*Results*. Pre-exposure of worms to* E*.* coli *OP50 with antibiotics rendered the worms susceptible to TcdB, whereas worms not exposed to antibiotic remained resistant (*n* = 90; log rank test, *p* < 0.05). FITC-phalloidin staining of TcdB treated worms revealed destabilization of intestinal cytoskeletal structures and distension of the intestinal lumen. TcdB treatment also resulted in intestinal barrier breach as demonstrated by FITC-dextran assay. FITC-dextran was restricted to the gut lumen and was not seen in the pseudocoelom of controls, whereas TcdB treated worms showed leakage of FITC-dextran in to the pseudocoelom. PI remained restricted to the intestinal lumen in controls, whereas treatment with TcdB resulted in PI entry and intracellular staining of the intestinal cells. Co-treatment of worms with TcdB in the presence of secreted/excreted products of a consortium of 33 healthy human gut microbial isolates protected worms against the effect of TcdB on intestinal barrier function, integrity of intestinal cell plasma membrane, and survival, in antibiotic pretreated worms.

*Conclusions*. The developed model system would be useful to study mechanisms of microbiota-mediated protection and to discover novel microbial therapeutics against* C*.* difficile*.

*Funding Agencies: *John Alexander Stewart Fellowship; Women's Giving Circle

# Sub article: A129: Human Catestatin Treatment Results in Gut Microbiota Dysbiosis in Mice

**Authors:** M. Rabbi, P. Munyaka, N. Eissa, A. Khafipour, E. Khafipour, J. Ghia

**Affiliations:** ^129.1^University of ManitobaWinnipegMBCanada; ^129.2^Animal ScienceUniversity of ManitobaWinnipegMBCanada

*Background*. An increased ratio of Firmicutes/Bacteroidetes has been reported in inflammatory bowel diseases (IBD) or inflammatory bowel syndrome (IBS). Gut bacterial homeostasis can be regulated by epithelial antimicrobial peptides, such as Chromogranin-A (CgA)-derived peptides, released by enterochromaffin cells. Catestatin (CTS) derived from the C-terminal portion of CgA possess antimicrobial properties* in vitro, *but its effect on gut microbiota is unknown. We hypothesized that CTS injection will alter the trasnient gut microbiota and those colonized in the coloninc tissue.

*Aims*. To evaluate the* in vivo* impact of a CTS treatment on gut microbial composition and functional changes using a naive mouse model.

*Methods*. CTS (human CgA_352-372_, 1.5 mg/kg/day) or normal saline were administered intrarectally to C57BL/6 male mice (7 weeks of age) for 6 days and then sacrificed. DNA was extracted from feces and colonic mucosa associated microbiota (MAM) samples and the V3-V4 region of bacterial 16S rRNA was amplified and subjected to Illumina sequencing. Alpha-diversity was calculated using Chao 1, while beta-diversity was determined using weighted and unweighted UniFrac distances in QIIME. Differences at the genus level were determined using partial least squares discriminant analysis (PLS-DA), and phylogenetic investigation of communities by reconstruction of unobserved states (PICRUSt) was used to determine the functional capacity of bacterial community.

*Results*. CTS treatment did not change the bacterial alpha-diversity of microbiota in fecal and MAM samples, however, significantly altered the beta-diversity between the groups. CTS-treated mice demonstrated a lower relative abundance of Firmicutes (*P* < 0.001) associated to a higher abundance of Bacteroidetes (*P* < 0.05) in their feces, however, no significant changes at the phylum level were observed in the CTS-treated MAM samples. At lower taxonomic levels, in both fecal and MAM samples, the PLS-DA analysis revealed an association between specific taxa and the treatment. In particular but not limited,* Bifidobacterium, Bacteroides, Prevotella *and* Parabacteroides *were positively associated to the CTS-treated group. Differences in microbial functional pathways were detected at fecal and MAM samples, including but not limited to nitrogen, nicotinate, nicotinamide metabolism and cell division and ribosome biogenesis.

*Conclusions*. This study supports the hypothesis that CTS treatment alters the gut microbiota composition under physiological conditions. This might open new venues for CTS as a new line of antibiotic or as a therapeutic agent to restore the dysbiosis observed during the development of intestinal disorders.

*Funding Agencies: *CCC, CIHR

# Sub article: A130: Acute 2,4 Dinitrobenzensulfonic Acid (DNBS)-Induced Colitis Results in Gut Microbiota Dysbiosis in Mice

**Authors:** A. Khafipour, P. Munyaka, M. Rabbi, N. Eissa, E. Khafipour, J. Ghia

**Affiliations:** ^130.1^University of ManitobaWinnipegMBCanada; ^130.2^Animal ScienceUniversity of ManitobaWinnipegMBCanada

*Background*. The most widely used experimental model of Crohn's disease (CD) is the 2,4 dinitrobenzenesulfonic acid (DNBS)-induced colitis, which is developed by intrarectal (i.r.) administration of DNBS. Until recently, much attention has been given to the role of mucosal response to DNBS treatment, and only few studies investigated colonic microbiota in relation to DNBS. We hypothesized that i.r. treatment can modify both transient gut microbiota and those colonized in the colon tissue.

*Aims*. The aim of the study was to determine the microbial composition and functional changes in mice treated with DNBS.

*Methods*. C57Bl/6 mice received a solution of DNBS (4 mg/kg, i.r.) plus ethanol (30%) and control mice only PBS. Three days later confirmation of inflammation was evaluated clinically and by analysis of colonic tissue cytokine levels. From colon tissue and fecal samples, the V3-V4 region of bacterial 16S rRNA was amplified and subjected to Illumina sequencing. Alpha- and beta-diversities were calculated in QIIME and subjected to PROC MIXED analyses of SAS and PERMANOVA, respectively. Differences at the genus level were determined using partial least squares discriminant analysis. Functional capacity of bacterial community was predicted using open source software PICRUSt. Differences in inflammatory markers were tested using one-way ANOVA followed by multiple comparison post hoc analysis with significance at *P* < 0.05.

*Results*. Disease severity and IL-1*β* and IL-6 cytokines were increased in DNBS-treated versus vehicle mice. DNBS treatment reduced bacterial alpha-diversity and significantly altered the beta-diversity of microbiota. DNBS treatment also significantly reduced the abundance of several bacterial taxa including but not limited to lachnospiraceae and christensenaleace of Firmicutes phylum, Comomonadaceace from Proteobacteria phylum and increased the abundances of Bacteroidaceae and Prevotellaceae from Bacterioidetes phylum and Enterobacteriaceae of Proteobacteria phylum. Predicted functional capacity of bacterial community in both fecal and colon tissue samples was altered by DNBS treatment, including but not limited to lipopolysaccharide biosynthesis, aminobenzoated degradation, lysine degradation, and fatty acid metabolism.

*Conclusions*. Colitis in DNBS model was accompanied with the disruption of gut microbiota. While colitis development is driven by interplay between mucosal epithelial damage, an inflammatory response, and a dysbiotic microbiota, it is however, not clear if DNBS directly induces dysbiosis, or if microbiota dysbiosis occurs as a result of mucosal damage and inflammatory response.

*Funding Agencies: *CCC, CIHR

# Sub article: A131: NOD2^−/−^ Mice Show a Unique Response in Microbiota and Mucosal T Cell Responses after Neonatal Exposure to Antibiotics

**Authors:** A. Goethel, W. Turpin, S. Rouquier, G. Zanello, D. Philpott, K. Croitoru

**Affiliations:** ^131.1^University of TorontoTorontoONCanada; ^131.2^Mount Sinai HospitalTorontoONCanada

*Background*. Antibiotic exposure during early life is associated with development of auto-inflammatory diseases, including asthma, multiple sclerosis and Crohn's disease, although the mechanism remains unclear. Exposure to antibiotics and the resultant changes in gut microbiota during early life may lead to disruption of normal mucosal immune development, as this is strongly influenced by commensal microbes.

*Aims*. We hypothesized that antibiotic disruption of the microbiota during early life would have a prolonged impact on both gut microbiota community structure and T cell function within the intestinal tract, resulting in defective immune tolerance to the gut microbiota in a genetically susceptible host (*Nod2* ^−*/*−^ *mice*), leading to increased susceptibility to colitis.

*Methods*. Heterozygous Nod2 (+/−) breeders were used to generate litters of Nod2 +/+ (WT), +/− (HET) and −/− (KO) littermates, who all received the same foundational maternal microbiota. From birth, WT and* Nod2* ^−*/*−^ littermates received amoxicillin (200 mg/L) in the drinking water until weaning. Amoxicillin transfers through the dam's breast milk to the pups until day 14, when the pups begin to drink the water directly. Fecal samples collected at weaning were analyzed by targeted quantitative PCR (qPCR) and 16S ribosomal RNA sequencing for microbiota composition. At 8 weeks of age, mucosal lymphocyte populations of the small and large bowel were analyzed via flow cytometry.

*Results*. Neonatal amoxicillin treatment resulted in a significant shift of the gut microbial community structure, irrespective of Nod2 genotype or sex. The antibiotic-driven shift was associated with a significant reduction of Bifidobacterium and Lactobacillus. Neonatal antibiotics resulted in a significant reduction in serum IgA levels at 8 weeks of age. Phenotypes of small intestinal lamina propria lymphocyte (LPL) populations were not different in water-treated WT and* Nod2* ^−*/*−^ littermates. However, antibiotic-treated* Nod2* ^−*/*−^ littermates showed an increase in Foxp3^+^ and ROR*γ*t^+^ T cells.

*Conclusions*. Together, this suggests that neonatal antibiotic perturbation of the microbiota development alters Nod2 signalling in microbe-driven immune responses.

*Funding Agencies: *CAG, CIHR, OGS and Mount Sinai Hospital

# Sub article: A132: Colonic Abnormalities in Manitoban Children with* Helicobacter pylori *Gastritis

**Authors:** U. Banik, C. Stefanovici, J. Griffin, W. El-Matary

**Affiliations:** ^132.1^Section of Pediatric GastroenterologyUniversity of ManitobaWinnipegMBCanada; ^132.2^Department of PathologyUniversity of ManitobaWinnipegMBCanada; ^132.3^University of ManitobaWinnipegMBCanada

*Background*.* Helicobacter pylori* (*H*.* pylori*) is a pathogenic bacterium that can cause chronic gastritis. In Canada, Aboriginal population and first generation immigrants are most at risk for acquiring the organism. Association between* H*.* pylori* and colonic pathology is under-investigated.

*Aims*. To examine the prevalence and nature of colonic changes in children diagnosed with* H*.* pylori* gastritis in Manitoba.

*Methods*. A comprehensive retrospective chart review for all children (≤17 years) diagnosed with* H*.* pylori* gastritis from January 1996 to May 2015 at the Children's Hospital, Winnipeg, Manitoba, was conducted. The medical records of children with* H*.* pylori* gastritis who had colonoscopy were thoroughly examined. Patients' demographics, symptoms, laboratory findings, indications for colonoscopy and colonoscopic findings were documented.

*Results*. During the study period, 231 patients were found to have* H*.* pylori* gastritis. The mean age at diagnosis was 12.3 ± 4.1 years, 108 (46.6%) were girls. Of the 231 patients, 37 (16%) patients were found to have colonoscopy performed. 22 (59%) out of a total 37 children who had colonoscopy had significant endoscopic and histopathological findings on colonoscopy including polyposis and colitis. Males with colonic changes were diagnosed earlier compared to those without. No significant difference in colonic changes was found among children from different ethnic Backgrounds.

*Conclusions*. A significant number of pediatric patients with* H*.* pylori *gastritis in Manitoba had colonic pathologies. Aboriginal population and first generation immigrants are not at increased risk for having colonic changes in conjunction with* H*.* pylori*. Our study highlights a possible relationship between* H*.* pylori *gastritis and colonic changes in children and warrants properly planned prospective studies to confirm our results.

*Funding Agencies: *None

# Sub article: A133: *Heliobacter pylori* among Obese and Non-Obese Patients in Najran, Saudi Arabia: A Controlled Cross-Sectional Study

**Authors:** A. Al-Zubaidi, A. Alzobydi, H. Alghamdy, A. Alakwa, K. Aman, R. Kurian, S. Kassim, A. Alshahrani

**Affiliations:** ^133.1^King khalid HospitalNajranSaudi Arabia; ^133.2^National gaurd hospitalAhsaSaudi Arabia; ^133.3^Aden UniversityAdenYemen; ^133.4^Taibah universityAl-Madinah Al-MunawwarahSaudi Arabia; ^133.5^Najran UniversityNajranSaudi Arabia

*Background*. The association between* Helicobacter pylori* infection (HPI) and obesity is contested.

*Aims*. We aimed to enumerate and stratify by gender obese and non-obese patients with HPI.

*Methods*. This controlled cross-sectional study took place in the Department of Endoscopy of a central hospital that received all referrals in the Najran region of Saudi Arabia. A total of 340 obese Saudi patients (body mass index = 48.04) who had undergone diagnostic upper endoscopy before sleeve gastrectomy, were compared with 340 age and gender matched normal weight (body mass index = 23.13) control patients who had undergone diagnostic upper endoscopy for other reasons. Data collected included diagnosis of HPI based on histology of 2 biopsy specimens from gastric antrum and items related to socio-demographic characteristics of patients. Descriptive and bivariate data analysis was performed.

*Results*. Mean patients' age was 29.30 ± 8.02 years, and 55% were males. Obese patients presented with significantly more HPI than non-obese patients (66% versus 50%, *p* = 0.001). Both male and female obese patients presented with significantly HPI than non-obese patients (65% versus 45%, *p* = 0.0001; 69% versus 54%, *p* = 0.009, resp.). There was no statistically significant difference between males and females with respect to HPI among obese patients (64% versus 69%, *p* = 0.388) and among non-obese the differences was marginal (44% versus 52%, *p* = 0.061).

*Conclusions*. In this study, the significance of* Helicobacter pylori* infection in obese Saudi male and female patients who had undergone endoscopy compared with non-obese patients was demonstrated.

*Funding Agencies: *None

# Sub article: A134: Indications for Biliary Metal Stent Use and Stent Type Preference in a Large Prospective Multi-Centre Canadian Registry

**Authors:** G. Sandha, A. Roy, T. Maniere, J. Telford, N. Arya, R. Mohamed, J. Ellsmere, P. Kortan, A. Singh, R. Bailey

**Affiliations:** ^134.1^University of AlbertaEdmontonABCanada; ^134.2^Centre Hospitalier de l'Université de Montréal*-*Saint*-*Luc HospitalMontrealQCCanada; ^134.3^Hopital Charles Le MoyneSherbrookeQCCanada; ^134.4^Providence HealthSt. Paul's HospitalVancouverBCCanada; ^134.5^Oakville*-*Trafalgar Memorial HospitalOakvilleONCanada; ^134.6^University of CalgaryCalgaryABCanada; ^134.7^Queen Elizabeth II Health Sciences CenterHalifaxNSCanada; ^134.8^St. Michael's HospitalTorontoONCanada; ^134.9^Victoria General HospitalVictoriaBCCanada; ^134.10^Royal Alexandra HospitalEdmontonABCanada

*Background*. The clinical indications for using biliary self-expanding metal stents (SEMS) and the preferences for type of SEMS in a real-life setting are not well documented.

*Aims*. The goal of the study is to document practice patterns pertaining to indications for biliary SEMS placement and stent type selection per standard of practice across Canadian hospitals.

*Methods*. This is a prospective, multi-centre and open-label study at 10 centres across Canada. Consecutive data was collected for a commonly used biliary SEMS in uncovered (UC), partially covered (PC) and fully covered (FC) versions (WallFlex®, Boston Scientific Corporation, Marlborough, MA, USA).

*Results*. To date 150 patients have been enrolled at 10 centres. Indications were palliation of malignant biliary obstruction (Group A) in 93/150 (62%), pre-operative drainage of malignant biliary obstruction with or without neoadjuvant therapy (Group B) in 21/150 (14%), treatment of a benign biliary stricture (Group C) in 21/150 (14%) and other indications (Group D) in 15/150 (10%). Group D indications were: EUS-guided cystgastrostomy for pancreatic fluid collections (7), EUS-guided cholecystoduodenostomy for recurring cholecystitis in non-surgical patients (2), refractory CBD stone disease with impacted stone in non-surgical candidate (2), intraductal papillary mucinous neoplasm (1), indeterminate biliary stricture (1), prevention of post-sphincterotomy bleeding (1) and extrinsic biliary compression (1). Stent type preferences are detailed in Table 21. Adverse events were seen in 2/150 (1.3%) and included acute pancreatitis (1) in Group C and recurrent cholangitis due to food impaction (1) in Group A, both in a FC stent.

*Conclusions*. For palliation of malignant biliary obstruction, UC stents seem to be preferred twice as commonly as FC stents. For benign biliary stricture treatment, FC stents are uniformly selected. PC stents are rarely used. No significant differences in adverse events are seen between UC and FC stents.

*Funding Agencies: *This study is sponsored by Boston Scientific Corporation, Marlborough, MA, USA

# Sub article: A135: Eus Increases the Identification of Unresectable Disease among Adults with Pancreatic Adenocarcinoma: A Meta-Analysis

**Authors:** P. James, Z. Meng, M. Zhang, P. Belletrutti, R. Mohamed, W. Ghali, D. Roberts, G. Martel, S. Heitman

**Affiliations:** ^135.1^Department of MedicineUniversity of OttawaOttawaONCanada; ^135.2^Department of Medicine and Community Health SciencesUniversity of CalgaryCalgaryABCanada; ^135.3^Department of SurgeryUniversity of CalgaryCalgaryABCanada; ^135.4^Department of SurgeryUniversity of OttawaOttawaONCanada

*Background*. Although preoperative endoscopic ultrasound (EUS) is increasingly being used among adults with pancreatic adenocarcinoma, it remains unclear if this procedure influences surgical management.

*Aims*. This systematic review sought to determine if preoperative EUS evaluation is associated with the identification of unresectable disease among adults with pancreatic adenocarcinoma.

*Methods*. We searched MEDLINE, EMBASE, bibliographies of included articles and conference proceedings for studies reporting original data regarding surgical management among patients with pancreatic adenocarcinoma. Our main outcome was the incremental benefit of EUS for the identification of unresectable disease (IB_EUS_). The pooled IB_EUS_ was calculated using random effects models. Heterogeneity was explored using stratified meta-analysis and meta-regression.

*Results*. Among the 4,505 citations identified, we included 9 cohort studies that examined the identification of unresectable disease (*n* = 1,030). Random effects meta-analysis suggested that EUS alone identified unresectable disease in 17% of patients (95% confidence interval (CI), 11–25%) who appeared to have resectable disease on CT scan. This number increased in studies where EUS was the only modality used after CT scan (24%, 95% CI 16–39%), and decreased when extensive imaging (CT, abdominal ultrasound and MRI) were performed before EUS (5%, 95% CI 3–11%). Among those studies that considered portal or mesenteric vein invasion as potentially resectable, EUS alone was able to identify unresectable disease in 15% of patients (95% CI 9–27%) after a CT scan was performed.

*Conclusions*. Existing evidence suggests that EUS evaluation is associated with increased identification of unresectable disease among adults with pancreatic adenocarcinoma.

*Funding Agencies: *CAG, CIHR, Alberta Innovates: Health Solutions

# Sub article: A136: Repeat Endoscopic Ultrasound-Guided Fine Needle Aspiration in Patients with Suspected Pancreatic Cancer: Diagnostic Yield and Associated Change in Access to Appropriate Care

**Authors:** D. Stanger, R. Enns, J. Telford, E. Lam

**Affiliations:** ^136.1^University of British ColumbiaVancouverBCCanada; ^136.2^St. PaulVancouverBCCanada; ^136.3^Univ. of CaliforniaVancouverBCCanada

*Background*. Pancreatic malignancy carries a poor prognosis. In order to direct appropriate therapy towards a potential pancreatic malignancy, a tissue diagnosis is often required.

Endoscopic ultrasound (EUS) is a contemporary technique that uses both endoscopy and ultrasound (US) to image the pancreas. Studies have demonstrated EUS is more sensitive than both transabdominal US and CT scan in detecting pancreatic solid masses.

Despite the fact that EUS-FNA has demonstrated instrumental diagnostic utility, recent literature has determined inconclusive diagnosis at initial EUS-FNA.

To obtain appropriate preoperative chemotherapy, definitive Class 5 cytology is required. It is important to clarify how we obtain appropriate diagnosis on those patients whose initial diagnosis is not easily obtained with the first EUS-FNA.

*Aims*

*Primary Objective*. To determine the cumulative yield of repeat EUS-FNA in patients with suspected pancreatic cancer.

*Secondary Objectives*. To determine how a second biopsy influences access to next steps in management.

To determine complications associated with a second EUS-FNA.

*Methods*. A retrospective cohort study was conducted evaluating the yield of repeat EUS-FNA in determining a cytological diagnosis in patients who had undergone an initial but inconclusive EUS-FNA for pancreatic lesions suspicious for malignancy.

Patients were selected from a database at one tertiary university-based referral centre for pancreatico-biliary disorders during the period of 2007–2014.

All patients included in the study underwent repeat EUS-FNA for investigation of a pancreatic mass.

*Results*. 45 repeat EUS-FNA procedures were performed.

Of the 45 repeat procedures, the cumulative yield for diagnosing pancreatic adenocarcioma was 17.8%.

22% of patients who underwent repeat EUS-FNA received treatment including either surgery, radiotherapy or chemotherapy. Not all of these patients had definitive cytological diagnoses of pancreatic adenocarcinoma.

The complication rate associated with repeat EUS-FNA was 6.7%. All complications were mild and not life-threatening.

The mean time from referral to 1st FNA, second FNA and inter-FNAs were 24.8 days, 67.8 days and 43.0 days, respectively.

*Conclusions*. Pancreatic biopsies often require a repeat procedure for diagnostic certainty. The cumulative yield of repeat EUS-FNA for diagnosing pancreatic adenocarcioma was found to be 17.8% (8/45). Repeat EUS-FNA facilitated access to care as 22% of patients who underwent repeat FNA were treated with either chemotherapy, radiotherapy or surgery. Complications from a second procedure were relatively common but were not severe.

*Funding Agencies: *None

# Sub article: A137: Comparison of a Novel Side Port Needle with a Conventional End Port Needle in Eus-Guided FNA of Solid Lesions

**Authors:** J. Tan, B. Tessier-Cloutier, D. Schaeffer, F. Donnellan

**Affiliations:** ^137.1^Vancouver General HospitalVancouverBCCanada; ^137.2^University of British ColumbiaVancouverBCCanada

*Background*. Endoscopic ultrasound (EUS) guided fine needle aspiration (FNA) plays an integral role in the diagnosis of pancreas and stomach solid lesions. The conventional needle used for EUS guided FNA has a single port at the end of the needle. Diagnostic accuracies rarely exceed 90% resulting in need for repeat EUS. The development of an additional novel side port needle with its “cheese-like" grating effect during movement of the needle in addition to the end needle port carries a theoretical advantage of increasing cellular acquisition thereby improving diagnostic yield.

*Aims*. To compare the sample cellularity obtained with both EUS guided FNA needles.

*Methods*. This randomized prospective study was carried out at Vancouver General Hospital Canada where competent adults undergoing EUS for FNA of a lesion seen on imaging were recruited. All consented patients were randomly allocated to either the conventional end port needle (Boston 22 G) or the novel side port needle (Olympus 22 G). Two consecutive passes were made using the allocated needle with tissue acquired sent into separate cytology containers labeled A and B. This FNA process was repeated using the other study needle for the same patient on the same day labeled C and D. FNA technique was standardized for all passes ie. Suction only. Pathologists were blinded to the needle used. Scoring for cytology cellularity were as follows: 0 for 0%, 1 for <5%, 2 for 5–30%, 3 for 30–60% and 4 for >60%. Scoring of core tissue acquired were defined as 0 for no cell cluster >0.5 mm, 1 for cell cluster 0.5 mm to 1 mm and 2 for cluster >1 mm.

*Results*. 20 patients were recruited between May 2014 and June 2015. 18 were referred for pancreatic mass with remaining 2 patients for gastric mass. The median age was 64.5 years with 12 males and 8 females. A total of 40 FNA cytology specimens were collected and included in the final analysis. (Table 22).

There was no difference in the total cytology score (92 versus 88; *p* = 0.74) or the cytology score per specimen (2.3 versus 2.2; *p* = 1.0) between the Boston and Olympus needles respectively.

There was a greater number of core specimens obtained with the Olympus needle (47.5% versus 37.5%; *p* = 0.05).

*Conclusions*. Our study demonstrates that a novel side port needle has no advantage over a conventional end port needle in terms of sample cellularity obtained with EUS guided FNA of solid lesions.

*Funding Agencies: *This study was kindly supported by a grant from Pancreas Centre BC

# Sub article: A138: Clinical, Endoscopic, and Cytopathologic Determinants of Non-Diagnostic Endoscopic Ultrasound Guided Fine Needle Aspiration in Solid Pancreatic Masses

**Authors:** M. Alfawaz, M. Sey, A. AlNasser, N. Hussain, M. Weir, M. Joseph, B. Yan

**Affiliations:** ^138.1^Western UniversityLondonONCanada

*Background*. Endoscopic ultrasound guided fine needle aspiration (EUS-FNA) diagnostic yield in solid pancreatic masses should be approximately 75% based on previously published studies. A recent quality improvement study of our EUS-FNA results for pancreatic masses revealed non-diangnostic results in 47% of cases, despite having rapid on-site evaluation (ROSE). This study was completed to determine reasons for low diagnostic EUS FNA in solid pancreatic masses.

*Aims*. The aim of this study was to determine the clinical, procedural, and cytopathologic features that predict a non-diagnostic EUS-FNA for a pancreatic mass.

*Methods*. Retrospective chart review of all EUS-FNA cases performed for pancreatic masses between January 2010 and Dec 31, 2014. Predictors of a non-diagnostic EUS-FNA including patient related risk factors for pancreatic cancer, imaging characteristics, tumor marker, EUS-FNA procedural factors, and ROSE evaluations were recorded. Cases were considered diagnostic if their cytopathology were reported as either (1) positive for a malignancy, or (2) negative for a malignancy in the setting of sufficient cellularity. Cases were deemed non-diagnostic if cytopathology were reported as: (1) suspicious for malignancy, (2) atypical, (3) indeterminate, or (4) insufficient. Potential predictors of non-diagnostic EUS-FNA were assessed using univariate and multivariate logistic regression modeling.

*Results*. A total of 254 pancreatic masses were included in this study. One hundred sixty were in the head of the pancreas, 61 in the body, and 15 in the tail, 1 in the uncinate, and 8 not reported.

Of these lesions, 103 were diagnostic and 142 non-diagnostic. No significant patient clinical factors predicted non-diagnostic FNA. The only statistically significant determinant for non-diagnostic FNA was mass location in the head of the pancreas.

On multivariate analysis, the odds ratio for a non-diagnostic specimen in the head compared to elsewhere in the pancreas else was 2.6 (*p* = 0.007).

EUS procedural factors (including needle size, number of passes, year of procedure, physician and trainee involvement) did not affect probability of diagnostic specimen. Non-diagnostic samples were not associated with any particular cytopathologist.

*Conclusions*. Lesions in the head of the pancreas were associated with a higher non-diagnostic EUS FNA rate compared to lesions elsewhere in the pancreas. The reason for this requires further study. Efforts to optimise sampling and interpretation of pancreatic head lesions should be a focus of quality improvement programs in centers with low diagnostic rates in EUS FNA.

*Funding Agencies: *None

# Sub article: A139: A Prospective, Randomized Trial on the Timing of Rectal Indomethacin Administration for the Prevention of Post-ERCP Pancreatitis: A Preliminary Report

**Authors:** S. Kenshil, P. D'Souza, R. Sultanian, G. Sandha, V. Bain, R. Hundal, M. Laghari, A. Montano-Loza, S. Zepeda-Gomez

**Affiliations:** ^139.1^University of AlbertaEdmontonABCanada

*Background*. Rectal administration of indomethacin has been shown to reduce the risk of post-ERCP pancreatitis. Most of the studies have been done with administration of indomethacin immediately after the procedure. There have been no comparative studies about peri-procedural timing of indomethacin administration.

*Aims*. To compare the efficacy of pre-ERCP versus post-ERCP administration of rectal indomethacin in preventing the occurrence of post-ERCP pancreatitis.

*Methods*. All patients undergoing ERCP were randomized to receive 100 mg of rectal indomethacin either before (Group 1) or after (Group 2) the procedure. Patients with previous sphincterotomy, hypersensitivity to NSAIDs, rectal disease, renal failure, coagulopathy, active peptic ulcer disease, pregnancy, breastfeeding or acute pancreatitis were not eligible to be included. Lipase levels were collected two to four hours post-procedure. Patients were also evaluated clinically and contacted 48 hours later to determine if they had symptoms suggestive of pancreatitis. Patients who met high-risk criteria for development of post-ERCP pancreatitis were analyzed as a subgroup.

*Results*. A total of 165 patients have been included in the study (99 females (60%), mean age 52 (range 20–91) years). Eighty patients were included in group 1 and 85 in group 2. The most common indication for ERCP was choledocolithiasis (73.9%). Technical success was achieved in 95.2% of patients (157/165). Post-ERCP pancreatitis occurred in 7.3% (12/165) of all cases; 7/80 in group 1 (8.75%) versus 5/85 (5.88%) in group 2 (*p* = 0.341). Sixty-two patients (37.6%) were classified as high-risk for post-ERCP pancreatitis. A sub analysis performed revealed that pancreatitis occurred in 9 patients categorized as high-risk in comparison to 3 in patients categorized as non-high risk (*p* = 0.007). Amongst the high-risk patients, 5/21 of group 1 and 4/34 of group 2 developed pancreatitis (*p* = 0.211). There were no cases of severe pancreatitis.

*Conclusions*. Our preliminary data suggests that the timing of indomethacin administration does not influence the rate of post-ERCP pancreatitis.

*Funding Agencies: *None

# Sub article: A140: Optimizing the Diagnostic Yield of EUS-FNA for Solid Pancreatic Lesions: A Single-Centre Quality Assurance Study

**Authors:** M. Abunassar, A. Chatterjee, B. Dube, C. Marginean, G. Martel, S. Murthy, A. Rostom, C. Dube, P. James

**Affiliations:** ^140.1^The Ottawa HospitalDepartment of MedicineDivision of GastroenterologyOttawaONCanada; ^140.2^University of Ottawa/OHRIOttawaONCanada; ^140.3^The Ottawa HospitalDepartment of PathologyOttawaONCanada; ^140.4^The Ottawa HospitalHPB SurgeryOttawaONCanada

*Background*. Endoscopic ultrasound-guided fine needle aspiration (EUS-FNA) is a safe and effective procedure for the investigation of pancreatic masses. Improving EUS-FNA diagnostic yield will reduce the necessity for repeat procedures, thereby reducing risk to patients and resource use.

*Aims*. To examine factors associated with EUS-FNA diagnostic yield at our centre.

*Methods*. We performed a retrospective chart review of EUS-FNA procedures performed for the sampling of solid pancreatic lesions between September 1st 2009 to August 31st 2015 at The Ottawa Hospital. Rapid on-site evaluation (ROSE) for EUS-FNA was introduced in September 2010. Data regarding patient demographics (age and sex), lesion location, procedure details (endoscopist, FNA needle gauge, suction technique, number of passes) and the reviewing pathologist were collected. In addition to descriptive statistics, univariate and multivariable analyses were performed to determine factors associated with diagnostic yield.

*Results*. 350 EUS-FNAs for solid pancreatic lesions were examined by chart review. 288 (82%) of the procedures involved ROSE. The median patient age was 66 (interquartile range (IQR) 57–76) years and 56% were female. The overall EUS-FNA diagnostic yield was 81%. The diagnostic yield by the following factors were observed: patient sex (male 78%, female 84%), endoscopist (A 81% versus B 82%), lesion location (head 84%, body 78%, tail 74%), needle gauge (g) (19 g 67%, 22 g 82%, 25 g 80%), and number of FNA passes performed (one 50%, two 70%, three 84%, four 79%, five 80%, six 82%). The diagnostic yield with ROSE was 81% compared to 75% without ROSE. 11 pathologists were involved in the EUS cytopathology review, with a wide range in the number of cases reviewed by each pathologist (from 1 to 68 cases) and in their diagnostic yield (from 67% to 93%). No single factor was found to be significantly (*p* < 0.05) associated with diagnostic yield in univariate or multivariate analyses.

*Limitations*. This was a retrospective study. Not all EUS-FNA cases have been captured to date.

*Conclusions*. Although our overall diagnostic yield is comparable to what is reported in the literature, there is an opportunity for improvement. Multidisciplinary FNA Cytopathology rounds have begun at The Ottawa Hospital with an aim to optimize at optimizing specimen acquisition, processing and evaluation.

*Funding Agencies: *The Ottawa Hospital Department of Medicine Patient Safety and Quality Research Grant

# Sub article: A141: Single-Operator Cholangioscopy Is More Cost-Effective Than Bile Duct Exploration for Management of Difficult Common Bile Duct Stones after Failed Conventional Ercp

**Authors:** J. Sandha, R. Ennis-Davis, G. Sandha

**Affiliations:** ^141.1^University of AlbertaEdmontonABCanada

*Background*. Common bile duct (CBD) stones are currently managed by ERCP with sphincterotomy and stone extraction with a balloon catheter or basket. However, some stones are difficult to extract by conventional means. These include multiple, large, impacted, or faceted stones, or those proximal to a stricture. Cholangioscopy with electro-hydraulic lithotripsy (EHL) is a modality to treat these difficult stones.

*Aims*. The aim of this study was to describe the clinical efficacy of a single-operator cholangioscopy system (SpyGlass*™*, Boston Scientific Corporation, Marlborough, MA, USA) for difficult stones and discuss possible cost savings by avoiding surgical intervention.

*Methods*. We performed a retrospective chart review of all patients referred for SpyGlass cholangioscopy with EHL for difficult stones. Clinical efficacy was defined as the successful clearance of the CBD of all stones. The total cost (based on Alberta Health Services reimbursement codes) was calculated by adding all costs associated with cholangioscopy, including any subsequent procedures, surgery, hospital stay, or treatment of any complications. This cost was compared with the projected cost of open and laparoscopic CBD exploration (OCBDE, LCBDE).

*Results*. A total of 51 patients with difficult CBD stones (35 female, median age 68 ± 16.4 years (range 30–88 years)) with 108 prior ERCPs (average 2.1/patient) were referred. They underwent 58 SpyGlass cholangioscopy ERCPs and 7 additional ERCPs (average 1.3/patient). The average procedure time was 67 minutes (range 24–124 minutes). The CBD was successfully cleared in 47/51 patients (93% clinical efficacy). Minor complications were seen in 7 patients (14%). These included mild EHL-induced CBD wall trauma in 4 patients, wire-induced cystic duct stump leak in 1 patient, mild post-ERCP pancreatitis in 1 patient and mild mucosal tearing at gastro-esophageal junction during extraction of a plastic stent in 1 patient. The average cost of all procedures was $4550. This compares to a projected cost of $7766 and $6175 for OCBDE and LCBDE, respectively. The average cost saving per patient using SpyGlass cholangioscopy instead of OCBDE or LCBDE was $3216 and $1625, respectively. The published rate of complications (bile leak, hemorrhage, and abscess) for CBDE is 3.2%. Treatment of these complications added $4977, $5216, and $3701 to the cost of CBDE.

*Conclusions*. Single-operator SpyGlass cholangioscopy with EHL is highly effective for the treatment of difficult CBD stones. By adopting this modality as primary treatment for these difficult stones, significant cost savings may be realised by avoiding surgical intervention.

*Funding Agencies: *None

# Sub article: A142: Double-Baloon Endoscopic Retrograde Cholangiopancreatography in Patients with Surgically Altered Anatomy: A Single Center Experience

**Authors:** J. Nilsson, A. Montano-Loza, S. Zepeda-Gomez

**Affiliations:** ^142.1^University of AlbertaEdmontonABCanada

*Background*. Balloon assisted enteroscopy has improved our ability to perform endoscopic retrograde cholangiopancreatography (ERCP) in patients with surgically altered anatomy. We reviewed the experience with double-balloon ERCP (DBE-ERCP) in patients with altered anatomy and suspicion of biliary obstruction in a tertiary center.

*Aims*. To assess procedure indications, rates of success and procedural related complications with DBE-ERCP.

*Methods*. Retrospective analysis of all patients who underwent DBE-ERCP at the University of Alberta hospital between August 2011 and September 2015.

*Results*. A total of 57 DBE-ERCPs were performed in 28 patients (16 males) with a mean age of 51 ± 19 years (range: 20–81) using a short-type double balloon enteroscope. Twenty-seven patients had a Roux-en-Y reconstruction (25 hepatico-jejunostomies) and one patient had a prior Billroth-II gastro-jejunostomy. There were 19 patients that had previous liver transplantation (9 cadaveric, 10 living donor).

Mean time from surgery to the first DBE-ERCP was significantly lower in liver transplant patients compared to other surgeries (1100 ± 1466 versus 3950 ± 3826 days, (*p* = 0.01)). There was a trend to earlier DBE-ERCP in living related versus cadaveric transplants (1826 ± 1907 versus 519 ± 619 (*p* = 0.06)).

The main indications for procedures were suspicion of stricture at the hepatico-jejunostomy (*n* = 25 (44%)), recurrent cholangitis (*n* = 21 (37%)) and stent retrieval (*n* = 8 (14%)). Therapeutic maneuvers included: stricture dilation (*n* = 31), extraction of stones (*n* = 10), stent placement (*n* = 10) and stent retrieval (*n* = 8). The hepatico-jejunostomy or major papilla was reached in 46 of 57 procedures (81%). Bile duct cannulation was successful in 40 of 46 procedures (87%).

The mean number of procedures per patient was 2 ± 1.5 (range: 1–7 procedures). The number of procedures was higher in those with liver transplantation compared to other surgeries (mean: 2.5 ± 1.7 versus 1.3 ± 0.48 (*p* = 0.04)). There were two patients with mild cholangitis that resolved with intravenous antibiotic therapy.

Fourteen patients required stenting and dilation of the hepatico-jejunostomy. No subsequent intervention was required in ten of these patients after a mean of 3.1 ± 1.9 (range 1–7) procedures. In 4 patients, subsequent percutaneous drainage (PTC) was required for failure of endoscopic therapy, mean time to PTC was 136 days ±104 (30–274).

*Conclusions*. DBE-ERCP allows for successful therapy in patients with surgically altered anatomy of the upper-GI tract. Our single center study suggests this is a safe, and effective first line option at managing post-surgical biliary obstruction/strictures, however more than one session is generally required to achieve good outcomes.

*Funding Agencies: *None

# Sub article: A143: EUS-Guided Cystgastrostomy Using a Self-Expanding Metal Stent Is a More Cost-Effective Strategy for the Treatment of Pancreatic Fluid Collections

**Authors:** S. Kenshil, C. Teshima, P. D'Souza, G. Sandha

**Affiliations:** ^143.1^University of AlbertaEdmontonABCanada; ^143.2^St. Michael's HospitalTorontoONCanada

*Background*. EUS-guided cystgastrostomy (EUS-CG) has become the mainstay for endoscopic treatment of symptomatic pancreatic fluid collections (PFCs). The traditional approach (Approach I) of inserting multiple double-pigtail plastic stents with sequential dilation of the tract with or without naso-cystic (NC) irrigation is successful but requires repeated endoscopic intervention. The clinical utility of newer approaches utilizing a fully covered expandable metal stent (FCEMS, Approach II) is less well understood.

*Aims*. Our aim is to compare the clinical efficacy and cost impact of these approaches in the management of PFCs.

*Methods*. We performed a retrospective chart review of patients that underwent EUS-CG for PFCs. Clinical efficacy was defined as symptomatic improvement and cyst resolution on cross-sectional imaging. Follow up was until stent removal. The total cost of each procedure was calculated based on Alberta Health Services reimbursement codes for each EUS-CG (including anesthesia and radiology costs), all subsequent procedures and hospital length of stay (HLOS) until cyst resolution was confirmed. The clinical efficacy and average cost of the two approaches was then compared.

*Results*. Between 11/2010 and 10/2015, 10 patients underwent Approach I and 5 patients underwent Approach II. Cyst resolution was documented in all cases with a mean follow up of 304 days in Approach I (range 86–628 days) and 64 days in Approach II (range 3–124 days). Patients in Approach I had a mean of 3.7 endoscopic interventions (range 1–8) and 11.9 days of HLOS (range 0–33 days) compared with 1 endoscopic intervention and 2.4 days (range 0–4 days) in Approach II, respectively. Complications were seen in 5/10 patients (50%) in Approach I (4 patients with pus developing in the cyst space after the index procedure requiring prolonged hospital stay and repeated NC irrigation and antibiotic treatment. One of these patients and another patient had bleeding from the CG site after the index procedure requiring blood transfusion) compared with 1/5 patients (20%) in Approach II (ER visit 6 days after the index procedure with pain and increased c-reactive protein but managed with outpatient antibiotic therapy). In Approach I, 3/10 patients received hydrogen peroxide flushing compared with none in Approach II. The average cost of Approach I was $18,413 compared with $6,151 for Approach II.

*Conclusions*. Despite the increased upfront cost of metal stents, the use of FCEMS for EUS-CG in the treatment of PFCs results in significant overall cost savings by reducing the need for multiple endoscopic re-interventions and drainage procedures.

*Funding Agencies: *None

# Sub article: A144: EUS-Guided Cholecystenterostomy Is Effective in the Management of High-Risk Cases of Acute Cholecystitis

**Authors:** S. Kenshil, G. Sandha

**Affiliations:** ^144.1^University of AlbertaEdmontonABCanada

*Background*. Acute cholecystitis is a common clinical entity and optimal management is open or laparoscopic cholecystectomy. For high-risk patients that are not surgical candidates, conservative management includes antibiotics and percutaneous cholecystostomy (PC). EUS-guided cholecystenterostomy (EUS-CE) is an emerging alternative to PC.

*Aims*. We describe two high-risk patients with acute cholecystitis deemed not to be surgical candidates managed with EUS-CE.

*Methods*. Using a linear array echoendoscope (Olympus America, Center Valley, PA) and fluoroscopic guidance, a trans-duodenal puncture of the gallbladder was performed with a 19-gauge FNA needle (Slimline Expect®, Boston Scientific, Marlborough, MA). A 0.035-inch guidewire (Jagwire®, Boston Scientific, Marlborough, MA) was then coiled within the gallbladder following which a fistula was created using a 10 French (Fr) cystotome (Cook Medical, Bloomington, IN). A fully covered 10 mm wide × 40 mm long metal biliary stent (Wallflex, Boston Scientific, Marlborough, MA) was then inserted to create the CE. After that a 7 Fr, 4 cm long double-pigtail plastic biliary stent (Cook Medical, Bloomington, IN) was positioned within the metal stent so as to minimize any risk of migration.

*Results*. EUS-CE was performed on 2 patients for conservative management of their acute cholecystitis. Both patients were deemed non-surgical candidates by the surgical team. Patient 1, a 58 year-old man with decompensated cirrhosis, was admitted to the ICU with recurring bacterial peritonitis thought to be secondary to translocation of bacteria from his gallbladder as a result of his repeated episodes of calculus cholecystitis. Patient 2, an 83 year-old man with multiple comorbidities, including a recent acute coronary syndrome, had multiple admissions secondary to repeat episodes of acute cholecystitis. As a result of his repeat courses of antibiotics, he developed refractory* Clostridium difficile* colitis. Both patients underwent successful EUS-CE with drainage of the gallbladder through the stents documented endoscopically. Both patients were subsequently discharged home from the hospital. Neither patient has required repeat admission for acute cholecystitis or repeat interventions as a result of the EUS-CE.

*Conclusions*. EUS-CE is an effective treatment for acute cholecystitis in high-risk patients that are not fit for surgery. It is less invasive and has better patient acceptance than PC. We advocate an increasing role for EUS-CE in the non-surgical management of acute cholecystitis.

*Funding Agencies: *None

# Sub article: A145: Diagnostic Yield of Endoscopic Ultrasound Guided Fine Needle Aspiration versus Fine Needle Biopsy for Solid Lesions

**Authors:** A. Kayal, C. Chan, M. Alsahafi, A. Weiss, M. Byrne, D. Schaeffer, F. Donnellan

**Affiliations:** ^145.1^Division of GastroenterologyVancouver General HospitalUniversity of British ColumbiaVancouverBCCanada; ^145.2^University of British ColumbiaVancouverBCCanada; ^145.3^Department of Anatomical PathologyVancouver General HospitalUniversity of British ColumbiaVancouverBCCanada

*Background*. Endoscopic ultrasound guided fine needle aspiration (EUS-FNA) is the standard technique for obtaining tissue samples. The Sharkcore Needle (Covidien) is a new fine biopsy needle (FNB) for obtaining core tissue at time of EUS.

*Aims*. To compare the diagnostic yield of a conventional EUS FNA needle with a new EUS FNB needle for solid lesions in close proximity to the upper GI tract.

*Methods*. A retrospective study of patients who underwent EUS for tissue acquisition of solid lesions using both a conventional FNA needle (Boston Scientific) and a novel FNB needle (Sharkcore/Covidien) in the same session between February and June 2015. Two passes were made with the FNA needle using a standard EUS technique (no stylet, with suction). Two passes were also made with the FNB needle using a slow pull technique on the first pass and suction on the second pass. All were examined by a GI pathologist for neoplasia, diagnostic or non-diagnostic. Diagnostic yield was calculated based on a confirmed diagnosis by EUS sampling or surgically resected specimen or a presumed diagnosis by radiological imaging and overall clinical picture.

*Results*. 21 patients were included in the study. Mean age was 58.2 and 8 were male (38%). 11 (52.4%) had a pancreatic mass while the rest included both gastric and duodenal subepithelial tumors, and mediastinal and intra-abdominal lesions.

Using the FNA method, in 18 out of 21 (85%) a diagnosis was made compared to 15 out of 21 (71.4%) using FNB technique. This was not statistically significant with a *p* value of 0.45 based on Fischer's exact test. Combining both methods 19 out of 21 (90.5%) had a diagnostic sample.

*Conclusions*. EUS-FNB does not appear to increase the diagnostic yield compared to EUS-FNA. However, combining both techniques may increase this yield.

*Funding Agencies: *None

# Sub article: A146: Drug Associated Acute Pancreatitis Secondary to Apixaban: A Case Report

**Authors:** H. Azhari, C. Ma, P. Kumar

**Affiliations:** ^146.1^University of CalgaryCalgaryABCanada; ^146.2^University of AlbertaEdmontonABCanada

*Background*. A wide variety of drugs are known to cause acute pancreatitis. Apixaban is a relatively new anticoagulant approved by Health Canada and the Federal Drug Administration (FDA) in 2012 for the treatment and prevention of venous thromboembolic events and prevention of thromboembolism in non-valvular atrial fibrillation (AF). A variety of adverse reactions have been reported with apixaban, with bleeding being the most significant.

*Aims*. Here, we present a case of recurrent acute pancreatitis secondary to treatment with apixaban, highlighting the importance of considering newer drugs as possible etiologies of acute pancreatitis. To the best of our knowledge, this is the first reported case of apixaban-associated pancreatitis.

*Methods*. An 87-year-old female with a known history of atrial fibrillation, hypertension, previous cholecystectomy, and gout presented with a one-day history of severe epigastric abdominal pain, nausea, and vomiting. She had been started on apixaban three weeks prior for persistent non-valvular atrial fibrillation: the decision for anticoagulation was made by her family physician in consideration of her moderate-to-high risk for AF-related thromboembolism. Examination showed a tender epigastrium and periumbilicus but without peritonitis, and investigations demonstrated leukocytosis (12.8 × 10*E*9/L) and an elevated lipase (>3000 U/L). She was admitted for conservative management including analgesia and intravenous hydration. Her apixaban was temporarily suspended and her acute pancreatitis resolved rapidly. The patient was discharged home but she restarted apixaban after discharge, and 17 days after resuming apixaban, she developed recurrent symptoms of acute pancreatitis and required re-hospitalization.

*Results*. Extensive investigations were performed to exclude other diagnostic possibilities. An incidental pancreas divisum was seen on magnetic resonance cholangiopancreatography (MRCP), but no other drugs, metabolic, or autoimmune causes were identified. Endoscopic ultrasound was also performed and did not show any choledocholithiasis or biliary sludge. It was therefore strongly suspected that apixaban might be the offending agent. The patient was discharged after permanently discontinuing apixaban, and has not had any recurrence of symptoms since.

*Conclusions*. When evaluated using the World Health Organization Uppsala Monitoring Centre (WHO-UMC) Causality Assessment for drug-related adverse effects, this case represents at minimum a probable to likely case of drug-induced pancreatitis given the plausible time correlation, response to drug withdrawal and re-challenge, and exclusion of other diagnostic etiologies.

*Funding Agencies: *None

# Sub article: A147: Main-Duct Intraductal Papillary Mucinous Neoplasm of the Pancreas Associated with Spontaneous Pancreaticoduodenal and Pancreaticogastric Fistulas

**Authors:** R. Almeida, C. Dargavel, J. Mosko

**Affiliations:** ^147.1^University of TorontoTorontoONCanada

*Background*. Intraductal papillary mucinous neoplasms (IPMNs) of the pancreas are slow growing neoplasms arising from the epithelial lining of the pancreatic duct system. IPMNs represent a spectrum ranging from benign to invasive carcinoma. IPMNs complicated by the development of fistulas, however, are rare.

*Aims*. To describe a case of a main-duct (MD) IPMN associated with spontaneous pancreaticoduodenal and pancreaticogastric fistulas.

*Methods*. Case report and literature review.

*Results*. A 90-year-old woman with a prior history of a distal pancreaticojejunostomy for a pancreatic ductal carcinoma in situ 18 years ago, presented with cholangitis. An endoscopic retrograde cholangiopancreatography (ERCP) at the initial institution was unsuccessful due to altered anatomy. She was then transferred for percutaneous transhepatic cholangiography drain placement, which achieved biliary drainage. An esophagogastroduodenoscopy undertaken prior to a repeat ERCP showed a large gastric lesion with central ulceration along the greater curvature of the proximal body, with mucinous extrusion from the center and further drainage emanating from the second part of the duodenum obscuring visualization of the papilla (Figure 14). A multiphase CT of the pancreas showed an abnormal pancreaticobiliary system with a complex loculated cystic lesion in the pancreatic bed, approximately 13 × 6 × 16 cm in size, compressing the stomach and communicating to the greater curvature of the stomach and the superior wall of the third part of the duodenum (Figure 15). Histological examination of the gastric biopsies showed superficial villous architecture and gastric foveolar type epithelium with intestinal metaplasia and low grade dysplasia (Figure 16). This constellation of endoscopic, radiographic and histologic features was suggestive of malignant transformation of a MD-IPMN with spontaneous fistulization to the stomach and the duodenum.

The IPMN-associated fistulization to adjacent viscera has an incidence rate of 1.9%–6.6%. The mechanistic basis is hypothesized to include mechanical pressure, tumor penetration and pancreatic enzyme related autodigestion. While predominantly associated with malignancy, fistulization has also been reported in benign IPMNs. IPMN fistulas commonly involve the duodenum, followed by the stomach, CBD and colon. Whilst CT and MRI imaging characterize and diagnose IPMN fistulas, definitive diagnosis depends on histopathology. Accurate prognostic data on IPMN fistulas is unknown, however, scant literature suggests a 5-year survival rate of 43% after resection.

*Conclusions*. The rare complication of fistula formation in IPMN preferentially involves the duodenum, and usually occurs in the setting of malignant transformation. While uncommon, IPMN fistulization should be considered in the setting of cholangitis.

*Funding Agencies: *None

# Sub article: A148: Use of Rectal Indomethacin for Post-ERCP Pancreatitis Prevention: A Quality Assurance Study

**Authors:** S. Rolland, S. Shanmuganathan, A. Chatterjee, S. Grégoire, H. Dhaliwal, P. James

**Affiliations:** ^148.1^University of OttawaOttawaONCanada

*Background*. The evidence to date suggests that rectal indomethacin should be provided for post-ERCP pancreatitis (PEP) prevention for all high risk cases. This also benefits patients at average risk of PEP as well.

*Aims*. The aim of this quality assurance study is to determine the current use of rectal indomethacin for PEP prevention in our centre as well as its association with risk of PEP.

*Methods*. This is a retrospective chart review study for all ERCP cases performed at our institution from January to March 2015. Data regarding patient demographics and clinical status, procedure indication, interventions performed and use of indomethacin for PEP prevention was collected.

*Results*. Data from 41 ERCP cases where a sphincterotomy was performed was collected. The median patient age was 71 years and 54% were female. 24% of cases included the use of indomethacin for PEP prevention. Among cases that involved females under 50 years or patients with a history of pancreatitis, 11% received rectal indomethacin. Among the cases considered, two patients were seen in hospital for PEP (risk 5%) and no other complications were identified.

*Conclusions*. Rectal Indomethacin for PEP prevention is underused in our centre, especially among higher risk patients. However, the overall risk of hospitalization for PEP remained low. This is a retrospective chart study with a very small sample of patients. However, these results will be used to develop an algorithm aimed at identifying patients at elevated risk of PEP and facilitating increased use of rectal indomethacin for PEP prevention.

*Funding Agencies: *None

# Sub article: A149: A Rare Nidus for Biliary Stone Formation

**Authors:** R. Battat, M. Drapeau, B. Faulques, J. Wyse

**Affiliations:** ^149.1^McGill UniversityMontrealQCCanada; ^149.2^Université de MontrealMontrealQCCanada

*Background*. Early complications of Laproscopic Cholecystectomy (LC) include haemorrhage, perforation of the gallbladder, common bile duct (CBD) injury and iatrogenic bowel and vascular injuries^1^. Late complications involve intra-abdominal bile leakage, sub-hepatic abscesses, retained bile duct stones, post-cholecystectomy syndrome and bile duct stricture^2^ Surgical clips placed on the cystic duct and arteries avoid cystic duct leakage and arterial bleeding, but allows the rare late LC complication of post-cholecystectomy clip migration (PCCM) with gallstone formation. While rare, consequences of this complication, such as ascending cholangitis, can be life threatening.

*Aims*. We describe a 54-year-old Caucasian female patient with Crohn's disease presenting with abdominal pain attributable to post cholecystectomy clip migration with choledocholithiasis.

*Methods*. NA

*Results*. A 54-year-old woman presented with one episode of vomiting, a one month history of anorexia, and postprandial right sided and epigastric abdominal pain. Her past medical history includes Crohn's disease diagnosed in 1976, requiring total colectomy and end ileostomy in 1977 and a small bowel resection for structuring in 1980. A cholecystectomy for biliary pancreatitis was performed in 2004. Physical exam revealed a comfortable patient with normal vital signs and tenderness to deep palpation in the right upper quadrant. Laboratory investigations revealed a total bilirubin of 25.6 *μ*mol/L; aspartate aminotransferase 73 IU/L; alanine aminotransferase 174 IU/L; gamma-glutamyl transferase 310 IU/L; alkaline phosphatase: 243 IU/L; amylase: 91 IU/L; lipase: 87 IU/L; CRP: 87.5 mg/dL, and a white blood cell count of 8.3 × 10^9^/L. Computed tomography scan demonstrated a metallic object within the CBD with dense material organised around it. The CBD was dilated to 2.3 cm with intra-hepatic biliary duct dilation. The patient was diagnosed with subacute CBD obstruction from choledocholithiasis with gall stone formation around a surgical clip nidus. Endoscopic retrograde cholangiopancreatography (ERCP) with sphincterotomy was performed and the CBD stone was extracted and all symptoms and laboratory abnormalities resolved (Figure 17 [Fig fig18]).

*Conclusions*. Up to 80 cases of post-cholecystectomy and post LC clip migration with biliary stone formation have been reported in the literature. Most cases occur in female patients with a median age of 60 years old. The primary indications for cholecystectomy in these patients were acute or chronic cholecystitis or biliary pancreatitis. The median time between the cholecystectomy and the development of symptoms and clip migration with gallstone formation was 26 months post-cholecystectomy. Most were successfully treated by ERCP. No explanation or risk factors have been validated to clarify how the clips migrated in the common bile duct.

*Funding Agencies: *None

# Sub article: A150: Genetic Analysis of Pancreatic Cyst Fluid for GNAS and KRAS Mutations to Aid Pancreatic Cyst Classification

**Authors:** L. Ayres, J. Ho, B. Sheffield, C. Carter, A. Weiss, M. Byrne, D. Schaeffer, F. Donnellan

**Affiliations:** ^150.1^Pancreas Centre British Columbia and Division of GastroenterologyVancouver General HospitalVancouverBCCanada; ^150.2^Anatomical PathologyVancouver General HospitalVancouverBCCanada

*Background*. Endoscopic ultrasound (EUS) guided fine needle aspiration (FNA) is routinely used to evaluate pancreatic cysts. Cytology and biochemistry are limited in their ability to fully characterize cystic lesions. Recently, analysis of cyst fluid for the presence of genetic mutations has become available. KRAS and GNAS mutations have been shown to correlate with intraductal pancreatic mucinous neoplasia (IPMN).

*Aims*. To assess the correlation of KRAS and GNAS mutations with cytological diagnosis and surgical pathology where available.

*Methods*. Eleven consecutive patients attending a tertiary referral centre for assessment of pancreatic cysts had cyst fluid genetic analysis for KRAS and GNAS mutations where there was a sufficient quantity of fluid so as not to compromise routine cytology and biochemistry.

*Results*. Of 11 patients, 4 had wild type (WT) KRAS/GNAS with corresponding cytological diagnoses of pseudocyst, benign columnar cells and liposarcoma (one cytological specimen was non-diagnostic). Four patients had a mutation (3 KRAS mutations alone, 1 dual mutations) with corresponding cytological/surgical diagnoses of IMPN *n* = 3 and pancreatic adenocarcinoma. In three cases it was not possible to amplify the DNA.

Where both cytology and genetic analysis was available there was 100% concordance of WT and mutations of KRAS and/or GNAS with non-mucinous lesions and mucinous/malignant lesions respectively.

*Conclusions*. Genetic analysis of pancreatic cyst fluid is helpful in differentiating non-mucinous and mucinous lesions.

*Funding Agencies: *Pancreas Centre British Columbia

# Sub article: A151: Performance of EUS-Guided Fine Needle Biopsy of Solid Pancreatic Masses

**Authors:** L. Ayres, E. Lam, J. Telford

**Affiliations:** ^151.1^St. Paul's HospitalVancouverBCCanada; ^151.2^Univ. of CaliforniaVancouverBCCanada; ^151.3^University of British ColumbiaVancouverBCCanada

*Background*. Fine needle biopsy (FNB) has been developed to procure histological samples of solid lesions during endoscopic ultrasound (EUS).

*Aims*. To assess the accuracy of a new FNB needle in solid pancreatic masses.

*Methods*. A retrospective review of 45 consecutive adult patients with solid pancreatic masses who underwent EUS-guided FNB by two experienced endosonographers at St. Paul's Hospital using the 25 gauge Sharkcore needle was performed. Diagnostic accuracy of FNB was compared to gold standard surgical pathology (where available) or six month follow up. If histology was suspicious for malignancy but further sampling was required to prove malignancy, this was categorized as a false negative, however if further sampling was not judged necessary by the interdisciplinary team this was categorized as a true positive.

*Results*. 50 procedures were carried out on 45 patients. Diagnosis was adenocarcinoma *n* = 31 (69%), lymphoma *n* = 4 (8.8%), post operative inflammatory *n* = 2 (4.4%) reactive/inflammatory change *n* = 2 (4.4%) and chronic pancreatitis, neuroendocrine tumour, sarcoma, metastatic melanoma, glandular atypia, normal pancreas all *n* = 1 (2.2%). The site of the tumor was pancreatic head in *n* = 30 (67%), uncinate process *n* = 3 (6.7%), genu *n* = 3 (6.7%), body *n* = 5 (11%) and tail *n* = 2 (4.4%). There were no technical failures of the needle. The median number of passes was 2 (range 2–4). Sufficient tissue was obtained in 100%. Performance characteristics per procedure were: diagnostic accuracy 90%, sensitivity 88.4% and specificity 100%. Significant complications occurred in 4%: bleeding *n* = 1 and pancreatitis *n* = 1.

*Conclusions*. Our initial experience using the Sharkcore FNB for solid pancreatic masses demonstrates a high diagnostic yield and an acceptable complication rate.

*Funding Agencies: *None

# Sub article: A152: An Evaluation of the Role of Transient Elastography in Assessing Pediatric Cystic Fibrosis Associated Liver Disease in Children with Cystic Fibrosis

**Authors:** S. Lam, H. Machida, R. Myers, C. Ortiz-Neira, S. Martin, J. Yap, J. deBruyn

**Affiliations:** ^152.1^University of CalgaryCalgaryABCanada; ^152.2^Alberta Children's HospitalCalgaryABCanada; ^152.3^University of AlbertaEdmontonABCanada

*Background*. Cystic fibrosis associated liver disease (CFLD) and its complications are increasingly recognized as the highest non-pulmonary cause of death in children with CF. The gold standard of liver biopsy for diagnosis of CFLD has limitations, including invasiveness, association with morbidity, and poor practicality for screening in children. Early ultrasonographic (US) changes may be subtle and subject to inter-observer variability.

*Aims*. The primary objective was to evaluate the diagnostic properties of Transient Elastography (TE) using FibroScan in children with CF for detection of CFLD, as defined by EuroCare Criteria. The secondary objective was to identify factors associated with the presence of CFLD.

*Methods*. Children from the Southern Alberta cystic fibrosis clinic at the Alberta Children's Hospital underwent liver stiffness measurements (LSM) by TE. Sensitivity, specificity, and receiver operator characteristic (ROC) curve of TE were calculated and compared to EuroCare criteria for diagnosis of CFLD (≥2 of the following: persistent abnormal liver biochemistry over 12 months, hepatosplenomegaly, or US abnormalities). Age, anthropometrics, hepatosplenomegaly, genotype, lung and pancreatic function, history of small bowel bacteria overgrowth and meconium ileus, severity of liver disease on US with validated scoring systems, and past medications were examined to determine any correlation with the presence of CFLD.

*Results*. Forty-one of 130 patients in the CF clinic completed the study. The median age was 8.5 years, (interquartile range (IQR) 5–12 years) with 56% females. The prevalence of CFLD was 9.7% (*n* = 4). The TE failure rate was 7.3%. (*n* = 3); An 18 month and 20 month old child were uncooperative, a 6 year old with autism spectrum disorder did not complete testing due to anxiety). Children with CFLD had significantly higher median LSM 13.6 kPa (IQR 5.7–27.8 kPa) compared to those without CFLD 4.6 kPa (IQR 3.2–5.1 kPa) (*p* = 0.0042). When a cut-off value of ≥5.3 kPa was used, the sensitivity, specificity, positive and negative predictive values were 100% (95% CI 39–100%), 87% (95% CI 71–95%), 44% (95% CI 26–64%), 100%. A ROC curve for detecting CFLD with this cut off was 0.93 (95% CI 0.87–0.98). No examined factors showed association with CFLD.

*Conclusions*. TE is well tolerated and successful in the majority of children with CF. TE has a role as a useful non-invasive test to screen and diagnose CFLD in children with CF.

*Funding Agencies: *Alberta Children's Hospital Research Institution Small Research Grant

# Sub article: A153: Role of Transient Elastrography in Assessment of Cystic Fibrosis-Associated Liver Disease

**Authors:** J. Woolfson, S. Raveendran, M. Chilvers, R. Schreiber, O. Guttman

**Affiliations:** ^153.1^BC Children's HospitalVancouverBCCanada

*Background*. Cystic Fibrosis-associated liver disease (CFLD) occurs in 30% of patients and is the 3rd most common cause of mortality in CF patients. Diagnosis is challenging as specific tests for detection of fibrosis in pediatric CFLD have not been developed and existing investigations do not correlate well with presence or severity of disease. Liver biopsy is rarely indicated because of the patchy nature of the disease. Transient Elastrography (TE) is a rapid non-invasive method for assessing liver fibrosis. Studies suggest it may be a valuable tool in pediatric patients, though its role in detecting CFLD has only begun to be explored. AST : platelet ratio index (APRI) has been validated as a surrogate marker of hepatic fibrosis in chronic liver diseases.

*Aims*. The purpose of this study was to assess the utility of TE and to determine the role of APRI and standard biochemistry in identifying liver fibrosis in CF patients.

*Methods*. Patients 2–18 years old were recruited from the British Columbia Children's Hospital CF clinic. Charts were reviewed for demographic and clinical data including bloodwork and abdominal imaging. Each patient underwent TE by a single trained operator. Patients were determined to have CFLD using standard criteria based on hepatic biochemistry, imaging and clinical examination. Where the original basis for CFLD diagnosis was unclear from chart review, patients maintained on ursodiol were included in the CFLD group.

*Results*. 55 patients were included in the study (50.9% male, mean age 11.6 (range 5.1–17.5) years). 49% were homozygous for ΔF508 gene, 36.3% were heterozygous, 7.3% had other mutations and 7.3% were genotype unknown. 22 patients had a diagnosis of CFLD (40%) and 20 of these were on ursodiol (90.9%). Two patients had ultrasound findings of cirrhosis and one had portal hypertension. Of the 22 CFLD patients, 45.5% were male (*P* = 0.586), 59% were homozygous for ΔF508 (*P* = 0.685) and 90.9% were pancreatic insufficient (*P* < 0.0001). All mean liver enzymes were higher in the CFLD group, significantly ALT (*P* = 0.031) and ALP (*P* = 0.015). Mean TE values were significantly higher in the CFLD group (5.92, range 3.9–16.5) versus no liver disease (4.54, range 2.1–7.2; *P* = 0.0147). APRI was higher in the CFLD group (0.396 versus 0.324, *P* = 0.1191). Linear regression showed a positive association between TE value and APRI (Slope 0.058; CI 0.038–0.79;* R* ^2^ = 0.386).

*Conclusions*. CFLD is one of the leading causes of morbidity in CF, but limitations of existing tests hamper diagnosis and monitoring. In this study, TE values were significantly higher in CFLD patients and correlate with APRI values, suggesting that TE may have clinical applications for identifying and following patients with this condition. Further research is needed at a larger scale to determine TE cutoff values for diagnosing CFLD.

*Funding Agencies: *None

# Sub article: A154: Liver Transplant in an Infant Presenting with Hepatic Failure Secondary to Severe Pyruvate Kinase Deficiency

**Authors:** M. Chartier, M. Paganelli, N. Ahmed, F. Alvarez

**Affiliations:** ^154.1^CHU Ste*-*JustineMontrealQCCanada; ^154.2^CHU*-*Sainte JustineMontrealQCCanada; ^154.3^McGill University Health CentreMontrealQCCanada

*Background*. Pyruvate kinase deficiency (PKD) is the most common cause of congenital non-spherocytic chronic hemolytic anemia and results from an erythrocyte enzyme defects. Patients with pyruvate kinase deficiency can have a broad spectrum of clinical manifestations, ranging from mild asymptomatic anemia to severe and transfusion dependent anemia. Most patients normally present with some degree of hemolysis, hyperbilirubinemia, anemia and splenomegaly. Only few reports have documented associated severe progressing liver failure.

*Aims*. To describe the case of an infant with severe pyruvate kinase deficiency leading to liver failure and requiring liver transplantation.

*Methods*. We retrospectively reviewed the medical chart of our patient with pyruvate kinase deficiency and liver failure. All articles about such a rare complication of pyruvate kinase deficiency published in the English literature from 1962 o October 2015 were reviewed.

*Results*. Our patient presented with severe hemolytic anemia and cholestasis at birth, requiring double exchange transfusion and repeated transfusions thereafter. He subsequently developed progressive cirrhosis, portal hypertension, ascites and liver failure requiring prolonged hospitalization and biweekly paracentesis. Two liver biopsies done more than one month apart showed progressive liver fibrosis. Despite extensive investigations, the only identified etiology for cholestasis and liver failure was compound heterozygous mutations for PKD and single heterozygous mutation for ABCB4, the latter being a likely benign variant. The patient was transplanted at 6 months of age and underwent a splenectomy during the same intervention. To the best of our knowledge, only three cases of severe hepatic failure secondary to PKD have been reported but this is the first to have successfully undergone liver transplant.

*Conclusions*. The hepatic failure in patients with severe pyruvate kinase deficiency is most likely multifactorial, involving prenatal hemolysis with subsequent bile ducts obstruction, minimal inflammation secondary to iron overload and extramedullary hematopoiesis, but the most likely explanation is that genetic mutations of PKLR in our patient affect both the expression of PK-R (in erythrocytes) and PK-L (in hepatocytes) with an inappropriate compensation of PKM2, leading to severe and fatal enzymatic defect.

*Funding Agencies: *None

# Sub article: A155: Acute Deterioration in Metabolic Control in a Child Post Liver Transplant for Maple Syrup Urine Disease

**Authors:** P. Kawada, S. Jain, A. Chan, J. Yap

**Affiliations:** ^155.1^University of AlbertaEdmontonABCanada

*Background*. In Maple Syrup Urine Disease (MSUD) the inherited deficiency is in the gene that codes for the branched-chain *α*-keto acid dehydrogenase (BCKDH) complex. The classical form of MSUD is the severest. BCKDH degrades branched-chain amino acids (BCAA), therefore deficiency leads to accumulation of leucine, isoleucine and valine along with allo-isoleucine which is diagnostic. Treatment is dietary protein restriction although acute decompensation, with high leucine, ketosis and metabolic acidosis with neurological symptoms, can still occur. Liver transplantation supplements BCKDH activity and stabilizes metabolic control. Acute deterioration following liver transplantation has only previously been described once.

*Aims*. As more patients are being transplanted for MSUD, it is important to recognize that with significant intercurrent illness, there is potential for metabolic decompensation; particularly in those with previously severe MSUD.

*Methods*. Case report.

*Results*. A 4 year old boy 3 years post liver transplant for severe, classical MSUD presented acutely with ataxia and profoundly elevated BCAA levels. Prior to admission, the post transplant course had been stable with normal allograft function and no acute metabolic decompensation. Post-transplant neurodevelopment was normal, apart from mild fine motor delay. The child had fever, diarrhea and vomiting 2 days prior to admission. At presentation to the emergency room, the child was lethargic, drowsy, dysarthric and ataxic. An impressive rise in the BCAA was seen (Table 23). He was rehydrated with two normal saline boluses and maintained on IV fluids which included 5% dextrose with improvement in symptoms. No additional metabolic treatment was required and he was discharged home the next day. There was no residual neurological sequelae.

*Discussion*. Post liver transplant, classical MSUD patients achieve satisfactory metabolic control allowing liberalization of dietary protein restriction. There has only been a single report of acute metabolic decompensation post transplant in a child who developed transient leucinosis in the context of gastroenteritis with severe dehydration. It is postulated that catabolic illness with dehydration transiently affects clearance of BCAA by the liver graft.

*Conclusions*. Classical MSUD liver recipients who are significantly unwell, especially if dehydrated, should have early BCAA monitoring and a sick day protocol that include high dextrose solutions.

*Funding Agencies: *None

# Sub article: A156: Adherence to Response-Guided Peg-Interferon and Ribavirin for People Who Inject Drugs with HCV Genotype 2/3 Infection: The Activate Study

**Authors:** E. Cunningham, B. Hajarizadeh, O. Dalgard, B. Conway, G. Foster, P. Bruggmann, M. Backmund, G. Robaeys, T. Swan, J. Amin, P. Marks, S. Quiene, M. Weltman, D. Shaw, A. Dunlop, M. Hellard, J. Bruneau, C. Staehelin, G. Dore, J. Grebely

**Affiliations:** ^156.1^The Kirby InstituteUNSWRandwickNSWAustralia; ^156.2^Akershus University HospitalOsloNorway; ^156.3^Vancouver Infectious Diseases CenterVancouverBCCanada; ^156.4^The Liver UnitQueen Marys University of LondonLondonUK; ^156.5^Arud Centres for Addiction MedicineZurichSwitzerland; ^156.6^Ludwig Maximilians*-*University MunichMunichGermany; ^156.7^Department of Gastroenterology and HepatologyZiekenhuis Oost LimburgGenkBelgium; ^156.8^Treatment Action GroupNew YorkNYUSA; ^156.9^Nepean HospitalSydneyNSWAustralia; ^156.10^Royal Adelaide HospitalAdelaideNSWAustralia; ^156.11^School of Medicine and Public HealthUniversity of NewcastleNewcastleNSWAustralia; ^156.12^Burnet InstituteMelbourneVICAustralia; ^156.13^Universite de MontrealMontrealQCCanada; ^156.14^Division of Infectious DiseasesUniversity Hospital and University of BernBernSwitzerland

*Background*. Adherence to HCV therapy impacts SVR, but data is limited among people who inject drugs.

*Aims*. This study assessed PEG-IFN adherence and associated factors.

*Methods*. Participants with HCV G2/3 who had recently injected drugs (last 12 weeks) or receiving opioid substitution therapy were recruited (2012–14) and received directly observed PEG-IFN and self-administered RBV. Participants with an RVR received 12 weeks (shortened duration) and those without RVR received 24 weeks (standard duration) therapy. The primary endpoint was SVR12 and 80/80 PEG-IFN adherence.

*Results*. Overall, 93 initiated HCV treatment (mean age 42; 82% men; 87% G3; 55% injected drugs in the last month; 70% on OST at baseline). Sixty-five percent (*n* = 60) received shortened treatment, while 29% (*n* = 27) received standard treatment. Six participants discontinued prior to week 4. 80/80 PEG-IFN adherence was 81% (*n* = 75), 6% missed ≥1 dose (on-treatment adherence >99%) and 27% (*n* = 22) discontinued early (virological failure (*n* = 1), lost to follow up/unwillingness (*n* = 10) and side effects (*n* = 11)). Treatment completion was higher in those receiving 12 versus 24 weeks of therapy (97% versus 52%, *P* < 0.01, Figure 19 [Fig fig20] [Fig fig21]). Injecting drugs in the last month did not impact 80/80 adherence (81% versus 80%, *P* = 0.95). Treatment duration of 12 weeks (those with RVR) was the only factor associated with ≥80/80 adherence (versus 24 weeks; AOR 40.6, 4.9–338.0). SVR was 63% (82%—12 weeks; 37%—24 weeks), and was associated with ≥80/80 adherence (79% versus 0%, *P* < 0.01).

*Conclusions*. High adherence to therapy was observed, irrespective of recent injecting drug use. Sub-optimal exposure was driven by early treatment discontinuation, not missed doses during therapy. Treatment completion and adherence were higher in people receiving 12 weeks of therapy compared to 24 weeks.

*Funding Agencies: *Merck & Co.

# Sub article: A157: Ledipasvir/Sofosbuvir (LDV/SOF) for 8 Weeks in Genotype 1 (GT1) Treatment-Naïve (Tn) Non-Cirrhotic (NC) Patients with HCV Viral Load (VL) <6 Million IU/mL (6 m); A Comparative Analysis of the Phase-3 Ion-3 Efficacy Data to Real World Effectiveness (RWE)

**Authors:** N. Tsai, M. Curry, P. Buggisch, S. Milligan, D. Mumm, M. Natha, E. Eggleton, B. Kreter, D. Brainard, K. Kowdley

**Affiliations:** ^157.1^Queens Medical CenterHonoluluHIUSA; ^157.2^Beth IsraelBostonMAUSA; ^157.3^ifi*-*Institute for Interdisciplinary MedicineHamburgGermany; ^157.4^TRIO Health AnalyticsLa JollaCAUSA; ^157.5^Gilead Sciences CanadaMississaugaONCanada; ^157.6^Gilead Sciences Inc.Foster CityCAUSA; ^157.7^Swedish MedicalSeattleWAUSA

*Aims*. The optimal duration of therapy to achieve SVR depends on multiple factors. Patients treated with LDV/SOF with 8, 12 or 24 weeks achieved SVR12 from 94–100% in the ION Phase 3 studies. Duration of therapy is based on treatment history, cirrhosis status and baseline VL. In a post-hoc analysis of the ION-3 (TN, NC patients) 8 week data, a VL < 6 M was shown to be the best predictor of SVR.

*Methods*. Diverse RWE LDV/SOF data is emerging from single-center (Buggisch) and multicenter (GECCO) retrospective chart reviews to large multicenter prospective cohorts (TARGET, TRIO). In this analysis, the Phase-3 ION-3 data is compared with several real-world cohorts. Patient demographics, characteristics and SVR12 data has been collated and compared.

*Results*. The ION-3 post-hoc analysis reported 123 patients who were TN, NC and VL < 6 M and treated with 8 weeks of LDV/SOF. Mean age was 52, 22% black, 72% GT1a; the SVR12 was 97%. In TARGET; 87% of GT1, TN, NC patients had a baseline VL < 6 M; 35% of these received an 8-week regimen. Preliminary SVR4 in 59 patients is 97%. In the TRIO cohort, 8-week therapy was initiated for 37% of patients with baseline VL < 6 M, Mean age was 57, 70% GT1a, 20% black and SVR12 data in 181 patients is 97%. Buggisch et al. shows 100% SVR12 (*n* = 44). Mean age is 50, 52% GT1a and 88.1% had comorbid conditions. The GECCO cohort also includes patients with baseline HCV VL > 6 M, advanced fibrosis and HIV/HCV co-infection; SVR4 is 100% (*n* = 44). Low rates of Adverse Events (AEs), relapse rates and discontinuations were seen in all 4 cohorts. Complete SVR12 data is expected in these cohorts by the time of presentation.

*Conclusions*. LDV/SOF for 8 weeks in the appropriate patient population yielded high SVR rates in ION-3. Analysis of real world effectiveness data from several diverse & heterogeneous cohorts from the US & EU show SVR outcomes that were consistent with the Phase-3 ION-3 results and supports the use of 8 weeks LDV/SOF in treatment-naive, non-cirrhotic GT1 patients with a baseline HCV VL <6 million IU/mL.

*Funding Agencies: *Gilead Sciences Inc.

# Sub article: A158: Long Term Efficacy and Safety of Tenofovir DF (TDF) in Chronic Hepatitis B Patients (CHB) with Documented Lamivudine Resistance (LAM-R): 5 Year Results from a Randomized, Controlled Trial

**Authors:** S. Fung, H. Hann, M. Elkashab, M. Khan, T. Berg, M. Fabri, A. Horban, I. Sporea, J. Flaherty

**Affiliations:** ^158.1^Toronto General HospitalTorontoONCanada; ^158.2^2Thomas Jefferson UniversityPhiladelphiaPAUSA; ^158.3^Toronto Liver CentreTorontoONCanada; ^158.4^Gilead Sciences CanadaIncMississaugaONCanada; ^158.5^University Hospital LeipzigLeipzigGermany; ^158.6^Clinic for Infectious DiseasesNovi SadSerbia; ^158.7^Medical University of WarsawWarsawPoland; ^158.8^Spitalul Clinic Judetean de UrgentaTimisoaraRomania; ^158.9^Gilead SciencesIncFoster CityCAUSA

*Background*. In CHB patients with LAM-resistance (LAM-R), TDF has shown efficacy comparable to FTC/TDF and no detectable TDF resistance at 2 years (Gastroenterology 2014; 146:980-88).

*Aims*. The final 5 year efficacy and safety results from this trial are presented.

*Methods*. CHB patients on LAM with HBV DNA >3 log_10 _IU/mL and with documented LAM-R (INNO-LiPA Multi-DR, v3) were randomized (1 : 1) to TDF or FTC/TDF and followed in a blinded fashion for 5 years.

*Results*. Two hundred eighty patients were randomized; 239 (85%) completed 5 years of treatment. At baseline, mean age was 47 years, most were male (75%) and non-Asian (66%); 53% were HBeAg-negative, and HBV genotype distribution (A®D) was 22%, 13%, 19%, and 43%, respectively. Mean (SD) HBV DNA was 5.7 (1.9) log_10 _IU/mL, and 42% had ALT ≤ ULN at baseline. At Year 5, virologic, serologic, and biochemical responses were similar among groups, and remained stable from Year 2 to 5 (Table 24). Nine patients (4-TDF, 5-FTC/TDF) discontinued due to an adverse event, including increased serum creatinine in 1 patient. Hepatocellular carcinoma was reported in 4 (1.4%) patients. Confirmed renal safety endpoints (both groups combined) over 5 years were: CrCL <50 mL/min in 19 (6.8%) patients (12 requiring dose modification), increases in serum creatinine of 0.3 and 0.5 mg/dL from baseline in 21 (7.5%) and 2 (0.7%) patients, respectively, and serum phosphorus <2 mg/dL in 3 (1.1%) patients. For both groups combined, mean declines in BMD (g/cm^2^) from baseline for hip and spine BMD, respectively, were 1.7% and 1.5% at Year 2, and 2.5%, and 1% at Year 5. Seven patients experienced fracture (all except 1 were trauma-related). No TDF resistance was detected through 5 years of treatment by population sequencing.

*Conclusions*. In LAM-R patients with CHB treated for 5 years with TDF, a high rate of HBV DNA suppression was achieved and maintained with no detectable TDF resistance. There is no apparent advantage of combination FTC/TDF in this population. Renal events associated with TDF occurred in up to 7.5% of patients, and average losses in bone mineral density of 1–2.5% were observed.

*Funding Agencies: *Gilead Sciences, Inc.

# Sub article: A159: On-Treatment HCV RNA Decline in Pre- and Post-Liver Transplant Patients with Different Degrees of Fibrosis and Cirrhosis: A Combined Analysis of the Solar Trials

**Authors:** T. Welzel, R. Reddy, M. Manns, S. Flamm, D. Mutimer, E. Gane, R. Hyland, J. McHutchison, D. Mumm, D. Samuel, M. Charlton, X. Forns, G. Everson, S. Zeuzem, N. Afdhal

**Affiliations:** ^159.1^Johann Wolfgang Goethe University Medical CenterFrankfurtGermany; ^159.2^University of PennsylvaniaPhilidelphiaPAUSA; ^159.3^Hannover Medical SchoolHannoverGermany; ^159.4^Northwestern University Feinberg School of MedicineChicagoILUSA; ^159.5^Queen Elizabeth HospitalBirminghamUK; ^159.6^University of AucklandAucklandNew Zealand; ^159.7^Gilead Sciences Inc.Foster CityCAUSA; ^159.8^GileadMississaugaONCanada; ^159.9^Universite Paris*-*SudVillejuifFrance; ^159.10^Intermountain Medical CenterMurrayUTUSA; ^159.11^Liver UnitIDIBAPS and CIBEREHDBarcelonaSpain; ^159.12^University of ColoradoDenverCOUSA; ^159.13^Beth Israel Deconess Medical CnterBostonMAUSA

*Aims*. In the SOLAR-1 and SOLAR-2 studies, ledipasvir/sofosbuvir (LDV/SOF) + ribavirin (RBV) for 12 or 24 weeks resulted in high SVR rates in genotype (GT) 1 or 4 HCV-infected patients with decompensated cirrhosis or who were liver transplant recipients. In this large combined post hoc analysis, we investigate whether on-treatment HCV RNA response varied by patient population and/or was predictive of treatment outcome.

*Methods*. Data from the identically designed SOLAR-1 & 2 studies were combined. Six groups of GT 1 or 4 patients were randomized to receive 12 or 24 wks of LDV/SOF + RBV treatment: patients without transplant and either CPT B cirrhosis, or CPT C cirrhosis; or patients who have undergone transplantation (post-OLT) and who were either without cirrhosis (F0 to F3), CPT A cirrhosis, CPT B cirrhosis, or CPT C cirrhosis. For analysis of early viral kinetics, the 12 and 24 wk treatment durations were combined. Serum HCV RNA was quantified using Roche CAP/CTM v2.0 with a lower limit of quantitation (LLOQ) of 15 IU/mL.

*Results*. Patients with advanced liver disease had SVR12 rates of 86–89%. Patients who were post-OLT had SVR rates that ranged from 96–98% in those with F0-F3 fibrosis to 60–75% of patients with severe hepatic impairment. Rapid HCV RNA declines were observed in all treatment groups. The majority of subjects achieved HCV RNA <LLOQ at WK 4. The percentages of patients who were <LLOQ or <LLOQ Target Not Detected (TND) at Wks 1, 2, 4, and 6 will be presented. Among baseline variables, high viral load and the presence of liver cirrhosis were associated with slower on treatment HCV RNA decline. An analysis of baseline variables, on-treatment HCV RNA and SVR12 will be presented.

*Conclusions*. Early viral decline during treatment is rapid in patients with compensated and decompensated HCV before and after liver transplantation. The proportion who achieved HCV RNA <LLOQ at Wk2 was highest in the patients without cirrhosis, similar among patients with CTP A and B cirrhosis, and lowest in those with CPT C cirrhosis.

*Funding Agencies: *Gilead Sciences Inc.

# Sub article: A160: Examination of Genetic Variation within the Hepatitis C Virus NS3 Helicase Using Deep Sequencing

**Authors:** C. Ablenas, N. Bensoussan, M. Powdrill, L. Mayrthaler, J. Pezacki, M. Gotte

**Affiliations:** ^160.1^McGillMontrealQCCanada; ^160.2^University of OttawaOttawaONCanada; ^160.3^Ludwig*-*Maximilians*-*UniversitätMunichGermany; ^160.4^University of AlbertaEdmontonABCanada

*Background*. Hepatitis C virus (HCV) subgenomic replicons are extremely useful for the study of viral replication. Replication enhancing mutations (REMs), also referred to as cell culture adaptive mutations, have been identified that result in efficient RNA amplification in the highly permissive Huh-7 hepatoma cell line. Within the HCV replicon REMs cluster to NS5a, as well as to the amino terminus of the NS3 helicase (NS3h), and at two positions in NS4b. In the context of NS3h, the majority of REMs map to domain 1, with few REMs in domains 2 and 3. Domains 1 and 2 are highly structurally related, containing the ATP binding site and the majority of the contacts with the RNA substrate, while domain 3 is primarily structural.

*Aims*. To use a deep sequencing approach to investigate genetic variation in NS3h using the replicon system. We compared genetic variation in domain 1, which contains the majority of NS3h REMs reported in the literature, to that in domain 3 where few REMs have been reported.

*Methods*. Deep sequencing of amplicons covering the different domains of NS3h was performed to detect minor variants arising during replication. Briefly, RNA was extracted from Huh7 cells harbouring the 1b replicon and reverse transcribed into cDNA. PCR amplicons were then amplified using barcoded PCR primers with 454 adaptor sequences and subjected to pyrosequencing on a GS Junior sequencer (Roche). The raw reads were processed, and minor variants calculated using Roche amplicon variant software. Select mutants were introduced back into the replicon by site-directed mutagenesis, and the replication capacity was measured using a luciferase reporter.

*Results*. The error rate calculated from variants sequenced in a region of domain 1 in NS3h was approximately 2-fold higher than that for a region in domain 3. This suggests a higher tolerance for variation in domain 1 as compared to domain 3, consistent with the greater number of REMs previously reported in domain 1. In agreement with previous reports from our group, the majority of the variants consisted of transitions due to the high bias by the NS5b polymerase to form G/U or U/G mismatches, with only a small number of transversions. At the level of the individual variants, we noted five mutations in domain 1 and one mutation in domain 3 that were detected repeatedly over 2 to 3 independent RNA transfections. When the replication capacities of select mutants were tested, four mutants were found to replicate ~4–9 fold higher than WT.

*Conclusions*. We found that genetic variation was better tolerated in domain 1 as compared to the more structural domain 3. In addition, certain nucleotide positions were identified as hot spots for specific mutations.

*Funding Agencies: *CIHR, CanHepC, and FRQS

# Sub article: A161: RNA Pull-Down Strategies to Investigate the Roles of Microrna-122-Associated Complexes in Hepatitis C Virus Infection

**Authors:** A. Bernier, S. Sagan

**Affiliations:** ^161.1^McGill UniversityMontrealQCCanada

*Background*. Approximately 200 million individuals worldwide are infected by hepatitis C virus (HCV), including more than 268 000 Canadians. MicroRNA-122 (miR-122) is a highly abundant liver-specific microRNA shown to interact at two “tandem" microRNA-binding sites in the 5′ end of the HCV genome. This unusual interaction promotes HCV RNA accumulation in both HCV-infected cells and the livers of infected patients. Mutation, truncation, or exchange of the 3′ terminal ribonucleotides of miR-122 for deoxynucleotides reduces HCV RNA accumulation. However, these nucleotides are not required for canonical miRNA activities. This suggests that sequences in the 3′ tail of miR-122 may mediate important interactions with viral or cellular factors involved in HCV RNA accumulation.

*Aims*. We hypothesize that miR-122 forms a distinct complex with host and/or viral proteins that together mediate HCV RNA accumulation. Hence, we aim at identifying and characterizing host and viral factors associated with non-canonical miR-122 complexes in HCV-infected cells to understand miRNA-mediated viral RNA accumulation and identify novel antiviral targets.

*Methods*. Alkyne-tagged miR-122 molecules are transfected into HCV RNA-harboring Huh-7 cells. Following miR-122 biotinylation by a click reaction, miR-122 ribonucleoprotein complexes from naïve and HCV-infected cells are isolated by streptavidin affinity purification. MiR-122-associated proteins are then analyzed by SDS-PAGE and liquid chromatography tandem mass spectrometry. Comparison of miR-122 complexes from naïve and miR-122 site 1 or 2 mutant HCV-infected cells will allow the identification of proteins acting specifically at each site of the HCV genome. Proteins interacting with the 5′ end of HCV in the absence of miR-122 will also be identified following pull-down of a mutant HCV RNA containing a BoxB stem-loop using biotinylated-*λ*N peptide.

*Results*. We demonstrate that alkyne-tagged miR-122 molecules are functional in mediating HCV RNA accumulation in Huh-7 cells. We show that the click reaction is stable under physiological conditions and permits efficient labeling and affinity purification of miR-122 molecules in cell lysates. Western blot of affinity purified miR-122 complexes show enrichment in the RNA-induced silencing complex (RISC) protein Argonaute 2. Additionally, we show that a mutant HCV RNA with a BoxB loop in the place of stem-loop I is replication competent.

*Conclusions*. We expect that the results will provide insight into a novel microRNA* “capping complex” *as well as a non-canonical* “microRNA enhancing complex”*. We anticipate that we will identify novel host-virus interactions important for viral replication that will provide new targets for therapeutic intervention.

*Funding Agencies: *CIHR, CanHepC; FRSQ

# Sub article: A162: Two Serologically Distict Forms of Occult Hepadnaviral Infection Accompanied by Hepatocellular Carcinoma Development Identified in the Woodchuck Model of Hepatitis B

**Authors:** T. Michalak, P. Mulrooney-Cousins, N. Churchill

**Affiliations:** ^162.1^Memorial UniversitySt. John'sNFCanada

*Background*. Woodchuck hepatitis virus (HBV) is molecularly and pathogenically closely related to hepatitis B virus (HBV). Both viruses cause similar liver pathology, where acute hepatitis (AH) can progress to chronic hepatitis (CH) and hepatocellular carcinoma (HCC). They also establish persistent, asymptomatic (occult) infections.

*Aims*. To determine molecular and immunological characteristics, and pathological consequences of experimentally induced occult hepadnaviral persistence in WHV-infected woodchucks. To recognize if different amounts of infectious WHV establish distinct forms of asymptomatic WHV carriage.

*Methods*. Woodchucks were followed for life after i.v. injection with 10 to 10^10^ DNase-digestion protected virions and examined for WHV DNA in serial serum, PBMC and liver biopsies, and for virus replication intermediates in PBMC and liver by PCR/nucleic acid hybridization-based assays. Serum WHV surface antigen (WHsAg), antibodies to WHV core (anti-WHc) and WHsAg (anti-WHs), liver histology, WHV-specific T cell responses, and infectivity of persisting WHV were assessed.

*Results*. Animals injected with >10^3^ virions developed serum WHsAg/anti-WHc-positive AH. In the majority (~90%) of animals, AH resolved, serum WHsAg seemingly completely cleared within 6–18 weeks post-infection (p.i.), anti-WHs developed, while anti-WHc and low levels of WHV DNA persisted for life in serum (≤100–200 copies/mL), PBMC and liver (~0.1–10 copies/*μ*g DNA) with evidence of WHV replication in the liver and the immune system. Intermittent minimal to moderate liver inflammation protracted during lifespan and HCC developed in ~20% of the animals. This form was termed secondary occult infection or SOI. The remaining ~10% of animals with AH progressed to CH and ~80% of them developed HCC. Woodchuck injected with 10 or 100 virions established serum WHsAg/anti-WHc-negative infection in which WHV replication was initially restricted to the lymphatic system. This primary occult infection (POI) with time (~3 year p.i.) spread to the liver and HCC developed in ~20% of animals in 5 years p.i. The virus persisting during both SOI and POI caused serum WHsAg-positive hepatitis advancing to HCC in some cases when concentrated and administered to virus-naïve woodchucks. SOI coincided with WHV-specific B and T cell immune responses and protection against reinfection, but POI was accompanied by virus-specific T cell but not B cell response and by a lack of immune protection against challenge with liver pathogenic doses of WHV (>10^3^ virions). WHV DNA integrated into the liver and the immune system in both SOI and POI.

*Conclusions*. The amount of WHV determines whether anti-WHc/anti-WHs-positive SOI or totally seronegative POI is established. These two forms of occult WHV infection.

*Funding Agencies: *CIHR

# Sub article: A163: Distribution of Hepatitis C Risk Factors and HCV Treatment Outcomes among Central Canadian Aboriginals

**Authors:** P. Parmar, D. Corsi, C. Cooper

**Affiliations:** ^163.1^Faculty of MedicineUniversity of OttawaOttawaONCanada; ^163.2^Ottawa Hospital Research InstituteOttawaONCanada; ^163.3^Division of Infectious DiseasesDepartment of MedicineUnivesity of OttawaOttawaONCanada

*Background*. Aboriginal Canadians face higher levels of lifestyle risk factors for hepatitis C exposure including drug use and incarceration.

*Aims*. We examined multiple risk factors clustering among Aboriginals.

*Methods*. The Ottawa Hospital Viral Hepatitis Clinic Cohort (January 2000–August 2013) was evaluated. Demographic data, HCV infection risk factors, and HCV treatment outcomes were assessed. Markers of socioeconomic status were based on area-level indicators linked to postal code.

*Results*. 55 (2.8%) Aboriginal and 1923 (97.2%) non-Aboriginals were evaluated. Aboriginals were younger (45.6 versus 49.6 years, *p* < 0.01). The distribution of gender (63.6% versus 68.3% male), HIV co-infection (9.1% versus 8.1%), genotype 1 infection (68.5% versus 65.4%), advanced fibrosis stage (29.2% versus 28.0% F2+), and SVR rate (56.3% versus 58.9%) was similar between Aboriginal and non-Aboriginals (*p* > 0.10). Aboriginal status was associated with a higher number of HCV risk factors, (mean 4.2 risk factors versus 3.1, *p* < 0.001) with an odds ratio of 2.5 (CI 1.4–4.4) for having at least 4 risk factors. This was not explained after adjustment for markers of socioeconomic status including income, social deprivation, and poor housing. Multivariable logistic regression suggested that SVR was unrelated to Aboriginal status (*p* = 0.83). Aboriginal patients interrupted therapy more often due to lost-to-follow-up, lesser adherence and substance abuse (25.0% versus 4.6%) and serious adverse events (25.0% versus 21.3%, *p* < 0.001).

*Conclusions*. Aboriginal Canadians have higher levels of HCV risk factors; even when adjusting for area-level socioeconomic status markers. Despite facing greater barriers to care, SVR rates were comparable with non-Aboriginals.

*Funding Agencies: *CIHR, OHTN

# Sub article: A164: Direct Acting Antiviral Treatment Uptake in a Canadian HIV/Hepatitis C Co-Infected Population

**Authors:** S. Saeed, E. Strumpf, M. Klein

**Affiliations:** ^164.1^McGill UniversityMontrealQCCanada

*Background*. In Canada approximately 14,000 people are co-infected with HIV and Hepatitis C (HCV). Co-infection accelerates progression to end-stage liver disease and is now one of the leading causes of death in this population. To reduce the clinical and health system burdens of advanced liver disease, co-infected individuals need to be treated and cured of HCV. Fortunately highly effective and well tolerated direct acting antivirals (DAAs) are available in Canada.

*Aims*. The aim of this study was to describe DAA treatment uptake in a co-infected population.

*Methods*. Using data from the Canadian Co-Infection Cohort (CCC) Study, we investigated second-generation DAA treatment initiations until database closure (early 2015). The CCC is a prospective longitudinal cohort of 1498 HIV/HCV co-infected individuals from 18 centers, representing ~23% the total co-infected population in Canada. Socio-demographic, clinical and behavioural information is collected via self-administered questionnaires/chart review bi-annually.

*Results*. Overall, 39 people in 2010; 35 (2011); 37 (2012); 37 (2013); 59 (2014); 15 (until Feb 2015) initiated HCV treatment. A total 66 people initiated DAAs. Sofosbuvir/ledipasvir were the most frequently used DAA combinations accounting for 18 (27%) of treatments, followed by 17 (26%) sofosbuvir/ribavirin +/− peg-interferon (peg-IFN), 7 (11%) simeprevir/sofosbuvir, 1 (2%) simeprevir/ribavirin/(peg-IFN) the remaining 23 (35%) treatments, were accessed through clinical trials.  Table 25 compares demographic, clinical and behavioural characteristics of CCC participants who initiated second generation DAA therapy either by standard of care or through clinical trials and those eligible for treatment but who did not receive DAAs.

*Conclusions*. Health Canada approved simeprevir/sofosbuvir in late 2013, followed by ledipasvir, however provinces have been slow and restrictive at rolling out DAA treatment. Early signs do show that the absolute number of HCV treatments have increased 59% between 2013 & 2014, in the co-infected population. Sub-population under represented in this initial treatment uptake wave were; women, people of Aboriginal decent and people who inject drugs. Steps should be taken to increase the availability of treatment in these vulnerable populations.

*Funding Agencies: *CIHR, Can-Hep C

# Sub article: A165: Ultrasound Predicts Steatosis in Patients with Chronic Hepatitis B

**Authors:** E. Kelly, R. Hudock, V. Feldstein, M. Peters

**Affiliations:** ^165.1^Department of Medicine, The Ottawa HospitalOttawaONCanada; ^165.2^UCSFSan FranciscoCAUSA

*Background*. Inflammation and fibrosis may impair the ability of ultrasound to identify steatosis in patients with chronic hepatitis B (CHB).

*Aims*. We determined the accuracy of ultrasound (US) in grading steatosis in patients with CHB compared to liver biopsy, and examined clinical factors associated with steatosis.

*Methods*. This was a single-center, retrospective study of all non-transplanted CHB patients undergoing US and same day liver biopsies from 2004–2014. Steatosis was graded by ultrasound as 0: none, 1: mild, 2: moderate or 3: severe. Liver histology graded steatosis (0: <5%, 1: <33%, 2: <66%, 3: ≥66%), steatohepatitis, and staged fibrosis. Significant steatosis was defined as grade 2/3 for both biopsy and ultrasound. Obesity was defined as a body mass index (BMI) of >30 in non-Asians and >27 in Asians (Asia Pacific obesity classification). Clinical variables within 6 months of liver biopsy were collected and their association with steatosis analyzed by univariate logistic regression.

*Results*. We studied 109 patients with CHB with a median (IQR) age of 45 (37–54) and BMI of 25.3 (22.0–27.7). Patients were predominantly Asian (83%, *n* = 91), male (62%, *n* = 68), and HBeAg negative (62%, *n* = 45). 27% of patients (*n* = 30) were obese, 9% (*n* = 9) had diabetes mellitus, 23% (*n* = 25) hypertension, and 31% (*n* = 34) hyperlipidemia and 21 (19%) met the definition for metabolic syndrome. 44% (*n* = 48) of patients had any steatosis on liver biopsy; 8% (*n* = 9) had significant steatosis. The absence of steatosis on US had excellent specificity in ruling out biopsy steatosis (92%). Steatosis on US accurately predicted presence of significant steatosis (grade ≥ 2) on liver biopsy (sensitivity 89%, specificity 94%, *p* < 0.001). US imaging suggesting any amount of steatosis poorly predicted the presence of steatosis on liver biopsy (sensitivity 60%), but improved to 84% in obese individuals (*p* = 0.001). Predictors of biopsy steatosis on univariate analysis included diabetes (*p* < 0.001), hypertension (*p* = 0.03), hypercholesterolemia (*p* = 0.02), and BMI (*p* < 0.001).

*Conclusions*. Metabolic risk factors (diabetes, hypertension, hyperlipidemia and obesity) were common in our cohort and highly associated with steatosis. The absence of steatosis on ultrasound effectively ruled out steatosis on liver biopsy. Ultrasound may over-estimate mild steatosis in CHB, but had excellent sensitivity and specificity in identifying patients with significant steatosis on biopsy. Ultrasound accuracy in predicting any steatosis improved in obese individuals, including Asian Americans with lower BMI cutoffs. Abdominal ultrasound can be used to predict clinically important steatosis on liver biopsy in CHB patients which may assist counselling on lifestyle modifications and aggressive management of metabolic risk factors.

*Funding Agencies: *CIHR, None

# Sub article: A166: The Effect of PTEN on HCV Infection

**Authors:** Q. Wu, Q. Liu

**Affiliations:** ^166.1^University of SaskatchewanSaskatoonSKCanada

*Background*. Hepatitis C virus (HCV) infection causes serious global public health problems. There are more than 130 million chronic HCV patients worldwide. Hepatocellular carcinoma (HCC) is the most deadly clinical consequence of HCV infection. Phosphatase and tensin homolog deleted on chromosome 10 (PTEN) can suppress PI3K-AKT pathway, one of the most critical cancer-promoting pathways. PTEN is frequently mutated or deleted in tumors including HCC. However, the role of PTEN in HCV replication and pathogenesis is not well characterized. PTEN protein contains an N-terminal PIP2 (phosphatidylinositol-4,5-bisphosphate)-binding motif, a phosphatase domain, a C2 domain, a C-terminal tail containing two PEST (proline, glutamic acid, serine, threonine) sequences and a PDZ (PSD-95/DLG/ZO-1)-binding interaction motif at the end. Three naturally occurring mutations on the phosphatase domain disrupt PTEN's phosphatase activity: C124S mutation abrogates both lipid and protein phosphatase activity; G129E mutation abrogates lipid phosphatase only; and Y138L mutation abrogates protein phosphatase only.

*Aims*. To determine the effect of PTEN on HCV infection and the underlying molecular mechanisms.

*Methods*. We characterized HCV infection after PTEN overexpression or knocking down. We also determined whether PTEN interacts with HCV viral proteins as a mechanism for its effect on HCV infection.

*Results*. TEN negatively regulated HCV viral entry by using HCV genotype 2a pseudo-particles. We also observed that PTEN Y138L (protein phosphatase deficient) but not C124S (lipid and protein phosphatase deficient) nor G129E (lipid phosphatase deficient) inhibited HCV viral entry. Knocking down PTEN significantly enhanced viral replication; consistently, PTEN overexpression significantly inhibited HCV replication and secretion. Interestingly, PTEN C124S and Y138L could no longer inhibit HCV replication and secretion. We also observed that neither knocking down nor overexpressing PTEN affected HCV RNA translation. In co-immunoprecipitation and pull-down assays, we showed that HCV core protein interacted with PTEN. HCV core aa. R50 was required for the interaction. PTEN could no longer inhibit HCV genomic replication carrying core R50A mutation.

*Conclusions*. PTEN regulates HCV viral entry, replication, and secretion, but not translation. The lipid phosphatase activity of PTEN is required for inhibiting HCV entry. The protein phosphatase activity of PTEN is required for inhibiting HCV replication and secretion. HCV core interacts with PTEN, which contributes to PTEN's effect on HCV replication. Our study may help justify further development of PTEN as a new drug target for HCV therapy.

*Funding Agencies: *CIHR, CanHepC

# Sub article: A167: Estimation of Fibrosis Progression Rates for Chronic Hepatitis C: A Systematic Review and Meta-Analysis Update

**Authors:** A. Erman, T. Hansen, J. Bielecki, M. Krahn, R. Thein

**Affiliations:** ^167.1^Leslie Dan Faculty of PharmacyUniversity of TorontoTorontoONCanada; ^167.2^Department of MedicineUniversity of TorontoTorontoONCanada; ^167.3^Toronto Health Economics and Technology Assessment Collaborative (THETA)University of TorontoTorontoONCanada; ^167.4^Dalla Lana School of Public HealthUniversity of TorontoTorontoONCanada

*Background*. Chronic Hepatitis C viral infection (HCV) when left untreated is a leading cause of cirrhosis, liver failure, cancer and transplantation, making it a major medical and economic burden. Given the recent availability of highly effective but costly antivirals, accurate estimation of HCV-disease progression is essential for evaluating the cost effectiveness of treatment and determining treatment prioritization.

*Aims*. The purpose of this study was to obtain the most up-to-date stage-specific and stage-constant liver fibrosis progression rates (FPR) in individuals with chronic HCV infection though an updated systematic review and meta-analysis.

*Methods*. Literature search was conducted using MEDLINE, EMBASE and PubMed databases covering a period of January 1990 to August 2014 and supplemented by reference and citation searches. In general, the review included published English and non-English peer-reviewed prognostic studies which examine liver fibrosis progression in HCV-infected individuals. Publication bias was assessed by Funnel plots and Egger's test for asymmetry. Stage-constant FPRs were estimated for each study via the indirect method using the fibrosis score distribution and the estimated duration of infection reported in each study. Stage-specific FPRs (F_0-1_, F_1-2_, F_2-3_, F_3-4_) were estimated using the Markov Maximum Likelihood estimation (MMLE) method developed by Yi et al^1^. Random and fixed effects meta-analyses were used to obtain pooled stage constant and stage-specific FPR estimates.

*Results*. Overall, the updated systematic review included a total of 152 reports of HCV-infected individuals (*n* = 53,982). The pooled stage-constant FPR estimates derived through the indirect method were 0.086 (95% CI, 0.085–0.086) and 0.102 (95% CI, 0.098–0.0106) METAVIR units per year for the fixed and random effects models respectively. The stage-specific FPRs based on the random effects model were F_0-1_: 0.111 (95% CI, 0.101–0.122); F_1-2_: 0.087 (95% CI, 0.078–0.096); F_2-3_: 0.121 (95% CI, 0.110–0.132); F_3-4_: 0.115 (95% CI, 0.105–0.127).

*Conclusions*. The current study provides the most recent/updated estimates of both stage-constant and stage-specific liver disease progression rates associated with chronic HCV infections through an updated meta-analysis and systematic review. These results are consistent with the original study but suggest a slightly slower disease progression for stage-specific FPRs.

*Funding Agencies: *CanHepC

# Sub article: A168: Estimation of Transient Elastography Based Liver Stiffness Progression Rates for Chronic Hepatitis C: A Systematic Review and Meta-Analysis

**Authors:** A. Erman, A. Sathya, J. Bielecki, J. Feld, J. Hoch, R. Thein, M. Krahn

**Affiliations:** ^168.1^Leslie Dan Faculty of PharmacyUniversity of TorontoTorontoONCanada; ^168.2^Department of MedicineQueens UniversityKingstonONCanada; ^168.3^Toronto Health Economics and Technology Assessment Collaborative (THETA)University of TorontoTorontoONCanada; ^168.4^University Health Network University of TorontoTorontoONCanada; ^168.5^Institute of Health PolicyManagementand EvaluationTorontoONCanada; ^168.6^Dalla Lana School of Public HealthUniversity of TorontoTorontoONCanada

*Background*. Chronic hepatitis C (CHC) is a leading cause of liver fibrosis, cirrhosis, cancer and transplantation. Although liver biopsy is the gold standard for determining the degree of liver disease, limitations with regards to its invasive nature and diagnostic accuracy due to sampling errors and intra- and interobserver variability have led to the development of non-invasive methods; among which transient elastography (TE) is the most common. TE uses a measure of liver stiffness (LSM) as a surrogate for fibrosis. The accurate diagnosis of liver fibrosis is essential for decision-making in CHC. Given its increased utilization in clinical practice, there is still a need for accurate prediction of non-invasive diagnosis of fibrosis.

*Aims*. The aim of the study is to estimate TE-based liver stiffness disease progression rates (LSPR) in treatment naïve CHC patients through a systematic review and meta-analysis.

*Methods*. Literature search was performed using MEDLINE, EMBASE, CochraneCENTRAL trials as well as clinical trial registries. The search covered January 1990 to February 2015 with no language limit. Studies were included if they were full-length original studies of over 20 treatment naïve CHC patients undergoing TE-based evaluation. Studies were excluded if LSPRs could not be calculated due to missing data (i.e., LSM, duration of infection). LSPRs were obtained for each study through either a direct method using difference in serial LSMs and time interval between them (ΔLSM/Δtime) or an indirect method using a single LSM and the duration of infection (DOI) assuming a baseline of 5.33 kPa for healthy liver (ΔLSM/DOI). Heterogeneity was evaluated using *I* ^2^ statistic. Pooled direct and indirect LSPRs were estimated through a fixed and random effects meta-analysis.

*Results*. The review identified 31 reports for indirect and 8 for direct estimates. Based on random-effects model indirect LSPR was 0.141 kPa/year (95% CI. 0.115–0.166) and the direct LSPR was 0.137 kPa/year (95% CI. 0.169–0.205). LSPRs were generally higher for HIV/HCV coinfected versus monoinfected cohorts (Table 26 [Table tab27]).

*Conclusions*. Overall these data are generally consistent with liver biopsy studies but suggest slower progression. Estimating disease progression through non-invasive* Methods* may allow for alternative ways to model CHC and overcome some limitations of biopsies.

*Funding Agencies: *CanHepC

# Sub article: A169: Utility of Quantitative Hepatitis B Surface Antigen (QHBSAG) Compared to HBV DNA Testing for Predicting Maternal Viremia Associated with Mother to Child Transmission (MTCT) of HBV in a Multiethnic Cohort of Pregnant Chronic Hepatitis B (CHB) Carriers in Canada

**Authors:** G. Samadi Kochaksaraei, S. Congly, T. Matwiy, E. Castillo, S. Martin, C. Coffin, C. L. Charlton

**Affiliations:** ^169.1^University of CalgaryCalgaryABCanada; ^169.2^Provincial Laboratory for Public Health (Prov Lab)EdmontonABCanada

*Background*. HBV MTCT despite immunoprophylaxis is linked to high maternal viremia, hence nucleos/tide analog (NA) therapy in highly viremic mothers (HBV DNA ≥ 7 log IU mL^−1^) is recommended to reduce maternal HBV DNA levels to below the threshold associated with vaccine failure. Quantitative HBsAg (qHBsAg) is a new test proposed for management of CHB, but there is limited data in pregnancy.

*Aims*. To determine the utility of qHBsAg as a surrogate marker of HBV DNA.

*Methods*. CHB pregnant patients were recruited from a hepatology outpatient practice or an obstetrics internal medicine clinic. Demographics and laboratory data, HBV DNA and qHBsAg were assessed in the second-third trimester. Statistical analysis was performed by Spearman's rank correlation and student's *t*-test.

*Results*. 99 women with 103 pregnancies, median age 32 (IQR 29–35), 65% Asian, 23% African, and 12% other (Hispanic, Caucasian) were enrolled. Overall, 23% (23/99) were HBeAg (+), median ALT was 21 U/L (IQR 14–30.25), median HBV DNA and qHBsAg was 2.79 log IU mL^−1^ (IQR 1.9–3.95) and 3.52 (IQR 2.87–4.12), respectively. There was a statistically significant difference in qHBsAg in HBeAg positive (+) versus HBeAg negative (−) patients (4.38 log IU mL^−1^ (IQR 3.61–4.88) versus 3.33 log IU mL^−1^ (IQR 2.76–3.78), *p* < 0.05) and HBV DNA (7.76 log IU mL^−1^ (IQR 2.83–8.29) in HBeAg (+) versus 2.56 log IU mL^−1^ (IQR 1.81–3.19) in HBeAg (−), *p* < 0.05). In HBeAg (+) patients, a significant correlation between qHBsAg titer and HBV DNA level (*r* = 0.796, *p* < 0.05) was noted while there was no significant correlation between qHBsAg titer and HBV DNA level in HBeAg (−) group (*r* = 0.179, *p* = 0.065). In receiver operating characteristic (ROC) analysis, the optimal qHBsAg cut-off values for predicting HBV DNA level associated with immunoprophylaxis failure (i.e., HBV DNA ≥ 7 log IU mL^−1^) was ≥4.33 log IU mL^−1^ (accuracy 98.6%, sensitivity of 94.7% and specificity of 94.4% (95% CI, 97%–100%, *p* < 0.05). Based on the direct cost per test for qHBsAg ($28) and HBV DNA ($152); qHBsAg may be a more cost-effective test for predicting high maternal viremia.

*Conclusions*. Serum qHBsAg positively correlates with HBV DNA in HBeAg (+) CHB pregnant patients and may be a more cost-effective test in assessing maternal viremia, and the need for NA therapy to prevent HBV immunoprophylaxis failure, especially in resource-limited settings.

*Funding Agencies: *None

# Sub article: A170: Treatment of Mixed Cryoglobulinemic Vasculitis with Direct Acting HCV Therapy

**Authors:** J. Emery, M. Kuczynski, D. La, S. Almarzooqi, J. Feld

**Affiliations:** ^170.1^University of TorontoTorontoONCanada; ^170.2^University Health Network University of TorontoTorontoONCanada; ^170.3^UHNTorontoONCanada

*Background*. Mixed cryoglobulinemia (MC) is a lymphoproliferative disorder with a strong association to HCV infection. Manifestations of MC range from asymptomatic to life threating with HCV eradication leading to significant improvements in morbidity. Traditionally, clearance of HCV has required a combination of PEGinterferon and ribavirin which achieves sustained virological responses in 36–64% of patients. Importantly, remission of MCV symptoms is seen in over 80% of those achieving SVR. However, expectations of SVR rates and side effects profiles in the primary treatment of HCV have rapidly changed in the era of novel direct acting antivirals (DAA). Dramatic impacts on SVR rates have been reported (over 90%) and replicated but little has been published on their efficacy in the subpopulation with MCV.

*Aims*. To investigate the efficacy and safety of DAA in the treatment of Mixed Cryoglobulinemia.

*Methods*. Patients with immunological evidence of HCV related mixed cryoglobulinemia and prior treatment with direct acting antivirals were identified at tertiary care medical centre. Treatment response was evaluated based on clinical, immunological and virological outcomes at treatment cessation and at 12 weeks post treatment. Treatment side-effects, use of rescue therapy and decompensating events were recorded to confirm safety.

*Results*. Seventeen symptomatic and fifty non-symptomatic patients were reviewed. To date, SVR12 was achieved in ten (92%) symptomatic and twenty nine (93.5%) asymptomatic patients. At SVR12 full immunological response was achieved in four (40%) symptomatic and nineteen (59%) asymptomatic patients with five (33%) patients achieving full clinical response. One patient (14%) on PEG-IFN based regimens and three (44%) patients on interferon-free regimens had full clinical response rates. Full immunological response rates were seen in four (40%) patients on PEG-IFN and nineteen (60%) on IFN free regimens.

All fifty seven (100%) patients were able to complete therapy. Two (3%) patients had direct therapy related side effects (significant ribavirin related anemia) with four (6%) and five (7%) patients requiring hospitalization for decompensation or vasculitis.

*Conclusions*. Direct acting antivirals are efficacious in achieving sustained virological responses in symptomatic and asymptomatic patients with cryoglobulinemia. Immunological and clinical response rates in patients achieving SVR12 are suboptimal compared to previous reports, which may reflect shorter treatment courses or lower use of interferon. Longer follow up of our cohort is required to make adequate conclusions about clinical efficacy. Overall, use of DAA's in patients with cryoglobulinemia is well tolerated in symptomatic patients.

*Funding Agencies: *None

# Sub article: A171: National Hepatitis C Education Learning Needs Assessment

**Authors:** H. Shah, K. Seto, C. Klassen, D. Emokpare, B. Conway, M. Kelley, E. Yoshida

**Affiliations:** ^171.1^Canadian Association for the Study of LiverEdmontonABCanada; ^171.2^Canadian Liver FoundationMarkhamONCanada; ^171.3^Canadian Association of Hepatology NursesVancouverONCanada; ^171.4^Family PracticeReginaSKCanada; ^171.5^Vancouver Infectious Disease ClinicVancouverBCCanada; ^171.6^Memorial UniversitySt. John'sNFCanada

*Background*. Hepatitis C virus (HCV) is highly prevalent in Canada, with estimates of between 250,000 to 400,000 infected individuals. Recently, new therapeutic paradigms have increased the number of infected persons eligible for treatment. However, without adequate education of healthcare professionals (HCPs), necessary treatments may not be delivered to the infected population. To inform educational activities that will grow capacity for HCV care in Canada, a learning needs assessment is necessary.

*Aims*. To assess the current knowledge of healthcare professionals (HCPs) in the area of hepatitis C management, including screening and treatment using new antivirals, as well as the educational needs of HCPs, at a national level.

*Methods*. A survey was distributed by facsimile and email to more than 3500 individuals in the fields of hepatology, gastroenterology, nursing, other medical specialties and primary care physicians (PCPs). The survey consisted of 29 questions collecting participant and practice demographics, access to resources, screening habits, communication, knowledge of new treatments, and educational preferences. Partially completed surveys were accepted, with analysis based on the number of respondents for each question. Standard descriptive statistical methods were used.

*Results*. 163 participants answered all or part of the survey. The largest proportion were nurses with 69 individuals identifying as a nurse and 6 as a nurse practitioner. Others included hepatologists (20%), PCPs (15%), gastroenterologists (12%) and other specialists (7%). Most providers reported seeing patients with cirrhosis (80%) and past IDU was the main risk factor (60%). All hepatologists prescribed HCV therapy compared to 8% of PCPs. There was large variability in access to fibroscan and allied health by physician group. All provider groups were comfortable counselling HCV patients but hepatologists had the highest level of comfort with prescribing therapy across all severity of patients. Hepatologists reported higher perceived knowledge levels about new therapies. All participants welcomed association-sponsored medical education.

*Conclusions*. There is significant engagement amongst all providers in Hepatitis C. Many different provider types are involved in aspects of Hepatitis C care. To ensure growing capacity for HCV care in Canada, PCPs and nurses should be provided targeted education and resources to screen and treat alongside other specialists.

*Funding Agencies: *Canadian Liver Foundation

# Sub article: A172: A National Assessment on Knowledge and Attitudes of 644 Primary Care Providers to HCV Infection and Therapy in the Era of All-Oral Therapy

**Authors:** H. Shah, A. Ramji

**Affiliations:** ^172.1^University of TorontoTorontoONCanada; ^172.2^Gastrointestinal Research InstituteVancouverBCCanada

*Background*. It is estimated that 250,000 or 0.8% of the Canadian population are infected with chronic hepatitis C virus (HCV), the leading cause of economic loss and health burden due to any infectious disease. HCV is underdiagnosed, despite being the only curable chronic viral infection. There is a lack of data on the knowledge gaps and attitudes of primary care physicians (PCPs) in the era of all-oral and highly effective HCV therapy as well as systematic barriers to care. Past research in the interferon-era demonstrated wide ranges of competency and attitudes towards therapy. A survey just prior to widely available all-oral therapies demonstrated that 60% of PCPs were not very confident or only somewhat confident screening patients for HCV and 21% felt confident in initiating treatment.^1^ It is unclear what impact better therapies have on these knowledge levels and attitudes.

*Aims*. To evaluate the knowledge of, attitudes and barriers to care in HCV by Canadian PCPs.

*Methods*. A survey invitation (without incentive) was sent to Canadian PCP's via email and fax from an established database. Responses were analyzed using standard descriptive statistical methods.

*Results*. This is the largest Canadian survey of HCV in PCPs (*n* = 644). 69% of PCPs were in a group practice setting, 21% in a solo practice and 10% at a walk-in clinic. Every Canadian province was represented. Only 33.4% of PCPs indicated they had a good understanding or felt confident in screening/testing for HCV. 66.9% of PCPs knew that HCV was curable and 69.8% were aware of treatment options. The highest perceived barriers in relation to testing for HCV (PCPs could choose more than one answer) included lack of guidelines (33.2%), uncertainty on which patients to test (27.6%) and uncertainty on which lab tests to order (22.2%), while many responded that there were no barriers (42.6%). People born between 1945 and 1975 are considered at high risk of HCV, but only 33.9% of PCPs said they would screen this group. 48.6% noted specialist referral wait time was a barrier. Other barriers to referrals included unawareness of specialists treating HCV in their region (22.0%) and uncertainty in appropriate HCV-related laboratory tests prior to referral (25.0%). 27.5% of PCPs were interested in treating patients with HCV.

*Conclusions*. Despite advances in HCV therapy, primary care knowledge gaps in screening, referrals and management remain large. An educational program focused for PCPs has been developed based on the results of this survey.

*Funding Agencies: *Unrestricted grant from Gilead Sciences Inc.

# Sub article: A173: HCV Treatment of HIV-HCV Co-Infected PWID at a Tertiary Clinic

**Authors:** S. Hakobyan, S. Vafadary, T. Raycraft, S. Sharma, B. Conway

**Affiliations:** ^173.1^Vancouver Infectious Diseases CentreVancouverBCCanada

*Background*. There is an over-representation of people who inject drugs (PWID) among HIV-HCV co-infected adults. Recent data indicate that HCV treatment regimens are equally effective in the setting of HIV co-infection. However, within co-infected PWID populations, the feasibility (and success rates) of such therapy has not yet been clearly established outside of clinical trials. The aim of this analysis was to address this gap in knowledge.

*Aims*. Our goal was to identify, recruit, and retain HCV-infected PWID in care. As such, we established a multi-disciplinary program targeted at maintaining a lasting relationship with such individuals. We hypothesized that this approach would facilitate a long-term relationship with this vulnerable population.

*Methods*. This program includes facilitated access to specialty medical care, access to support services, comprehensive management of social needs, and addiction treatment. We have conducted a retrospective analysis of all HIV co-infected patients treated for HCV infection at our centre. This analysis correlates the likelihood of achieving sustained virologic response (SVR) following HCV treatment with a range of baseline demographic and clinical variables, including housing and active drug use.

*Results*. Of 522 HIV-infected individuals regularly seen at our centre, 247 (47.3%) were co-infected with HCV. Among the latter, 167 (67.6%) were PWID and 77 (31.2%) have completed HCV treatment (72 interferon-based, 5 all-oral regimens), 46 (59.7%) of which had genotype 1 infection. The mean age of treated patients was 51, 70 (90.9%) were male, 24 (31.2%) were on opiate substitution, 73 (94.8%) were on HIV treatment (62/73 with full virologic suppression), 21 (27.3%) were homeless, and 33 (42.9%) attended weekly HCV support groups. The SVR rate was 46.8% (36/77), 3/5 (60%) on all-oral regimens, 21/46 (45.7%) with genotype 1 infection, and 3/3 (100%) for patients with genotype 1 on all-oral regimens. Success rates were higher in subjects on methadone at 16/24 (66.7%), and no lower in those who were homeless 11/21 (52.4%) or active PWID 26/54 (48.1%).

*Conclusions*. PWID with HIV co-infection can be successfully treated for HCV infection within multi-disciplinary programs such as ours. Such programs will serve as an important tool to address the HCV epidemics in vulnerable populations often considered as “core transmitters" of HCV and HIV infection, especially as highly effective all-oral regimens become the standard of care.

*Funding Agencies: *None

# Sub article: A174: HCV Re-Infection in High-Risk People Who Inject Drugs

**Authors:** B. Conway, S. Vafadary, T. Raycraft, F. Zahedieh, S. Sharma, S. Hakobyan

**Affiliations:** ^174.1^Vancouver Infectious Diseases CentreVancouverBCCanada

*Background*. People who inject drugs (PWID) constitute the majority of cases of HCV infection in Canada. Although a number of strategies have been developed to engage them in care, reluctance to implement them relates at least in part to concerns about re-infection following successful HCV treatment. We have examined this issue in a prospective longitudinal cohort to establish whether this concern is confirmed in clinical practice.

*Aims*. The aim of this study was to assess the risk of HCV re-infection in a high-risk PWID population.

*Methods*. Within a multidisciplinary program to engage and treat PWID, we have documented 45 cases of HCV therapy having resulted in a sustained virologic response (SVR) in which patients continued to engage in high-risk behaviour for HCV acquisition after SVR was achieved. These individuals have been followed prospectively to document recurrent viremia, with the performance of HCV RNA testing every 6 months, more frequently if elevated ALT or symptoms of acute hepatitis were noted. The endpoint of this analysis is a positive HCV RNA test following the clear establishment of an SVR.

*Results*. Among the 666 HCV positive patients, with a mean age of 52.8 years, there were 51 (7.6%) females. Of these patients, 86 (12.9%) were co-infected with HIV, 419 (62.9%) had genotype 1, and 616 (92.5%) were previously treatment naïve. In a mean of 5.95 person-years of follow-up/subject, 4 cases of re-infection were noted (1.49/100 person-years) with all being HIV co-infected patients and 3 being genotype 1. The only factor associated with an increased risk of HCV re-infection was use of stimulants.

*Conclusions*. In our cohort, PWID successfully treated for HCV infection experience re-infection at a lower rate than previously encountered in uninfected at-risk individuals, and this negative outcome is often associated with stimulant use. The risk of HCV re-infection in individuals receiving care in multidisciplinary programs such as ours has probably been overestimated.

*Funding Agencies: *None

# Sub article: A175: Evaluation of Treatment of HCV Infection in People Who Inject Drugs

**Authors:** A. Alimohammadi, S. Sharma, S. Hakobyan, B. Conway

**Affiliations:** ^175.1^Vancouver Infectious Diseases CentreVancouverBCCanada

*Background*. Approximately 70% of HCV infected individuals in Canada are people who inject drugs (PWID). However, many healthcare providers require PWID to be drug-free for 6–12 months before commencing HCV treatment, fearing high reinfection rates. The aim of this study was to illustrate that HCV treatment could be successful in PWID in the right circumstances, without requiring a mandated period of abstinence.

*Aims*. The aim of this study was to assess the effectiveness of HCV treatment among active PWID populations.

*Methods*. A retrospective observational study was conducted in active PWID (currently injecting recreational drugs) receiving HCV therapy between 2011 and 2015 at a multi-disciplinary inner city clinic, favoring engagement and retention in care of the target population. Data regarding HCV treatment, HIV co-infection status, as well as demographic and social variables was collected. The primary endpoint was a sustained virologic response (SVR) with respect to HCV infection.

*Results*. We treated 40 eligible subjects (34 male) with a median age of 53 years, 24 (60%) genotype1a/b, 10 (25%) genotype 3, 33 (83%) previously treatment naïve, 11 (27.5%) co-infected with HIV. With respect to illicit drug use, there were 25 (63%) using heroin, 28 (70%) using cocaine, 9 (22.5%) using other stimulants and 23 (58%) on opiate substitution therapy. With respect to HCV therapy, 25 (63%) received IFN-based and 15 (37%) all-oral regimens. In total, 31 (78%) subjects achieved SVR, 17 (68%) and 14 (93%) on IFN-based and all-oral regimens (*p* < 0.05 favoring all-oral regimens). Within the study population, 7 (64%) with HIV co-infection, 18 (75%) with genotype 1, 9 (90%) with genotype 3, 21 (84%) on heroin, 21 (75%) on cocaine and 7 (78%) using other stimulants achieved SVR. Three (8%) discontinued due to toxicity and 4 (10%) experience a virologic Relapse. There were no cases of recurrent viremia with a mean follow-up period of 600 days.

*Conclusions*. Active PWID can be effectively treated for HCV infection with high SVR rates, especially with all-oral regimens. With structured post-treatment follow-up, rates of recurrent viremia can be minimized, enhancing the feasibility of programs to increase treatment uptake in high-risk populations of “core transmitters” of HCV infection.

*Funding Agencies: *None

# Sub article: A176: Differential Expression Profile of Circulating Micrornas Mir-24 and Mir-223 in Hepatitis C-Infected Patients Who Achieve a Treatment-Based Viral Cure

**Authors:** A. Hyrina, P. Steven, M. Krajden, E. Tam, F. Jean

**Affiliations:** ^176.1^University of British ColumbiaVancouverBCCanada; ^176.2^QIAGENManchesterUK; ^176.3^BC Centre for Disease ControlVancouverBCCanada; ^176.4^LAIR CentreVancouverBCCanada

*Background*. Hepatitis C virus (HCV) hijacks host lipid metabolic pathways as part of its replication cycle; this likely plays a role in viral pathogenesis. MicroRNAs (miRNAs) are small non-coding RNAs that silence gene expression by binding to mRNA transcripts and act as regulators of cellular processes. However, the function of miRNAs in lipid homeostasis during HCV infection and after viral clearance and their association with progressive liver disease are poorly understood. To help clarify the role of circulating miRNAs known to be involved in hepatocellular lipid metabolism, we determined circulating levels of three candidate miRNAs (miR-122, miR-24, and miR-223) from the plasma of HCV-infected patients undergoing interferon-based treatment.

*Aims*. In this study, we investigated how circulating levels of miR-122, miR-24 and miR-223 change during chronic HCV infection and after viral cure.

*Methods*. Circulating levels of miR-122, miR-24, and miR-223 from 94 patients with HCV were measured using qRT-PCR assays at multiple time-points before, during, and after interferon therapy. Liver fibrosis was scored by biopsy and/or FibroScan® before treatment. Serum HCV RNA levels and routine blood parameters were measured at each sample collection.

*Results*. We demonstrated that circulating miR-122 decreased after HCV viral clearance, correlating with the normalization of liver-specific enzymes (AST, *r* = 0.544, *p* ≤ 0.0001; ALT, *r* = 0.618, *p* ≤ 0.0001) and with a linear relationship to the liver injury APRI score, while miR-24 and miR-223 levels significantly increased after viral clearance (*p* ≤ 0.01). In contrast, circulating levels of miR-24 and miR-223 in patients who underwent an HCV treatment-based relapse remained unchanged, while miR-122 levels demonstrated a statistically significant increase in abundance (*p* ≤ 0.001). Quantitative correlation in amounts of circulating miR-24 and miR-223 was also detected (*r* = 0.91, *p* ≤ 0.0001) in relation to viral clearance but not in miR-122.

*Conclusions*. Our findings provide the first experimental evidence of upregulation of circulating miR-24 and miR-223 in plasma in HCV-infected patients who achieve an interferon-based virological cure. These results also reveal that miRNAs known to act as regulators of lipid metabolism may be correlated with interferon-based therapeutic outcomes in patients with HCV infection, suggesting that dynamic changes in the lipidome following viral cure as assessed by miRNA expression may be correlated with the risk of progressive liver disease.

*Funding Agencies: *Research Collaboration Agreement with QIAGEN Sciences LLC (No 14-0810 to F. Jean) and the National CIHR Research Training in Hepatitis C to A. Hyrina

# Sub article: A177: Hepatitis B Reactivation Prophylaxis for Patients Undergoing Chemotherapy for Lymphoma in Canada: Current Practice in Hematology/Oncology

**Authors:** G. Ou, K. Savage, L. Shepherd, J. Connors, E. Yoshida

**Affiliations:** ^177.1^University of BCVancouverBCCanada; ^177.2^Queen's UniversityKingstonONCanada

*Background*. Patients receiving cytotoxic chemotherapy have an increased risk of hepatitis B virus (HBV) reactivation and related hepatitis, which are associated with significant morbidity and mortality. Previous studies in the United States have demonstrated low rates of HBV screening and reactivation prophylaxis among patients undergoing chemotherapy.

*Aims*. To determine the current practice pattern of Canadian hematologists/oncologists in regards to screening for HBV infection and consideration of HBV reactivation prophylaxis for patients undergoing chemotherapy for lymphoma.

*Methods*. We conducted a survey in May 2015. Members of Canadian Hematology Society (*n* = 410) and NCIC Clinical Trials Group (*n* = 124) were invited by email to participate in an online, 9-multiple choice survey. Those with concomitant membership in both organizations received duplicate invitations.

*Results*. In total, there were 69 participants. 64/67 (96%) participants reported routine screening for HBV infection prior to chemotherapy. For the remaining participants, two physicians only screen patients with established risk factors for HBV; and another physician confined screening to patients with risk factors for HBV undergoing rituximab therapy. 64/67 (96%) participants routinely prescribe antiviral prophylaxis and/or consult another specialist for patients with positive HBV surface antigen (HBsAg) but no evidence of hepatic inflammation. However, only 51/66 (77%) participants routinely prescribe antiviral prophylaxis and/or consult another specialist for patients with negative HBsAg but positive anti-HBV core antibody (anti-HBc); two would prescribe prophylaxis if HBV DNA is also positive; and one would prescribe prophylaxis if rituximab is used in this setting.

*Conclusions*. Canadian hematologists/oncologists are screening and offering HBV prophylaxis to most of the patients at risk of HBV reactivation during chemotherapy. Future efforts should be directed at ensuring that all at-risk patients, including those with positive anti-HBc/negative HBsAg, receive appropriate prophylaxis.

*Funding Agencies: *None

# Sub article: A178: Access to New Hepatitis C Therapy: Do the Sickest Patients Receive Treatment? Practice Audit

**Authors:** J. Kiberd, G. Hirsch, C. Burgess, J. Igoe, M. Laryea, K. Peltekian, M. McLeod

**Affiliations:** ^178.1^Dalhousie University & Queen Elizabeth II Health Sciences CentreHalifaxNSCanada; ^178.2^Queen Elizabeth II Health Sciences CenterHalifaxNSCanada; ^178.3^DalhousieHalifaxNSCanada; ^178.4^Dalhousie UniversityHalifaxNSCanada

*Background*. Success rates of second generation DAA therapy for HCV have climbed to 95% with very few adverse effects. The course of treatment has been halved with most achieving viral eradication in just 12 weeks. However, this new oral regimen is costly. In Nova Scotia, a 12-week regimen of oral therapy for HCV is covered only in presence of fibrosis.

*Aims*. To determine if the patients who did not have third party coverage had worse liver disease compared to those who did have access through third party coverage. To determine the reasons that HCV infected patients are not on oral therapy in our center.

*Methods*. Retrospective chart review was performed on all patients who had documented HCV infection and had at least one-year follow-up between Apr 2014-15 in our clinic. New treatment status, sex, age, platelets, AST, ALT, INR, bilirubin, and creatinine were recorded. MELD, FIB4, and APRI scores were calculated and used to assess liver disease severity. Reasons for not having access to new therapy were “no third party coverage,” “alcohol/opioid dependence,” “considered palliative” or “Other.”

*Results*. 454 patients were identified with HCV infection. Fifty-one were removed because they did not have lab work or did not have coverage information resulting in 403 patients included in this audit. Sixty-two percent were male with a mean age of 55.8 years (95% CI: 54.8, 56.8). Cirrhotic patients made up 42% (168) of the sample as defined by low platelet count. Only 37% (148) of patients received new therapy. Median MELD score was 6 (IQR: 6, 8), median APRI was 0.75(IQR: 0.38, 1.62), and median FIB4 score was 2.06(IQR: 1.26, 4.28). High MELD score was predictive of new treatment (OR: 4.47, 95% CI: 2.02, 9.86). Both APRI and FIB4 scores were also predictive of new treatment (*p* = 0.01). Not having third party coverage was associated with lower MELD (*p* = 0.01) but not APRI and FIB4 scores. Of the 244 patients who were not on new oral therapy, 188 (77%) were due to lack of third party payer. 12 (5%) were deemed to have substance abuse and were not considered for treatment; while 12 (5%) were considered palliative due to competing health problems.

*Conclusions*. Patients who did not receive new DAAs had comparable liver disease severity to those who received drug. Patients who do have third party coverage had faster access to treatment as compassionate treatment was given to those only in the presence of severe liver disease.

*Funding Agencies: *None

# Sub article: A179: Laboratory Assessment of 1207 Hepatitis B Surface Antigen Positive Patients in Calgary, Alberta in 2014

**Authors:** K. Lau, A. Shaheen, T. Ricento, M. Swain, S. Lee, K. Burak, C. Coffin

**Affiliations:** ^179.1^University of CalgaryCalgaryABCanada; ^179.2^Alberta Health ServicesCalgaryABCanada

*Background*. Addressing chronic hepatitis B (CHB) infection is a priority for the Canadian health care system. Despite awareness of its prevalence and impact on morbidity and mortality, there is no data about how health care providers in Canada comply with current guidelines.

*Aims*. (1) In a 1-year period, we aim to characterize prevalence of Hepatitis B virus (HBV) infection and clinical status of all identified CHB carriers within a large urban Canadian centre (Calgary, Alberta).

(2) Assess if required laboratory investigations were appropriately done for identified HBV surface antigen (HBsAg) positive.

*Methods*. Based on Calgary Lab services administrative data, we identified all adult patients with positive HBV surface antigen within the Calgary Zone (Alberta Health Services), between January 1st and December 31st 2014, under an approved University of Calgary ethics protocol. All related demographic and relevant laboratory information on this cohort was extracted within 6 months of a positive HBsAg test. Non-parametric statistical methods were used for analyses.

*Results*. We identified 1207 patients with positive HBsAg in the Calgary area (48% female (582/1207), median age 44 (IQR 36–55), 4% patients (22/1207) hepatitis C virus co-infected). With a population of 1.2 million (municipal census) in 2014, the prevalence of CHB is 1%. In 51% (610/1207) with completed HBV E antigen (HBeAg) test, only 12% (75/610) were HBeAg positive. Overall 98% (1184/1207) had single alanine aminotransferase (ALT) testing within 6 months with median ALT 37 (IQR 25–55). 9.6% (114/1207) were found to have elevated ALT at single time-point, and 62% (71/114) with HBV DNA testing completed showed a median viral load of 2.8 log IU/mL (1.2–5.8). Among patients with normal ALT, 57% (608/1070) had HBV DNA testing and 89% (542/608) had low-level viremia (median HBV DNA 2.4 log IU/mL (1.2–3.3)). 59% (67/114) of patients with high ALT also had detectable HBV DNA and 39% (26/67) had HBV DNA levels that could potentially meet treatment criteria (73% male (19/26), median age 45 (IQR: 31–56)). Available referral/follow-up data show that overall 23% (277/1207) had seen a hepatologist within the previous 3 years.

*Conclusions*. Our results shows a higher prevalence (1%) of CHB in Calgary than the estimates for Canada (0.76%). More importantly, only a quarter (23%) of positive HBsAg patients are directed to specialists, suggesting that majority of patients lack appropriate care, surveillance, or treatment of HBV. Improvements in the current public health screening of HBV and subsequent referral to a hepatologist are necessary.

*Funding Agencies: *CIHR

# Sub article: A180: HCV Infection Causes Multiple Forms of Programmed Cell Death in Infected and Neighbouring Uninfected Cells

**Authors:** H. Kofahi, N. Taylor, M. Grant, R. Russell

**Affiliations:** ^180.1^Memorial UniversitySt. John'sNFCanada

*Background*. Chronic infection with HCV increases the risk of developing cirrhosis and hepatocellular carcinoma. Induction of programmed cell death (PCD) in the infected liver plays a role in the pathogenesis and studying it will help to understand the mechanism of development of these liver diseases.

*Aims*. To study the effect of HCV infection on inducing different forms of PCD in infected and neighbouring cells, and to analyze the mechanism by which it is induced.

*Methods*. Huh-7.5 cells were infected with the JFH1T strain of HCV. Cell viability was measured by MTT assay. DNA fragmentation was tested by staining with propidium iodide (PI) and detecting hypodiploid cells. Apoptosis was tested by detecting cPARP-positive cells and by inhibiting caspase-3. Bystander apoptosis was detected by co-culturing infected Huh-7.5 cells and S29 cells, then detecting cPARP-positive cells in the S29 cell population. Co-culturing infected Huh-7.5 and S29 cells in a transwell plate was used to test the role of cell-to-cell contact in the induction of bystander apoptosis. Induction of pyroptosis was tested by measuring the LDH activity and by staining them with the caspase-1-specific FAM-YVAD-FMK-FLICA. The induction of pyroptosis was confirmed by testing the effect of inhibiting caspase-1 on DNA fragmentation. Bystander pyroptosis was tested by staining a co-culture of Huh-7.5 and S29 cells with FAM-YVAD-FMK-FLICA.

*Results*. Infection reduced the viability of Huh-7.5 cells and induced DNA fragmentation. HCV infection increased the number of cPARP-positive cells. Inhibiting caspase-3 resulted in a significant decrease in DNA fragmentation, indicating that HCV infection induces apoptosis. cPARP-positive cells were found in both Huh-7.5 and S29 cell populations demonstrating the induction of bystander apoptosis. We could not detect any increase in the number cPARP-positive S29 cells in a transwell co-culture, indicating that direct cell-to-cell interaction is required for the induction of bystander apoptosis. Virus infection increased the activity of LDH in the supernatant of infected cells. The number of active-caspase-1-positive cells increased significantly following infection. Inhibition of caspase-1 resulted in a significant reduction in the number of hypodiploid cells, confirming the induction of pyroptosis in the infected population. A significantly higher number of S29 cells stained positive for active-caspase-1 when co-cultured with infected Huh-7.5 cells, providing evidence for the induction of bystander pyroptosis.

*Conclusions*. HCV infection induces two forms of PCD: apoptosis and pyroptosis directly and indirectly (bystander). Bystander apoptosis was found to be contact dependent.

*Funding Agencies: *CIHR

# Sub article: A181: The Community Pop-Up Clinic as a Tool of Engagement for Vulnerable Populations with HCV and HIV Infections

**Authors:** B. Conway, S. Vafadary, S. Sharma, F. Zahedieh, J. Shravah, T. Raycraft, S. Hakobyan

**Affiliations:** ^181.1^Vancouver Infectious Diseases CentreVancouverBCCanada

*Background*. The Downtown East Side Vancouver (DTES) is known for a high prevalence of HCV and HIV infection. Despite available services, significant numbers of patients remain undiagnosed or unengaged in care. There is a need to develop innovative structures to address this issue and understand the level of knowledge about infection and the interest to seek care.

*Aims*. The aim of the study was to evaluate the effectiveness of community pop-up clinics (CPCs) at DTES sites and assess the willingness of participants to receive care.

*Methods*. Participants were evaluated at community pop-up clinics (CPCs) held at DTES sites (including InSite, the only supervised injection facility in North America). HCV and HIV point-of-care testing was offered. Participants also completed targeted questionnaires to collect demographic information, knowledge about HCV infection, and desire to receive care. A $10 incentive was offered for participation.

*Results*. Since January 2014, 1,850 individuals (mean age 46.5, 82.0% male) were tested, with 631 (34.1%) infected with HCV including 57 (3.1%) co-infected with HIV. Of 840 PWID, 435 (51.8%) were infected with HCV, and 32 (1.7%) co-infected with HIV A total of 136 and 15 were not previously aware of being infected with HCV and HIV respectively. Participants identified HCV transmission as occurring through casual contact (14.1%), unprotected sex (36.9%), sharing needles (45.6%), sharing injection equipment (36.3%), or blood transfusion (42.2%). Only 37.1% were aware of curable treatment being available for HCV infection, and 53.7% would consider treatment for it where it was offered.

*Conclusions*. Despite the widespread availability of HIV and HCV services in DTES, our program identified 136 and 15 new cases of HCV and HIV infection and offered individuals the opportunity to engage in care. There is a significant gap in HCV transmission knowledge, but general willingness to receive care. Innovative low-threshold programs must be developed to engage those individuals in care.

*Funding Agencies: *None

# Sub article: A182: Hepatitis B and Hepatitis C Virus Case Finding in a Medium Security UK Prison

**Authors:** S. Patel, B. Clarke, G. Bird

**Affiliations:** ^182.1^Maidstone HospitalEssexUK; ^182.2^Maidstone HospitalMaidstoneUK

*Aims*. Chronic hepatitis B and C are common in prisons in the UK but there is no systematic testing of prisoners. We set out to assess the feasibility of blood spot testing for HBV and HCV in a medium security prison. A secondary objective was to evaluate the number of prisoners referred on and treated in the local liver clinic.

*Methods*. A walk-in blood spot service, advertised to prisoners, was provided in a medium security prison to undergo testing for hepatitis B and C. Follow up appointments at the local hospital for further assessment in all positive cases was arranged. All prisoners tested were notified of their results. Negative results or those who were antibody positive but PCR negative were informed by letter. Those with positive results were given an appointment to attend Prison Healthcare or were directly informed by our research nurse.

*Results*. 160 male prisoners were tested using the blood spot testing kit. The results show that 1.25% (*n* = 2) tested positive for Hepatitis B core antibody, whilst no prisoners tested positive for Hepatitis B surface antigen.

33.75% (*n* = 54) were HCV antibody positive. Those positive for HCV went on to be tested for HCV RNA (*n* = 38, 23.75%.) Of those HCV RNA positive, 50% (*n* = 19), 2.63% (*n* = 1), 42.10% (*n* = 16) were found to have the genotype 1,2 and 3 respectively. 5.26% (*n* = 2) could not be genotyped.

Of those that were HCV RNA positive, four (10.5%) were released with no forwarding address and four (10.5%) declined to attend healthcare. Thirty patients were offered a clinic appointment, five (13.2%) have started antiviral therapy and continue to be followed up in the liver clinic. 21.1% prisoners did not attend their appointment.

*Conclusions*. This study shows that blood spot HCV testing in prisons has the potential to identify chronic HCV cases. Conversely, there was no evidence of chronic HBV in our sample. Access to the liver clinic occurred in over half of the HCV cases and treatment initiated in only a small number of referrals. Obstacles to access and treatment included logistical problems in having patients attend the hospital clinic relating to staffing and transport availability. Furthermore, the routine of the prison service to move prisoners every 12–24 months can interrupt their availability for hospital attendance and continuity of healthcare.

Blood spot testing kits are easily obtainable and relatively cheap, their use is easy to learn and was accepted well by the prisoner population. Identification of chronic HCV in the prison population has the potential to reduce harmful behaviours, cross infection and provide treatment with antiviral therapy. The number of patients ultimately starting treatment was a relatively small proportion of those eligible, but these individuals would have not have otherwise been able to start antiviral therapy.

*Funding Agencies: *Gilead

# Sub article: A183: Characterization of HCV Infected PWID in the Setting of Clinical Care in Canada (CAPICA): A Retrospective Study

**Authors:** B. Conway, C. Cooper, J. Bruneau, J. Feld, L. Deshaies, C. Fraser, G. Macphail, J. Powis, C. Steingart, K. Stewart, R. Tomas, D. Webster, J. Cox, M. Drolet, M. Mcgovern

**Affiliations:** ^183.1^Vancouver Infectious Diseases CentreVancouverBCCanada; ^183.2^The Ottawa HospitalOttawaONCanada; ^183.3^Centre hospitalier de l'Université de MontréalMontrealQCCanada; ^183.4^University Health Network University of TorontoTorontoONCanada; ^183.5^Clinique Médicale St*-*David De L'AuberivièreLévisQCCanada; ^183.6^Cool Aid Community Health CentreVictoriaBCCanada; ^183.7^Calgary Urban Project Society (CUPS)CalgaryABCanada; ^183.8^Toronto Community Hep C ProgramTorontoONCanada; ^183.9^Sanguen Health CentreWaterlooONCanada; ^183.10^Saskatoon Infectious Disease Care NetworkSaskatoonSKCanada; ^183.11^Clinique médicale l'ActuelMontrealQCCanada; ^183.12^Dalhousie UniversitySaint JohnNBCanada; ^183.13^McGill University Health CentreMontrealQCCanada; ^183.14^Merck CanadaKirklandQCCanada

*Background*. HCV in people who inject drugs (PWID) represents a considerable healthcare and societal burden both in terms of morbidity and mortality. Current HCV treatment uptake in this population is low, possibly related to barriers at the level of patient, provider or the healthcare system. Data gathering in HCV PWID will provide insight into characteristics, which contribute to barriers for treatment uptake and can further be utilized for implementation of targeted medical and psychosocial intervention.

*Aims*. In this observational study, we will describe HCV disease in PWID currently followed in Canadian clinics. Demographics and health determinants, characteristics of illicit substance use, medical, psychologic and social co-morbidities, HCV disease characteristics, HCV treatment history and use of opiate substitution therapy (OST) will be obtained.

*Methods*. A multicenter, retrospective, randomly generated database/chart review will be performed to collect and summarize data on 450 patients from twelve centers across Canada. Subjects receiving medical care, with chronic HCV infection, and a history of injection drug use within twelve months will be included. Patients with HIV co-infection will be excluded.

*Results*. Data collection will be completed by December 2015. Demographics and health determinants, characteristics of the injection drug addiction, co-morbidities, HCV disease characteristics, HCV treatment and OST history of these patients will be described.

*Conclusions*. This study will allow us to better characterize HCV infection in PWID currently engaged in care in Canada. This information will help optimize protocols for HCV treatment in this important population.

*Funding Agencies: *Merck Canada Inc.

# Sub article: A184: Restrictions for Reimbursement of Direct-Acting Antiviral Treatment for Hepatitis C Virus Infection in Canada

**Authors:** A. Marshall, S. Saeed, L. Barrett, C. Cooper, C. Treloar, J. Bruneau, J. Feld, L. Gallagher, M. Klein, M. Krajden, L. Taylor, G. Dore, J. Grebely

**Affiliations:** ^184.1^The Kirby InstituteUNSW AustraliaSydneyNSWAustralia; ^184.2^Faculty of MedicineMcGill UniversityMontrealQCCanada; ^184.3^Faculty of MedicineDalhousie UniversityHalifaxNSCanada; ^184.4^Department of MedicineUniversity of OttawaOttawaONCanada; ^184.5^Centre for Social Research in HealthUNSW AustraliaSydneyNSWAustralia; ^184.6^CRCHUMUniversité de MontréalMontrealQCCanada; ^184.7^University of TorontoTorontoONCanada; ^184.8^Vancouver Coastal HealthVancouverBCCanada; ^184.9^BC Centre for Disease ControlVancouverBCCanada; ^184.10^Department of MedicineBrown UniversityProvidenceRIUSA

*Background*. Interferon-free, direct-acting antiviral (DAA) HCV regimens are highly effective, achieving sustained virologic response (SVR) above 90%. However, because the list price for these therapies is prohibitively high in Canada, universal drug coverage is a challenge.

*Aims*. The aim of this study was to appraise reimbursement criteria for the following HCV DAA regimens in Canada: sofosbuvir, ledipasvir-sofosbuvir, simeprevir, and ombitasvir-paritaprevir-ritonavir plus dasabuvir.

*Methods*. Reimbursement criteria for the four HCV DAA therapies were collected for ten provinces and three territories in Canada from April 22 to October 12, 2015. Data were extracted from health ministerial websites with a focus on: (1) minimum fibrosis stage required; (2) prescriber type restrictions; and (3) drug and alcohol use restrictions. Two investigators collected all data and then cross-checked responses.

*Results*. Depending on the HCV DAA therapy, 80–92% of provinces/territories limited access to persons with moderate fibrosis (≥F2 METAVIR or equivalent), and 25–55% of provinces/territories restricted prescriber type to specialists only. There were no drug and alcohol use restrictions. However, there were several inclusion/exclusion criteria that were left to the discretion of the physician (e.g., methadone or equivalent in prior 6 months).

*Conclusions*. This first review of HCV DAA funding eligibility criteria in Canada showed less reimbursement heterogeneity by jurisdiction compared to the United States. Nonetheless, substantial heterogeneity in provincial/territorial HCV reimbursement criteria exists, which could be minimised through the development and adoption of national management guidelines. Lastly, accessing criteria was challenging, supporting the need for greater transparency of information.

*Funding Agencies: *University International Postgraduate Award—UNSW Australia (Australia), Viral Hepatitis Clinical Research Program—Kirby Institute, UNSW Australia (Australia), CanHepC Trainee Scholarship (Canada)

# Sub article: A185: The Benefits of Onsite Transient Elastography Use in an Inner City Community Health Centre

**Authors:** C. Fraser, R. Milne, A. Drost

**Affiliations:** ^185.1^UBC Department of Family PracticeVancouverBCCanada; ^185.2^Cool Aid Community Health CentreVictoriaBCCanada

*Background*. The Cool Aid Community Health Centre is situated in the Access Health Centre (CACHC); a multi-agency building which is home to multiple services designed to reduce critical barriers to access of health and social services. At CACHC approximately 4,000 patients, 40% of whom are Hepatitis C (HCV) positive, receive comprehensive integrated primary health care in a low barrier, culturally competent setting. We have previously identified access to transient elastography (TE) or FibroScan (C) assessment as a significant barrier to HCV treatment for our clients. Over 2 years of referral to offsite specialist for assessment we obtained 68 scans with 28% lost to follow up for initial assessment or interpretation. With our pilot project of on-site assessment using a portable TE machine and immediate interpretation, we were able to obtain 117 scans with less than 3% loss to follow up over 3 days. This led to the acquisition of a permanent, on-site portable TE machine at our community health centre. We believe that this is the first community health centre in Canada to have integrated an on-site machine into primary care practice.

*Aims*. Retrospective analysis of the impact of on-site TE diagnostic testing within the context of primary care for an inner city cohort of individuals with chronic HCV. We will review the outcome of the first 300 scans performed by primary care clinicians in our clinic and evaluate the following: (i) percentage of patients retained in health services (ii) percentage of F2+ that initiated and completed treatment, achieved SVR (iii) time from referral to scan -time from scan to treatment start. Will include a subanalysis of HIV coinfection, patients that identify as First Nations or Aboriginal, and substance use.

*Methods*. Mixed qualitative/quantitative data review.

*Results*. We anticipate an increased understanding of the role of the therapeutic relationships in linking patients to care, and demonstrating the importance of ensuring the accessibily of diagnostic tools in community health centres and other low threshold settings. Based on preliminary data reviewed we anticipate increased retention in health care, increased engagement in HCV treatment as well as rapid progression through initial assessment and treatment start time.

*Conclusions*. Community health centres play an important role in the management and treatment of HCV as they are low threshold settings where trusted relationships exist with vulnerable individuals. The presence of onsite TE at CACHC improves patient engagement and retention in health care, streamlines HCV treatment assessment and initiation, and is easily integrated by primary care clinicians into routine practice.

*Funding Agencies: *Gilead Sciences

# Sub article: A186: Interferon and Interferon-Stimulated Gene Expression in Ciculating Immune Cells in Persistent Symptomatic and Occult Hepatitis C Virus Infections

**Authors:** T. Michalak, S. MacParland, T. Pham, A. Chen, C. Corkum

**Affiliations:** ^186.1^Memorial UniversitySt. John'sNFCanada

*Background*. Although the liver is the main site of hepatitis C virus (HCV) invasion and source of symptoms in hepatitis C, HCV also replicates in immune cells. The access to hepatic tissue for diagnostic and research purposes is inherently limited. In contrast, peripheral blood mononuclear cells (PBMC) and their subsets are readily available. In addition to symptomatic HCV infection, persistent asymptomatic HCV carriage, termed occult HCV infection (OCI), has been identified. Notably, the same immune cell subsets support HCV replication in both chronic hepatitis C (CHC) and OCI.

*Aims*. To determine relationships between different forms of HCV infection (CHC versus OCI), response and non-response to interferon (IFN)-based therapy, HCV load and replication status, and the immune cell expression of IFNs, interferon-stimulated genes (ISGs) and other genes relevant to antiviral immune responses. Also, to assess how exposure to exogenous IFN*α* impacts induction of ISGs in PBMC of patients with CHC and those of healthy individuals.

*Methods*. Gene expression and HCV RNA load and replication status in total PBMC and PBMC-derived T cells were evaluated by specific real-time RT-PCR assays.

*Results*. The studies showed that CHC and OCI are characterized by distinct IFN and cytokine gene expression profiles in PBMC, that an antagonistic relation exists between cell-associated HCV RNA and expression of IFN*α* and MxA (but not IFN*γ*), and that responsiveness to PegIFN*α*/Ribavirin therapy in CHC patients is associated with significantly higher expression of IFN*α*, IFN*γ* and IFN*λ* in PBMC and lower HCV loads in the cells. Also, expression of ISGs was found to be significantly augmented and less responsive to upregulation following IFN*α* treatment of PBMC from HCV-infected patients compared to PBMC of healthy controls.

*Conclusions*. Analysis of IFN and ISG expression profiles in circulating immune cells provide insights to HCV immunology and imply that functions of immune effector cell are altered in persistent both symptomatic and occult (asymptomatic) HCV infections.

*Funding Agencies: *CIHR

# Sub article: A187: Atlantic Multi-Organ Transplant Program Quality Improvement Project: Ischemic-Reperfusion Injury and Graft Dysfunction Post Liver Transplant

**Authors:** A. Khorasani-zadeh, S. Gruchy, M. Laryea, M. Walsh, K. Peltekian

**Affiliations:** ^187.1^Dalhousie UnivHalifaxNSCanada

*Background*. The incidence of graft dysfunction due to hepatic preservation injury (HPI) may be as high as 27% after deceased-donor liver transplant (LTx). The extent of hepatocellular damage is commonly assessed according to the opening aspartate aminotransferase (OAST) levels. The trends have not been documented in our Program.

*Aims*. This quality improvement project was designed to evaluate frequency, trends and outcomes of HPI as determined by measurement of the OAST levels in LTx recipients within Atlantic Canada (AC).

*Methods*. We used the Atlantic Multi-Organ Transplant Program (MOTP) database to extract data on our LTx patients between 2010 and 2015 (Table 28 [Table tab29]). Patient identifiers were removed and we used MINITAB for statistical analysis. Three groups of patients were compared according to the extent of HPI. Group 1 (Minor injury: AST < 1000 U/L), group 2 (moderate: AST 1000–5000 U/L), and group 3 (severe: AST > 5000 U/L). Postoperative HPI of the transplanted graft was estimated by peak values of the enzyme AST during the first 72 hours post surgery.

*Results*. There were a total 123 LTx in 115 patients, with 8 retransplants. OAST levels within the first 72 hours after LTx were 2,124 ± 2,274 (mean ± SD) U/L with a median of 1,220 U/L. The mean peak AST, deaths, retransplants, death or retransplantation and patient status (CanWAIT classification) are demonstrated in (Table 28). During the mean follow up of 913 + 639 days with a median 901 days, there were 25 deaths due to graft failures. Those with severe injury had death or graft failure of 38.9%, versus 29.1% in those with moderate injury and 20.0% in those with minor injury (Table 28).

*Conclusions*. OAST levels post LTx are a well known measure of hepatocellular injury due to ischemia-reperfusion. This quality improvement project will allow us to identify reversible factors that may reduce HPI and postoperative morbidity and mortality.

*Funding Agencies: *None

# Sub article: A188: Liver Injury Associated with Anti-TNF Therapy in Paediatric IBD

**Authors:** A. Ricciuto, B. Kamath, P. Church, T. Walters, S. Ling, A. Griffiths

**Affiliations:** ^188.1^The Hospital for Sick ChildrenTorontoONCanada

*Background*. Drug-induced liver injury (DILI) is a rare complication of anti-tumour necrosis factor (TNF) therapy. It has not previously been described in a paediatric inflammatory bowel disease (IBD) population, despite the widespread use of these biologics in children.

*Aims*. To report the frequency and outcomes of anti-TNF-associated liver injury in children with IBD at a tertiary paediatric centre, so as to test the hypotheses that it is an infrequent but serious occurrence and that anti-TNF discontinuation leads to recovery.

*Methods*. This is a single-centre retrospective review performed at the Hospital for Sick Children. Records of all IBD patients receiving anti-TNF therapy were reviewed in order to ascertain the frequency of DILI with follow-up until October 2015. Causality was assessed using the Roussel-Uclaf Causality Assessment Method (RUCAM).

*Results*. Of over 500 children and teenagers treated with anti-TNF antibodies for Crohn's disease and ulcerative colitis, 6 patients, all with Crohn's disease, were considered to have liver disease “possibly” related to anti-TNF therapy based on the RUCAM score. 5 were treated with infliximab (IFX) and 1 with adalimumab (ADA). Time from drug initiation to recognition of liver enzyme elevation ranged from 2.3 to 58.3 weeks. In all cases, the pattern of injury was hepatocellular without synthetic dysfunction, and all but 1 patient were asymptomatic. 2 patients underwent liver biopsy while on IFX. The first patient, with peak ALT 401, met criteria for “definite” autoimmune hepatitis (AIH) as per the Simplified Diagnostic Criteria for AIH. Cessation of IFX therapy was associated with prompt and marked improvement in liver biochemistry with near-normalization of ALT within 12 weeks. The patient has remained well off anti-TNF therapy. The second patient, with peak ALT 205 and GGT 102, displayed features potentially suggestive of early primary sclerosing cholangitis, including mild biliary duct dilatation and focal periductal fibrosis. However, liver enzymes normalized completely after IFX discontinuation and rose again to twice the upper limit of normal with its resumption. Furthermore, ANA titre increased while on IFX and decreased after drug cessation. Of the 4 patients in whom anti-TNF therapy was continued, 3 achieved liver enzyme normalization after widely variable intervals, up to 1.4 years. Also notable are the findings of at least one positive autoantibody in 5/6 patients and widely variable trough levels, suggesting no correlation between drug level and likelihood of liver injury.

*Conclusions*. The development of DILI in children receiving anti-TNF therapy is very rare. Nevertheless, triggering of autoimmune hepatitis can occur; early recognition and cessation of therapy are important.

*Funding Agencies: *CAG

# Sub article: A189: Unexplained Ascites in an Adolescent Female: Possible Association with Excessive Ingestion of Methylone

**Authors:** J. Stanisz, J. Terry, J. Zeidler, R. Issenman, H. Brill

**Affiliations:** ^189.1^Section of Pediatric GastroenterologyUniversity of CalgaryCalgaryABCanada; ^189.2^Department of Pathology and Molecular MedicineMcMaster UniversityHamiltonONCanada; ^189.3^Division of Gastroenterology and NutritionDepartment of PediatricsMcMaster UniversityHamiltonONCanada

*Background*. New substances have emerged as popular forms of achieving a psychoactive “high”. Synthetic cathinones, commonly marketed as “bath salts”, contain a number of amphetamine-like substances, which produce sympathomimetic effects and are powerful central nervous system stimulants. High doses of such agents, particularly MDMA (3,4-methylenedioxy-methamphetamine), can result in liver injury, presenting as abrupt onset of jaundice and fatigue with transaminase elevation. In rare cases, these agents can cause acute liver failure. Ascites with such agents has not been described.

*Aims*. We describe a case of possible association between methylone ingestion and ascites.

*Methods*. A case of unexplained ascites in an adolescent female was reviewed. The literature on amphetamine use and potential liver toxicities was explored and summarized.

*Results*. A 16 year old girl presented to hospital with progressive ascites and hepatosplenomegaly of unknown etiology. Liver enzymes, bilirubin and liver function tests were normal aside from albumin, which was transiently low. Ultrasound showed moderate ascites and hepatosplenomegaly. Infectious and autoimmune etiologies were ruled out. Ascites analysis was compatible with a transudative rather than an exudative process. A transjugular liver biopsy showed dilatation of the sinusoids and non-specific inflammation. A repeat core needle liver biopsy showed an unusual featureless non-refractile grey substance within the sinusoidal Kupffer cells and in macrophages present in the portal tracts. A sparse portal lymphohistiocytic infiltrate was present along with histologic features of portal hypertension. A drug history revealed that the patient had ingested a substance called “Pink Rock” in large quantities prior to the onset of her symptoms. This substance was provided for analysis and was identified as methylone (beta-keto-MDMA), a drug similar to the amphetamine derivative MDMA (3,4-methylenedioxy-methamphetamine). Her ascites resolved over the next few months with diuretic therapy and avoidance of the ingested substance.

*Conclusions*. In this case, we postulate that methylone or co-ingested substances led to blockage of the hepatic sinusoids with macrophages containing unidentified material assumed to have been used to “cut" the active drug, resulting in portal hypertension and ascites. This is the first case report identifying this effect with MDMA- or amphetamine-like agents.

*Funding Agencies: *None

# Sub article: A190: A Case Of Ramipril-Associated Cholestatic Liver Injury

**Authors:** T. Kulai, D. Forner, T. Arnason, M. McLeod

**Affiliations:** ^190.1^Dalhousie UniversityHalifaxNSCanada

*Background*. Angiotensin-converting enzyme inhibitors (ACEIs) are commonly used to treat hypertension. Rare reports of ACEI-induced hepatotoxicity have been described, most notably a cholestatic pattern of injury related to captopril.

*Aims*. A case of drug-induced cholestatic liver injury with ramipril is described.

*Methods*. Retrospective chart review and literature review.

*Results*. A 67-year-old male presented to the emergency department with a three-week history of jaundice and pruritis. Eight weeks prior, he began taking ramipril and clopidogrel after sustaining an ST-elevation myocardial infarct. Home medication changes included increased doses of bisoprolol and atorvastatin. Two weeks prior to presentation at the emergency room, he was seen by internal medicine with similar symptoms and atorvastatin was discontinued. Past medical history was significant for previous acute cholestatic liver injury 20 years earlier, which was attributed to methimazole after a negative workup for causes of liver disease. Physical examination revealed jaundice, but was otherwise unremarkable. Blood work demonstrated AST 47 U/L, ALT 46 U/L, total bilirubin 230 *μ*mol/L, direct bilirubin 176 *μ*mol/L, ALP 470 U/L, INR 1.4 and albumin 29 g/L. Abdominal ultrasound with Doppler and endoscopic retrograde cholangiopancreatography showed no bile duct obstruction. Work up for infectious and autoimmune causes was negative. Percutaneous liver biopsy showed marked cholestasis with minimal inflammatory activity and no fibrosis, most consistent with medication-induced cholestasis and similar to his previous pathology report from 20 years earlier. With discontinuation of ramipril, the patient demonstrated prolonged cholestatic hepatitis with partial biochemical improvement.

*Conclusions*. There are only four reported cases of ramipril-induced liver injury in the literature (two cholestatic, one unclassifiable and one mixed) and no documented cases of cross-hepatotoxicity between methimazole and ramipril. Cholestasis did not improve in the previous two cases until 6 weeks and 14 months after drug discontinuation, respectively. Here, causality assessment with the Council for International Organizations of Medical Sciences scale was 7 (probable). Three key mechanisms have previously been suggested as the cause of ramipril-induced hepatotoxicity, including metabolic interaction, hypersensitivity, and bradykinin-mediated effects, although no model currently exists to confirm these hypotheses. Similarly, the mechanism of methimazole-induced hepatotoxicity is unknown, although immune-mediated toxicity and reactive metabolite formation are suspected to be involved. Recurrent hepatotoxicity of the same phenotype (cholestasis in this case) is typical for patients with a second episode of drug-induced liver injury, regardless of the causative medication.

*Funding Agencies: *None

# Sub article: A191: Hepatic Ductopenia and Vanishing Bile Duct Syndrome following Anabolic Androgenic Steroid Use

**Authors:** R. Alkhiari, T. Xenodemetropoulos

**Affiliations:** ^191.1^McMaster UniversityAncasterONCanada; ^191.2^McMaster UniversityHamiltonONCanada

*Background*. Vanishing bile duct syndrome (VBDS) is a rare group of disorders that result in a progressive destruction of intrahepatic duct and hepatic ductopenia, highlighted by a significant reduction in the number of intrahepatic biliary ductsloss. It has been linked to a variety of etiologies, including idiopathic presentations, medication exposure, autoimmune conditions, graft versus host disease, infections, and malignancy.

Drug toxicity counts for 2–5% of hospitalized patients of jaundice. Cholestasis is usually resolved after discontinuation of the offending drugs but might persist and end up with VBDS, Multiple drugs has been reported in association with VBDS include antibiotics, NSAIDs, anticonvulsants, anabolic steroids, and others.

*Aims*. Case report.

*Methods*. Case report and Literature review.

*Results*. A 29-year-old- male presented with a 6 day history of progressive jaundice and severe pruritus. He was previously healthy and was not using medication. Five weeks prior to his presentation, he had started a cycle of an androgenic anabolic steroid for total of 4 weeks for body building. On examination, he was stable. abdomen was soft to palpation with mild tenderness in the right upper quadrant. He had significant scleral and dermal icterus.

Initial laboratory findings revealed significantly elevated total bilirubin of 131 mmol/L, conjugated 94.2 mmol/L, ALT 316 U/L, and within normal ALP. all other basic blood work were normal. Abdominal ultrasound was normal. patient was asked to discontinue all supplements, empiric ursodeoxycholic acid, with weekly blood work for monitoring.

Two weeks later, bilirubin was noted to have progressively increased to 828 mmol/L. The patient was admitted to hospital with recurrent nausea and anorexia. Comprehensive investigations were sent, including serology for viral hepatitis which all were negative. The patient was treated supportively and ultimately sent home with outpatient follow up.

Six weeks later, the patient demonstrated persistent symptoms of jaundice and severe pruritus. Despite his symptoms, the bilirubin level began to decline.

Given his clinical presentation, we proceded with liver bioposy which showd acute VBDS with marked ductopenia and severe hepato-canalicular cholestasis which were felt to be in keeping with medication-associated toxicity.

Over the subsequent 8 weeks, he experienced a progressive clinical and biochemical improvement with supportive treatment and a close monitor.

*Conclusions*. The corner stone of the management of acute liver injury in AAS is complete cessation of the offending agent and supportive management. Cholestyramine has been used empirically in many cases for management of pruritic symptoms. Almost all reported cases with acute liver injury improve over 3 to 12 months with supportive management.

*Funding Agencies: *None

# Sub article: A192: Detection of Ophthalmic Acid in Serum from Acetaminophen-Induced Acute Liver Failure Patients Is More Frequent in Non-Survivors

**Authors:** G. Kaur, E. Leslie, H. Tillman, W. Lee, D. Swanlund, C. Karvellas

**Affiliations:** ^192.1^University of AlbertaEdmontonABCanada; ^192.2^Medical University of South CarolinaCharlestonSCUSA; ^192.3^The University of Texas SouthwesternDallasTXUSA

*Background*. Acetaminophen (APAP) hepatotoxicity is related to the formation of* N*-acetyl-p-benzoquinone imine (NAPQI), which is detoxified through conjugation with reduced glutathione (GSH). Ophthalmic acid (OA) is an analogue of GSH in which cysteine is replaced with 2-aminobutyrate. Metabolomics studies of mice with APAP-induced acute liver failure (APAP-ALF) identified OA as a marker of oxidative stress and hepatic GSH consumption.

*Aims*. The aim of the current study was to determine whether OA is detectable in APAP-ALF human patients either early (day 2) or late (day 4) and whether OA levels were associated with in-hospital survival in the absence of liver transplant.

*Methods*. The aim of the current study was to determine whether OA is detectable in APAP-ALF human patients either early (day 2) or late (day 4) and whether OA levels were associated with in-hospital survival in the absence of liver transplant.

*Results*. Survivors had significantly lower admission bilirubin (4.2 versus 5.7 mg/dL) and lactate levels (3.3 versus 6.5 *μ*mol/L, *p* < 0.05 for all). During the first 7 days of the study, survivors were less likely to require mechanical ventilation (55% versus 88%), vasopressor support (9.8% versus 67%) or renal replacement therapy (26% versus 63%, *p* < 0.001 for all). Non-survivors were more likely to have detectable OA levels early (31% versus 15%, *p* = 0.034) and late (27% versus 11%, *p* = 0.02). However there were no significant differences in mean OA levels between non-survivors and survivors (early 0.48 versus 0.36, late 0.43 versus 0.37, *P* > 0.5 for all).

*Conclusions*. OA was detectable more frequently in APAP-ALF non-survivors but mean OA levels were not associated with survival. The routine clinical administration of* N*-acetyl cysteine could replenish GSH levels and prevent OA production.

*Funding Agencies: *Transplant Fund Value Added (University of Alberta)

# Sub article: A193: Prevalence of Non-Alcoholic Fatty Liver Disease and Relationship with Metabolic Syndrome Parameters in Canadian Bariatric Surgery Patients

**Authors:** K. Schwenger, B. Arendt, A. Teterina, S. Fischer, T. Jackson, A. Okrainec, J. Allard

**Affiliations:** ^193.1^University of TorontoHamiltonONCanada; ^193.2^UHNTorontoONCanada

*Background*. Non-alcoholic fatty liver disease (NAFLD) affects 74–98% of morbidly obese individuals undergoing bariatric surgery. While liver biopsies document non-alcoholic steatohepatitis (NASH) in 9.8% to 44%. It is not clear if these rates were documented before or after a very low calorie diet (VLCD) routinely prescribed before laparoscopic Roux-en-Y gastric bypass (RYGB).

*Aims*. This study aims to determine the prevalence of simple steatosis (SS) and NASH at the time of RYGB, post VLCD and assess the relationship between liver histology and metabolic syndrome.

*Methods*. Patients were recruited at the Toronto Western Hospital Bariatric Clinic. Patients' data was collected and a VLCD diet was prescribed for a duration of 1 week per 100 lbs body weight. Before RYGB, blood samples were collected to measure liver enzymes, insulin resistance (IR), diabetes status, and lipid profile; anthropometry was also measured. During the RYGB, a wedged liver biopsy was performed. Based on histology, patients were divided in 3 groups: normal liver (NL), SS and NASH. The groups were compared by Kruskal-Wallis test followed by Wilcoxon ranked sum, or chi-square and Fisher's exact test. Significance level was set at 0.05.

*Results*. 94 patients have been studied: 73% female, median age of 44 y, body mass index (BMI) of 46.8 kg/m^2^; the mean duration of the VLCD diet was 2.60 ± 0.66 (SD) weeks and weight loss per week of VLCD was 3.69 kg ±2.58. 59 patients (63%) had SS, 10 (10%) had NASH and 25 (27%) had NL. VLCD weight loss, BMI, lipid profile, ALP, fasting glucose, insulin, presence of type 2 diabetes and IR were not different between the three groups. ALT (median (min, max)) was significantly higher in NASH (56 (20, 138) U/L) versus SS (29.5 (1.5, 83) U/L) and NL (17.5 (9, 36) U/L); SS ALT was also significantly higher than NL. AST (37 (18, 120) U/L) was significantly higher in NASH versus NL (19 (14, 42) U/L). HbA1c was significantly higher in NASH (0.06 (0.05, 0.09)) compared to SS (0.06 (0.05, 0.07)) and NL (0.06 (0.04, 0.07)) (excluding 15 individuals on hypoglycemic medication). The proportion with IR was significantly greater in SS (93%) and NASH (100%) groups compared to NL (67%).

*Conclusions*. This is the first study on Canadian bariatric patients reporting on the prevalence of NAFLD. In our population considering the VLCD routinely used prior to RYGB, the prevalence of NASH was low (10%) while SS is within range when compared to published reports. The proportion of patients with IR was higher in NAFLD but IR was also present in 67% of patients with NL. This suggests that mechanisms other than IR contribute to NAFLD in this population.

*Funding Agencies: *CIHR

# Sub article: A194: Pre- and Post-Operative Prognostic Value of Cardiopulmonary Exercise Testing in Liver Transplant Candidates: A Systematic Review

**Authors:** M. Ney, B. Vandermeer, P. Tandon, M. Haykowsky

**Affiliations:** ^194.1^University of AlbertaEdmontonABCanada; ^194.2^University of Texas at ArlingtonArlingtonTXUSA

*Background*. We need better tools to predict mortality pre- and post-liver transplantation. The MELD and Child Pugh scores are limited by their liver centric focus and inability to account for functional capacity. Cardiopulmonary exercise testing (CPET), the gold-standard measure for assessing functional capacity has shown promise as an independent prognostic tool in several large cirrhosis studies. To date however, this information has not been incorporated into clinical guidelines or practice.

*Aims*. We aimed to systematically review and if possible, meta-analyze studies evaluating CPET in adult patients with cirrhosis being assessed for liver transplantation.

*Methods*. Inclusion criteria: (i) English language, (ii) 100% with cirrhosis, (iii) age ≥ 18, and (iv) pre-transplant CPET testing. Studies were excluded if no clinical outcomes were reported in association with CPET testing.

*Results*. A total of 7 studies and 1107 patients were included. The primary etiology of liver disease was alcohol (46%). Three studies reported pre-transplant mortality, all showing a significant predictive value but all using different cut-points for CPET testing (i.e. ≥ 60% predicted VO_2_ versus mean AT and peak VO_2_). Seven studies reported post-transplant mortality with heterogeneous prognostic utility. These studies were heterogeneous with regards to when the outcome was measured and how data were reported. Three of the seven studies with post-transplant data were amenable to pooling to determine the diagnostic capacity of the pre-transplant peak VO_2_ and AT readings but were too heterogeneous for further analysis. Five of 7 studies were significant for peak VO_2_ as a predictor of post-transplant mortality. Three out of 4 studies assessing AT found it to be a significant predictor of post transplant mortality. The receiver operating curve analysis (ROC) of the 4 amenable studies demonstrated that peak VO_2_ was a sensitive (0.85, 95% CI 0.65–0.95) but not specific (0.58, 95% CI 0.42–0.73) predictor of post transplant mortality (Figure 22).

*Conclusions*. There is a large amount of evidence supporting the prognostic utility of CPET data to optimize the prediction of pre- and post- liver transplant outcomes. In the current analysis, peak VO_2_ is a sensitive but not specific marker of post-transplant mortality. High heterogeneity and variable data reporting methods prohibit a traditional pooled meta-analysis. An individual patient data meta-analysis would be the ideal next step to standardize the baseline prognostic factors, relevant CPET cut-points, timing of clinical outcomes and statistical analyses across studies and clarify the role of CPET as a predictive tool in liver transplantation.

*Funding Agencies: *None

# Sub article: A195: Nonalcoholic Steatohepatitis Diagnosed by the Serum Biomarker Cytokeratin 18 Is Frequent In HIV Infected Patients and Is Associated with Liver Fibrosis by Transient Elastography

**Authors:** G. Sebastiani, L. Lennox, C. Pexos, B. Lebouche, M. Klein

**Affiliations:** ^195.1^McGill University Health CenterMontrealQCCanada

*Aims*. Nonalcoholic steatohepatitis (NASH) is a leading cause of end-stage liver disease and the third indication for liver transplantation in Canada. HIV infected patients are at high risk of NASH due to metabolic comorbidities, long-lasting use of antiretroviral medications, chronic inflammation related to HIV. Nevertheless, due to the invasiveness of liver biopsy, data on NASH in HIV mono-infected patients are scarce. No study has employed cytokeratin 18 (CK-18), a validated biomarker for the non-invasive diagnosis of NASH, and transient elastography (TE) with controlled attenuation parameter (CAP) to dissect prevalence and cofactors of NASH in HIV mono-infected patients without hepatitis B or C.

*Methods*. This was a prospective cohort study of consecutive HIV mono-infected persons enrolled at McGill University Health Centre. Patients with significant alcohol intake or coinfection with hepatitis B or C were excluded. NASH was diagnosed by CAP >232 dB/m and CK-18 level >246 U/L. Significant liver fibrosis and cirrhosis were defined as TE measurement >8 kPa and >13 kPa, respectively. A subgroup of patients with a non-invasive diagnosis of NASH underwent liver biopsy. Spearman's rho was used to investigate correlations between CK-18 and other factors. Cofactors associated with NASH were determined by multivariate logistic regression models.

*Results*. Overall, 122 HIV mono-infected persons (mean age 51.6 years, 80.8% men) were included. Median CK-18 levels were 90.7 U/L (IQR 54.9–138.2). Prevalence of NASH was 9.8%. Significant liver fibrosis and cirrhosis were found in 9.1% and 2.5% of cases, respectively. Liver histology was requested in 10 out of 12 patients with a non-invasive diagnosis of NASH and it confirmed such a diagnosis in all cases. Serum CK-18 levels correlated with CAP value (*r* = 0.21, *p* = 0.02), TE measurement (*r* = 0.34, *p* < 0.001) and ALT (*r* = 0.59, *p* < 0.001). After adjusting for age and BMI, elevated ALT (odds ratio = 48.85, 95% CI 5.24–455.27; *p* = 0.001) and TE measurement (odds ratio = 1.31, 95% CI 1.02–1.69; *p* = 0.03) were independent predictors of NASH. Moreover, after adjustments for BMI and albumin, NASH was an independent predictor of significant liver fibrosis (odds ratio = 23.49, 95% CI 2.75–200.85; *p* = 0.004).

*Conclusions*. NASH diagnosed by cytokeratin 18 and CAP is frequent in HIV mono-infected persons, particularly in those with elevated ALT and higher TE measurement. Importantly, NASH diagnosed by CK-18 and CAP is a predictor of significant liver fibrosis by TE. Longitudinal studies are needed to evaluate the impact of non-invasive screening strategies and interventions aimed at reducing morbidity and mortality due to liver disease in this population.

*Funding Agencies: *CIHR Canadian HIV Trials Network; unrestricted research funds from Merck, study number IIS#51841

# Sub article: A196: Effectiveness of a Multi-Disciplinary Clinic in Treating Low-Risk NAFLD

**Authors:** S. Jayakumar, M. Swain, A. Shaheen

**Affiliations:** ^196.1^University of CalgaryCalgaryABCanada; ^196.2^Univ CalgaryCalgaryABCanada

*Background*. NAFLD is currently the leading cause of elevated liver enzymes in North America, but few patients have much knowledge regarding their disease. We established a non-physician led multi-disciplinary clinic, where patients deemed to be at low risk for disease progression attend a half-day education workshop.

*Aims*. To assess patient knowledge and satisfaction with a multidisciplinary workshop for the management of low-risk NAFLD, and assess how this compares to standard of care.

*Methods*. Patients initially triaged as NAFLD are randomized to be seen by a hepatologist or to have further work-up to assess if they are possible candidates for the education workshop. Patients randomized to potentially attend the workshop are stratified as low risk based on bloodwork, past medical and social history, and Fibroscan. Low risk patients attend a 3 hour education workshop. They are asked to fill out a survey pre- and post-workshop to assess patient knowledge and satisfaction. Surveys are also sent to the referring physician to assess physician satisfaction. Both patients and the referring physician are given a NAFLD pamphlet, test results, guidelines for management, and when to re-refer to hepatology. Patients attending a hepatologist clinic visit are given similar surveys to assess patient knowledge and satisfaction, as the standard of care comparator.

*Results*. In a period of 1 year (October 2014–October 2015), 136 patients were seen in the education workshop. There were 6 surveys without a before/after matching survey. Prior to the workshop, 99 patients felt that they had low knowledge (score 1–3 out of 5) about their disease, whereas afterwards only 5 patients scored their knowledge as low. Prior to the workshop, 80 patients felt comfortable with their referring physician managing their disease, compared to 116 patients after the workshop. 120 patients expressed satisfaction with the overall education workshop (defined as a score of 4 or 5 out of 5). Results from the comparator (standard of care) surveys are still pending.

*Conclusions*. In patients with low-risk NAFLD, a non-physician based, multi-disciplinary workshop results in increasing patient knowledge and patient satisfaction.

*Funding Agencies: *None

# Sub article: A197: Safety Profile of Liver Fibroscan in Patients with Cardiac Pacemakers and Implantable Cardioverter-Defibrillators

**Authors:** Y. Chan, S. Pranke, S. Nosib, L. Worobetz

**Affiliations:** ^197.1^University of SaskatchewanSaskatoonSKCanada; ^197.2^Royal University HospitalSaskatoonSKCanada

*Background*. Right heart failure and amiodarone exposure are known causes of cirrhosis. Conversely, emerging evidence suggests that non-alcoholic fatty liver disease (NAFLD) is associated with coronary artery diseases and arrhythmias. However, the FibroScan (Echosens, France), a widely-available, non-invasive device to detect liver fibrosis and steatosis is currently contraindicated in patients with cardiac pacemakers (PM) or implantable cardioverter-defibrillators (ICD).

*Aims*. To determine the safety profile of liver FibroScan in patients with PM or ICD and the prevalence of cirrhosis in this population.

*Methods*. Consecutive outpatients undergoing routine device interrogations at a tertiary level teaching hospital underwent liver FibroScan with concurrent cardiac device monitoring using FibroScan M or XL probe as per manufacturer's guidelines. PM or ICD performance data, device types, patient demographics, and medical history were collected. Cutoff values for significant fibrosis (F2–4), cirrhosis (F4), and massive steatosis were set at kPA >9, kPA >14, and >300 dB/m, respectively.

*Results*. Interim analysis of the first 61 of 200 planned subjects, with 27 models of implanted cardiac devices from 5 companies (Medtronic, Sorin, ELA Medical, Boston Scientific, St. Jude), did not demonstrate any adverse events as defined by abnormal device sensing/pacing or ICD firing. This population included subjects undergoing active pacing (*n* = 27, 44%) and with right sided PM placement (*n* = 1). None of the subjects had any clinical signs of decompensated congestive heart failure or cirrhosis during the exam. Based on their Fibroscan readings, the prevalence of clinically significant fibrosis (F2–F4), cirrhosis (F4), and massive steatosis (S3) were 26% (*n* = 16), 15% (*n* = 9), and 23% (*n* = 14).

*Conclusions*. Liver FibroScan can be safely used in patients with PM or ICD. Preliminary results suggested the prevalence of significant liver fibrosis and cirrhosis is in this cohort as compared to the general population.

*Funding Agencies: *None

# Sub article: A198: Can Serological Markers Be Used to Better Define Primary Biliary Cholangitis-Autoimmune Hepatitis Overlap Syndrome?

**Authors:** H. Nguyen, A. Shaheen, M. Fritzler, M. Swain

**Affiliations:** ^198.1^University of CalgaryCalgaryABCanada; ^198.2^Univ CalgaryCalgaryABCanada

*Background*. Autoimmune liver diseases (AILD), including Autoimmune Hepatitis (AIH) and Primary Biliary Cholangitis (PBC), are characterized by a constellation of clinical, biochemical (including autoantibodies) and histological features that can facilitate diagnosis. However, there are patients that harbor features of more than one AILD; called “Overlap Syndromes" (OS). It is estimated that up to 18% of patients with PBC can be classified as having overlap features of PBC-AIH. The recognition of PBC-AIH OS is important for the prognostication and treatment of this condition. Specifically, PBC-AIH OS patients have an increased frequency of cirrhosis and can exhibit suboptimal response to Ursodeoxycholic acid therapy when compared to patients with PBC alone. Various serological markers, including anti-double stranded DNA (anti-dsDNA) and anti-P53, have been previously suggested to be robust markers for identifying PBC-AIH OS.

*Aims*. We intend to evaluate the utility of various serological markers (including anti-dsDNA and anti-P53) for their ability to identify PBC-AIH OS in our well defined PBC patient cohort.

*Methods*. Stored blood samples from 109 PBC patients were analyzed by Mitogen Diagnostic Laboratory (Calgary) for a number of serological markers, including anti-dsDNA, anti-P53, anti-Ro52/TRIM21, anti-YB1, anti-MPP1, GW182, GE-1, and Ago2. Patient serum serological profiles were then compared to clinical data obtained from retrospective patient chart reviews (including patient demographics, primary diagnosis, biochemical profile, documentation of PBC-AIH OS, and degree of liver fibrosis).

*Results*. A total of 109 PBC patient charts were analyzed and matched to serological data. The mean age was 65.3 years (range 36 to 90 years). 92.7% of the patients were female versus 7.3% males. 6.4% (7/109) of patients fulfilled biochemical and histological criteria for the diagnosis of PBC-AIH OS. Anti-dsDNA was found in 28.6% of AIH-PBC OS patients using the Crithidia luciliae immunofluorescent assay, but in 0% when a chemiluminescence immunoassay was used. Anti-P53 was found in none of the PBC-AIH OS, but was positive in 28.4% of patients without OS. Anti-Ro52/TRIM21 was found in 71.4% of PBC-AIH OS patients versus 26.5% of those without OS. Further multivariate analysis is pending.

*Conclusions*. In contrast to previous reports, our findings do not support the utility of anti-dsDNA or anti-P53 as useful serological markers for PBC-AIH OS. The detection of anti-dsDNA in this OS cohort was highly assay dependent. However, anti-Ro52/TRIM21 may be useful in the identification of PBC-AIH OS and warrants further study. Further analysis is expected to highlight additional potential associations between serological and clinical variables in PBC-AIH OS.

*Funding Agencies: *CIHR

# Sub article: A199: Mean Corpuscular Volume Serves as Marker for Therapeutic Levels of Azathioprine in Autoimmune Hepatitis

**Authors:** R. Bhanji, A. Czaja, A. Mason, M. Ma, V. Bain, A. Montano-Loza

**Affiliations:** ^199.1^University of AlbertaEdmontonABCanada; ^199.2^Mayo Clinic College of MedicineRochesterMNUSA; ^199.3^Los Alamos National LaboratoryEdmontonABCanada

*Background*. Azathioprine in combination with prednisone is the first line treatment of autoimmune hepatitis (AIH). The former is either given as a fixed dose of 50 mg daily or as weight-based dosing of 1-2 mg/kg/day.

*Aims*. The purpose of the current study was to determine if the mean corpuscular volume (MCV) correlates with levels of 6-thioguanine nucleotides (6-TGN), and if MCV correlates with the treatment response.

*Methods*. We analyzed 40 patients with definitive diagnosis of AIH according to the international criteria who were being treated with azathioprine. All patients had liver biopsy at diagnosis and treatment responses were graded as complete and incomplete response. Fifty-four blood samples were tested for 6-TGN. Samples were analyzed for 6-TGN by reverse phase high-performance liquid chromatography and concentrations were expressed as pmol/8 × 10^8^ RBCs. Therapeutic levels were considered between 310 and 750 pmol/8 × 10^8^.

*Results*. Mean age was 47 ± 17 years and 25 patients were females (63%). Six-TGN ranged from 27 to 980 pmol/8 × 10^8^. There was a positive correlation between MCV and 6-TGN level (*r* 0.47, *P* < 0.001; Figure 23(a) [Fig fig24]). The area under the curve using ROC analysis for levels of MCV and therapeutic levels was 0.85 (95% CI 0.72–0.97, *P* < 0.001; Figure 23(b)). A cutoff value of MCV ≥ 96 had 88% sensitivity and 78% specificity for therapeutic levels of 6-TGN. Azathioprine dose was not related to 6-TGN levels (*p* = 0.5). Lastly, excluding patients with diagnosis of cirrhosis (*n* = 4), patients with 6-TGN in therapeutic levels (85% versus 46%, *P* = 0.004) and MCV ≥ 96 had higher frequency of complete remission at the time of 6-TGN determination (74% versus 42%, *P* = 0.04).

*Conclusions*. MCV has a positive correlation with 6-TGN levels and might be useful in clinical practice as a surrogate marker to determine if patients achieved therapeutics levels of azathioprine.

*Funding Agencies: *None

# Sub article: A200: A Novel Gene Mutation in ABCB11 in Siblings with Progressive Familial Intrahepatic Cholestasis Type 2

**Authors:** J. Griffin, A. Mhanni, J. Hartley

**Affiliations:** ^200.1^Department of Pediatrics and Child HealthWinnipegMBCanada; ^200.2^Department of Biochemistry and Medical GeneticsWinnipegMBCanada

*Background*. Progressive familial intrahepatic cholestasis (PFIC) is an autosomal recessive disease that causes severe liver disease often leading to liver failure. PFIC type 2 is caused by mutations in ABCB11, a gene which encodes a hepatocellular transporter protein called the bile salt export pump (BSEP). This transporter moves bile salts out of hepatocytes. In PFIC type 2, the build up of bile salts in hepatocytes leads to liver dysfunction and can cause liver failure.

*Aims*. To describe a novel mutation in ABCB11 in two siblings of Canadian Aboriginal Cree ancestry correlating with PFIC type 2 phenotype.

*Methods*. A retrospective chart review of two siblings with PFIC type 2. Sibling A was initially diagnosed with idiopathic cirrhosis leading to liver failure. She had complications including renal failure, Vitamin D deficient rickets, portal hypertension with gastrointestinal bleeding, malnutrition, ascites, encephalopathy, and spontaneous bacterial peritonitis. She underwent liver transplantation at 8 years of age.

Sibling B initially presented at 6 months of age with rickets. He was also found to have renal tubular acidosis, elevated liver enzymes and had a history of neonatal cholestasis. He had ongoing issues with failure to thrive, steatorrhea and pruritus. The gamma-glutamyl transpeptidase (GGT) levels always remained normal. He underwent liver biopsy, which showed giant cell transformation, portal tract expansion, and lobular inflammation. He subsequently underwent genetic testing for PFIC type 2.

*Results*. Sequencing of ABCB11 in both siblings revealed a novel missense mutation, c.2471G>A (p.G824E) and a deletion, c.3765 (+1_+5) del 5. The missense mutation is suspected to be pathogenic based on its location in a conserved functional domain, and in silico analyses. The deletion is predicted to affect RNA splicing. Parents were unavailable for carrier confirmation.

*Conclusions*. The findings on liver biopsy, combined with the phenotypic characteristics of both siblings indicate that these novel mutations in ABCB11 are associated with PFIC type 2 disease in these siblings of Cree ancestry. Hepatologists and geneticists need to be aware of this mutation for future patients who may present with similar phenotypes.

*Funding Agencies: *None

# Sub article: A201: Combination Tenofovir/Emtricitabine and Lopinavir Provides Durable Biochemical Responses for Patients with Primary Biliary Cholangitis (PBC)

**Authors:** E. Lytvyak, A. Mason

**Affiliations:** ^201.1^University of AlbertaEdmontonABCanada

*Background*. We have recently shown that the majority of patients with primary biliary cholangitis (PBC) have evidence of human betaretrovirus proviral integrations in their biliary epithelium, justifying the use of antiviral therapy to treat disease. In mouse models of PBC with betaretrovirus infection, combination reverse transcriptase inhibitors and HIV protease inhibitors have demonstrated utility in abrogating cholangitis. Furthermore, combination lopinavir/ritonavir (LPRr) and tenofovir/emticitabine (TDF/FTC) has been reported to be efficacious in normalizing liver tests within 12 months in a patient with HIV, human betaretroviurs infection and PBC (Lancet 2011).

*Aims*. To assess whether 24 months therapy with TDF/FTC and LPRr can normalize hepatic biochemistry in patients with unrepsonsive to ursodiol.

*Methods*. Nine PBC patients unresponsive to ursodiol with Alk Phos levels 2x the upper limit of normal were randomized into a crossover study with daily TDF/FTC 300/200 mg and LPRr 800/200 mg versus placebo for 6 months—followed by an open label study for a total of 24 months. Enrollment was limited, as the majority of patients could not tolerate LPRr. Ultimately only 3 patients remained on TDF/FTC and LPRr for 24 months, whereas 6 remained on TDF/FTC alone and 19 clinic patients were used as a historic controls. PBC 40 was used to assess symptoms.

*Results*. Patients on TDF/FTC alone demonstrated comparable Alk Phos levels to a historical control group fulfilling study inclusion criteria. Whereas patients on TDF/FTC and LPRr experienced a significant and durable reduction in (A) Alk Phos and (B) ALT. All patients on antiviral therapy experienced improvement in the mean PBC-40 scores, where as those on TDF/FTC and LPRr had marked improvement in itch score versus those on TDF/FTC alone (*p* = 0.06). One of the three PBC patients normalized all hepatic biochemistry on TDF/FTC and LPRr.

*Conclusions*. Although TDF/FTC and LPRr are not powerful antivirals for the human betaretrovirus, these studies are the first to show a clinically meaningful improvement in hepatic biochemistry as well as improvement in symptoms. It was encoruaging to observe normalization of all hepatic biochemistries in one of three patients maintained on TDF/FTC and LPRr. However, the frequency of side effects from LPRr is more than double reported for HIV. Accordingly, better tolerated combinations of antiretroviral therapy will be required for future studies.

*Funding Agencies: *CIHR

# Sub article: A202: Late-Onset Wilson Disease: A Diagnostic Dilemma Reported

**Authors:** R. Mitchell, H. Ko

**Affiliations:** ^202.1^University of British ColumbiaVancouverBCCanada

*Aims*. This abstract presents a case of late-onset Wilson disease in a patient with biopsy showing non-alcoholic fatty liver disease (NAFLD).

*Methods*. This case report is based on retrospective chart review.

*Results*. This report presents a case of an obese 53 year-old Chinese woman with a history of ulcerative colitis, who was found to have transaminitis during routine blood work. Work up for abnormal liver enzymes revealed negative hepatitis B antigen, undetectable HBV DNA, negative immune markers, but low serum ceruloplasm (<0.08 g/L). She was also found to have elevated cholesterol and triglyceride levels. Abdominal ultrasound showed moderate fatty infiltration of the liver. 24-hour urine copper excretion was done and was found to be elevated (1.69 *μ*mol/d).

Given her high urine copper excretion, she was further worked up for Wilson disease. Slit-lamp examination did not show any evidence of Kayser-Fleischer rings. She then underwent a liver biopsy that showed features consistent with moderate steatohepatits suspicious for, but not diagnostic of Wilson disease. Hepatic parenchymal quantification of copper demonstrated a tissue copper dry weight of 4.42 *μ*mol/g, consistent with the diagnosis of Wilson disease. The patient was given a diagnosis of Wilson Disease and non-alcoholic fatty liver disease (NALFD).

Subsequent MRI revealed no central nervous system involvement of Wilson disease. The patient was started on penicillamine at 750 mg and further counseled on weight loss strategies.

*Conclusions*. Wilson disease is classically described as a disease of children and young adults. There have been several case reports, however, describing the disease in older adults. The diagnostic features and genetic background of patients with late onset Wilson disease are not different than those with early onset. This case presents a diagnostic dilemma and approach to the diagnosis of Wilson disease in an older patient with a history, physical exam and other findings consistent with NAFLD. The most frequently observed histological abnormality in patients with Wilson disease is steatohepatitis, which is also seen in NAFLD.

As the prevalence and detection of NAFLD increases, so too does the clinical suspicion. This case highlights the importance of considering the diagnosis of late-onset Wilson disease in this population, and presents an approach to diagnosis and treatment in such a patient.

*Funding Agencies: *None

# Sub article: A203: Current Practices in Screening for Complications of Primary Sclerosing Cholangitis: A Single-Centre Retrospective Analysis

**Authors:** N. Tabarsi, S. Ip, R. Mitchell, B. Bressler, R. Enns, H. Ko

**Affiliations:** ^203.1^University of British ColumbiaVancouverBCCanada; ^203.2^St. Paul's HospitalVancouverBCCanada

*Background*. Patients with primary sclerosing cholangitis (PSC) are known to be at increased risk for numerous complications including cholangiocarcinoma, colorectal cancer, gallbladder cancer, and metabolic bone disease. Recent guidelines for screening have been published to encourage early identification of these conditions.

*Aims*. To describe the rates of adherence to current guidelines on screening for complications in patients with PSC.

*Methods*. A retrospective chart review was conducted of patients with confirmed PSC under the care of gastroenterologists at an academic tertiary care center in Vancouver between January 2010 and July 2015. Data collected and analyzed included demographics, medical history, biochemical and imaging results, and operative records.

*Results*. Seventy nine patients with PSC were identified. The mean age at diagnosis was 38 ± 19 years old, and 56% of patients were asymptomatic at presentation. Diagnosis of PSC was confirmed by ERCP in 46% of patients, MRCP in 34%, and biopsy in 20%. 74% had a concomitant diagnosis of inflammatory bowel disease (of which 74% had ulcerative colitis, 24% Crohn's disease, and 2% indeterminate colitis). Among patients with PSC and IBD, 49% had colonoscopy for colorectal cancer surveillance every one to two years. 68% of PSC patients without IBD underwent screening colonoscopy. Only 14% of PSC patients had a bone mineral density test. After exclusion of patients with prior cholecystectomy or liver transplantation, 9% had an annual ultrasound for gallbladder cancer surveillance.

*Conclusions*. Despite recent guidelines advocating close surveillance of PSC patients for complications of their disease, our results suggest that even in an academic tertiary care center there is considerable room for improvement in practice. Strategies to enhance guideline adherence are required.

*Funding Agencies: *None

# Sub article: A204: Ultrasound (US) Graded Hepatosteatosis in Non Alcoholic Fatty Liver Disease (NAFLD) Assessed through Contrast Enhanced Ultrasound (CEUS) and Fibroscan versus Normal Controls and Chronic Hepatitis C (CHC) without Cirrhosis and Steatosis

**Authors:** S. Cocciolillo, R. Tavani, A. Sepe, A. Ciccaglione, L. Grossi, L. Marzio

**Affiliations:** ^204.1^Gabriele d'Annunzio UniversityPescara*-*ChietiPescaraItaly

*Background*. NAFLD and Hepatitis C are frequent causes of chronic liver disease in the worldwide.

*Aims*. To assess Liver Stiffness and Intra-parenchymal Blood Flow through Fibroscan and CEUS in NAFLD compared to Controls and CHC without cirrhosis and steatosis. NAFLD patients were divided following the US classification of hepatic steatosis as previously published.

*Methods*. 33 NAFLD patients, 16 patients with CHC without cirrhosis and steatosis and 17 healthy controls were enrolled. NAFLD patients were stratified by US imaging in mild or grade 1 (11 patients), moderate or grade 2 (16 patients) and severe or grade 3 (6 patients). Through a longitudinal intercostal scan right liver parenchyma (LP) was identified, then SonoVue was injected i.v. and digital recording was started for 3 min. Next step was to identify an area of interest in the LP in order to assess blood flow through a dedicated software. Evaluated parameters were: Percent Maximal Contrast Activity (Peak%), Time To Peak (TTP sec), Regional Blood Volume (RBV cm^3^) and Regional Blood Flow (RBF cm^3^/sec). 24 to 48 hours later Fibroscan was performed to evaluate Liver Stiffness measured in kiloPascal (kPa).

*Results*. Peak% and RBF were significantly reduced in NAFLD versus controls (*p* < 0.05) and in US grade 2 and 3 versus CHC and US grade 1 (*p* < 0.05). TTP was significantly longer (*p* < 0.05) in US grade 2 only and RBV was significantly reduced (*p* < 0.05) in US grade 3 versus controls, CHC and US grade 1 and 2. US grades of steatosis had a negative linear correlation with CEUS parameters (Peak%  *r* = −0.574, *p* < 0.01; TTP *r* = −0.349, *p* < 0.05, RBV LP: *r* = −0.503, *p* < 0.01, RBF LP: *r* = −0.557, *p* < 0.01) and a positive linear correlation with Liver Stiffness (*r* = 0.376, *p* < 0.05). Liver Stiffness was significantly increased in all US grades of steatosis and CHC versus controls and CHC versus US grade 1 and 2 (Table 30).

*Conclusions*. A reduction of Peak% and blood flow in the liver parenchyma is found in liver steatosis in comparison to controls and CHC and is negatively correlated with the US grades of steatosis. Liver stiffness increases also progressively with the US grades of steatosis and is higher in CHC and US grade 3. These data suggest that steatosis is a progressive liver disease and changes of intraparenchymal blood flow could be present and detectable before the onset of fibrosis.

*Funding Agencies: *None

# Sub article: A205: Association between Elevated Serum IGG4 Concentrations and the Phenotype of Patients with Primary Sclerosing Cholangitis (PSC)

**Authors:** B. Eksteen, A. Montano-Loza, M. Khan, K. Patel, M. Shiffman, C. Levy, K. Kowdley, R. Myers, H. Janssen

**Affiliations:** ^205.1^University of CalgaryCalgaryABCanada; ^205.2^University of AlbertaEdmontonABCanada; ^205.3^Gilead Sciences CanadaIncMississaugaONCanada; ^205.4^University of TorontoTorontoONCanada; ^205.5^Liver Institute of VirginiaRichmondVAUSA; ^205.6^University of MiamiMiamiFLUSA; ^205.7^Swedish Medical CenterSeattleWAUSA; ^205.8^GileadFoster CityCAUSA; ^205.9^Toronto Western HospitalTorontoONCanada

*Background*. Elevated sIgG4 has been reported in 9–15% of PSC patients and is associated with a more aggressive disease course.

*Aims*. Our aim was to compare the characteristics of PSC patients with elevated and normal sIgG4.

*Methods*. We measured sIgG4 (BN II System; Siemens, Malvern, PA) in PSC patients enrolled in a phase 2b trial of simtuzumab. Corticosteroid and/or anti-TNF-*α* therapies were prohibited. The associations between elevated sIgG4 (>140 mg/dL) with demographics, body mass index (BMI), ulcerative colitis (UC), use of ursodeoxycholic acid (UDCA), liver biochemistry, FibroTest, ELF, sLOXL2 (VIDAS® LOXL2; bioMérieux, Marcy L'Etoile, France), liver fibrosis staged by the Ishak classification, and Mayo risk score (MRS) were determined. MRCP data will be available at the time of presentation.

*Results*. Among 234 patients, 34 (14.5%) had elevated sIgG4. These patients were older than those with normal sIgG4, but sex, race, UC, and use of UDCA did not differ between groups (Table 31 [Table tab32] [Table tab33]). Although liver biochemistry did not differ, patients with elevated sIgG4 had lower serum albumin and higher platelet levels compared with those with normal sIgG4. FibroTest, ELF and sLOXL2, the proportion of patients with bridging fibrosis or cirrhosis, and MELD and MRS were similar between groups. A sensitivity analysis examining a sIgG4 cut-off of >201 mg/dL revealed similar findings.

*Conclusions*. A small proportion of PSC patients have elevated sIgG4. In this clinical trial cohort, the sIgG4 level does not have a significant impact on PSC phenotype including disease severity assessed biochemically, histologically, or according to conventional prognostic indices.

*Funding Agencies: *Gilead Sciences, Inc.

# Sub article: A206: The Role of PGC-1**α** In NAFLD-Associated Liver Cancer

**Authors:** M. Léveillé, A. Besse-Patin, J. Estall

**Affiliations:** ^206.1^Université de MontréalMontréalQCCanada

*Background*. Nonalcoholic fatty liver disease (NAFLD) is the most common cause of abnormal liver biochemistry and affects approximately one third of the population living in North America. It is now estimated that 10–22% of hepatocellular carcinoma (HCC) cases are attributed to fatty liver disease. Although fatty liver and obesity have a large impact on HCC risk, the mechanisms underlying the relationship between diet-related hepatic metabolic disease and cancer development are poorly understood. PGC-1*α* is a transcriptional coactivator that regulates mitochondrial biogenesis and function. It plays a central role in the regulation of cellular energy metabolism and participates in the regulation of both carbohydrate and lipid metabolism. Interestingly, low levels of PGC-1*α* are reported in patients diagnosed with inflammatory fatty liver disease and human liver tumours. However, mechanistic studies in cells and mouse models are few and have yet to clearly define a causative or facilitating role for PGC-1*α* in cancer development and progression.

*Aims*. We aim to determine whether low hepatic PGC-1*α*, when combined with a western diet, alters the DNA damage response and/or metabolic pathways associated with cancer progression to promote the development of NAFLD-associated liver cancer.

*Methods*. We reduced PGC-1*α* specifically in mouse liver using a cre/lox approach. We first analyzed gene expression of pathways commonly associated with cancer (qPCR) in primary hepatocytes isolated from mice fed a chow or high-fat/high-fructose diet (HFHF), with either reduced (50%, LH—liver heterozygotes) or complete loss of hepatic PGC-1*α* (LKO, liver knockouts). We also treated primary hepatocytes with the direct-acting alkylating agent ethylnitrosourea (ENU). DNA damage was detected by immunoblotting of the phospho-histone H2A variant (H2AX). We performed complementary gain-of-function studies in primary hepatocytes following over-expression of PGC-1*α* using adenovirus.

*Results*. Studies in primary hepatocytes suggested that PGC-1*α* influenced genes programs that control S-adenosylmethionine (SAMe) metabolism, cell cycle progression, and fibrosis in a dose-dependent manner. The effects were significantly potentiated when combined with a high-fat/high-fructose diet. Moreover,* in vitro* exposure to ENU suggested that PGC-1*α* expression levels influenced the DNA damage response in primary hepatocytes.

*Conclusions*. In conclusion, changes in PGC-1*α* expression appear to regulate gene pathways involved in HCC development and this is influenced by a western diet. As a next step, we aim to determine whether altered levels of PGC-1*α* influence liver tumour development* in vivo*. This data implicates PGC-1*α* as an important mitigating factor in the development of obesity- and NASH-associated liver cancer.

*Funding Agencies: *Canadian Liver Foundation

# Sub article: A207: Hemochromatosis: High Liver Iron No Cirrhosis, No Iron with Major Symptoms?

**Authors:** P. Adams

**Affiliations:** ^207.1^University HospitalLondonONCanada

*Background*. Hemochromatosis has been defined historically as an inherited condition in which excess iron accumulates progressively which may lead to liver cirrhosis, and arthrritis. Since the* HFE* gene was discovered in 1996, typical C282Y homozygotes have been discovered without iron overload and patients have been identified with severe hepatic iron overload without liver damage.

*Aims*. To determine the prevalence of non-cirrhosis amongst C282Y homozygotes with a liver iron concentration > 280 *μ*mol/g. The clinical database was reviewed to find major morbidity in non-expressing C282Y homozygotes.

*Methods*. C282Y homozygotes with liver biopsy data were reviewed to study the relationship of liver iron concentration to liver damage. No patients in this group had alcoholism or chronic viral hepatitis. C282Y homozygotes with no iron overload as determined by a normal serum ferritin and transferrin saturation were reviewed for major morbidity that may be releated to genetic hemochromatosis rather than iron overload. A potentially toxic liver iron concentration was considered to be > 280 *μ*mol/g, normal 0–35. (Adams PC, Am J Gastro 2001; 567). Multiple regression models were used to study the relationships of liver iron, ferritin, age, and male gender to the development of cirrhosis in C282Y homozygotes.

*Results*. There were 42 C282Y homozygotes with a liver iron concentration > 280 *μ*mol/g. There were 19 patients in this group without cirrhosis. Five of these patients were reported to have liver fibrosis but no fibrosis was seen in 14 patients. An 89 year old woman had a liver iron concentration of 329 *μ*mol/g, no fibrosis and became a liver donor for a succesfful liver transplant. Mean liver iron concentration in cirrhotic C282Y homozygotes was higher (496 *μ*mol/g range 285–814) compared to non-cirrhotics (393 *μ*mol/g, 282–632, *p* = .007). Multiple regression (*n* = 120) showed that liver iron concentration (*p* = .0005), serum ferritin (*p* = .0002) and male gender (*p* = .04) were associated with the development of cirrhosis.

An 18 year male C282Y homozygous man presented with typical hemochromatosis arthropathy in his knees despite a normal ferritin and transferrin saturation and had both knees replaced at age 36. A 55 year old female C282Y homozygote with a normal serum ferritin and transferrin saturation underwent an elective liver translant for ciirrhosis. The explant showed no iron overload and no obvious etiology for the cirrhosis.

*Conclusions*. The clinical consequences of being a C282Y homozygote for the* HFE* gene are unpredictable. An elevated liver iron does not always lead to cirrhosis. Two rare cases of cirrhosis and joint replacement are reported in the absence of iron overload. The role of co-modifying genes in the clinical expression of hemochromatosis is being studied using exome sequencing.

*Funding Agencies: *None

# Sub article: A208: Sequential Algorithm of Non-Invasive Tools to Assess Severe Fibrosis in Non Alcoholic Fatty Liver Disease

**Authors:** A. Shaheen, P. Rye, S. Urbanski, M. Swain, S. Jayakumar

**Affiliations:** ^208.1^University of CalgaryCalgaryABCanada; ^208.2^Univ CalgaryCalgaryABCanada

*Background*. The prevalence of Non-Alcoholic Fatty Liver Disease (NAFLD) is markedly increasing. Multiple non-invasive tools exist to assess fibrosis in NAFLD.

*Aims*. To assess the performance of several clinical tools and to develop a stepwise algorithm to identify severe fibrosis in NAFLD patients.

*Methods*. Forty-four consecutive patients with NAFLD who underwent liver biopsy were included in our pilot cohort at the University of Calgary. Fibrosis assessment tools including: Transient elastography (Fibroscan; TE, Ecosens, France), AST-to-Platelet ratio (APRI), Fib-4 score (Age, ALT, AST and platelet count), NAFLD fibrosis score index (including Age, BMI, diabetes, AST, ALT, platelet count and albumin) were used to assess severe fibrosis (METAVIR F3-4) within 3 months after liver biopsy. Performance of these tools was assessed using area under receiver operating curve (AUROC). A sequential algorithm using best screening tools according to accuracy of their positive predictive value (PPV) and negative predictive value (NPV) was developed in this derivation cohort.

*Results*. In our cohort median age was 51.5 years (IQR: 43–60). Fourteen patients (32%) had severe fibrosis (F3-4). Patients with severe fibrosis had higher TE score (16.2 versus 9.7 kPa, *P* = 0.02), APRI (0.89 versus 0.37, *P* < 0.001) and Fib-4 (1.99 versus 1.10, *P* < 0.001) versus patients with less fibrosis. There was no difference in NAFLD fibrosis score between these groups (0.10 versus −0.97, *P* = 0.14). Using a TE cutoff of 12.2 kPa we were able to classify correctly 76% of the cohort (AUROC; 0.73: 0.60–0.86), while NAFLD fibrosis score classified 68% correctly (cutoff 0.044; AUROC 0.64: 0.48–0.78). However, by applying APRI at a cutoff of 0.58 accuracy was 84% and AUROC was 0.84 (0.70–0.93), and for FIB-4 at a cutoff of 1.95 accuracy was 80% and AUROC was 0.82 (0.67–0.92). We developed an algorithm based on FIB-4 and APRI performance which demonstrated that f both Fib-4 < 1.95 and APRI < 0.58 the accuracy to rule out severe fibrosis was 92% (*n* = 24/26). Also, if Fib-4 > 1.95 and APRI > 0.58 the accuracy to rule in severe fibrosis was 80% (*n* = 8/10). For discordant results (*n* = 8), TE = 12 kPa classified indeterminate results with 88% accuracy (*n* = 7/8).

*Conclusions*. We propose an algorithm of using FIB-4 and APRI to screen for severe fibrosis in NAFLD patients (accuracy 92% to rule out severe fibrosis, and 80% to rule in severe fibrosis). We propose that APRI and FIB-4 can be used as screening tools for severe fibrosis in NAFLD patients by general practitioners. TE is an excellent modality to assess indeterminate NAFLD cases.

*Funding Agencies: *CIHR

# Sub article: A209: Prebiologic Evaluation: Follow-Up Survey of Practice Patterns in Nova Scotia

**Authors:** J. Kiberd, M. Stewart, S. Gruchy

**Affiliations:** ^209.1^Dalhousie UniversityHalifaxNSCanada; ^209.2^University of CalgaryCalgaryABCanada; ^209.3^Dalhousie UnivHalifaxNSCanada

*Background*. There has been a significant increase in the use of biologic therapy across Canada. Given the risks associated with this therapy, appropriate pre-treatment evaluation is important.

*Aims*. This study reports the impact a pre-biologic therapy initiation checklist that was distributed across Nova Scotia with the goal of enhancing and streamlining care for patients receiving TNF-alpha inhibitor therapy. Changes in practice were examined using pre- and post-checklist distribution surveys.

*Methods*. A pre-checklist survey was carried out in July 2012. The checklist was then distributed in February 2013 with a post-checklist survey conducted in July 2014. A total 63 specialists were provided with the checklist and invited to participate in the surveys.

*Results*. Of the 63 surveys distributed, 35 (55.5%) were returned in both the pre- and post-surveys. The number of responding specialists was different with dermatology (8 versus 6), rheumatology (9 versus 5), and gastroenterology (18 versus 24). In the post-survey, the percentage reporting evaluation of risk activity/symptoms was high (HIV (88%), chronic infection (97%), herpes simplex (62%), perianal sepsis (69%) and cervical dysplasia in women (52%)). The reported testing was also high (CBC (100%), LFT (97%), TB Skin Test (100%), CXR (89%), creatinine (89%), CRP (94%), and HBV (94%)). Less frequent testing included ESR (63%), HIV (65%), varicella antibodies (64%), and urinalysis (21%). Vaccinations that were considered important included Seasonal Influenza (73%), Pneumococcal (71%), HBV (61%), Rubella (47%), Tetanus (41%), and HPV (30%). Specialists were less likely to provide vaccinations (37%) and most referred the patients to their primary care physician (97%). Routine warnings to patients were universal for infection (100%), but less for allergic/infusion reactions (71%) and cancer (63%).

Figure 25 [Fig fig26] shows that several practices increased between the pre and post survey. These included HIV serology (*p* = 0.017), Hepatitis B serology (*p* = 0.021), arranging vaccinations (*p* = 0.044), and referral to family physician for vaccinations (*p* = 0.033). Varicella zoster serology was found to have decreased (*p* = 0.016). Many of the other activities increased but not significantly.

*Conclusions*. The “Biologic Checklist” appears to increase the pre-treatment evaluation and vaccination rate in patients receiving biologic therapy.

*Funding Agencies: *None

# Sub article: A210: Analysis of Safety and Efficacy of Sofosbuvir-Based Therapy in Liver Transplant Assessed Hepatitis C Patients

**Authors:** B. Thomas, B. Aljudaibi, P. Marotta, K. Qumosani, P. Adams, M. Levstik

**Affiliations:** ^210.1^Western UniversityLondonONCanada; ^210.2^London Health Science CentreLondonONCanada; ^210.3^London heatlh sciences CenterUniversity of Western OntarioLondonONCanada; ^210.4^University HospitalLondonONCanada; ^210.5^London Health Sciences CentreLondonONCanada

*Background*. Hepatitis C (HCV) infection remains the most common indication for liver transplant despite our current novel therapies with substantial cure rates. HCV Infection management in prospective and post-liver transplant patients has been evolving over the past decade with the adoption of newer treatment strategies given the tolerability these agents. Recent studies have evaluated the use of IFN-free therapies in compensated and decompensated cirrhosis has shown promise with maintaining undetectable viral loads post transplant. HCV Patients treated with Sofosbuvir-based therapy have seen hepatic recovery albeit the degree and specific patient population in which this occurs is undetermined. The safety and efficacy of Sofosbuvir-based therapy in the transplant eligible liver disease population currently is unclear.

*Aims*. To assess the safety and efficacy of Sofosbuvir-based therapy in patients with HCV infection undergoing transplant assessment.

*Methods*. Analysis of prospectively collected data of a cohort HCV patients who have undergone liver transplant assessment at London Health Sciences Centre from January 2014 to December 2014. Patients who had commenced Sofosbuvir-based therapy were selected. Patient outcomes included sustained virologic response (SVR), MELD-Na score, Child-Pugh score and liver transplant status were analyzed.

*Results*. Interim analysis was performed on 44 patients. A total of 7 patients (16%), all genotype 1, had commenced Sofosbuvir-based therapy with 5 patients completing therapy acheiving SVR. The mean MELD-Na score of these patients was 20.4 and mean Child-Pugh score was 9.3. 3/7 patients on therapy died, 1 from small bowel ischemia after completing therapy and 2 deaths prior to completion of therapy, both patients died from sepsis. 1 patient who acheived SVR was removed from the transplant list because of substantial clinical improvement, Child-Pugh B pre-treatment and Child-Pugh A post-treatment. 1 patient remained on the transplant list after acheiving SVR. In total, 18 patients underwent orthotopic liver transplantation, of these 2 patients completed treatment and acheived SVR prior to transplantation. 8 patients were pending approval for Sofosbuvir-based therapy with 1 death awaiting approval. None of the patients discontinued therapy.

*Conclusions*. In this preliminary analysis, 25% of the HCV patients who achieved SVR were taking off the transplant list because of substantial clinical improvement. However, a larger sample size with be presented at Canadian Digestive Disease Week.

*Funding Agencies: *None

# Sub article: A211: Retrospective Analysis of an Ontario Based Diagnostic Referral Program Using Transient Elastography

**Authors:** K. Boctor, M. Magnes, M. Elkhashab

**Affiliations:** ^211.1^Toronto Liver CentreTorontoONCanada

*Background*. Canadian consensus guidelines for hepatitis B and C management have incorporated transient elastorgraphy as a validated, non-invasive means of stratifying liver disease (1). In 2010, The Toronto liver Centre created the first FibroScan® referral program in Ontario, and has since been serving the community.

*Aims*. The purpose of this analysis was to understand how Ontario physicians currently use fibroscan testing alongside the approved guidelines in the management and follow-up of chronic liver disease.

*Methods*. 5438 patients were referred to The Toronto Liver Centre from June 17, 2010 to October 28, 2014 for fibroscan testing & stratification of liver fibrosis/steatosis. Referrals were assessed for: referring physician specialty & ethnicity, catchment area, patient demographics, disease etiology, fibrosis stage at referral & time between serial fibroscans.

*Results*

*Physicians*. A total of 262 physicians referred patients for fibroscans; 63% were General Practitioners, 29% Gasteroenterologist/Hepatologists, 3% Infectious Disease, & 5% others. Of the 262 physicians, 67% were Chinese, 13% South Asian & 20% Unknown.

The majority of patients referred for a fibroscan were diagnosed with HBV or HCV (3399, 866) respectively. 1,186 (21.8%) had other liver conditions; fatty liver being more prevalent. Catchment area extended to 25 cities within Ontario & 1 city outside of Canada.

*Patient Demographics*. Results from fibroscan tests were reported for 5,437 patients, 58% men versus 42% women. Median age was 53 years; women referred being older with a median age of 54 years versus 52 years for men.

*Fibrosis Stage*. Approximately 67.8% of patients had a fibrosis score of F0 or F1. A fibrosis score of F3 was reported for 6.4%, while a fibrosis score of F4 or cirrhosis was reported for 10.3% of patients.

*Serial Fibroscans*. Preliminary analysis of the available data for serial fibroscans shows no additional benefit in routine measurements of liver stiffness in a period less than 12 to 14 months.

*Conclusions*. Referrals from general practioners comprised 63% of our database; where 16.7% of patients had liver fibrosis of F2 to F4, thereby qualifying those patients for treatment reimbursement. Furthermore, considering the large catchment area for this analysis despite the lack of OHIP coverage, proves that fibroscan testing is widely accepted by patients and physicians within the community. Moreover, seeing that only 4.8% of patients underwent serial fibroscans, there's a need for regulation of the frequency by which patients should be referred for fibroscan testing. We plan to assess this factor in the near future.

*Acknowledgments*. Jean Palmer-statistical analysis.

*Funding Agencies: *Gilead Sciences Canada for financial support of statistical analysis, None

# Sub article: A212: Retrospective Analysis of Chronic HBV Patients Referred to TLC; An Ontario Based FibroScan® Referralprogram

**Authors:** K. Boctor, M. Elkhashab, M. Magnes

**Affiliations:** ^212.1^Toronto Liver CentreTorontoONCanada

*Background*. In 2001, statistics Canada estimated the number of HBV-infected patients to be 600,000 (1), that number has increased to approximately 715,000 based on the 2% general population HBV carrier status (2,3). With the availability of novel diagnostic testing and the long term use of oral nucleos/tide analogue antiviral therapies, it has become necessary to develop a systematic method to monitor patients over longer periods of time that is both cost-effective, and poses no risk to the patients. Therefore, Canadian consensus guidelines for the management of hepatitis B have incorporated transient elastorgraphy as a validated, non-invasive means of stratifying liver disease (4).

*Aims*. The aim of this analysis is to assess the current use of FibroScan technology by physicians in Ontario, and its role in long-term management in chronic HBV.

*Methods*. 3399 patients were referred to The Toronto Liver Centre from June 17, 2010 to October 28, 2014 for fibroscan testing and stratification of liver fibrosis. Referrals were assessed for: patient demographics, relevant blood work (LFTs, platelets, HBV DNA) available upon examination, and stage at which patients were referred for testing.

*Results*. The median age was 53 years. Women were older, median 54 years versus 52 years for men. 90.3% of patients had a liver fibrosis staging of ≤F2, with a mean liver stiffness and SD of 5.65 kPa,7.34, respectively.

Of the 3399 patients referred, 78% had results for ALT, 77% for platelets, 12% for HBV DNA; none had HBeAg/Ab results available at time of testing. Only 0.03% (111) of patients referred with ≤F2 were recommended for therapy, based solely on family history, blood work, or viral load. Patients who had fibrosis >F2 and were recommended for treatment, had a median platelet count of 101, *P* = 0.0005.

It was observed that an increasing fibrosis score (>F2) paralleled an increase in ALT value, and a decrease in platelet count.

*Conclusions*. Current CASL guidelines for the management of hepatitis B suggest HBV treatment to commence at ≥F2; with the understanding that each patient requires assessment on individual basis. FibroScan® testing provides clinicians in Ontario with an excellent screening tool for liver staging, as well as a non-invasive means by which patients can be monitored over timely intervention and long-term management.

*Acknowledgments*. Jean Palmer for statistical analysis.

*Funding Agencies: *Gilead Sciences Canada, Inc. for financial support of statistical analysis

# Sub article: A213: Choledocholithiasis in Infancy

**Authors:** K. Prowse, J. Dowhaniuk, M. Hussein, M. Sherlock

**Affiliations:** ^213.1^McMaster UniversityHamiltonONCanada

*Background*. Symptomatic choledocholithiasis in infancy is uncommon^1^. While the underlying pathophysiology is unclear, increased incidence is observed in infants with prematurity, infection, dehydration, parenteral nutrition, furosemide and gastrointestinal dysfunction^2,3^. A small number of case reports describe favourable outcome with conservative management ^1^.

*Aims*. Assess conservative management of choledocholithiasis.

*Methods*. We describe two cases of stone resolution using a combination of ursodeoxycholic acid and antibiotics. These cases suggest the potential application of this safe, noninvasive therapy as initial management in infants with choledocholithiasis^1^.

*Results*

*Case  1*. 2 month old healthy term baby presented with scleral icterus, conjugated jaundice and acholic stools. Abdominal ultrasound (US) revealed dilation of the common bile duct (CBD) and intrahepatic bile ducts with an echogenic shadowing focus in the distal CBD measuring 5 × 4 × 3 mm, consistent with a stone. The patient was started on intravenous (IV) Ampicillin, Gentamicin and Metronidazole for 10 days along with oral ursodeoxycholic acid. 5 days into treatment, a liver biopsy was performed revealing cirrhosis with severe diffuse cholestasis, severe hepatocellular degeneration, ductal proliferation and portal fibrosis, favouring an obstructive etiology. Intraoperative cholangiogram confirmed a dilated CBD however no visible stone was observed. A repeat US performed 14 days later reported a normal CBD and resolved choledocolithiasis. Liver enzyme elevation, acholic stools and jaundice resolved.

*Case  2*. 4 month old healthy term baby presented with jaundice and acholic stools. Abdominal US revealed a dilated CBD and intrahepatic bile ducts with a 4 mm stone in the distal CBD. The patient was treated with IV Ampicillin, Gentamicin, Metronidazole and oral ursodeoxycholic acid. Serial abdominal US were performed which demonstrated resolution of choledolithiasis after 10 days of treatment. Acholic stools and hyperbilirubinemia resolved.

Investigations including metabolic, viral, thyroid and hemolysis work up were completed for both patients and revealed no abnormalities.

*Conclusions*. This case series highlights the potential benefit of a non-invasive approach to the management of choledocholithiasis, which may lead to resolution in both clinical symptoms and radiologic evidence of obstruction, avoiding the need for an invasive procedure. The postulated mechanism of action is a reduction in inflammation and edema associated with cholangitis, following antibiotic treatment^1^. The use of ursodeoxycholic acid may help facilitate the passage of the stone by stimulating bile flow.^1^

*Funding Agencies: *None

# Sub article: A214: Public Drug Coverage Is Associated with Delayed Access to Anti-Tumor Necrosis Factor Therapy for Patients with Inflammatory Bowel Disease in a Universal Healthcare System

**Authors:** A. Rumman, R. Candia, J. Sam, R. Thanabalan, K. Croitoru, G. Greenberg, M. Silverberg, H. Steinhart, G. Nguyen

**Affiliations:** ^214.1^Mount Sinai Hospital Centre for Inflammatory Bowel DiseaseTorontoONCanada

*Background*. Anti-TNF therapy is effective for the treatment of inflammatory bowel disease (IBD). Disparities in access are suspected in patients with public insurance coverage compared to patients with supplemental private insurance.

*Aims*. The main objective of this study was to evaluate if the type of insurance payer is a predictor of the time between prescription and administration of anti-TNF therapy in IBD patients in Ontario.

*Methods*. We conducted a retrospective cohort study of IBD patients who were prescribed anti-TNF therapy between January 2007 and June 2014. The time interval from prescription to first administration of anti-TNF therapy and rates of hospitalizations and ER visits were compared between those with private and public insurance.

*Results*. There were 268 patients with IBD who were prescribed anti-TNF therapy. The median time interval from prescription to administration of anti-TNF therapy was 11 days longer for those publically insured versus those privately insured (34 versus 23 days, *p* = 0.036). After excluding patients who first received anti-TNF therapy in-hospital or through compassionate use programs, this disparity increased to 22 days (57 versus 35 days, *p* = 0.036). Compared to privately insured patients, patients with public coverage were more likely to be steroid-dependent or refractory (56.6% versus 71.4%, *p* = 0.05, resp.) and report previous use of immunomodulators (53.4% versus 71.4%, *p* = 0.007, resp.).

In multivariable analysis, publicly insured patients were less likely to receive timely access to anti-TNF therapy compared to those with private coverage (adjusted hazard ratio, 0.61; 95% CI: 0.42–0.88). Following the decision to start anti-TNF therapy, publicly-funded patients had significantly more IBD-related hospital admissions (14.8 versus 4.84 admissions per 1000 person-months, *p* < 0.001) and ER visits (34.1 versus 9.75 ER visits per 1000 person-months, *p* < 0.001) compared with patients with private drug coverage.

*Conclusions*. IBD patients in Ontario experienced greater delays in access to anti-TNF therapy if they required public coverage compared with private insurance. Furthermore, patients with public coverage required more hospitalization and had higher ER utilization rates. Further studies are needed to assess the economic impact of barriers in access biologic therapy.

*Funding Agencies: *The study was funded by a peer-reviewed grant sponsored by the Future Leaders in IBD program (FLIBD)

# Sub article: A215: Disparities in Care, Surgery, and Diagnostic Delay in Canadian Immigrants with Inflammatory Bowel Disease

**Authors:** E. Benchimol, D. Manuel, T. To, N. Mojaverian, G. Nguyen, A. Guttmann

**Affiliations:** ^215.1^University of OttawaOttawaONCanada; ^215.2^University of TorontoTorontoONCanada

*Background*. Canada has amongst the highest rates of IBD in the world, and the highest proportion of immigrants amongst G8 nations. Disparities in health services use and outcomes in immigrants have not been assessed.

*Aims*. Determine disparities in pre-diagnosis delay, specialist care, health services use, and surgical outcomes in immigrants with IBD compared to non-immigrants.

*Methods*. The Ontario Crohn's and Colitis Cohort (OCCC) was used to identify all incident cases of IBD in children (1994–2009) and adults (1999–2009) with validated algorithms. Linked immigration data identified those who arrived to Ontario after 1985. Countries of birth were grouped into regions based on World Bank classification. We linked to health administrative data, and compared diagnostic delay (time from first code for IBD-related sign/symptom to first contact with an IBD-specific diagnostic code), post-diagnosis health services use, and physician specialist care in immigrants and non-immigrants. We used Poisson, Cox proportionate hazard or logistic regression models controlling for sex, age at diagnosis, income and rural/urban. Rates of IBD-specific visits (using ICD codes for CD and UC) were determined, as were IBD-related visits (CD/UC codes or signs/symptoms of IBD).

*Results*. IBD was diagnosed in 1552 immigrants, and 29,126 non-immigrants. Immigrants had similar diagnostic delay as non-immigrants (CD: HR 0.998, 95% CI 0.89–1.12, UC: HR 0.935, 95% CI 0.82–1.06). Immigrants had greater IBD-specific outpatient health services use after diagnosis (OR 1.2, 95% CI 1.2–1.3), ED visits (OR 1.6, 95% CI 1.3–1.9), and hospitalizations (OR 1.2, 95% CI 1.02–1.4). There was no significant difference in IBD-related health services use. Risk of surgery in immigrants was lower for CD (HR 0.66, 95% CI 0.43–0.995), driven by a lower risk in those from the Middle East (HR 0.40, 95% CI 0.18–0.88). Risk of colectomy in UC was also lower (HR 0.52, 95% CI 0.31–0.87), driven by those from East Asia (HR 0.39, 95% CI 0.15–0.996) and the Middle East (HR 0.32, 95% CI 0.13–0.78). Immigrants were more likely to see a gastroenterologist for IBD-specific (OR 1.37, 95% CI 1.34–1.40) and IBD-related (OR 1.20, 95% CI 1.18–1.22) reasons, and had a greater proportion of outpatient visits to a gastroenterologist.

*Conclusions*. While immigrants had greater health services use for IBD-specific reasons, they had lower risk of surgery. There was no significant difference in diagnostic delay between immigrants and non-immigrants, but immigrants with IBD were seen by gastroenterologists more often than non-immigrants. Future research will investigate differences in biological, clinical, and social factors to explain these differences.

*Funding Agencies: *Ontario Ministry of Health and Long-Term Care Academic Health Sciences Centres AFP Innovation Fund

# Sub article: A216: Identification of Biopsy-Proven Pediatric Celiac Disease from Ontario Health Administrative Data: A Cautionary Example of the Importance of Validation

**Authors:** J. Chan, D. Mack, S. Hawken, D. Manuel, J. de Nanassy, E. Benchimol

**Affiliations:** ^216.1^School of EpidemiologyPublic Health and Preventive MedicineUniversity of OttawaOttawaONCanada; ^216.2^Children's Hospital of Eastern OntarioUniversity of OttawaOttawaONCanada; ^216.3^Ottawa Hospital Research InstituteUniversity of OttawaOttawaONCanada

*Background*. Over recent years, Canada has experienced increased awareness of celiac disease (CD) and gluten-sensitive conditions in children, which may have resulted in increased burden on the health system. Population-based health administrative data have been used to conduct epidemiology and health services research in children with chronic diseases, but identification algorithms have not been validated for CD.

*Aims*. Validate an algorithm based on health administrative data diagnostic codes to accurately identify children with biopsy-proven CD.

*Methods*. All cases of biopsy-proven CD diagnosed 2005–2011 in Ottawa were identified through chart review and search of the pathology database from the Children's Hospital of Eastern Ontario (CHEO), and linked to the Ontario health administrative data to serve as positive reference standard. All other children living within Ottawa served as the negative reference standard. Case-identifying algorithms based on outpatient physician visits with associated ICD-9/10 codes for CD plus endoscopy billing codes were constructed and tested. Sensitivity, specificity, PPV and NPV, with 95% confidence intervals (CI), were determined for each algorithm. Poisson regression, adjusting for sex and age at diagnosis, was used to explored the trend in outpatient visits associated with a CD diagnostic code from 1995–2011.

*Results*. Approximately 200 algorithms were tested. The best algorithm to identify CD consisted of an endoscopy billing claim follow by 1 or more adult or pediatric gastroenterologist encounters after the endoscopic procedure. The sensitivity, specificity, PPV, and NPV for the algorithm were: 70.4% (95% CI 62.1–78.8%), >99.9% (95% CI >99.9–100%), 53.3% (95% CI 45.4–61.2%) and >99.9% (95% CI >99.9–100%). The algorithm identified 1289 suspected CD cases from the administrative data. There was a 9% annual increase in the use of this combination of CD-associated diagnostic codes in physician billing data 1995 and 2011 (RR 1.09, 95% CI 1.07–1.10, *P* < 0.001).

*Conclusions*. Ontario health administrative data is not suitable for identifying incident pediatric CD cases with its current structure and variables. The tested algorithms suffer from poor sensitivity and/or poor PPV, which increase the risk of case misclassification that could lead to biased estimation of CD incidence rate.

*Funding Agencies: *CHEO Research Institute

# Sub article: A217: Estimating Incidence and Prevalence of Crohn's Disease and Ulcerative Colitis: Approaches to Address Misclassification in Administrative Health Data

**Authors:** J. Pena-Sanchez, L. Yan, J. Jones, L. Lix

**Affiliations:** ^217.1^University of SaskatchewanSaskatoonSKCanada; ^217.2^University of ManitobaWinnipegMBCanada; ^217.3^Dalhousie UniversityHalifaxSKCanada

*Background*. Administrative health data are widely used to conduct studies about the epidemiology of chronic diseases. However, diagnoses contained in administrative health data are prone to misclassification errors. While a number of different methodological approaches have been taken to address misclassification errors, there is no uniformly recommended method and few comparisons about the impact that these methods will have on population estimates of disease.

*Aims*. To estimate the number of incident and prevalent cases of Crohn's disease (CD) and ulcerative colitis (UC) applying a Monte Carlo (MC) method and a two-source capture-recapture (CR) model to address misclassification error for case definitions applied to administrative health data.

*Methods*. Using hospital and physician data from the province of Saskatchewan, we applied a validated case definition to ascertain incident and prevalent cases of CD and UC for adults from April 1990 to March 2010. Our MC method adjusts the observed number of cases of CD/UC, based on prior estimates of sensitivity and specificity of the case definition. Second, we applied two-source CR models (physician billing and hospital data). Finally, we proposed a two-phase method that estimated incidence/prevalence using CR models; then adjusted these estimates with the MC method to account for the less-than-perfect specificity of the case definition.

*Results*. Annual crude incidence ranged from 13.7–18.6 and 14–12.4 per 100,000 for CD and UC, respectively. The crude prevalence of CD and UC varied from 89.5–160.4 and 62.7–56.5 per 100,000, respectively. For the MC method, the adjusted numbers of incident cases of CD and UC were between 1% and 20% higher than the number of observed cases. The adjusted numbers of prevalent cases of CD and UC were on average 2% and 14%, respectively, less than the observed cases. The CR models estimated more incident cases than the observed (on average, 15% for CD and 21% for UC). Minor increases in the adjusted prevalent cases were observed (on average, 1% for CD and 2% for UC). The estimates of the two-phase approach demonstrated an increasing trend over time of the incident cases of CD, while UC showed a stable trend. The prevalence estimates showed a constant rising tendency for CD, whereas UC presented a slight decrease over time.

*Conclusions*. We estimated incidence/prevalence of CD/UC, correcting for potential misclassification issues and proposing a two-phase approach. In Saskatchewan from 1990–2009, constant increasing trends in the incidence and prevalence of CD were identified, whereas for UC a flat trend in the incidence and a minor decline in the prevalence were observed.

*Funding Agencies: *Saskatchewan Health Research Foundation

# Sub article: A218: Inflammatory Bowel Diseases Patients Are at Lower Risk of Acute Coronary Syndrome

**Authors:** A. Shaheen, C. Ma, R. Panaccione, C. Seow, K. Novak, S. Ghosh, M. Stapleton, G. Kaplan

**Affiliations:** ^218.1^University of CalgaryCalgaryABCanada; ^218.2^University of CalgaryCalgary ABABCanada

*Background*. The association between inflammatory bowel disease (IBD) and acute coronary syndrome (ACS) is controversial. Previous studies report different risk magnitude for developing ACS among IBD patients.

*Aims*. To assess the association between ACS and IBD using a large population-based database.

*Methods*. This study was conducted using the 2008 Nationwide Inpatient Sample (NIS) database. First, we identified all patients admitted with a primary diagnosis with ACS (including unstable angina, non-ST elevation MI and ST elevation MI). We matched them to controls according to age, gender, race, admission type (elective versus non-elective) and US region. In this phase we assessed predictors of ACS including IBD. In the second stage, we identified all patients admitted primarily with IBD diagnosis; ulcerative colitis (UC) or Crohn's disease (CD). We matched IBD patients to controls according to age, gender, race, admission type and region. In the second phase we assessed rates and predictors of developing ACS during hospitalization as a secondary diagnosis. We used weighted regression models to assess the impact of risk factors on developing primary or secondary ACS and adjusted for patient and hospital characteristics.

*Results*. There were 143,831 ACS admissions matched to 143,773 control admissions. ACS patients had higher rates of hypertension (67.5% versus 59.8%), smoking (31.4% versus 19.1%), dyslipidemia (52.3% versus 28.3%), diabetes (32.2% versus 28.6%), and obesity (10.1% versus 7.3%), but not IBD (0.4% versus 0.7%) (*P* value < 0.001 for all comparisons). Traditional ACS risk factors were associated with higher risk of developing ACS. However, history of IBD was associated with lower risk (adjusted OR: 0.63 (95% CI: 0.54–0.74)). In the second phase, 19,650 patients admitted primarily with IBD flare were matched to 19,649 controls. Rates of developing ACS during hospitalization were less common in IBD patients (0.5% versus 1.8%, *P* < 0.001). IBD patients had lower rates of traditional ACS risk factors (hypertension: 24.2% versus 32.2%; dyslipidemia 9.5% versus 13.7%; obesity: 3.7% versus 8.1%; diabetes: 8.4% versus 16.8%; *P* < 0.001). However, smoking rates were similar compared to controls (20.6% versus 19.6%, *P* = 0.49). Patients admitted with IBD flare were less likely to suffer from ACS during hospitalization (0.31 (0.23–0.41)).

*Conclusions*. In this large population-based study, we demonstrate that patients admitted with ACS had lower rates of IBD, and conversely, patients admitted with IBD flare are also less likely to develop secondary ACS. Prospective studies are needed to validate our findings.

*Funding Agencies: *None

# Sub article: A219: End of Life Health Care Costs and Utilization in Inflammatory Bowel Disease: A Population-Based Study

**Authors:** S. Murthy, P. James, P. Tanuseputro

**Affiliations:** ^219.1^Department of MedicineUniversity of OttawaOttawaONCanada; ^219.2^Institute for Clinical Evaluative SciencesOttawaONCanada

*Background*. Inflammatory bowel disease (IBD) is associated with substantial morbidity and health care use. The period preceding death represents a time of heightened health care use that could be curtailed with early implementation of cost-effective approaches to care. However, there are no reports of costs or health care use associated with end-of-life (EOL) IBD care.

*Aims*. To compare direct EOL health care costs and utilization between IBD and non-IBD patients.

*Methods*. A population-based retrospective cohort study was conducted of all decedents within the province of Ontario, Canada, between April 1, 2010 and March 31, 2013. IBD patients were compared to non-IBD patients on direct health care costs in the last year of life and acute care use in the last 90 days of life, controlling for age, sex, income quintile, rurality and co-morbidity burden.

*Results*. Of 264,754 Ontario decedents, 2,214 (0.83%) had IBD. On average, IBD patients spent close to 16 of their last 90 days of life in an acute care setting and an additional 2.1 adjusted hospital days (95% CI 1.5–2.8 days) as compared to non-IBD patients. IBD patients also incurred higher EOL costs than non-IBD patients in most health care sectors, with the major cost differential attributable to acute care costs (Table 34 [Table tab35]). After adjustments, IBD decedents cost the Ontario health care system an additional $7,234 per person (95% confidence interval (CI) $5,005–$9464) during the last year of life as compared to non-IBD decedents. Overall, EOL care in IBD patients costs the Ontario health care system in excess of $5 million CAD annually over and above baseline EOL costs in the general population.

*Conclusions*. IBD diagnosis is associated with substantially higher EOL costs, which is largely attributable to acute hospital care. For many IBD patients, shifting EOL care from acute care settings to increasing supports in the community could lead to a decrease in health care costs while possibly increasing quality of life.

*Funding Agencies: *CIHR

# Sub article: A220: Patients with Cirrhosis Are at an Increased Risk of Having a Positive Fit Test

**Authors:** J. McGrath, J. Leonard, M. Borgaonkar, D. Pace, S. Antle, J. Quinlan, A. Asokan

**Affiliations:** ^220.1^Eastern HealthSt. John'sNFCanada; ^220.2^Health Sciences CentreSt. John'sNFCanada; ^220.3^Memorial UniversitySt. John'sNFCanada; ^220.4^Memorial University of NewfoundlandSt. John'sNFCanada

*Background*. Patients with cirrhosis of the liver are at high risk for a variety of complications including malignancies. There are a few small reports that liver disease may increase the risk of colonic adenomas and colon cancer.

*Aims*. The aim of this study was to evaluate if patients with cirrhosis are more likely to have a positive Fecal Immunohistochemical Test (FIT) test compared to patients without cirrhosis.

*Methods*. This study was conducted through Memorial University's Division of Gastroenterology and Hepatology during 2014 and 2015. Patients aged 50–74 at average risk for colon cancer were identified in hepatology clinic. These patients were enrolled in the provincial colon cancer screening program which uses a FIT. Participants who were FIT positive were further evaluated via colonoscopy. Data regarding patient's FIT status, presence of liver disease, presence of cirrhosis, presence of adenomas, age, gender and other factors were collected.

*Results*. This study enrolled 117 patients, 80 of which completed the FIT test, giving a 68% completion rate. Of the 80 patients who completed FIT testing, 17 were FIT positive. Of the 17 FIT positive patients, 12 had cirrhosis. Patient's identified as having cirrhosis showed a relative risk of 7.200 (2.754–20.115; CI 95%; *p* < 0.001) for having a positive FIT test compared to those patients without cirrhosis.

*Conclusions*. Patients with cirrhosis have an increased risk of having a positive FIT test compared to patients without cirrhosis. Given that rates of adenomas were similar between FIT positive patients with cirrhosis and FIT positive patients without cirrhosis, those with cirrhosis may be at an increased risk for developing colonic adenomas.

*Funding Agencies: *None

# Sub article: A221: Crohn's Disease Incidence and Municipal Soft Water in the Province of Quebec

**Authors:** R. Issenman, S. Odeh

**Affiliations:** ^221.1^McMaster UniversityHamiltonONCanada

*Background*. Global mapping of the incidence of Crohn's Disease, (Economou) indicates that Crohn's Disease occurs with unusually high incidence in urban “hot spots” in Canada, Wales, Scotland, Australia and New Zealand. These urban areas are all located in alluvial flood plains with remarkably soft water.

*Aims*. We asked whether water softness correlated with the incidence of Crohn's disease in Quebec province for which comprehensive population health data exists. (P. Michel et. al. Geographical Variation of Crohn's Disease Residual Incidence in the Province of Quebec, Canada. International Journal of Health Geographics (2010) 9 : 22).

*Methods*. We compared RAMQ administrative data (1995–2000) on the incidence of Crohn's disease in an adult population in Quebec (Michel) to governmental reports of water quality defining the calcium content of soft water as (<100 ppm total mineral content) and hard water as (>170 ppm total mineral content) for areas outside of the major urban areas using the Fisher exact *t*-test at *p* = 0.05 level of statistical significance.

*Results*. Data on both the incidence of Crohn's disease and water quality was available for 60 of a total of 67 administrative units accounting for a total population of 1,000,000 out of a total of 7,200,000 adults living in Quebec at the time (1996). The Montreal metropolitan area was excluded as the diversity of an intense urban area would tend to obscure any possible correlations Thirty districts were categorized as having a high or very incidence of CD and 30 districts described as having a low or very low. 13 of 30 districts with high or veryhigh CD Incidence non-soft water. compared to 25 districts characterized by a low incidence of CD and non-soft water suggesting a significant association between high or very CD incidence and municipal soft water provision (*p* = 0.04). By contrast hard water could not be shown to confer protection from the development of CD.

*Conclusions*. The Darwin lakes project (Smohl) has shown that the progressive softening of waters in the Great Lakes/St. Lawrence watershed over the past 30 years resulting from the combined effects of acid rain and deforestation has had a profound effect on the aquatic biology in these source waters for 30,000,000 people who live in eastern Canada and the Northeastern US. This study raises the question of whether the loss of natural chelation of minerals in water interacting with other changes in the environment are related to the dramatic increases in the incidence of CD and atopic disease in this population over the same time period. Comparative data sets indicate a correlation between extremes of soft water and a higher incidence of CD in Quebec Province. We are presently examining the role of water quality on alterations of the intestinal permeability and the microbiome to better understand this association.

*Funding Agencies: *None

# Sub article: A222: A Longitudinal Population-Based Evaluation of Inflammatory Bowel Disease Drug Utilization in Saskatchewan

**Authors:** J. Jones, S. Stewart, J. Pena-Sanchez, S. Fowler, L. Lix

**Affiliations:** ^222.1^QE II HSCDalhousie UniversityHalifaxNSCanada; ^222.2^QE II HSCDalhousie UniversityCanadaNSCanada; ^222.3^University of SaskatchewanSaskatoonSKCanada; ^222.4^ManitobaWinnipegMBCanada

*Background*. Evidence-based recommendations relating to Inflammatory Bowel Disease (IBD) pharmacotherapy (PT) have changed over time with more emphasis on prevention using steroid-sparing medications.

*Aims*. To describe the frequency of IBD prescription drug dispensals in a Saskatchewan (SK) population-based IBD cohort and to determine the influence of prescription decade, patient demographics, prescriber specialty, and geographic locale on the frequency of prescription dispensation.

*Methods*. This was a historical, population-based cohort study in which adult patients (≥18 years) meeting a validated administrative definition of IBD in the province of SK were identified between 1975 and 2011. The demographic, prescription drug, hospital separation, and physician visit files of the IBD cases were linked internally at the SK Ministry of Health. Descriptive analyses of the frequency of IBD drug dispensations were explored by decade (1970–80; 1981–90; 1991–2000; 2001–11) with results summarized as means (SD and ranges) and medians (SE with IQR). The influence of patient age, gender, IBD diagnosis, disease duration, prescription locale, and prescription provider on the relative frequency of prescriptions of each IBD drug was evaluated.

*Results*. Preliminary analyses reveal that the IBD cohort consists of 8821 prevalent cases. There were 469,295 prescriptions dispensed for IBD-specific medications. Gastroenterologists' (GIs) prescriptions comprised 9% of all dispensals, and 30% of all IBD-specific dispensals. Over time, there was a clear increase in the proportion of IBD medications prescribed by GIs versus other providers. There was a sharp decline in the proportion of patients prescribed SSZ beginning in 1980 (98% in ‘85, 62% in ‘86, 31% in ‘90 to 5% in ‘11) followed by an abrupt rise in the frequency of prescriptions for non-sulpha 5-ASAs. A notable rise in the proportion of dispensals for immunomodulator and anti-TNF was observed. Older patients were less likely to receive IBD medications from a GI than younger ones (38% of prescriptions (age 18–30) from GIs, versus 14% (age 70–80) and 5% (age 80+)). There was also an effect of location, with 40% of prescriptions for urban patients being written by GIs, versus 20% and 23% for rural and semi-rural populations.

*Conclusions*. Changes in IBD drug dispensals over time has likely been influenced by the emergence of high-level evidence supporting the use of these therapies. Additional longitudinal data analyses exploring interactions between decade, health resource utilization and other factors likely to affect drug dispensals are planned.

*Funding Agencies: *Saskatchewan Health Research Foundation and Canadian Foundation for Innovation

# Sub article: A223: The Rising Burden of Inflammatory Bowel Disease in North America from 2015 to 2025: A Predictive Model

**Authors:** S. Coward, F. Clement, T. Williamson, N. Molodecky, G. Hazlewood, K. McBrien, S. Ng, S. Heitman, C. Seow, R. Panaccione, S. Ghosh, G. Kaplan

**Affiliations:** ^223.1^University of CalgaryCalgaryABCanada; ^223.2^Chinese University of Hong KongHong KongChina

*Background*. The prevalence of Inflammatory Bowel Disease (IBD) in North America (NA) has been increasing. The financial burden of IBD is substantial (e.g., in 2012 the annual direct medical cost of IBD exceeded $1.2 billion in Canada).

*Aims*. To predict the prevalence and direct medical costs of Crohn's disease (CD) and ulcerative colitis (UC) in NA in 2015 and 2025.

*Methods*. A systematic review of population-based studies reporting prevalence of CD and UC after 1990 in Canada and the US. Data was extracted, and negative binomial models created to predict the prevalence of CD/UC in Canada and the US in 2015 and 2025. Change in prevalence over time was reported as annual percentage change (APC) with a 95% confidence interval (CI). Extrapolated population data from Statistics Canada and the US Census Bureau was used to estimate the total number of individuals at risk. Consumer Price Indexes were used to extrapolate direct medical costs.

*Results*. In 2015 the prevalence of IBD in NA is 0.54% and 1,952,470 persons are estimated to have IBD, with the prevalence significantly increasing by 2.39% per year (95% CI: 1.39%, 3.41%) over the next decade (Table 36). The prevalence of CD will rise significantly in Canada (APC = 3.75; 95% CI: 2.57,4.93) and the US (APC = 3.27; 95% CI: 0.17,6.53) (Table 36). By 2025, 0.9% of Canadians and 0.6% of Americans will have IBD, with direct healthcare costing over $28 billion (Table 36).

*Conclusions*. The prevalence of IBD will rise significantly over the next decade with 0.7% of individuals living in NA having IBD. In 2025 over 2.7 million individuals will have IBD and over $28 billion will be spent on direct healthcare costs. The substantial burden of IBD in NA over the next decade necessitates multifactorial solutions including innovating the delivery of healthcare and studying interventions that prevent the development of IBD.

*Funding Agencies: *None

# Sub article: A224: Gastrointestinal Symptoms in Children and Adults with Type 1 Diabetes: Relation to Diabetes Complications, Glycemic Control and Presence or Absence of Celiac Disease

**Authors:** F. Mahmud, E. DeMelo, K. Noordin, A. Clarke, K. Sahota, D. Nandamalvan, E. Assor, P. Marcon, O. CD-DIET Study Group

**Affiliations:** ^224.1^The Hospital for Sick ChlidrenTorontoONCanada

*Background*. The reported frequencies and type of gastrointestinal (GI) symptoms in patients with type 1 diabetes (T1D) is variable across studies but appears to be related to age and complications of T1D.

*Aims*. To describe GI symptoms and associated comorbidities in T1D children and adults.

*Methods*. Individuals aged 8–45 years with T1D duration ≥ 1 year completed a self-reported questionnaire—Gastrointestinal Symptom Scale (GISS) as part of the screening phase of the Celiac Disease & Diabetes—Dietary Intervention & Evaluation Trial (CD-DIET). GI symptoms were evaluated over the previous 7 days, with a 9-item symptom questionnaire and a Visual Analog Score for symptom severity.

*Results*. 1735 patients completed the questionnaire; 341 (19.6%) children aged 8–12 years, 577 (33.3%) adolescents aged 13–18 years and 817 (47.1%) adults aged 19–45 years. Overall, 76.5% children, 79.7% adolescents and 70.3% adults reported no GI symptoms. Within the group that reported ≥1 GI symptoms (*n* = 440), the most commonly reported symptoms in children were upper (46.3%) and lower (37.5%) abdominal pain, and nausea (25.0%); similar to adolescents who reported lower (38.5%) and upper (34.2%) abdominal pain as well as nausea (29.1%). In adults, loose stool (38.3%), lower (37.4%) and upper (23.0%) abdominal pain were most frequently reported. While no statistically significant differences were seen within age groups in terms of frequency of symptoms, the reported frequencies of both loose stool (*p* < 0.0001) and upper GI pain (*p* < 0.001) were signifcant different between age groups. Overall, subjects who reported ≥1 complication (*n* = 334) were 1.43 times more likely to report at least one GI symptom (*p* = 0.0069) with those reporting the presence of autoimmune thyroid disease or diabetes retinopathy being 1.51 and 2.32 times more likely to report a GI symptom (*p* = 0.023  &  *p* = 0.014, resp.). The average duration of T1D was 4.98 ± 2.78 years for children, 6.99 ± 4.07 years for adolescents, and 16.58 ± 8.92 years for adults. Overall, those who reported a GI symptom had a significantly greater duration of T1D (12.4 years) compared to those who did not report any GI symptoms (10.7 years) (*p* = 0.0064). No significant associations were identified between GI symptoms and glycemic control (HbA1c), or between GI symptoms and the presence of Celiac Disease detected through screening.

*Conclusions*. The frequency of GI symptoms was low in all age groups of T1D subjects studied. The pattern of symptoms differed between the age groups and a significant association was seen between GI symptoms and T1D complications and duration.

*Funding Agencies: *None

# Sub article: A225: People with Depression Have Increased Risk of Developing the Inflammatory Bowel Diseases (IBD)

**Authors:** A. Frolkis, I. Vallerand, J. Hubbard, M. Lowerison, E. Jordan, M. Swain, C. Barnabe, S. Patten, G. Kaplan

**Affiliations:** ^225.1^University of CalgaryCalgaryABCanada

*Aims*. We assessed the effect of depression on risk of developing the inflammatory bowel diseases (IBD).

*Methods*. The Health Improvement Network (THIN) was used to identify a cohort of patients with new onset depression using Read Codes for a diagnosis of depression. THIN patients that did not meet the diagnostic criteria for depression were controls. To avoid inclusion of prevalent depression cases, a one-year washout period was applied to all patients following registration in THIN. The outcome of incident IBD was defined using a validated algorithm. Parametric and nonparametric tests were conducted to provide descriptive statistics and compare age, sex, and socioeconomic status in the depression and control groups. Cox proportional hazards multivariable regressions were performed to evaluate the risk of the outcome of IBD among patients with an exposure of depression after controlling for age, sex, and socioeconomic status.

*Results*. We identified 9,831,356 patients, of whom 416,385 (4.2%) developed new onset depression. Median follow-up time was 5.1 years; (interquartile range (IQR) 9.1). Median age at index date for those with depression (35.5; IQR 24.1) and the control group (29.4; IQR 32.1) was significantly different (*p* < 0.001). The sex distribution was also significantly different between those with and without new onset depression (65% versus 51% female; *p* < 0.001). A total of 293 depression patients developed IBD, compared to 6,021 in the control group. Depression was associated with an increased risk of developing IBD (hazard ratio (HR) 1.16; 95% confidence interval (95% CI) 1.03–1.31), even after adjustment for age, sex, and socioeconomic status (HR 1.13; 95% CI 1.01–1.27).

*Conclusions*. Depression increases the risk of developing IBD, both independently and after adjustment for confounders. These results may impact counseling and management of both depression and IBD.

*Funding Agencies: *Alberta Innovates Health Solutions

# Sub article: A226: A Population-Based Cohort Study Evaluating the Impact of the Saskatchewan Multidisciplinary Inflammatory Bowel Disease Clinic

**Authors:** J. Pena-Sanchez, J. Jones, L. Lix, W. Li, G. Teare

**Affiliations:** ^226.1^University of SaskatchewanSaskatoonSKCanada; ^226.2^Dalhousie UniversityHalifaxSKCanada; ^226.3^University of ManitobaWinnipegMBCanada; ^226.4^Health Quality CouncilSaskatoonSKCanada

*Background*. Integrated models of care bring together a multidisciplinary team of healthcare providers to ensure timely, multifaceted care including the appropriate diagnosis, treatment, and management of patients with inflammatory bowel disease (IBD). The Saskatchewan (SK) Multidisciplinary IBD Clinic (MDIBDC) was established in 2009, implementing a patient-centred healthcare model for patients with IBD. Objective evaluations of the impact these models of care have on health resource utilization have not been undertaken.

*Aims*. To evaluate the impact of the MDIBDC exposure on health resource utilization and prescription drug utilization by patients with a diagnosis of IBD in SK. The objectives are to estimate rates of IBD-related hospitalizations, surgical treatments, and prescription medication utilization amongst a cohort of patients managed within the MDIBDC and to compare these rates to those patients not managed within the clinic.

*Methods*. This is a retrospective population-based cohort study from 2009 to 2015. The cohort includes all SK adult residents with a diagnosis of IBD as per a validated administrative case definition for IBD. This definition is based on diagnoses recorded in hospital records and physician billing claims. The study cohort will be classified as exposed and non-exposed to the MDIBDC. Regression models, with propensity score adjustment to control for confounding effects of healthcare utilization variables, will be used to test for differences in IBD-related hospitalizations, surgical treatments, and prescription medications between the groups.

*Results*. This work is in progress. Analysis of data to date reveals that there were 6,736 IBD cases within the cohort during the study period. More than half of the cases (52.4%) were women. The mean age and duration with the disease were 42.2 (SD = 16.4) years and 9.2 (SD = 5.3) years, respectively. The median numbers of physician visits and hospitalizations with the diagnosis of IBD were 22 and 4, respectively. Individuals were classified as exposed or non-exposed. Encrypted billing codes of physicians within the MDIBDC were used to link their billing codes with the administrative data of each cohort member. We expect to have further results in February 2016.

*Conclusions*. Our research will demonstrate the impact of the MDIBDC on health resource utilization and prescription drug utilization of patients with IBD. We hypothesize that differences will exist in the rates of IBD-associated hospitalizations, IBD-associated surgeries, and the proportion of individuals with prolonged corticosteroid exposure between individuals managed and non-managed within the MDIBDC.

*Funding Agencies: *Saskatchewan Health Research Foundation and Crohn's & Colitis Canada

# Sub article: A227: Defining the Epidemiological Features and Specialist Referral Patterns of Primary Biliary Cirrhosis (PBC) Patients within A Defined Canadian Population

**Authors:** A. Shaheen, J. Newman, H. Nguyen, P. Crotty, M. Fritzler, M. Swain

**Affiliations:** ^227.1^University of CalgaryCalgaryABCanada; ^227.2^Univ CalgaryCalgaryABCanada

*Background*. Primary Biliary Cholangitis (PBC) is an autoimmune liver disease that can progress to cirrhosis and death. Treatment of PBC patients with UDCA can slow disease progression. A positive serological test for anti-mitochondrial antibody (AMA) is diagnostic for PBC.

*Aims*. to assess referral patterns of all patients that had AMA (titer ≥ 1 : 80) and to compare the demographic and clinical characteristics of this cohort according to whether they were followed by a specialist or non-specialist care provider.

*Methods*. We identified all adult patients with a newly found positive AMA (titer ≥ 1 : 80) within the Calgary Zone (Alberta Health Services), between April 1st 2012 and December 31st 2014 (approved by Ethics Review Board, University of Calgary). Physicians who had ordered the AMA testing were identified and classified as gastroenterologist, hepatologist, or other (internist or family doctor). The time from referral to clinic appointment was determined for all referrals made to a gastroenterologist or hepatologist. Patient demographic and laboratory data were obtained for all AMA +ve patients. Non-parametric statistical methods were used for analyses.

*Results*. We identified 146 AMA positive patients, and 113 patients (77.4%) had an AMA titer ≥ 1.80. Of these 113 patients, 78 (69.0%) were followed by a hepatologist, and 6 (5.3%) by a gastroenterologist. Among patients referred to a hepatologist (*n* = 33/78 (42.3%)), the median time from referral to clinic appointment was 245 days (IQR 161–435). Five patients died in our cohort during the study period, and 3 of these patients had been evaluated by either a gastroenterologist or hepatologist prior to death. The group of patients referred to a gastroenterologist or hepatologist were compared to the group that were not referred, and were found to be of similar with regards to median age, gender distribution, and magnitude of liver test abnormalities. However, patients who were not referred to specialist care had significantly lower AMA titers (median: 1 : 160 versus 1 : 640, *P* = 0.01).

*Conclusions*. We identified that 25% of PBC cases with high titer AMA positivity were not referred to specialist care. Given the availability of an effective therapy (i.e., UDCA), our research goals are to determine the reasons for AMA testing and for different referral patterns for patients found to be AMA +ve, delineate characteristics of those patients treated with UDCA, and to document biochemical response to UDCA therapy in treated patients according to referral pattern.

*Funding Agencies: *CIHR

# Sub article: A228: MOSAIC: An International Multicenter Prospective Observational Study to Evaluate the Epidemiology, Humanistic and Economic Outcomes of Treatment for Chronic Hepatits C Virus (HCV)- Interim Analysis, Canada

**Authors:** N. Ackad, W. Ghesquiere, S. Shafran, E. Tam, S. Lee, D. Webster, M. Elkashab, C. Pinsonnault

**Affiliations:** ^228.1^Abbvie CorporationSaint*-*LaurentQCCanada; ^228.2^University of British ColumbiaVictoriaBCCanada; ^228.3^University of AlbertaEdmontonABCanada; ^228.4^LAIR CentreVancouverBCCanada; ^228.5^University of CalgaryCalgaryABCanada; ^228.6^Saint John Regional HospitalSaint JohnNBCanada; ^228.7^Toronto Liver CentreTorontoONCanada

*Background*. Direct acting antivirals present an alternative to Interferon (IFN) based therapy for patients with chronic Hepatitis C (c-HCV).

*Aims*. The MOSAIC study Aims to characterize patients with c-HCV and assess the impact of IFN-containing treatment on health-related quality of life, work-related productivity, activities of daily living and health care utilization.

*Methods*. MOSAIC is a prospective, observational study conducted in 30 countries. Consecutive c-HCV patients were enrolled; with 48 week follow up for those who initiated an IFN-based treatment within 12 weeks of enrolment. We report the interim 12-week results from the Canadian cohort.

*Results*. By December 2014, 335 patients were invited and 278 were enrolled: 188 treatment naïve and 90 treatment experienced. 54 (19.4%) patients were initiated on IFN-based regimen: DAA + peg-IFN + RBV (*n* = 5), peg-IFN + RBV (*n* = 18), IFN + RBV (*n* = 1) and other (*n* = 30). For physicians and patients respectively, waiting for other treatment options (37.5% and 12.9%), presumed tolerability (14.7% and 7.1%) and for physicians contraindication (18.5%) were the most frequent reasons for not starting IFN treatment.

Mean (SD) (Range) age of the 54 treated patients was 51.8 (10.30) (31–74) years; with mean (SD) (Range) duration of HCV diagnosis of 10.9 (10.2) (0–46) years. Of the 54 treated patients 11 had cirrhosis (1 decompensated), 20 minimal or no fibrosis, 15 bridging fibrosis, 4 portal fibrosis and for 4 fibrosis stage was not reported. Genotype was G1: 34 (63.0%), G3: 11 (20.4%), G2:6 (11.1%) and *n* = 1 (1.9%) each for G4, G5 and G6. Baseline HCV-RNA level was <800,000 IU/mL: 17 (31.5%), > 800,000 IU/mL: 26 (48.1%) × IU/mL, positive by qualitative assay: 1 (1.9%), and not reported: 10 (18.5%).

Of the treated patients 25 (46.3%), 17 (35.4%) and 10 (27.8%) respectively were employed at baseline, 4 and 12 weeks.

During the first 12 weeks of treatment 15 (28%) patients had ≥1 outpatient consultations of which 5 had 1, 4 had 2-3, 4 had 4-5 and 2 had 7-8. No hospitalizations occurred during the first 12 weeks of treatment.

*Conclusions*. A small proportion of c-HCV patients are treated with IFN and they experience a substantial impact on QoL and productivity. Anticipated availability of new IFN-free regimens is the main reason for not initiating treatment with IFN.

*Funding Agencies: *None

# Sub article: A229: Cumulative Results of Helicobacter Pylori Therapy Using Sequential Therapy, Bismuth Quadruple Therapy, Levofloxacin Based Triple Therapy and Other Regimens

**Authors:** J. Buttenschoen, P. D'Souza, S. Veldhuyzen van Zanten

**Affiliations:** ^229.1^University of AlbertaEdmontonABCanada

*Background*. Success rates of classic first line therapy to treat* Helicobacter pylori *(Hp) infection, PPI-Clarithromycin (C) Amoxicillin (A) or PPI-C-metronidazole (M) have been declining in Canada. Better new regimens include sequential and concomitant therapy. 10 day twice daily Sequential therapy (ST) consists of a PPI and amoxicillin 1.0 g for 5 days, followed by PPI-CM (both 500 mg bid) for an additional 5 days. Alternative treatment regimens include bismuth based quadruple therapy with tetracycline, metronidazole (PPI-BMT) and the twice daily combination of PPI amoxicillin and Levofloxacin (500 mg bid), PPI-AL.

*Aims*. To determine the efficacy of the sequential, bismuth quadruple, levofloxacin therapies and other regimens for eradication of Hp and to determine the cumulative success rates when given following treatment failures.

*Methods*. Patients diagnosed with* H*.* pylori *infection on histology or urea breath test were included inthis retrospective study. Cure of Hp was assessed by histology and/or culture or UBT. Patients who had no post-treatment testing for Hp were excluded. The per protocol efficacy of different regimens was calculated as well as cumulative success rates when given as 2nd, 3rd or 4th line therapy.

*Results*. 350 patients met the inclusion criteria. 14 patients were lost to follow-up or had no assessment for cure done. Of the remaining 336 patients 43% were male and 57% female. The mean age was 51 ± 16.4 years, range 13–90 years. Treatment duration was 7 days in 19% 10 days in 63% and 14 days in 18%. Success rates of the different regimens are shown in Table 37. PPI-CA cure rate was 45% (25/56) for duration of therapy of 7 days, 41% (7/17) for 10 days and 63% (17/27) for 14 days.

Our preferred 10 day treatment strategy: 1st ST, 2nd PPI-BMT, 3rd PPI-AL resulted in a cumulative success rate of 96%. Fourth line 10 day treatment with PPI-A (1.0 g bid) and Rifabutin (150 mg bid) cured an additional 50% (2/4) of patients.

*Conclusions*. The efficacy of standard triple therapy (PPI-CA) for H. pylori is unacceptably low and no longer should be used. 10 day sequential therapy was superior to triple therapy for first-line treatment and achieved cure in 82%. The treatment strategy: sequential 1st line, bismuth quadruple 2nd and levofloxacin triple 3rd cures 96% of patients.

*Funding Agencies: *None

# Sub article: A230: Red Blood Cell Transfusions and Iron Therapy for Patients Presenting with Acute Upper Gastrointestinal Bleeding: A Survey of Gastroenterologists

**Authors:** K. Fortinsky, M. Martel, R. Razik, G. Spiegle, S. Grover, K. Pavenski, A. Weizman, Z. Gallinger, L. Kwapisz, A. Barkun

**Affiliations:** ^230.1^Mount Sinai HospitalTorontoONCanada; ^230.2^McGill University Health CenterMontrealQCCanada; ^230.3^UWOWhitbyONCanada; ^230.4^McGill UniversityThe Montreal General HospitalGI DivisionMontrealQCCanada

*Background*. There currently exists only 1 completed RCT to evaluate transfusions in patients with acute upper gastrointestinal bleeding (UGIB). Physician transfusion practices in UGIB are largely based on experience and can vary considerably.

*Aims*. To document gastroenterologists' current transfusion practices and iron prescribing rates to patients with acute upper gastrointestinal bleeding.

*Methods*. A web-based survey was sent to 500 gastroenterologists across Canada. The survey included simulated cases (see Table 38) where physicians were required to choose specific transfusion thresholds as well as multiple-choice questions related to iron therapy and current guidelines. Descriptive and inferential statistics (Chi-square and *t*-tests) were carried out.

*Results*. The overall questionnaire response rate was 41%. Transfusion practices differed by up to 50 g/L in terms of hemoglobin (HgB) thresholds for transfusion. Transfusions were more liberal in hemodynamically unstable patients compared to stable patients (mean HgB of 86.7 g/L versus 71.0 g/L, *p* < 0.0001). 57% of respondents transfused 2 units of RBC's as initial management. Patients with coronary artery disease (mean HgB of 84.0 g/L versus 71.0 g/L, *p* < 0.0001) or cirrhosis (mean HgB of 74.4 g/L versus 71.0 g/L, *p* < 0.01) were transfused at higher thresholds than healthy patients, as were patients on warfarin (mean HgB of 75.3 g/L versus 71.0 g/L, *p* < 0.001). Only 15% or respondents would transfuse more liberally if the patient was on dabigatran, rivaroxaban, or apixaban. 56% of respondents felt more likely to be held legally responsible for the complications related to “under-transfusing” than the complications associated with “over-transfusing”. Only 15% of gastroenterologists prescribe iron to patients with UGIB who are anemic upon discharge.

*Conclusions*. Healthy and hemodynamically stable patients are being transfused at a HgB below 70 g/L while higher thresholds are used in patients who are unstable or who have underlying cardiac disease or cirrhosis. Many clinicians are not following current guidelines and are transfusing patients at a HgB threshold of 100 g/L. Few clinicians are prescribing iron on discharge to anemic patients. The transfusion practices of gastroenterologists in the vary widely and more high-quality evidence is needed to assess the efficacy and safety of selected transfusion thresholds in patients with UGIB.

*Funding Agencies: *None

# Sub article: A231: A Canadian Experience with a Protocolized Barrett's Endoscopic Therapy Program: Durability, Efficacy and Outcomes from 2010–2015

**Authors:** T. Ishikawa, P. Belletrutti, M. Gupta

**Affiliations:** ^231.1^University of CalgaryCalgaryABCanada

*Aims*. Radiofrequency ablation (RFA), +/− Endoscopic Mucosal Resection (EMR), has been validated as a treatment option for Barrett's esophagus (BE) and early esophageal cancer. However, little is known regarding outcomes of BE therapy in Canada. We set out to evaluate the efficacy, durability and safety of these techniques in Calgary, a tertiary care center in Canada.

*Methods*. A retrospective review of a prospectively maintained database of patients undergoing RFA +/− EMR for biopsy-proven intestinal metaplasia (IM), dysplasia (D), or intramucosal carcinoma (IMCa) from June 2010 to April 2015. Our primary outcome was the rate of complete remission of IM (CRIM) or dysplasia (CRD) defined as confirmed eradication on two negative consecutive endoscopies with biopsies. Secondary outcomes were treatment-related complications, number and type of treatments used and quality of life (QoL) based on a questionnaire previously used in the BE literature.

*Results*. 31 patients (27 male; mean age 62 ± 11 years), with a mean BE length of 6.9 cm (±3.8 cm) underwent endoscopic therapy for BE from 2010 to 2014 (two with IMCa, ten with high grade dysplasia (HGD), ten with low grade dysplasia and nine with no dysplasia (ND), but additional risk factors for esophageal cancer (long segment, family history of esophageal cancer or age of BE diagnosis < 30)). EMR was also performed in 9 (29%) patients. CRIM/D were achieved in 28/31 (90.3%) of patients. Patients required a mean of 3.1 ± 1.3 RFA sessions to achieve CRD and 3.2 ± 1.3 RFA sessions to achieve CRIM. During a mean follow-up period of 17.9 ± 11.8 months, durable CRIM/D was achieved in 27/28 (96.4%) patients (one IM recurrence only). Treatment-related complications included stricture formation requiring balloon dilation in 2/31 (6.4%) and chest pain requiring hospitalization in 1/31 (3.1%). The main QoL outcome was 5/7 (71.4%) felt reduced stress about the present condition of their esophagus one year after starting treatment.

*Conclusions*. In the medium term, RFA +/− EMR is a safe, effective and durable treatment option for BE in Canada. In Calgary, greater than 80% of patients achieved CRIM/D and >95% maintained CRIM/D at a mean of 18 months follow-up. Patients also perceive a reduction in stress pertaining to BE after intervention.

*Funding Agencies: *None

# Sub article: A232: Eosinophilic Oesophagitis: Demographics & Disease Characteristics in New Zealand Children. A Prospective Study

**Authors:** A. Sheikh, A. Day, J. Sinclair, N. Dickson, H. Evans

**Affiliations:** ^232.1^Starship Children's HealthAucklandNew Zealand; ^232.2^University of OtagoChristchurchNew Zealand; ^232.3^New Zealand Paediatric Surveillance UnitDunedinNew Zealand

*Background*. Eosinophilic oesophagitis (EoE) is a rare, chronic, & relapsing immune/antigen-mediated disease characterised by symptoms of oesophageal dysfunction with an eosinophil predominant inflammation of the oesophageal mucosa. There is a paucity of data among the New Zealand (NZ) paediatric population.

*Aims*. This 3-year prospective study aimed to characterise this disease better in NZ children, and to verify initial treatment strategies adopted by physicians throughout the country. Here we present preliminary data from the first 19 months of the study.

*Methods*. Information on new diagnoses of paediatric EoE was obtained via the NZPSU through monthly questionnaires sent out to all paediatricians & other specialists working with children throughout NZ.

*Results*. 31 new cases (28 male) were reported to the NZPSU from Feb 2014 to Aug 2015. 74% were of European descent with a median age of 8 years (0.6–15). Dysphagia was the most common symptom (35%), followed by vomiting (29%), food refusal (26%), epigastric pain (19%) & weight loss (19%). Other symptoms reported were food impaction, nausea, failure to thrive, non-specific abdominal pain, and diarrhoea. 2 patients were asymptomatic. 71% had a co-morbid history of & 55% had at least one first degree relative with atopy or food allergy. 61% had abnormal endoscopic findings, of which linear furrows and white plaques were the most common. 39% had normal oesophageal mucosa on endoscopy. Only 35% received a proton pump inhibitor (omeprazole) prior to endoscopy; 4 patients continued this post-endoscopy. 9 patients (29%) were initially managed with dietary manipulation alone (7 with an elimination diet, 2 with an elemental formula); 1 patient required a nasogastric tube for their feeds. 19 (61%) and 3 (10%) patients were treated with swallowed fluticasone propionate and oral prednisone respectively. Leukotriene receptor antagonists and immunosuppressive therapy were not used in any of the patients. 25 patients (81%) have a repeat endoscopy planned to monitor response to treatment.

*Conclusions*. The demographics and disease characteristics of our patients with paediatric onset EoE in NZ are similar to that reported in the current medical literature. Long term prospective observational data obtained from this cohort of patients, should significantly improve our knowledge of this rare condition.

*Funding Agencies: *None

# Sub article: A233: Is the Lymphocytic Esophagitis a New Clinical Entity?

**Authors:** D. Daoud, A. Therrien, G. Soucy, M. Bouin

**Affiliations:** ^233.1^CHUMMontréalQCCanada; ^233.2^Centre Hospitalier de l'Université de MontréalMontrealQCCanada; ^233.3^CHUMMontrealQCCanada

*Background*. Lymphocytic esophagitis is a new pathological entity. Its clinical significance is still unknown. The association with some digestive symptoms remains to be established.

*Aims*. Our hypothesis is that the lymphocytic esophagitis is associated with a specific symptomatology and endoscopic features. Therefore, our objective was to identify clinical and endoscopic characteristics associated with lymphocytic esophagitis.

*Methods*. A retrospective study from January 1st, 2010 to June 1st, 2015 was performed at the University of Montreal Medical Center. From the internal database of the department of pathology, all patients with a histological diagnosis of lymphocytic esophagitis were selected for inclusion. Patients with oesophageal cancer, eosinophilic esophagitis and oesophageal surgery were excluded. The demographic, clinical, endoscopic and histopathological data were obtained through review of computerized medical records.

*Results*. 74 patients were included (mean age 56.7 ± 16 years, 53% men). The main gastrointestinal symptoms warranting investigation were dysphagia (61%), heartburn (34%) and chest pain (11%). The main indications for gastroscopy were: a suspicion of esophagitis (41%), a suspected neoplasia (13%), a follow-up for Barrett oesophagus (9%), an oesophageal stricture (9%), GERD which do not respond to PPI (8%), or anemia. The endoscopic features were: a normal oesophagus or an uncomplicated hiatal hernia (44%), esophagitis or ulcers (15%), oesophageal stenosis (12%), a suspicion of Barrett's oesophagitis (9%), white plaques (8%), oesophageal polyps (7%), oesophageal ring (6%), felinization (5%), candida (3%).

*Conclusions*. Lymphocytic Esophagitis affects both middle-aged men and women. The main symptom is dysphagia in 61% of cases but gastroesophageal reflux is present in one third of cases. Gastroscopy is normal in 44% of cases, but lesions in favour of GERD are often concomitant (esophagitis, ulcers, stricture, Barrett) which suggests a possible pathophysiological link.

*Funding Agencies: *None

# Sub article: A234: The Prevalence of Helicobacter Pylori in Quebec Is Low and Highly Dependant on the Country of Origin

**Authors:** G. Hassan, J. de Repentigny, S. Sidani, G. Soucy, M. Bouin

**Affiliations:** ^234.1^Centre Hospitalier de l'Université de MontréalMontréalQCCanada; ^234.2^Université de MontréalMontrealQCCanada; ^234.3^Centre Hospitalier de l'Université de MontréalMontrealQCCanada

*Background*. The prevalence of Helicobacter pylori (Hp) infection in Canada is estimated between 20 to 30% of the population [1-3]. Several studies have shown, however a significant decrease in the prevalence of Hp infection in Western countries because of its effective eradication treatment. Among the available tests, identification of Hp on endoscopic biopsies has excellent sensitivity and specificity if biopsies are made as recommended. There is currently no data on the prevalence of Hp in Quebec.

*Aims*. The aim of this study was to evaluate the prevalence of Hp infection in Quebec. The secondary objectives were to investigate demographic factors associated with this infection and to estimate the quality of endoscopic biopsies.

*Methods*. Retrospective, Cross-sectional study of 500 patients who had esophago-gastro-duodenoscopy (EGD) with gastric biopsies to look for Hp, from July 1st to December 31, 2011. Of these, 150 cases were randomly selected to study the quality of biopsies (localization) and concomitant use of anti-secretory medications (PPIs or H2 blockers) and/or antibiotics. The main criterion for exclusion was an incomplete medical record or EGD report. Demographic variables studied were age, sex, country of birth, indication for EGD, endoscopic findings and presence or absence of Hp on histology. The statistical analysis used consisted of a logistic regression of variables associated with Hp.

*Results*. During the 6 months study, 1351 EGDs were requested to rule out Hp. Analysis of 538 cases was carried out to include 500 cases for the study (38 excluded because of incomplete files). In this population (mean age 56 ± 8 years, 57.1% women) the prevalence of Hp was 13.1%. Age and sex were not significantly different between the groups with and without Hp. The prevalence of Hp was significantly different with place of birth: North America and Western Europe (8%), South America (35%), Africa (25%), Asia (31%). Biopsies were performed in the gastric antrum alone in 55.6% and in the antrum and body in 22.8%. 54% of patients were on anti-secretory therapy and/or under antibiotics for Hp.

*Conclusions*. The prevalence of Hp is 13% in our study population. It is however highly variable depending on the place of birth of the patients. However, the biopsies are rarely performed in both the antrum and gastric body, which could lead to an underestimation of the prevalence of Hp.

*Funding Agencies: *None

# Sub article: A235: Medical Audit: A Practice Review of the Rate of H. Pylori Obtained during Acute Management of Upper Gastrointestinal Bleeding

**Authors:** S. Moosavi, E. Lam

**Affiliations:** ^235.1^University of British ColumbiaVancouverBCCanada

*Background*. Peptic ulcer disease (PUD) is one of the main causes of acute upper gastrointestinal bleed (UGIB). The major risk factors of PUD are Helicobacter pylori (HP) infection and NSAIDs use. The most recent guideline from European Society of Gastroenterology on the management of non-variceal UGIB recommends investigating for the presence of HP in acute UGIB secondary to PUD.

*Aims*. To determine whether obtaining biopsy during the upper endoscopy (EGD) for acute UGIB is a routine practice in our center.

*Methods*. In a six-month period between October 2014 to March 2015, 98 patients were admitted to Saint Paul's Hospital, Vancouver, British Columbia, with initial diagnosis of UGIB. 13 patients were excluded: 6 had UGIB outside the aforementioned period, 2 had no official records of EGDs, and 5 had lower endoscopies. 85 with EGDs for UGIB were included in this study. Patients' age, gender, EGD findings, PUD Forrest classification, HP biopsy, and any further recommendation for HP serology were documented.

*Results*. The average age of included subjects was 66 years, with 29 females and 56 males. 37 patients (41.4%) had documented PUD as the most likely cause of UGIB, with Forrest classification III (23/36), IIC (4/36), IIB (2/36), IIA (5/36), IB (2/36), and IA (1/36), recording the most severe PUD pathology per patient. Other causes of UGIB in index patients were: 10 cases of esophagitis (i.e., post-variceal banding and GE junction ulcers), 9 with gastropathies (i.e., erosions, gastritis), 14 patients with normal EGDs, 7 with angiodysplasias (i.e., AVM, GAVE, portal hypertensive gastropathy), 6 with Mallory-Weiss tears, 1 with a bleeding submucosal lesion, 1 with an ulcerated hyperplastic polyp and 1 with variceal UGIB.

45 patients (52.9%) had HP biopsies from gastric antrum and body. 1 patient became combative prior to planned biopsy, so instead HP serology was recommended. 1 patient who had HP biopsy during EGD was also empirically started on appropriate HP eradication treatment. After looking more closely at the UGIB etiologies, 7 out of 37 (19%) patients with confirmed PUD did not have biopsy obtained for HP or any recommendations regarding further HP testing at the time of endoscopy.

*Conclusions*. We have demonstrated that obtaining H. pylori biopsy in the setting of acute upper gastrointestinal bleeding may not be obtained routinely, despite strong recommendation for such practice during the endoscopic management of UGIB, particularly secondary to PUD. Further quality improvement projects are required to evaluate such limitations, and implement the quality measures to ensure H. pylori biopsy will become part of the routine management of acute upper gastrointestinal bleed in the setting of PUD.

*Funding Agencies: *None

# Sub article: A236: Peroral Endoscopic Myotomy for Jackhammer Esophagus; The Lower Esophageal Sphincter, to Cut or Not to Cut?

**Authors:** R. Bechara, H. Ikeda, H. Inoue

**Affiliations:** ^236.1^Digestive Diseases CentreShowa UniversityTokyoJapan

*Background*. The most recent definition hypercontractile esophagus is a distal contractile integer (DCI) of ≥8000 mmHg·s·cm in ≥20% of swallows and has been coined “Jackhammer Esophagus”. Jackhammer esophagus is rare, occurring in approximately 4% of cases referred to a tertiary esophageal center. Surgical myotomy has not been widely performed due to the usual requirement of a long myotomy to achieve clinical success which necessitates a combined abdominal and thoracic approach. With peroral endoscopic myotomy (POEM), a long myotomy is possible without increased morbidity or technical difficulty.

*Aims*. The inclusion of the lower esophageal sphincter (LES) in the myotomy is debated and variably performed by POEM operators. POEM was performed on four patients with Jackhammer esophagus at our center. Here we present the clinical and manometric results and discuss the treatment implications.

*Methods*. Between March 2014 and July 2015, four patients underwent POEM for treatment of Jackhammer Esophagus at our center. POEM was perfromed in the stadard fashion. When the LES was included in the myotomy, the tunnel was advanced 2-3 cm into the gastric cardia. However, when the LES was not included the tunnel and myotomy were advanced to the proximal end of the gastroesophageal junction. Esophageal manometry, upper endoscopy, and clinical (Eckardt score, GERD symptoms) examinations were performed 2 months post-POEM.

*Results*. The manometric and clinical results are summarized in Table 39 [Table tab40] [Table tab41] [Table tab42] [Table tab43] [Table tab44] [Table tab45]. All patients had uneventful procedures without any intra or post-procedure adverse events. No patients developed clinical or endoscopic evidence of reflux. With inclusion of the LES in POEM for Jackhammer esophagus, three patients (#2, 3, 4) had excellent clinical results. In contrast, one patient (#1) in which the LES was not included, developed regurgitation and dysphagia. However, after the second POEM that included the LES, the symptoms of dysphagia and regurgitation resolved.

*Conclusions*. POEM is a suitable treatment for patients with Jackhammer esophagus. Based on our clinical experience, physiologic and manometric observations, we believe the obligatory inclusion of the LES in justified. Inclusion of the LES minimizes the risk of symptom development from iatrogenic ineffective esophageal motility or subsequent progression to achalasia.

*Funding Agencies: *None

# Sub article: A237: Post-Polio Syndrome Dysphagia-Esophageal Fibrosis Poses a Procedural Risk of Upper Esophageal Perforation

**Authors:** J. Coneys, N. Viallet, C. Bernstein

**Affiliations:** ^237.1^University of ManitobaWinnipegMBCanada

*Aims*. Post-polio syndrome (PPS) is clinical diagnosis characterized by neuromuscular weakness, fatigability, and pain occurring years after recovery from acute poliomyelitis. PPS is an uncommon cause of dysphagia and bulbar dysfunction related to impaired oropharyngeal muscle function. The cause of neuromuscular dysfunction in PPS is not definitively understood, but in patients with dysphagia it is assumed that the esophagus would be widely patent and symptoms would occur on a transfer and motility basis. Muscular atrophy is commonly seen post poliomyelitis infection; however, muscular hypertrophy has been reported in rare instances.

*Methods*. There are no prior reported cases of structural abnormalities of the hypopharynx or proximal esophagus in association with PPS and there are no reports of an associated risk of perforation at the time of endoscopy.

*Results*. A 74 year old woman presented with a 5 year history of progressive oropharyngeal solid and liquid dysphagia. She had initial paralytic poliomyelitis as a teenager with both bulbar and limb muscle involvement. Her dysphagia improved within approximately one year of infection with mild residual solid food dysphagia. Her swallowing symptoms were relatively stable for approximately 50 years prior to deterioration.

Initial investigation via laryngoscopy showed no structural abnormality; however, a video fluoroscopic swallowing study showed severely impaired pharyngeal function with silent aspiration and reduced opening of the upper esophageal sphincter.

At the time of esophagogastroduodenoscopy (EGD) there was difficulty with esophageal intubation, thought related to cricopharyngeal spasm. A complete EGD was performed without evident abnormality. Post procedurally there was increasing pain and subsequent CT scanning showed significant retropharyngeal air and pneumomediastinum.

Urgent ENT evaluation and esophagoscopy showed an abrasion and stenosis at the level of the cricopharyngeus that prevented esophageal intubation necessitating placement of a 24 f bougie. Subsequent open left neck exploration showed a 5 mm perforation at the level of the cricopharyngeus. The cricopharyngeus was thickened and densely fibrotic with the consistency of very thick scar tissue. A cricopharyngeal myotomy was performed for improvement in symptoms. No specimen was submitted to pathology.

*Conclusions*. Dysphagia is a common symptom in PPS patients and EGD is a commonly performed investigation in the evaluation of dysphagia. Our case highlights a possible increased risk of EGD in this patient population attributable to anatomic and functional changes of the upper esophageal sphincter leading to muscular thickening and fibrosis. We recommend appropriate caution at the time of EGD in this patient group and minimization of unnecessary procedures.

*Funding Agencies: *None

# Sub article: A238: Pediatric Esophageal Strictures; Presentation, Causes and Management at a Tertiary Care Hospital

**Authors:** A. Assiri, A. Saeed, A. Sarkhy

**Affiliations:** ^238.1^Prince Abdullah bin Khalid Celaic Disease Research ChairKing Saud UniversityRiyadhSaudi Arabia; ^238.2^King Khalid University HospitalKing Saud UniversityRiyadhSaudi Arabia

*Background*. Esophageal strictures in children are not uncommon and the causes are variable ranging from structural defects, corrosive injury, inflammatory/allergic conditions and acidic reflux. Gastroesophageal reflux and eosinophilic esophagitis are commonly seen conditions in children mainly presenting with vomiting, dysphagia, food impaction, abdominal pain and rarely with stricturing affect. Endoscopy is the most frequently used modality for diagnosing as well as for managing these children with balloon dilatation.

*Aims*. To describe the clinical characteristics, causes and management outcomes of esophageal strictures among Saudi children.

*Methods*. This is a retrospective analysis of children diagnosed with esophageal strictures over a period of seven years in a single tertiary care center. We included children younger than 18 years of age with diagnosis of esophageal strictures. We collected data about the clinical characteristics of the patients, details of the strictures, endoscopic findings, treatment and the outcomes. Esophageal atresia cases were not included.

*Results*. Twenty eight children were identified with esophageal strictures. Twenty were males (71.4%) with a median age of 9 years (range: 6 months–18 years). The most common presentation was dysphagia in 25 (89.3%) and vomiting in 17 (60.7%) followed by food impaction in 5 (17.9%), epigastric pain in 3 (10.7%) and hematemesis in 1 (3.6%). Gastro-esophageal reflux disease (GERD) 9 (32%) and eosinophilic esophagitis (EOE) 6 (21.4%) were the leading causes of esophageal strictures followed by different types of structural causes; Achalasia 4 (14.3%), Congenital esophageal stenosis 2 (7.1%), esophageal web 1 (3.6%), external compression from vascular ring and from neck mass were seen in 1 (3.6%) each. Caustic strictures were seen in 4 (14.3%). Lower esophageal and short segment strictures were the main features in GERD, EOE and achalasia, long strictures were present mainly in caustic ingestions and few cases of EOE.

Endoscopic dilatation under general anesthesia was the primary modality of treatment in all of these cases. Median number of dilatation sessions per patient was 6.6 (range 1–12) with median duration of 12 weeks (range; 8–18 weeks). No morbidity (esophageal perforation, bleeding) or mortality were associated with the balloon dilation.

*Conclusions*. Esophageal strictures are not uncommon in children. GERD and EOE are the primary causes of esophageal strictures in our group, followed by structural causes. Endoscopic dilatation is a safe modality in managing different types of esophageal strictures.

*Funding Agencies: *None

# Sub article: A239: Correlations between Hepatic Morphometric Collagen Content, Histologic Fibrosis Staging, and Serum Markers in Patients with Advanced Fibrosis due to Nonalcoholic Steatohepatitis (NASH)

**Authors:** K. Patel, S. Jayakumar, G. Minuk, M. Khan, R. Loomba, S. Caldwell, E. Lawitz, S. Harrison, R. Myers

**Affiliations:** ^239.1^University of TorontoTorontoONCanada; ^239.2^University of CalgaryCalgaryABCanada; ^239.3^University of ManitobaWinnipegONCanada; ^239.4^Gilead Sciences CanadaIncMississaugaONCanada; ^239.5^University of CaliforniaSan DiegoCAUSA; ^239.6^North Shore LIJ Heath SystemCharlottesvilleVAUSA; ^239.7^Texas Liver InstituteSan AntonioTXUSA; ^239.8^San Antonio Military Medical CenterSan AntonioTXUSA; ^239.9^GileadFoster CityCAUSA

*Aims*. Our aim was to determine the relationships between hepatic collagen assessed by morphometry with serum fibrosis markers, Ishak fibrosis staging, and other histologic features of NASH in patients with advanced fibrosis.

*Methods*. The study included adults with NASH and bridging fibrosis (Ishak stage 3 or 4) or cirrhosis (stage 5 or 6) enrolled in two phase 2b trials of simtuzumab, a monoclonal antibody against lysyl oxidase-like-2 (LOXL2). Liver biopsies were graded centrally according to the NAFLD Activity Score (NAS) and hepatic collagen in sirius red-stained biopsies was quantified via computer-assisted morphometry. Serum LOXL2 (sLOXL2) was measured using an immunoassay (VIDAS® LOXL2; bioMérieux, Marcy L'Etoile, France). The associations between hepatic collagen and Ishak fibrosis stage, noninvasive fibrosis markers (sLOXL2, FibroTest, ELF, APRI, FIB-4, and NAFLD Fibrosis Score (NFS)), and the NAS and its components were determined.

*Results*. 429 of 477 randomized patients (89.9%) with biopsies acceptable for computerized morphometry were included. The median age was 56 years (IQR 50–60), 62% were female, 52% had cirrhosis, and the median hepatic collagen content was 8.4% (IQR 5.2–14.7%). Hepatic collagen was moderately correlated with fibrosis stage (Spearman *ρ* = 0.59; *P* < 0.001; Figure 27 [Fig fig28] [Fig fig29]). Hepatic collagen was correlated with sLOXL2, ELF, FibroTest, APRI, FIB-4, and NFS (*ρ* = 0.24–0.38; all *P* < 0.001) and inversely associated with NAS (NAS 0–2: 11.9% versus 3-4: 8.7% versus 5–8: 8.0%; *P* = 0.01) and hepatic steatosis (<5%: 12.1% versus 5–33%: 8.2% versus >33%: 7.1%; *P* < 0.001), but was not influenced by lobular inflammation or hepatocellular ballooning.

*Conclusions*. In patients with advanced fibrosis due to NASH, hepatic collagen is correlated with fibrosis stage assessed semi-quantitatively and using serum fibrosis markers. With increasing hepatic collagen, steatosis severity declines.

*Funding Agencies: *Gilead Sciences, Inc.

# Sub article: A240: The Use of Albumin in Decompensated Cirrhosis: Are the Indications Appropriate and the Desired Outcomes Achieved?

**Authors:** X. Xiong, H. Tan, F. Wong

**Affiliations:** ^240.1^Division of GastroenterologyToronto General HospitalUniversity of TorontoTorontoONCanada

*Background*. Albumin is the most abundant protein in the circulation. The recent recognition that it has many physiological functions in addition to the maintenance of oncotic pressure led to increased use in patients with decompensated cirrhosis. The current approved indications include: (i) prevention of circulatory dysfunction following large volume paracentesis (LVP); (ii) diagnosis and adjunctive therapy of hepatorenal syndrome (HRS); and (iii) prevention of HRS in patients with spontaneous bacterial peritonitis (SBP).

*Aims*. To determine the indications and appropriateness for albumin use in patients with decompensated cirrhosis at Toronto General Hospital (TGH).

*Methods*. This was a prospective study enrolling patients who received albumin infusions either as inpatients or outpatients at TGH. Data collected include demographics, etiology and complications of cirrhosis, baseline blood works, indications for albumin use, the dose received and patient outcome. All patients were followed till hospital discharge, and clinical outcome noted.

*Results*. 100 patients (M: 67) at a mean age of 61.4 ± 10.7 years were enrolled, with alcohol (33%), viral hepatitis (36%), or both (2%) as major etiologies of cirrhosis. 99 had ascites at enrolment, and 75 had refractory ascites. 21 had chronic kidney disease (serum creatinine or SCr > 133 *μ*mol/L for >6 months), while 27 had acute kidney injury (acute increase in SCr by either 0.3 mg/dL in <48 hours or by 50% from baseline). Baseline laboratory tests were (mean ± standard deviation): Hgb 107.3 ± 25.1 gm/L, Na 133.6 ± 6.1 mmol/L, SCr 146.9 ± 123.5 *μ*mol/L, INR 1.6 ± 0.6, and albumin 30.5 ± 6.0 g/L. Baseline Child-Pugh score was 9.4 ± 1.7 and MELD score was 17.3 ± 8.1. Amount of ascites drained for LVP was 5.4 ± 2.4 L, with a median dose of 50 g of albumin infused (IQR 25).

*Conclusions*. 80% of albumin use at TGH follows standard guidelines with the desired outcomes. The latest indication in the treatment of non-HRS cases of AKI is an area that deserves further investigations, as albumin is effective in reversing these cases of AKI. The use of albumin in hyponatremia though not an approved indication, appears effective.

*Funding Agencies: *None

# Sub article: A241: Natural History of Patients with Cirrhosis and Hyponatremia

**Authors:** A. Shivji, Y. Tze, F. Wong

**Affiliations:** ^241.1^9N/983 Toronto General HospitalTorontoONCanada

*Background*. Hyponatremia is common in advanced cirrhosis. The clinical setting at which hyponatremia occurs in cirrhosis has not been well documented, and the prognostic implications of increasing severity of hyponatremia not well defined.

*Aims*. To evaluate the clinical setting and the prognostic implications of various levels of hyponatremia in patients with decompensated cirrhosis and ascites.

*Methods*. The hospital electronic database was queried for serum [Na] < 135 mmol/L for all patients for the calendar year 2010. Cirrhotic patients with hyponatremia (serum [Na] <135 mmol/L X2 in ≤48 hours) were included. Exclusion criteria were concomitant known causes of hyponatremia, patients with malignancy, or awaiting a live donation liver transplant, or life expectancy of <1 month were also excluded. Data collected were: demography, blood work, medication use, complications of cirrhosis, co-morbid conditions and survival.

*Results*. 141 cirrhotic patients with ascites (mean age: 57.1 ± 11.4 years, M : F 97 : 44) with 192 episodes of mild (serum [Na] = 131–134 mmol/L), moderate (serum [Na] = 121–130 mmol/L) or severe (serum [Na] ≤ 120 mmol/L) hyponatremia were included. The major etiology of cirrhosis was alcohol (45%), viral (30%), with diabetes (*n* = 40) and systemic hypertension (*n* = 39) as the most common co-morbidities. Complications of cirrhosis noted were hepatic encephalopathy (29%), variceal bleed (17%), and spontaneous bacterial peritonitis (13%). Hepatorenal syndrome only occurred infrequently at 2.1%. Patients were followed for a mean of 318 days. 3 patients received TIPS, 31 had liver transplant and 3 were lost to follow-up. Sixty-one deaths during follow-up were related to liver failure (*n* = 20), sepsis (*n* = 11) and multi-organ failure. Survival was not different between the 3 groups.

*Conclusions*. Hyponatremia is common amongst cirrhotic patients with ascites, associated mostly with diuretic use, leading to hepatic encephalopathy in 1/3 of patients. Increasing severity of hyponatremia is observed with worsening renal dysfunction. Hyponatremia does not seem to impact survival independently in this cohort of patients, as liver failure seems to be the major cause of death in patients with advanced cirrhosis.

*Funding Agencies: *None

# Sub article: A242: Spleen Stiffness: The Missing Link in Detecting Clinically Significant Portal Hypertension

**Authors:** K. Boctor, M. Magnes, M. Elkhashab

**Affiliations:** ^242.1^Toronto Liver CentreTorontoONCanada

*Background*. Varices are a common feature of cirrhosis, and their prevalence parallels the severity of portal hypertension and liver dysfunction. Understandably gasteroesophageal varices, as well as portal hypertensive gastropathy progress at varying degrees and may regress entirely with timely intervention (1). In recent years, transient elastography has been utilized to assess spleen stiffness measurement (SSM), and its role in predicting presence of esophageal varices in cirrhotic patients (2-3).

*Aims*. This study aims to assess the use of spleen stiffness measurement using transient elastorgraphy in predicting varices in a heterogenous population of cirrhotic patients.

*Methods*. 47 consecutive cirrhotic patients underwent both liver (LSM) and spleen stiffness measurements. 31 of the 47 patients had upper endoscopy to assess for the presence of varices.

*Results*. Patients had a mean age of 60.9 years, BMI of 30.7, where 61%  & 39% were males and females, respectively. The mean LSM of all patients equaled 24.51 kPa, while mean SSM was 35.74 kPa.

Using a *t*-test analysis for LSM of 20.00 kPa, the best cut-off value for SSM was equivalent to 20.90 kPa. A SSM ≥ 20.90 kPa had a Sp = 1.00, Sn = 0.90, PPV = 0.87 in relation to presence of varices.

Using a one-way Anova test, a *P* = 0.000 was seen for patients with SSM greater than 20.90 kPa who also tested postive for the presence of varices. No statistical significance (*P* = 0.933) was seen within different disease groups for patients with spleen stiffness >20.90 kpa.

*Conclusions*. The power of spleen stiffness measurements using transient elastograhpy, lays in its non-invasive properties and strong ability in predicting clinically significant portal hypertension; more specifically varices. In our study, a liver stiffness value greater than 20.00 kPa (4–6), wasn't consistent in predicting presence of varices; where as a spleen stiffness ≥20.90 kPa was 100% sensitive for the presence of varices. Spleen stiffness in turn, will allow clinicians timely non-invasive screening and intervention for varices. A larger study assessing spleen stiffness based on disease etiology in association with precense of varices is planned in the near future.

*Funding Agencies: *None

# Sub article: A243: The Clinical Relevance of Liver to Spleen Stiffness Ratios versus Individual Spleen Stiffness Values in Predicting Clinically Significant Portal Hypertension in Cirrhotic Patients

**Authors:** K. Boctor, M. Magnes, M. Elkhashab

**Affiliations:** ^243.1^Toronto Liver CentreTorontoONCanada

*Background*. In our previous study utilizing a heterogeneous population consisting of 25% cirrhotic patients (1-2), we established that spleen stiffness measurement (SSM) equivalent to 19.45 kPa or greater was significant for the detection of developing clinically significant portal hypertension (CSPH). While, a liver to spleen stiffness (LSM : SSM) ratio had a specificity of .88 and sensitivity of 0.63 for detecting CSPH.

*Aims*. Our aim is to further explore these finding in a larger number of cirrhotic patients. We also evaluated the clinical significance of LSM : SSM ratios versus of individual SSM values in providing greater sensitivity, and specificity for the detection of CSPH.

*Methods*. 52 consecutive cirrhotic patients (Mean age 60.9/BMI 30.7) underwent both LSM & SSM (in Supine (S) & right lateral decubitus (RLD) positions) utilizing M-probe. Half of patients had proven portal hypertension in the form of varices +/− portal hypertensive gastropathy.

Patients were divided into 2 main groups; those in whom LSM were below, or above 20.0 kPa respectively (1-2). We then further divided patients with LSM > 20.0 kpa based on disease etiology: Chronic HBV (B), Chronic HCV (C), NAFLD (F), & Autoimmune hepatitis (A).

*Results*. 100% success rate was achieved with the following number of patients in each sub-group: B (12), C (15), F (14), A (11).

LSM : SSM ratio for all patients <20.0 kPa was 0.46. Ratios for patients with LSM < 20.0 kPa in supine and RLD respectively were: B (0.46, 0.59), C (0.39, 0.62), F (0.42, 0.40), A (0.35, 0.45). LSM : SSM ratio for all patients >20.0 kPa was 0.83. Ratios for patients with LSM > 20.0 kPa in supine and RLD respectively were: B (0.58, 0.94), C (0.37, 0.79), F (0.48, 0.77), A (0.39, 0.78).

The mean SSM in RLD position for all patients was 27.64 kPa with a Pr = 0.997. RLD-SSM means with groups were as follows: B (25.87), C (27.02), F (27.09), A (30.05).

The mean SSM in RLD & S positions for all patients with LSM < 20.0, are 14.8 kPa &35.7 kPa respectively.

The mean SSM in RLD & S positions for all patients with LSM > 20.0, are 34.9 kPa & 35.60 respectively.

Using *t*-test analysis for LSM of 20.0 kPa, the best cut-off value for RLD-SSM is equal to 20.90 kPa, yielding a SN = 0.77, and SP = 0.80.

*Conclusions*. A ratio of LSM : SSM equal to 0.83 and an individual SSM of 20.9 kPa or greater are both indicative of clinically significant portal hypertension; and therefore, of variceal formation. However, it appears that liver to spleen stiffness ratio is less effected by body position and disease etiology. Therefore, LSM : SSM offers a superior clinical picture as to the degree of spleen involvement and subsequently the degree of portal hypertension in cirrhotic patients. Further studies are needed to confirm these intriguing finding.

*Funding Agencies: *None

# Sub article: A244: Further Exploration of the Effect of Body Positioning on Spleen Stiffness in Cirrhotic Patients

**Authors:** K. Boctor, M. Magnes, M. Elkashab

**Affiliations:** ^244.1^Toronto Liver CentreTorontoONCanada

*Background*. In our previous study consisting of a heterogeneous population of cirrhotic and non-chirrhotic patients, our findings suggested that, spleen stiffness using transient elastography was less dependent on body position in cirrhotic patients (1).

*Aims*. We sought to analyze the effect on position in patients with varying degrees of cirrhosis using transient elastography both in supine (S), and right lateral decubitus (RLD) positions in a larger group of cirrhotic patients. We further explored the effect of liver disease on spleen stiffness measurement (SSM) in cirrhotic patients.

*Methods*. 132 consecutive tests were conducted on cirrhotic patients (Mean age 60.9/BMI 30.7) underwent both LSM & SSM (S and RLD positions) utilizing transient elastography. 50% of patients had proven portal hypertension in the form of varices +/− Portal hypertensive gasteropathy.

A liver stiffness measurement (LSM) of 20.0 kPa was deemed clinically significant for the prediction of portal hypertension (2-3).

Patients were divided into 2 main groups; those in whom LSM were below, or above 20.0 kPa respectively. We then further divided patients with LSM > 20.0 kpa based on disease etiology: Chronic HBV (B), Chronic HCV (C), NAFLD (F), & Autoimmune hepatitis (A).

*Results*. 100% success rate was achieved with 21 patients with LSM < 20.0 kPa (Group I) & 31 patients yielding a LSM > 20.0 kPa (Group II). 78.8% of SSM completed in supine, 78.8% of SSM completed in RLD.

In the S-position, groups I & II had a mean SSM of 35.60 kPa & 35.70 kPa, respectively; Pr = 0.988, showing no statistical significance.

In the RLD-position, groups I & II had a mean SSM of 14.80 kPa & 34.90 kPa, respectively; Pr = 0.0003; showing a strong statistical significance.

In group I, SSM measured (kPa) in S and RLD-positions were as follows respectively: B (36.31, 19.19), C (14.13, 15.28), F (43.74, 11.08) & A (54.56, 12.82).

In group II, SSM measured (kPa) in S and RLD-positions were as follows respectively: B (34.40, 32.21), C (26.79, 35.54), F (31.91, 35.00) & A (55.73, 36.93).

Using *t*-test analysis for LSM of 20.00 kPa, the best cut-off value for RLD-SSM is equal to 20.90 kPa, yielding a SN = 0.77, and SP = 0.80.

*Conclusions*. Our findings suggest that spleen stiffness is dependent on the degree of cirrhosis (LSM > 20.0), as well as, liver disease etiology. RLD position is both more specific and sensitive in predicting increased LSM; thereby making it the preferred position for the prediction of clinically significant portal hypertension in cirrhotic patients.

*Funding Agencies: *None

# Sub article: A245: Health Related Quality of Life in Ten Year Survivors of Paediatric Liver Transplantation Measured by the Peltql: A Novel Disease-Specific Questionnaire

**Authors:** M. Miserachs, A. Otley, A. Dhawan, J. Bucuvalas, S. Gilmour, M. Stormon, L. Ee, V. Ng

**Affiliations:** ^245.1^The Hospital for Sick ChildrenTorontoONCanada; ^245.2^Dalhousie UniversityHalifaxNSCanada; ^245.3^3King's College HospitalLondonUK; ^245.4^Cincinnati Children's HospitalCincinnatiOHUSA; ^245.5^University of AlbertaEdmontonABCanada; ^245.6^The Children's Hospital at WestmeadStdneyNSWAustralia; ^245.7^7Royal Children's HospitalBrisbaneQLDAustralia

*Background*. Less than 1/3 of patients alive 10 years after paediatric liver transplantation (LT) in the Studies of Paediatric Liver Transplant (SPLIT) database fulfilled a research composite definition of an “ideal ten-year survivor”. Missing within this composite profile were patient-reported subjective outcome variables such as Health Related Quality of Life (HRQOL) and Mental Health.

*Aims*. To compare outcomes of HRQOL and Mental Health between ideal 10 year survivors and non-ideal survivors.

*Methods*. This was an international multi-center cross-sectional analysis characterizing patients who have survived >10 years from LT enrolled in the Paediatric Liver Transplant Quality of Life (PeLTQL) Study Group database. Subjects were categorized as ideal survivors if a “yes” answer was obtained from all 13 historically, clinically, and biochemically obtainable variables. HRQOL was assessed with three well-validated tools: The PeLTQL, PedsQL TM and PedsQL. Data from completed Screen for Child Anxiety Related Disorders (SCARED) scales and the Children's Depression Inventory Short Form (CDI-S) were also reviewed.

*Results*. A total of *N* = 57 (56% female, median patient age 14, range 11–18 years) subjects were reviewed, with 13 (22%) identified as an “ideal survivor". Total PeLTQL scores were not significantly different between ideal (median 68.8, range 52.8–88.4) and non-ideal (median 69.6, range 27.9–96.1, *p* = 0.8) survivors. The generic PedsQL scores were also not significantly different between ideal (median 79.4, range 28–90) and non-ideal (median 83.7, range 9–99, *p* = 0.4) survivors. While there were no significant differences in SCARED (anxiety) or CDI-S (depression) scores between ideal and non-ideal survivors, SCARED (anxiety) scores above the established clinical cut-scores were found in 6/12 (50%) ideal survivors compared to 12/44 (27%) in non-ideal survivors. In addition, higher CDI-S (depression) scores above the clinical established cut score were found in 2/13 (15%) ideal survivors compared to 5/44 (11%) non-ideal survivors.

*Conclusions*. Amongst subjects meeting the recently proposed “ideal survivor" profile, HRQOL assessment was not significantly better in ideal survivors compared to non-ideal survivors. Attention to the risk for anxiety remains an important finding for the long-term survivor of paediatric LT.

*Funding Agencies: *None

# Sub article: A246: Markedly Elevated Serum Alpha-Fetoprotein Levels Not Caused by Hepatic Malignancy in Two Infants with End Stage Liver Disease—A Case Series

**Authors:** E. Crowley, T. Gerstle, F. Shaikh, M. Greer, V. Ng

**Affiliations:** ^246.1^Division of GastroenterologyHepatology and NutritionHospital for Sick Children—SickKidsTorontoONCanada; ^246.2^Division of General SurgeryHospital for Sick ChildrenTorontoONCanada; ^246.3^Division of Hematology/OncologyHospital for Sick ChildrenTorontoONCanada; ^246.4^Department of Diagnostic ImagingHospital for Sick ChildrenTorontoONCanada

*Background*. Alpha Fetoprotein (AFP) is a classical tumor marker for epithelial liver tumours. However, when elevated in a pre liver transplant patient and a true source for malignancy cannot be sourced, they pose a diagnostic dilemma and a therapeutic challenge.

*Aims*. To describe the clinical course of two infants with markedly elevated serum AFP levels who underwent successful liver transplantation after negative extensive investigations for presumed hepatic malignancy.

*Methods*. This case series with systematic literature review was approved by the Research Ethics Board at SickKids. A retrospective chart review of the electronic medical records was undertaken.

*Results*. Infant A, Asian term female, presented with persistent neonatal cholestasis at 4 months. Expedited liver biopsy revealed biliary atresia. The infant was referred for liver transplant assessment. A hepatic lesion was noted on ultrasound amidst a grossly cirrhotic liver. The AFP peaked at 91,621 mcg/L (normal <275 mcg/L). Such marked elevation in AFP levels prompted extensive radiological investigations. Consultations were sought, culminating with consensus discussion at Surgery-Pathology-Radiology multidisciplinary meetings. At time of explant, histopathological analysis revealed no areas suspicious for malignancy.

Infant B, 3 month old term Asian male, presented with persistent cholestasis and synthetic liver dysfunction. A liver biopsy and intraoperative cholangiogram demonstrated clear opacification of the duodenum and biliary tree. The infant was referred to the Liver Transplantation Program due to deteriorating liver function. During evaluation, his AFP increased from 39,396 mcg/L to 156,406 mcg/L peaking to 618,000 mcg/L. A peripancreatic/porta hepatis mass was noted on CT and he was suspended on the liver transplant list. The clear natural history of his disease would have led to death. Extensive investigations and consultations were performed to evaluate for malignancy. The cause for the elevated AFP in the setting of end stage cholestatic liver disease cannot be explained.

*Conclusions*. As per the American Association Study of Liver Diseases liver transplant guideline, hepatocellular carcinoma is not a contraindication to transplant. However, extrahepatic disease is an absolute contraindication. The subsequent evaluation of an increasing serum AFP titre in a potential liver transplant recipient may delay the procedure while an explanation is sought.

*Funding Agencies: *None

# Sub article: A247: Concentrations of 6-Thioguanine Nucleotide Correlate with Infliximab and Adalimumab Levels in Patients with Inflammatory Bowel Disease on Combination Therapy

**Authors:** A. Trajkovski, O. Kelly, A. Weizman, G. Nguyen, H. Steinhart, M. Silverberg, K. Croitoru

**Affiliations:** ^247.1^Mount Sinai HospitalTorontoONCanada; ^247.2^Mount Sinai HospitalUniversity of TorontoTorontoONCanada

*Background*. Combining immunomodulators with anti-TNF (tumour necrosis alpha) therapy in Inflammatory Bowel Disease (IBD) is associated with higher drug levels and mucosal healing. Optimal thiopurine dosing remains unclear. Recent data suggest correlation between 6-Thioguanine (6TG) and infliximab (IFX) levels.

*Aims*. We examined if this correlation extended to Adalimumab (ADA) levels and associations with mucosal healing.

*Methods*. A cross-sectional study of IBD patients receiving combination therapy (IFX/ADA + thiopurine) was performed. Patients with simultaneous anti-TNF level/antibody (ADA/IFX; Prometheus assay) and thiopurine metabolite testing (6TG + MMP) were included. Endoscopic remission was defined as Mayo <1 (Ulcerative colitis; UC) and SESCD < 3 (Crohn's disease; CD). Primary outcomes were anti-TNF level and antibody to anti-TNF and were analyzed as continuous and dichotomized variables with calculated IFX/ADA cutoff values best predicting mucosal healing. 6TG levels were examined as continuous and in quartile distribution (1: 0–124; 2: 125–250; 3: 251–400; 4: >400 pmol/8 × 10^8^ RBC).

*Results*. 64 patients on combination were included (34 ADA, 30 IFX). Gender, age, phenotype and treatment duration were not significantly different between groups. ADA group had greater endoscopic activity (70% versus 55%, *p* = 0.01). Mean 6TG (326, *p* = 0.84) and MMP levels (1572, *p* = 0.07) were similar. Mean IFX and ADA levels were higher in combination therapy compared to a monotherapy cohort (12.3 versus 10.2 *μ*g/mL IFX, *p* = 0.04, 11.4 versus 9.8 *μ*g/mL ADA, *p* = 0.04). There was a trend toward lower antibodies (2.6 versus 7.9 U/mL IFX, 3.5 versus 6 U/mL ADA) compared to monotherapy (*p* = 0.26, 0.35). Higher anti-TNF levels were associated with endoscopic remission. Anti-TNF levels inversely correlated with Mayo score (*r* = −0.69, *p* = 0.008, AUC 0.6 [0.46–0.83]) and SESCD (*r* = −0.4, *p* = 0.039, AUC 0.64 [0.48–0.83]). 6TG levels were associated with higher anti-TNF levels. Based on ROC, using a cutoff IFX >7.6 (sens. 82% spec. 62%), 6TG >125 was associated with attaining clinically adequate IFX levels (*p* = 0.001, *r* = 0.49, Fishers exact *p* = 0.018). Using a cut off of >6.6, (sens. 83%, spec. 54%), 6TG >125 correlated with clinically adequate ADA levels (*r* = 0.58, *p* = 0.001, Fishers exact *p* = 0.009). 6TG values in quartiles(Q)2 and 3 (125–400) were associated with therapeutic anti-TNF levels (*p* = 0.001) and endoscopic healing (*p* = 0.001). Antibody presence was associated with endoscopic activity (*p* = 0.05). 6TG levels in Q2 and 3 had fewer antibodies but did not reach significance.

*Conclusions*. 6TG levels >125 pmol/8 × 10^8^ RBC correlate with therapeutic IFX/ADA levels and endoscopic remission.

*Funding Agencies: *CAG, CIHR, Prometheus Laboratories provided Infliximab and Adalimumab level and antibody testing

# Sub article: A248: Ultrasound Shear Wave Elastography and Contrast Enhancement Biomarkers in Crohn's Disease Strictures

**Authors:** C. Lu, X. Gui, W. Chen, K. Novak, S. Ghosh, S. Wilson

**Affiliations:** ^248.1^University of AlbertaEdmontonABCanada; ^248.2^University of CalgaryCalgaryABCanada; ^248.3^University of CalgaryCalgary ABABCanada

*Background*. Inflammation and fibrosis lead to thickened and narrowed bowel in Crohn's Disease (CD) as detected on ultrasound (US). US shear wave elastography (SWE) assesses tissue elastic properties providing a quantitative measurement of stiffness. SWE differentiates inflammation from fibrosis in ex vivo CD bowel. Neo-angiogenesis is a vital component of bowel wall inflammation. Quantification of bowel wall vascular perfusion and inflammation is uniquely detected by contrast enhanced ultrasound (CEUS).

*Aims*. We aim to prospectively correlate SWE of ileal CD in-vivo to CEUS peak enhancement (PE), and to pathology grades of inflammation, fibrosis, and muscular hypertrophy of resected bowel. We predict patients with higher SWE score will have greater bowel stiffness attributable to fibrosis and muscular hypertrophy, and concurrent chronic inflammation.

*Methods*. 105 consecutive CD patients (Mar to Oct 2014) attending outpatient US appointments received greyscale US. In patients with ileal CD and bowel wall thickness (BWT) > 4 millimetres, SWE and PE using microbubble (Definity®) contrast was measured at point of maximal BWT (*n* = 95). An average of ten SWE readings were collected; Virtual Touch Quantification; Acuson S3000, Siemens Medical Solutions USA, Inc or with ElastPQ, Philips Epiq 5 (Bothell, WA). Analysis of variance compared PE and mean SWE with histological grades. Fifteen patients had ileal resections within an average of 71.0 ± 66.9 days from time of US. Two gastrointestinal pathologists scored specimens for inflammation, fibrosis, and muscular hypertrophy for comparison to SWE measurements.

*Results*. In fifteen ileal specimens, chronic inflammatory histological change exceed active inflammation (*p* < 0.001). Mean in-vivo SWE measurements for patients who had and did not have surgery were 2.8 ± 0.7 m/s and 2.2 ± 0.8 m/s (*p* < 0.01), respectively. Of the operated patients, there was an inverse relationship between PE and both fibrosis, *r* = −0.59, *p* = 0.02, and SWE, *r* = −0.61, *p* = 0.03. Strictured bowel specimens had more smooth muscle hypertrophy than fibrosis, *p* < 0.001. There was a correlation between SWE and muscular hypertrophy, *r* = 0.59, *p* = 0.02 and no significant relationship with SWE and fibrosis scores (*p* > 0.05).

*Conclusions*. SWE of small bowel CD increases when there is stiffer bowel attributed to smooth muscle hypertrophy. Overall, CD patients with lower CEUS PE parameters have more chronic bowel inflammation. A novel observation; higher SWE is correlated with bowel smooth muscle hypertrophy, and is inversely related to PE, providing possible differentiation between active and chronic bowel inflammation to improve selection between medical therapy and surgery.

*Funding Agencies: *None

# Sub article: A249: Efficacy of Hepatitis B Counseling and Vaccination in Patients with Inflammatatory Bowel Disease Receiving Anti-TNF Therapy

**Authors:** T. Dang, C. Lu, R. Lumb, S. Krahn, K. Kroeker, R. Fedorak

**Affiliations:** ^249.1^University of AlbertaEdmontonABCanada; ^249.2^Los Alamos National LaboratoryEdmontonABCanada

*Background*. Immunosuppressive therapies are considered a cornerstone of inflammatory bowel disease (IBD) treatment but carry increased risk of infections. Hepatitis B virus (HBV) screening and vaccination is recommended in all IBD patients prior to initiation of anti-TNF therapy. Despite recommendations, studies indicate less than optimal screening for HBV in the IBD population.

*Aims*. (1) Determine the proportion of IBD patients on anti-TNF therapy who have been appropriately screened and vaccinated against HBV. (2) Measure the immune response to HBV vaccinations for non-immune patients receiving anti-TNF therapy.

*Methods*. Patients receiving infliximab or adalimumab were enrolled in this retrospective cohort study. Patient demographics, IBD characteristics, HBV vaccination history, and treatment history were obtained through a mail-out survey and chart review. HBV serology was obtained to determine vaccination status. Adequate protection against HBV was defined as HBs-Ab > 10 U/L. Non-immune patients were contacted for follow-up for HBV vaccinations and given a prescription for a standard series of HBV vaccination. HBV serology was re-measured at 3 months post-vaccination to determine response to vaccination.

*Results*. This study is ongoing; to date 250 patients on anti-TNF therapy have been enrolled. Of these patients (mean age of 38 years), only 39.8% of patients recall receiving their childhood vaccinations and 27.8% report receiving adult HBV boosters. Very few patients (1.2%) report having HBV vaccination counseling with similarly few non-immune patients receiveing vaccinations (2.5%) prior to initiation of anti-TNF therapy. Only 43.6% of patients had HBV serology checked prior to initiation of anti-TNF therapy and only 31.6% were known to be adequately protected against HBV. When this was sub-divided into patients who were initiated on anti-TNF prior to 2010 (before HBV screening and vaccination was widely recommended), only 9.38% of patients had HBV serology checked compared to 42.2% (*p* value < 0.001) of patients initiated after 2010. Fewer patients who were initiated pre-2010 were found to be adequately protected against HBV compared to those initiated post-2010 (15.6% versus 37.2%, *p* value = 0.04).

*Conclusions*. HBV screening and vaccination is currently recommended in all IBD patients. However, there is a lack of counseling perceived by patients regarding HBV vaccinations. Although rates of appropriate HBV screening prior to initiation of anti-TNF is improving, there remains a considerable proportion of patients whose HBV status remains unknown. The second phase of our study will investigate the immune response of IBD patients receiving anti-TNF to the standard HBV vaccinations.

*Funding Agencies: *None

# Sub article: A250: The Fc Gamma Receptor IIA Gene Variant Increases Macrophage Inflammatory Responses and Reduces Antibody-Mediated Anti-Inflammtory Macrophage Activation

**Authors:** L. Kozicky, Z. Zhao, S. Menzies, K. Harnden, L. Sly

**Affiliations:** ^250.1^UBCVancouverBCCanada

*Background*. Inflammatory bowel disease (IBD) is a chronic immune-mediated disease characterized by inflammation along the gastrointestinal tract. A gene variant in the Fc*γ*RIIa predisposes people to develop IBD and has been linked to a failure to respond to anti-TNF*α* therapy. The Fc*γ*RIIa susceptibility SNP changes the receptor from a low to a high affinity receptor but its role in IBD remains unknown.

Antibody-based biological therapies (anti-TNFa antibodies) have revolutionized treatment for IBD but 10% of patients do not response to anti-TNF*α* therapy. Macrophages are key players in the inflammatory response and contribute to inflammation in IBD. However, macrophages have non-specific antibody receptors, which activate macrophages to produce large amounts of the anti-inflammatory cytokine, IL-10, in response to inflammatory stimuli.

*Aims*. Based on this,* we hypothesize that* the Fc*γ*RIIa susceptibility SNP predisposes people to develop IBD and fail anti-TNF*α* therapy by inhibiting anti-inflammatory Fc*γ*R macrophage activation. To address this hypothesis, we will determine:

*Aim  1*. Which Fc*γ*Rs activate anti-inflammatory macrophage responses.

*Aim  2*. The effects of Fc*γ*RIIa gene variants on innate inflammatory responses.

*Aim  3*. The effects of Fc*γ*RIIa gene variants on antibody-mediated macrophage activation.

*Methods*. Peripheral blood monocyte-derived macrophages (MDMs) were prepared from healthy control subjects. MDMs were stimulated with lipopolysaccharide (LPS), pooled IgGs, or both, in the presence or absence of blocking antibodies to Fc*γ*Rs. Subjects were genotyped for the Fc*γ*RIIa gene variant. MDMs from subjects were stimulated with LPS or LPS + IgGs. Cell supernatants were assayed for cytokines by ELISA.

*Results*. Fc*γ*RI and Fc*γ*RIIb/c were required for antibody-mediated IL-10 production in LPS-stimulated macrophages. Subjects with the high affinity Fc*γ*RIIa produced more pro-inflammatory cytokines in response to LPS, and produced less IL-10 and more pro-inflammatory cytokines in response to LPS + antibody compared to subjects, who do not have the gene variant.

*Conclusions*. Multiple Fc*γ*Rs are required for limiting immune responses. Macrophages from subjects with the Fc*γ*RIIa susceptibility variant produce more inflammatory cytokines in response to stimuli and are less effective at reducing pro-inflammatory cytokine production when treated with antibody. These data are consistent with a model in which the high affinity Fc*γ*RIIa susceptibility variant sequesters antibody from the Fc*γ*Rs that mediate anti-inflammatory macrophage activation. Future studies will investigate whether the Fc*γ*R susceptibility gene variant can be used to predict whether a patient will fail to respond to anti-TNF*α* therapy.

*Funding Agencies: *CAG

# Sub article: A251: Safety of Anticoagulation in Non-Hospitalized IBD Patients

**Authors:** I. Plener, A. Rumman, M. Cino, G. Nguyen

**Affiliations:** ^251.1^University of TorontoTorontoONCanada; ^251.2^University of TorontoUniversity Hospital NetworkTorontoONCanada; ^251.3^Mount Sinai HospitalUniversity of TorontoTorontoONCanada

*Background*. Patients with IBD have a 3-4 fold increased risk of venous thromboembolic disease (VTE), up to 16-fold higher during periods of moderate-severe disease. Due to concomitant gastrointestinal bleeding there are concerns regarding anticoagulation safety. Currently, there are no consensus statements addressing VTE prophylaxis during outpatient IBD flares.

*Aims*. To characterize the rates of major and minor bleeding in non-hospitalized IBD patients on anticoagulation. Secondary aims to assess efficacy and safety of anticoagulation and VTE recurrence.

*Methods*. Retrospective study evaluating patients, over 18 years old, with UC and CD. All patients initiated on anticoagulation for VTE were included. Primary endpoint included major and minor bleeding episodes^*^. Secondary endpoints included mortality due to bleeding, transfusions and recurrent thrombosis. The frequency and distribution of study variables was determined using descriptive analyses. Categorical data were compared using the chi-square statistic. Cumulative person-time incidence rates of major and minor bleeding were calculated.

*Results*. Fifty-eight patients included. Median duration of anticoagulation therapy was 19.0 months (IQR 8.0–45.0). In patients on LMWH bridging, median treatment was 6.1 months (IQR 2.0–9.1). A total of 2475 person-months of anticoagulation therapy studied. 1 major and 8 minor bleeding episodes recorded. Of those, 2 were perioperative. The rate of minor bleeding events was 3.88 events per 100 patient-years of anti-coagulation therapy (95% CI 1.8–7.37). The rate of major bleeding was 0.485 events per 100 patient-years of anti-coagulation therapy (95% CI 0.024–2.39). The major bleeding event occurred in the setting of severe UC requiring colectomy. No mortality was reported. A total of 6 recurrent thrombotic events were detected. Rate of recurrent VTE: 3.03 events per 100 person-years of anticoagulation therapy (95% CI 1.23–6.30).

*Conclusions*. Our data suggests that ambulatory IBD patients are at similar risk of major or minor bleeding compared to the general population. Incidence of minor bleeding in non-atrial fibrillation is reported to be 2.84 to 3.71 in NOACs, and 4.10 per 100 patient years on warfarin. In IBD patients who did experience minor bleeding, small dose adjustments or careful monitoring were implemented. Up to 40% of patients had active disease at the time of thrombosis, highlighting the known increased risk of VTE in IBD patients. This study highlights the safety of anticoagulation in the outpatient setting and the importance of its use in moderate-severe IBD flares in ambulatory patients.

*Funding Agencies: *None

# Sub article: A252: Activation of the Constitutive Androstane Receptor Enhances Intestinal Epithelial Wound Healing and Accelerates Recovery from Experimental Colitis

**Authors:** G. Hudson, A. Zamponi, L. Alston, A. Zhao, T. Chang, S. Hirota

**Affiliations:** ^252.1^University of CalgaryCalgaryABCanada; ^252.2^University of British ColumbiaVancouverBCCanada

*Background*. Compounds released from the intestinal microbiota play a role in maintaining mucosal homeostasis, but little is known about how they are sensed by the gut. Recently the pregnane X receptor (PXR) was identified as a receptor for microbial metabolites and shown to play a key role in regulating intestinal epithelial barrier and wound healing following mucosal injury. In this study, we sought to determine whether the constitutive androstane receptor (CAR), which shares similar structural, functional and pharmacological features with the PXR, plays a role in regulating wound healing.

*Aims*. We sought to assess whether the CAR regulates the wound healing process and determine its role in an in vivo model of intestinal mucosal healing.

*Methods*. Caco-2 intestinal epithelial cells were used to model wound healing. Wounds formed using Ibidi inserts were treated with CITCO, a selective CAR agonist, and imaged for 24 hours. To interrogate the intracellular events involved in the CITCO-induced responses, wound healing assays were performed in the presence of agents to inhibit p38 and ERK1/2 signaling. CAR-induced cell proliferation and migration were also assessed via CITCO treated Caco-2 monolayers. The role of the CAR was also examined in a mouse model of mucosal damage and repair, wherein C57/Bl6 mice were treated with dextran sulphate sodium (DSS) for five days with seven days recovery while receiving daily dosing with rodent specific CAR agonist TCPOBOP (3 mg/kg daily, PO). Prior to sacrifice, intestinal permeability was quantified. Tissues were removed for histological assessment and MPO assay.

*Results*. Activation of the CAR significantly enhanced wound closure in Caco-2 monolayers. This effect was associated with increased cell migration, with minimal effects on cell proliferation. At concentrations that enhanced wound closure, CITCO increased p38 MAP kinase activation. Additionally, inhibition of p38 MAP kinase signaling significantly reduced CITCO-induced enhancement of wound closure. Finally, treating mice with rodent specific CAR agonist, TCPOBOP, enhanced recovery following exposure to DSS, attenuating the intestinal barrier dysfunction and histological damage.

*Conclusions*. Our data suggest that the CAR regulates the cellular events that contribute to wound healing. While the microbe-derived compounds that activate the CAR have yet to be identified, its close relative, the PXR, has been shown to respond to such ligands. Taken together, we posit that xenobiotic receptors play a key role in regulating the response to injury and may provide new therapeutic targets to enhance mucosal repair.

*Funding Agencies: *CCC, Canadian Foundation for Innovation; Dr. Lloyd Sutherland Investigatorship in IBD/GI Research

# Sub article: A253: ER Stress Inhibits Mitochondrial Stress-Induced Loss of Barrier Function via Autophagy

**Authors:** F. Lopes, A. Wang, J. Reyes, R. van Dalen, D. Philpott, D. McKay

**Affiliations:** ^253.1^University of CalgaryCalgaryABCanada; ^253.2^University of TorontoTorontoONCanada; ^253.3^Uni. CalgaryCalgaryABCanada

*Background*. Genetic susceptibility traits, such as mutations in genes that control autophagy, and inappropriate reactions against a component of the commensal microbiota are key elements in the development of IBD. We recently showed that perturbed mitochondrial function results in increased transcytosis of commensal bacteria across a model gut-derived epithelium in vitro. Given the relationship between mitochondria, the endoplasmic reticulum (ER) and autophagy, we sought to assess the role of concomitant ER and metabolic stress on epithelial permeability.

*Aims*. To couple ER stress (i.e., an unfolded protein response (UPR)) with exposure to an inhibitor of mitochondrial activity in normal epithelial cells and those with a genetic susceptibility trait, that is, loss of function of the Autophagy-related Protein 16-L1 (ATG16L1) gene, and determine the impact of this on barrier function.

*Methods*. T84 and HCT116 epithelial cells, normal or ATG16L1 knocked-down (by siRNA and CRISPR-cas9 resp.), were co-treated with dinitrophenol (DNP, uncouples oxidative phosphorylation) and tunicamycin (TU) (an ER stressor). Barrier function was assessed by (1) transepithelial electrical resistance (TER) and FITC-dextran translocation (paracellular pathway) and internalization of inert fluorescent beads and viable* E*.* coli* (HB101) and bacteria translocation (transcellular permeability). The UPR and autophagy were measured by Western Blot. Mouse colonoids were cultured to analyze mechanisms in non-modified cells.

*Results*. TU induced an UPR within 8 h that was marked at 16 h post-treatment, but in contrast to DNP, it did not affect epithelial barrier function. When epithelial cells were simultaneously treated with DNP + TU, the DNP-induced increase in epithelial paracellular permeability was unaffected. However, DNP-induced increased intracellular viable* E*.* coli* and translocation of bacteria was blocked by TU, which was paralleled by increased expression of markers of autophagy in T84 epithelia. Epithelia treated with, rapamycin (induces autophagy) did not affect DNP-induced internalization or inert beads into T84 cells but did reduce the number of viable intracellular bacteria; TU treatment of epithelia lacking ATG16L1 displayed increased viable bacteria in DNP-cotreated cells. Finally, TU also induced autophagy in mouse colonoids.

*Conclusions*. While ER stress has been implicated in the pathophysiology of colitis, we find that an unfolded protein response can protect against mitochondrial stress-induced loss of barrier function via up-regulated autophagy, which likely mediates killing of internalized bacteria.

*Funding Agencies: *CIHR

# Sub article: A254: Knowledge, Perceptions, and Attitudes Towards Medication Adherence and Pregnancy in Inflammatory Bowel Disease

**Authors:** Z. Gallinger, A. Rumman, G. Nguyen

**Affiliations:** ^254.1^University of TorontoTorontoONCanada; ^254.2^Mount Sinai HospitalUniversity of TorontoTorontoONCanada

*Background*. When considering pregnancy, women of childbearing age with inflammatory bowel disease (IBD) often have to balance the risks and benefit of their IBD medications against the potential for active disease. Fortunately, women with quiescent disease can expect to have a pregnancy with similar outcomes to the general population. With the exception of methotrexate, thalidomide, and cylcosporine, the majority of commonly used medications appear to be safe to use during pregnancy. Still, survey studies of IBD cohorts have shown higher rates of “voluntary childlessness” in patients with IBD compared to the regular population. Limited data exists on medication adherence in pregnant women with IBD.

*Aims*. This study assessed which factors contribute to medication adherence during pregnancy in women with IBD. We also attempted to evaluate the thoughts processes of female IBD patients when faced with the decision of taking potentially teratogenic medications, compared with stopping or switching to medications with less potential for adverse effects.

*Methods*. Female patients completed a self-administered, structured survey. We collected demographic data, medication history, and self-reported adherence to IBD medications during pregnancy. We assessed knowledge and perceptions of IBD medication safety in pregnancy. A time trade-off (TTO) analysis was done to assess health utilities for continuing or discontinuing IBD medications during pregnancy.

*Results*. A total of 204 women completed the survey (mean age was 32.8 years). Current or previous pregnancy was reported by 101 patients (median parity 2, median gravity 1). While pregnant, 42 (41.6%) participants reported stopping a prescribed IBD medications. Of those, seventeen participants (40.5%) reported stopping medications without the advice of a physician. Participants with current or previous pregnancy were less likely to routinely rely on the internet (35.6% versus 51.5%, *p* < 0.01) and on family and friends (4.0% versus 45.6%, *p* < 0.001) for medication safety information. They were also less likely to be non-compliant with IBD medications during pregnancy to avoid possible harm to the fetus (26.7% versus 43.7%, *p* < 0.001). TTO analysis was completed by 31 patients. When presented with the option of continuing a potentially teratogenic medication, switching to less effective medication that is non-teratogenic or stopping medication all together, participants consistently preferred to switch (Figure 30).

*Conclusions*. Women with IBD report significant non-adherence to medications during pregnancy. This is driven by concerns about safety and uncertainty about teratogenic effects. Programs should focus on increasing education surrounding medication safety in pregnancy.

*Funding Agencies: *None

# Sub article: A255: Critical Role of Chromogranin-A in the Context of Colorectal Inflammation: Human and Animal Studies

**Authors:** N. Eissa, M. Rabbi, R. Mesgna, A. Khafipour, C. Bernstein, J. Ghia

**Affiliations:** ^255.1^ImmunologyInternal Medicine section of GastroenterologyUniversity of ManitobaWinnipegMBCanada

*Background*. The use of murine models of colitis and a limited number of clinical observations suggest that Chromogranin-A (CgA), released by the enterochromaffin cells plays a critical role in the development of colonic inflammation. CgA is elevated in inflammatory bowel disease (IBD) patients and macrophages dysregulated. Having demonstrated that a peptide derived from-CgA, Catestatin, can supress colitis and regulate the macrophages, we hypothesized that (i) in human rectal biopsies the level of CgA is correlated with inflammatory markers and (ii) in an animal model of colitis, the lack of CgA would modify the course of the colitis.

*Aims*. To determine the expression of CgA and its correlation with pro and anti-inflammatory markers in rectal biopsies from IBD and healthy individuals and to investigate the effect of the lack of CgA using an experimental model of ulcerative colitis and on the alternatively activated (AAM) and classically activated macrophages (CAM)s.

*Methods*. Rectal human biopsies were collected, the correlation between CgA mRNA level with TNF-*α*, IL-6, IL-1*β*,* MCP1* and IL-10 mRNA levels were determined. In parallel, colitis was induced in CgA-C57BL/6-deficient (CgA^−/−^) & wild type (CgA^+/+^) mice by administration of dextran sulfate sodium (DSS 5%, 5 days). Disease activity index (DAI), macro- and microscopic scores were evaluated. Myeloperoxidase (MPO) activity, CgA, TNF-*α*, IL-6, IL-1*β*, CAMs markers (MCP1, iNOS, MIP-1*α*, MIP-1*β*), IL-10, and AAMs markers (Arg-1, YM1) were quantified in colon using ELISA/RT-qPCR. In-vitro, peritoneal macrophages isolated from naïve CgA^−/−^ and WT mice were exposed to IL-4/13 (20 ng/mL) or LPS (100 ng/mL) for 6 h to polarize AAMs and CAMs; AAMs, CAMs markers and cytokines were determined by ELISA/RT-qPCR.

*Results*. In human rectal biopsies, CgA demonstrated a significant positive linear correlation with TNF-*α*, IL-6, IL-1*β*, MCP1 and a significant negative linear correlation with, IL-10. DAI, macro- microscopic scores, colonic* MPO* activity, TNF-*α*, IL-6, IL-1*β* and CAMs markers were significantly decreased in CgA^−/−^ mice, conversely IL-10 and AAMs markers were increased. In vitro, expression of IL-1*β*, and CAMs markers were decreased, and IL-10, AAMs markers were increased in polarized macrophages isolated from CgA^−/−^ mice.

*Conclusions*. CgA signalling is critical in the pathogenesis of colonic inflammation. In human clinical biopsies, a linear relation depending on the nature of the inflammatory markers was determined. While, in experimental colitis, the lack of CgA increased the expression of AAMs markers and suppressed CAMs markers. These findings reveal new insights into the mechanisms of colonic inflammation, which may ultimately lead to novel therapeutic strategies in human IBD.

*Funding Agencies: *CCC, CIHR

# Sub article: A256: Systematic Functional Screens of Genes from IBD-Associated Loci in IBD-Relevant Cell Based Models

**Authors:** P. Goyette, G. Boucher, J. Paquette, N. Morin, A. Alikashani, M. Burnette, H. Gosselin, C. Beauchamp, L. Thauvette, M. Rivard, G. Lavallée, F. Dupuis, G. Charron, J. Rioux

**Affiliations:** ^256.1^Montreal Heart InstituteMontrealQCCanada; ^256.2^Université de Montréal and Montreal Heart InstituteMontrealQCCanada

*Background*. Recent genome-wide association studies in inflammatory bowel diseases (IBD) have identified ~200 genomic regions involved in disease risk. While some of these associations define regions with only one or few genes, or with a strong functional candidate gene, the majority contains many genes, making the identification of causal genes difficult. Follow-up fine mapping and sequencing studies have begun to dissect out the causal genes/variants in some regions, while focused functional studies have identified a number of important biological pathways that are involved in disease pathogenesis, but this essential information remains limited to only a handful of risk loci. To unravel disease mechanisms, it is critical to study the genes within newly discovered IBD regions in a cell-specific context. We believe that the majority of disease-causative genes located within IBD susceptibility loci are involved in a limited set of key biological pathways.

*Aims*. To address this, we have undertaken systematic functional screens of genes across the entire set of IBD loci in key cell line models for IBD (intestinal epithelial and immune cell lines) in order to decipher their functional roles and obtain a broader understanding of the key biological pathways involved IBD pathogenesis.

*Methods*. We have used a stable lentiviral open reading frame (ORF) overexpression model to screen all IBD loci genes in relevant cell models (HEK293, HT29, Jurkat, THP-1) and performed gene expression profiling.

*Results*. A strong correlation in expression profiles was observed from the independent infections of each ORFs highlighting the robustness of the experimental approach. Our data also supports previously observed effects of specific target ORFs, such as the induction of expected gene targets for the transcription factor HNF4A in the intestinal epithelial HT29 cell line and the previously observed opposing impact of the SMAD3 and SMAD7 molecules. Using expression profiling data, in combination with different in silico analytic methods (GSEA, pathway/function enrichment, TFBS enrichment) we aim to define common biological pathways involved in disease pathogenesis.

*Conclusions*. As with other broad scale approaches, our analysis is undoubtedly imperfect, however we believe that using a systematic approach in IBD-relevant cell types can provide an important window to better understanding IBD. The novel biological pathways identified through this study should help the future development of novel targeted molecules with therapeutic potential.

*Funding Agencies: *CCC, CIHR, Génome Canada, Génome Québec

# Sub article: A257: Telephone versus Clinic Folow-Up in Management of Pateints with Inflammatory Bowel Disease

**Authors:** U. Chauhan, D. Armstrong, S. Halder, J. Marshall, F. Tse, M. Pinto Sanchez, Y. Farbod, J. Popov, S. Kaasalainen, P. Moayyedi

**Affiliations:** ^257.1^McMaster University Medical CentreHamiltonONCanada; ^257.2^McMaster UniversityHamiltonONCanada

*Background*. Management of Inflammatory Bowel Disease (IBD) requires frequent contact with health care providers, but clinic visits impose an economic burden and can cause psychosocial distress.

*Aims*. This study examines the effectiveness of telephone contact versus a clinic appointment among patients followed for IBD.

*Methods*. Consecutive IBD patients were randomly assigned to either clinic follow-up visit (CFV) by an IBD nurse practitioner or telephone follow-up visit (TFV) with an IBD nurse practitioner 3 months after their current appointment. Standardized questionnaires, including demographics, IBD phenotype, disease activity, medication, quality of life, resource utilization, anxiety and depression were completed at baseline and six months visits using an on-line survey. Patient satisfaction and preference were evaluated in focus group sessions.

*Results*. Sixty patients were recruited with (median age for CFV 33.5 (IQR 28–53.25 years) versus 35 (IQR 27–48 years) of whom 62% were female and 88% had Crohn's disease, and 90% were receiving biological therapy. The mean parking and travel costs for patients randomized to CFV were $25.83, and their average loss of income was $17.00. The median duration of health care contact was longer in the CFV group (52 minutes (IQR 38–81) versus 17 minutes (IQR 15.0–21.2); *p* ≤ 0.01), with a wait time was longer in CFV (median 31.6 (IQR 8–56) versus 0 minutes *p* < 0.01). Rates of interim health care contact did not differ between the two arms. No significant change in health-related quality of life (Short Inflammatory Bowel Disease Questionnaire) or satisfaction (Patient Satisfaction Questionnaire) from baseline to 6-month follow-up was observed. There was also no significant change in C-reactive protein (CRP), Harvey-Bradshaw Index (Crohn's disease) or Partial Mayo Score (ulcerative colitis). At 6 months subjects in the TFV arm had lower median total HADS score (8 versus 12, *p* = 0.045) and lower median HADS depression score (*p* = 0.046). A common theme mentioned by TFV subjects in focus groups was their satisfaction with time and money saved via telephone communication. The patients preferred telephone visits when in remission and clinic visits during relapse of their disease.

*Conclusions*. Our feasibility study has shown that telephone visit is cost saving and preferred by patients with IBD. This mode of follow-up care was also associated with better anxiety and depression scores. Further research is needed to explore how TFV can best be integrated in patient management algorithms.

*Funding Agencies: *This study was supported by Hamilton Health Sciences Clinical Health Professional Investigator Grant

# Sub article: A258: Antibiotics and Bowel Preparation Enhance the Ability of Fecal Microbial Transplantation to Reshape the Gut Microbiota in IL-10^−/−^ Mice

**Authors:** B. Millan, H. Park, N. Hotte, R. Fedorak, D. Kao, K. Madsen

**Affiliations:** ^258.1^University of AlbertaEdmontonABCanada

*Background*. Although highly effective for the treatment of* C*.* difficile* colitis, fecal microbial transplantation (FMT) has shown less efficacy in inflammatory bowel disease (IBD). A lack of benefit of FMT is associated with failure of the donor microbial community to engraft in the recipient. IL-10^−/−^ mice exhibit a microbial dysbiosis and develop colitis by 12–14 wks of age.

*Aims*. The aim of this study was to determine if pre-treatment of IL-10^−/−^ mice with antibiotics (abx) or a bowel preparation would enhance engraftment of the donor microbial community.

*Methods*. IL-10^−/−^ mice (*n* = 20) received the following treatments: (1) FMT only; (2) bowel preparation (BP) with PEG3350 (17.9 mEq/L) overnight followed by FMT; (3) vancomycin (0.5 g/L) and metronidazole (1.0 g/L) for 3 days followed by FMT; (4) vancomycin and metronidazole plus BP followed by FMT. A pooled fecal sample from wild-type mice was used for the single FMT. Stool was collected at baseline, day 0 (following treatment), 3 days following FMT, and at day 30. 16SrRNA sequencing was performed using Illumina MiSeq. Lipocalin-2 (LCN-2) was measured in stool to assess gut inflammation.

*Results*. Mice administered FMT with no preparation or just a BP had increased LCN-2 at day 30 indicating the development of colitis. In contrast, mice pre-treated with abx prior to receiving an FMT had no increase in LCN-2 over the 30 days, suggesting a prevention or delayed development of colitis. Principal coordinates analyses showed samples from control mice which just received an FMT or BP to cluster together at all time points and separate from the donor indicating limited engraftment of the donor community. Samples from mice treated with abx clustered separately from baseline. This was associated with significant increases in the relative abundance of Proteobacteria and decreases in Prevotella. Following FMT, samples from mice pre-treated with abx showed a community profile close to the donor sample, with decreases in Proteobacteria and Bacteroides and increases in Akkermansia, Lactobacillus, and Prevotella. By day 30, the abx group had reverted back towards their original composition. However, mice receiving both abx and BP showed a higher degree of engraftment and retained the donor community profile longer than antibiotic treatment alone.

*Conclusions*. Pre-treatment with abx and a BP resulted in an enhanced engraftment of the donor microbial community and a reduction in colitis. These results indicate that preparation of the host has substantial effects on the efficacy of FMT and should be considered when determining the pre-treatment regimen for human patients undergoing FMT.

*Funding Agencies: *CIHR

# Sub article: A259: Illness Perceptions in Inflammatory Bowel Disease Influence Psychological Outcomes

**Authors:** D. Tripp, H. Yurgan, C. Nickel, M. Ropeleski, S. Vanner, M. Beyak

**Affiliations:** ^259.1^Queen's UniversityKingstonONCanada

*Background*. The uncertain etiology of IBD leads patients to report a variety of causes for this illness. Currently, no IBD research has captured what patients qualitatively report to be the cause of their IBD in relation to symptom and psychosocial outcomes.

*Aims*. To determine how patients' own perception of the cause of illness influences important psychological outcomes.

*Methods*. Patients attending a teritary hospital IBD clinic completed a questionnaire package assessing demographics, illness perceptions, depressive symptoms, pain, pain catastrophizing, and QOL. Directed content analysis was used to categorize patients' qualitatively reported causes associated with their IBD. Multivariate Analyses of Variance were then used to examine the differences between qualitative response groups by pain, quality of life, pain catastrophizing, and depressive symptoms.

*Results*. The investigators categorized five qualitative patient-reported causes for IBD: biological (*n* = 76; 40.6% of sample), psychological (*n* = 61; 32.6%), diet/lifestyle (*n* = 22; 11.8%), unknown (*n* = 20; 10.7%) and environmental (*n* = 8; 4.3%). Patients attributing a psychological cause to their IBD reported greater depressive symptoms than those who reported a biological or unknown cause. Patients reporting a psychological cause also reported poorer emotional quality of life than those who reported a biological cause.

*Conclusions*. Nearly a third of patients attribute a psychological cause for their IBD. Attributing a psychological cause to IBD is associated with poorer outcomes. Patient qualitative reports of the cause of their IBD are important to consider because they are linked to depression and poorer quality of life. These disease attributions may be modifiable, and also may be a marker for increased burden of psychological dysfunction in IBD.

*Funding Agencies: *CCC

# Sub article: A260: Inhibition of B-RAF Decreases Systemic Inflammation in Animal Models of Colitis

**Authors:** A. Zalasky, M. Laffin, N. Hotte, B. Millan, A. Agrawal, J. Walker, K. Madsen

**Affiliations:** ^260.1^University of AlbertaEdmontonABCanada

*Background*. The mitogen-activated protein kinase (MAPK)/ERK pathway has an integral role in the modulation of inflammatory pathways and is upregulated in patients with inflammatory bowel disease (IBD). BRAF is a serine/threonine protein kinase that regulates the MAPK/ERK1/2 pathway. Dabrafenib (DAB) is a BRAF inhibitor used in the treatment of metastatic melanomas.

*Aims*. The aim of this study was to test the hypothesis that inhibition of BRAF by the oral administration of DAB would inhibit inflammatory pathways directly in epithelial cells and reduce the severity of inflammation in mouse colitis models.

*Methods*. HT-29 epithelial cells were treated with lipopolysaccharide (LPS) and tumor necrosis factor (TNF) ± DAB (1 *μ*g/mL). RT-PCR was used to measure gene expression. Adult IL10^−/−^ mice (*n* = 8) were treated with DAB (30 mg/kg) daily via oral gavage for 25 days. Controls were gavaged with vehicle. Mouse weights were measured daily and fecal samples collected for measurement of lipocalin-2 (LCN-2) as a marker of inflammation. Serum and tissue samples (ileum, colon) were analyzed for cytokine levels by MesoScale Discovery Platform. Dextran sulfate sodium (DSS) was used to induce colitis in wild-type mice. Mice (*n* = 12) were given DSS (2.5% in drinking water) for 5 days followed by water for 2 days. Treatment mice (*n* = 6) were given DAB (30 mg/kg) via daily oral gavage for the 7 days. Animal weight, hemocult, and stool consistency were scored daily (DAI). CXCL1/KC levels were measured by ELISA in ileal and colonic homogenates and serum.

*Results*. In HT-29 epithelial cells, incubation with DAB prevented an LPS and TNF-induced increase in IL-1*β* and also significantly down-regulated expression of iNOS, iCAM, and COX2. Treatment of IL10^−/−^ mice with DAB resulted in a significant decreased expression of IL-1*β* and IL-2 in serum and decreased IL-2, IL-4, IL-6, IL-12 and CXCL1/KC in the terminal ileum (*p* < 0.05). However, there were no significant effects of DAB treatment on colonic cytokine expression or in lipocalcin levels. A similar finding was seen in DSS-treated mice, where DAB treatment had no effect on colitis severity as evidenced by DAI scores and actually increased expression of CXCL1/KC in the colon. However, DAB treatment did reduce serum levels of CXCL1/KC (*p* < 0.002) in DSS treated mice.

*Conclusions*. An oral inhibitor of BRAF was ineffective at reducing colonic inflammation in both an acute and chronic animal model. However, the findings that DAB reduced inflammatory mediators in cell culture and also in serum and ileum suggest that the compound may have been absorbed in the small intestine and not reached the colon, indicating a possible role for DAB in the control of systemic inflammation.

*Funding Agencies: *CIHR

# Sub article: A261: Endothelial TLR4 Is a Key Player in Gut Inflammation

**Authors:** T. Wang, D. Sigalet, F. Zamp, E. de Heuvel, C. Waterhouse

**Affiliations:** ^261.1^University of CalgaryCalgaryABCanada

*Aims*. Intestinal commensal microflora triggers an aberrant immune response in patients of inflammatory bowel diseases (IBD) and experimental colitis. Toll-like receptor 4 (TLR4), the receptor for lipopolysaccharide (LPS), has complex functions in the gastrointestinal tract. The role of TLR4 might be cell-type-dependent. To date, the role of endothelial TLR4 in IBD remains unclear. In this study, we tested our hypothesis that endothelial TLR4 is upregualted in gut inflammation and provokes neutrophil recruitment.

*Methods*. Wild type (WT) C57BL/6 mice, mice with endothelial specific deficiency of TLR4 (TLR4^ΔEndo^,* VE*-*Cadherin (VE*-*Cad*)-*Cre* mice crossed to* TLR4*-*loxp* mice) as well as mice harboring* TLR4*-*loxp* and* Ve*-*Cad*-*Cre* ^−*/*−^ (TLR4^loxp^Cre^−/−^) were used in this study. The administration of 4% dextran sodium sulfate (DSS) in drinking water was used to induce colitis for 3 or 5 days. At Day 5, part of the animals were sacrificed and others received regular water for 9 days. Full-length colons were harvested at Day 5 and 14 for histological damage. Colon myeloperoxidase (MPO) was measured as the marker for neutrophil infiltration. Immunofluorescence (IF) and flow cytometry were used to show the expression of TLR4. Chemokine MIP2 concentration was measured by ELISA. The process of neutrophil-endothelial cell interaction was recorded by intravital microscopy. Colonoscopy biopsy samples from IBD patients were collected for IF staining.

*Results*. At Day 3, elevated expression of endothelial TLR4 was observed in DSS-induced colitis WT mice, whereas the MPO level was similar to that of non-treated mice and significantly lower than that of Day 5. In the recovery phase, TLR4^ΔEndo^ manifested a trend to faster body weight restoration, although no difference of body weight change was observed between TLR4^ΔEndo^ and TLR4^loxp^Cre^−/−^ during acute colitis. TLR4^ΔEndo^ had longer colons and improved histological score compared to TLR4^loxp^Cre^−/−^ at both Day 5 and Day 14. Assessment by MPO, TLR4^ΔEndo^ had significantly less neutrophil infiltration at both Day 5 and Day 14. A lower number of adhered neutrophils and a higher speed of neutrophil rolling were observed in the vasculature of colons in TLR4^ΔEndo^ at both Day 5 and Day 14. At Day 5, TLR4^ΔEndo^ had lower levels of MIP2 in the colons. In agreement with experimental colitis, the expression of endothelial TLR4 in inflamed mucosa of IBD patients was also upregulated compared to that of normal mucosa.

*Conclusions*. Endothelial TLR4 is upregulated in the colons of experimental colitis mice and this upregulation occurs prior to neutrophil recruitment. Loss of endothelial TLR4 improves histological damage and inhibit neutrophil recruitment in the colons of experimental colitis mice.

*Funding Agencies: *None

# Sub article: A262: Epithelial SHP-2 Activation Protects the Intestinal Mucosa against Colitis Induced by Dextran Sulfate Sodium or Citrobacter Rodentium

**Authors:** J. Gagné Sansfaçon, S. Tremblay, C. Qu, A. Menendez, N. Rivard

**Affiliations:** ^262.1^Université de SherbrookeSherbrookeQCCanada; ^262.2^Aflac Cancer and Blood Disorders CenterAtlantaGAUSA; ^262.3^University of SherbrookeSherbrookeQCCanada

*Background*. SHP-2 is a protein tyrosine phosphatase expressed in most embryonic and adult tissues. SHP-2 regulates many cellular functions and major signalling pathways including the RAS/MAPK, PI3K/Akt and JAK/STAT. Interestingly, variations within the human gene locus encoding SHP-2 have been associated with increased susceptibility to develop ulcerative colitis (Narumi et al., 2009). Furthermore, we recently reported that mice with conditional deletion of SHP-2 in intestinal epithelial cells rapidly develop severe colitis (Coulombe et al., 2013).

*Aims*. Since our previous results suggest that SHP-2 may protect the intestinal epithelium against mucosal injury, we tested this hypothesis in vivo.

*Methods*. We have generated mice expressing a constitutive active form of SHP-2 specifically in intestinal epithelial cells (IEC), using the Cre-loxP system (*Shp*-2^*IEC*-*E*76*K*^ mice). These mice were challenged with Dextran Sulfate Sodium (2.5% DSS in water for 7 days; 3 × 7 day-cycles of 1.5% DSS/2 weeks of recovery) to induce chemical colitis. *Shp*-2^*IEC*-*E*76*K*^ mice were also inoculated by oral gavage with* Citrobacter rodentium* to induce infectious colitis. Stool consistency, rectal bleeding and colon hardness were monitored to calculate the disease activity index and histological analysis were performed.

*Results*. Results show that 1 month after birth, *Shp*-2^*IEC*-*E*76*K*^ mice exhibit increased number of Goblet cells and mucus secretion (Alcian blue staining). By contrast, Paneth cell number was decreased as well as the expression of several antimicrobial peptides including lysozyme, defensins and Reg3*γ*/*β*. These changes were associated with higher ERK1/2 activation levels and lower expression of *β*-catenin protein and its target genes (*Axn2*,* Ccnd1*). Importantly, *Shp*-2^*IEC*-*E*76*K*^ mice lost much less weight and exhibited less diarrhea and bloody stools after DSS treatment while showing nearly no histological alterations at the microscopic level. Additionally, *Shp*-2^*IEC*-*E*76*K*^ mice exhibited faster recovery (body weight curve) after chronic DSS treatment. Finally, *Shp*-2^*IEC*-*E*76*K*^ mice were less sensitive to* C*.* rodentium* infection with a decrease in bacterial load when compared to control littermates.

*Conclusions*. In conclusion, these studies demonstrate that SHP-2 activation exerts protective actions against mucosal damage and during infection with an A/E bacterial pathogen. Thus, this suggests that SHP-2 might represent a potential therapeutic target in disorders associated with chronic mucosal ulcerations.

*Funding Agencies: *CIHR, FRQS

# Sub article: A263: Dietary Restrictions in Inflammatory Bowel Disease

**Authors:** F. Bergeron, L. D'Aoust, M. Lemoyne, M. Bouin

**Affiliations:** ^263.1^Université de MontréalMontrealQCCanada

*Background*. Inflammatory bowel disease is a chronic condition in which episodes of remission alternate with exacerbation of the disease. Several digestive symptoms may lead to a restrictive diet and many patients associate some foods as trigger of disease exacerbation. Consequently, dietary restrictions may persist even when the disease is in remission. The Canadian population has yet to be studied to identify the prevalence of dietary restrictions in patients with inflammatory bowel disease.

*Aims*. Compare the frequency of dietary restrictions between periods of remission and exacerbation in patients with inflammatory bowel disease.

*Methods*. Prospective study conducted from October 2013 to March 2014 at St-Luc Hospital, a tertiary center. All patients 18 years or older with a confirmed diagnosis of inflammatory bowel disease who consulted the outpatient clinic were approached for inclusion. Participants completed a self-assessed form on the characteristics of their disease and on dietary restrictions according to the disease activity. The dietary restrictions were assessed using a list of foods on which each participant identified the items they restrict from their diet when the disease is in remission. The same list was then re-assessed by the patient to identify the items they restrict during exacerbation. The exclusion of at least one item on the list was considered to be a restrictive diet.

*Results*. 246 participants were included (mean age: 44.9 ± 12.6 years, 56.9% females, 70.7% Crohn's Disease and 29.3% Ulcerative Colitis). During remission, 74.8% of participants adopt a restrictive diet compared to 91.1% during exacerbation (*p* < 0.001). An average of 10 ± 12 items are excluded from the diet in periods of remission, compared to an average of 24 ± 19 items during exacerbation (*p* < 0.001). During remission, 80.5% (*n* = 140) of the Crohn's Disease subgroup have a restrictive diet, in comparison to 61.1% (*n* = 42) in the Ulcerative Colitis subgroup (*p* = 0.01). During exacerbation, 93.1% (*n* = 162) of the Crohn's Disease subgroup adopt a restrictive diet, in comparison to 86.1% (*n* = 62) in the Ulcerative Colitis subgroup (*p* = 0.08).

*Conclusions*. A restrictive diet is adopted by 74.8% of participants during remission, compared to 91.1% during exacerbation. Patients with Crohn's Disease are more prone to restrict their diet compared to those affected with Ulcerative Colitis, and this during remission (80.5% versus 61.1%) and exacerbation (93.1% versus 86.1%).

*Funding Agencies: *None

# Sub article: A264: The Gut Microbiome Differentiates Clinical Phenotypes in Moderate to Severe Crohn's Disease: Results from the CERTIFI Study

**Authors:** T. Ding, S. Telesco, C. Monast, C. Brodmerkel, T. Yatsunenko, A. Das, P. Schloss

**Affiliations:** ^264.1^University of MichiganAnn ArborMIUSA; ^264.2^Janssen Scientific AffairsLLCSpring HousePAUSA; ^264.3^Second Genome IncSan FranciscoCAUSA

*Background*. The pathogenic role of the gut microbiome in inflammatory bowel disease (IBD) has been highly studied but remains unknown.

*Aims*. The aim of this study was to investigate the relationship between the fecal microbiome and clinical phenotypes in pts with moderately-severely active CD.

*Methods*. CERTIFI was a Phase 2b multicenter, randomized, double-blind, placebo controlled clinical trial to evaluate the efficacy and safety of ustekinumab therapy in pts with moderately- severely active CD who had previously not responded to anti-TNF*α* therapy. Fecal samples from 100 pts, collected at screening and stored at −80°C, were selected for microbiome analysis. Bacterial DNA was extracted from fecal samples and subjected to 16S rRNA sequencing and shotgun metagenomic sequencing. 16S rRNA sequencing was performed on the GS-FLX 454 Titanium platform and the sequences were assigned genus-level annotations. Metagenomic sequencing was performed on the Illumina HiSeq 2000 using 100 base pair paired-end processing. Filtered sequences were mapped against the MetaCyc database of metabolic pathways and enzymes. Spearman correlation and Adonis were applied to identify bacterial taxa or pathways associated with clinical variables.

*Results*. The gut microbiome of individuals with CD was characterized by pronounced inter-personal variation in the presence and relative abundance of specific bacterial taxa. Despite this heterogeneity, bacterial taxa and pathways correlated with patient sub-groups defined by specific baseline clinical traits. The baseline CDAI score significantly associated with the relative abundance of several bacteria, including Parabacteroides (rho = −0.42, *P* < 1*e* − 4). The metagenomic data supported this result, demonstrating correlation between specific metabolic pathways and CDAI score. Baseline CRP, fecal calprotectin (FCALP), and lactoferrin (FLACT) concentrations also correlated with baseline bacterial abundances of specific taxa, including Dialister (rho = 0.36, *P* = 3*e* − 4, Spearman correlation with FCALP), and with metagenomic data. Previous response to anti-TNF*α* therapy did not significantly correlate with the abundance of any specific bacteria or with metagenomic data.

*Conclusions*. The fecal microbiome demonstrated the ability to discriminate clinical phenotypes in moderately-severely active CD pts who had previously not responded to anti-TNF*α* therapy. The strongest associations between metadata and the microbiome, supported by 16S and metagenomic data, were observed for CDAI score, FCALP, and FLACT. The results suggest the potential application of the fecal microbiome as a molecular marker of disease severity in CD.

*Funding Agencies: *Janssen R & D, LLC

# Sub article: A265: Functional Analysis of a Coding Variant in* GPR65* That Is Associated to Chronic Inflammation

**Authors:** V. Mercier, G. Charron, D. Devost, A. Alikashani, C. Beauchamp, L. Villeneuve, G. Boucher, S. Foisy, P. Goyette, T. Hébert, A. Inoue, J. Rioux

**Affiliations:** ^265.1^Université de Montréal & Montreal Heart InstituteMontréalQCCanada; ^265.2^Montreal Heart InstituteMontrealQCCanada; ^265.3^Department of Pharmacology and TherapeuticsMcGill UniversityMontréalQCCanada; ^265.4^Graduate School of Pharmaceutical Sciences Tohoku UniversityAoba-kuSendaiJapan

*Background*. Inflammatory bowel diseases (IBD), mainly comprising ulcerative colitis (UC) and Crohn's disease (CD), result in the chronic inflammation of the gastrointestinal tract. Genome-wide association studies have associated the 14q31 locus, including the genes* galactosylceramidase (GALC) *and* G protein*-*coupled receptor 65 (GPR65)*, to both phenotypes.* GPR65* encodes a pH-sensing G protein-coupled receptor.

*Aims*. Our objective was to define pathways downstream of activation of GPR65 and to evaluate the impact* GPR65* ^*^ *231Leu* on these pathways.

*Methods*. We used HEK 293 cells stably expressing GPR65 and deficient for either G*α*s/olf, G*α*q/11 or G*α*12/13 to evaluate the impact of these mutations on actin cytoskeletal remodeling and cAMP accumulation using cAMP detection, F-actin accumulation and G-LISA RhoA activation assays.

*Results*. We determined that* GPR65* is expressed in lymphoid and mucosal tissues as well as in immune cell lines and human primary immune cells. We also found that its expression is significantly increased in inflamed biopsies from UC patients compared to non-inflamed biopsies and biopsies from healthy controls. The most associated variant in the locus 14q31 (rs8005161; *p* = 2,35 × 10^14^) is correlated (*r* ^2^ = 1) to a missense coding variant of* GPR65 *(rs3742704: Ile231Leu). Upon activation by low pH, GPR65 stimulates accumulation of cAMP, formation of stress fibers and activation of the RhoA pathway. We demonstrated that cAMP accumulation upon activation of GPR65 was, at least partly, dependent on the G*α*s pathway and only slightly or not at all on the G*α*q/11 pathway. Our results also revealed that* GPR65* ^*^ *231Leu *variant reduced GPR65-dependent stress fiber formation and inhibited the increase of filamentous actin (F-actin) content versus free globular-actin (G-actin).

*Conclusions*. We are currently pursuing the characterization of the impact of* GPR65* ^*^ *231Leu* variant on actin remodelling with G*α*s/olf, G*α*q/11 or G*α*12/13 deficient cells. The actin cytoskeleton is important for many cellular processes in normal immune cells including motility, intercellular interactions, endocytosis, cytokinesis, signal transduction, maintenance of cell morphology and autophagy, a cellular process that digests and recycles proteins. Furthermore, variants within genes coding for proteins involved in some of these cellular processes, like autophagy, have been associated in susceptibility to IBD.

*Funding Agencies: *U.S. National Institute of Diabetes and Digestive and Kidney Diseases

# Sub article: A266: Inflammatory Bowel Disease Wellness and Nutrition Questionnaire

**Authors:** A. Harding, T. Mombourquette, K. Phalen-Kelly, J. Kiberd, J. Wangsgaard, G. Shannan, B. Currie, J. Jones, S. Williams, J. Stewart

**Affiliations:** ^266.1^Queen Elizabeth II Health Sciences CentreHalifaxNSCanada; ^266.2^Mount Saint Vincent UniversityHalifaxNSCanada

*Background*. Inflammatory bowel disease (IBD) patients struggle with diet and nutrition and most find it necessary to adopt changes in their dietary patterns to address their symptoms. Previous studies have found that while the majority of IBD patients view nutritional information as very important, only a small group report receiving adequate information on the topic. Both disease and nutritional status may interact to affect body image.

*Aims*. To assess diet and nutrition educational needs, interests, and perceptions of IBD patients and to identify what diets IBD patients follow. To determine what affect IBD has on their daily life and body image satisfaction.

*Methods*. Self-administered questionnaires were completed by patients attending the outpatient IBD clinic at the QEII Health Sciences Centre in Halifax in Sept. 2015. All patients over the age of 18 with a diagnosis of Crohn's Disease (CD), Ulcerative Colitis (UC) or Indeterminate Colitis were invited to participate. UC patients who had a total colectomy were excluded. This study was conducted in two phases. Phase one (development) included creation and face and content validation with patient and professional samples. During phase two (implementation) the revised 38-question survey was used to collect information on patient demographics, symptoms, lifestyle, body image, diet, and education interests.

*Results*. Fifty surveys were completed by study participants. Three surveys were removed from analysis due to missing data. The mean age of the sample was 44 years (range 24–84). Forty-five percent [21] of the sample was female. Seventy percent [33] of participants had a diagnosis of CD and 30%  [14] had a diagnosis of UC. The mean BMI was 28 ± 6 kg/m^2^ and was not significantly different between disease groups (*p* = 0.79). Sixty percent [28] of participants reported feeling less sexually attractive as a result of their disease. Thirty-two percent [15] of participants believed diet caused their disease and 83%  [39] believed diet could cause worsening of symptoms. Of the total sample, 87%  [41] participated in food avoidance and 55% [26] reported following a special diet to manage IBD symptoms. Eight-five percent [40] of participants reported they want more nutritional education. Sixty-eight percent [32] were interested in this material being presented on a website and 47%  [22] were interested in a print medium.

*Conclusions*. This study supports that dietary beliefs and food intake are very important aspects of disease management for IBD patients. The results of this study will inform future education and research development.

*Funding Agencies: *None

# Sub article: A267: Intensification of Infliximab Induction Regimen Improves Response Rate in Steroid-Refractory Paediatric Ulcerative Colitis

**Authors:** S. Ho, A. Sharma, K. Frost, T. Walters, P. Church, A. Griffiths

**Affiliations:** ^267.1^Hospital for Sick ChildrenTorontoONCanada

*Background*. Infliximab is commonly given as rescue therapy for children and adolescents hospitalized with steroid-refractory ulcerative colitis (UC), and increasingly as an alternative to thiopurines in those with steroid-dependent disease. The demonstration of rapid loss of infliximab from serum in patients with active colitis has led to intensification of dosing, but the efficacy of this practice in children has not previously been assessed.

*Aims*. We reviewed our single-centre experience with intensified versus standard infliximab dosing in UC patients treated for steroid-refractory (SR) and steroid-dependent (SD).

*Methods*. The records of all UC patients aged <18 years who received planned 3-dose infliximab induction between June 2003 and November 2014 at the Hospital for Sick Children, Toronto, were reviewed. Patients were categorized as SR, unresponsive to steroids or SD, clinical remission achievable with steroids, but not maintained as steroids tapered. Patients were induced with standard regimen, 5 mg/kg/dose (rounded up to the nearest 100 mg) given at Week 0, 2, 6 or intensified regimen, ≥7 mg/kg and/or 3 induction doses given within 5 weeks. Clinical remission and response were assessed at Week 8 using physician global assessment (PGA) and paediatric ulcerative colitis activity index (PUCAI). Clinical response was defined by decreased of PUCAI ≥20 from baseline; clinical remission by PUCAI <10 and PGA of inactive disease.

*Results*. 125 children (59% male; median age at diagnosis 12.7 years (IQR 9.7–15.3)) received infliximab treatment for SR (*n* = 74) or SD (*n* = 51) UC. Induction regimen was standard in 73 (58%) and intensified in 52 (42%).  Table 46 shows patients response to infliximab induction. SR patients had higher clinical response and remission with intensified induction compared to standard induction. No difference in clinical response or remission observed in SD patients treated with standard versus intensified induction. Among 35 primary non-responders, 20 had colectomy within 6 months following stopping infliximab. Intensified induction is the only factor identified to influence the likelihood of achieving clinical response in overall patient group (OR = 2.64, 95% CI 1.12–6.27).

*Conclusions*. Intensification of infliximab induction is beneficial in the treatment of children with steroid-refractory UC, but does not improve primary response rates in ambulatory steroid-dependent patients. Point-of-care infliximab level testing would guide optimal dosing for all patients.

*Funding Agencies: *Data extraction for this study was supported in part by an investigator-initiated grant from Janssen

# Sub article: A268: The Association between Anxiety and Depression in Health Related Quality of Life with Inflammatory Bowel Disease

**Authors:** U. Chauhan, Y. Farbod, J. Popov, D. Armstrong, S. Halder, S. Kaasalainen, J. Marshall, F. Tse, P. Moayyedi

**Affiliations:** ^268.1^McMaster University Medical CentreHamiltonONCanada; ^268.2^McMaster UniversityHamiltonONCanada

*Background*. Inflammatory Bowel Diseases (IBD), including Crohn's disease (CD) and Ulcerative Colitis (UC) are chronic disorders of the gastrointestinal tract, which can cause major life challenges. Their lifelong progression is associated with substantial psychological burden, which increases the risk of mental health disorder. Symptoms of anxiety and depression have been reported in patients with IBD independent of disease activity. IBD outcomes have further been reported to affect the health related quality of life in the individuals suffering from this chronic disorder.

*Aims*. Our study examines the association between depression and anxiety and health related quality of life in IBD patients.

*Methods*. A prospective cohort study was conducted over a 6-month period. Standardised questionnaires were collected at baseline and at 6 months using LimeSurvey and included the Hospital Anxiety and Depression Scale (HADS). Health related quality of life was captured using Short IBD questionnaire (SIBDQ). The relationship between depression and anxiety score, and health related quality of life was assessed using the Spearman's rho correlation coefficient.

*Results*. Sixty respondents with age range 18 to 76 were included. The majority (65%) were between ages 18 to 40; 61% were female, and 85% had CD. The mean (SD) HADS anxiety score at baseline and 6 months were 8.4 (3.3) and 7.4 (4.5); depression scores were 3.7 (3.0) and 4.3 (4.0), respectively. The SIBDQ total score at the baseline was significantly correlated with HADS anxiety (*r* = 0.51, *p* < 0.0001) and HADS depression scores (*r* = 0.64, *p* < 0.0001). Furthermore, the SIBDQ total score at the 6-month visit was also significantly correlated with the HADS anxiety (*r* = 0.63, *p* < 0.0001) and HADS depression scores (*r* = 0.67, *p* < 0.0001).

*Conclusions*. The data shows a significant correlation between SIBDQ total score, and HADS anxiety and depression score. Clinical anxiety and depression could be a result of IBD and various life challenges, which occur during young adult lives as majority of our patients, were under the age of 40. During this time major life events such as university education, graduation, finding a job, marriage, having children, and stabilizing self for a lifetime occurs. This can lead to anxiety and depression symptoms especially in context of dealing with chronic life long illness.

*Funding Agencies: *None

# Sub article: A269: Endogenous Opioid Signalling in Chronic Inflammatory Bowel Disease (IBD) Prevents Pronociceptive Actions of High Dose Opioid Treatment

**Authors:** J. Jaramillo Polanco, C. Lopez Lopez, A. Lomax, S. Vanner

**Affiliations:** ^269.1^Queen's UniversityKingstonONCanada

*Background*. The utility of narcotic analgesics in the treatment of IBD-associated pain is limited because escalating doses of these drugs can paradoxically induce pronociceptive signalling in patients leading to worsening pain. During inflammation, the endogenous opioid system may mitigate this action but it is unclear whether this occurs in inflammatory bowel disease (IBD). We and others have previously shown that endogenous opioids secreted by colonic immune cells during chronic colitis in mice inhibit nociceptive dorsal root ganglia (DRG) neurons.

*Aims*. The present study evaluated the effect of overnight exposure to high concentrations of the *μ*-opioid receptor agonist DAMGO on the excitability of nociceptive DRG neurons, and whether endogenous opioid signaling during chronic colitis can alter these actions.

*Methods*. Mice with chronic colitis (treated with 3 cycles of 2% DSS, each cycle of 5 days with DSS and 5 days with water) were euthanized and colonic tissue supernatants collected. Nociceptive DRG neurons were dissociated from control mice and incubated overnight with cDSS supernatants and 10 *μ*M DAMGO (alone or together). The effects on excitability of neurons was measured using perforated patch clamp to record changes in rheobase and action potential discharge at twice rheobase.

*Results*. Overnight exposure to a high concentration of opioid (10 *μ*M DAMGO) had a pronociceptive effect on DRG neurons. The rheobase decreased by 33% (*p* < 0.01) and action potential discharge increased by 12% (*p* = NS). In contrast, neurons incubated with cDSS supernatant containing endogenous opioids had an antinociceptive effect. The rheobase increased by 28% (*p* < 0.05) and the action potential discharge decreased by 26% (*p* = NS). Importantly, cDSS supernatant abolished the pro-nociceptive effect of overnight incubation with high dose DAMGO (rheobase increased by 60%, *p* < 0.001; action potential discharge decreased by 26%, *p* = NS). This beneficial action was also evident when the cDSS supernatant was applied following the application of high dose DAMGO.

*Conclusions*. These data suggest that the endogenous opioid system in chronic colitis can protect against the detrimental pronociceptive effect of high dose exogenous opioids. Thus, targeting this system could be exploited to prevent the development of opioid analgesic tolerance in IBD patients.

*Funding Agencies: *CCC

# Sub article: A270: Validation of Administrative Data for Capturing Crohn's Disease Patients Requiring Surgical Bowel Resection

**Authors:** C. Ma, R. Panaccione, G. Moran, E. Benchimol, C. Seow, Y. Leung, K. Novak, M. Iacucci, S. Ghosh, G. Kaplan

**Affiliations:** ^270.1^University of CalgaryCalgaryABCanada; ^270.2^University of NottinghamNottinghamUK; ^270.3^Children's Hospital of Eastern OntarioOttawaONCanada

*Background*. Administrative databases have been widely used to evaluate surgical outcomes in Crohn's disease (CD) patients but the validity of administrative data for defining the diagnosis of CD and CD-related bowel resections has not been adequately validated.

*Aims*. To evaluate the accuracy of International Classification of Disease (ICD) coding in identifying patients who are admitted for CD and undergo bowel resection.

*Methods*. Population-based surveillance was conducted in the Calgary Health Zone between January 1 and December 31, 2011 using the Discharge Abstract Database to identify adults (≥18 years) admitted for CD who underwent surgical resection using Canadian Classification of Health Intervention (CCI) coding. Surgical resection codes were stratified by site of resection, surgical approach, surgical urgency, and post-surgical anatomy (anastomosis, stoma, or pouch). The administrative data was validated against chart review and reported as a positive predictive value (PPV) with 95% confidence interval (CI).

*Results*. The administrative database identified 104 admissions of CD requiring bowel resection and correctly identified the diagnosis of CD in 101/104 patients (97.1%, Figure  1). Administrative data was highly predictive for small bowel (PPV 0.86 (95% CI: 0.70–0.95)) and large bowel CD (PPV 1.00 (0.80–1.00)), but was less accurate for ileocolonic CD (PPV 0.67 (0.46–0.83)). Sensitivity for ileocolonic CD improved when K50.8 and K50.9 (CD, unspecified) codes are combined (0.85 (0.68–0.94)).

112 surgical resections were performed. The administrative data was accurate in identifying partial small (PPV 0.87 (0.75–0.94)) or large bowel resections (PPV 0.81 [0.64–0.91]), but less accurate for partial rectal excisions (PPV 0.57 (0.22–0.88)). It was also accurate for defining elective surgery (PPV 0.90 (0.79–0.96)), and open (PPV 0.93 (0.84–0.97)) versus laparoscopic (PPV 0.83 (0.67–0.93)) approach but was only moderately predictive of post-surgical anatomy (Table 47).

*Conclusions*. In CD patients requiring bowel resection, administrative data accurately identifies large or small bowel CD, surgical urgency, location, and approach but is limited for defining ileocolonic CD and post-surgical anatomy. This may reflect the heterogeneous clinical phenotype and complex operations required in this cohort.

*Funding Agencies: *None

# Sub article: A271: MUC2 Mucin Deficiency Disrupts Iron Homeostasis and Increases Susceptibility towards LPS Induced Inflammation and Mortality

**Authors:** M. Kumar, F. Moreau, A. Leon Coria, A. Tawiah, K. Chadee

**Affiliations:** ^271.1^University of CalgaryCalgaryABCanada

*Background*. While the etiology of inflammatory bowel disease (IBD) is unknown, increased gut permeability is the most common feature among IBD patients. MUC2 mucin is the major component of the colonic mucus layer that separates gut microbiota from the underlying epithelia and its deficiency results in disruption of epithelial barrier function.* Muc2* ^−*/*−^ mice exhibit IBD-like symptoms and are ideal models for studies related to IBD. Due to increased translocation of microbial components,* Muc2* ^−*/*−^ mice show excessive immune cell activation with increased risk for exaggerated immune responses against inflammatory stimulus. Sepsis is an unregulated heightened inflammatory response against microbial components, which may lead to multiple organ failure and death. The risk of sepsis in IBD patients is poorly understood.

*Aims*. To assess if the underlying IBD conditions exhibited by* Muc2* ^−*/*−^ mice could increase susceptibility towards severe inflammatory disease using the LPS-induced sepsis model.

*Methods*. LPS was administered in antibiotic-treated and untreated mice i.p. 5 mg/kg body weight (BW) and monitored for symptoms of sepsis such as weight loss and mortality. One group of mice was pretreated with 100 *μ*M deferoxamine (DFO), an iron chelator, i.p., 1 h prior to LPS administration. Mice were euthanized and sera, peritoneal exudates and spleen cells were used to quantify cytokines/stress markers/iron levels. RNA from peritoneal exudates and liver were used to determine the expression of pro-inflammatory cytokines and hepcidin by RT-PCR.

*Results*.* Muc2* ^−*/*−^ showed constitutively increased levels of endotoxemia (LPS in serum) and elevated levels of pro-inflammatory cytokines IFN-*γ*, TNF-*α* and IL-1*β* in serum as compared to* Muc2* ^*+/+*^ littermates. Peritoneal exudate and spleen cells from* Muc2* ^−*/*−^ mice showed elevated reactive oxygen species (ROS) and cleaved caspase 3. In response to LPS challenge,* Muc2* ^−*/*−^ mice demonstrated significant weight loss and mortality associated with high iron load in the serum as compared to* Muc2* ^*+/+*^. Hepatic hepcidin levels were normal in* Muc2* ^−*/*−^ mice and exhibited decreased rate/levels of hypoferremia following LPS challenge. Predictably* Muc2* ^−*/*−^ pre-treated with DFO decreased mortality significantly in response to LPS. Following antibiotic treatment,* Muc2* ^−*/*−^ had lower levels of serum LPS and was protected against LPS induced mortality.

*Conclusions*. These results suggest that chronic IBD conditions as mimicked in* Muc2* ^−*/*−^ mice, results in dysregulation of iron homeostasis and increased oxidative stress that can increase susceptibility to systemic inflammation such as sepsis.

*Funding Agencies: *None

# Sub article: A272: Long Term Evolution after Proctocolectomy and Ileal Pouch-Anal Anstomosis for Ulcerative Colitis

**Authors:** J. Hercun, R. Lahaie, C. Richard, P. Poitras

**Affiliations:** ^272.1^CHUM-Hôpital Saint-LucMontrealQCCanada

*Aims*. Total proctocolectomy and ileal pouch-anal anastomosis (IPAA) is a surgical procedure for ulcerative colitis (UC). As UC is a form of inflammatory bowel disease (IBD) affecting exclusively the colon, this procedure is considered curative for patients with UC. Our objective was to verify the postoperative evolution of UC patients with IPAA and observe the impact of preoperative factors on long term clinical outcomes.

*Methods*. Files of all 302 patients with an IPAA performed between 1985 and 2014 at the CHUM Hôpital Saint Luc in Montreal were reviewed. 163 cases had a minimal follow-up of 5 years and were included in our analysis. All patients included had a diagnosis of UC; a diagnosis of Crohn's disease (CD) was a contraindication for IPAA. Patients were classified as having classic UC or as having indeterminate colitis (IC) in situations where certain doubts arose regarding the diagnosis.

*Results*. Mean age at diagnosis was 28.8 years and at surgery was 35.9 years. 53% of patients were male. Patients had a mean follow-up of 139 months postoperatively. Preoperatively, 145 cases were classified as definite UC while 18 were considered as IC.

Episodic inflammation of the ileal pouch (pouchitis) was seen in 64 patients: 42 were isolated cases responding to standard therapy; in 22 cases pouchitis was associated with CD. 56% of patients in the IC group had an episode of pouchitis, as opposed to 37% of UC patients.

The initial diagnosis of UC was revised postoperatively for a diagnosis of CD in 35 patients. This diagnosis was based on inflammation of the ileum or proximal GI tract in 19 cases, on fistulising perianal disease in 11 cases and on a combination of both in 5 cases. CD was confirmed in the first 5 years of follow-up in 12 cases, between 5 to 10 years after surgery in 14 cases and after more than 10 years in 9 cases. The mean delay for diagnostic revision was 88 months (between 3 to 270 months). A revised diagnosis of CD was necessary in 50% of the IC patients and in 18% of the UC patients.

*Conclusions*. Proctocolectomy and IPAA offers a cure for UC in a majority of cases. However, the possibility of CD undiagnosed pre-procedure and revealed postoperatively should not be underestimated (at least 13% in our complete series of 302 patients or 21% for those followed more than 5 years). Pouchitis and perineal fistula can be the manifestations of CD after IPAA. CD can appear many months/years after surgery with its frequency increasing over time. Patients with IC were more at risk of seeing their initial diagnosis of UC revised for CD during their long term evolution after IPAA.

*Funding Agencies: *None

# Sub article: A273: Interferon-Y Release Assay versus Tuberculin Skin Test in Patients with Moderate-to-Severely Active Ulcerative Colitis: Results from the PURSUIT UC Program

**Authors:** C. Marano, E. Hsia, S. Xu, W. Sandborn, P. Rutgeerts

**Affiliations:** ^273.1^Janssen R&DLLCSpring HousePAUSA; ^273.2^UCSDLa JollaCAUSA; ^273.3^U of Hosp GasthuisbergLeuvenBelgium

*Background*. The tuberculin skin test (TST) is commonly used to detect latent and active TB.

*Aims*. To report the results of an interferon-y release assay (IGRA) versus (TST) as a screening tool for latent TB (LTB) infection in pts with UC in PURSUIT.

*Methods*. UC pts with moderately-severely active UC were screened for LTB using standard TST and the IGRA to assess eligibility for entry into the induction studies of golimumab (PURSUIT-SC and PURSUIT-IV). Any pt with a newly identified positive finding for TB on a diagnostic test in whom there was no evidence of active TB was permitted to enter provided appropriate treatment for LTB was initiated before or at the time of the first dose of study agent. TST was performed per Mantoux method, using 5 tuberculin units (TU) of purified protein derivative (PPD) standard or 2 TU of PPD RT-23. The TST was deemed positive for LTB infection per local country guidelines for defining an immunosuppressed host or, in the absence of local guidelines, per the presence of induration ≥5 mm. The IGRA used to screen for LTB was QuantiFERON-TB Gold In-Tube test. Overall IGRA and TST results were assessed. The impact of prior BCG vaccination and concomitant medication (i.e., corticosteroids and/or immunomodulators) on outcome was also assessed.

*Results*. 1283 pts had both IGRA and TST screening prior to GLM treatment. Among these pts, 8.7% had at least one test yielding positive for LTB, including 6.2% with positive results only by TST, 3.7% with positive results only by IGRA, and 1.2% with positive results on both tests. The rate of indeterminate results for TB on IGRA was 7.7%. Agreement between the TST and IGRA results, measured by the kappa coefficient, was 0.135 (95% CI, 0.050–0.220; *p* = 0.028). Among pts with positive IGRA, 31.3% had positive TST. Among pts with positive TST, 19.0% had positive IGRA results. Overall, 501 (40.5%) of 1283 pts had previously received BCG vaccine; among this vaccinated group, the rate of positivity for LTB by TST was 10.4% versus 5.0% for IGRA positivity. Among pts who had not received BCG vaccine, the rate of positivity by TST was 1.9% versus 2.8% for IGRA positivity. When IGRA was repeated in pts whose results were initially indeterminant, the majority of pts (67.0%) were IGRA negative on repeat whereas the number of pts who were positive was 5.3%; IGRA remained indeterminate for 27.7%. Overall, 2.1% tested indeterminate on first and repeat screening. Concomitant corticosteroid and/or immunomodulator use did not appear to have an impact on results.

*Conclusions*. Comparison of IGRA and TST in a large cohort of pts with UC suggest that the IGRA provides greater specificity and possibly greater sensitivity than the TST in pts with moderate-severe UC.

*Funding Agencies: *Janssen R & D, LLC

# Sub article: A274: Fecal Lactoferrin and Calprotectin as Surrogate Markers of Mucosal Healing: Post-Hoc Analysis from the PURSUIT SC Induction Study

**Authors:** W. Reinisch, B. Feagan, C. Marano, H. Zhang

**Affiliations:** ^274.1^1U für Innere Medizin III/McMaster UVienna/HamiltonAustria; ^274.2^Robarts Clinical Trials IncLondonONCanada; ^274.3^Janssen R&DLLCSpring HousePAUSA; ^274.4^Janssen R & DLLCSpring HousePAUSA

*Background*. Previously we reported greater improvement in both fLac & fCal levels observed in UC pts who demonstrated clinical response & endoscopic healing in PURSUIT SC induction GLM study (Presented at ACG 2014, Oct 17–22 Phila, Pa).

*Aims*. Assess association of fecal lactoferrin (fLac) & calprotectin (fCal), with Mayo endoscopy subscores using data from PURSUIT SC induction study of GLM.

*Methods*. PURSUIT SC evaluated induction therapy with SC GLM. Pts with Mayo scores of 6–12 inclusive, including endoscopic subscore ≥2 were rand to PBO/PBO, GLM 200 mg/100 mg, or GLM 400 mg/200 mg at Wks 0 & 2. Mucosal healing was assessed at Wk 6 using Mayo endoscopy subscore. Stool samples were collected for fLac & fCal Wk 0 thru Wk 6. Area under a ROC curve (AUC) assessed the association of Wk 0 &6 fLac & fCal with Mayo endoscopy subscores of 0 (normal or inactive disease) & subscores of 0 or 1 (endoscopic healing) at Wk 6. Cutoffs of fLac & fCal were explored to determine the balance of sensitivity & specificity for endoscopy subscores of 0 or 0 or 1.

*Results*. Endoscopy subscores of 0 or 1 were associated with the lowest concentrations of fLac & fCal. Wk 0 fLac & fCal were poor predictors for endoscopic healing at Wk 6 (AUCs < 0.63). AUC analysis showed that fLac & fCal at Wk 6 are fair surrogate markers for normal/inactive endoscopic disease activity at Wk 6 with AUCs of 0.77 & 0.79, respectively. Similar AUCs were observed for endoscopy subscores of 0 or 1. Cutoffs of fLac <50 *μ*g/mL & fCal <250 mg/kg at Wk 6 offered reasonable sensitivity & specificity (fLac 0.74, 0.67 & fCal 0.79, 0.70) for normal/inactive endoscopic disease activity at Wk 6. In contrast, cutoffs of fLac <100 *μ*g/mL & fCal <500 mg/kg at Wk 6 offered similar sensitivity & specificity (fLac 0.73, 0.63 & fCal 0.74, 0.64) for endoscopy subscores of 0 or 1.

*Conclusions*. At Wk 6, Mayo endoscopy subscores were positively associated with levels of fLac & fCal. FLac < 50 *μ*g/mL & fCal < 250 mg/kg at Wk 6 demonstrated reasonable sensitivity & specificity for normal/endoscopic disease activity. These data suggest that fLac and fCal might be useful surrogates for endoscopic improvement.

*Funding Agencies: *Janssen R & D, LLC

# Sub article: A275: Impact of Inflammatory Bowel Diseases on Menopause Experience

**Authors:** E. Donaldson, V. Huang, S. Ross, B. Sydora

**Affiliations:** ^275.1^University of AlbertaEdmontonABCanada

*Background*. Inflammatory Bowel Diseases (IBD) are debilitating, chronic intestinal disorders usually requiring extensive medical intervention. Menopause is a natural stage in a woman's aging process defined by the end of menstruation and frequently associated with a variety of physical and psychological symptoms due to hormonal changes. Symptoms can be influenced by medical treatment, diet and lifestyle. It is not known whether and how IBD and its treatment affects menopause; neither is it clear how menopause affects the clinical presentation of IBD.

*Aims*. The objective of this pilot project was to study the interaction between menopause and clinical presentation of IBD.

*Methods*. Women age 30–65 with IBD were recruited from the University of Alberta Zeidler IBD clinic. Women completed a survey regarding their obstetric and medical history and medication use. Menopause experience was explored using the validated menopause-specific quality of life (MENQOL) questionnaire which uses 29 selected menopause symptoms in 4 categories (vasomotor, psychosocial, physical, sexual) to assess how much a woman is “bothered” by these symptoms on a scale from 1 (symptom not experienced) to 8 (extremely bothered by symptom).

Disease activity was determined by partial Mayo and modified HBI scores.

*Results*. Seventy-one women (45 with Crohn's Disease, 21 with Ulcerative colitis, one with indeterminate colitis, and 4 with undetermined diagnosis) were recruited comprising women in pre-menopausal (25), peri-, (15), and post-menopausal (31) stage. IBD onset at a young age correlated with early menopause (*R* ^2^ linear = 0.438). The average age of final menstruation in postmenopausal IBD patients excluding those with surgical menopause was low (44.2 ± 7.9 years) with a median of 46.6 compared to the median age of natural menopause for North American females (51.4). No difference was found in menopause symptom experience between CD and UC patients; however, MENQOL scores for the psychological, physical, and sexual categories were significantly higher in post-menopausal women with active disease (5.0 ± 1.7, 5.5 ± 1.0, 6.8 ± 0.8) compared to those in remission (2.6 ± 1.4, 3.7 ± 1.5, 2.8 ± 1.8). Disease activity appeared not to impact vasomotor symptoms including hot flashes and night sweats (4.4 ± 2.4 for women with flares versus 4.3.7 ± 2.4 for women in remission).

*Conclusions*. Our data demonstrate an effect of IBD onset and severity on the age of menopause onset and symptom perception. Future studies with higher enrolment numbers will address confounders such as medical history, medications, and lifestyle. Knowledge gained from this study will support women with IBD in health choices when coping with hormonal changes and symptoms during menopause.

*Funding Agencies: *WCHRI

# Sub article: A276: Histological and Endoscopic Assessment in Ulcerative Colitis: Results from the PURSUIT Trial

**Authors:** R. Strauss, B. Feagan, J. Colombel, C. Marano, S. Xu, L. Peyrin-Biroulet, G. de Hertogh, W. Sandborn

**Affiliations:** ^276.1^Janssen R&DLLCSpring HousePAUSA; ^276.2^Robarts Clinical Trials IncLondonONCanada; ^276.3^Mt Sinai Med CtrNYNYUSA; ^276.4^Janssen R & DLLCSpring HousePAUSA; ^276.5^Nancy U HospVandoeuvreFrance; ^276.6^U Hosp LeuvenLeuvenBelgium; ^276.7^UCSDLa JollaCAUSA

*Background*. Histologic assessment may provide an additional & potentially more accurate measurement of disease activity than endoscopy.

*Aims*. Assess features of the Geboes histological score associated with endoscopic healing.

*Methods*. PURSUIT-SC evaluated SC GLM induction therapy. Pts with Mayo scores of 6–12 inclusive, including endoscopic subscore >2 were rand to PBO/PBO, GLM 100 mg/50 mg, GLM 200 mg/100 mg, or GLM 400 mg/200 mg at wks 0 & 2. At wks 0 & 6, pts had two adjacent biopsies (bx) specimens obtained within 15–20 cm of the anal verge. One blinded pathologist reviewed each bx using Geboes score to assess histological features of structural change only, chronic inflammatory infiltrate, lamina propria eosinophils, lamina propria neutrophils, neutrophils in the epithelium, crypt destruction, & erosions & ulcers. Endoscopy assessments were made using local Mayo endoscopic subscore; subscores of 0 or 1 were defined as endoscopic healing.

*Results*. Among 89 pts in the substudy, 343 bx (166 paired from wk 0 & 6) were collected. Among all bx, endo scores of 0, 1, 2, or 3 were 4%, 20%, 45%, & 30%, respectively. Pts with endoscopy scores of 0 & 1 had very similar histological features. Histological features associated with endoscopic healing included: absence of histological evidence of ulceration & erosion, no evidence of crypt destruction, & only minimal neutrophil infiltration of the epithelia (<5%). For subsequent analysis, these pts were considered to have “histological healing (HH)”. “HH” by this definition does not mean complete histological normalization). “HH" rates at wk6 by Mayo endoscopy scores of 0,1, 2, & 3 were 100% (14/14) 68.1% (47/69), 19.9% (31/56), & 16.3% (17/104), respectively. Pts with “HH" had lower degrees of structural changes, chronic inflammation versus those who did not have “HH”. There was 88% concordance for “HH” between adjacent bx. “HH” was associated with lower rates of rectal bleeding & decreased stool frequency.

*Conclusions*. Absence of histological evidence of ulceration & erosion, no evidence of crypt destruction, & only minimal neutrophil infiltration of the epithelia (<5%) may constitute a definition of “HH” in UC. This definition is highly reproducible in adjacent bx specimens. Pts with histological healing are more likely to have endoscopic healing accompanied by reduced rectal bleeding & stool frequency.

*Funding Agencies: *Janssen R & D, LLC

# Sub article: A277: Circulating Inflammatory Cytokine Profiles in Children with Newly Diagnosed IBD: Determining Trends in Ulcerative Colitis and Crohn's Disease Patients

**Authors:** A. Foster, H. Gill, L. Yee, A. Marwaha, E. Dullaghan, K. Jacobson

**Affiliations:** ^277.1^BC Children's HospitalVancouverBCCanada

*Background*. It is hypothesized that a component of Inflammatory Bowel Disease (IBD) pathogenesis involves dysregulation of the immune system and an associated inflammatory response. Signalling proteins such as cytokines and chemokines are one mechanism responsible for driving this process. Limited knowledge is currently available on the cytokine profiles seen in pediatric IBD patients.

*Aims*. To define cytokine signatures in newly diagnosed pediatric IBD patients using Mesoscale technology.

*Methods*. This prospective study included patients who were newly diagnosed with IBD at BC Children's Hospital between January 2012 and June 2013. Patients were between 6 and 17 years-old and had either not yet started on therapy, or were within the first two weeks of initiation of a 5-ASA compound. Healthy controls were also recruited as a comparator group. Plasma levels of cytokines were measured using multiplex assays manufactured by MesoScale Discovery.

*Results*. Twenty-nine patients with IBD (16 Crohn's disease (CD), 13 ulcerative colitis (UC)) and 23 healthy controls (HC) were enrolled and completed cytokine testing. When compared to HC, UC patients had significant elevations in IL-8, IL-13 and IL-17 (*p* < 0.05). In addition, those with moderate to severe UC also had elevations of IL-5 and IL-23. In contrast, CD patients had significant differences in IFN-*γ*, IL-6, IL-8, IL-17 and TNF*α* when compared to HC (*p* < 0.05). When CD patients were analyzed according to severity, only those with mild disease differed with respect to TNF*α* and IL-17. A direct comparison of CD to UC cytokine profiles showed a significant dissimilarity between both IFN-*γ*, with higher levels in CD and IL-13 with higher levels in UC.

*Conclusions*. There is variation in plasma cytokine distribution patterns seen in newly diagnosed pediatric CD and UC patients along with healthy control subjects. Improved understanding of these differences may help explain the heterogeneous nature of IBD, and provide potential targets for therapy and inflammatory signatures for monitoring disease response to therapy. Further research is needed to characterize IBD biomarkers and understand their utility in the management of IBD.

*Funding Agencies: *CDRD, Genome BC

# Sub article: A278: Functional Studies of* C1orf106*, a Susceptibility Gene Associated in IBD, Highlight Its Potential Role in Intestinal Barrier Integrity

**Authors:** C. Lévesque, G. Charron, A. Alikashani, C. Beauchamp, L. Villeneuve, S. Foisy, G. Boucher, P. Goyette, J. Rioux

**Affiliations:** ^278.1^Université de MontréalMontreal Heart InstituteMontréalQCCanada; ^278.2^Montreal Heart InstituteMontrealQCCanada

*Background*. Inflammatory bowel diseases (IBD) involve chronic inflammation of the digestive tract and include ulcerative colitis (UC) and Crohn's disease (CD). IBD may result from defects in the homeostasis of immune system and/or intestinal epithelium. A dysfunction in the epithelial barrier may lead to a sustained immune response against the gut flora. Genome-wide association studies (GWAS) have identified ~200 loci in IBD; among these, the 1q32 region is associated with risk in both CD (*p* < 2 × 10^−11^) & UC (*p* < 6 × 10^−7^). This locus contains the gene* C1orf106*.

*Aims*. Our targeted re-sequencing study has identified a rare variant, Y333F (*p* = 0.009) in C1orf106. For this reason, we prioritized C1orf106 for functional analysis to determine its potential causality.

*Methods*. We profiled* C1orf106* expression using microarray analysis in human tissues and epithelial cell lines. As it is known that intestinal epithelial cells participate in, and are influenced by inflammatory processes, we also examined the impact of IL-1*β* treatment on the expression of* C1orf106* in Caco-2 epithelial cells. To provide further biological context, we generated LS174T colorectal cells that stably overexpress the Y333F alleles and performed protein stability assay as well as immunofluorescence to localize C1orf106.

*Results*. Our expression profiling of C1orf106 demonstrated that it is mostly expressed in small intestine and colon as well as in colonic epithelial cell lines. Furthermore, its expression is increased by 40% during differentiation of Caco-2 colonic epithelial cells into polarized epithelium. Moreover, while IL-1*β* treatment increased mRNA expression of IL-6 and IL-8 in Caco-2 cells, it had no effect on C1orf106 expression suggesting that it is not regulated by IL-1*β* pro-inflammatory cascade. We demonstrated that the rare variant can impact protein stability following cycloheximide treatment. C1orf106 is partially localized with ZO-1, used as a tight junction (TJ) marker. We did observe tighter colocalization with E-cadherin, a canonical marker for adherens junctions (AJ), typically located below the TJ complex.

*Conclusions*. AJ and TJ play an essential role in the establishment of epithelial barrier. Given the localization of C1orf106 at these regions suggests its possible implication in epithelial barrier homeostasis.

*Funding Agencies: *U.S. National Institute of Diabetes and Digestive and Kidney Diseases

# Sub article: A279: Model Gut Epithelia Lacking the NOD2 Protein Display Enhanced Intracellular Commensal* E*.* coli *in Response to Mitochondrial Stress Induced by Dinitrophenol: A Role for the Map Kinase ERK

**Authors:** A. Saxena, D. McKay

**Affiliations:** ^279.1^University of CalgaryCalgaryABCanada; ^279.2^Uni. CalgaryCalgaryABCanada

*Background*. Maintenance of the barrier function of the gut is energy dependent, and cohorts of patients with IBD can have increased permeability and display structural and functional evidence to dysfunctional mitochondria. Model epithelia treated with dinitrophenol (DNP; uncouples oxidative phosphorylation) have increased paracellular permeability and internalization of* E*.* coli*. The role, if any, of loss of the intracellular bacterial sensor NOD2—a genetic susceptibility trait for IBD—on the reduced barrier function evoked by DNP was unknown.

*Aims*. We hypothesized that the absence of NOD2 will leave transporting epithelium more susceptible to the barrier dysfunction induced by mitochondrial stress.

*Methods*. The human colonic T84 epithelial cell line was treated with* E*.* coli +/− *DNP and levels of NOD2 expression determined by qPCR and immunoblot. Epithelial internalization of inert beads, fluorescent dead* E*.* coli* and viable* E*.* coli*, as indices of transcellular permeability, was determined in normal epithelia and those in which NOD2 was knocked down (KD) by siRNA (transient) or shRNA (stable). The role of reactive oxygen species (ROS) and Erk was assessed pharmacologically by mitoTEMPO and U0126, respectively.

*Results*. NOD2 protein was undetectable in control cells and significantly increased in epithelia treated with DNP +/−* E*.* coli*. The non-specific internalization of beads and* E*.* coli* observed in normal T84 cells was increased in NOD2 KD cells, which showed increased phosph-ERK1/2 4 h post-treatment: U0126 resulted in reduced* E*.* coli* internalization in control and NOD2 KD cells. Crucially, NOD2 KD T84 cell monolayers pre-treated with DNP for 4 h and then exposed to* E*.* coli *for 1 h had enhanced ROS generation, and use of the mitochondrial-targeted antioxidant, mitoTEMPO, reduced the internalization of* E*.* coli* and inhibited the enhanced pERK signal.

*Conclusions*. Compromised epithelial barrier function due to perturbed mitochondrial function is exaggerated in the absence of NOD2. Mechanistically the increase in epithelial transcellular permeability was via mitochondria-derived ROS upstream of ERK1/2 signaling. Thus, the absence of NOD2 in transporting gut epithelia may enhance a barrier defect in IBD, observed as increased internalization and/or prolonged existence of intracellular bacteria in the context of metabolic-stress.

*Funding Agencies: *CIHR

# Sub article: A280: Topical Active Vitamin D Increases Host Suseptibility to Salmonella Induced Colitis

**Authors:** E. Bosman, N. Ryz, B. Vallance

**Affiliations:** ^280.1^BC Children's HospitalVancouverBCCanada

*Background*. Inflammatory bowel disease (IBD) patients that are also vitamin D deficient show increased disease activity, potentially reflecting exaggerated inflammatory responses to gut bacteria. Oral vitamin D supplementation studies report inconsistency in the ability to increase serum vitamin D levels in patients, likely due to inflammation at the site of absorption. Investigating other routes to deliver active vitamin D to IBD patients is needed to potentially alleviate the symptoms and gastrointestinal pathology of IBD. We chose topical administration, as the lipid soluble properties of vitamin D ensure diffusion through the skin layers after application.

*Aims*. This study demonstrates how topical application of active vitamin D affects gastrointestinal pathology in a* Salmonella*-induced-colitis model in mice.

*Methods*. C57Bl/6 mice were treated daily with 125 ng topical 1,25(OH)_2_D_3_ (calcitriol) to increase serum active vitamin D levels prior to and throughout an oral* Salmonella typhimurium ΔAroA* infection. Bacterial burdens and intestinal pathology were asessed on day 3 and day 6 post infection (p.i.).

*Results*. The calcitriol treated mice showed increased bacterial burdens in the cecum and at systemic sites at day 6 p.i. whereas this effect was not observed at day 3 p.i. Although pro-inflammatory cytokine (IFN*γ* & IL 6) expression was reduced after calcitriol treatment, the site of infection showed greater infiltration of neutrophils. Notably, following infection, mice lacking the T cell receptor *β* (TCR*β*) suffered a similar delay in pathogen clearance to that seen in C57B/6 mice treated with calcitriol, however upon their treatment with calcitriol, no further change in burdens was noted.

*Conclusions*. These results suggest that calcitriol interferes with the capacity of T cells to control intracellular* S*.* typhimurium* and alleviate gastrointestinal inflammation. Ongoing studies are examining the expression of the vitamin D receptor on T cells and granulocytes as well as exploring how topical calcitriol increases host susceptibility to* Salmonella *induced colitis.

*Funding Agencies: *NRC

# Sub article: A281: Capillary Flow Rates Are Increased in the Duodenum of Pediatric Ulcerative Colitis Patients

**Authors:** D. Zaidi, L. churchill, H. Huynh, M. Carroll, E. Wine

**Affiliations:** ^281.1^University of AlbertaEdomontonABCanada; ^281.2^University of albertaEdmontonABCanada; ^281.3^University of AlbertaEdmontonABCanada

*Background*. Pediatric Inflammatory Bowel Diseases (IBD), including Crohn disease (CD) and ulcerative colitis (UC), are globally on the rise. Extraintestinal manifestations in both CD and UC are indicative of systemic involvement in disease pathogenesis. While disease site typically differs between CD (small and large bowel) and UC (colorectal), duodenal involvement has also been reported in children with IBD. We have previously found increased epithelial gaps in uninflamed duodenum in UC patients using probe-based confocal laser endomicroscopy (pCLE), an optical imaging technique that enables visualization of the mucosal surface and microvascular circulation. Thus, assessing structural and circulatory changes in the duodenum even in the absence of inflammation is critical to better understand early stages of disease pathogenesis.

*Aims*. We hypothesized that children with IBD have changes in duodenal microvascular circulation (as detected by pCLE). The aim of our study was to assess changes in capillary flow rate in duodenal images obtained using pCLE during endoscopy in children with IBD and correlate them with disease activity and inflammatory markers.

*Methods*. pCLE was used to analyze capillary blood flow rate in non-inflamed duodenum of IBD and non-IBD patients. Fifty-six patients (33 non-IBD, 14 CD and 9 UC), between the ages of 3–18 were included in the study. Confocal imaging of the duodenum was conducted during endoscopy. Images of villi with visible blood vessels were captured as video sequences. Capillary flow rate was calculated using stringent criteria analyzing frame-by-frame as:

Distance travelled by blood cells/Duration of the sequence = capillary flow rate (*μ*m/ms)

*Results*. Duodenal capillary flow rate, blindly measured by 2 reviewers, was significantly higher in UC patients (0.78 ± 0.07 *μ*m/ms) as compared to non-IBD (0.59 ± 0.03 *μ*m/ms) and CD patients (0.65 ± 0.04 *μ*m/ms). There was no correlation between PUCAI, PCDAI, serum CRP, ESR, and capillary flow rate in CD and UC patients.

*Conclusions*. The study shows, for the first time, increased capillary blood flow in the duodenum of UC patients in the absence of localized inflammation, that was unrelated to inflammatory markers and disease activity, suggesting that vascular changes may not result from inflammation alone. Thus, early vascular changes can be assessed with pCLE during endoscopy. Further analysis at the molecular level will provide important insight into early vascular changes and their role in disease pathogenesis.

*Funding Agencies: *None

# Sub article: A282: The Colonic Mucosa in Crohn's Disease (CD): Evaluation by Confocal Laser Endomicroscopy (CLE)

**Authors:** S. Kaur, U. Dutta, E. Press, D. Armstrong

**Affiliations:** ^282.1^McMaster UniversityHamiltonONCanada; ^282.2^Postgraduate Institute of Medical Education & Research (PGIMER)ChandigarhIndia; ^282.3^Western UniversityLondonONCanada

*Background*. CD is characterised by patchy inflammation, a relapsing course, increased mucosal permeability and fibrosis. CLE colonoscopy combines standard imaging and in vivo cellular level mucosal imaging. There are few data on colonic mucosal structure assessed by fluorescein-based CLE in CD.

*Aims*. To assess colonic mucosal structure and fluorescein density in inflamed and non-inflamed mucosa in active and inactive CD.

*Methods*. CD patients and healthy colon cancer screening patients underwent CLE colonoscopy (Pentax ISC-1000 CLE, Tokyo, Japan) with conscious sedation. Fluorescein 10% (5 mL, IV) given on cecal intubation allowed CLE imaging. For active CD patients, a target (T) inflamed area and a matched (M), non-inflamed area were imaged by CLE and biopsied for routine histology, permeability and microbiome studies. For inactive CD patients and controls, imaging and biopsies were taken from non-inflamed T and M areas. CLE images were analysed (ImageJ; imagej.nih.gov/ij/) to determine crypt area, grey scale density, diameter and inter-cryptal distance (Figure 31 [Fig fig32] [Fig fig33]). Data (mean (SD)) were analysed by “*t*” test for paired data to compared T and M in all 3 subject groups.

*Results*. CLE image analyses (Table 48 [Table tab49] [Table tab50]) are shown for 14 active CD (7 F; mean age (SD): 37 (9) yrs) and 21 inactive CD patients (17 F; 48 (12)  yrs) and 6 healthy controls (3 F; 56 (16) yrs).

*Conclusions*. For active CD patients, crypt grey scale density was greater in inflamed (T) than in non-inflamed (M) areas with a trend for greater crypt diameter and area in T areas; T and M did not difffer for inactive CD patients or controls. Active CD patients were younger than inactive CD patients or controls.

Colonoscopically-identified inflammation in active CD shows greater fluorescein leakage, suggesting greater mucosal permeability compared with non-inflamed mucosa; inactive CD and normal colonic mucosa show no significant differences in permeability. Fluorescein leakage is not associated with structural changes in crypt size or density. Further research should assess the effect of patient age on colonic mucosal structure and permeability in Crohns' disease.

*Funding Agencies: *Supported, as an Investigator-Initiated Study, by AbbVie Canada

# Sub article: A283: Biological Profiles of Patients with Inflammatory Bowel Disease Based on Genetic Risk Factors

**Authors:** S. Ivison, J. Thompson Legault, A. Paradis, L. Odagiu, M. Goupil, G. Boucher, A. Forest, R. Kohen, A. Bitton, J. Rioux, C. Des Rosiers, M. Levings, S. Lesage

**Affiliations:** ^283.1^Child and Family Research InstituteVancouverBCCanada; ^283.2^Montreal Heart Research InstituteMontrealQCCanada; ^283.3^Hopital Maisonneuve*-*RosemontMontrealQCCanada; ^283.4^Montreal Heart InstituteMontrealQCCanada; ^283.5^McGill University Health CenterMontrealQCCanada; ^283.6^McGill UniversityMontrealQCCanada; ^283.7^Université de MontréalMontrealQCCanada; ^283.8^University of British ColumbiaVancouverBCCanada

*Background*. The inflammatory bowel diseases (IBD) are common (~1/150 Canadians) and genetically well-characterized diseases with 200 validated genetic risk factors. The IBD Genomic Medicine Consortium (iGenoMed) considered a list of ~295 candidate genes located within 163 of these loci, to guide the development of bioassays for predicting susceptibility to disease. Interestingly, many of the IBD candidate genes target different aspects of a limited number of biological pathways.

*Aims*. We postulate that identification of patient-specific alterations in these pathways will allow for a better understanding of disease mechanisms, heterogeneity of clinical course and the development of novel target-specific diagnostic tools and tailored therapies.

*Methods*. We therefore undertook a multisystems approach to query specific biological pathways that were selected based on the genetic risk factors and their corresponding biological pathways.

*Results*. In this systems approach, we developed and validated assays to characterize peripheral blood mononuclear cells at steady state or upon stimulation to specific ligands, to determine the serum profile of cytokines and chemokines using a multiplex ELISA system, and to assess various classes of plasma metabolites using mass spectrometry-based methods. Our preliminary results demonstrate that various biological pathways predicted by genetic risk factors vary between IBD patients and healthy donors. In this study, we have thus established the baseline conditions to investigate the relevance of using known genetic risk factors for designing bioassays interrogating biological pathways relevant to IBD. Our next step is to apply this multisystems approach to a pan-Canadian prospective cohort of patients undertaking treatment with biologics, namely anti-TNF and anti-integrins, for predicting the effectiveness of response to therapy.

*Conclusions*. In addition to predicting response to therapy, which would considerably reduce socioeconomic costs associated with biologics treatment, the results emanating from this work may yield useful biomarkers in predicting disease susceptibility as well as for identifying patient-specific alterations to guide personalized treatment.

*Funding Agencies: *CCC, CIHR, Genome Canada, Genome Québec, Genome British Columbia

# Sub article: A284: Improved Resolution of Genetic Association Signals in Inflammatory Bowel Diseases Reduces the List of Candidate Variants and Genes

**Authors:** G. Boucher, J. Rioux, I. (IIBDGC)

**Affiliations:** ^284.1^Montreal Heart InstituteMontrealQCCanada; ^284.2^Montreal Heart Institute and Université de MontréalMontrealQCCanada; ^284.3^IIBDGCMontrealQCCanada

*Background*. Over the last years, the collective efforts of scientists from the International Inflammatory Bowel Disease Genetics Consortium (IIBDGC) have led to the discovery and confirmation of 200 genetic loci associated to IBD, a chronic gastrointestinal inflammatory disorder. For most of these loci however, the association is not yet resolved to specific variants or genes.

*Aims*. This study aims to reduce the list of plausible candidate variants and genes for IBD using statistical fine-mapping methods and integration of functional annotation and expression quantitative trait loci (eQTL) datasets.

*Methods*. Statistically fine-mapping the association to single or few variants requires a large cohort and high-density genotyping. The IIBDGC thus genotyped 67,852 individuals on the Immunochip, a custom genotyping chip designed to provide high coverage of genetic variation within loci associated to autoimmune and inflammatory diseases. Three different Bayesian methods were applied to identify independent signals of association and attribute a posterior probability to each variant within the loci. Candidate variants were annotated for plausible or known functions.

*Results*. We identified 139 independent associations within 94 previously reported IBD loci. Of these, 45 associations were resolved to a single variant with a posterior probability greater than 50%, including 18 variants being causal with 95% certainty. These 45 variants are significantly enriched for variants with presumed or known functional impact. Specifically, 13 are protein-coding changes, 3 directly disrupt a transcription factor binding site and 10 show tissue specific epigenetic marks. The latter category shows enrichment in specific immune cells among associations stronger in CD and gut mucosa among associations stronger in UC. Based on a physical proximity of 50 kb from the fine-mapped signal, the list of candidate genes can now be reduced by a factor of two (from 669 to 331). Noteworthy, for 29 loci, a single gene is now included within 50 kb of the fine-mapped association signal.

*Conclusions*. Fine-mapping of associated genetic loci using large and densely genotyped cohorts can achieve a resolution to a single or a few potential causal variants, thus contributing to our understanding of the biological mechanisms underlying complex diseases such as IBD.

*Funding Agencies: *U.S. National Institute of Diabetes and Digestive and Kidney Diseases

# Sub article: A285: Changes in Lymphatic Vessel Morphology and Drainage Ability in Acute and Chronic Ileitis

**Authors:** S. Rehal, P. von der Weid

**Affiliations:** ^285.1^University of CalgaryCalgaryABCanada

*Background*. Crohn's disease (CD) is a chronic inflammatory disease, commonly presented as terminal ileitis. CD pathogenesis is multifactorial, with defects in adaptive immunity. Adaptive immunity relies on the trafficking of antigen from the gut to lymph nodes via mesenteric lymphatic vessels. Alterations in lymphatic physiology can modulate antigen transport to lymph nodes. Changes in the mucosal lymphatic system have been documented in biopsies and surgical resections from CD patients, specifically, lymphangiogenesis, lymphangiectasia and development of tertiary lymphoid organs (TLO). In contrast, very little is known about mesenteric lymphatic morphology and function.

*Aims*. Here, we use both acute and chronic murine models of ileitis to assess the status of the mesenteric lymphatic system draining the inflamed ileum.

*Methods*. Acute ileitis was induced by administration of 2.5% DSS in drinking water for 7 days. TNFΔARE mice were used at twenty weeks of age as the chronic ileitis model. Lymphangiectasia of mesenteric lymphatics was visually assessed by stereomicroscopy and lymphadenitis of mesenteric lymph nodes (MLNs) by histology. Real time PCR and confocal immunofluorescence were used to assess lymphangiogenesis and lymphoid neogenesis in the ileum, lymphatic vessels and MLNs targeting genes for VEGFR-3, PROX-1, Lyve-1 and LT*β*R and antibodies against smooth muscle *α*-actin, PROX-1, Podoplanin, LT*β*R, PNad1, VEGFR-3 and CCL21. Lymph flow was evaluated by fluorescent lipid tracking in gut, blood, feces and MLNs.

*Results*. TNFΔARE mice displayed larger damage to the ileum than DSS mice. Similarly, TNFΔARE mice revealed more severe lymphangiectasia, lymphandenitis and lymphangiogenesis. An extensive TLO formation was observed associated with lymphatic vessels in the mesentery of TNFΔARE mice. These TLOs histologically appear as lymphoid structures and express lymphangiogenic genes. Measurement of fluorescent lipid distribution suggests enhanced lymph flow in TNFΔARE mice, but reduced lymph flow in DSS mice.

*Conclusions*. Dynamic changes in lymphatic morphology and function occur during ileitis, which appear more important in chronic (TNFΔARE) than acute (DSS) model. Furthermore, novel description of mesenteric TLOs in TNFΔARE mice was made. These structures have a morphology very consistent with primitive lymph nodes. Lymph flow was shown to be compromised during DSS inflammation and inversely increased in TNFΔARE mice. The lymph flow changes might suggest an initial protective decrease in lymph flow to limit the inflammatory response, however in chronic inflammation, enhanced lymph flow might implicate that the lymphatic system is overwhelmed. These lymphatic changes can potentially modulate the dendritic cell trafficking dynamics during inflammation.

*Funding Agencies: *NIH

# Sub article: A286: Calculation of Fecal Calprotectin in Healthy and IBD Pregnancy

**Authors:** M. Morris, J. Jones, G. Zello, J. Alcorn, J. Pena-Sanchez, S. Fowler

**Affiliations:** ^286.1^College of Pharmacy and NutritionUniversity of SaskatchewanSaskatoonSKCanada; ^286.2^Dept of MedicineDalhousie UniversityHalifaxNSCanada; ^286.3^Dept of MedicineUniversity of SaskatchewanSaskatoonSKCanada

*Background*. Inflammatory Bowel Disease (IBD) has a peak incidence of 18–35 years, making management during pregnancy common. Although endoscopy can be safely performed in pregnancy, patients and clinicians prefer to avoid invasive procedures during this time, making the use of non-invasive surrogate markers desirable. Fecal calprotectin (FC) has been shown to distinguish IBD from irritable bowel syndrome and has a strong relationship with endoscopic disease activity in IBD. Traditional biomarkers (CRP, albumin) are known to be affected by pregnancy. The effect of pregnancy on FC is not known.

*Aims*. To establish FC reference values for healthy pregnant patients and to determine the feasibility of using FC to follow disease activity in pregnant patients with IBD.

*Methods*. Patients were recruited as part of a prospective, observational cohort. Healthy pregnant patients (≥18 years with no history of GI disorders) were recruited from an obstetrical clinic; pregnant patients with IBD were recruited from the IBD clinic at the University of Saskatchewan. Baseline demographic and medical information was recorded. FC was measured during each trimester (T1–3) and within 1 to 6 months post-partum (PP). FC was determined using a Quantum Blue® FC High Range Rapid Test (ALPCO Immunoassays, Salem, NH). Samples under the detection limit of 100 and 30 *μ*g/g where recorded as 50 and 15 *μ*g/g, respectively. Descriptive statistics were used to describe patient demographics. FC levels were expressed as medians with interquartile ranges (IQR). The Wilcoxon Matched-pairs Signed Rank test was used to identify median differences of FC at different time points. The Mann-Whitney *U* test was used to compare medians of FC between healthy and IBD patients.

*Results*. Forty-six healthy pregnant participants and 12 IBD patients were included. Median FC values of 15 *μ*g/g were found at all time points in the healthy pregnancy group (overall median 15 *μ*g/g, IQR 38). For IBD patients, median FC values of 477, 337, 469, and 439 *μ*g/g were found in T1–T3 and PP, respectively (overall median 416 *μ*g/g, IQR 516). Significant differences in FC were identified between healthy and IBD pregnant women (*p* < 0.001).

*Conclusions*. The determination of FC in healthy pregnancy has never been performed. This study established reference values for FC in pregnancy with normal values seen throughout all pregnancy trimesters. As opposed to traditional biomarkers used in IBD, such as CRP and albumin, FC does not appear to be affected by pregnancy, making it a useful biomarker to follow in pregnant IBD patients.

*Funding Agencies: *None

# Sub article: A287: Sacroiliitis Is Underdiagnosed in IBD and Associated with Male Gender, History of Arthritis and Non-Penetrating Crohn's Disease

**Authors:** N. Li, O. Kelly, J. Chan, R. Inman, M. Silverberg

**Affiliations:** ^287.1^Mount Sinai HospitalTorontoONCanada; ^287.2^Arthritis Centre of ExcellenceTorontoONCanada

*Background*. Sacroiliitis, an inflammatory joint condition associated with ankylosing spondylitis is common in patients with inflammatory bowel disease (IBD). Despite growing awareness, it often goes undiagnosed. Few data indicate what IBD features should heighten suspicion for this extraintestinal manifestation (EIM). Computed tomography (CT) imaging has been suggested as the imaging modality of choice for diagnosis of sacroiliitis.

*Aims*. To assess prevalence of sacroiliitis in IBD patients undergoing abdomino-pelvic CTs and determine associations between IBD disease features and sacroiliitis.

*Methods*. IBD patients who had undergone abdomino-pelvic CT (any indication; 2006–2015) were identified. Using a standardized CT scoring system, sacroiliitis was confirmed. Two blinded readers scored scans using two models: (1) ankylosis or ≥3 erosions; (2) ankylosis, ≥3 erosions, ≥0.5 cm iliac sclerosis, or ≥0.3 cm sacral sclerosis. Assuming 25% prevalence of sacroiliitis in IBD, a minimum sample of 288 patients was required. IBD clinical and endoscopic activity scoring was performed. Clinical activity was defined as Harvey Bradshaw Index (HBI) >4, Mayo > 2 or documentation of activity by the attending physician. Endoscopic activity was defined as SES-CD >4 or Mayo subscore > 1. Lab parameters, age, IBD symptoms, behavior, location, smoking, medications, history of arthritis, arthralgia and other EIMS were reviewed. Comparisons were made between patients with and without sacroiliitis.

*Results*. 316 patients were included (50.3% male; 74% Crohn's disease (CD)). Using CT scoring, 49 patients (15.5%) were diagnosed with sacroiliitis. Radiologists commented on sacroiliitis in 33% of those cases. 5 of 49 patients had ever been referred to a rheumatologist. 33 of 49 patients identified as sacroiliitis had abdominal X-rays; 64% of whom fulfilled the imaging component of the modified New York criteria for ankylosing spondylitis. There was no significant difference in prevalence of sacroiliitis between CD and ulcerative colitis (UC). Sacroiliitis was associated with male gender (63.3% versus 47.9%, OR 1.8 (1.01–3.5) *p* = 0.04), previous history of arthralgia/arthritis (41% versus 12%, OR 4.7 (2.2–9.9), *p* < 0.0001) and pain as a predominant IBD symptom (77.7% versus 56.9%, *p* = 0.03). In CD, absence of fistulizing disease on CT was associated with sacroiliitis (*p* = 0.02). Osteoporosis was more common in those with sacroiliitis (*p* = 0.15). Disease activity, location, lab parameters, smoking or medication were not associated.

*Conclusions*. Sacroiliitis is underdiagnosed in IBD and associated with male gender, previous arthritis and non-penetrating CD. The data support use of targeted low dose CT for screening in high risk patients.

*Funding Agencies: *CAG, CIHR

# Sub article: A288: Therapeutic Drug Monitoring for Infliximab in the Public Health Care System: The Quebec Experience

**Authors:** L. Rioux, G. Jobin, G. Aumais, J. Lachance, M. Provençal, R. Robitaille

**Affiliations:** ^288.1^Hôpital Maisonneuve-RosemontMontréalQCCanada; ^288.2^Hôpital Maisonneuve-RosemontMontrealQCCanada

*Background*. Inflammatory bowel diseases (IBD) such as Crohn's disease and ulcerative colitis can be treated with infliximab (IFX), a chimeric monoclonal human-mouse antibody which inhibits the cytokine TNF-alpha. The results of therapeutic drug monitoring (TDM) for IFX and the detection of antibodies to infliximab (ATI) have somewhat recently come to the forefront as essential tools in IFX treatment follow-up. As supporting proof, in February 2014, the INESSS (Quebec National Institute for Health Service Excellence) recommended these tools be published in the Quebec Index of Medical Biology Procedures while also mandating the Maisonneuve-Rosemont Hospital (HMR) to use ELISA (enzyme-linked immunosorbent assay) for the detection of concomitant IFX and ATI.

*Aims*. Portray the use of IFX TDM within the Quebec public healthcare system and describe service provision at the HMR Biochemistry Division by means of performance indicators.

*Methods*. Datamining within the HMR laboratory information system for the period spanning from June 1st, 2014 through to May 31st, 2015.

*Results*. After one year, 4884 concomitant IFX and ATI tests had been carried out in Quebec. Almost every provincial administrative health territory requested that tests be done, with the exception of some Northern Quebec Regions (10, 17 and 18). The highest-volume users are, in order: Montreal (1644 tests), Montérégie (610 tests) and Capitale-Nationale (606 tests). The number of tests constantly rose on a monthly basis to reach a total of 696 tests altogether in May 2015. The average turnaround time, defined as the timeframe elapsing from the laboratory's receipt of the sample to the time the result becomes available, was 14 days. Furthermore, only 24% of IFX test results fell into the therapeutic range (3–7 *μ*g/mL) while 44% of IFX test results fell into the sub-therapeutic range (3 *μ*g/mL). In the absence of IFX, where the method of ATI detection allows for free ATI detection alone, 37% of the results were negative for ATI (5 AU/mL) while 37% of the results were considered weakly positive (5–130 AU/mL) and 24% strongly positive (>130 AU/mL).

*Conclusions*. These results confirm the relevance of using IFX TDM as a follow-up to IBD treatments. In addition, TDM is predominantly employed by doctors who practice in the vicinity of a major university medical center. This data could benefit from further study, conjointly with a better characterization of the reasons for prescription, in order to elaborate clinical practice guidelines on TDM for IFX.

*Funding Agencies: *None

# Sub article: A289: Elevated Fecal Calprotectin in the Second or Third Trimester of Pregnancy Predicts Preterm Delivery in Women with Inlfammatory Bowel Disease

**Authors:** K. Smith, J. Bal, L. Ambrosio, R. Foshaug, R. Fedorak, V. Huang

**Affiliations:** ^289.1^University of AlbertaEdmontonABCanada; ^289.2^Los Alamos National LaboratoryEdmontonABCanada

*Background*. Inflammatory Bowel Diseases (IBD) are a group of chronic diseases, including Crohn's disease (CD) and ulcerative colitis (UC), affecting women in their reproductive years. Women with IBD, especially with active disease, during pregnancy are at increased risk of complications such as preterm delivery. It is difficult to confirm active IBD during pregnancy as pregnant women often have gastrointestinal symptoms and serum biomarkers of inflammation (e.g., C-reactive protein) can increase due to pregnancy. Fecal calprotectin (FCP), a biomarker of gastrointestinal inflammation, is known to correlate with active IBD. It is unknown whether FCP can predict preterm delivery in women with IBD.

*Aims*. To evaluate whether FCP measured in the second or third trimester of pregnancy in women with IBD can predict preterm delivery.

*Methods*. Pregnant IBD patients (18–45 yrs) from our Pregnancy in IBD research clinic were recruited to complete clinical disease activity scores (partial Mayo (pMayo) for UC, modified Harvey Bradshaw index (mHBI) for CD), and provide a stool sample after 13.0 weeks of gestation. Clinically active disease was defined by a pMayo score ≥2 or mHBI score ≥5. The stool was analyzed for FCP using the Quantum Blue High Range Reader. Elevated FCP was defined by FCP ≥200 *μ*g/g. Delivery outcome was categorized as preterm delivery (<37.0 wks) versus full term (≥37.0 wks). The median FCP was compared between groups by delivery outcome.

*Results*. A total of 18 women (UC = 15, CD = 3) provided all clinical and biological samples for a total of 25 study visits. The median gestational age at study visit was 26.0 (IQR: 19.0–32.0) wks. Out of the 25 visits, 14 showed elevated FCP ≥200 *μ*g/g. Women were in clinical remission at 16 of the 25 visits, yet had elevated FCP at 7 (43.7%) of these 16 visits. Women had an elevated FCP at 14 of the 25 visits, but had clinically active disease at only 7 (50%) of these 14 visits. Two women delivered preterm at 36.0 weeks and 36.4 weeks; they had significantly higher FCP compared to those who delivered full term (>1800 *μ*g/g versus 194.0 (IQR: 99.0–592.0) *μ*g/g, *p* = 0.039). An elevated FCP predicted preterm delivery in 29% of cases (4 out of 14 visits with elevated FCP versus 0 out of 11 visits with normal FCP).

*Conclusions*. Elevated fecal calprotectin measured after 13.0 weeks gestational age (2nd or 3rd trimester) in women with IBD predicts preterm delivery. Since clinical disease activity scores do not consistently reflect objectively active IBD during pregnancy, assessment of disease activity using a biomarker such as fecal calprotectin could be used to risk-stratify women with IBD during pregnancy.

*Funding Agencies: *None

# Sub article: A290: Crohn's Disease: A Retrospective Cohort Study of the Use of Infliximab and Patient Response to Infliximab in a Large Teaching Hospital

**Authors:** L. Rioux, M. Gauthier-Richard

**Affiliations:** ^290.1^Hôpital Maisonneuve-RosemontMontréalQCCanada

*Background*.* Context*: Crohn's disease (CD) is a common and chronic inflammatory disease of the intestine for which long-term treatment presents challenges. Anti-TNF-*α* biological agents such as infliximab (IFX) are currently the most effective treatment option for moderate to severe cases. Despite a strong initial response, over the course of treatment a substantial proportion of patients experience a decline in efficacy, undesirable side effects or allergic reactions.

*Aims*. To document the evolution of Hôpital Maisonneuve-Rosemont (HMR) patients suffering from CD and undergoing IFX-centred treatments in an attempt to identify factors that influence patient response and increase treatment efficacy.

*Methods and Data Analysis*. A thorough patient-file review was carried out to track patient evolution. Statistical analysis was used to analyze influencing factors.

*Results*. To date, 182 patient files contained sufficient data to merit effective analysis. 94.5% of patients with CD on IFX treatment plans responded well to the induction phase. During maintenance therapy, 54.4% of patients experienced a decline in efficacy requiring one to several adjustments in dose or in frequency. At the time of the file review, 81.3% were still receiving IFX treatment with 43.2% undergoing treatment for 5 years or more. Patients undergoing combination therapy with immunosuppressant drugs (azathioprine or 6-mercaptopurine) persisted with IFX treatments for a longer duration (75.6 months) than those in monotherapy (45.2 months, *p* < 0.001).

*Conclusions*. At HMR, IFX has been successfully administered as a long-term treatment. Statistical analysis shows that concurrent immunosuppression therapy lengthens the duration of IFX treatment efficacy. There is no statistical evidence to suggest that gender, the disease's location or that patient age upon commencing treatment has any influence on patient response to IFX.

*Funding Agencies: *None

# Sub article: A291: Can Gastroenterologists Rely on Fecal Calprotectin in Lieu of More Invasive Testing or CRP in Management of IBD?

**Authors:** C. Bernstein, H. Singh, W. El-Matary, E. Abej

**Affiliations:** ^291.1^University of ManitobaWinnipegMBCanada

*Background*. Fecal calprotectin (FCAL) has emerged as a popular biomarker of intestinal inflammation in IBD.

*Aims*. The aim of our study was to determine the correlation of FCAL to traditional confirmatory tests and other biochemical inflammatory markers, and the impact of FCAL results on decision-making in management of IBD patients.

*Methods*. 179 patients with IBD (64 children (ages 4–17) and 115 adults) attending the clinics of 3 gastroenterologists were asked to bring in a stool sample for FCAL testing. The FCAL test results were correlated with serum albumin (alb), hemoglobin (Hg) and CRP done within 2 weeks of collecting stool samples for FCAL testing and with diagnostic imaging (computed tomography enterography (CTE) or magnetic resonance enterography (MRE)), or colonoscopy or flexible sigmoidoscopy, done within a month. The choice of blood testing and imaging was left to the physicians' discretion. We also assessed how the FCAL results were used in clinical decision-making in terms of further investigations or change in therapy. FCAL was done using the Quantum Blue® Lateral Flow Reader and within 24 hr of stool collection. FCAL value of 250 mcg/g of stool used as cut off point of positive test. The impact of FCAL results on patient management was assessed by a questionnaire given to the participating gastroenterologists.

*Results*. 139 stool samples (78%) were returned. 19 persons underwent CTE or MRE, 24 underwent colonoscopy or flexible sigmoidoscopy, 113 had alb, 108 had Hg, and 101 had CRP. There was no significant difference for FCAL results for those with active disease by CTE or MRE (*p* = 0.24), colonoscopy or flexible sigmoidoscopy (*p* = 0.4), anemia (*p* = 0.29) or elevated CRP (*p* = 0.25). However, persons with low alb (<34 g/L, *n* = 16) were more likely to have elevated FCAL (87.5%) than persons with normal serum albumin (*n* = 97, 55%, *p* = 0.02, relative risk 1.6 (95% CI 1.2, 2.1)). Based on a positive FCAL test clinicians made a change in therapy or investigations in 65 (88%). On the other hand, based on a negative FCAL clinicians made no change in therapy or further investigations in 51 (78%).

*Conclusions*. The minority of patients in this cohort had imaging, however FCAL results were not significantly associated with radiological or endoscopic evidence of disease activity. Among alb, Hg and CRP, only a low alb was associated with an elevated FCAL. Gastroenterologists made clinical decisions based on FCAL although when imaging/endoscopy was undertaken the association with FCAL was poor. While previous studies have shown a correlation between FCAL and disease activity, our study suggests that FCAL may not be able to replace direct investigations of disease activity in usual clinical practice. In addition, importantly our study also demonstrates FCAL and CRP cannot be used interchangeably in usual clinical practice.

*Funding Agencies: *None

# Sub article: A292: The Utility of Infliximab Therapeutic Drug Monitoring among Patients with Inflammatory Bowel Disease and Concerns for Loss of Response: A Retrospective Study

**Authors:** C. Shuster, R. Mitchell, N. Shahidi, C. Galorport, M. DeMarco, G. Rosenfeld, B. Bressler, R. Enns

**Affiliations:** ^292.1^St. Paul's HospitalDivision of GastroenterologyDepartment of MedicineUniversity of British ColumbiaVancouverBCCanada; ^292.2^Department of Pathology and Laboratory MedicineUniversity of British ColumbiaVancouverBCCanada

*Background*. With the emergence of infliximab (IFX) therapeutic drug monitoring (TDM), objective decision making among patients with concerns for loss of response is now more readily available. However, questions still remain about whether the availability of TDM results leads to improved outcomes.

*Aims*. We sought to evaluate the impact of IFX TDM on outcomes among patients with inflammatory bowel disease (IBD) and concerns for loss of response.

*Methods*. Between Nov-2014 to Jun-2015 patients ≥ 19 years of age with IBD who had IFX TDM due to concerns for loss of response were considered for inclusion. A range of 3–7 mg/mL was used to define the IFX therapeutic window. For specimens with IFX <3 mg/mL, anti-drug antibody (ADA) testing was performed with >8 mg/mL indicating an actionable amount of ADA present. Patients were grouped by TDM results. Group 1-high ADA/low IFX levels; Group 2-low ADA/low IFX levels; Group 3-low ADA/therapeutic IFX levels. Subsequently, appropriate change in management was assessed: Group 1-switch to an alternative anti-tumor necrosis factor agent; Group 2-IFX dose optimization; Group 3-switch to an out-of-class biologic agent. Our primary outcome was remission (Harvey Bradshaw Index ≤ 4 for Crohn's disease (CD) or partial Mayo Score ≤ 2 with no individual sub-score > 1 for ulcerative colitis (UC) and C-reactive protein < 5 mg/L) at 6 months after TDM.

*Results*. 41 patients (15 UC, 26 CD) who had 6-month follow-up data available were included. Mean age was 38 years (range 20–81) with 54% being male. After TDM 2, 12, and 27 patients were in groups 1, 2 and 3 respectively. Of these, 34% underwent an appropriate change in therapy with groups 1 (100%) and 2 (75%) having high adherence. Conversely, only 7% of patients in group 3 underwent an appropriate change in management, with the remainder undergoing dose optimization (26%) or no change in biologic therapy (67%). At 6 months, 51% of patients had achieved remission. More patients who underwent an appropriate change in therapy achieved remission (57% versus 48%; *p* = 0.585); this did not reach statistical significance.

*Conclusions*. In this preliminary analysis, appropriate interpretation of TDM did not increase the likelihood of remission. However, further evaluation is needed, specifically, among those with low ADA/therapeutic IFX levels as very few patients underwent an appropriate change in therapy. This may reflect the lack of readily available out-of-class options.

*Funding Agencies: *None

# Sub article: A293: Canadian Patient and Caregiver Perspectives on Subsequent Entry Biologics for Inflammatory Bowel Disease

**Authors:** G. Attara, R. Bailey, B. Bressler, J. Marshall, R. Panaccione, G. Aumais

**Affiliations:** ^293.1^Gastrointestinal SocietyVancouverBCCanada; ^293.2^Univ of AlbertaEdmontonABCanada; ^293.3^Pacific Gastroenterology AssociatesVancouverBCCanada; ^293.4^McMaster University Medical CentreHamiltonONCanada; ^293.5^University of CalgaryCalgaryABCanada; ^293.6^Université de MontréalMontréalQCCanada

*Background*. Biologic medications have revolutionized the treatment of inflammatory bowel disease (IBD). Subsequent Entry Biologics (SEBs) are not identical in structure, therapeutic equivalence, or approved indications, but are biosimilar to an original biologic medication. Given the recent presence of SEBs in Canada, there might be implications for IBD patients and/or caregivers.

*Aims*. To understand the perspectives of IBD patients and caregivers regarding SEBs and how Canadian drug programs will manage these products.

*Methods*. During early 2015, the Gastrointestinal Society hosted a survey on its two websites: www.badgut.org and www.mauxdeventre.org. The physicians included in this project shared the links with their patients. To qualify, survey participants confirmed they either had IBD or were a caregiver of a person with IBD. Questions included demographic information, disease characteristics, as well as their understanding and opinions regarding SEBs, including the possibility of switching from an innovator biologic to a biosimilar.

*Results*. 423 respondents: 317 English, 106 French. 68% had Crohn's disease, 30% ulcerative colitis, 2% indeterminate IBD. 77% had at least a basic understanding of biologics and were currently prescribed an originator biologic (Remicade®, Humira®, or Simponi®). 76% had heard about SEBs. Majority of patients selected cost then manufacturing process, as the top ways SEBs might differ from originator biologics. 52% believed that having the same international non-proprietary name (INN) implied that patients could safely switch between the products during a course of treatment and expect the same effectiveness and safety, even though Health Canada states they are not interchangeable. 95% of those surveyed said that it is important their physician, together with them, have sole authority to decide the most suitable biologic medication to treat their disease. The most important considerations in choosing SEB treatment for patients were safety, efficacy, and that both the originator and biosimilar have identical review and approval processes. 96% of respondents were concerned about limited medication choice during induction and/or maintenance therapy.

*Conclusions*. IBD patients who responded to our survey were quite familiar with SEBs, although they expressed high concerns around safety, efficacy, and regulatory process. The patient choice directive is very strong and shows the need for open dialogue among patients, physicians, manufacturers, and regulatory bodies for the safe introduction of SEBs into the marketplace.

*Funding Agencies: *Janssen Inc.

# Sub article: A294: Can Fecal Calprotectin Predict the Future?

**Authors:** L. Kwapisz, M. Mosli, N. Chande, B. Yan, M. Beaton, J. Micsko, W. Barnett, K. Bax, T. Ponich, J. Howard, A. Tirolese, R. Lannigan, J. Gregor

**Affiliations:** ^294.1^UWOWhitbyONCanada; ^294.2^Western UniversityLondonONCanada; ^294.3^The University of Western OntarioLondonONCanada; ^294.4^London Health Sciences CentreLondonONCanada

*Background*. Fecal calprotectin (FC) is a marker of bowel inflammation that is currently used to diagnose and evaluate inflammatory bowel disease (IBD). In a previously reported prospective diagnostic cohort study, rapid FC testing was helpful in identifying patients with active IBD (Kwapisz et al., Saudi J Gastro 2015). The same cohort was then followed up for one year and re-evaluated.

*Aims*. The aim of this study is to assess if FC levels could predict future bowel inflammation manifesting as IBD relapse requiring escalation of therapy or diagnosis of IBD in patients previously diagnosed with IBS at baseline.

*Methods*. 126 consecutive adult patients who presented to outpatient clinics with lower gastrointestinal symptoms provided high range FC samples within 4 weeks of their baseline scheduled endoscopic assessment. All patients were followed up for at least one year and monitored clinically for any change in symptomatology, escalation of therapy, or development of IBD, confirmed endoscopically. IBD flare-ups required endoscopic confirmation. Escalation of therapy included any intensification in dosage, frequency, or addition of new therapies for IBD such as: 5-ASA agents, corticosteroids, immunosuppressants, TNF antagonists, leukocyte trafficking inhibitors, investigational drugs, or need for surgery. Diagnosis of IBD was based on conventional clinical, endoscopic and histologic criteria.

*Results*. 126 patients, of whom 66 were females, were included with a mean age of 44.4 years (±16.7). At baseline, 72 had known IBD and active endoscopic evidence of disease activity. Utilizing an FC cut-off of 100 *μ*g/g, 66% (33/50) of patients with endoscopically active IBD went on to have escalation in therapy within one year. Among those with FC levels <100 *μ*g/g, only 18% (4/22) required an increase in therapy. Thirty three percent (2/6) of patients with quiescent IBD at baseline who had FC levels >100 *μ*g/g, required escalation in therapy due to disease flare up, whereas none of those with FC levels <100 *μ*g/g (0/12) needed change in therapy. Lastly, for patients who did not have IBD and had normal endoscopic evaluation with an FC level >100 *μ*g/g, none (0/17) were diagnosed with IBD within one year.

*Conclusions*. Elevated FC concentrations in the absence of endoscopically visible IBD can predict future relapses requiring escalation of therapy in those known to have IBD, and future development of IBD in IBS patients.

*Funding Agencies: *None

# Sub article: A295: The Impact of Private Insurance Access on Inflammatory Bowel Disease-Related Emergency Department Use

**Authors:** N. Bollegala, G. Nguyen

**Affiliations:** ^295.1^University of TorontoTorontoONCanada; ^295.2^Mount Sinai HospitalUniversity of TorontoTorontoONCanada

*Background*. Inflammatory Bowel Disease (IBD)-related Emergency Department (ED) visits are a costly component to current healthcare expenditures. Patients who are discharged directly from the ED (aka “treat and release” ED visits) present an area for quality improvement.

*Aims*. To determine the impact of private insurance on IBD-related “treat and release” ED visits. The secondary outcome was cost per IBD-relate ED visit.

*Methods*. A retrospective cohort study was performed on the 2006 Nationwide Emergency Department Sample (NEDS). Comparisons were made between patients with access to private insurance versus those without. Continuous variables were compared using the Student's *t*-test. Categorical variables were compared using the chi-square. Alpha (type 1 error; two-tailed) was set at <0.05. Predictor variables were selected a priori for the primary outcome. A univariable selection strategy was used for the secondary outcome with split sample validation. Multivariable survey weighted logistic and linear regression models with clustering by hospital were created for the primary and secondary outcomes, respectively. Model fit and assumptions were assessed.

*Results*. 19,324 patients were included in the stratified analytic sample. 9,272 (47.98%) patients reported private insurance as their primary payment method. 10,052 (52.02%) patients reported an alternative payment form. The private insurance group was statistically younger, less likely to reside in an urban setting and had more representation within the highest income quartile. The OR of a “treat and release” ED visit was 1.47 (95% CI 1.34–1.62) for no private insurance compared to private insurance. On average, the cost per ED visit of patients without private insurance was $214.80 ± 48.48, *p* < 0.001 less than those with private insurance.

*Conclusions*. Lack of private insurance is an important predictor of IBD-related “treat and release” ED visits.

*Funding Agencies: *None

# Sub article: A296: Cost-Utility Analysis Shows Adalimumab Is Cost-Effective for the Management of Ulcerative Colitis

**Authors:** C. Beilman, T. Nguyen, V. Ung, C. Ma, K. Wong, K. Kroeker, T. Lee, H. Wang, A. Ohinmaa, P. Jacobs, B. Halloran, R. Fedorak

**Affiliations:** ^296.1^University of AlbertaEdmontonABCanada; ^296.2^Institute of Health EconomicsEdmontonABCanada

*Background*. Adalimumab (ADA) is effective for the induction and maintenance of remission in patients with moderate to severe ulcerative colitis (UC). Currently, biologic therapies are used in cases where patients fail or are intolerant to conventional medical therapies. If biologic therapies are not available, patients often choose to remain in an unwell state rather than undergo colectomy.

*Aims*. The aim of the study was to evaluate the real-life cost-effectiveness of ADA in patients with moderate to severe UC who are refractory to thiopurines or have become corticosteroid dependent.

*Methods*. A previously established Markov model was used to simulate disease progression of patients with moderate to severe UC in situations where ADA is readily available compared to situations when it is unavailable. Utility scores and transition probabilities between health states were determined by using data from randomized controlled trials and real-life rates published by expert inflammatory bowel disease centers. Healthcare costs were obtained from the Ontario Case Costing Initiative and the Alberta Health Schedule of Medical Benefits. An exploratory analysis examining dose escalation was conducted due to the high rate of patients who undergo dose escalation as a result of loss of response to ADA.

*Results*. The mean induction rate of ADA was 87%, which is contrasted to the 34% of patients who responded to corticosteroids and the 57.0% who remained “unwell" despite treatment. Complications which necessitated the cessation of a treatment occurred in approximately 8% of the patients on ADA and 3% of the patients on chronic corticosteroids. The incremental cost-effectiveness ratios (ICER) for readily available ADA treatment of UC were $40,000 and $59,000 per quality-adjusted life year (QALY), compared with ongoing medical therapy in an unwell state, at 5-year and 10-year treatment time horizons, respectively. The exploratory analysis revealed ICERs associated with ADA dose escalation to be $77,000 and $102,000 per QALY at 5-year and 10-year treatment time horizons, respectively.

*Conclusions*. Considering real-life patient preferences to avoid colectomy, ADA is cost-effective according to a willingness-to-pay threshold of $80,000 per QALY for treatment of moderate to severe UC. Dose escalation will increase these costs.

*Funding Agencies: *Centre of Excellence for Gastrointestinal Inflammation and Immunity Research (CEGIIR)

# Sub article: A297: Early Initiation of Anti-TNF Therapy Does Not Reduce Colectomy or Hospitalization Rates in Ulcerative Colitis: A Retrospective Cohort Study

**Authors:** C. Ma, C. Beilman, V. Huang, K. Wong, K. Kroeker, L. Dieleman, B. Halloran, R. Fedorak

**Affiliations:** ^297.1^University of AlbertaEdmontonABCanada

*Background*. Biologic agents targeting tumor necrosis factor alpha are effective in the management of ulcerative colitis (UC), but their use is often postponed until after failure of other treatment modalities.

*Aims*. To determine if earlier treatment with infliximab or adalimumab improves clinical and surgical outcomes in UC patients.

*Methods*. A retrospective cohort study was conducted evaluating UC outpatients on a maintenance regimen with infliximab or adalimumab from 2003–2014. Patients were stratified by time to first anti-TNF exposure; early initiation was defined as starting treatment within three years of diagnosis. Primary outcomes were colectomy, UC-related hospitalization, and clinical secondary loss of response. Kaplan-Meier analysis was used to assess time to the primary outcomes.

*Results*. 115 UC patients were included (78 infliximab, 37 adalimumab). Median follow-up was 175.6 weeks (IQR 72.4–228.4 weeks). Fifty-seven (49.6%) patients received anti-TNF therapy within three years of diagnosis; median time to treatment in this group was 38.1 weeks compared to 414.0 weeks in the late initiator cohort (*p* < 0.0001). Patients requiring early anti-TNF therapy had more severe endoscopic disease at induction (mean Mayo endoscopy sub-score 2.46 versus 1.86, *p* < 0.001) and trended towards increased risk of colectomy (17.5% versus 8.6%, *p* = 0.16) and UC-related hospitalization (43.9% versus 27.6%, *p* = 0.07). In subgroup analysis excluding patients with severe Mayo 3 endoscopic disease, there were no differences in hospitalization, secondary loss of response, or colectomy rates.

*Conclusions*. Anti-TNF therapy is initiated earlier in patients with severe UC but early treatment within three years of diagnosis with infliximab or adalimumab does not delay or prevent hospitalization or colectomy. These findings are in contrast to those for early treatment for Crohn's disease (Ma et al., IBD in press).

*Funding Agencies: *None

# Sub article: A298: Microarray Analysis of Crohn's Disease and Correlation with Traditional Clinical and Histologic Features

**Authors:** V. Jovanovic, J. Chang, A. Thiesen, R. Fedorak, P. Halloran, B. Halloran

**Affiliations:** ^298.1^University of AlbertaEdmontonABCanada; ^298.2^University of AlbertaEdmontonABCanada

*Background*. As a T cell-mediated disease of the gastrointestinal epithelium, Crohn's disease (CD) is likely to share pathogenic elements with other T cell-mediated inflammatory diseases. Recently we showed that ulcerative colitis manifested large-scale molecular disturbances that correlated with endoscopic and histologic features (IBD 20:2353, 2014).

*Aims*. We hypothesized that ileal CD would manifest similar molecular disturbances correlating with endocsopic and histologic features.

*Methods*. We studied 27 patients in 31 biopsies with ileal CD, characterizing the clinical, endoscopic and histological features and defined the mRNA phenotype using microarray analysis of ileal biopsies. We measured the expression of pathogenesis-based transcript sets (PBTs) previously published for ulcerative colitis representing effector T cells, macrophages, IFNG effects, and parenchymal injury-repair response and dedifferentiation (Table  1). The molecular features were then correlated with conventional assessments including clinical features (modified Harvey Bradshaw index (HBI), simple endoscopic score for CD (SES-CD), c-reactive protein, albumin) and histologic features (lamina propria neutrophilic and lymphoplasmacytic infiltrate, crypt abscess, ulcers present and crypt architectural distortion).

*Results*. CD ileal biopsies arranged by injury-repair score (IRRAT) manifested coordinate transcript changes with IFNG-induced transcripts (GRIT), macrophage transcripts (QCMAT), and injury-repair transcripts increasing while parenchymal transcripts (PT) decreased (Figure 34). Lymphoplasmacytic infiltrate was significantly correlated with IRRAT (*p* = 0.005) and negatively correlated with parenchymal transcript expression (*p* = 0.01). Neutrophilic lamina propria infiltrate (*p* = 0.03) and number of ulcers (*p* = 0.03) also correlated with IRRAT. No significant correlation was seen between the molecular features and the HBI (*p* = 0.5), SES-CD (*p* = 0.8) or CRP (0.2).

*Conclusions*. The molecular phenotype of CD manifests a large-scale coordinate disturbance similar to that in ulcerative colitis and other T cell-mediated diseases, reflecting changes in inflammatory cells and parenchymal elements and correlating with histologic assessment, especially the lymphoplasmacytic and neutrophilic lamina propria infiltrate, but not with the clinical and endoscopic features. While this may be related to CD in different stages of healing, it raises further questions about our clinical and endoscopic assessments of CD. Novel molecular systems for quantitating and staging the disease elements in the tissues in CD may add a significant new dimension to patient management beyond our current standards.

*Funding Agencies: *None

# Sub article: A299: The Within-Stool and Within-Day Variability of Fecal Calprotectin in Patients with Active Inflammatory Bowel Disease

**Authors:** L. Du, R. Foshaug, V. Huang, K. Kroeker, L. Dieleman, B. Halloran, K. Wong, R. Fedorak

**Affiliations:** ^299.1^University of AlbertaEdmontonABCanada

*Background*. Fecal calprotectin (FC) constitutes approximately 60% of neutrophil cytosolic proteins, and its measured level in feces is proportional to the degree of neutrophil migration into the bowel lumen. The use of FC as a non-invasive stool biomarker for differentiating IBD from IBS has been well validated, and there is a strong correlation between FC and the presence of endoscopic inflammatory lesions. However, recent studies have demonstrated within-day and between-day FC variability in patients with active IBD, possibly limiting the reliability of using a single sample for monitoring disease activity and guiding management.

*Aims*. To assess the within-stool and within-day variability of fecal calprotectin concentrations in IBD patients with clinically active disease.

*Methods*. This is a prospective observational study evaluating a cross-sectional cohort of IBD patients ≥18 years with clinically active disease based on their Harvey Bradshaw Index or Partial Mayo Score. Eligible patients were asked to collect three samples from each of three bowel movements (morning, afternoon, and evening). FC concentrations were measured by a rapid, quantitative point-of-care test using lateral flow technology (Quantum Blue®). For FC levels in different samples from the same bowel movement, an intraclass correlation coefficient was used to determine the test-retest reliability. One-way ANOVA was calculated to determine the effect of time of day on the level of fecal calprotectin.

*Results*. The overall within stool correlation coefficient was 0.93, demonstrating excellent test-retest reliability within samples. One-way ANOVA results of samples collected at different times of day ranged from *F* = 21.35 (*p* = 0.007) to *F* = 35.76 (*p* = 0.003), suggesting significantly higher FC levels in morning samples compared to evening samples.

*Conclusions*. The preliminary results of our study suggest that within a single bowel movement, fecal calprotectin levels are robustly reproducible. However, there appears to be significant variation in FC concentrations between different bowel movements within the same day, and this should be considered when interpreting FC results.

*Funding Agencies: *Centre of Excellence for Gastrointestinal Inflammation and Immunity Research

# Sub article: A300: A Retrospective Analysis on Anti-TNF Trough Level Monitoring in Inflammatory Bowel Disease Patients with a Loss of Response to Anti-TNF Therapy

**Authors:** M. Laghari, C. Prosser, K. Kroeker, L. Dieleman, B. Halloran, R. Fedorak, V. Huang

**Affiliations:** ^300.1^University of AlbertaEdmontonABCanada

*Background*. Infliximab (IFX) is effective in inducing and maintaining remission in patients with moderate to severe Inflammatory Bowel Disease (IBD). However, Secondary Loss of Response (LOR) during maintenance therapy is a major concern with often unknown etiology. Secondary LOR is often due to subtherapeutic drug levels and can theoretically be managed depending on the drug trough level. The therapeutic trough level varies both in the literature and clinically with some patients requiring higher levels to maintain remission.

*Aims*. Our aims are (i) to characterize therapeutic interventions based on IFX trough levels at the University of Alberta IBD Clinic and (ii) to evaluate whether these interventions have a response in IBD outpatients with a Secondary LOR to IFX.

*Methods*. We conducted a retrospective chart review for IBD characteristics and Anti-TNF history for adult IBD outpatients on maintenance IFX therapy who had IFX trough levels drawn between January 2013 and March 2015, at the University of Alberta IBD Clinic. We documented interventions at the time of Secondary LOR: Expectant management (no change in IFX); Dose intensification; Switching within class; Switching out of class. We set a “therapeutic" drug range of 3–10 mcg/mL, with <3 mcg/mL and >10 mcg/mL, sub and supratherapeutic respectively.

*Results*. 38 patients on maintenance IFX had drug trough levels (*n* = 57). Of these, 22 (38.6%) levels were for a Secondary LOR, of which 16 (73%) were therapeutic (median 5.45; IQR 2.63), 5 (23%) subtherapeutic (median 2.7; IQR 2) and 1 (4%) supratherapeutic (10.1 mcg/mL). In the subtherapeutic range, 4/5 (80%) had dose intensification with 75% responding, and 1/5 (20%) had no change to IFX but had some intervention (antibiotics, steroids, immunomodulators and/or 5-ASA) with a 100% response. In the therapeutic range, there was no change in 8/16 (50%) with an 87.5% response; Dose intensification in 7/16 (43.7%) with a 71.4% response and switch out of class in 1/16 (6%) with a 100% response. In the supratherapeutic range, 1/1 (100%) were switched out of class with a 100% response.

*Conclusions*. In IBD outpatients with a Secondary LOR to IFX, most physicians dose intensify if the patient has subtherapeutic levels and switch out of class if supratherapeutic, both with good response rates. In the “therapeutic” range however, the intervention is mixed, with almost half dose intensifying with approximately a two-third response rate, suggesting that the currently viewed therapeutic drug range might not be adequate, or even, more individual patient based.

*Funding Agencies: *None

# Sub article: A301: Comparison of Commercially Available Assays for Infliximab Concentrations and Antibodies to Infliximab with Assays Developed at Janssen and Used in Clinical Studies of Remicade® (Infliximab) in IBD Patients

**Authors:** J. Marini, J. Sendecki, F. Cornillie, J. Popp, M. Blank, S. Black, K. Chun, A. Gils, T. Van Stappen, L. Therien, D. Hamann, T. Rispens, G. Shankar

**Affiliations:** ^301.1^Janssen R & DLLCSpring HousePAUSA; ^301.2^MSD International GmbHKriensSwitzerland; ^301.3^Janssen Scientific AffairsLLCHorshamPAUSA; ^301.4^LabCorpBurlingtonNCUSA; ^301.5^KULeuvenBelgium; ^301.6^DynacareBramptonONCanada; ^301.7^SanquinAmsterdamNetherlands

*Background*. Infliximab (IFX) concentrations and antibodies (Ab) to IFX (ATI) titers can be informative in assessing IBD response.

*Aims*. In collaboration with KU Leuven, Sanquin, Dynacare and LabCorp, the accuracy and reliability of commercially available IFX and ATI assays were compared to assays used by Janssen for Remicade® (IFX) IBD studies.

*Methods*. Samples were prepared by Janssen and shipped to labs. KU Leuven (assay distributed by apDia and R-biopharm) and Dynacare used enzyme-linked immunosorbent assay (ELISA) to measure IFX and ATI. Sanquin used ELISA to measure IFX and a radioimmunoassay to measure ATI. LabCorp used electrochemiluminescence immunoassay (ECLIA) to measure IFX and ATI. Janssen used ELISA for IFX quantification, and ELISA (“old”) and ECLIA (“new”) for ATI assessments.

*Results*.* IFX Assays*:* Specificity*: All assays were specific as they detected IFX, but not 5 mg/mL of adalimumab, certolizumab pegol, golimumab or siltuximab.* Selectivity*: TNFa (0.5–50 ng/mL) did not interfere with IFX detection; ATI titers ≥10 interfered with IFX assessment.* Accuracy*: Confirmed by 3 independent measurements of IFX (0.125–20 mg/mL) spiked sera from untreated IBD patients and with IFX measured in sera from IFX-treated IBD patients.* Precision*: IFX assays were precise, determined by inter-occasion reproducibility (2 wks between assays; Dynacare assessed the same day). The correlation of Janssen IFX results to IFX results from Sanquin, Dynacare, KU Leuven, and LabCorp were 0.936, 0.945, 0.968 and 0.972, respectively.

*ATI assays*:* Specificity*: Assays were specific in detecting anti-IFX Ab, and not affected by high titers of Ab against other monoclonal Ab drugs (ustekinumab and golimumab).* Selectivity*: Sanquin, LabCorp and new Janssen methods were drug tolerant (IFX > 10 ug/mL); ATI results from Dynacare and KU Leuven were affected by IFX concentrations at 2 *μ*g/mL or higher. Concentrations of free or bound TNFa (≤5 ng/mL) did not interfere with ATI detection; a supraphysiologic TNFa concentration (50 ng/mL) resulted in false positive results except Sanquin's.* Precision*: All assays were reproducible (2 wks between assays; Dynacare assessed samples same day).

*Conclusions*. Results from all labs were similar and significantly correlated to the results of Janssen assays. These results may aid in interpretation of data from commercial assays and assays used in IBD clinical studies of Remicade (IFX).

*Funding Agencies: *Janssen Research and Development, LLC

# Sub article: A302: Interventions for Treating Lymphocytic Colitis

**Authors:** N. Al Yatama, N. Chande, T. Bhanji, J. MacDonald

**Affiliations:** ^302.1^The University of Western OntarioLondonONCanada; ^302.2^Robarts Research InstituteUniversity of Western OntarioLondonONCanada

*Background*. Lymphocytic colitis is a subtype of microscopic colitis characterized by chronic, watery non-bloody diarrhea with normal endoscopic and radiologic findings. The etiology is unknown.

*Aims*. To evaluate the efficacy and safety of treatments for clinically active lymphocytic colitis. This is an update of a Cochrane review.

*Methods*. MEDLINE, PUBMED and EMBASE, Web of Science, Scopus and the Cochrane Library databases were searched from database inception to June 2015. Randomized controlled trials of medical interventions therapies for biopsy-proven, clinically active lymphocytic colitis were considered for inclusion. The relative risk and corresponding 95% confidence intervals for each dichotomous outcome and the mean difference and corresponding intervals for each continuous outcome were calculated. A random-effects model was used for the pooled analysis.

*Results*. Six RCTs were identified. Two trials (*N* = 56) compared budesonide 9 mg/day to placebo. At week 6 or 8, 88% (28/32) of patients in the budesonide group had a clinical response compared to 38% (9/24) of patients in the placebo group (RR 2.37, 95% CI 1.36–4.14; *p* = 0.002). In one study patients received beclometasone dipropionate 5 mg/day (*n* = 18), beclometasone dipropionate 10 mg/day (*n* = 13) or mesalazine 2.4 g/day (*n* = 5). No statistically significant difference in clinical response was observed between the 3 groups at week 8 (RR 0.97,; 95% CI 0.75–1.24; *p* = 0.8) and month 12 (RR 1.29; 95% CI 0.40–4.18; *p* = 0.67). One study compared oral mesalazine 800 mg tid (*n* = 20) to mesalazine 800 mg tid plus cholestyramine 4 g qd (*n* = 21). At month 6, 85% (17/20) of patients treated with mesalazine had clinical response compared to 86% (18/21) of those who received mesalazine plus cholestyramine (RR 0.99, 95% CI 0.77–1.28; *P* = 0.95). One study compared bismuth subsalicylate 2358 mg qd (*n* = 3) with placebo (*n* = 2). There was no statistically significant difference in clinical (RR 5.25, 95% CI 0.41–67.73; *p* = 0.2) or histological response (RR 1.33, 95% CI 0.27–6.61; *p* = 0.72) between groups. One trial compared probiotics (OptiBac®; *n* = 24) with placebo (*n* = 22) bid. All patients received loperamide (1 mg/day). Patients in the probiotics group were significantly less likely to experience decreased abdominal pain and frequency of defecation (*p* < 0.001).

*Conclusions*. Evidence indicates that budesonide may be effective for treating active lymphocytic colitis. Short-term therapy with beclometasone dipropionate may be effective, reported side effects include nausea, sleepiness and mood change. Weak evidence suggests that mesalazine with or without cholestyramine may be effective for treating lymphocytic colitis. No conclusions can be made regarding bismuth subsalicylate. Probiotics may attenuate lymphocytic colitis symptoms. More research is needed in this area.

*Funding Agencies: *None

# Sub article: A303: Inflammatory Bowel Disease and Pregnancy: Assessment of Patient Knowledge

**Authors:** M. Niazi, N. Deol, S. Fowler

**Affiliations:** ^303.1^Department of MedicineUniversity of SaskatchewanSaskatoonSKCanada; ^303.2^Div of GastroenterologyDept of MedicineUniversity of SaskatchewanSaskatoonSKCanada

*Background*. Inflammatory Bowel Disease (IBD) is a disease of the young, with a peak incidence between 18–35 years of age. Management of IBD during pregnancy is therefore common. Previous studies have demonstrated poor IBD-specific reproductive knowledge in women. Approximately 50% of female patients with IBD worry about infertility, despite studies showing that infertility rates are comparable to the general population when disease is well controlled. Patient concerns regarding IBD and pregnancy are evidenced by the observed increased rate of voluntary childlessness in IBD patients at 14–36% compared to 2.5–28% in the general population. Potential reasons for increased voluntary childlessness include concerns related to medication associated teratogenicity, the risk of passing on IBD to their children, and the impact of pregnancy on their disease. Previous studies have shown that a potentially modifiable factor associated with better knowledge and lower odds of childlessness among female IBD patients was discussion of family planning with a physician, specifically a gastroenterologist.

*Aims*. This study assesses IBD-specific reproductive knowledge and its impact on family planning among female patients in the Saskatoon Health Region.

*Methods*. A questionnaire was developed including questions from Crohn's and Colitis Pregnancy Knowledge Score (CCPKnow), a previously validated questionnaire, as well as questions pertaining to demographics, pregnancy and IBD history, and source of information on these topics. This was distributed to female IBD patients between the ages of 18–45 years seen in the Multidisciplinary IBD Clinic. Descriptive statistics were used to describe patient demographics and summarize results. Simple linear regression was used to assess correlations between source of knowledge and patient knowledge level.

*Results*. Thirty-four questionnaires were completed. Patients scored poorly on survey questions overall with 44% of patients answering <50% of questions correctly. Patient knowledge level was poorest (<25% correct) for questions pertaining to effect of IBD on fertility, inheritance of IBD, and safety of medications in pregnancy. However, >90% of patients were aware of the importance of having well controlled IBD to ensure good pregnancy outcomes.

*Conclusions*. Our study has identified 3 key areas of knowledge deficit including the effect of IBD on fertility, inheritance of IBD, and safety of IBD medications during pregnancy. This information will be used to develop educational materials and tools to address areas of knowledge deficiency. Dedicated resources within an IBD clinic are needed to improve patient knowledge in these areas.

*Funding Agencies: *None

# Sub article: A304: An Individualized and Multi-Faceted Transition Intervention Is Needed for Pediatric Patients with Inflammatory Bowel Disease

**Authors:** N. Klostermann, L. McAlpine, E. Wine, K. Goodman, K. Kroeker

**Affiliations:** ^304.1^Division of GastroenterologyUniversity of AlbertaEdmontonABCanada; ^304.2^Division of Pediatric GastroenterologyUniversity of AlbertaEdmontonABCanada; ^304.3^Division of Gastroenterology and School of Public HealthUniversity of AlbertaEdmontonABCanada

*Background*. A quarter of inflammatory bowel disease (IBD) patients are diagnosed in childhood and transition to adult care at 16–18 years old. Interventions that support young adults with chronic illness through the transition phase can improve the skills and knowledge required for a successful transition from pediatric care.

*Aims*. This needs assessment will inform the design of an intervention to improve the transition experience for IBD patients. The goals were to ascertain views of young adult IBD patients on transition, whether a transition intervention would be helpful, preferred content and format of a potential intervention and whether they have deficits in key skill and knowledge areas.

*Methods*. This mixed methods study utilized semi-structured qualitative interviews with 20 particpants, age 17–20 years, who transitioned during 2013–2015 from the Stollery Children's Hospital to the Zeidler Clinic in Edmonton, Alberta. Three validated assessments were used to evaluate any deficits in self-management/self-advocacy, medication adherence and knowledge of IBD. Interview responses were analyzed thematically. Non-parametric tests were used to compare scores on the assessments to published estimates.

*Results*. The idea of a transition intervention was well received by study participants. Discussion of what information an intervention should convey centered on medications, disease and what to expect during transition. The top three preferred ways to receive an intervention were one-on-one with a healthcare practitioner, handouts and websites. Themes generated are shown in Figure 35.

As shown in Table 51: participants scored above the published estimate for knowledge. Medication adherence and transition skill scores were comparable to published estimates; however, this means 1/3 of participants are poor adherers, and only 1 out of 20 had mastered 90% of transition skills.

*Conclusions*. Based on this assessment, we are designing an interactive website to address skill deficits and knowledge areas of interest. It will be individualized and multi-faceted, support the shift in responsibility and teach about transition.

*Funding Agencies: *None

# Sub article: A305: A Randomized Controlled Trial Using a Personalized “Alberta Anti-Inflammatory Diet” for Prevention of Relapse in Ulcerative Colitis

**Authors:** A. Keshteli, R. Valcheva, C. Nickurak, B. Halloran, S. van Zanten, K. Kroeker, R. Fedorak, K. Madsen, L. Dieleman

**Affiliations:** ^305.1^University of AlbertaEdmontonABCanada; ^305.2^University Of AlbertaEdmontonABCanada

*Background*. Epidemiological studies have suggested dietary intake to be related to higher incidence of ulcerative colitis (UC). However, there are sparse data on the role of specific dietary factors in triggering disease relapse or if dietary intake can be controlled to reduce disease relapse.

*Aims*. The aim of this randomized controlled clinical trial (RCT) study was to assess the effectiveness of a dietary intervention for maintenance of remission in UC patients.

*Methods*. In this 6-month RCT, adult UC patients in clinical remission (assessed by partial Mayo score) who had a clinical relapse during the previous 18-month were randomized to either an “Alberta-based Anti-inflammatory Diet” or to a diet based on Canada's Food Guide. Patients in the intervention group received an individual one hour dietary counselling as well as specific menu plans from a registered dietitian in 4 face-to-face interviews (at baseline, and 1, 3, and 6 months) and three telephone interviews (at month 2, 4 and 5). The anti-inflammatory diet was designed to increase patients' intakes of probiotics, prebiotics, soluble fibers, omega-3 PUFA and decrease red meat intake. The control group adhered to the Canada's Food Guide. The primary outcome was clinical flare and secondary outcomes were changes in serum C-reactive protein (CRP), quality of life (assessed by short inflammatory bowel disease questionnaire (SIBDQ)), and fecal calprotectin (FC). All were assessed at baseline and at month 6/or at time of flare. This study is registered on clinicaltrials.gov (NCT02093780).

*Results*. In these preliminary findings, 14 patients were randomized to each group. Mean age was 37.7 ± 15.0 years and 16 (57.1%) were females. Four patients (28.6%) in the control and 5 patients (35.7%) in the intervention group relapsed (*p* = 1.0). There was no statistically significant difference between the two diet groups in terms of changes in mean serum CRP and SIBDQ scores during the study. Interestingly, patients randomized to the control group had a 3-fold increase in mean FC levels from baseline to 6 months, while the patients on the anti-inflammatory diet had a 50% decrease in their FC levels (*p* = 0.1 obtained from repeated measures ANOVA, Figure 36 [Fig fig37] [Fig fig38] [Fig fig39]).

*Conclusions*. Dietary counselling and increased intake of anti-inflammatory type foods may be effective in reducing FC levels and colonic inflammation in UC patients. These encouraging preliminary findings will need to be confirmed in a larger sample size as well as investigations performed into the mechanisms underlying these results.

*Funding Agencies: *Alberta Innovates Biosolutions

# Sub article: A306: First Case Report of CML in CD Paitient Using Adalimumab: Risk of Malignancy with Biological Therapy and Challenges in Communicating Information to the Patient

**Authors:** A. Dhillon, A. Ilnyckyj, N. Narula

**Affiliations:** ^306.1^University of ManitobaWinnipegMBCanada; ^306.2^McMaster UniversityHamiltonONCanada

*Background*. A 14 year old was diagnosed with Crohn's disease. She was treated with prednisone, 5-ASA, and 6-MP. At age of 20 6-MP was started, initially 75 mg daily for 18 months and 100 mg daily for 4 months. At age 22, adalimumab was started, induction followed by 40 mg sc biweekly. Over several months therapy was escalated to 80 mg sc weekly and clinical remission was attained.

After 18 months of adalimumab, the family physician noted increased WBC 17.8 (baseline 11–14). Hematology advised a bone marrow and the diagnosis of CML was made. Imatinib was started with prompt normalization of the WBC.

At the time of diagnosis of CML, adalimumab was stopped for 6 months. The patient's CD recurred and adalimumab was restarted. There was no increase in her WBC with restarting adalimumab. Currently, both CD and CML are in remission/control with adalimumab and imatinib respectively for 4 months.

The patient's understanding of her CML remains that exposure to adalimumab caused the malignancy. This is based on her understanding of the discussion of cancer risk with her treating physician when she started adalimumab and of her reading of the product's monogram.

*Aims*. Review of the literature to determine the incidence of CML in the setting of biologic therapy and to highlight the need to explicitly discuss specific cancer risks when starting biological therapy

*Methods*. The literature was searched for the terms Crohn's disease, Chronic Myeloid Leukemia, adalimumab, imatinib and no other reports of CML while on adalimumab have been reported. The literature and product monogram documents an increased risk of NHL and non melanoma skin cancers.

*Results*. Our patient had several years of antimetabolite exposure, followed by relatively short exposure to adalimumab. Since CML is an acquired neoplasm, one can speculate that combined drug exposure, possibly the young age of drug exposure contributed to the development CML. Alternatively, the mechanism of her neoplasm may be independent of any of her Crohn's therapy. The neoplasm is rare and there are no reports of it in patients using biological therapy. Standard consenting to the product does not include risk of CML. There are some cancers that are well described to be associated with biological therapy.

*Conclusions*. This case illustrates the diagnosis of a rare malignancy in a young person with CD receiving biological therapy.

When patients receiving biological agents develop neoplasm, the biological agent's role is questioned. Clear information in the consenting process may assist the patient in adapting to this unfortunate and challenging circumstance. Clinicians should have a good working knowledge of the types of described cancer complications with biological therapy when consenting patients.

*Funding Agencies: *None

# Sub article: A307: Cytomegalovirus (CMV) Colitis Trigerring Inflammatory Bowel Disease (IBD) in an Immunocompetent Adult: A Case Report and Review of the Literature

**Authors:** A. Bitton, M. Shehab

**Affiliations:** ^307.1^McGill UniversityMontrealQCCanada; ^307.2^McgillMontrealQCCanada

*Background*. Cytomegalovirus (CMV) colitis is a rare condition in immunocompetent patients. When severe, CMV colitis can lead to significant morbidity and mortality. We describe CMV colitis developing in an immunocompetent adult and leading to IBD. We also provide a brief review of the literature related to this condition.

*Aims*. N/A.

*Methods*. N/A.

*Results*. A 36 year-old male, with no known past medical conditions but positive family history of ulcerative colitis, presented with a 5-day history of bloody diarrhea and mucus in the stool, associated with fever. On physical examination, he was noted to have swinging pyrexia, ranging between 37 and 40°C. No other signs were observed during the examination. His initial blood investigations and stool cultures did not reveal any abnormalities. The patient was admitted for further investigations. Computed tomography (CT) scan of the abdomen showed diffuse circumferential wall thickening involving the descending and sigmoid colon, consistent with colitis. Patchy non-specific colitis was observed during colonoscopy. Biopsies revealed non-specific inflammatory process. One week following his admission, pancytopenia and splenomegaly developed. A blood film was then ordered, which revealed non-specific findings. He was started on piperacillin/tazobactam and vancomycin for his febrile neutropenia. A complete viral serology workup showed positive CMV IgG and IgM, as well as a high CMV polymerase chain reaction (PCR) titer. A repeat colonoscopy with biopsies was positive for CMV. Immunodeficiency was then ruled out with the appropriate investigations. A 3-week course of intravenous ganciclovir therapy was completed. The patient then reported complete resolution of his symptoms. After four weeks he remained with diarrhea. A colonoscopy revealed left sided active colitis. Biopsies were consistent with ulcerative colitis. Patient was started on oral 5-ASA. On subsequent follow-up visit his symptoms had almost completely resolved.

*Conclusions*. The diagnosis of CMV colitis should be considered in immunocompetent adults presenting with a clinical picture of acute infectious diarrhea. In severe cases, early diagnosis and treatment with the appropriate antiviral therapy is essential in order to avoid serious complications. CMV colitis may trigger the onset of Ulcerative colitis.

*Funding Agencies: *None

# Sub article: A308: Using Infliximab Trough Levels and Fecal Calprotectin Levels Together to Guide Clinical Decisions Has the Potential to Improve Outcomes in Inflammatory Bowel Disease Patients on Maintenance Infliximab Therapy

**Authors:** A. Amin, V. Huang

**Affiliations:** ^308.1^University of AlbertaEdmontonABCanada

*Background*. Infliximab (IFX) induces and maintains clinical remission in Crohn's Disease (CD) and Ulcerative Colitis (UC). Up to 45% of patients with CD and 60% of patients with UC on maintenance IFX therapy develop secondary loss of response (LOR). Studies recommend IFX trough levels (ITLs) be maintained from “detectable" up to 10 *μ*g/mL to prevent LOR. However, patients continue to have LOR despite adequate ITLs indicating they continue to have inflammation. Fecal Calprotectin (FCP) is a marker of neutrophilic infiltration into the GI tract. When elevated, FCP predicts LOR to maintenance IFX therapy (sensitivity 0.80, specificity 0.82). We previously showed clinicians would change clinical decisions if they had knowledge of ITLs and FCP levels (FCPLs).

*Aims*. To determine if 6 month clinical outcomes in IBD outpatients on IFX maintenance therapy could be improved if clinicians had knowledge of both ITLs and FCPLs (compared to not having level knowledge).

*Methods*. Adult IBD outpatients on IFX maintenance therapy had disease activity scores, ITLs and FCPLs measured. At the time, clinical decisions were made based on clinical presentation. Subsequent 6 month outcomes (e.g., investigation, steroids, etc.) were recorded. An expert clinician panel was then presented with the clinical data, ITLs and FCPLs and hypothetical decisions were made. Hypothetical 6 month outcomes based on those hypothetical decisions will be proposed based on the literature. Differences between actual and hypothetical outcomes will also be analyzed. Statistical analyses included: medians with interquartile ranges (IQR); proportions; percentages; and contingency tables. Receiver operating curves and area under the curve analyses will also be performed.

*Results*. 31 IBD outpatients on IFX maintenance therapy (between 2013 and 2014) who had ITLs and FCPLs drawn were included. Demographics: median age 40.0 (IQR 29.0–59.0) years; 21 (67.7%) females; 23 (74.2%) had CD; 14 (45.2%) were in clinical remission; and 23 (74.2%) were on concomitant immunosuppresion. Median ITL was 9.4 (IQR 5.4–16.1) and median FCPL was 97.0 (IQR 14.0–362.0).  Table 52 shows: (1) that the initial decision differed from the hypothetical decision in 14/31 (45.2%) of cases (bold); and (2) outcomes that could be related to initial decisions that were discrepant from the hypothetical decisions in 25/31 (80.6%) of cases (italic).

*Conclusions*. While previous studies have explored the use of ITLs and FCPLs in isolation, the preliminary results of this study demonstrate that knowledge of ITLs and FCPLs can aid clinicians to optimize the management of IBD outpatients on IFX maintenance therapy in order to improve patient outcomes.

*Funding Agencies: *None

# Sub article: A309: An Online Educational Portal Is Effective in Improving Knowledge Regarding Reproduction and IBD

**Authors:** K. Wierstra, K. Smith, L. Ambrosio, L. Dieleman, B. Halloran, K. Kroeker, R. Fedorak, K. Berga, V. Huang

**Affiliations:** ^309.1^University of AlbertaStony PlainABCanada; ^309.2^University of AlbertaEdmontonABCanada; ^309.3^University Of AlbertaEdmontonABCanada; ^309.4^Los Alamos National LaboratoryEdmontonABCanada; ^309.5^MacEwan UniversityEdmontonABCanada

*Background*. Inflammatory Bowel Disease (IBD) is a chronic disease involving inflammation of the gastrointestinal tract. IBD affects patients through their adolescent and young adult years. Many IBD patients have a lack of knowledge regarding their disease and reproduction.

*Aims*. The aim was to evaluate an online educational portal in improving knowledge regarding reproduction and IBD among IBD patients.

*Methods*. An online educational portal covering the topics of IBD and fertility, pregnancy, delivery, and breast feeding was developed by the University of Alberta IBD group. IBD patients (age 18 to 45 yrs) were invited to participate in this study through clinic and advertisements (paper and social media). Participants were randomized into two groups: (1) text-only webpages and (2) interactive modules (short videos, slide sets, self-quizzes, FAQ), and then given two months to access the online educational portal. Participants completed pre- and post-study Crohn's and Colitis Pregnancy Knowledge (CCPKnow) questionnaires to assess their pregnancy-related IBD knowledge. Responses were grouped as: poor (0 to 7), adequate (8 to 10), good (11 to 13) or very good (14 to 17). Pre and post CCPKnow scores were compared using non-parametric tests.

*Results*. As of October 1, 2015, 36 of the registered participants (5 males and 31 females) have completed the study Knowledge scores improved from pre-study CCPKnow 7.7 (IQR: 4.0–12.0) points to post-study 16.0 (IQR: 14.3–16.0) points (*p* value < 0.01). Participants in the interactive group improved their CCPKnow scores by 2.0 more points than the text-only group, 8.0 (IQR: 3.5–12.0) points versus 6.0 (IQR: 4.0–9.0) points, respectively.

*Conclusions*. Access to an online educational portal improves knowledge regarding reproduction and IBD. Interactive modules improve knowledge more than text-only webpages.

*Funding Agencies: *AHIS

# Sub article: A310: SAPHO Syndrome 10 Months after Initiation of Remicade for Crohn's Disease: Case Report

**Authors:** N. Clermont Dejean, S. Plamondon

**Affiliations:** ^310.1^Université de SherbrookeSherbrookeQCCanada

*Background*. SAPHO (Synovitis Acnea Pustulosis Hyperosteosis and Osteitis) syndrome has an estimated incidence of 1/10 000 person-year. The main clinical features are recurrent aseptic axial osteomyelitis associated with specific dermatologic conditions, most commonly palmoplantar pustulosis (PPP). An association between SAPHO syndrome and Crohn's disease (CD) has been described in literature. This particular variant of SAPHO syndrome is considered a rare extraintestinal manifestation (EIM) of CD.

*Aims*. We report a case of SAPHO syndrome after treatment of CD with infliximab and review the associations made between SAPHO syndrome and CD.

*Methods*. The case notes were reviewed after informed consent from the patient and his parents. A review of the literature was performed using Medline Ovid with the keywords: SAPHO syndrome, sterile osteomyelitis, psoriasis, infliximab, anti-TNF.

*Results*. A 15-year-old male with ileal CD presented in 2011 with a two-week history of arthralgia in the right wrist and sterno-clavicular area as well as a rash. His CD had been treated with infliximab for the past ten months with good clinical and radiological response. On presentation, he was afebrile, had pain on palpation of the clavicles and right wrist without overt arthritis, and multiple squamo-erythematous plaques on his scalp, face, right arm, torso and armpits as well as micro-papules on both palms. Serum inflammatory markers were markedly elevated. A bone gallium scintigraphy demonstrated osteomyelitis of the sterno-clavicular regions, distal right radius and trochanter. Blood and skin cultures were negative. The rash was diagnosed as pustular psoriasis and in view of the multiple sterile osteomyelitic lesions the final diagnosis of SAPHO was made. Since the complication occurred while on anti-TNFs in a patient who had no previous EIMs, the medication was replaced with oral methotrexate. Osteomyelitic lesions rapidly improved, but an MR-enterography 4 months later confirmed the recurrence of ileitis with a 10-cm terminal ileal stenosis for which the patient underwent an ileocecal resection. He continued methotrexate and as of September 2015 has had no recurrence of Crohn's disease or SAPHO.

To our knowledge, this is the second case of SAPHO syndrome diagnosed following therapy with infliximab for CD. The first case, reported by Van Den Eynde et al. in 2007, describes a patient who developed migratory hip, thigh and back pain with a papulopustular rash diagnosed as PPP after 2 doses of infliximab. This patient was treated with pamidronate, clarithromicine, sulfasalazine, methyl-prednisone and methotrexate with a good response and no further recurrence.

*Conclusions*. SAPHO syndrome has been regarded as a rare extraintestinal manifestation of CD. Our case suggests it may also occur as a complication of anti-TNF therapy.

*Funding Agencies: *None

# Sub article: A311: Small-Fiber Neuropathy in a Pediatric Patient with Ulcerative Colitis on Tumor Necrosis Factor Alpha-Inhibitor Treatment

**Authors:** J. Breton, C. Deslandres, E. Haddad, G. D'Anjou

**Affiliations:** ^311.1^Université de MontréalMontréalQCCanada; ^311.2^Hôpital Sainte*-*JustineMontréalQCCanada

*Background*. Neurological complications associated with inflammatory bowel disease (IBD) although uncommon have been associated with significant morbidity and may represent relevant diagnostic issue. Furthermore, increasing use of biological therapies for IBD, which has been associated with different neurological adverse effects, has likely influenced the incidence and clinical presentation of this complication. Peripheral neuropathies are one of the most frequent complications and diverse phenotypes have been described.

*Aims*. To describe a pediatric patient with ulcerative colitis and autoimmune hepatitis who developed small-fiber neuropathy while being treated on tumor necrosis factor (TNF) alpha inhibitor with successful response to intravenous immunoglobulin.

*Methods*. We retrospectively reviewed the medical chart of our patient. We performed a review of the literature using the PUBMED database. The following search terms were used: “neuropathy”, “small-fiber neuropathy” and/or “neurological disease” in combination with “inflammatory bowel disease”, “anti-TNF”, “anti-ganglioside”.

*Results*. We described a 17 years old girl with autoimmune hepatitis and ulcerative colitis who developed severe burning neuropathic pain affecting the proximal lower extremities while being treated on TNF alpha-inhibitor. Skin biopsy confirmed a non-length-dependent small fiber neuropathy. Investigations for potential causes revealed abnormal anti-GM2 titer. Immune-mediated pathogenesis was suggested by rapid response to intravenous immunoglobulin. Whether this neurological complication was related to TNF alpha-inhibitor therapy or to our patient's underlying immune dysregulation or even to the presence of unrelated anti-ganglioside antibodies remains to be elucidated.

*Conclusions*. Non-length-dependent small fiber neuropathy is not as well characterized as length-dependent small-fiber neuropathy in the IBD population. Our case report is unique as it describes a distinct clinico-pathological pattern of small-fiber neuropathy associated with IBD and TNF alpha inhibitor therapy with findings suggestive of predominant dorsal root ganglia degeneration on skin biopsy. To our knowledge, this is the youngest patient developing small-fiber neuropathy during the course of an inflammatory bowel disease. Peripheral neuropathies associated with IBD in the pediatric population have rarely been described which emphasizes the need for future pediatric studies on this complication.

*Funding Agencies: *None

# Sub article: A312: Pateint Experience Living with Inflammatory Bowel Disease

**Authors:** U. Chauhan, J. Popov, Y. Farbod, D. Armstrong, S. Halder, S. Kaasalainen, J. Marshall, F. Tse, P. Moayyedi

**Affiliations:** ^312.1^McMaster University Medical CentreHamiltonONCanada; ^312.2^McMaster UniversityHamiltonONCanada

*Background*. Inflammatory Bowel Disease (IBD) is a lifelong chronic inflammation presents early in life; the two main phenotypes are Crohn's disease (CD) and Ulcerative Colitis (UC). The chronic relapsing nature of the disease results in lasting physical, psychological and social stress.

*Aims*. The primary objective of this study was to examine patients' experiences and challenges associated with living with IBD.

*Methods*. Using a qualitative descriptive approach, data was collected using five focus group sessions with a total of 17 individuals with diagnosis of IBD. The focus group sessions were audiotaped, transcribed, coded and analyzed using thematic content analysis.

*Results*. Seventeen IBD patients with average age of 44 (25–77 years); 15 CD, 2 UC; 8 F, 9 M; average disease duration of 19 years were included. The main theme across the 5 focus groups was: “IBD controls your life”. This was further divided into 4 sub-themes: (1) long duration from onset of symptoms to diagnosis; (2) poor experience with initial treatment; (3) lack of public knowledge of IBD requiring self-advocacy; (4) managing the psychosocial, emotional and financial impact of disease. There were similar experiences and challenges, including lack of understanding from school and work force were reported. Initial symptoms were dismissed as attention seeking in those diagnosed during teen years. Following diagnosis, patients' main concern was choosing the right career path while effectively managing their IBD. However, advanced prearrangement of special accommodation for schoolwork, and time off from school and work for follow-up appointments continued to be a source of difficulty. Inability to hold a fulltime employment, early retirement and out of pocket expenses not covered by various funding agencies lead to financial struggles. Psychosocial impacts were often associated with the inability to travel and attend social events due to IBD-induced constraints. This led to feelings of isolation, exclusion, and deep depression. An additional consensus was that current biological treatments are more effective and tolerable than the use of corticosteroids and surgical intervention, which may have caused additional health problems.

*Conclusions*. Despite the demographic differences, there were many common themes among the 5 focus groups. The general consensus was the greatest difficulties associated with living with IBD was garnering understanding from others and learning to live their lives around the disease. The main, unifying theme that emerged was that IBD plays a significant role in many different facets of the lives of the patients, and learning to manage and cope with the burden of symptoms and its effects on daily life becomes a lifelong process.

*Funding Agencies: *None

# Sub article: A313: A Case of Hepatosplenic Gamma-Delta T Cell Lymphoma with Intravascular Lymphoma Like Features in a Young Male with Autoimmune Hepatitis

**Authors:** M. Miles, S. Shivakumar

**Affiliations:** ^313.1^Dalhousie UniversityHalifaxNSCanada

*Background*. Hepatosplenic gamma delta T-cell lymphoma (HSGDTL) is a rare and aggressive hematologic malignancy. There are no definitive risk factors for HSGDTL but an association with immunosuppressive agents has been well documented in renal transplant recipients and patients with inflammatory bowel disease (IBD). Specifically, the use of thiopurines for at least two years in young males with IBD has been linked to an increased risk of developing HSGDTL. To our knowledge, however, previous cases of HSGDTL in autoimmune hepatitis patients have been minimally reported in the literature.

*Aims*. We hope our case report will contribute to better understanding the association between T-cell lymphoma and long term use of immunosuppressive agents as well as identify new populations, specifically autoimmune hepatitis patients, who may be at risk of developing this deadly disease.

*Methods*. We obtained consent to report a case of a 22-year-old male on azathioprine and prednisone for autoimmune hepatitis who developed hepatosplenic gamma-delta T cell lymphoma.

*Results*. Our patient presented to hospital with abdominal pain, night sweats and 20 lbs of weight loss. On exam he had hepatomegaly and splenomegaly but no lymphadenopathy. His initial lab tests were WBC 5.39 × 10^9^/L, Hb 70 g/L, platelets 25 × 10^9^/L, total bilirubin 74 *μ*mol/L, AST 151 U/L, ALT 55 U/L and LDH 5932 U/L. He had marked hepatosplenomegaly on abdominal CT scan. Bone marrow and liver biopsies confirmed the presence of HSGDTL. Unfortunately he did not respond to chemotherapy and passed away. On autopsy he had multiorgan involvement of an intravascular lymphoma like (IVLL) T-cell population.

*Conclusions*. As far as we know, a widespread multi-organ intravascular lymphoma in a HSGDTL patient is exceedingly rare. In fact we found only one other case in the literature. Therefore, it is difficult to assess the impact of this finding. It is possible the presence of intravascular lymphoma represents a subtype of HSGDTL and may provide insight into the aggressive nature of this disease. We must also consider an association between autoimmune hepatitis and HSGDTL with IVLL component independent of immunosuppressive therapy. However, future research into this little known and seldom reported malignancy will be required to fully appreciate these findings.

*Funding Agencies: *None

# Sub article: A314: Spindle Cell Squamous Cell Carcinoma in a Patient with Crohn's Disease on Long-Term Immunosuppression: A Case Report and Literature Review

**Authors:** M. Cino

**Affiliations:** ^314.1^University of TorontoTorontoONCanada

*Background*. Treatment with thiopurines increases the risk of non-melanoma skin cancers (NMSC) in patients with inflammatory bowel disease (IBD). This risk is higher in patients with Crohn's disease as compared to ulcerative colitis. Spindle cell squamous cell carcinoma (SpSCC) is a rare NMSC of the head and neck. It is most often found in the larynx or oral cavity and is rarely confined to the skin. SpSCC most commonly presents as a polypoid lesion with or without ulceration though appearance varies. Histological examination shows elongated, spindle-shaped cells in a pinwheel formation staining positive for keratin proteins. Treatment is surgical excision. Unfortunately, there is a high rate of local recurrence and metastatic potential.

*Aims*. N/A.

*Methods*. A 67 year old non-smoking, Caucasian male with Crohn's disease on Azathioprine (Aza) for twelve years presented with a raised lesion on the right cheek. His dose of Aza ranged from 50 to 100 mg daily. Pathology from the excised lesion identified a poorly differentiated SpSCC. Immunohistochemistry stained positive for keratin proteins. Three months after the lesion was excised, a 1 by 2 cm raised, ulcerated lesion appeared on the forehead concerning for recurrent SpSCC. Aza was immediately discontinued.

*Results*. Aza causes photosensitivity. Skin damage occurs at low doses of sunlight exposure. Use of Aza results in incorporation of 6-thioguanine (6-TG) into skin cell DNA. 6-TG absorbs UVA light and creates reactive oxygen species resulting in DNA mutation and increased risk of malignancy. Long et al. showed that recent (<90 days) use of Aza increased the risk of developing NMSC with an odds ratio of 3.56 (95% CI, 2.81–4.50). Persistent (>365 days) use of Aza further increased the risk of developing NMSC with an odds ratio of 4.27 (95% CI, 3.08–5.92). Abaas et al. found a 2 fold increase in the risk NMSC after 2 years of exposure, climbing to a 3.6 fold increase observed by 5 years. The risk of NMSC fell back to baseline after discontinuation of the medication irrespective of the previous cumulative dose.

*Conclusions*. Our case highlights the important and significant risk of NMSC in patients with inflammatory bowel disease (IBD) on Aza. Patients with IBD should be counselled about the increased risk of NMSC before starting a thiopurine. In addition, patients taking thiopurines should be advised to minimize other risk factors for NMSC, such as sun exposure and cigarette smoking. Current guidelines suggest regular screening dermatological examinations for all patients taking thiopurines.

*Funding Agencies: *None

# Sub article: A315: FODMAPS Alter the Metabolome and Symptoms in Irritable Bowel Syndrome Patients

**Authors:** K. McIntosh, D. Reed, T. Schneider, F. Dang, A. Keshteli, G. De Palma, K. Madsen, P. Bercik, S. Vanner

**Affiliations:** ^315.1^Western UniversityLondonONCanada; ^315.2^GIDRUQueen's UniversityKingstonONCanada; ^315.3^University of AlbertaEdmontonABCanada; ^315.4^Farmcombe InstituteMcMaster UniversityHamiltonONCanada

*Aims*. FODMAP (fermentable oligo-, di-, and mono-saccharides and polyols) diets can induce irritable bowel syndrome (IBS) symptoms but the mechanism(s) are poorly understood. We used low and high FODMAP diets to examine how metabolomic changes might underlie IBS symptomatology.

*Methods*. We performed a randomized controlled, blinded study of ROME III IBS patients. Patients were randomized to a low (*n* = 20) or high (*n* = 20) FODMAP diet for 3 weeks. Symptoms were assessed using the IBS severity scoring system (IBS-SSS). The metabolome was evaluated by comparing baseline and post diet measurements using the lactulose breath test (LBT), metabolic profiling in urine using direct infusion (DI-MS) and gas chromatography mass spectrometry (GC-MS), and partial 16S rRNA gene profiling (Illumina) to analyze stool microbiota composition.

*Results*. Thirty-seven patients (19 low FODMAP; 18 high FODMAP) completed the 3 week diet. The IBS-SSS was reduced in the low FODMAP (*p* < 0.001) but not the high FOMAP group. Metabolic profiling of urine showed both patient groups were similar at baseline but differed significantly after the 3 week diet (*p* < 0.01). Four metabolites (histamine, p-hydroxybenzoic acid, a phosphatidylcholine, and putrescine) were primarily responsible for discrimination between the two groups. Histamine was reduced 8 fold in the low FODMAP group (*p* < 0.001) but not the high FODMAP group. LBTs showed a minor reduction in H_2_ gas production with the low FODMAP diet that was not significant unless corrected for baseline differences (*p* < 0.05). Partial 16s rRNA gene profiling did not reveal differences in *α* and *β* diversity of the fecal microbiota before and after the diets but when comparing diets after 3 weeks we observed increased bacterial richness (*p* = 0.047) in the low FODMAP group (IBS-C excluded). The low FODMAP diet decreased the relative abundance of Propionibacteriaceae (Actinobacteria) and several butyrate producing bacteria.

*Conclusions*. A low FODMAP diet reduced IBS symptoms and was associated with significant changes in the metabolome and microbiota, but the study was not powered to correlate these. Changes in histamine levels suggest FODMAPS are linked to immune activation and could worsen symptoms, given that histamine sensitizes nerves that mediate pain. Microbiota changes resulting from the low FODMAP diet could improve symptoms by decreasing FODMAP fermentation products (e.g., gas and SCFA).

*Funding Agencies: *CIHR

# Sub article: A316: Protease-Mediated Effects of Commensal Bacteria on Nociceptive Dorsal Root Ganglia Neurons

**Authors:** J. Sessenwein, S. Vanner, A. Lomax, E. Petrof

**Affiliations:** ^316.1^Queens UniversityKingstonONCanada; ^316.2^queen'sKignstonONCanada; ^316.3^Queen's UniversityKingstonONCanada

*Background*. The mucosal barrier that separates the microbiota from the nervous system is disrupted during gastrointestinal (GI) inflammation. It is presently unknown whether translocation of bacteria during GI inflammation modulates visceral sensation and thereby contributes to pain. The primary aim of this study was to determine if a defined community of commensal GI microbes (MET-1) alters pain signaling.

*Aims*. Accordingly we examined whether supernatant containing the secretory products of MET-1 altered the excitability of dorsal root ganglion (DRG) neurons, and which downstream signaling pathways may be involved. In addition, we sought to identify candidate bacterial mediators of this effect.

*Methods*. DRG neurons were dissociated from mice and incubated overnight in sterile MET-1 supernatant (1 : 10–1 : 1000 dilution in sterile media) or sterile control media, in the presence or absence of various inhibitors. Electrophysiological experiments were performed after 24 hours.

*Results*. MET-1 decreased the excitability of DRG neurons by significantly increasing rheobase, 92.8 pA (*n* = 7; 1 : 100 MET-1), versus 50.0 pA (*n* = 30; controls), *p* < 0.001; Mann-Whitney test. This was accompanied by a hyperpolarization of the DRG resting membrane potential, −68.4 mV (*n* = 7; 1 : 100 MET-1), versus −58.3 mV (*n* = 30; controls), *p* < 0.05; Mann-Whitney test. In addition, MET-1 increased voltage-gated K^+^ current (*p* < 0.001; 2 way ANOVA). Addition of a nuclear factor kappa B (NF*κ*B) inhibitor (SC-514, 20 *μ*M) or an ERK1/2 inhibitor (PD 98059, 30 *μ*M) blocked the effects of MET-1. A bacterial protease inhibitor cocktail (1 : 10,000) abrogated MET-1 effects on DRG neurons. The serine protease inhibitor (FUT-175, 50 *μ*M) abolished the effects of MET-1 on the excitability of DRG neurons but not inhibitors of cysteine, acid proteases, metalloproteases, or aminopeptidases.

*Conclusions*. Serine proteases secreted by MET-1 can directly impact the function of DRG neurons, through NF*κ*B and ERK1/2-dependent pathways. On the basis of these observations, pain signaling from the gut to the brain may be modulated by microbiota-neuronal interactions.

*Funding Agencies: *Crohn's and Colitis Canada

# Sub article: A317: Anti-Gliadin Antibodies Levels and HLA-DQ8 Celiac Susceptibility Gene Are Important in the Development of Behavioural and Motility Changes Associated with Gluten Sensitivity

**Authors:** M. Pigrau, G. De Palma, S. Sidani, P. Miranda, J. McCarville, S. Cocciolillo, J. Lu, E. Verdu, S. Collins, P. Bercik

**Affiliations:** ^317.1^McMaster UniversityHamiltonONCanada

*Background*. Non-celiac gluten sensitivity (NCGS) is a frequent and poorly understood disorder, which shares many clinical features with irritable bowel syndrome and celiac disease, including gastrointestinal symptoms and frequent psychiatric comorbidity. The prevalence of anti-gliadin (AGA) and HLA-DQ2/8 genes is higher than in general population and seem to predict symptomatic response to gluten-free diet. It has been shown that gluten sensitization and challenge in genetically predisposed mice leads to immune activation and gut cholinergic nerve dysfunction, however the in vivo correlates of these findings are unknown.

*Aims*. To investigate the effect of gliadin challenge on behaviour and motility in gliadin-sensitized wild type and NOD-DQ8 mice.

*Methods*. C57Bl/6 and NOD-DQ8 mice maintained on a gluten-free diet were orally sensitized with cholera toxin with or without gliadin weekly for 3 weeks. The mice were then challenged with gliadin or vehicle 3 times per week for 2 weeks. Gastrointestinal motility (bead videofluoroscopy) and behavioural profiles (step-down, light preference and tail suspension tests) were assessed before and after gliadin challenge. Mice were sacrificed thereafter and tissue samples were collected. Statistical analysis was performed using Mann-Whitney or *t*-tests, as appropriate.

*Results*. Gliadin sensitization alone did not affect motility or behaviour in C57Bl/6 or in NOD-DQ8 mice. After gliadin challenge, only gliadin-challenged NOD-DQ8 mice with high serum AGA levels displayed anxiety-like behaviour compared to controls, and tended to have delayed gastrointestinal transit. Although a mild reduction in the villi/crypt ratio was observed in sensitized and challenged NOD-DQ8 compared to controls, no overt inflammation, atrophic lesions or tissue transglutaminase 2 antibodies were observed in either group.

*Conclusions*. Our data suggest that the presence of the AGA and HLA-DQ8 celiac susceptibility gene is important in the development of behavioural and motility changes associated with gluten sensitivity. Further characterization of AGA will be important to develop a needed serological biomarker to identify populations of NCGS patients that can benefit from gluten restriction.

*Funding Agencies: *CIHR

# Sub article: A318: High Resolution Is Superior to Conventional Esophageal Manometry in Assessing Patients with Post-Fundoplication Esophageal Symptoms

**Authors:** Y. Tse, N. Diamant, A. Correia, L. Liu

**Affiliations:** ^318.1^University Health NetworkTorontoONCanada; ^318.2^University of TorontoTorontoONCanada

*Background*. Post-fundoplication dysphagia is common and affects 5–10% of patients. However, studies in the past failed to demonstrate pre-op esophageal manometry (EM) findings predict post-op dysphagia or difference in post-op EM findings between patients with or without dysphagia. This could be secondary to the diagnostic accuracy of conventional EM and definitions of abnormality used in different studies. High-resolution manometry (HRM) now becomes the gold standard modality; however, no study has been done to assess the diagnostic yield of HRM in comparison with conventional manometry (CM) to help guide treatment in patients suffering from post-fundoplication dysphagia.

*Aims*. To compare diagnostic yield of HRM and CM in patients with post-fundoplication dysphagia.

*Methods*. We reviewed 36 consecutive post-fundoplication patients who presented for HRM between Feb 2008 and Oct 2011. HRM findings were reported by an expert. Line tracing recordings from 5 sensors, mimic CM, of each HRM study were extracted and reviewed by another blinded expert experienced in interpretation of CM. Comparison of the findings (focusing on esophageal body and lower esophageal sphincter (LES) motor profile) between HRM and CM was performed by an independent assessor.

*Results*. Of the 36 patients, 11 (31%) were male (age 53.3, range 21–81 years). Majority (72%) of patients reported dysphagia as one of their symptoms. The sensitivity and specificity of identifying the presence of bolus pressure using CM were low (36.8% and 11.8% resp.). CM failed to identify the presence of hiatal hernia in 62.5% of cases or misinterpret the hiatal hernia as bolus pressure in 37.5%. For the LES profile, differences were found in 61% of reports regarding the basal and/or residual pressure. This led to a difference in overall impression of the manometry findings in 44.4% of reports.

*Conclusions*. Our study shows that, comparing with CM, HRM provides more accurate information, especially for bolus pressure, hiatal hernia, and LES motor profile, which could alter management of patients' esophageal symptoms after fundoplication.

*Funding Agencies: *None

# Sub article: A319: Hirschsprung Disease as a Challenging Disease: Data from a Pediatric Hirschsprung Cohort in Quebec, Canada

**Authors:** F. Righini-Grunder, N. Mamoun, A. Le-Nguyen, N. Pilon, R. Soret, A. Aspirot, C. Faure

**Affiliations:** ^319.1^Ste Justine hospitalMontrealQCCanada; ^319.2^UQAMMontrealQCCanada; ^319.3^CHU Ste JustineMontrealQCCanada

*Background*. Hirschsprung disease (HSCR) is a congenital disorder of the enteric nervous system, incidence 1 : 5000 of live births and male to female ratio of 4 : 1. Treatment is surgical resection of the aganglionic segment and anal pull-through surgery. Bowel dysfunctions such as fecal incontinence or constipation are known complications and can impair quality of life.

*Aims*. Determination of the phenotype and long-term outcome of a HSCR population.

*Methods*. Retrospective study of patients with HSCR diagnosed between 1994 and 2014 at Sainte-Justine Hospital.

*Results*. 101 patients were identified (22 F). 68 patients had short form (rectosigmoid), 16 long form (descending ± transverse ± ascending colon), 5 total colonic and 4 extended aganglionosis; data not available in 8. 37 patients had other malformations (cardiac malformations, *n* = 27; intestinal atresia, *n* = 3; urinary tract malformation, *n* = 6; skeletal malformation, *n* = 9; sensory-neuronal anomalies, *n* = 9; endocrinopathies, *n* = 9). 14 patients were diagnosed with Trisomy 21, 2 with Smith Lemli Opitz syndrome and 2 with Ondine syndrome. Five patients died after birth (4 with Trisomy 21 and one with Ondine syndrome). Patients underwent modified Swenson or modified Soave procedure. Median age at first surgery (one-step repair, *n* = 68; two-step repair with colostomy or ileostomy, *n* = 26; n.a., *n* = 7) was 5.5 weeks (range 1–412 weeks), median weight at first surgery was 3.55 kg (range 2.45–18.9 kg). Necrotizing enterocolitis and/or bowel perforation occured in 23 patients pre-surgery (short form, *n* = 12; long form, *n* = 5; total colonic, *n* = 3; extended form, *n* = 2, n.a., *n* = 1) and in 15 after surgery (short form, *n* = 10; long form, *n* = 4; extended form, *n* = 1). Post-surgery follow-up was available in 87 patients (median duration 61 months, range 3–223 months). Anal dilatations were performed in 71 children (40 with anastomotic anal stenosis) from 3 to 189 weeks of age, maximal daily/minimal monthly. Constipation and fecal incontinence were reported in 36 and 51 patients respectively (23 suffered from both). Median age at date of diagnosis of constipation and fecal incontinence was 41 and 51 months respectively.

*Conclusions*. Distribution of type and age was comparable with the literature. Complications prior to surgery were more frequent in the longer form than in the short form (40% versus 18%). During follow-up fecal incontinence was more present than constipation.

This study demonstrates the potentially complicated and complex course of HSCR. If phenotype, surgery, complications and genotype influence the long-term outcome has to be confirmed. A prospective study in collaboration with the university of Québec in Montreal is ongoing.

*Funding Agencies: *None

# Sub article: A320: Medication Use Is Associated with Esophageal Manometric Abnormalities

**Authors:** D. Jacob, S. Pradhan, L. Wilsack, M. Buresi, M. Curley, M. Gupta, A. Shaheen, C. Andrews

**Affiliations:** ^320.1^Department of Gastroenterology and HepatologyUniversity of CalgaryCalgaryABCanada; ^320.2^University of CalgaryCalgaryABCanada

*Background*. Surprisingly little is known about the effects of medication on esophageal motor physiology. Many manometries show nonspecific abnormalities, and it is difficult to know if the abnormalities represent a primary dysmotility versus medication side effects.

*Aims*. We hypothesized that medications known to affect intestinal or colonic motility could also have measurable effects on esophageal pressure and/or function.

*Methods*. All patients with dysphagia or chest pain who underwent high-resolution esophageal manometry (HRM) with impedance, over a 22-month period were analyzed. Any patients with achalasia, connective tissue disorder, eosinophilic esophagitis or structural lesions on endoscopy were excluded. Detailed medication history on the day of the HRM was taken. Medication types were grouped into classes and tested along with age, gender, and height in multiple linear regression analyses to assess for association with HRM endpoints.

*Results*. Of a total 204 patients that were included in this analysis, 63.2% were females and 36.8% were males. 70.6% reported dysphagia, while 29.4% reported chest pain as the primary complaint. 67.2% of these patients were assessed as having ineffective esophageal motility using HRM. Regular narcotic use and female gender were found to be significant predictors of higher LES mean basal pressure, whereas PPI use was associated with lower LES mean basal pressure (Table 53). Anticholinergic use was associated with more failed swallows (assessed by Chicago Classification). No associations were seen between medication classes and LES residual pressure, distal contractile integral, distal latency, or intrabolus pressure. The proportion of narcotic use in patients with normal manometry versus abnormal manometry was not significantly different.

*Conclusions*. In patients presenting with dysphagia and/or chest pain as the primary complaint:

1. Regular narcotic use and female gender are predictors of increased LES mean basal pressure
2. PPI use is associated with lower LES mean basal pressure, however it is difficult to ascertain whether this might be secondary to underlying reflux versus the medication itself.
3. Anticholinergic use is associated with more failed swallows (assessed per the Chicago classification).

*Funding Agencies: *None

# Sub article: A321: The Serine Protease Trypsin Evokes Long Term Hyperexcitability in Nociceptive DRG Neurons via Endosomal Signaling

**Authors:** N. Jiménez-Vargas, C. Lopez Lopez, J. Jaramillo Polanco, B. Nigel, S. Vanner

**Affiliations:** ^321.1^Queen's UniversityKingstonONCanada; ^321.2^Monash UniversityMolbourneVICAustralia

*Background*. Intestinal proteases are important mediators of increased pain signaling in irritable bowel syndrome (IBS). Our previous studies have shown that activation of protease activated receptor 2 (PAR2) on nociceptive (pain sensing) dorsal root ganglia (DRG) neurons evoke long term hyperexcitability but the mechanisms of this sustained pain signaling are unclear. Recent studies show that trypsin activates canonical PAR2 signaling causing endocytosis whereas elastase and cathepsin S act at non-canonical sites, neither of which result in endocytosis.

*Aims*. We hypothesized that the sustained nociceptive signaling observed by trypsin PAR2 activation results from canonical endosomal signaling and exploited the known differences in trypsin PAR2 activation compared to elastase and cathepsin S to test this hypothesis.

*Methods*. DRG neurons (T9–T13) from C57BL/6 mice were pre-incubated with Trypsin (50 nM, 10 min), elastase (10 U/mL (390 nM), 1 h) or cathepsin S (500 nM, 1 h) then washed with F12 media. We measured neuronal excitability by perforated patch-clamp, recording changes in rheobase (minimum current to fire action potential) and action potential discharged at twice rheobase immediately after washing with F12 media (time 0) or 30 min later (time 30). Dynamin (30 *μ*M dyngo4a) and clathrin (30 *μ*M pitstop2) inhibitors of endocytosis were applied 15–30 min before the agonists. Two way ANOVA and post hoc Bonferroni tests were used to analyze the data.

*Results*. Trypsin, elastase, and cathepsin S increased DRG neuronal excitability at time 0 and evoked a sustained response (time 30) (Table 54 [Table tab55]). The inhibitors of endocytosis had no effect at time 0 but blocked the long term excitability of trypsin (dyngo4a and pitstop2) but not cathepsin S or elastase (dyngo4a).

*Conclusions*. These data demonstrate that the sustained nociceptive signaling mediated by trypsin is dependent on endosomal signaling. This intracellular signaling could provide a novel therapeutic target in disorders such as IBS. In contrast, elastase and cathepsin S also evoked sustained signaling but was not dependent on endocytosis, implying that non-canonical signaling could also be important in some disorders.

*Funding Agencies: *CIHR

# Sub article: A322: Distinct Intracellular Pathways Mediate GDNF-Induced Neuronal Survival and Axonal Outgrowth in the ENS

**Authors:** N. Gharib, R. Yeung, S. Lourenssen, M. Blennerhassett

**Affiliations:** ^322.1^Queen's UniversityKingstonONCanada; ^322.2^Queens UnivKingstonONCanada

*Background*. The neurotrophin glial cell line-derived neurotrophic factor (GDNF) is essential for the survival and development of the enteric nervous system (ENS) both during prenatal and post-natal life. In the ENS, GDNF signals through the RET/GFR*α*1 receptor complex but little is known about downstream signalling cascades.

*Aims*. Since they may mediate discrete aspects of ENS structure and function, we examined the roles in GDNF-dependent pathways in the post-natal ENS.

*Methods*. An established in vitro co-culture model of myenteric neurons, intestinal smooth muscle and glia was obtained by dissociation of the neonatal rat neuromuscular layer. While GDNF is initially essential for survival, the addition of GDNF to established co-cultures induces stimulation of axonal outgrowth. The roles of molecular intermediates were established using western blotting for phosphorylated isoforms and immunocytochemistry for cellular correlates. For the latter, antibodies detecting HuD-positive neuronal cell bodies and SNAP-25 labelled axons were used for quantification of neuron and axon numbers, while H333258 was used as a pan-cellular marker.

*Results*. The importance of Src kinase as a RET adaptor protein was studied by application of the Src inhibitor PP2 to co-cultures prior to addition of GDNF (50 ng/mL). Initial evaluation showed a concentration-dependency of effect, where PP2 at 1 *μ*M decreased axon number in established co-cultures to 25 ± 7% (*n* = 3) of the control condition (GDNF 50 ng/mL). There was no non-specific cytotoxicity for PP2 ≤1 *μ*M. In studies of acute survival, 1 *μ*M PP2 decreased neuronal survival to 67 ± 12% (*n* = 4) of control without effect on non-neuronal cells, again indicating a selective dependency of GDNF signalling on Src activation. Similar testing of the roles of p38MAPKinase and JNK signalling showed divergent outcomes. Inhibition of p38MAPKinase with SB203580 (20 *μ*M) reduced both GDNF-induced survival to 47 ± 7% (*n* = 3) of GDNF alone, and GDNF-induced axonal outgrowth to control levels (ie, similar to medium alone). However, inhibition of JNK phosphorylation with SP600125 (20 *μ*M) did not affect GDNF-induced neuronal survival but blocked GDNF-induced axonal extension in established co-cultures.

*Conclusions*. Elsewhere, Src activation occurs immediately proximal to Ret phosphorylation. We conclude that Src kinase activation may act similarly in the ENS, existing at the top of a signaling hierarchy of GDNF-mediated outcomes. In this, p38MAPKinase may be pre-eminent over intermediates with limited actions (i.e., JNK). Distinct molecular pathways in GDNF-mediated outcomes may identify molecular targets for interventions that support the ENS in diverse disease states.

*Funding Agencies: *NSERC

# Sub article: A323: Epigenetic Regulation of Gene Expression in Proliferating Intestinal Smooth Muscle Cells

**Authors:** Q. Bonafiglia, M. Blennerhassett

**Affiliations:** ^323.1^Queen's UniversityKingstonONCanada; ^323.2^Queens UnivKingstonONCanada

*Background*. Intestinal inflammation causes proliferation of intestinal smooth muscle cells (ISMC), contributing to the thickened intestinal wall and the dysmotility symptoms observed in inflammatory bowel disease (IBD). In vitro, prolonged proliferation of ISMC results in loss of their normal contractile phenotype. This is characterized by decreased expression of glial cell-line derived neurotrophic factor (GDNF), as well as smooth muscle marker proteins *α*-smooth muscle actin (SMA) and SM22-*α* (SM22). GDNF, secreted by proliferating ISMC, is essential for survival and growth of enteric neurons in the adult intestine. Consequently, loss of contractile proteins and GDNF expression will contribute to the cellular basis of intestinal dysfunction in IBD.

*Aims*. The mechanism behind loss of normal ISMC contractile phenotype upon repeated proliferation is currently unknown, and we hypothesized that epigenetic alterations to gene expression may underline these progressive changes.

*Methods*. This was investigated in cultured rat circular colonic smooth muscle cells (CSMC), studied as either low passage (P1–3) or high passage (P10–15) cell lines. All cultures were passaged uniformly at confluence. Epigenetic change was primarily approached through the use of agents that reverse histone acetylation (trichostatin) or DNA methylation (5-azacytidine), used at concentrations that did not cause cytotoxicity. Outcomes were evaluated using western blotting, immunocytochemistry, RT-PCR and proliferation and functional assays.

*Results*. Inhibition of DNA methyltransferases with 5-azacytidine (AZA; 2.0 *μ*M) increased expression of SMA (273 ± 151%; *n* = 3) and SM22 expression (130 ± 62%; *n* = 2) in high passage CSMC. However, inhibiting histone deacetylase complexes with trichostatin (TSA; 0.1 *μ*M) decreased SMA expression (−74 ± 18.5%; *n* = 2) but increased SM22 expression (419 ± 242%; *n* = 2), suggesting variable responses to TSA intervention among different cell lines. Furthermore, media isolated from AZA-treated high passage CSMC showed increased support of neuronal survival and axonal outgrowth, evidence for an increase in GDNF expression.

*Conclusions*. We conclude that histone deacetylation and DNA methylation occurred in repeated passage CSMC, and that epigenetic changes to gene expression may account for their altered phenotype. The outcomes of TSA and AZA treatment suggest that modulation of an epigenetic component of smooth muscle phenotype could be a novel therapeutic option in treating dysmotility in IBD patients.

*Funding Agencies: *NSERC

# Sub article: A324: Correlating Colonic Motor Patterns with Luminal Pressure Waves in High Resolution Colonic Manometry

**Authors:** J. Chen, Z. Yang, S. Parsons, J. Huizinga

**Affiliations:** ^324.1^McMaster UniversityHamiltonONCanada; ^324.2^Renmin Hospital of Wuhan UniversityWuhanChina

*Background*. High Resolution Colonic Manometry (HRCM) is developing in several countries and its clinical significance is undergoing renewed debate. To understand the mechanisms behind the propulsive and non-propulsive pressure patterns, it is important to understand the motor patterns, the contractile activities of the circular muscle, that are associated with the pressure changes.

*Aims*. Our aim was to correlate motility with pressure changes using the intact colon of the rabbit.

*Methods*. We combined high-resolution spatio temporal mapping (colonic diameter changes along the 3-teaniated proximal colon of the rabbit) with high-resolution manometry (41 sensors 0.25 cm apart).

*Results*. Five motor patterns and associated luminal pressures were analyzed: (1) high amplitude circular muscle contractions with a propagating velocity of 0.35 cm/s were associated with propagating pressure waves with amplitudes of >100 mmHg at a similar velocity. (2) Fast propagating contractions with a velocity of 4–6 cm/s were associated with pan-colonic simultaneous pressure waves with amplitudes of 20–30 mmHg. (3) Low amplitude circular muscle contractions that propagated in both directions, called ripples, when propagating without interruption at 2 cm/s or higher, caused simultaneous pressure waves. When ripples became chaotic, that is, when propagation directions changed very often due to interactions with haustral folds, there were no measurable pressure changes. (4) Rhythmic haustral boundary contractions (0.5 cpm), which form colonic haustra, had a very low velocity of 0.013 cm/s. This dominant pattern in spatio-temporal diameter maps was not readily identifiable with current HRCM. (5) The colon exhibited abundant isolated local contractions, particularly strong at the haustral boundaries, resulting in local pressure transients whose strength was related to the contraction amplitude.

*Conclusions*. In summary, the velocity of propulsive motor patterns is a key factor in determining the features of the luminal pressure waves recorded with HRCM. Contractions with propagation velocities between 0.1–2 cm/s present as propulsive pressure waves; velocities above this range present as pan-colonic simultaneous pressure waves. Therefore, it should be discouraged to use “contraction" terminology in HRCM, such as HAPCs (high amplitude propagating contractions). Contractions with velocities below 0.1 cm/s are not measurable by HRCM at standard sensitivity. Local contractions appear to be faithfully registered as local pressure transients. In conclusion, there is no simple relationship between motor patterns and pressure activity. Understanding this relationship is essential for accurate interpretation of HRCM.

*Funding Agencies: *CIHR

# Sub article: A325: Inducible NO Synthase (iNOS)-Derived NO Mediates Impaired Excitability of Vagal Afferents in Obesity

**Authors:** S. Park, M. Beyak

**Affiliations:** ^325.1^Queen's UniversityKingstonONCanada

*Background*. We have demonstrated that when obesity is induced with a high fat diet, NO plays an important role in reduction in intestinal vagal afferent excitability (Park and Beyak DDW 2015). Inducible NO synthase (iNOS) is upregulated in response to obesity.

*Aims*. The aim of the present study was to examine if iNOS is involved in impairement of vagal afferent excitability in obesity.

*Methods*. C57Bl6J mice were rendered obese by feeding a diet of 60% calories from fat (HFF) for 12–16 weeks; mice fed 10% calories from fat (LFF) were used as controls. Nodose ganglion neurons (NGN) were dissociated. Patch clamp recordings were performed 18–24 h post-dissociation. NO was measured using a Nitrate/Nitrite fluorometric Assay kit. Serum was collected from LFF and HFF mice with/without L-NIL IP injection (10 mg/kg).

*Results*. Serum NO content was determined by nitrite/nitrate accumulation via a fluorometric assay. Total nitrite was significantly higher in HFF than LFF serum (29.0 ± 4.1 (HFF) versus 15.9 ± 0.7 (LFF), ^⁎⁎^0.0062, *n* = 9). A selective iNOS synthase inhibitor, L-NIL reduced nitrite in HFF, not in LFF serum (21.7 ± 6.2 (HFF) versus 10.9 ± 1.2 (HFF + N-NIL), *n* = 3). L-NIL in vitro increased the excitability of NGN from HFF mice. L-NIL (5 mM, 30 min) significantly decreased rheobase (112.1 ± 14.6 pA (*n* = 14, HFF) versus 67.0 ± 5.9 pA (*n* = 10, HFF + L-NIL) ^⁎^ *p* = 0.0206) and significantly increased number of APs at twice rheobase (1.4 ± 0.1 (*n* = 14, HFF) versus 2.2 ± 0.4 (*n* = 10, HFF + L-NIL) ^⁎^ *p* = 0.0295). Rheobase and number of APs at twice rheobase were not significantly changed by L-NIL in LFF. Another selective iNOS synthase inhibitor, 1400 W increased the excitability of NGN from HFF mice. 1400 W (100 nM, 30 min) significantly decreased rheobase (112.1 ± 14.6 pA (*n* = 14, HFF) versus 53.3 ± 5.8 pA (*n* = 9, HFF + 1400 W) ^⁎⁎^ *p* = 0.0053) and significantly increased number of APs at twice rheobase (1.4 ± 0.1 (*n* = 14, HFF) versus 2.9 ± 0.8 (*n* = 9, HFF + 1400 W) ^⁎^ *p* = 0.0220). 1400 W significantly increased input resistance (285.3 ± 32.8 MΩ (*n* = 14, HFF) versus 515.7 ± 44.5 MΩ (*n* = 9, HFF + 1400 W) ^⁎⁎⁎^ *p* = 0.0004) in HFF neurons. 1400 W did not make a significant change in electrophysiological parameters in NGNs from LFF mice.

*Conclusions*. Diet-induced obesity enhanced iNOS-NO activity impairing vagal afferent excitability. We suggest that the iNOS-NO pathway may be one of the important mechanisms for vagal afferent hyposensitivity in obesity.

*Funding Agencies: *CIHR

# Sub article: A326: Mechanosensitive K+ Conductance Negatively Regulates Membrane Potnetial in Vagal Afferent Neurons during Mechanical Stress: Role of Nitric Oxide (NO)

**Authors:** S. Park, M. Beyak

**Affiliations:** ^326.1^Queen's UniversityKingstonONCanada

*Background*. Mechanical distension of the gut can be sensed and transduced to the CNS through gastrointestinal vagal afferents, resulting in satiety and other critical GI reflexes. Therefore understanding of the factors regulating detection of mechanical stimuli is important. Our previous study reported that cross-talk exists between mechanosensitive cation channels (TRPV4) and K^+^ channels to balance membrane excitability in vagal afferent neurons.

*Aims*. This study was designed to understand how NO might affect K^+^ conductances regulating mechanosensation.

*Methods*. Hypotonic solution (200 mosm) was used as mechanical stimulus. Osmolality was altered by changing mannitol concentration, while maintaining constant electrolyte composition. Whole cell current clamp recordings from mouse nodose ganglion neurons (cell bodies of vagal afferents) were perfomed.

*Results*. Membrane potentials were measured when external isotonic solutions were changed to hypotonic. Potential was depolarized in 5 min upon hypotonic solution, then hyperpolarized (recovered to resting and more hyperpolarized) after 10 min in hypotonic solution (−50.6 ± 1.7 mV (iso), −25.6 ± 2.6 mV (hypo-5 min), −63.9 ± 2.8 mV (hypo-10 min), *n* = 7). hypotonic solution. Resting membrane potentials (mV) were significantly hyperpolarized from −56.9 ± 1.2 (iso) to −60.2 ± 1.2 (hypo) (*p* = 0.0053^⁎⁎^ *n* = 19, resp.). Rheobase (pA) was significantly increased (iso, 62.6 ± 4.6 versus hypo, 78.9 ± 6.4, *p* = 0.0049^⁎⁎^ *n* = 19, resp.) and the number of APs at 2x Rheobase was higher in isotonic solution (iso, 2.5 ± 0.5 versus hypo, 1.8 ± 0.4  *n* = 19, resp.). The change of potentials was diminished in the presence of NO synthase inhibitor, L-NNA (−50.4 ± 1.8 mV (iso), −47.6 ± 2.3 mV (hypo-5 min), −46.3 ± 3.9 mV (hypo-10 min), *n* = 8). The change of potential showed a smaller and slower depolarization, no recovery phase in the presence of ODQ, sGC (soluble guanylyl cyclase, intracellular receptor for NO) inhibitor (−50.4 ± 1.8 mV (iso), −47.6 ± 2.3 mV (hypo-5 min), −46.3 ± 3.9 mV (hypo-10 min), *n* = 8).

*Conclusions*. Mechanical stimulation of vagal afferent cell membranes induces hyperpolarization due to activation of the NO-cGMP pathway. NO-sGC- K^+^ channels pathway may play an important role in negative feedback regulation under mechanical stress in vagal afferent neuron.

*Funding Agencies: *CIHR

# Sub article: A327: Deoxycholic Acid Activation of Proximal Colon Afferent Nerve

**Authors:** E. Villalobos-Hernandez, C. Barajas-Lopez, D. Reed

**Affiliations:** ^327.1^Queens UniversityKingstonONCanada; ^327.2^Instituto Potosino de Investigacion Cientifica y TecnologicaSan Luis PotosiMexico

*Background*. Food and stress are two factors associated with irritable bowel syndrome (IBS). In a subpopulation of IBS patients, bile acids are increased in the colon and may trigger abdominal discomfort. However, little is known about activation of colonic sensory nerves by bile acids nor the interaction of bile acids and stress.

*Aims*. The aim of this study was to examine activation of afferent nerves innervating the colon by deoxycholic acid (DCA), a major secondary bile acid, and whether this activation could be modulated by stress hormones.

*Methods*. An in vitro preparation of the C57BL/6 mouse proximal colon was employed to perform extracellular recordings of nerves en route to the superior mesenteric/coeliac complex. DCA (100 *μ*M–1 mM) was perfused into the lumen in the absence or presence of atropine 10 *μ*M plus hexamethonium 200 *μ*M, as well as the stress hormones; epinephrine 5 nM and corticosterone 1 *μ*M. Afferent nerve activation by a ramp distension protocol was examined both before and after DCA. Discriminated units, based on size and shape were considered as responders if the firing rate increased 25% above baseline. Data are expressed as mean ± SEM.

*Results*. Following intraluminal application of DCA, 1/8 units responded to 100 *μ*M, 3/11 units responded to 300 *μ*M, and 6/15 units responded to 500 *μ*M. In another series of experiments, ramp distension was applied before and during intraluminal application of DCA 1 mM. In these experiments, 11/23 units responded to DCA before distension while 17/23 units had increased firing after distension. The increased firing rate in responding units was significantly increased compared to time matched controls (164.4 ± 51.84% versus −7.77 ± 5.64%; *p* < 0.05). Using the same protocol in the presence of atropine and hexamethonium, only 1/16 units responded to DCA before distension but 13/16 units had increased firing after distension. This increase did not reach statistical significance compared to DCA alone (57.97 ± 8.53% versus 164.4 ± 51.84%, *p* = 0.09). DCA perfusion in the presence of stress hormones significantly increased firing in responding units compared to DCA (131.5 ± 44.99% versus 57.97 ± 8.53%, *p* < 0.05). Response to mechanical distension analyzed in 10 mmHg increments to a maximum of 60 mmHg showed no difference in the presence of DCA compared to control response when corrected for baseline firing rate. DCA plus stress hormones also did not change mechanical activation (*p* > 0.05).

*Conclusions*. This data suggests that DCA can increase firing of proximal colon afferent fibers independent of changes in motility. DCA induced activation is augmented by stress hormones. This increase excitation of afferent fibers was not accompanied by changes in mechanical activation.

*Funding Agencies: *Queen's University, Kingston General Hospital

# Sub article: A328: NO Enhances BK Conductance in Vagal Afferent Neurons and in Diet Induced Obesity

**Authors:** S. Park, M. Beyak

**Affiliations:** ^328.1^Queen's UniversityKingstonONCanada

*Background*. We have demonstrated that when obesity is induced with a high fat diet, there is a resultant reduction in intestinal vagal afferent excitability, in which NO plays an important role (Park and Beyak DDW 2015).

*Aims*. The aim of the present study was to examine the large-conductance Ca^2+^-activated K^+^ conductance (BK) regulated by NO in vagal afferents and to examine how diet induced obesity impacts this signaling pathway.

*Methods*. Nodose ganglion neurons (NGN) were dissociated from C57Bl6J mice. C57Bl6J mice were rendered obese by feeding a diet of 60% calories from fat (HFF) for 12–16 weeks; mice fed 10% calories from fat (LFF) were used as controls. Patch clamp recordings were performed 18–24 h post-dissociation. To evaluate BK conductance* After*-*hyperpolarization* (AHP) were analyzed, and the specific BK channel blocker, iberiotoxin was used.

*Results*. During an action potential, membrane depolarization and Ca^2+^ entry through Ca^2+^ channels activate BK channels, which help to terminate the action potential, produce fast AHP. Iberiotoxin (10 nM), BK channel inhibitor, significantly decreased the time of upward phase in AHP (AHP peak to recovery, control; 90.5 ± 10.5, IbTx; 68.4 ± 6.0 msec, paired *t*-test ^⁎^ *p* = 0.0394, *n* = 7), reflecting BK conductance force against the upward phase in AHP. NO donor, SNP (100 mM, 30 min pre-incubation) increased the time of upward phase in AHP (control; 80.3 ± 4.9, SNP; 126.4 ± 12.2 msec, unpaired *t*-test ^⁎^ *p* = 0.0457, *n* = 7, each). The effect of SNP was blocked by IbTx (SNP; 105.3 ± 11.8, SNP + IbTx; 80.8 ± 8.5 msec, paired *t*-test ^⁎⁎^ *p* = 0.0066, *n* = 11). Substrate for NO synthase, L-arginine significantly increased the time of upward phase in AHP (control; 80.3 ± 4.9, L-Arg; 107.1 ± 10.4 msec, unpaired *t*-test ^⁎^ *p* = 0.0394, *n* = 11, 13, resp.). NO synthase inhibitor, L-NNA (0.1 mM, 30 min) significantly decreased the time of upward phase in AHP (L-NNA; 51.3 ± 5.4 msec, unpaired *t*-test ^⁎⁎⁎^ *p* = 0.0009, *n* = 10). There were no significant changes in AHP phase with L-NIL (5 mM, 30 min), a selective inhibitor of iNOS (L-NIL; 75.9 ± 6.5 msec, NS, *n* = 13). However, L-NIL (5 mM, 30 min) significantly decreased the time of upward phase in AHP in neurons from obese mice (HFF; 174.5 ± 24.1, HFF + L-NIL; 73.4 ± 3.6 msec, unpaired *t*-test ^⁎⁎⁎^ *p* < 0.0001, *n* = 9, 13, resp.) (LFF; 114.9 ± 9.2, LFF + L-NIL; 84.6 ± 11.6 msec, unpaired *t*-test ns, *n* = 9, 8, resp.).

*Conclusions*. NO increased BK conductance which negatively regulates vagal afferent excitability under normal physiology and in obesity. iNOS-derived NO opens BK channels in nodose neurons from obese mice.

*Funding Agencies: *CIHR

# Sub article: A329: Sacral Nerve Stimulation in the Treatment of Fecal Incontinence: The Experience of a Canadian Centre

**Authors:** C. Parker, D. Elterman, L. Liu

**Affiliations:** ^329.1^University of TorontoTorontoONCanada; ^329.2^University Health NetworkTorontoONCanada

*Background*. Patients with fecal incontinence (FI) have a poor quality of life as it negatively impacts social function. Conventional therapy involves dietary and behavioural modifications, pelvic floor physiotherapy, pharmacotherapy and biofeedback. However, some patients fail to respond to these therapies. Surgical options for FI have variable outcomes and, often, carry significant morbidity. Sacral nerve stimulation (SNS) is a less invasive therapeutic option for these patients. This involves the implantation of an electrical stimulator that acts on the sacral nerve to improve continence and pelvic floor function. This treatment of FI has been widely adapted in Europe and the US but is relatively new in Ontario, Canada.

*Aims*. To review the experience of the use of SNS in the treatment of FI at a quaternary centre in Ontario, Canada.

*Methods*. A retrospective chart review was performed on patients with FI who either had an SNS implanted or who passed a screening exam and are awaiting device implantation. The medical records for these patients were reviewed. Data regarding their demographic information, co-morbidities, FI symptoms, associated urinary symptoms and, in those who received the SNS implant, response to therapy were collected. Descriptive statistics were used to analyze the data.

*Results*. 7 patients received the SNS implant and 3 patients had passed a screening exam and are currently awaiting the implant. All 10 patients were female and the average age was 61.8 (SD = 9.8). 7 patients (70%) had 1 or more vaginal deliveries and 3 (30%) had traumatic deliveries (described as required forceps or vaginal repair). 4 patients (40%) had previous ano-rectal surgery. 5 patients (50%) had associated urge urinary incontinence and 1 (10%) had stress urinary incontinence. 5 patients (50%) had daily FI, 2 (20%) had weekly FI, 1 (10%) had occasional FI but significant urinary symptoms, and 2 (20%) had an unknown frequency of FI based on the chart review. 3 patients (30%) had a sense of urgency associated with FI. 6 of the 7 patients who received an SNS implant have had follow-up post implantation. All of these patients experienced at least a 50% reduction in episodes of urgency and fecal incontinence and 3 patients had a greater than 90% response in these symptoms.

*Conclusions*. This study examines the patients who have received SNS for the treatment of FI unresponsive to conventional therapy in a quaternary Canadian centre. All patients, albeit a small number, who had returned for follow-up subjectively experienced an improvement in FI. This data will be used to develop a robust clinical program for the treatment of medically refractory FI and to design new research initiatives that evaluate the role and mechanism of action of SNS in the treatment of FI.

*Funding Agencies: *None

# Sub article: A330: Is Jackhammer Esophagus Clinically Easy to Recognize?

**Authors:** W. Zhu, T. Ngo, E. Neshkova, M. Bouin

**Affiliations:** ^330.1^St*-*Luc Hospital—CHUMMontrealQCCanada

*Background*. Jackhammer esophagus (JE) is an esophageal hypercontractility motor disorder first described in 2012. The diagnosis is made by high-resolution manometry according to the criteria of the Chicago classification. An association with dysphagia has been reported but the clinical expression of JE remains unclear.

*Aims*. The primary objective of this study was to identify the gastrointestinal symptoms associated with JE. The secondary objective was to determine the relevance of standard digestive investigations in the diagnostic work-up.

*Methods*. This was a retrospective study performed between January and June 2015 at the Laboratory of Neurogastroenterology of Saint-Luc Hospital. All patients with a diagnosis of JE using the Chicago Classification were included. The data collected from patients' records were as follow: age, gender, digestive symptoms, proton pump inhibitors use, esophagogastroduodenoscopy (EGD), esophageal manometry and esophageal pH monitoring results.

*Results*. Twelve patients were included (mean age 56 ± 13 years; 83% women). Digestive symptoms that led to esophageal manometry were dysphagia (58%, *n* = 7), epigastric pain (58%, *n* = 7), heartburn (33%, *n* = 4) and retrosternal pain (17%, *n* = 2). EGD reports were analyzed in 92% of patients: 73% were normal and 17% showed only a hiatal hernia without complication. Esophageal biopsies were done in four patients (33%); all were normal but one showed lymphocytic exostosis. Apart from manometric criteria specific to JE, esophageal manometry has also shown the following abnormalities: (1) a relaxation defect of the lower esophageal sphincter in 42% of patients (*n* = 5), (2) a hypertonic lower esophageal sphincter without relaxation defect in one patient, and (3) an absence of peristalsis in one patient. Three patients had 24-hour pH monitoring and gastroesophageal reflux disease was confirmed in one patient. 42% of patients were on proton pump inhibitors at the time of manometry.

*Conclusions*. Our study showed that there was a female predominance for JE. The digestive symptoms associated with JE varied and could be dysphagia, pain or gastroesophageal reflux disease. EGD was usually normal and did not orient the diagnosis while manometry revealed a dysfunction of the lower esophageal sphincter in almost half of the patients.

*Funding Agencies: *None

# Sub article: A331: Dietary Levels of Monosaccharides and Saturated Fat Are Higher in Patients with Endoscopically Active versus Quiescent Inflammatory Bowel Disease

**Authors:** A. Mohammadi, O. Kelly, S. Maltais, N. Khamestan, M. Silverberg

**Affiliations:** ^331.1^Mount Sinai HospitalTorontoONCanada

*Background*. Malnutrition in patients with inflammatory bowel disease (IBD) is well documented and patients often self-report symptoms in response to particular foods. Yet, little is known about the contribution of specific macro/micro nutrient profiles to disease course and behaviour.

*Aims*. This study aimed to determine the association of specific dietary components with endoscopic disease activity in a rigorously phenotyped IBD population.

*Methods*. Patients with confirmed IBD attending for colonoscopic evaluation were included. Patients undergoing colonoscopy completed a 4-day food record diary just prior to the procedure. Endoscopic activity was evaluated using the SES-CD (simple endoscopic score for CD) and Mayo endoscopic subscore for UC. Endoscopic activity was defined as SES-CD >4 or Mayo score > 1. Dietary intake was converted to nutrient components using the ESHA Food Processor software. Blood parameters including albumin, CRP and WCC were obtained on day of colonoscopy. Other pertinent information collected included disease duration, Montreal classification for location and behaviour, current treatment, previous surgery, smoking status, extraintestinal manifestations and family history.

*Results*. 30 IBD patients were included in the analysis (50% female, mean age 35.5 years), 14 with CD and 16 with UC. There were 20 patients (10 CD, 10 UC) with active endoscopic disease and 10 patients (4 CD, 6 UC) with endoscopic remission (inactive). The reported intake of saturated fat was significantly higher in patients with active UC versus inactive UC (36.3 ± 21.6 grams versus 18.5 ± 3.9 grams, *p* = 0.03). The intake of monosaccharides was significantly higher in patients with active CD versus inactive CD (10.8 ± 8.7 grams versus 2.3 ± 3.8 grams, *p* = 0.03). No statistically significant differences were seen in average daily caloric intake between active and inactive groups.

*Conclusions*. Diet substantially varies in IBD patients. A higher dietary intake of saturated fat and monosaccharides prior to endoscopy was associated with more active disease. The composition of the diet may affect disease course and endoscopic activity. These preliminary data will be extended to a larger cohort with account taken for confounding variables in future analyses.

*Funding Agencies: *None

# Sub article: A332: Vitamin D3 Supplementation Increases Susceptibility to Bacteria Induced Colitis

**Authors:** N. Ryz, K. Jacobson, B. Vallance

**Affiliations:** ^332.1^Child and Family Research InstituteVancouverBCCanada

*Background*. Vitamin D deficiency increases the risk of developing inflammatory bowel disease (IBD), a disease characterized by exaggerated immune responses to luminal bacteria. While it is unclear how vitamin D impacts IBD development, it is recognized that vitamin D plays an important role in host defense against pathogenic microbes. However, the mechanisms underlying vitamin D's ability to affect a host's susceptibility to infection is poorly understood.* Escherichia coli* is a pathobiont associated with IBD. Intestinal mucosa associated* E*.* coli* have been observed in greater numbers in patients with IBD compared to healthy controls, and these bacteria have been shown to play a role in driving intestinal inflammation. Since clinically important strains of* E*.* coli* do not colonize mice, researchers rely on the related mouse-specific bacterial pathogen* Citrobacter rodentium* (Cr). Following infection, Cr intimately attaches to epithelial cells lining the gut, resulting in barrier disruption, crypt hyperplasia and a strong Th1/Th17 response. We have previously shown that treating mice with active vitamin D, 1,25(OH)_2_D_3_ increased host susceptibility to Cr by suppressing immune responses. However, the effect of supplemental vitamin D3 (cholecalciferol) on enteric infection is unknown.

*Aims*. The aim of this work is to determine the impact of dietary vitamin D3 (VD3) supplementation on host responses to Cr infection. It is hypothesized that dietary VD3 supplementation is protective against* Cr* infection.

*Methods*. Weanling C57Bl/6 mice were fed either VD3 sufficient (1000 IU) or VD3 supplemented (20,000 IU) diets for 5 weeks, then infected with Cr and sacrificed at day 10 pi. Pathogen burdens were determined by plating tissues. Intestinal tissues were analyzed for damage by histological scoring. Inflammatory mediators were assessed by RT-qPCR.

*Results*. VD3 supplemented mice lost significantly more body weight and carried 40 fold higher bacterial burdens in the ceca, compared to VD3 sufficient mice at day 10 pi. VD3 supplemented mice also carried significantly more culturable Cr in the spleen and liver at day 10 pi, indicating greater and more frequent bacterial translocation to these systemic sites. Histologically, the distal colon of VD3 supplemented mice appeared most damaged, with increased ulceration, hyperplasia and cell sloughing. There was a trend for higher levels of inflammatory mediators TNF-*α*, IL-1*β*, IL-6 and IL-17A in the ceca of VD3 supplemented mice, compared to sufficient mice at day 10 pi.

*Conclusions*. Supplemental vitamin D can increase susceptibility to* Cr* infection. These results may have important implications for patients with IBD who are at risk of enteric infection and supplementing with high levels of vitamin D.

*Funding Agencies: *Vanier, UBC FYF

# Sub article: A333: Effect of Manganese Removal from Home Parenteral Nutrition Solutions on Whole Blood Levels and Magnetic Resonance Imaging of the Brain: A Five-Year Cohort Study

**Authors:** J. Jin, O. Saqui, J. Allard

**Affiliations:** ^333.1^University of TorontoTorontoONCanada; ^333.2^University Health NetworkTorontoONCanada

*Background*. Manganese (Mn) is included in many commercial pre-mixed multiple trace element (TE) additives for home parenteral nutrition (HPN). However, there is a risk of over-supplementation because of inherent levels of Mn due to contamination of many additives. Over-supplementation can produce Mn toxicity with neurologic symptoms and basal ganglia deposits on brain magnetic resonance imaging (MRI) and could theoretically contribute to PN associated liver disease (PNALD).

*Aims*. In 2009 we reported that whole blood Mn levels were above the upper limit of normal (ULN) in a sample of 16 HPN patients with 81% having MRI findings. Subsequently, we removed Mn supplementation from all our HPN patients. We present a 5-year follow-up here.

*Methods*. This is a prospective cohort study on 11 of the 16 patients who remained alive on HPN. All patients had Mn removed from their PN over the 5 years and had yearly monitoring of levels. Eight of these patients had a repeat MRI to compare with the original imaging for resolution of basal ganglia deposits. Patient demography, clinical history and bloodwork were recorded.

*Results*. Five out of 6 patients who initially had elevated Mn levels had normal levels 5 years after Mn was removed from PN. All patients who had Mn levels measured serially had a decrease in levels. The mean percent decrease of Mn was 38.1% (range 10.1%–53.8%). Two patients had Mn levels above the ULN despite the absence of Mn supplementation in their PN prescription. Six out of 8 patients who had repeat MRIs had complete resolution of basal ganglia deposition. Of the 2 patients with persistent MRI abnormalities, both had current Mn levels within the upper limits of normal. There was a trend in the decrease of ALT and ALP but this did not reach significance.

*Conclusions*. Removal of Mn as an additive in HPN solutions resulted in resolution of MRI abnormalities in most patients. Over 5 years, all patients except for one, maintained normal blood Mn levels. Therefore, Mn levels should be monitored regularly and supplementation be individualized.

*Funding Agencies: *None

# Sub article: A334: Patient Pathology and Role of Dietitian in a Combined Outpatient Community Gastroenterology/General Surgery Clinic

**Authors:** T. Bardell, S. Pritchett

**Affiliations:** ^334.1^Queen's UniversityKingstonONCanada

*Background*. Patients present to both gastroenterology and general surgery clinics with diseases for which diet plays an important role in management. Many physicians cannot invest as much time in patient education relating to disease-specific nutrition as they would like. An outpatient specialist clinic may be the ideal setting to involve a dietician in patient care.

*Aims*. To describe the views and knowledge of nutrition for conditions in which diet is important in a combined outpatient community general surgery/gastroenterology clinic. To evaluate the sources of diet-related information that patients use.

*Methods*. A convenience sample of patients was asked to complete a self-administered questionnaire designed to assess patient knowledge and opinion of nutrition as it relates to their condition. Demographic information was collected, and patients were asked to rate their prior knowledge about the role of nutrition in managing their condition.

*Results*. One hundred, fifty-three patients participated in the study between January and June 2015. Nineteen percent had inflammatory bowel disease, 18.3% irritable bowel syndrome, 12.4% chronic diarrhea, 7.1% were gluten intolerant, and 34% were overweight. Of the above conditions, only gluten allergy/sensitivity was predictive of belief that nutrition was important (*p* = 0.02). Sixty-nine percent believed nutrition is important or very important and 6.53% indicated that they believe nutrition plays no role at all. When asked about knowledge of specific dietary advice, only 16 patients believed they were highly educated. Less than half of patients felt that they had adequate time to discuss diet with their specialist (30.7%) or their family physician (37.8%). Of the 21% who went elsewhere for dietary information, the most common sources were Naturopaths (46.4%) and the Internet (21.4%). Thirty percent were very interested in referral to a dietitian.

*Conclusions*. Although two thirds of patients believe that nutrition plays a role in their gastrointestinal-related disease, the majority of patients did not feel that they were highly educated on these specific diets. This patient sample had limited access to nutrition counseling through their family physician and specialist and thus had to seek information through alternative resources such as the Internet, which may be less reliable. Patient acceptance of dietitian referral was good, and incorporating a dietitian in a specialist clinic may be beneficial.

*Funding Agencies: *None

# Sub article: A335: Morphological and Gene Transcription Alterations in the Aged Terminal Ileum

**Authors:** S. Tremblay, G. Grenier, A. Menendez

**Affiliations:** ^335.1^University of SherbrookeSherbrookeQCCanada

*Background*. Aging is frequently characterized by significant changes in gastrointestinal function, often leading to pathologies such as malnutrition, small intestinal bacterial overgrowth and a higher prevalence of infectious or inflammatory diseases and cancers. However the nature, extent and contribution of specific factors to the gastrointestinal dysfunction of old age are largely undefined. A detailed understanding of the molecular alterations occurring in the aged intestinal microenvironment can be helpful for the prevention and treatment of gastrointestinal dysfunction.

*Aims*. The aim of this study is to characterize how the microenvironment of the terminal ileum is modified with age and how those changes impact important elements of intestinal homeostasis.

*Methods*. Cohorts of aged and young mice were studied for the number and composition of the ileal microbiota. Electron microscopy and histological staining were used to determine morphological changes of the ileal epithelium. Global gene expression analyses were done by DNA microarrays and qPCR.

*Results*. Ileal aging associated with changes in major commensal bacterial groups, most notable a local increase of lactose-fermenting enterobacteria, without significant changes in total bacterial numbers. We observed dramatic disruptions of epithelial morphology in the ileum of most aged animals, most marked at the villi tips, suggesting defects on the integrity of the epithelial barrier. Increased numbers of intermediate cells were found in the ileal crypts and along the villi, suggesting inefficient terminal differentiation of the secretory cell lineage in the aged ileum. Nonetheless, goblet cell hyperplasia was evident, along with increased mucin production, and significant upregulation of multiple inflammatory and proliferation markers. Electron microscopy confirmed cellular degeneration of the villi and showed abnormal apical accumulation of intracellular vesicles in enterocytes; also, the presence of atypical secretion granules in the Paneth cells of aged mice. Global gene expression analyses reveal marked alterations in the transcript levels of multiple cellular transporters and a generalized downregulation of genes associated to the metabolism of xenobiotics.

*Conclusions*. Significant morphological and transcriptional changes accompany the aging of the terminal ileum. The data supports the notion that ileal aging is associated with an exacerbated local pro-inflammatory status. The functional implications of these alterations are currently under study.

*Funding Agencies: *NSERC, FQRNT

# Sub article: A336: Nutritional Status on Post-Operative Outcomes in Patients with Inflammatory Bowel Disease Undergoing Colectomy: A Single-Centre Experience

**Authors:** G. Ou, H. Ko

**Affiliations:** ^336.1^University of British ColumbiaVancouverBCCanada

*Background*. Patients with inflammatory bowel disease (IBD) refractory to medical therapy may require surgical intervention. However, these patients are at risk of malnutrition due to poor appetite and inflammation, which can impair wound healing and predispose to infections. Malnutrition is associated with increased in-hospital mortality, but its relationship to post-operative outcomes in IBD patients remains unclear.

*Aims*. To determine the relationship between nutritional status, pre-operative parenteral nutrition (PN) and post-operative outcomes in IBD patients.

*Methods*. A retrospective review was initiated at a university-affiliated tertiary care centre in Vancouver, BC. Patients of age ≥ 19 with IBD undergoing first-time colectomy between October 2010 and September 2015 are considered for inclusion. Nutritional status was assessed by body mass index (BMI), weight change from baseline, and subjective global assessment per registered dietician. Clinical disease activities are rated with partial Mayo Score for ulcerative colitis and Harvey-Bradshaw index for Crohn's disease. Post-operative complication is defined as any adverse event occurring within 30 days of surgery that requires additional medical/surgical intervention.

*Results*. Twenty-five patients with IBD underwent colectomy with ileostomy formation between September 2014 and September 2015, of whom 17 (68%) were male. Mean age was 42.0 ± 17.6 years. Indications included medically refractory disease (*n* = 21, 84%), malignancy (*n* = 3, 6%), and hemorrhagic shock (*n* = 1, 4%). At the time of surgery, 8 patients were deemed to be well-nourished/mildly malnourished, 11 were moderately malnourished, and 4 were severely malnourished (2 unavailable). Mean BMI was 22.3 ± 4.6. Mean drop in weight from baseline was 11.4 ± 10.9%. Thirteen (52%) patients experienced an adverse event post-operatively (4 infections, 5 ileus, 3 hypovolemia, 1 stoma bleeding), of whom 4 (out of 8) were considered to be well-nourished/mildly malnourished, 6 (out of 11) were moderately malnourished, and 3 (out of 4) were severely malnourished. Of the 4 patients who received pre-operative PN, 1 patient developed ileus and 1 developed hypovolemia due to high ostomy output.

*Conclusions*. Poor nutritional status may be associated with higher incidence of post-operative complication. Additional data will be obtained for complete analysis.

*Funding Agencies: *None

## Appendix

For general information for the 2016 Canadian Digestive Diseases Week™ (CDDW™), please visit http://downloads.hindawi.com/journals/cjgh/2016/4792898/CDDW.pdf.

For information about CAHN Education Day on Saturday, February 27, 2016, at Montreal, Quebec, please see http://downloads.hindawi.com/journals/cjgh/2016/4792898/CAHN-Education-Day.pdf.

For information regarding the 5th Canadian Symposium on HCV, Theme “We're not Done Yet: Remaining Challenges in Hepatitis C,” on February 26, 2016, at Fairmont The Queen Elizabeth Hotel, Montréal, QC, please click the following link http://downloads.hindawi.com/journals/cjgh/2016/4792898/5th Canadian Symposium on HCV.pdf.

For maps of the Convention Floor, Executive Level, and Grand Hall Main Lobby and details about CDDW 2016 Exhibit plan, please visit http://downloads.hindawi.com/journals/cjgh/2016/4792898/Maps and exhibit plan.zip.
